# Supplementary material for: A Humanized Yeast Phenomic Model of Deoxycytidine Kinase to Predict Genetic Buffering of Nucleoside Analog Cytotoxicity
Source: Genes (Basel). 2019 Sep 30;10(10):770. doi: 10.3390/genes10100770 (PMC6826991; doi:10.3390/genes10100770)
Supplement: Supplementary file 1 [file genes-10-00770-s001.zip › Additional_Files/Additional_File4_InteractionCytarabine.zip!/Additional_File4_InteractionCytarabine/B - InteractionPlots_Cytarabine_DAmPs.pdf]

YDL227C Scatter RF for L with SD

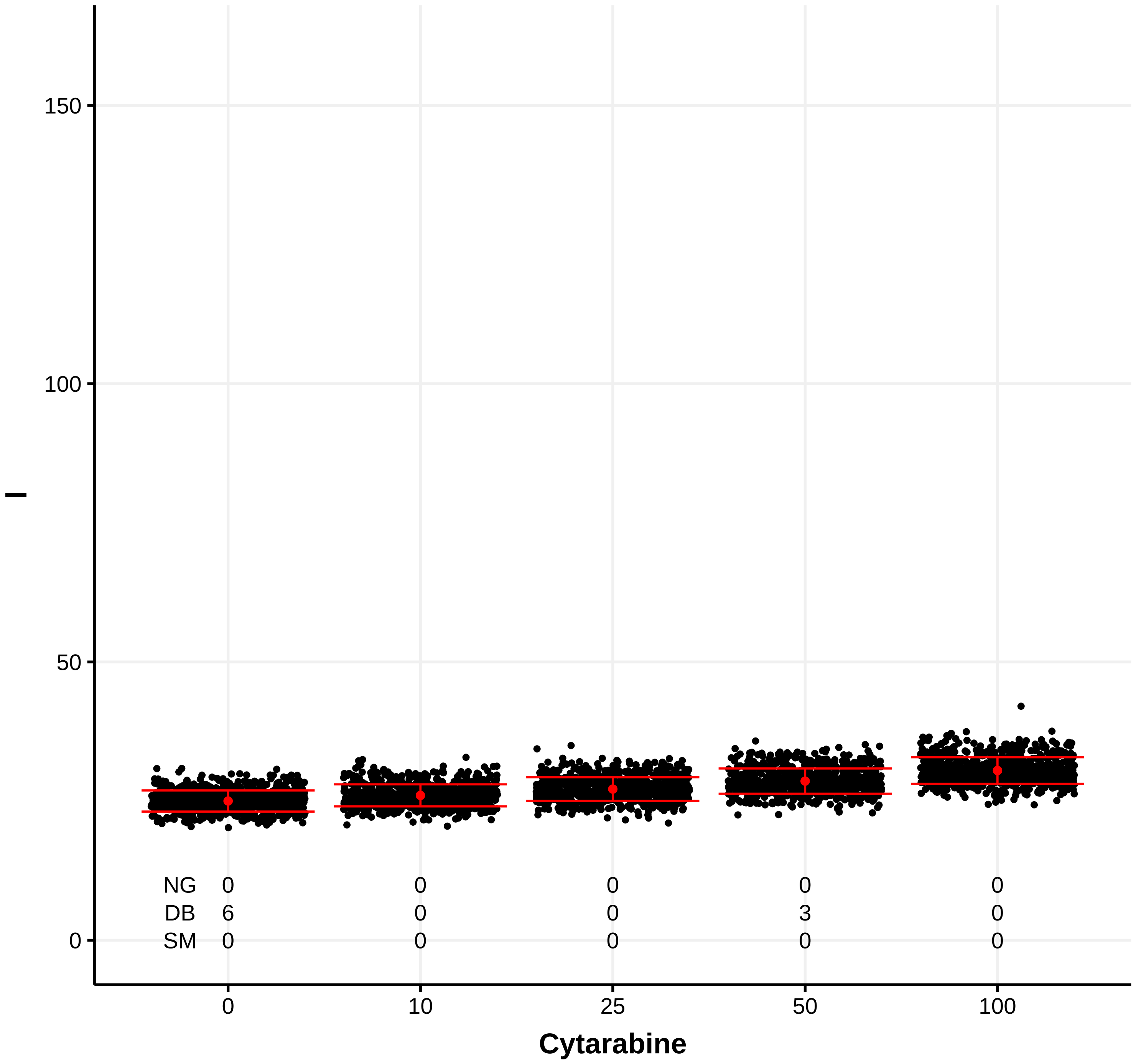

YDL227C Scatter RF for K with SD

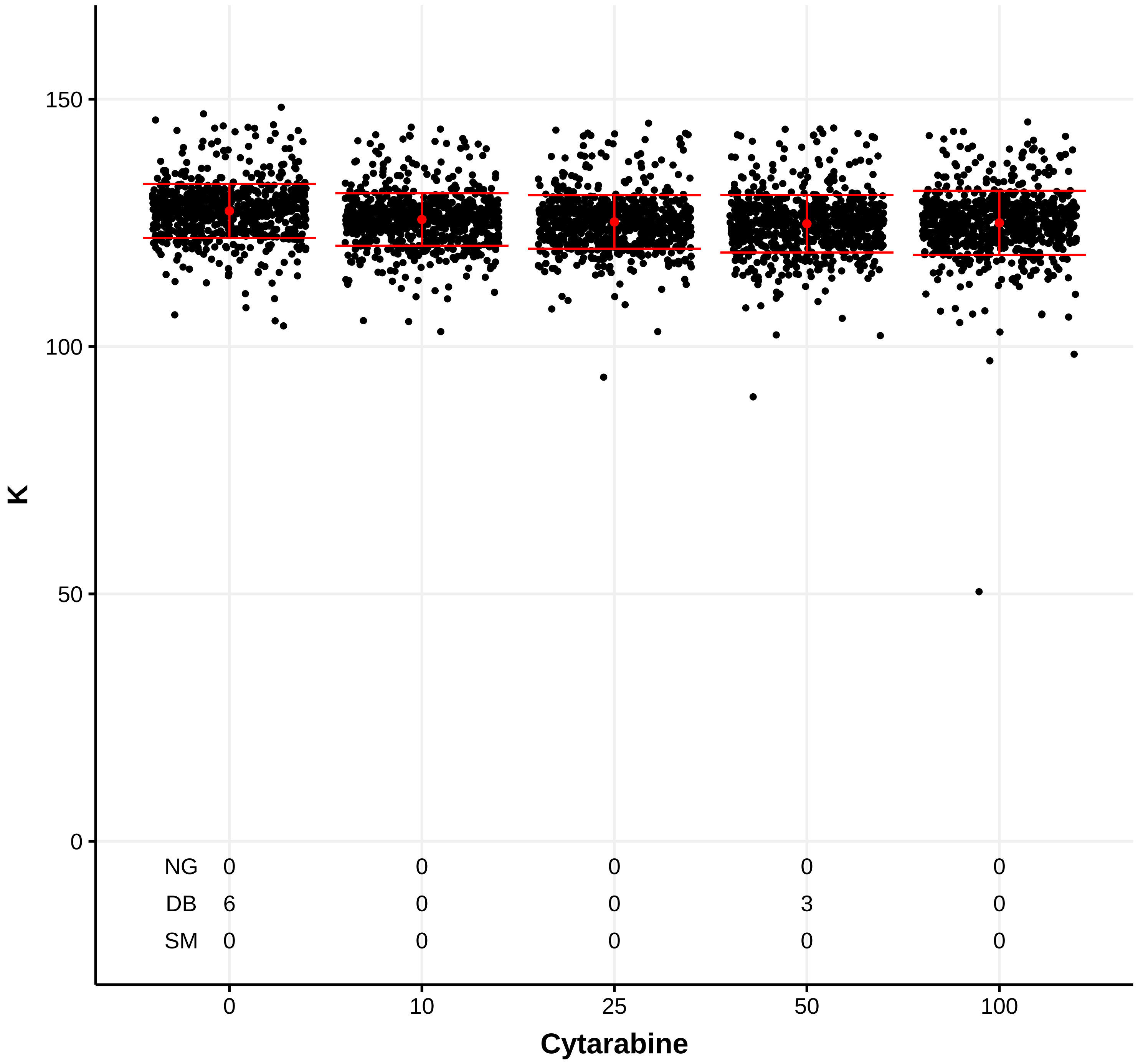

YDL227C Scatter RF for r with SD

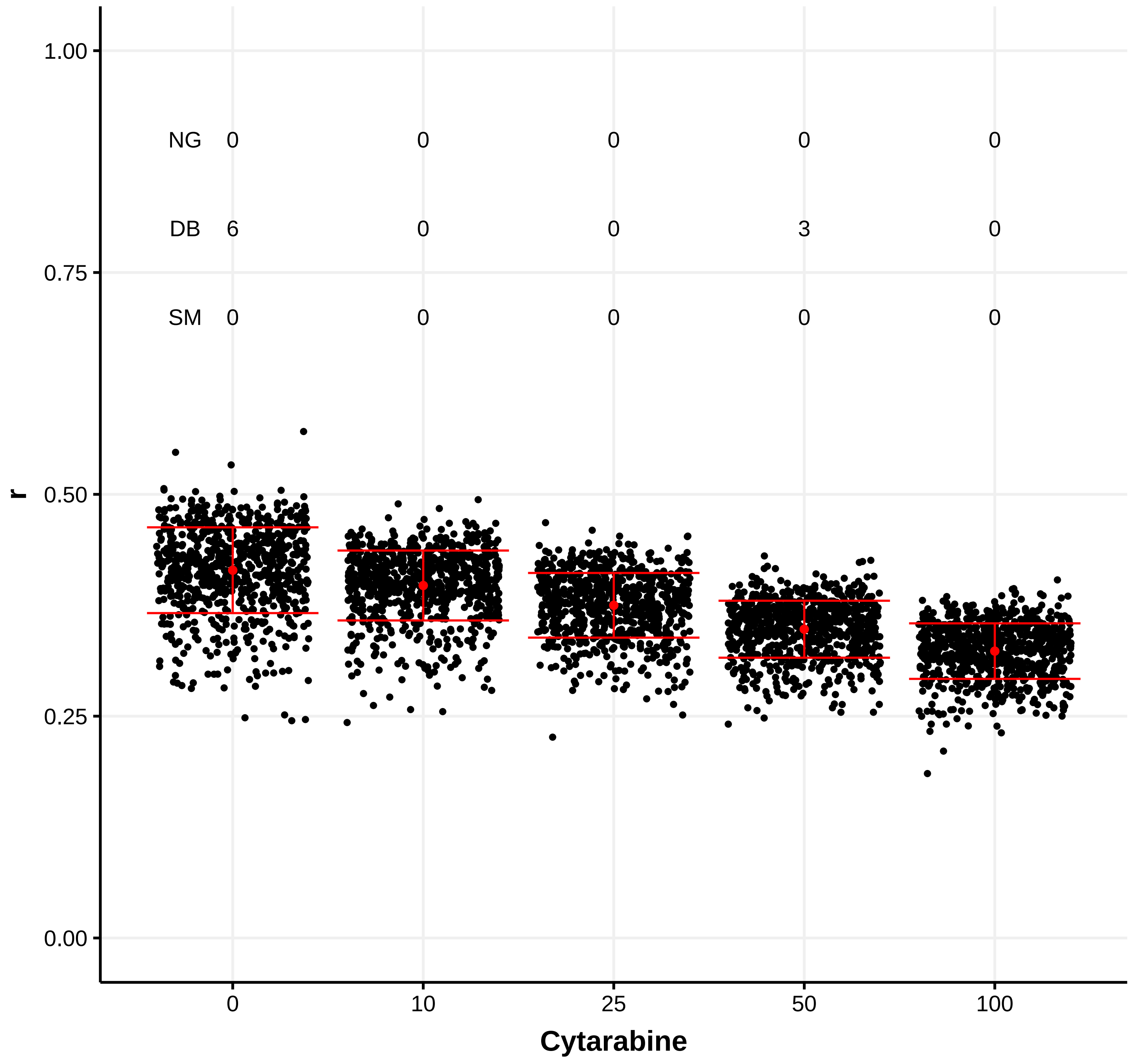

YDL227C Scatter RF for AUC with SD

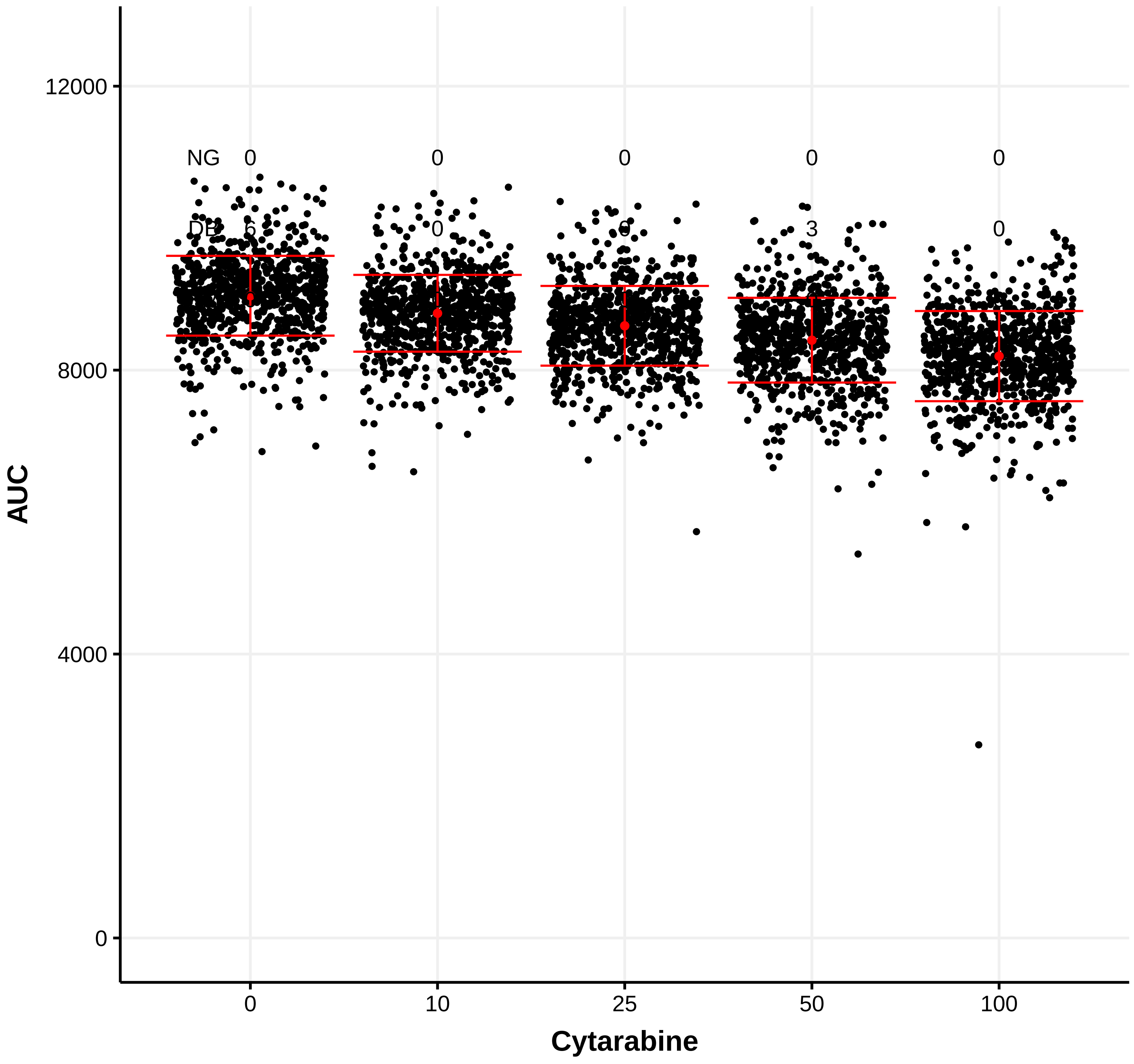

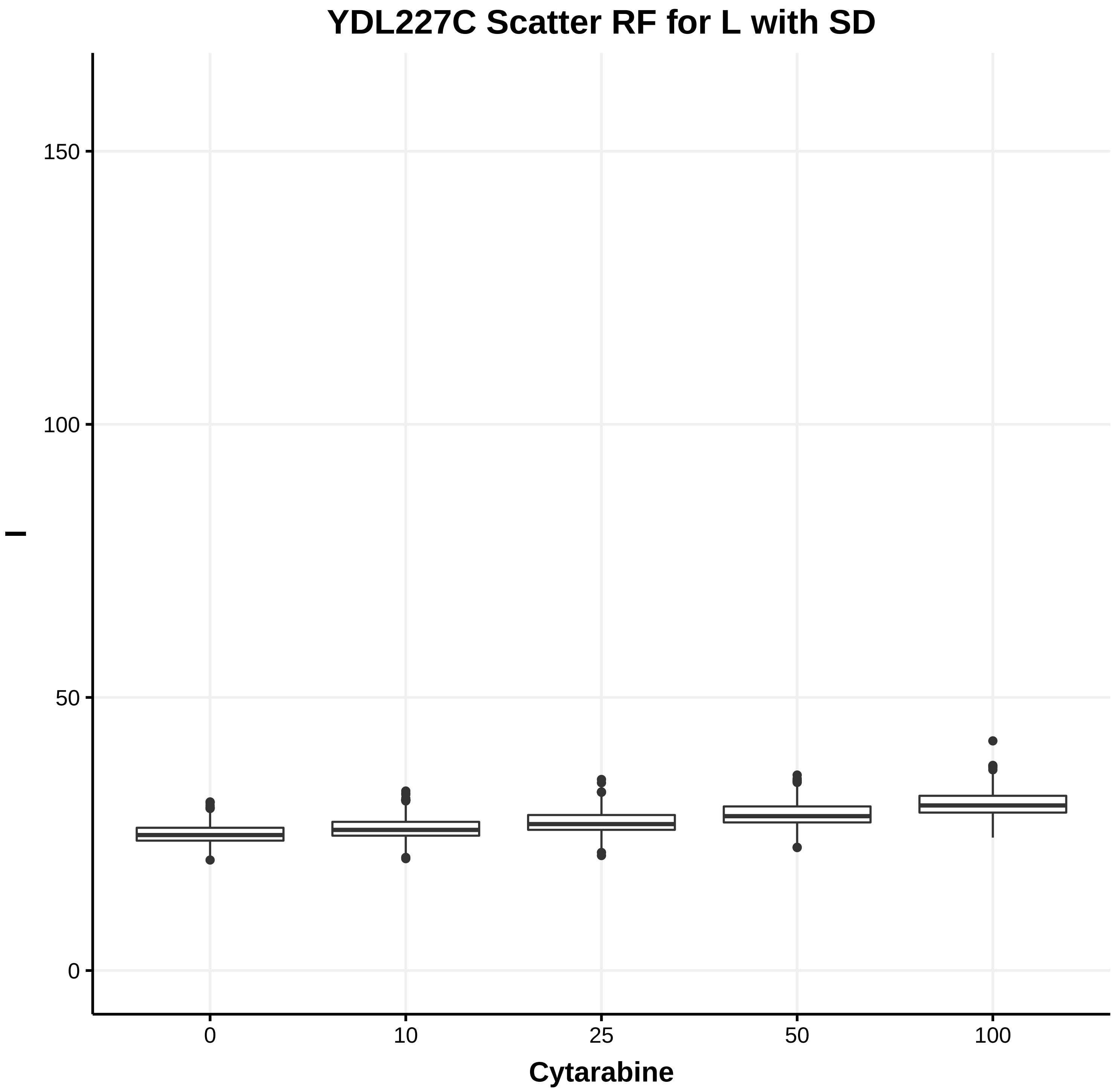
YDL227C Scatter RF for L with SD
l
150
100
50
0
0
10
25
50
100
Cytarabine
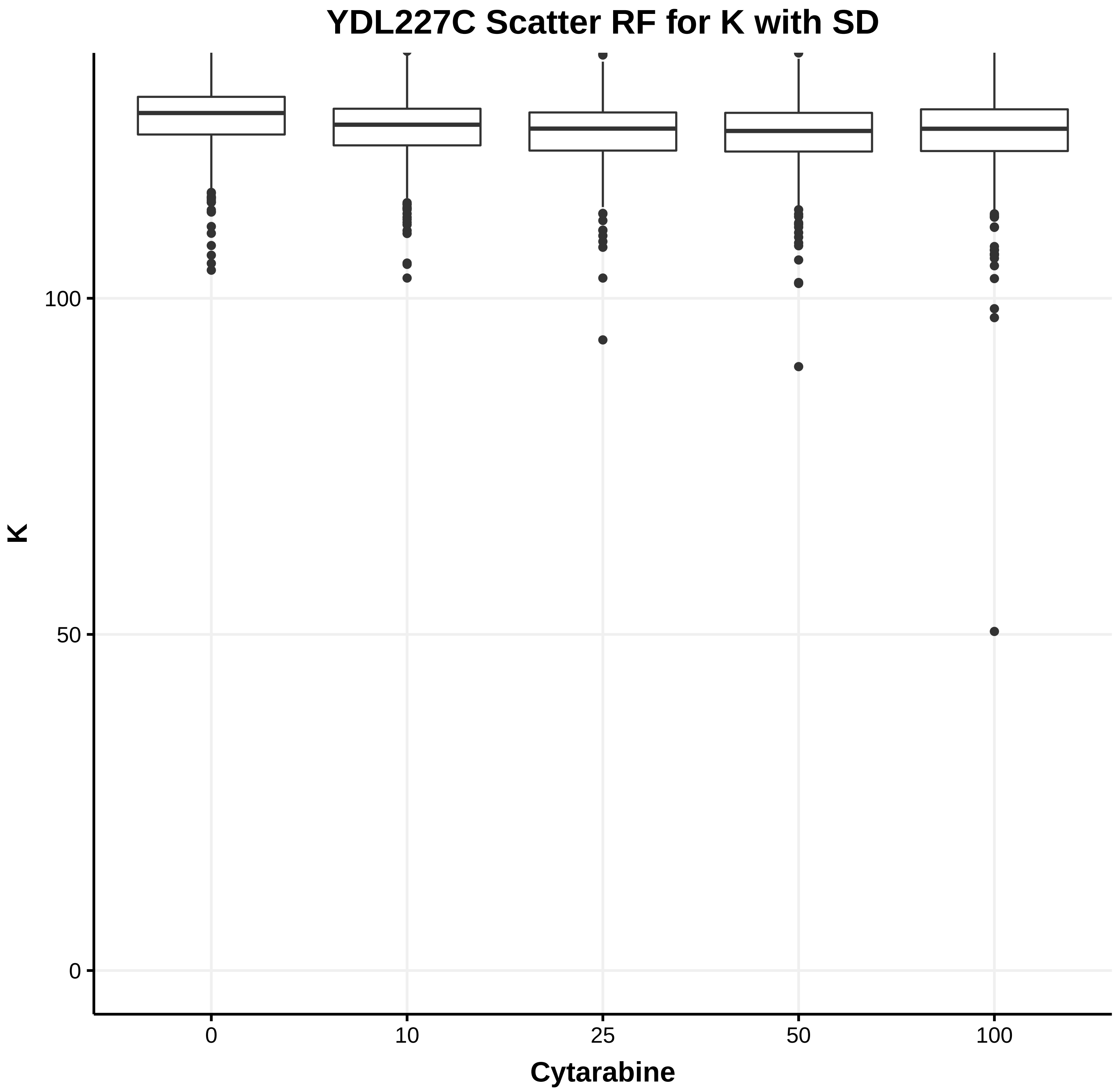
YDL227C Scatter RF for K with SD
K
100
50
0
0
10
25
50
100
Cytarabine
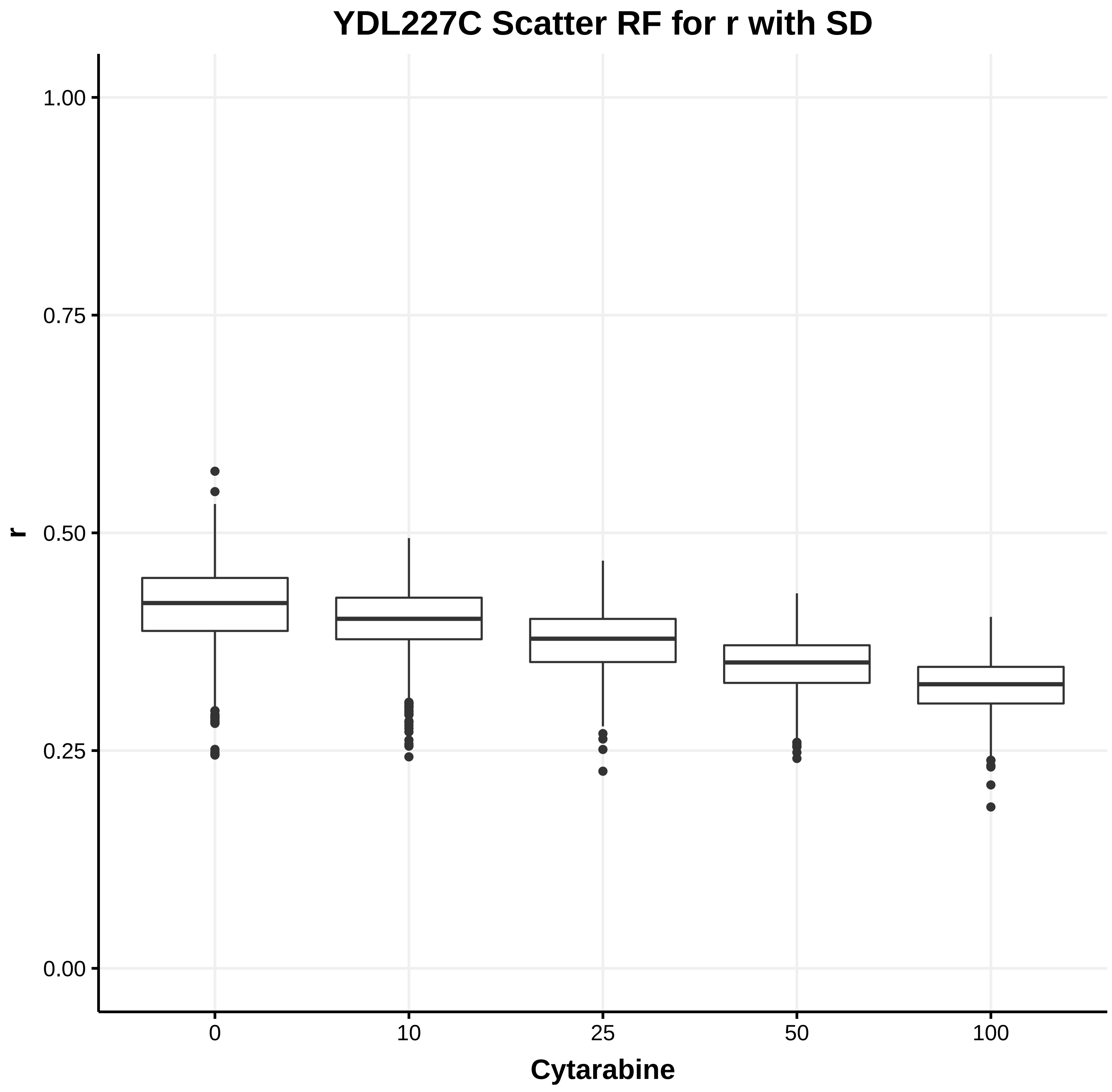
YDL227C Scatter RF for r with SD
r
1.00
0.75
0.50
0.25
0.00
0
10
25
50
100
Cytarabine
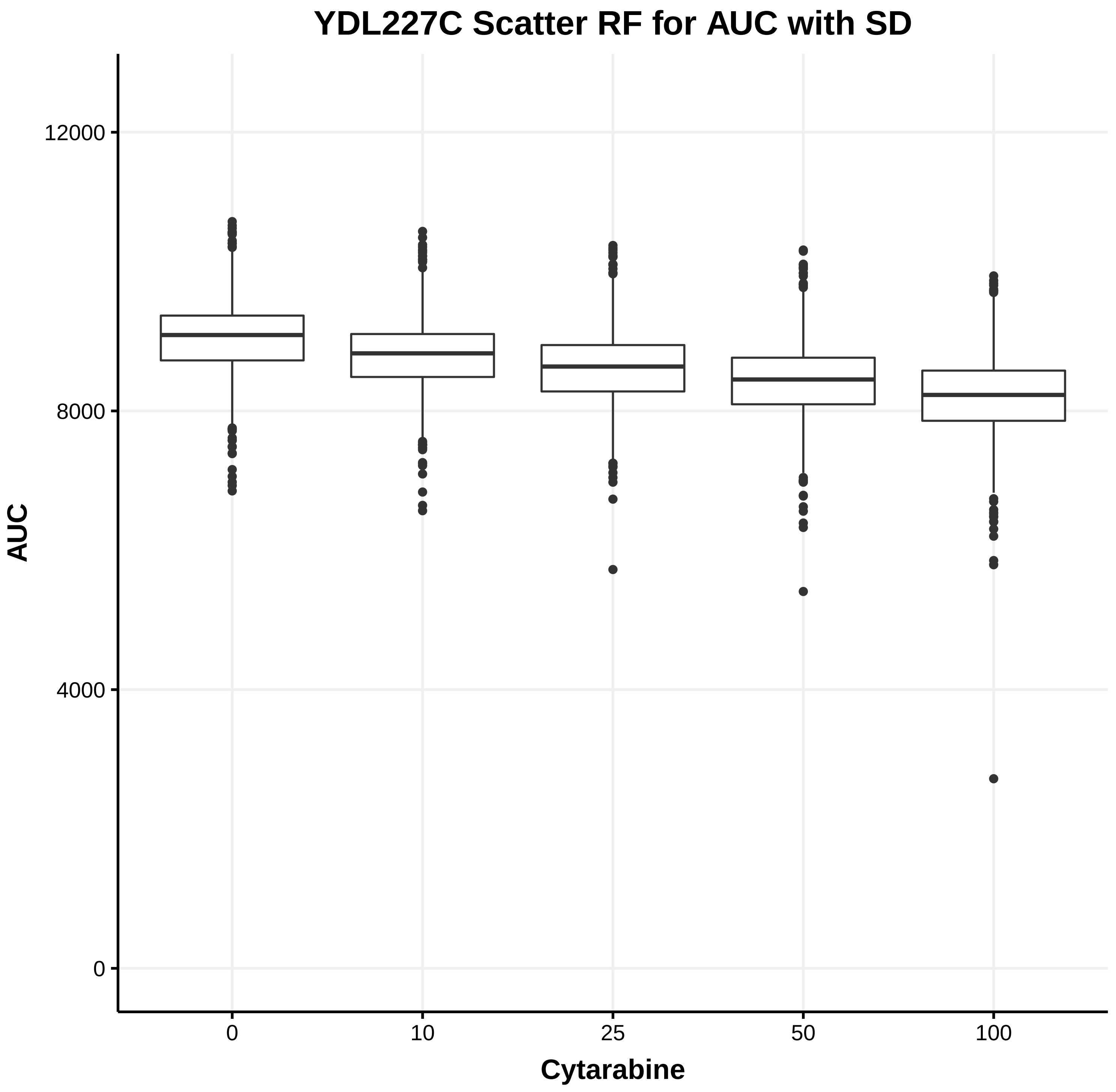
YDL227C Scatter RF for AUC with SD
AUC
12000
8000
4000
0
0
10
25
50
100
Cytarabine

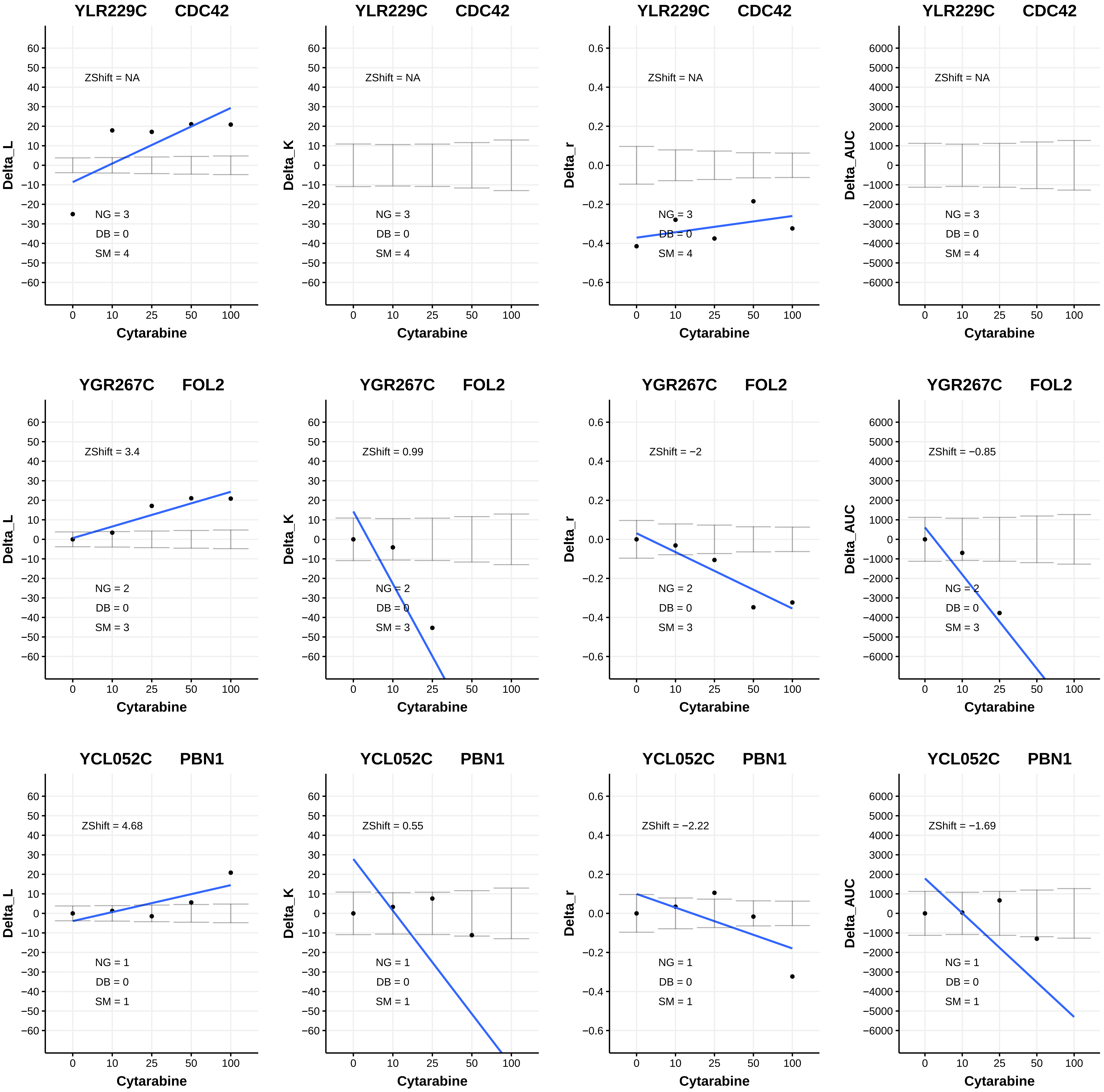

YLR229C CDC42
ZShift = NA
NG = 3
DB = 0
SM = 4
Delta_L
Delta_K
Delta_r
Delta_AUC
Cytarabine
0
10
25
50
100
YGR267C FOL2
ZShift = 3.4
ZShift = 0.99
ZShift = −2
ZShift = −0.85
NG = 2
DB = 0
SM = 3
YCL052C PBN1
ZShift = 4.68
ZShift = 0.55
ZShift = −2.22
ZShift = −1.69
NG = 1
DB = 0
SM = 1

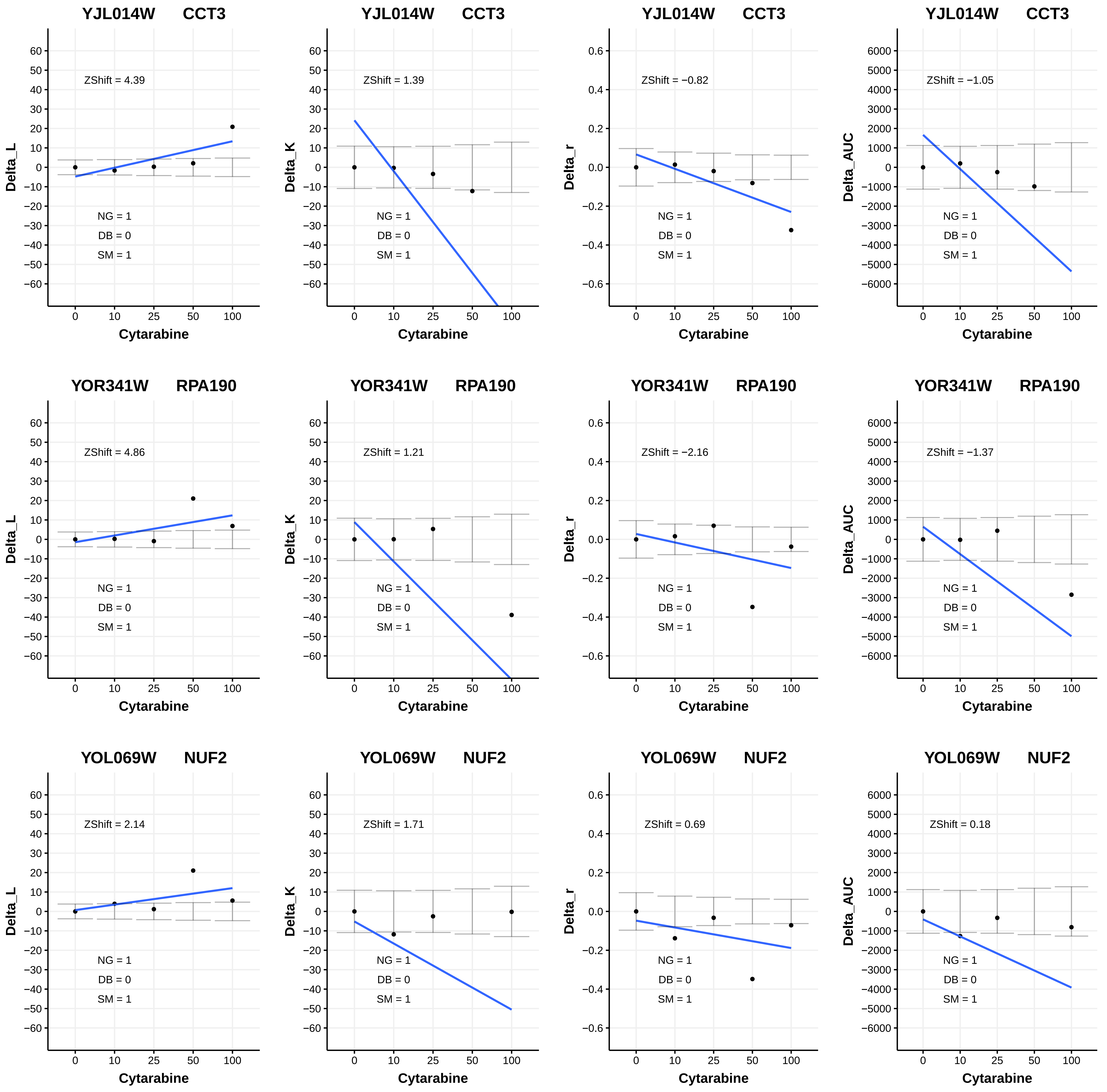
YJL014W CCT3
ZShift = 4.39
NG = 1
DB = 0
SM = 1
Delta_L
Cytarabine
YJL014W CCT3
ZShift = 1.39
NG = 1
DB = 0
SM = 1
Delta_K
Cytarabine
YJL014W CCT3
ZShift = −0.82
NG = 1
DB = 0
SM = 1
Delta_r
Cytarabine
YJL014W CCT3
ZShift = −1.05
NG = 1
DB = 0
SM = 1
Delta_AUC
Cytarabine
YOR341W RPA190
ZShift = 4.86
NG = 1
DB = 0
SM = 1
Delta_L
Cytarabine
YOR341W RPA190
ZShift = 1.21
NG = 1
DB = 0
SM = 1
Delta_K
Cytarabine
YOR341W RPA190
ZShift = −2.16
NG = 1
DB = 0
SM = 1
Delta_r
Cytarabine
YOR341W RPA190
ZShift = −1.37
NG = 1
DB = 0
SM = 1
Delta_AUC
Cytarabine
YOL069W NUF2
ZShift = 2.14
NG = 1
DB = 0
SM = 1
Delta_L
Cytarabine
YOL069W NUF2
ZShift = 1.71
NG = 1
DB = 0
SM = 1
Delta_K
Cytarabine
YOL069W NUF2
ZShift = 0.69
NG = 1
DB = 0
SM = 1
Delta_r
Cytarabine
YOL069W NUF2
ZShift = 0.18
NG = 1
DB = 0
SM = 1
Delta_AUC
Cytarabine

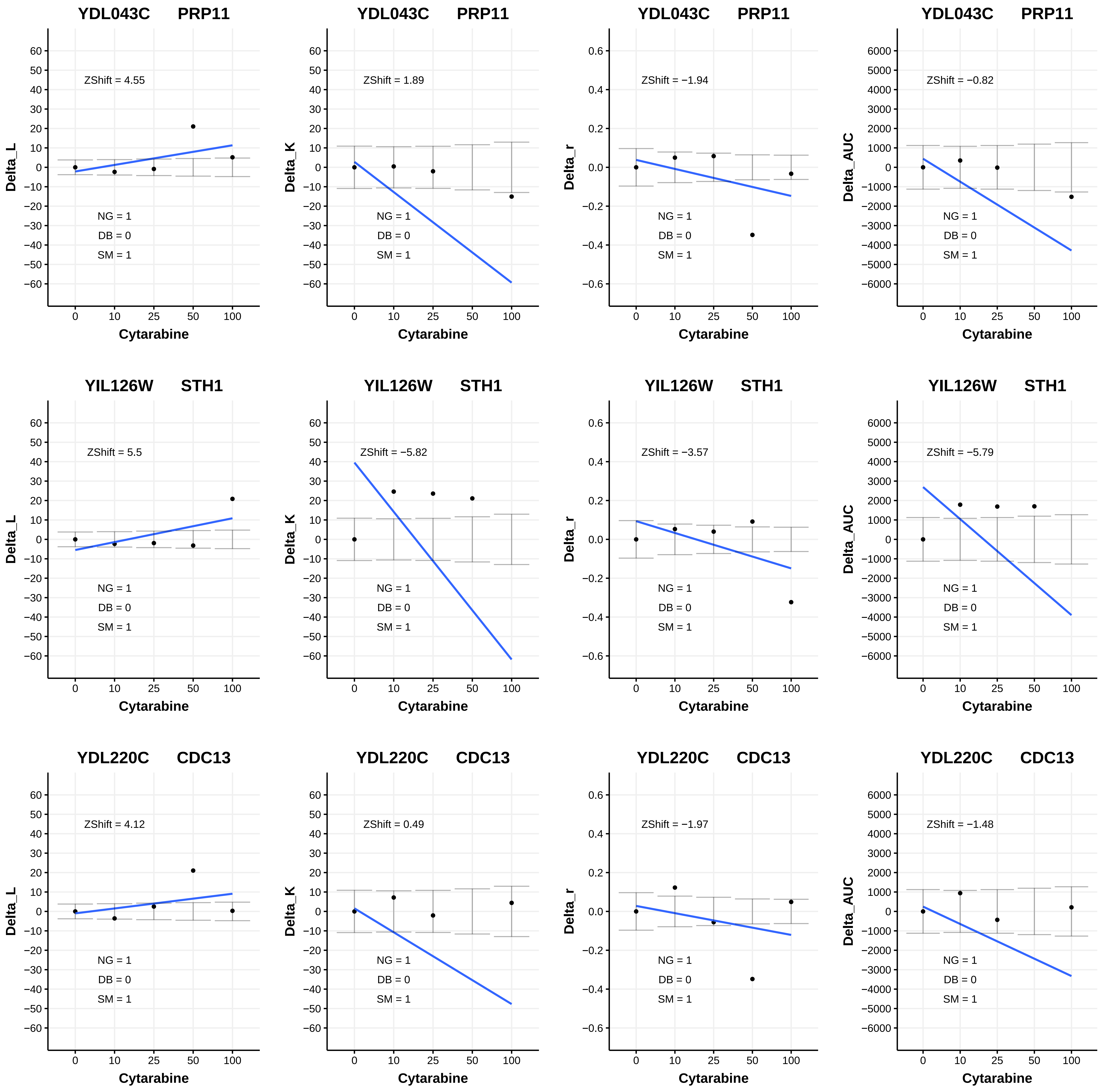
YDL043C PRP11
ZShift = 4.55
NG = 1
DB = 0
SM = 1
Delta_L
Cytarabine
YDL043C PRP11
ZShift = 1.89
NG = 1
DB = 0
SM = 1
Delta_K
Cytarabine
YDL043C PRP11
ZShift = −1.94
NG = 1
DB = 0
SM = 1
Delta_r
Cytarabine
YDL043C PRP11
ZShift = −0.82
NG = 1
DB = 0
SM = 1
Delta_AUC
Cytarabine
YIL126W STH1
ZShift = 5.5
NG = 1
DB = 0
SM = 1
Delta_L
Cytarabine
YIL126W STH1
ZShift = −5.82
NG = 1
DB = 0
SM = 1
Delta_K
Cytarabine
YIL126W STH1
ZShift = −3.57
NG = 1
DB = 0
SM = 1
Delta_r
Cytarabine
YIL126W STH1
ZShift = −5.79
NG = 1
DB = 0
SM = 1
Delta_AUC
Cytarabine
YDL220C CDC13
ZShift = 4.12
NG = 1
DB = 0
SM = 1
Delta_L
Cytarabine
YDL220C CDC13
ZShift = 0.49
NG = 1
DB = 0
SM = 1
Delta_K
Cytarabine
YDL220C CDC13
ZShift = −1.97
NG = 1
DB = 0
SM = 1
Delta_r
Cytarabine
YDL220C CDC13
ZShift = −1.48
NG = 1
DB = 0
SM = 1
Delta_AUC
Cytarabine

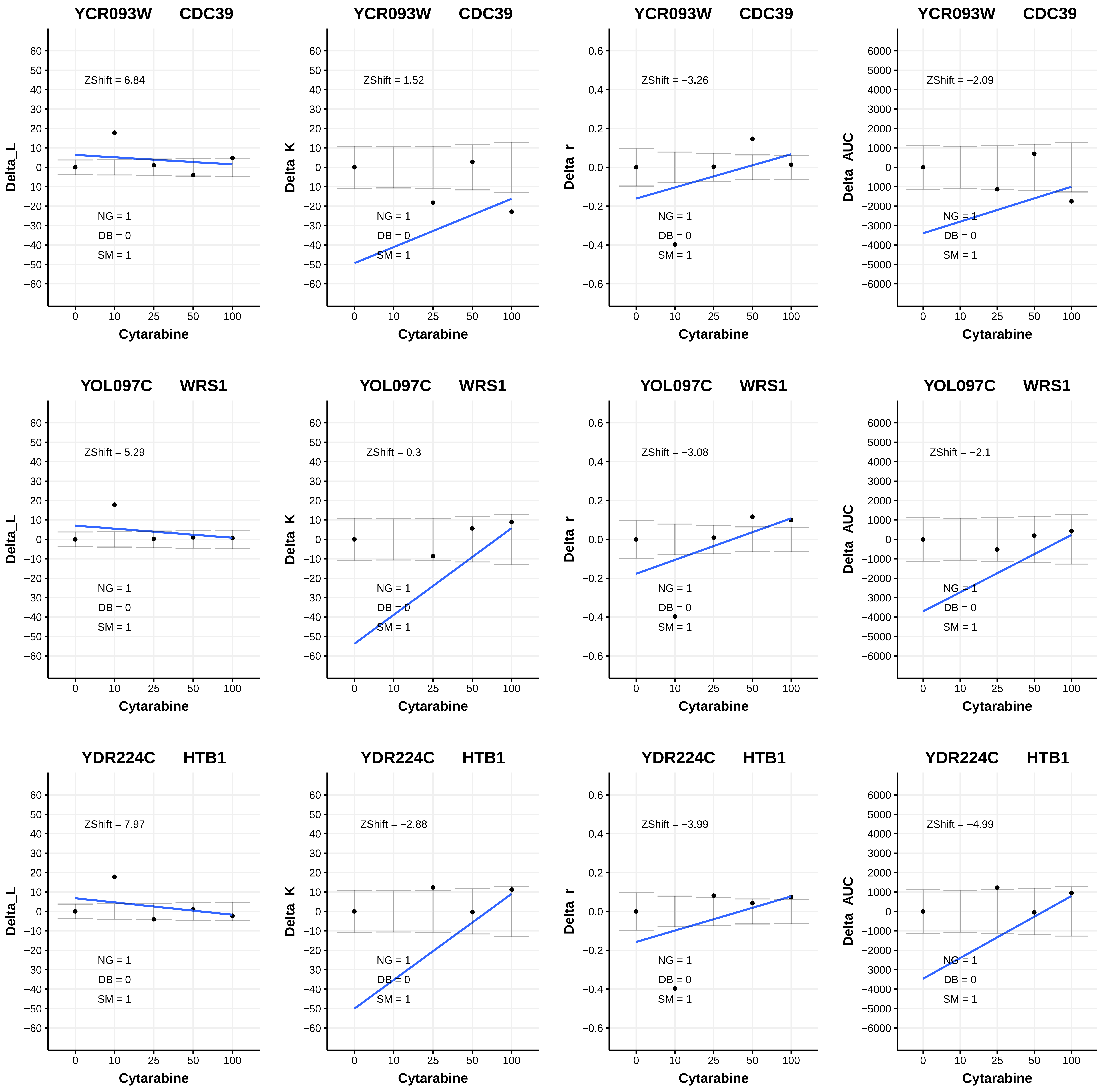

YCR093W CDC39
ZShift = 6.84
NG = 1
DB = 0
SM = 1
Delta_L
Cytarabine
YCR093W CDC39
ZShift = 1.52
NG = 1
DB = 0
SM = 1
Delta_K
Cytarabine
YCR093W CDC39
ZShift = −3.26
NG = 1
DB = 0
SM = 1
Delta_r
Cytarabine
YCR093W CDC39
ZShift = −2.09
NG = 1
DB = 0
SM = 1
Delta_AUC
Cytarabine
YOL097C WRS1
ZShift = 5.29
NG = 1
DB = 0
SM = 1
Delta_L
Cytarabine
YOL097C WRS1
ZShift = 0.3
NG = 1
DB = 0
SM = 1
Delta_K
Cytarabine
YOL097C WRS1
ZShift = −3.08
NG = 1
DB = 0
SM = 1
Delta_r
Cytarabine
YOL097C WRS1
ZShift = −2.1
NG = 1
DB = 0
SM = 1
Delta_AUC
Cytarabine
YDR224C HTB1
ZShift = 7.97
NG = 1
DB = 0
SM = 1
Delta_L
Cytarabine
YDR224C HTB1
ZShift = −2.88
NG = 1
DB = 0
SM = 1
Delta_K
Cytarabine
YDR224C HTB1
ZShift = −3.99
NG = 1
DB = 0
SM = 1
Delta_r
Cytarabine
YDR224C HTB1
ZShift = −4.99
NG = 1
DB = 0
SM = 1
Delta_AUC
Cytarabine

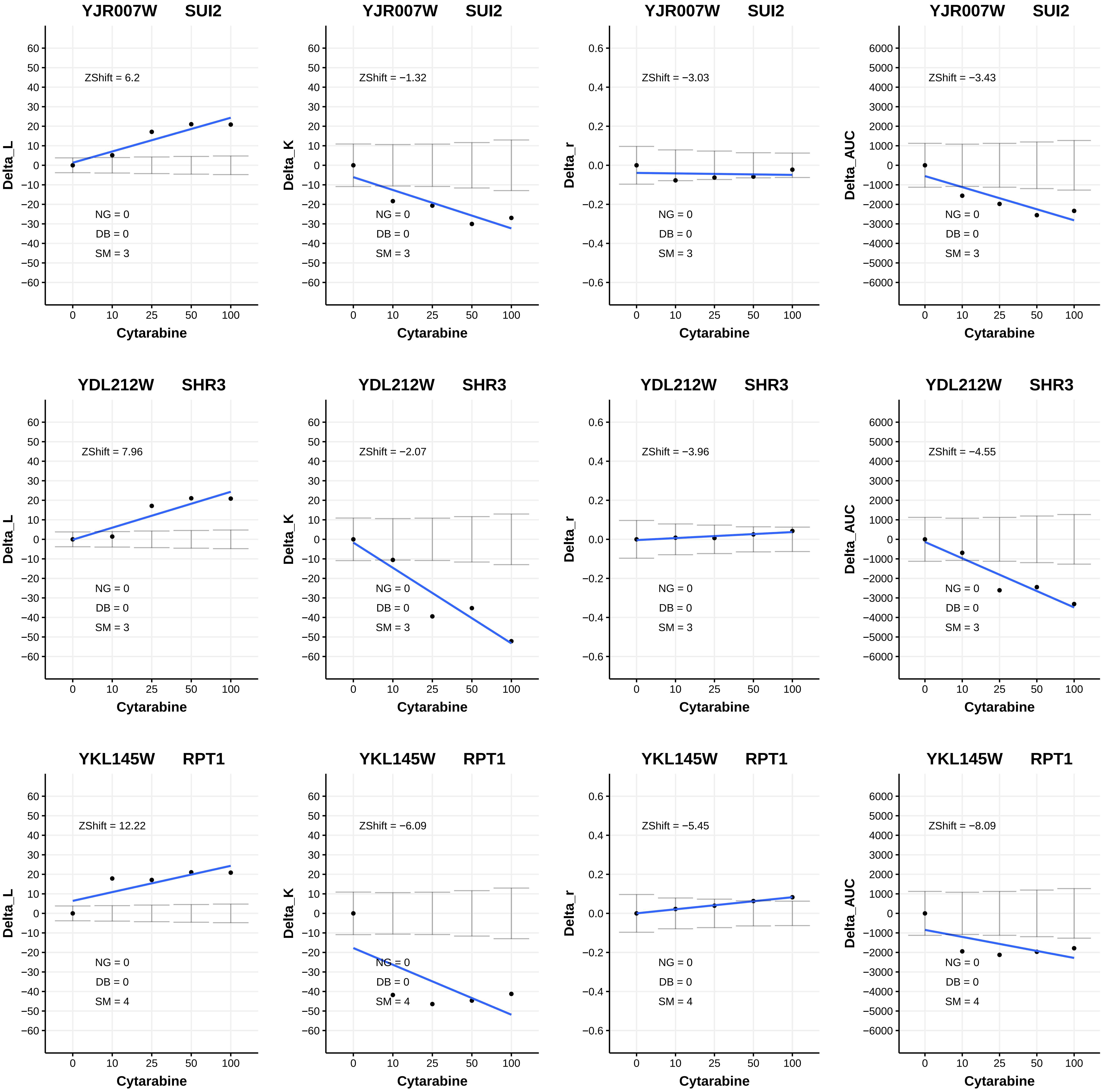
YJR007W SUI2
ZShift = 6.2
NG = 0
DB = 0
SM = 3
Delta_L
Cytarabine
YJR007W SUI2
ZShift = −1.32
NG = 0
DB = 0
SM = 3
Delta_K
Cytarabine
YJR007W SUI2
ZShift = −3.03
NG = 0
DB = 0
SM = 3
Delta_r
Cytarabine
YJR007W SUI2
ZShift = −3.43
NG = 0
DB = 0
SM = 3
Delta_AUC
Cytarabine
YDL212W SHR3
ZShift = 7.96
NG = 0
DB = 0
SM = 3
Delta_L
Cytarabine
YDL212W SHR3
ZShift = −2.07
NG = 0
DB = 0
SM = 3
Delta_K
Cytarabine
YDL212W SHR3
ZShift = −3.96
NG = 0
DB = 0
SM = 3
Delta_r
Cytarabine
YDL212W SHR3
ZShift = −4.55
NG = 0
DB = 0
SM = 3
Delta_AUC
Cytarabine
YKL145W RPT1
ZShift = 12.22
NG = 0
DB = 0
SM = 4
Delta_L
Cytarabine
YKL145W RPT1
ZShift = −6.09
NG = 0
DB = 0
SM = 4
Delta_K
Cytarabine
YKL145W RPT1
ZShift = −5.45
NG = 0
DB = 0
SM = 4
Delta_r
Cytarabine
YKL145W RPT1
ZShift = −8.09
NG = 0
DB = 0
SM = 4
Delta_AUC
Cytarabine

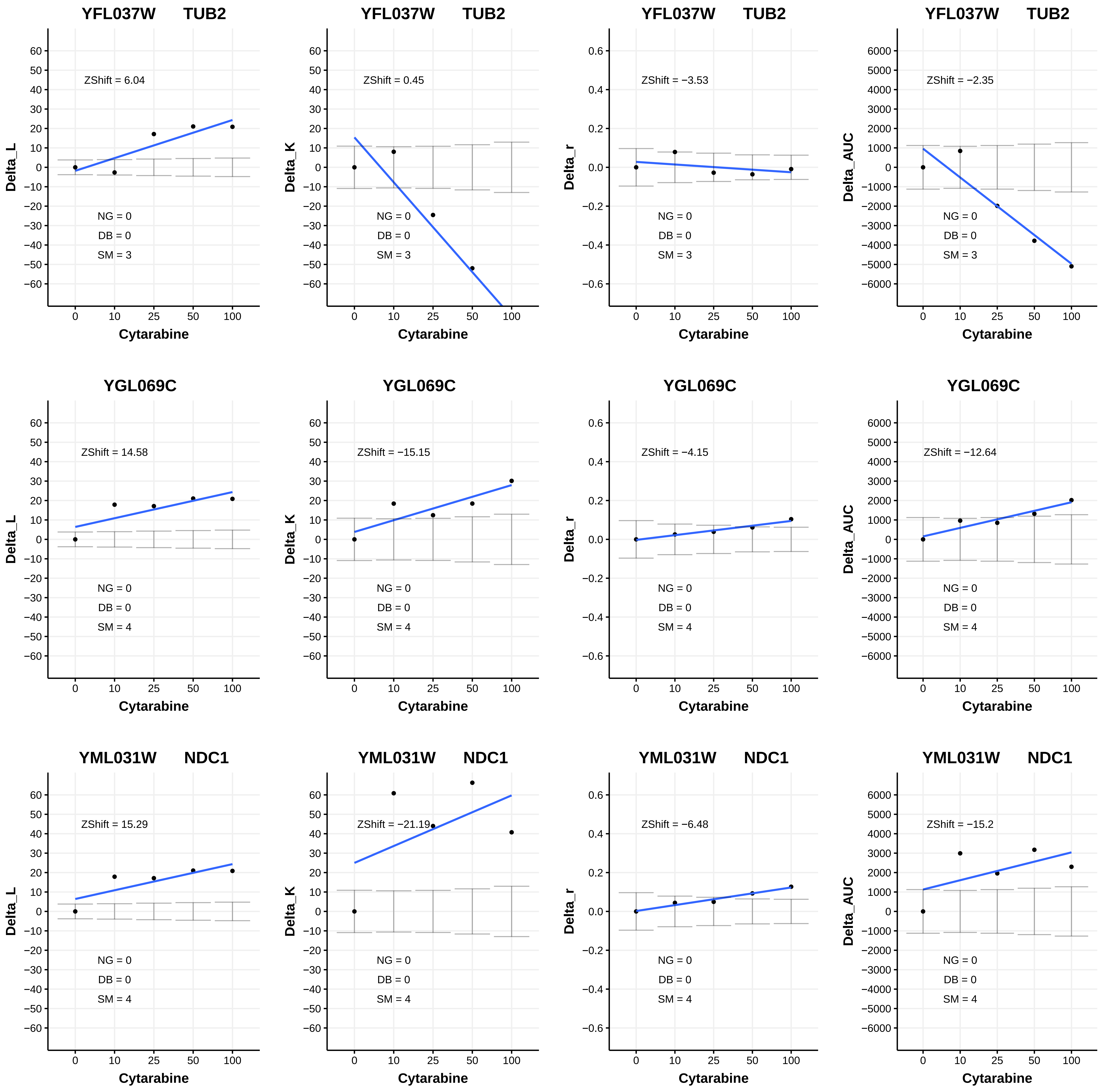
YFL037W TUB2
ZShift = 6.04
NG = 0
DB = 0
SM = 3
Delta_L
Cytarabine
YFL037W TUB2
ZShift = 0.45
NG = 0
DB = 0
SM = 3
Delta_K
Cytarabine
YFL037W TUB2
ZShift = −3.53
NG = 0
DB = 0
SM = 3
Delta_r
Cytarabine
YFL037W TUB2
ZShift = −2.35
NG = 0
DB = 0
SM = 3
Delta_AUC
Cytarabine
YGL069C
ZShift = 14.58
NG = 0
DB = 0
SM = 4
Delta_L
Cytarabine
YGL069C
ZShift = −15.15
NG = 0
DB = 0
SM = 4
Delta_K
Cytarabine
YGL069C
ZShift = −4.15
NG = 0
DB = 0
SM = 4
Delta_r
Cytarabine
YGL069C
ZShift = −12.64
NG = 0
DB = 0
SM = 4
Delta_AUC
Cytarabine
YML031W NDC1
ZShift = 15.29
NG = 0
DB = 0
SM = 4
Delta_L
Cytarabine
YML031W NDC1
ZShift = −21.19
NG = 0
DB = 0
SM = 4
Delta_K
Cytarabine
YML031W NDC1
ZShift = −6.48
NG = 0
DB = 0
SM = 4
Delta_r
Cytarabine
YML031W NDC1
ZShift = −15.2
NG = 0
DB = 0
SM = 4
Delta_AUC
Cytarabine

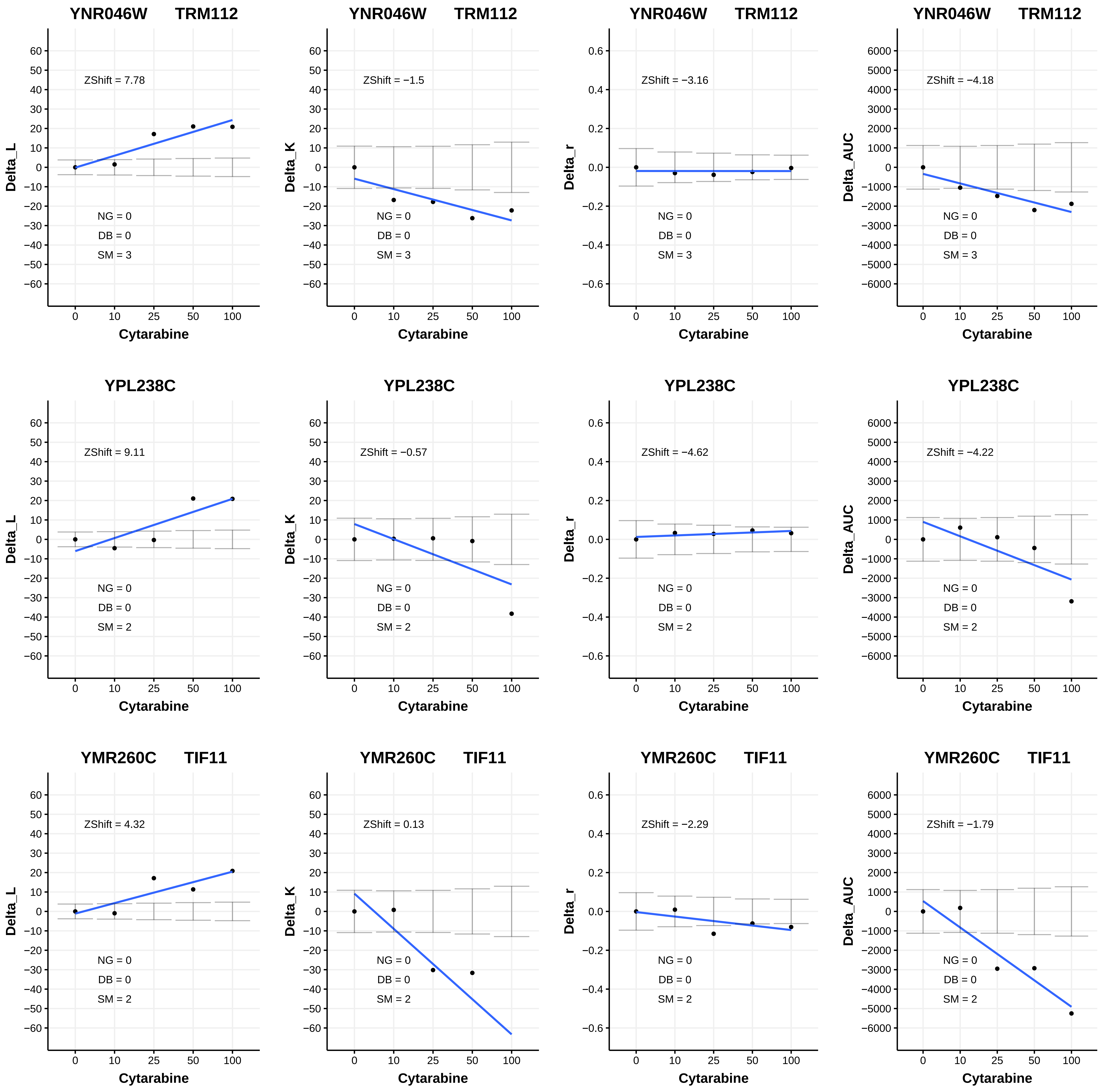
YNR046W TRM112
ZShift = 7.78
NG = 0
DB = 0
SM = 3
Delta_L
Cytarabine
YNR046W TRM112
ZShift = −1.5
NG = 0
DB = 0
SM = 3
Delta_K
Cytarabine
YNR046W TRM112
ZShift = −3.16
NG = 0
DB = 0
SM = 3
Delta_r
Cytarabine
YNR046W TRM112
ZShift = −4.18
NG = 0
DB = 0
SM = 3
Delta_AUC
Cytarabine
YPL238C
ZShift = 9.11
NG = 0
DB = 0
SM = 2
Delta_L
Cytarabine
YPL238C
ZShift = −0.57
NG = 0
DB = 0
SM = 2
Delta_K
Cytarabine
YPL238C
ZShift = −4.62
NG = 0
DB = 0
SM = 2
Delta_r
Cytarabine
YPL238C
ZShift = −4.22
NG = 0
DB = 0
SM = 2
Delta_AUC
Cytarabine
YMR260C TIF11
ZShift = 4.32
NG = 0
DB = 0
SM = 2
Delta_L
Cytarabine
YMR260C TIF11
ZShift = 0.13
NG = 0
DB = 0
SM = 2
Delta_K
Cytarabine
YMR260C TIF11
ZShift = −2.29
NG = 0
DB = 0
SM = 2
Delta_r
Cytarabine
YMR260C TIF11
ZShift = −1.79
NG = 0
DB = 0
SM = 2
Delta_AUC
Cytarabine

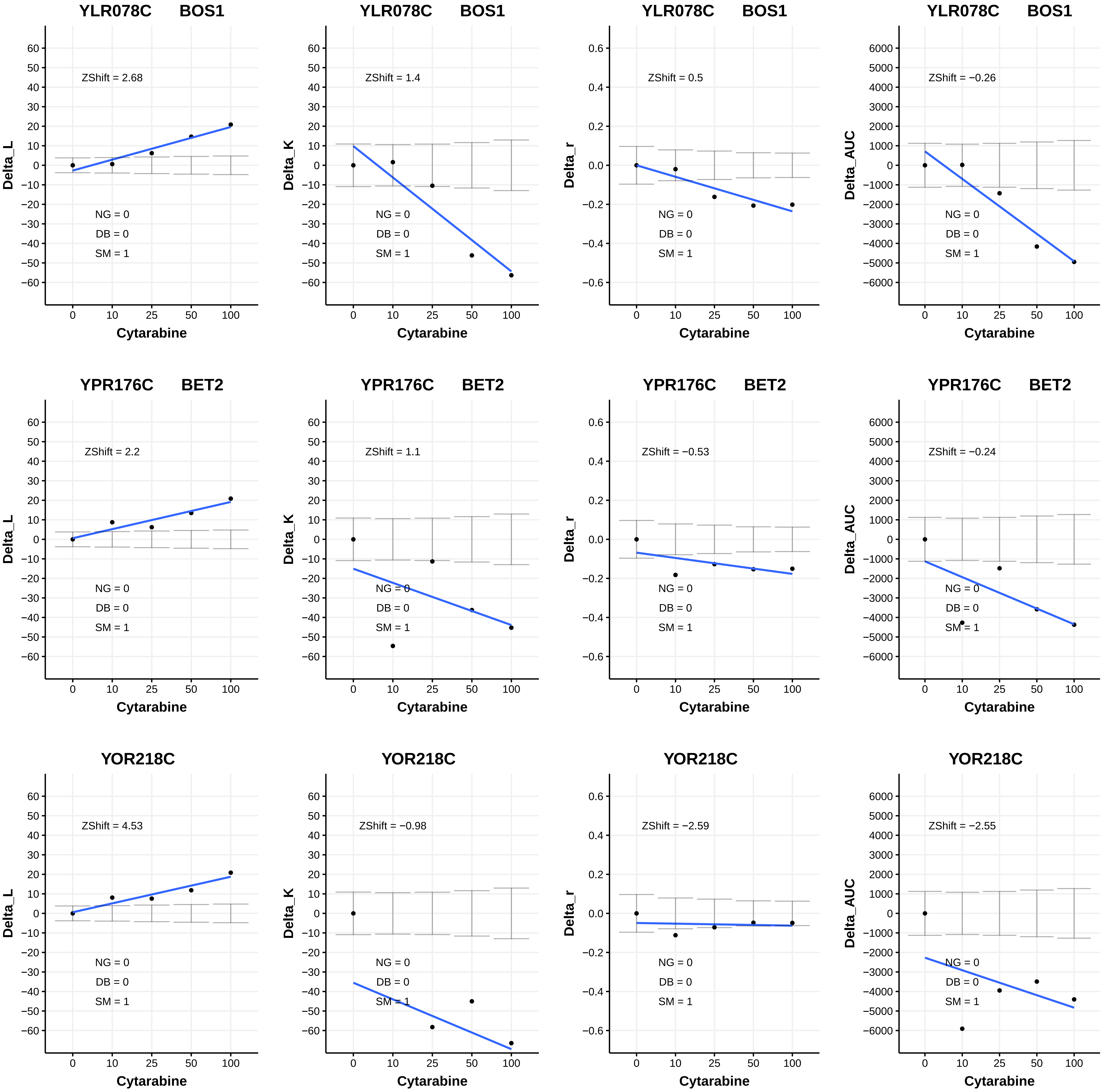
YLR078C BOS1
ZShift = 2.68
NG = 0
DB = 0
SM = 1
Delta_L
Cytarabine
YLR078C BOS1
ZShift = 1.4
NG = 0
DB = 0
SM = 1
Delta_K
Cytarabine
YLR078C BOS1
ZShift = 0.5
NG = 0
DB = 0
SM = 1
Delta_r
Cytarabine
YLR078C BOS1
ZShift = −0.26
NG = 0
DB = 0
SM = 1
Delta_AUC
Cytarabine
YPR176C BET2
ZShift = 2.2
NG = 0
DB = 0
SM = 1
Delta_L
Cytarabine
YPR176C BET2
ZShift = 1.1
NG = 0
DB = 0
SM = 1
Delta_K
Cytarabine
YPR176C BET2
ZShift = −0.53
NG = 0
DB = 0
SM = 1
Delta_r
Cytarabine
YPR176C BET2
ZShift = −0.24
NG = 0
DB = 0
SM = 1
Delta_AUC
Cytarabine
YOR218C
ZShift = 4.53
NG = 0
DB = 0
SM = 1
Delta_L
Cytarabine
YOR218C
ZShift = −0.98
NG = 0
DB = 0
SM = 1
Delta_K
Cytarabine
YOR218C
ZShift = −2.59
NG = 0
DB = 0
SM = 1
Delta_r
Cytarabine
YOR218C
ZShift = −2.55
NG = 0
DB = 0
SM = 1
Delta_AUC
Cytarabine

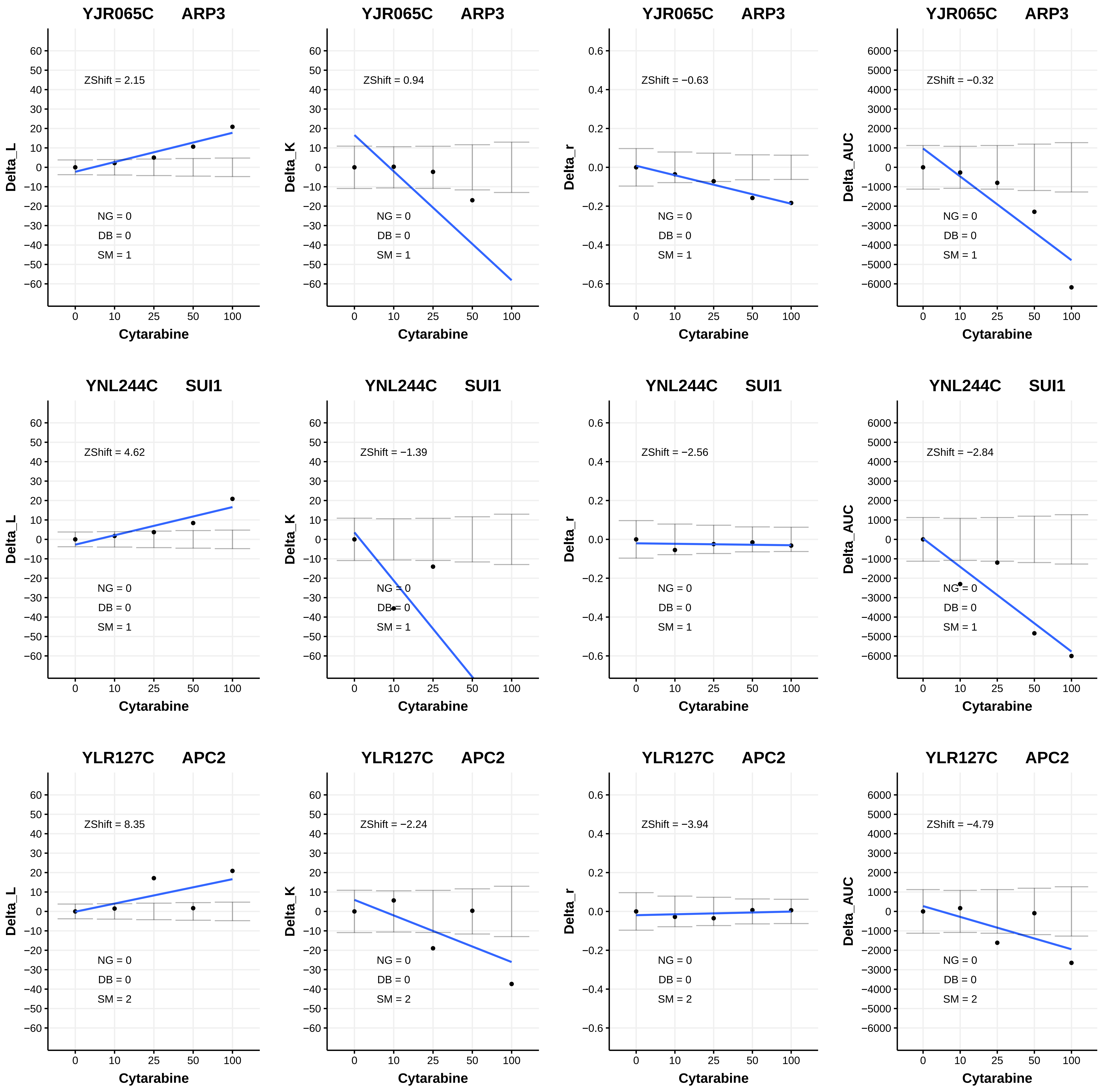
YJR065C ARP3
ZShift = 2.15
NG = 0
DB = 0
SM = 1
Delta_L
Cytarabine
YJR065C ARP3
ZShift = 0.94
NG = 0
DB = 0
SM = 1
Delta_K
Cytarabine
YJR065C ARP3
ZShift = −0.63
NG = 0
DB = 0
SM = 1
Delta_r
Cytarabine
YJR065C ARP3
ZShift = −0.32
NG = 0
DB = 0
SM = 1
Delta_AUC
Cytarabine
YNL244C SUI1
ZShift = 4.62
NG = 0
DB = 0
SM = 1
Delta_L
Cytarabine
YNL244C SUI1
ZShift = −1.39
NG = 0
DB = 0
SM = 1
Delta_K
Cytarabine
YNL244C SUI1
ZShift = −2.56
NG = 0
DB = 0
SM = 1
Delta_r
Cytarabine
YNL244C SUI1
ZShift = −2.84
NG = 0
DB = 0
SM = 1
Delta_AUC
Cytarabine
YLR127C APC2
ZShift = 8.35
NG = 0
DB = 0
SM = 2
Delta_L
Cytarabine
YLR127C APC2
ZShift = −2.24
NG = 0
DB = 0
SM = 2
Delta_K
Cytarabine
YLR127C APC2
ZShift = −3.94
NG = 0
DB = 0
SM = 2
Delta_r
Cytarabine
YLR127C APC2
ZShift = −4.79
NG = 0
DB = 0
SM = 2
Delta_AUC
Cytarabine

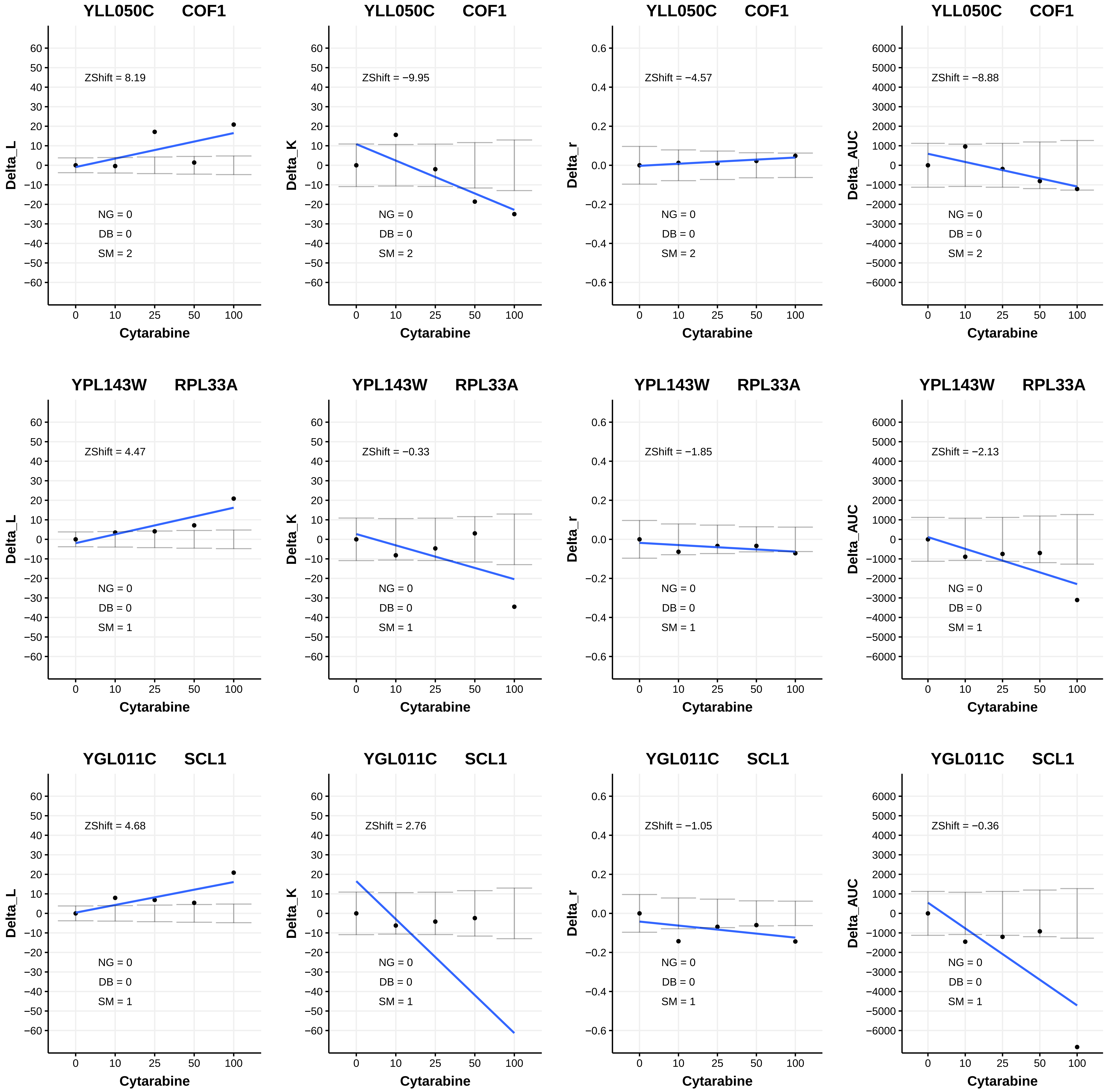
YLL050C COF1
ZShift = 8.19
NG = 0
DB = 0
SM = 2
Delta_L
Cytarabine
YLL050C COF1
ZShift = −9.95
NG = 0
DB = 0
SM = 2
Delta_K
Cytarabine
YLL050C COF1
ZShift = −4.57
NG = 0
DB = 0
SM = 2
Delta_r
Cytarabine
YLL050C COF1
ZShift = −8.88
NG = 0
DB = 0
SM = 2
Delta_AUC
Cytarabine
YPL143W RPL33A
ZShift = 4.47
NG = 0
DB = 0
SM = 1
Delta_L
Cytarabine
YPL143W RPL33A
ZShift = −0.33
NG = 0
DB = 0
SM = 1
Delta_K
Cytarabine
YPL143W RPL33A
ZShift = −1.85
NG = 0
DB = 0
SM = 1
Delta_r
Cytarabine
YPL143W RPL33A
ZShift = −2.13
NG = 0
DB = 0
SM = 1
Delta_AUC
Cytarabine
YGL011C SCL1
ZShift = 4.68
NG = 0
DB = 0
SM = 1
Delta_L
Cytarabine
YGL011C SCL1
ZShift = 2.76
NG = 0
DB = 0
SM = 1
Delta_K
Cytarabine
YGL011C SCL1
ZShift = −1.05
NG = 0
DB = 0
SM = 1
Delta_r
Cytarabine
YGL011C SCL1
ZShift = −0.36
NG = 0
DB = 0
SM = 1
Delta_AUC
Cytarabine

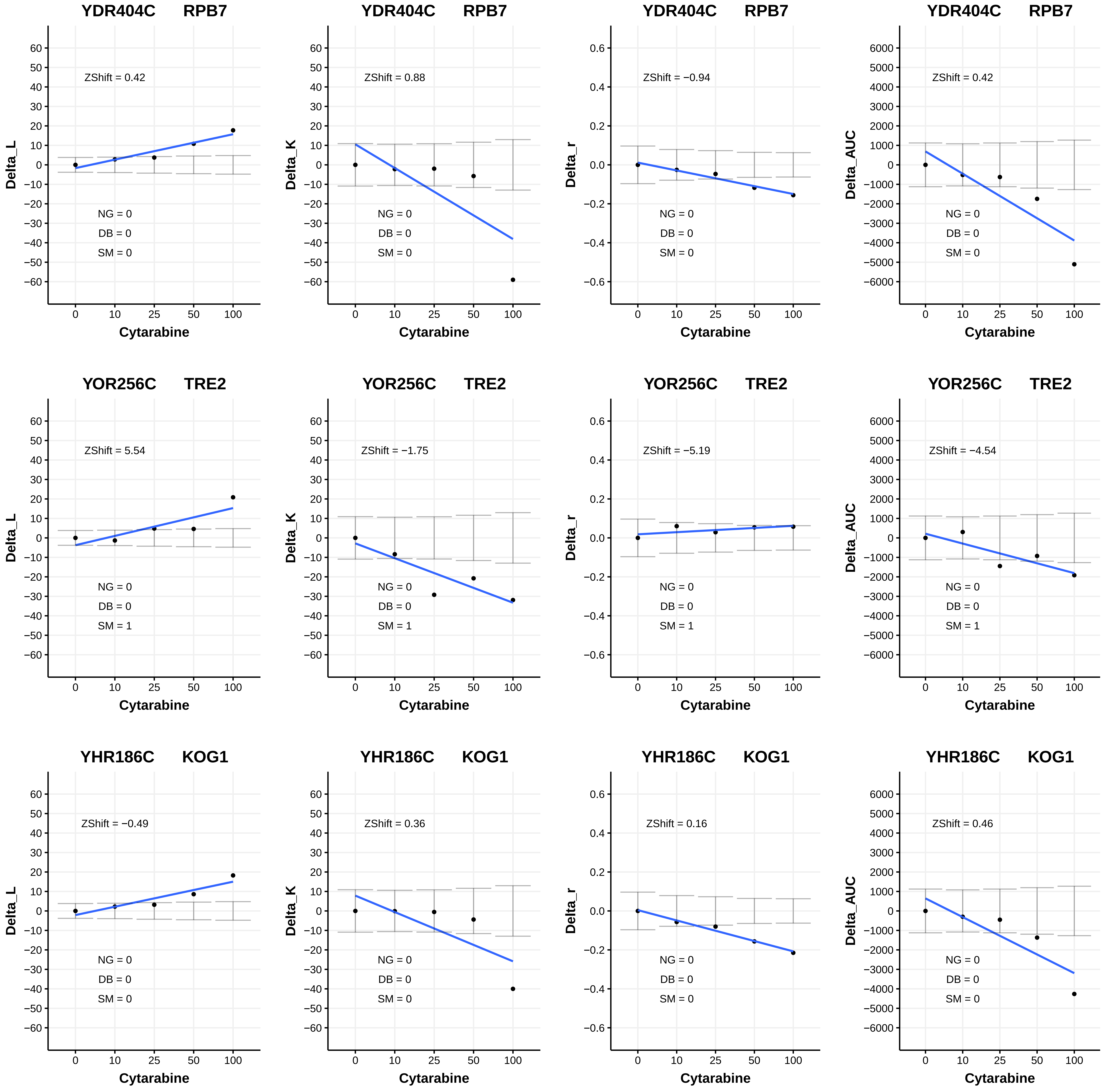

YDR404C RPB7
ZShift = 0.42
NG = 0
DB = 0
SM = 0
Delta_L
Cytarabine
YDR404C RPB7
ZShift = 0.88
NG = 0
DB = 0
SM = 0
Delta_K
Cytarabine
YDR404C RPB7
ZShift = −0.94
NG = 0
DB = 0
SM = 0
Delta_r
Cytarabine
YDR404C RPB7
ZShift = 0.42
NG = 0
DB = 0
SM = 0
Delta_AUC
Cytarabine
YOR256C TRE2
ZShift = 5.54
NG = 0
DB = 0
SM = 1
Delta_L
Cytarabine
YOR256C TRE2
ZShift = −1.75
NG = 0
DB = 0
SM = 1
Delta_K
Cytarabine
YOR256C TRE2
ZShift = −5.19
NG = 0
DB = 0
SM = 1
Delta_r
Cytarabine
YOR256C TRE2
ZShift = −4.54
NG = 0
DB = 0
SM = 1
Delta_AUC
Cytarabine
YHR186C KOG1
ZShift = −0.49
NG = 0
DB = 0
SM = 0
Delta_L
Cytarabine
YHR186C KOG1
ZShift = 0.36
NG = 0
DB = 0
SM = 0
Delta_K
Cytarabine
YHR186C KOG1
ZShift = 0.16
NG = 0
DB = 0
SM = 0
Delta_r
Cytarabine
YHR186C KOG1
ZShift = 0.46
NG = 0
DB = 0
SM = 0
Delta_AUC
Cytarabine

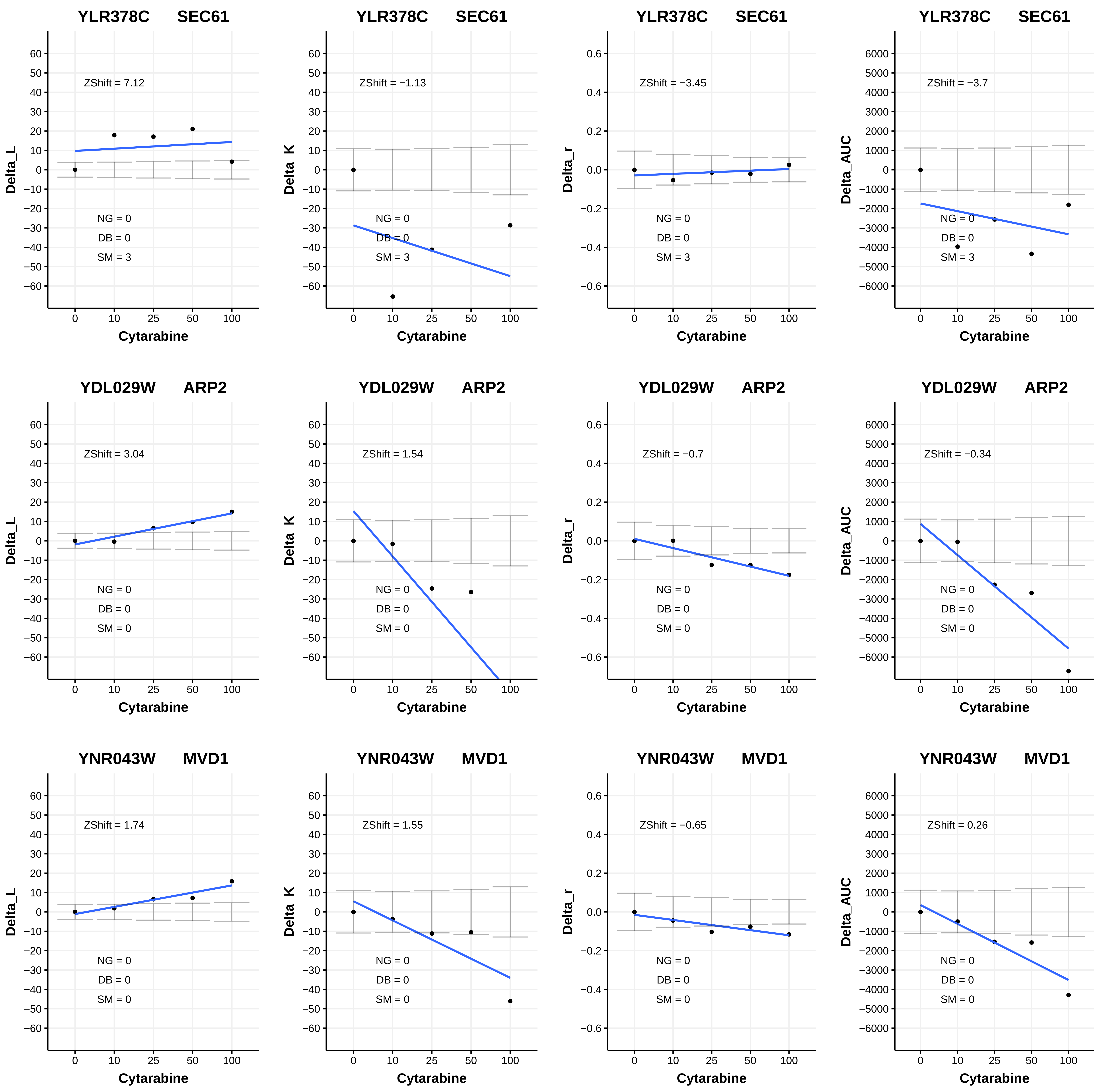
YLR378C SEC61
ZShift = 7.12
NG = 0
DB = 0
SM = 3
Delta_L
Cytarabine
YLR378C SEC61
ZShift = −1.13
NG = 0
DB = 0
SM = 3
Delta_K
Cytarabine
YLR378C SEC61
ZShift = −3.45
NG = 0
DB = 0
SM = 3
Delta_r
Cytarabine
YLR378C SEC61
ZShift = −3.7
NG = 0
DB = 0
SM = 3
Delta_AUC
Cytarabine
YDL029W ARP2
ZShift = 3.04
NG = 0
DB = 0
SM = 0
Delta_L
Cytarabine
YDL029W ARP2
ZShift = 1.54
NG = 0
DB = 0
SM = 0
Delta_K
Cytarabine
YDL029W ARP2
ZShift = −0.7
NG = 0
DB = 0
SM = 0
Delta_r
Cytarabine
YDL029W ARP2
ZShift = −0.34
NG = 0
DB = 0
SM = 0
Delta_AUC
Cytarabine
YNR043W MVD1
ZShift = 1.74
NG = 0
DB = 0
SM = 0
Delta_L
Cytarabine
YNR043W MVD1
ZShift = 1.55
NG = 0
DB = 0
SM = 0
Delta_K
Cytarabine
YNR043W MVD1
ZShift = −0.65
NG = 0
DB = 0
SM = 0
Delta_r
Cytarabine
YNR043W MVD1
ZShift = 0.26
NG = 0
DB = 0
SM = 0
Delta_AUC
Cytarabine

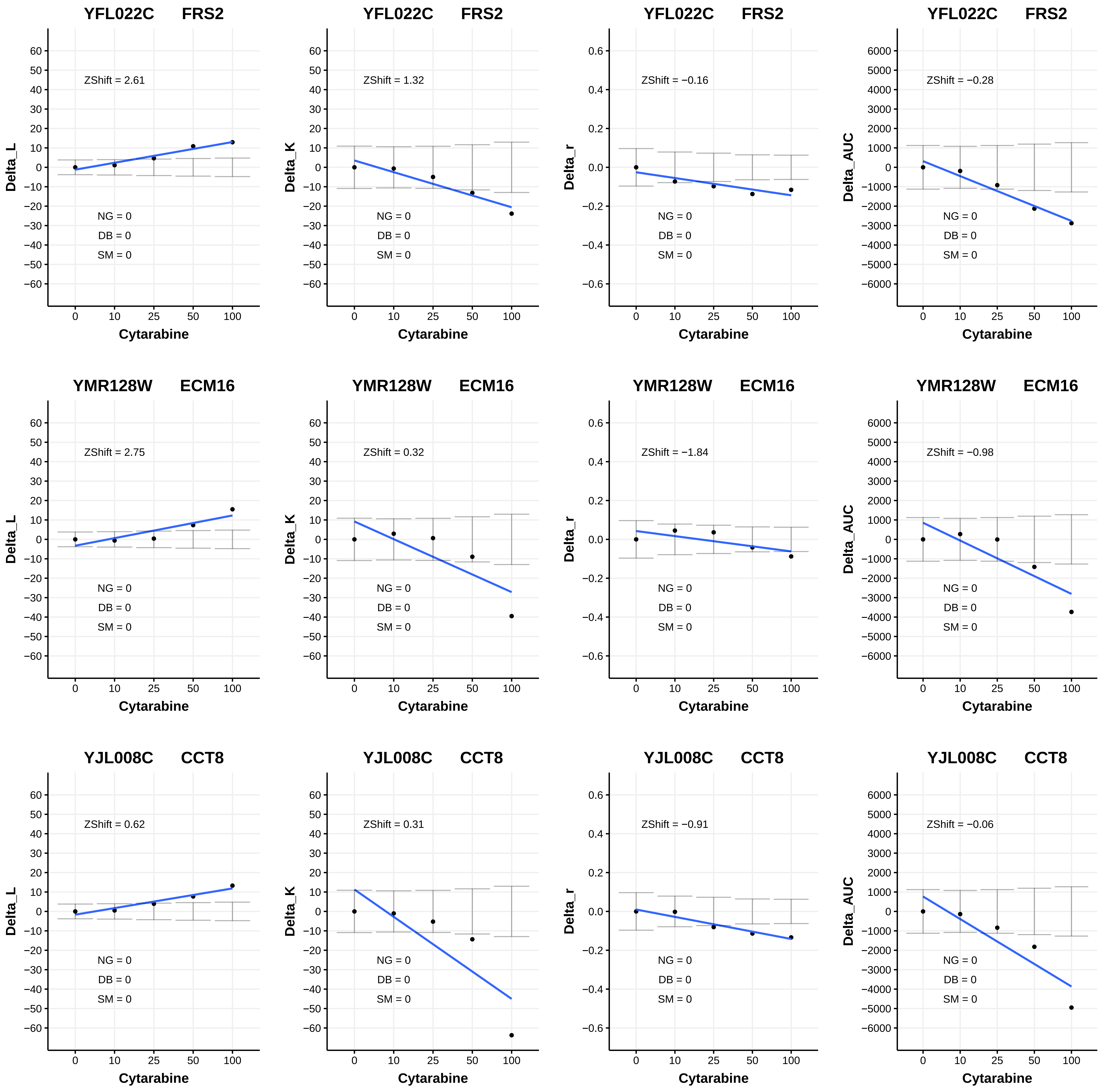
YFL022C FRS2
ZShift = 2.61
ZShift = 1.32
ZShift = −0.16
ZShift = −0.28
YMR128W ECM16
ZShift = 2.75
ZShift = 0.32
ZShift = −1.84
ZShift = −0.98
YJL008C CCT8
ZShift = 0.62
ZShift = 0.31
ZShift = −0.91
ZShift = −0.06
NG = 0
DB = 0
SM = 0
Delta_L
Delta_K
Delta_r
Delta_AUC
Cytarabine
0
10
25
50
100

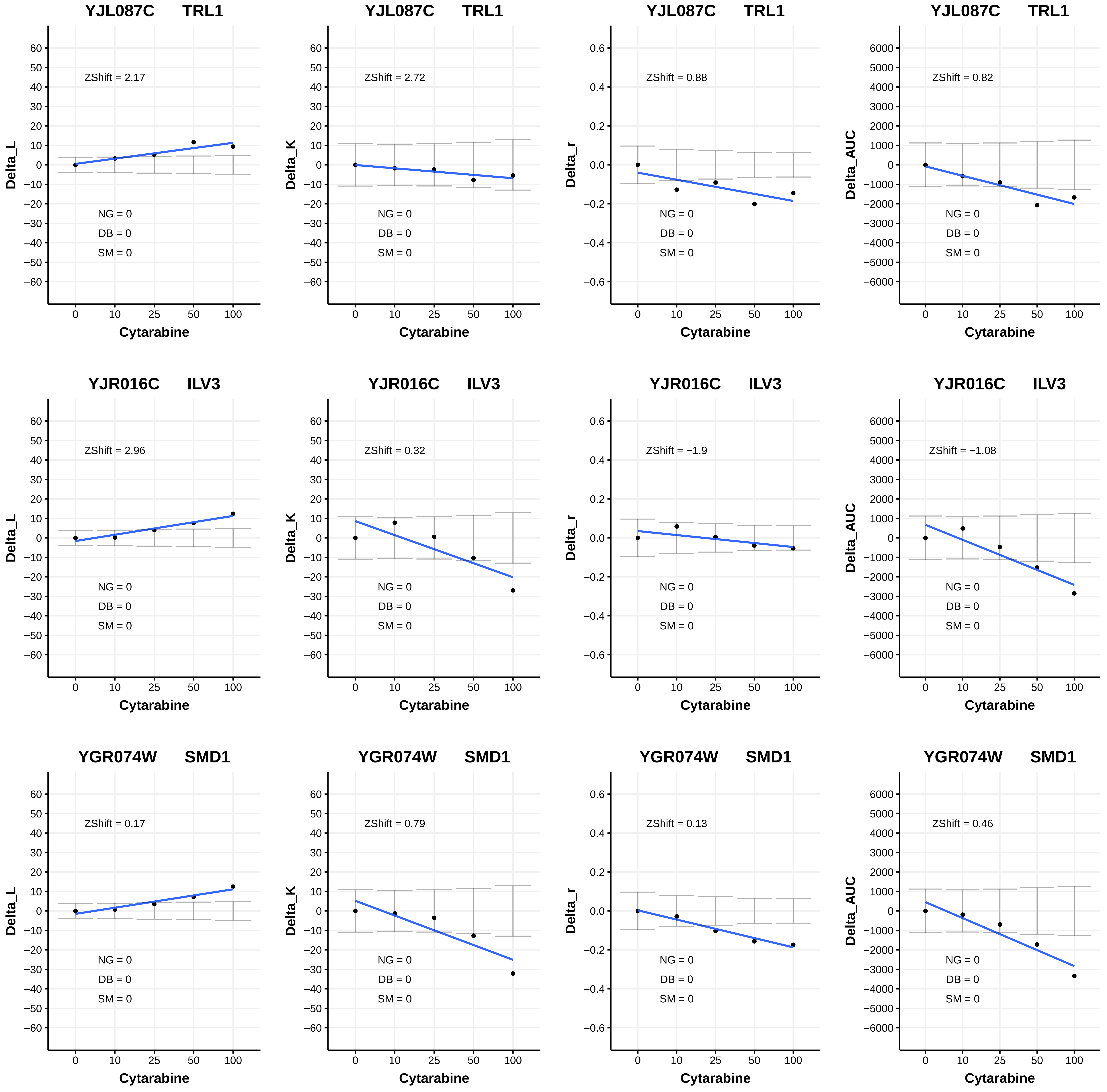
YJL087C TRL1
ZShift = 2.17
ZShift = 2.72
ZShift = 0.88
ZShift = 0.82
YJR016C ILV3
ZShift = 2.96
ZShift = 0.32
ZShift = −1.9
ZShift = −1.08
YGR074W SMD1
ZShift = 0.17
ZShift = 0.79
ZShift = 0.13
ZShift = 0.46
NG = 0
DB = 0
SM = 0
Delta_L
Delta_K
Delta_r
Delta_AUC
Cytarabine
0
10
25
50
100

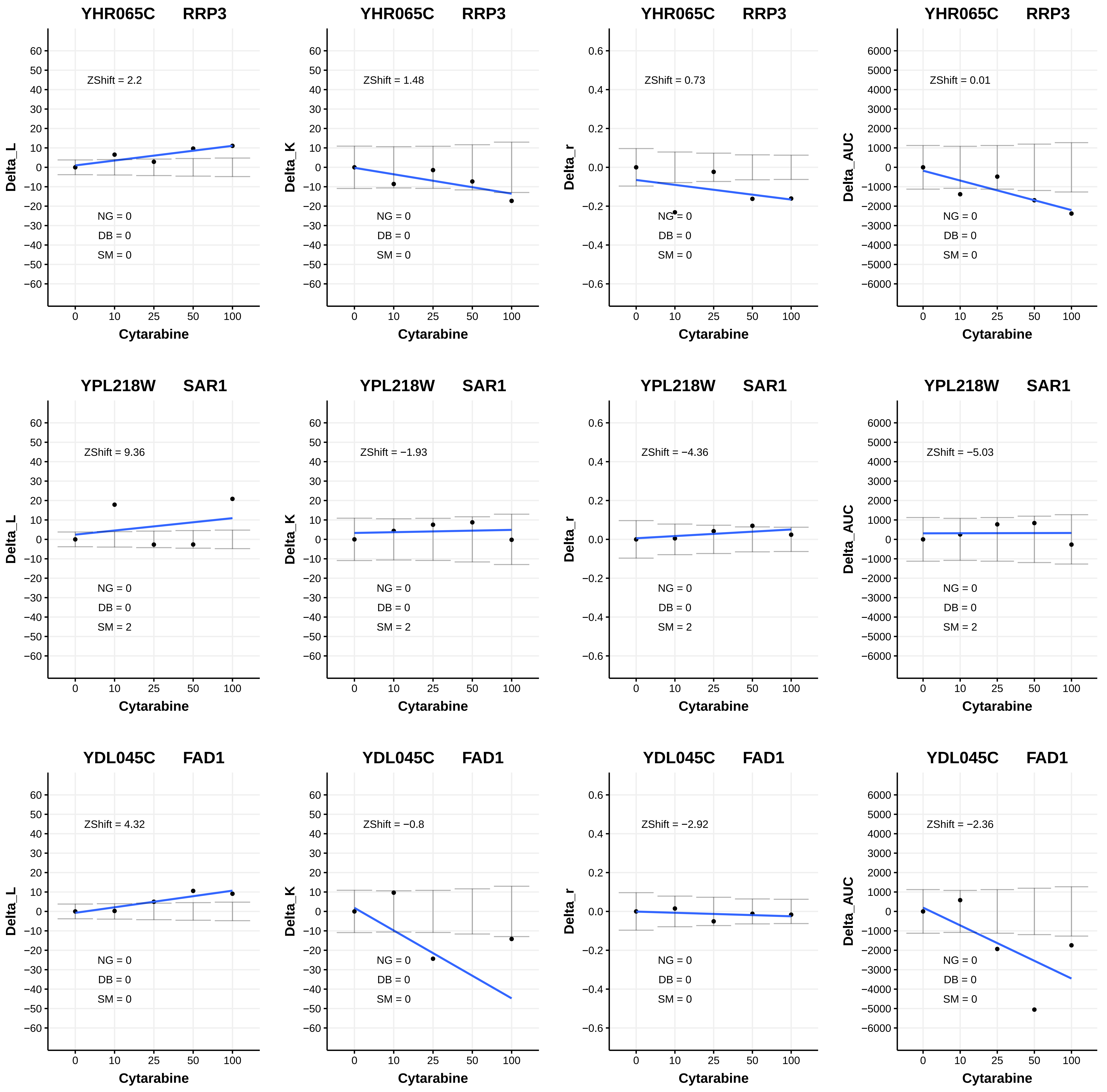

YHR065C RRP3
ZShift = 2.2
NG = 0
DB = 0
SM = 0
Delta_L
Cytarabine
YHR065C RRP3
ZShift = 1.48
NG = 0
DB = 0
SM = 0
Delta_K
Cytarabine
YHR065C RRP3
ZShift = 0.73
NG = 0
DB = 0
SM = 0
Delta_r
Cytarabine
YHR065C RRP3
ZShift = 0.01
NG = 0
DB = 0
SM = 0
Delta_AUC
Cytarabine
YPL218W SAR1
ZShift = 9.36
NG = 0
DB = 0
SM = 2
Delta_L
Cytarabine
YPL218W SAR1
ZShift = −1.93
NG = 0
DB = 0
SM = 2
Delta_K
Cytarabine
YPL218W SAR1
ZShift = −4.36
NG = 0
DB = 0
SM = 2
Delta_r
Cytarabine
YPL218W SAR1
ZShift = −5.03
NG = 0
DB = 0
SM = 2
Delta_AUC
Cytarabine
YDL045C FAD1
ZShift = 4.32
NG = 0
DB = 0
SM = 0
Delta_L
Cytarabine
YDL045C FAD1
ZShift = −0.8
NG = 0
DB = 0
SM = 0
Delta_K
Cytarabine
YDL045C FAD1
ZShift = −2.92
NG = 0
DB = 0
SM = 0
Delta_r
Cytarabine
YDL045C FAD1
ZShift = −2.36
NG = 0
DB = 0
SM = 0
Delta_AUC
Cytarabine

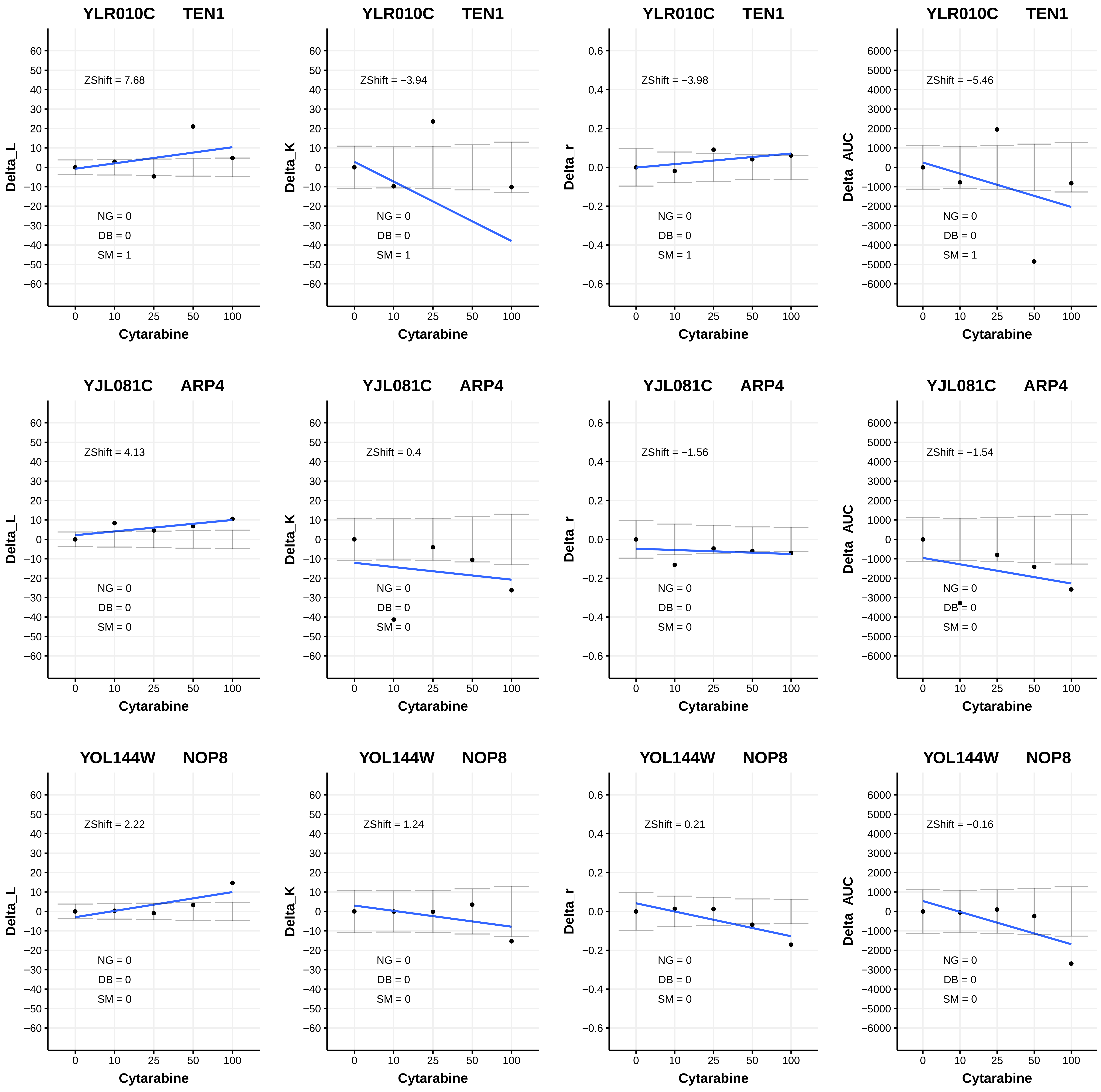

YLR010C TEN1
ZShift = 7.68
NG = 0
DB = 0
SM = 1
Delta_L
Cytarabine
YLR010C TEN1
ZShift = −3.94
NG = 0
DB = 0
SM = 1
Delta_K
Cytarabine
YLR010C TEN1
ZShift = −3.98
NG = 0
DB = 0
SM = 1
Delta_r
Cytarabine
YLR010C TEN1
ZShift = −5.46
NG = 0
DB = 0
SM = 1
Delta_AUC
Cytarabine
YJL081C ARP4
ZShift = 4.13
NG = 0
DB = 0
SM = 0
Delta_L
Cytarabine
YJL081C ARP4
ZShift = 0.4
NG = 0
DB = 0
SM = 0
Delta_K
Cytarabine
YJL081C ARP4
ZShift = −1.56
NG = 0
DB = 0
SM = 0
Delta_r
Cytarabine
YJL081C ARP4
ZShift = −1.54
NG = 0
DB = 0
SM = 0
Delta_AUC
Cytarabine
YOL144W NOP8
ZShift = 2.22
NG = 0
DB = 0
SM = 0
Delta_L
Cytarabine
YOL144W NOP8
ZShift = 1.24
NG = 0
DB = 0
SM = 0
Delta_K
Cytarabine
YOL144W NOP8
ZShift = 0.21
NG = 0
DB = 0
SM = 0
Delta_r
Cytarabine
YOL144W NOP8
ZShift = −0.16
NG = 0
DB = 0
SM = 0
Delta_AUC
Cytarabine

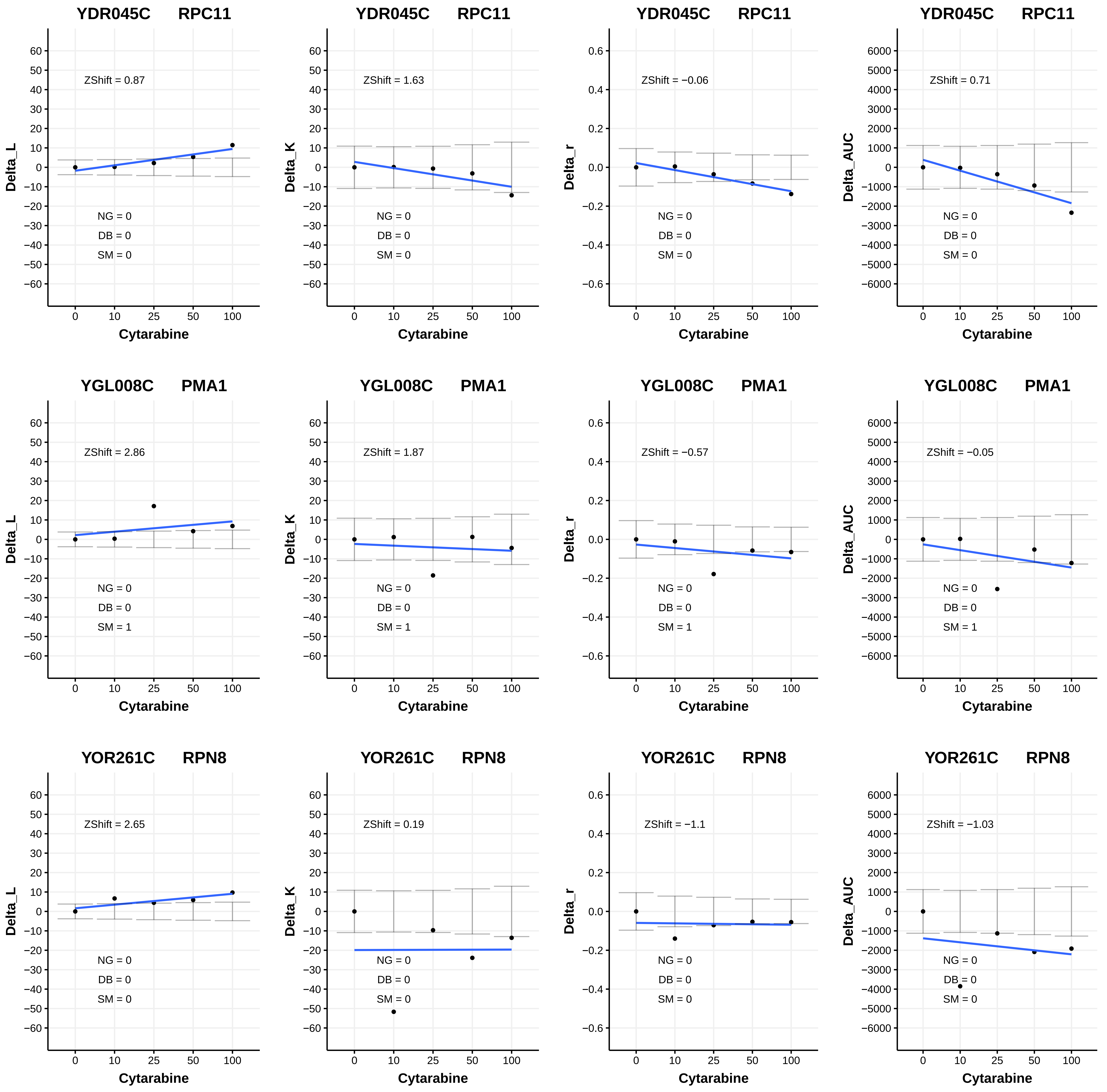
YDR045C RPC11
Delta_L
ZShift = 0.87
NG = 0
DB = 0
SM = 0
Cytarabine
YDR045C RPC11
Delta_K
ZShift = 1.63
NG = 0
DB = 0
SM = 0
Cytarabine
YDR045C RPC11
Delta_r
ZShift = −0.06
NG = 0
DB = 0
SM = 0
Cytarabine
YDR045C RPC11
Delta_AUC
ZShift = 0.71
NG = 0
DB = 0
SM = 0
Cytarabine
YGL008C PMA1
Delta_L
ZShift = 2.86
NG = 0
DB = 0
SM = 1
Cytarabine
YGL008C PMA1
Delta_K
ZShift = 1.87
NG = 0
DB = 0
SM = 1
Cytarabine
YGL008C PMA1
Delta_r
ZShift = −0.57
NG = 0
DB = 0
SM = 1
Cytarabine
YGL008C PMA1
Delta_AUC
ZShift = −0.05
NG = 0
DB = 0
SM = 1
Cytarabine
YOR261C RPN8
Delta_L
ZShift = 2.65
NG = 0
DB = 0
SM = 0
Cytarabine
YOR261C RPN8
Delta_K
ZShift = 0.19
NG = 0
DB = 0
SM = 0
Cytarabine
YOR261C RPN8
Delta_r
ZShift = −1.1
NG = 0
DB = 0
SM = 0
Cytarabine
YOR261C RPN8
Delta_AUC
ZShift = −1.03
NG = 0
DB = 0
SM = 0
Cytarabine
0 10 25 50 100

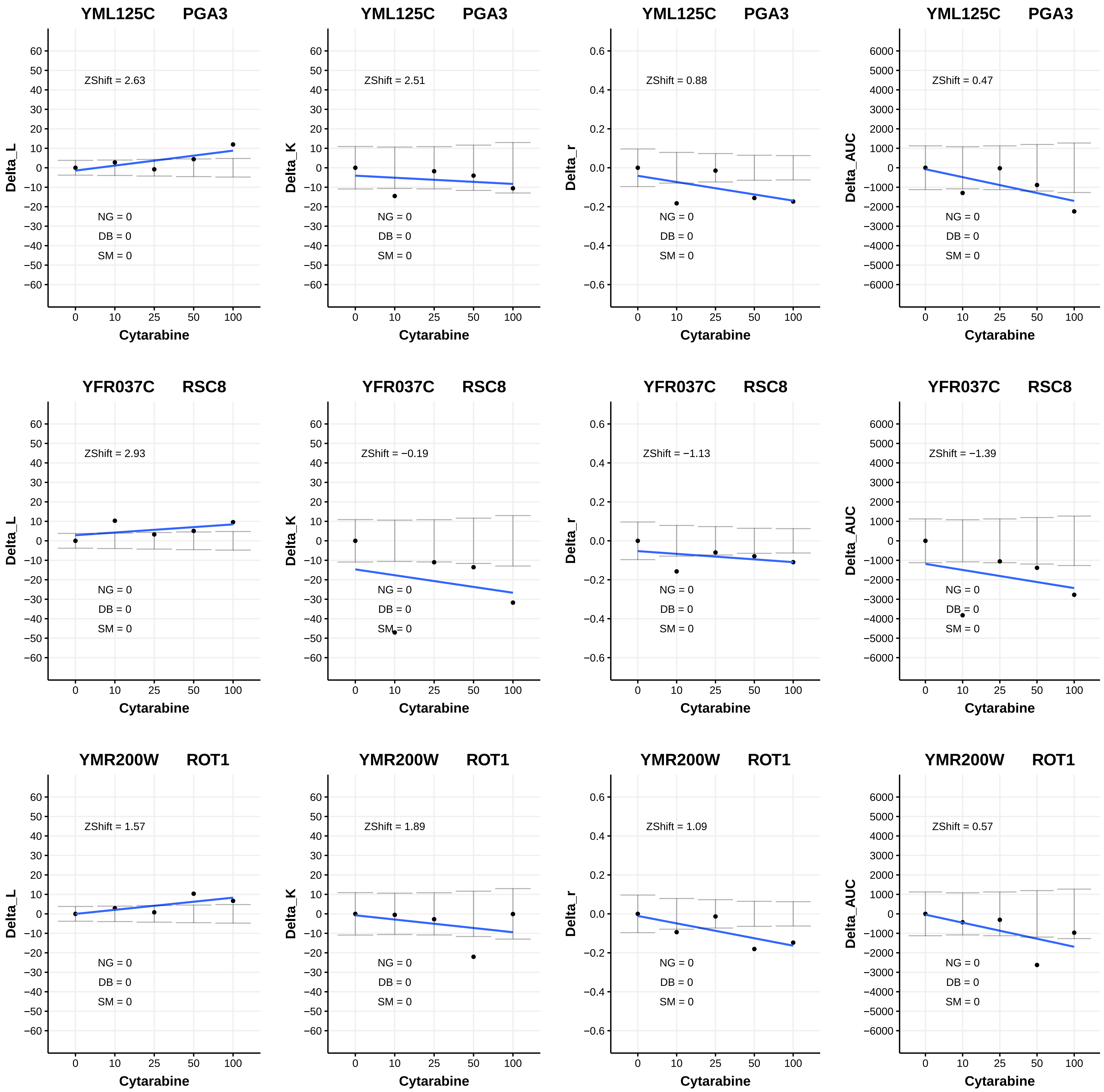

YML125C PGA3
ZShift = 2.63
ZShift = 2.51
ZShift = 0.88
ZShift = 0.47
YFR037C RSC8
ZShift = 2.93
ZShift = −0.19
ZShift = −1.13
ZShift = −1.39
YMR200W ROT1
ZShift = 1.57
ZShift = 1.89
ZShift = 1.09
ZShift = 0.57
NG = 0
DB = 0
SM = 0
Delta_L
Delta_K
Delta_r
Delta_AUC
Cytarabine
0 10 25 50 100

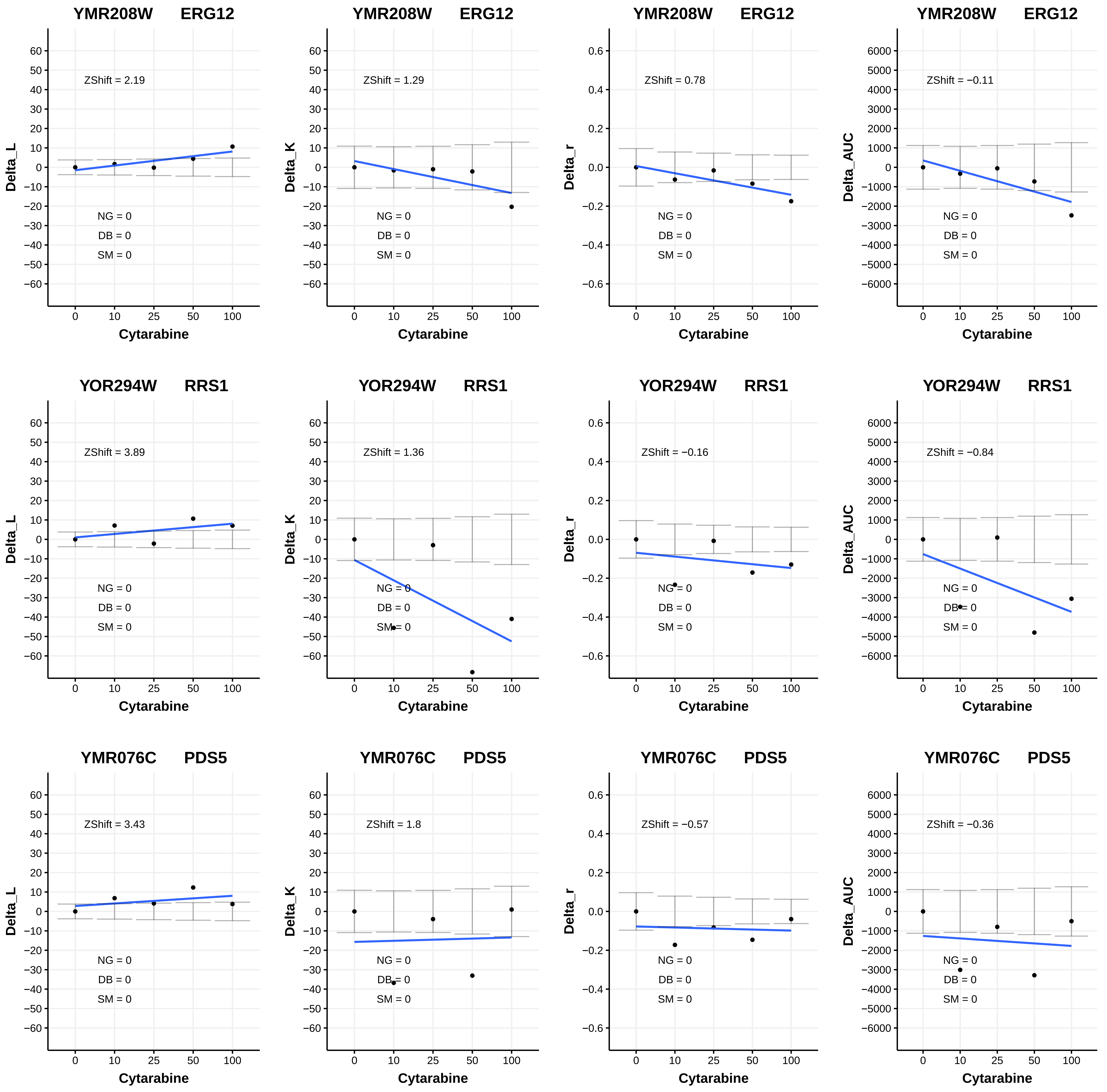

YMR208W ERG12
ZShift = 2.19
NG = 0
DB = 0
SM = 0
Delta_L
Cytarabine
YMR208W ERG12
ZShift = 1.29
NG = 0
DB = 0
SM = 0
Delta_K
Cytarabine
YMR208W ERG12
ZShift = 0.78
NG = 0
DB = 0
SM = 0
Delta_r
Cytarabine
YMR208W ERG12
ZShift = −0.11
NG = 0
DB = 0
SM = 0
Delta_AUC
Cytarabine
YOR294W RRS1
ZShift = 3.89
NG = 0
DB = 0
SM = 0
Delta_L
Cytarabine
YOR294W RRS1
ZShift = 1.36
NG = 0
DB = 0
SM = 0
Delta_K
Cytarabine
YOR294W RRS1
ZShift = −0.16
NG = 0
DB = 0
SM = 0
Delta_r
Cytarabine
YOR294W RRS1
ZShift = −0.84
NG = 0
DB = 0
SM = 0
Delta_AUC
Cytarabine
YMR076C PDS5
ZShift = 3.43
NG = 0
DB = 0
SM = 0
Delta_L
Cytarabine
YMR076C PDS5
ZShift = 1.8
NG = 0
DB = 0
SM = 0
Delta_K
Cytarabine
YMR076C PDS5
ZShift = −0.57
NG = 0
DB = 0
SM = 0
Delta_r
Cytarabine
YMR076C PDS5
ZShift = −0.36
NG = 0
DB = 0
SM = 0
Delta_AUC
Cytarabine

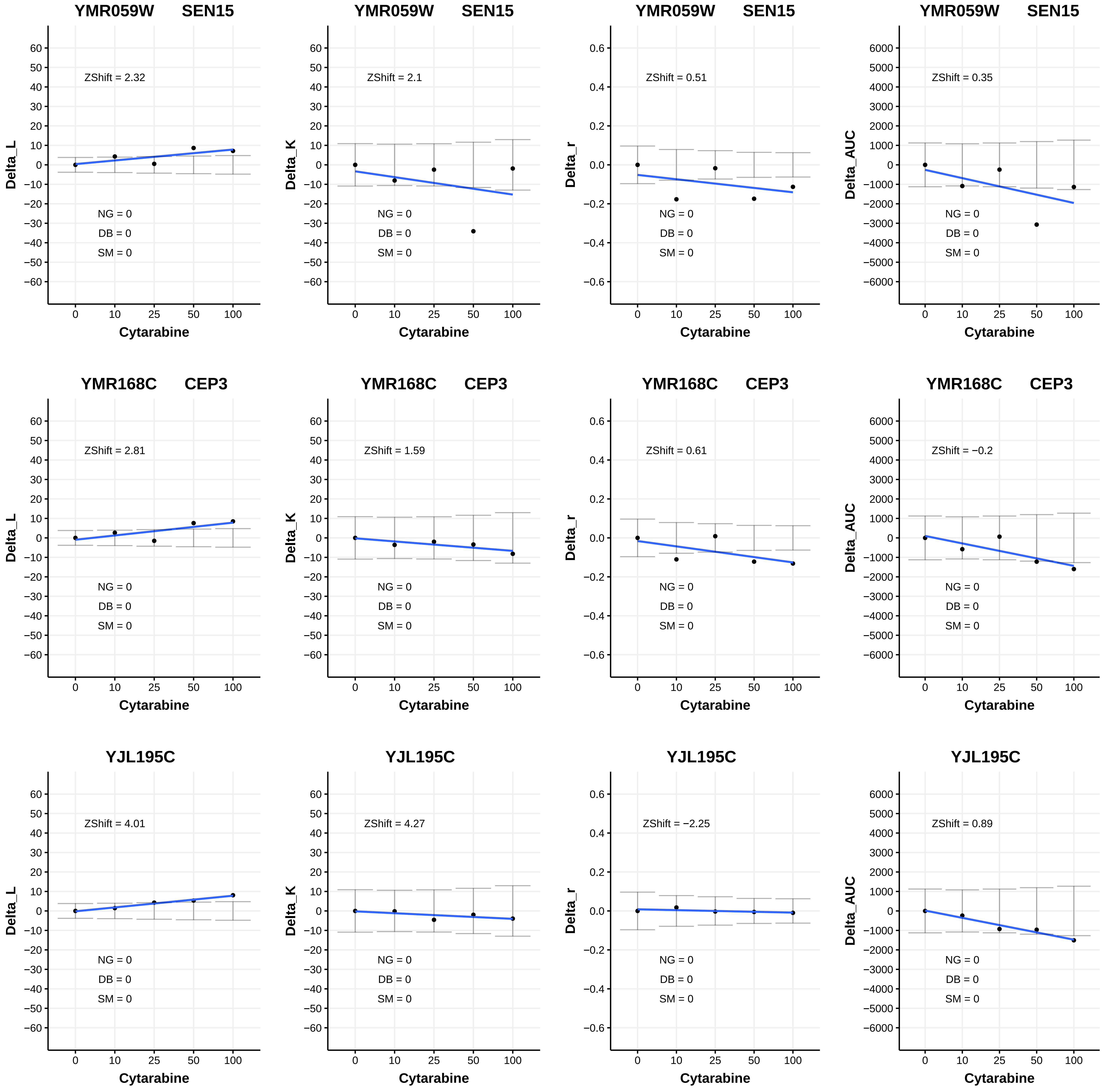
YMR059W SEN15
ZShift = 2.32
ZShift = 2.1
ZShift = 0.51
ZShift = 0.35
YMR168C CEP3
ZShift = 2.81
ZShift = 1.59
ZShift = 0.61
ZShift = −0.2
YJL195C
ZShift = 4.01
ZShift = 4.27
ZShift = −2.25
ZShift = 0.89
NG = 0
DB = 0
SM = 0
Delta_L
Delta_K
Delta_r
Delta_AUC
Cytarabine
0
10
25
50
100

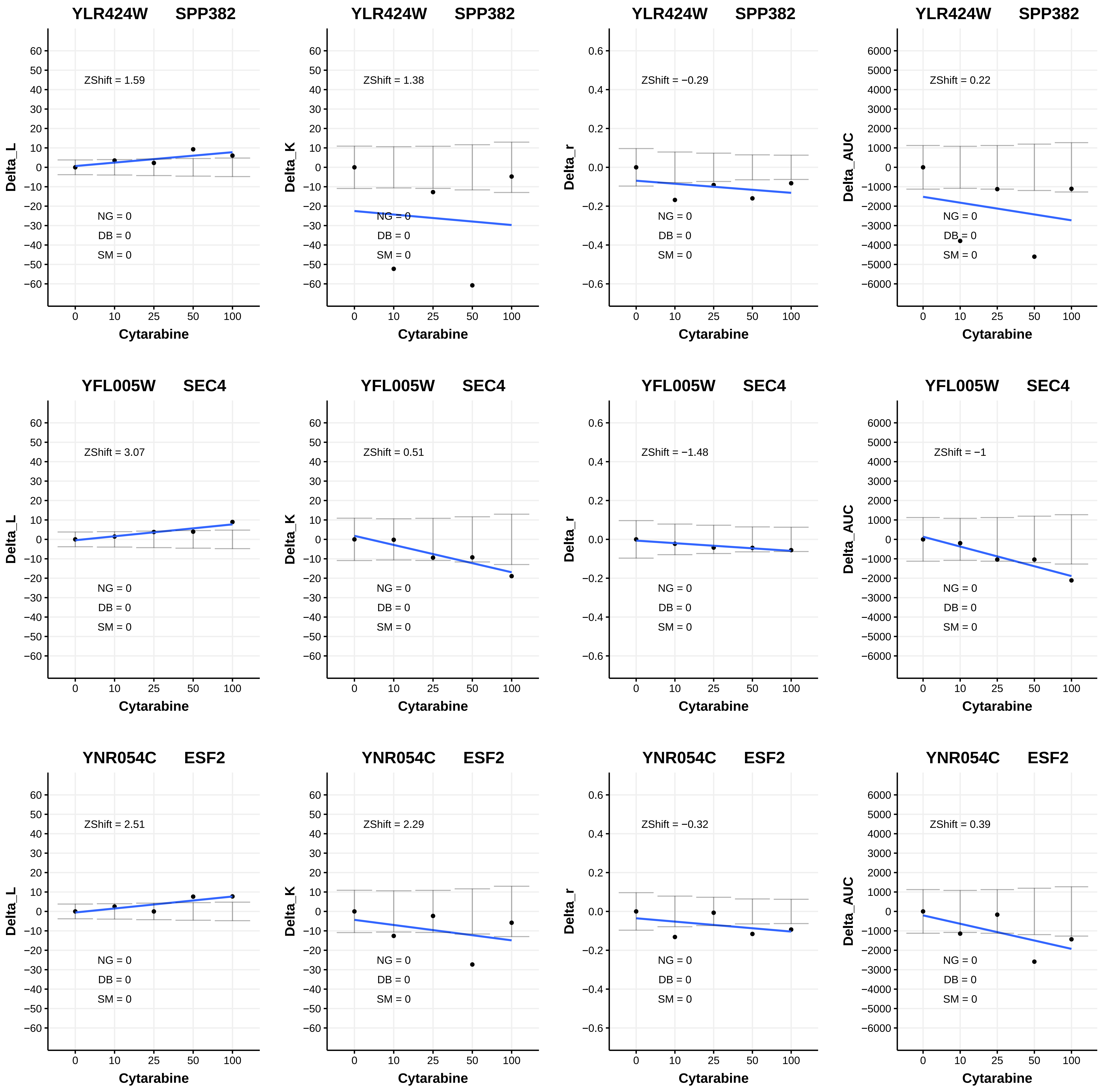
YLR424W SPP382
ZShift = 1.59
ZShift = 1.38
ZShift = −0.29
ZShift = 0.22
YFL005W SEC4
ZShift = 3.07
ZShift = 0.51
ZShift = −1.48
ZShift = −1
YNR054C ESF2
ZShift = 2.51
ZShift = 2.29
ZShift = −0.32
ZShift = 0.39
NG = 0
DB = 0
SM = 0
Delta_L
Delta_K
Delta_r
Delta_AUC
Cytarabine

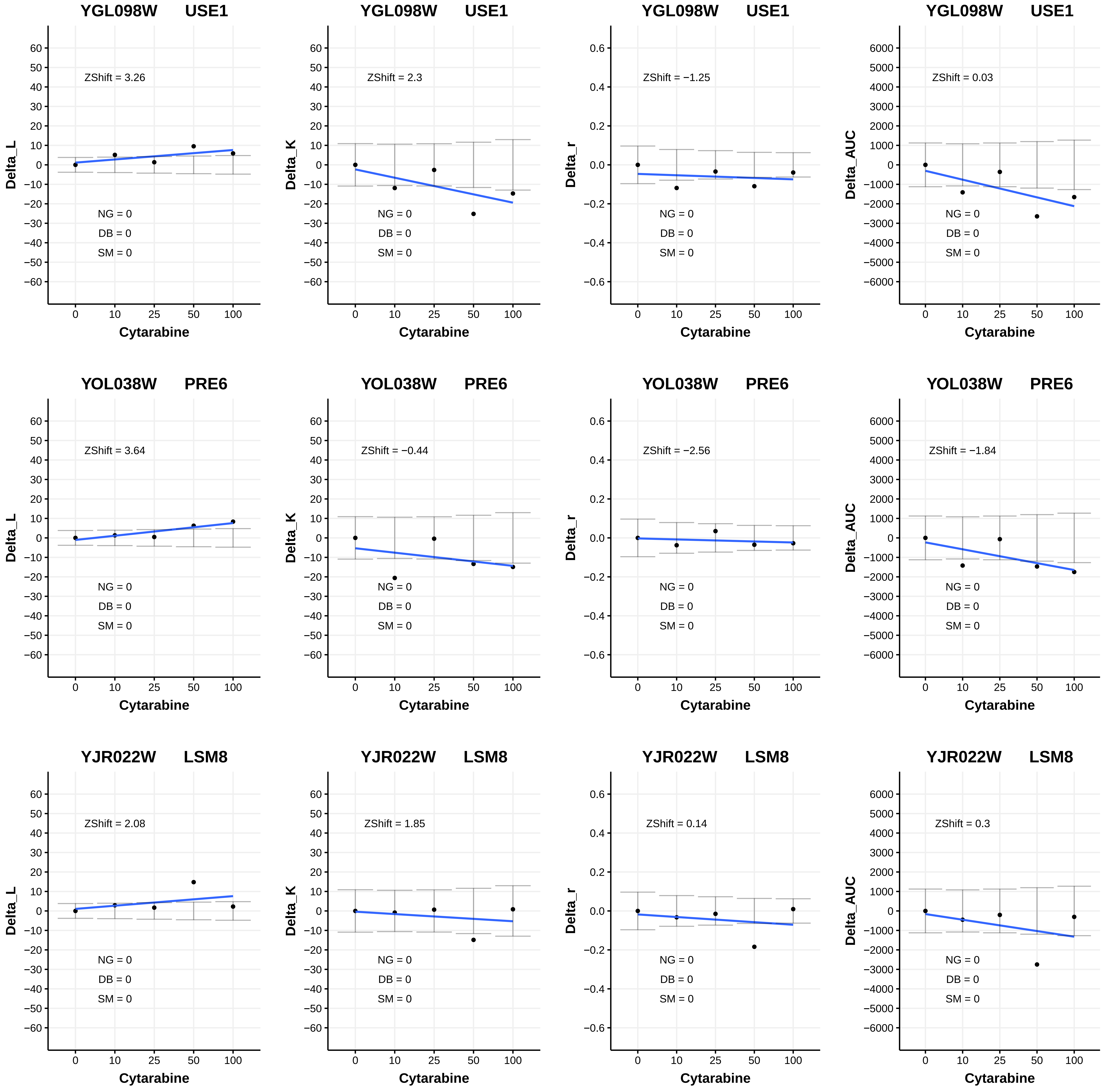
YGL098W USE1
ZShift = 3.26
NG = 0
DB = 0
SM = 0
Delta_L
Cytarabine
YGL098W USE1
ZShift = 2.3
NG = 0
DB = 0
SM = 0
Delta_K
Cytarabine
YGL098W USE1
ZShift = −1.25
NG = 0
DB = 0
SM = 0
Delta_r
Cytarabine
YGL098W USE1
ZShift = 0.03
NG = 0
DB = 0
SM = 0
Delta_AUC
Cytarabine
YOL038W PRE6
ZShift = 3.64
NG = 0
DB = 0
SM = 0
Delta_L
Cytarabine
YOL038W PRE6
ZShift = −0.44
NG = 0
DB = 0
SM = 0
Delta_K
Cytarabine
YOL038W PRE6
ZShift = −2.56
NG = 0
DB = 0
SM = 0
Delta_r
Cytarabine
YOL038W PRE6
ZShift = −1.84
NG = 0
DB = 0
SM = 0
Delta_AUC
Cytarabine
YJR022W LSM8
ZShift = 2.08
NG = 0
DB = 0
SM = 0
Delta_L
Cytarabine
YJR022W LSM8
ZShift = 1.85
NG = 0
DB = 0
SM = 0
Delta_K
Cytarabine
YJR022W LSM8
ZShift = 0.14
NG = 0
DB = 0
SM = 0
Delta_r
Cytarabine
YJR022W LSM8
ZShift = 0.3
NG = 0
DB = 0
SM = 0
Delta_AUC
Cytarabine

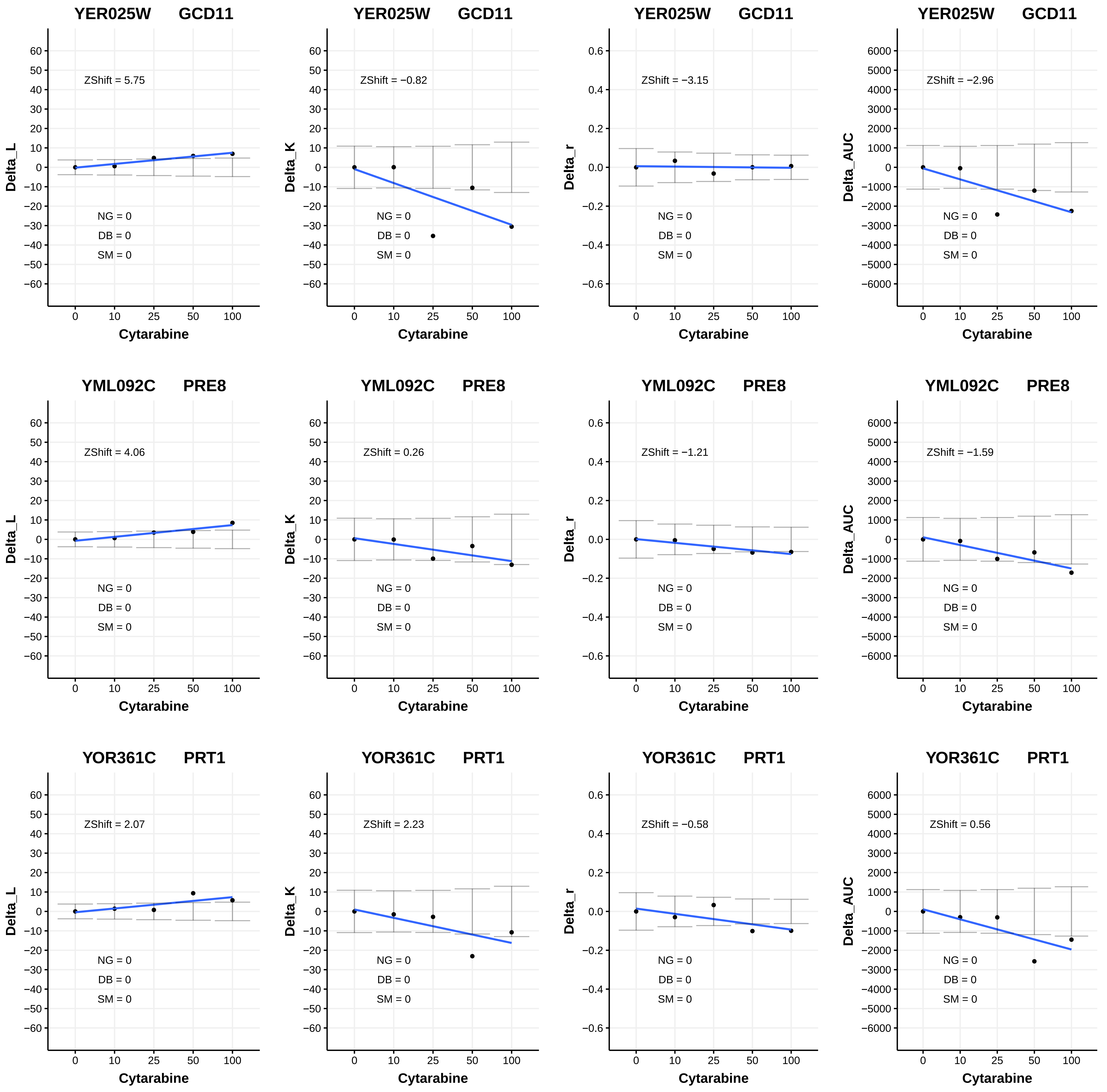
YER025W GCD11
ZShift = 5.75
NG = 0
DB = 0
SM = 0
Delta_L
Cytarabine
YER025W GCD11
ZShift = −0.82
NG = 0
DB = 0
SM = 0
Delta_K
Cytarabine
YER025W GCD11
ZShift = −3.15
NG = 0
DB = 0
SM = 0
Delta_r
Cytarabine
YER025W GCD11
ZShift = −2.96
NG = 0
DB = 0
SM = 0
Delta_AUC
Cytarabine
YML092C PRE8
ZShift = 4.06
NG = 0
DB = 0
SM = 0
Delta_L
Cytarabine
YML092C PRE8
ZShift = 0.26
NG = 0
DB = 0
SM = 0
Delta_K
Cytarabine
YML092C PRE8
ZShift = −1.21
NG = 0
DB = 0
SM = 0
Delta_r
Cytarabine
YML092C PRE8
ZShift = −1.59
NG = 0
DB = 0
SM = 0
Delta_AUC
Cytarabine
YOR361C PRT1
ZShift = 2.07
NG = 0
DB = 0
SM = 0
Delta_L
Cytarabine
YOR361C PRT1
ZShift = 2.23
NG = 0
DB = 0
SM = 0
Delta_K
Cytarabine
YOR361C PRT1
ZShift = −0.58
NG = 0
DB = 0
SM = 0
Delta_r
Cytarabine
YOR361C PRT1
ZShift = 0.56
NG = 0
DB = 0
SM = 0
Delta_AUC
Cytarabine

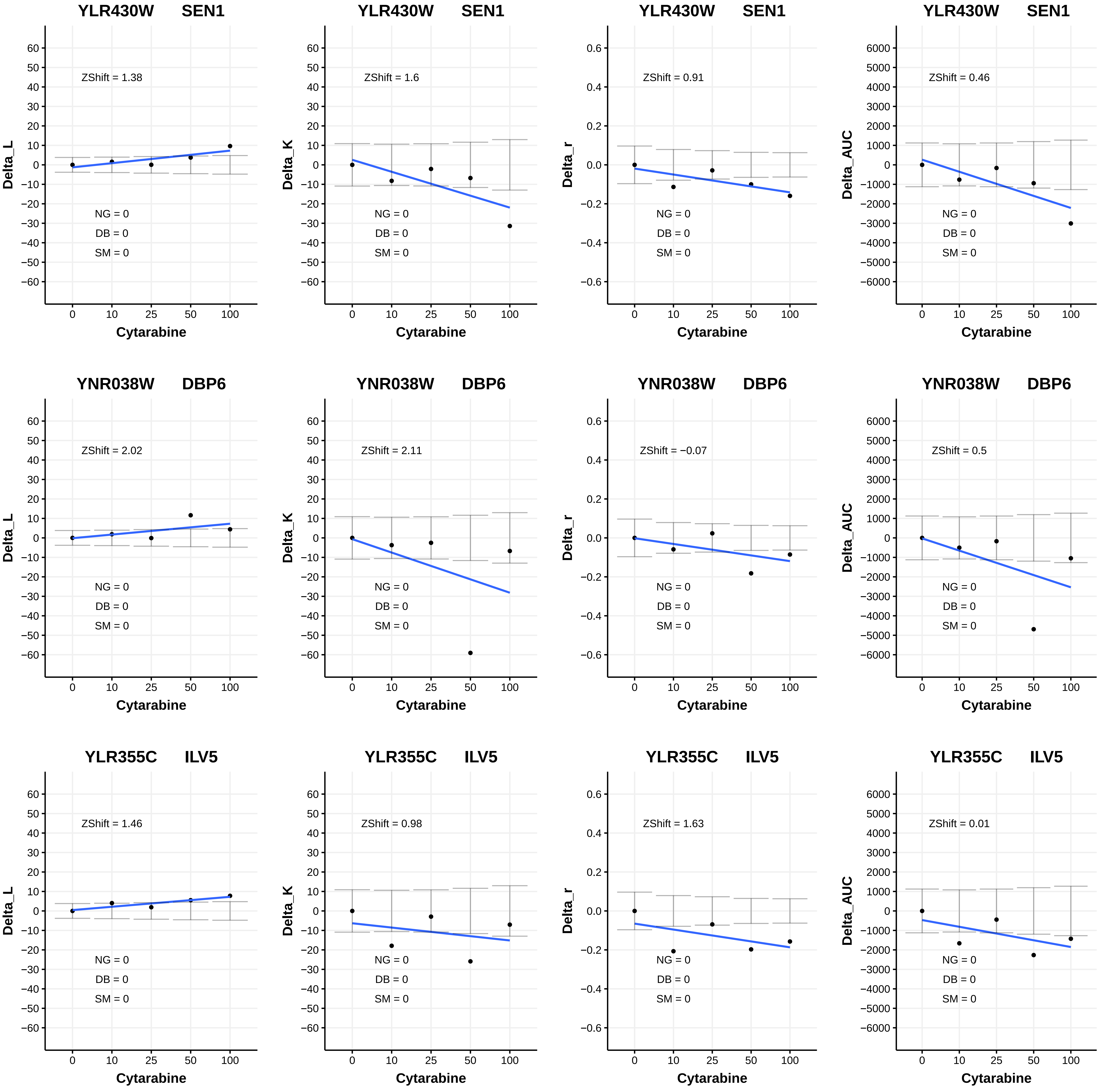

YLR430W SEN1
ZShift = 1.38
ZShift = 1.6
ZShift = 0.91
ZShift = 0.46
YNR038W DBP6
ZShift = 2.02
ZShift = 2.11
ZShift = −0.07
ZShift = 0.5
YLR355C ILV5
ZShift = 1.46
ZShift = 0.98
ZShift = 1.63
ZShift = 0.01
NG = 0
DB = 0
SM = 0
Delta_L
Delta_K
Delta_r
Delta_AUC
Cytarabine

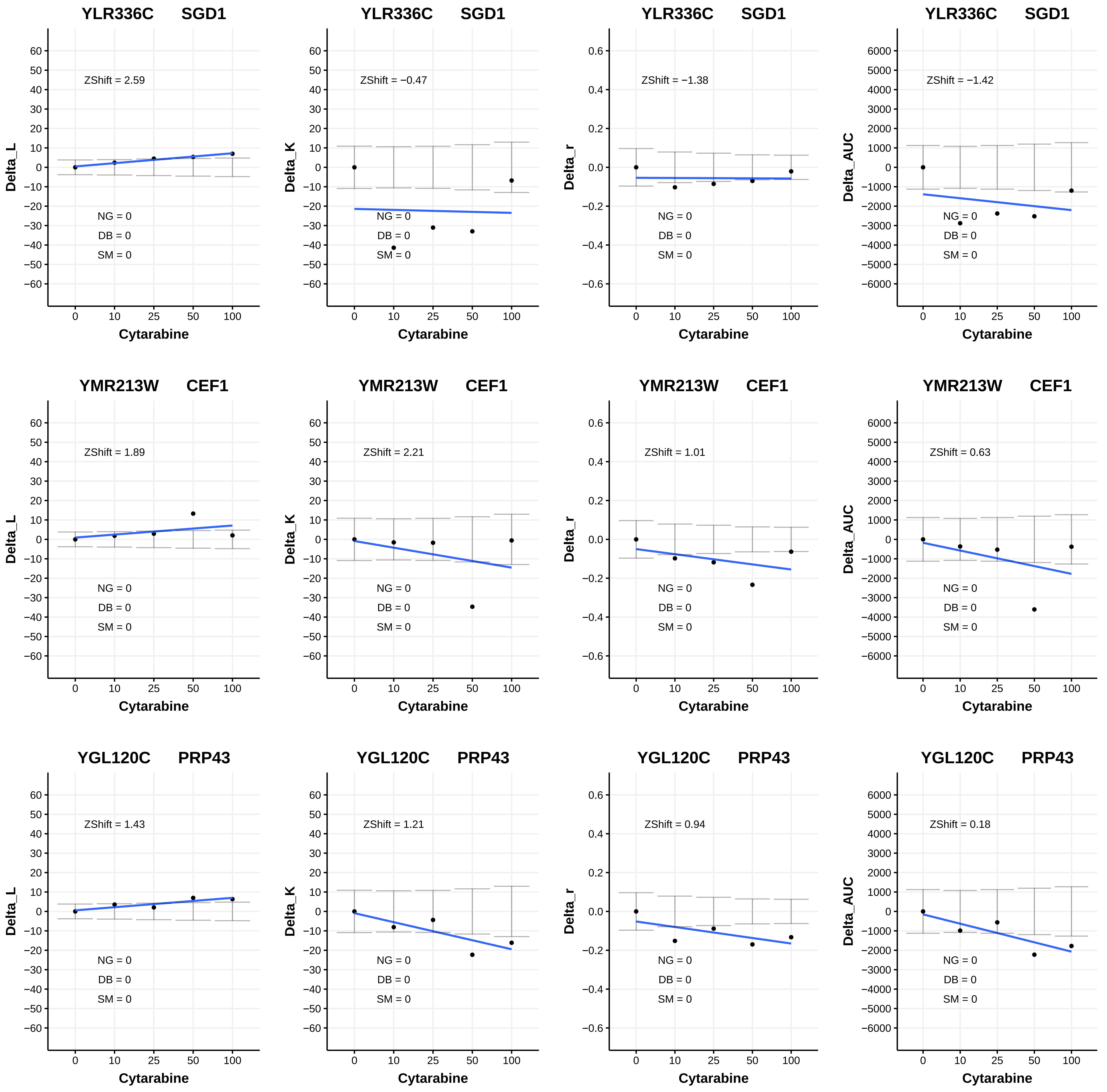
YLR336C SGD1
ZShift = 2.59
NG = 0
DB = 0
SM = 0
Delta_L
Cytarabine
YLR336C SGD1
ZShift = −0.47
NG = 0
DB = 0
SM = 0
Delta_K
Cytarabine
YLR336C SGD1
ZShift = −1.38
NG = 0
DB = 0
SM = 0
Delta_r
Cytarabine
YLR336C SGD1
ZShift = −1.42
NG = 0
DB = 0
SM = 0
Delta_AUC
Cytarabine
YMR213W CEF1
ZShift = 1.89
NG = 0
DB = 0
SM = 0
Delta_L
Cytarabine
YMR213W CEF1
ZShift = 2.21
NG = 0
DB = 0
SM = 0
Delta_K
Cytarabine
YMR213W CEF1
ZShift = 1.01
NG = 0
DB = 0
SM = 0
Delta_r
Cytarabine
YMR213W CEF1
ZShift = 0.63
NG = 0
DB = 0
SM = 0
Delta_AUC
Cytarabine
YGL120C PRP43
ZShift = 1.43
NG = 0
DB = 0
SM = 0
Delta_L
Cytarabine
YGL120C PRP43
ZShift = 1.21
NG = 0
DB = 0
SM = 0
Delta_K
Cytarabine
YGL120C PRP43
ZShift = 0.94
NG = 0
DB = 0
SM = 0
Delta_r
Cytarabine
YGL120C PRP43
ZShift = 0.18
NG = 0
DB = 0
SM = 0
Delta_AUC
Cytarabine

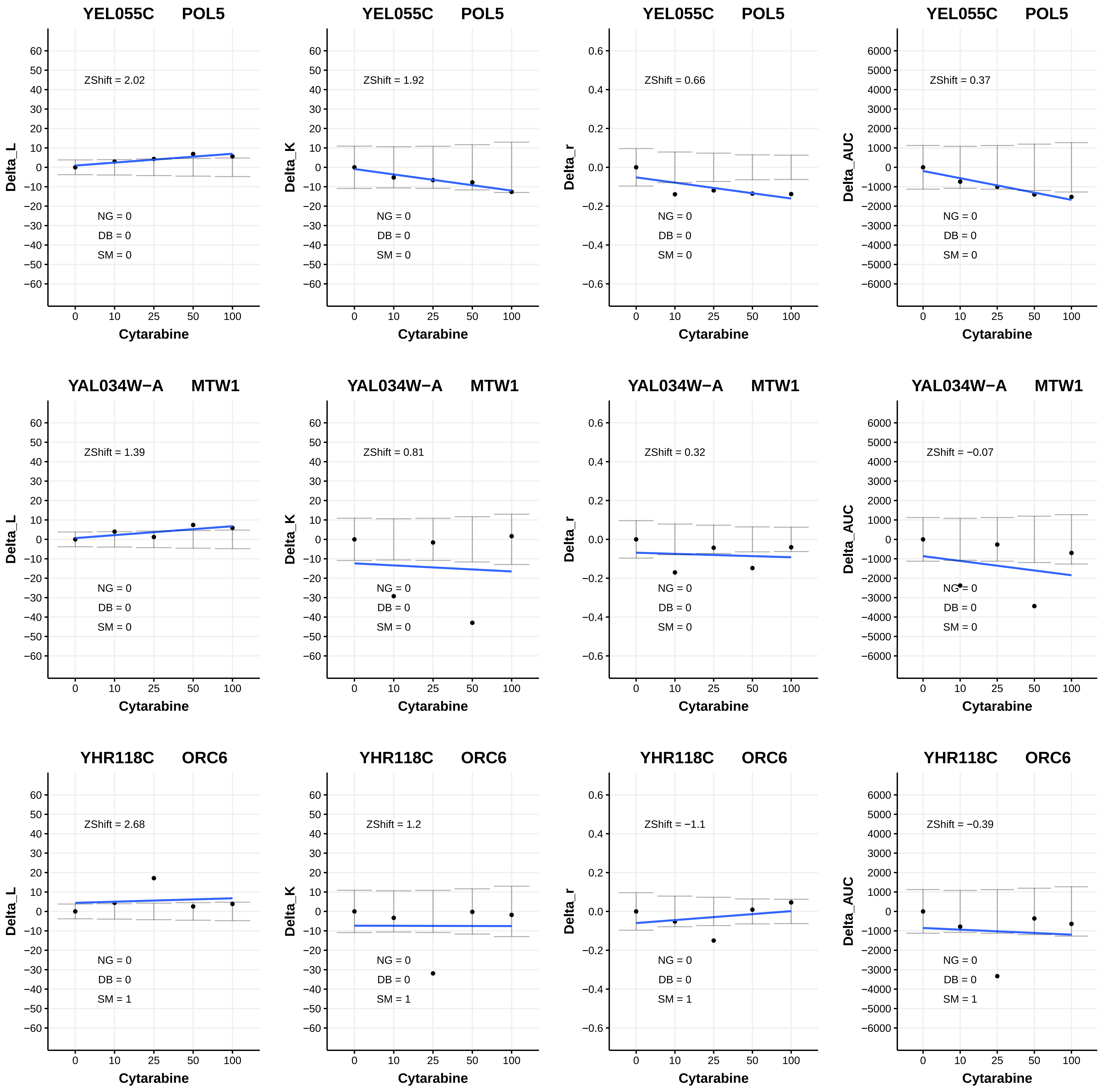
YEL055C POL5
ZShift = 2.02
ZShift = 1.92
ZShift = 0.66
ZShift = 0.37
Delta_L
Delta_K
Delta_r
Delta_AUC
NG = 0
DB = 0
SM = 0
Cytarabine
0 10 25 50 100
YAL034W−A MTW1
ZShift = 1.39
ZShift = 0.81
ZShift = 0.32
ZShift = −0.07
NG = 0
DB = 0
SM = 0
YHR118C ORC6
ZShift = 2.68
ZShift = 1.2
ZShift = −1.1
ZShift = −0.39
NG = 0
DB = 0
SM = 1

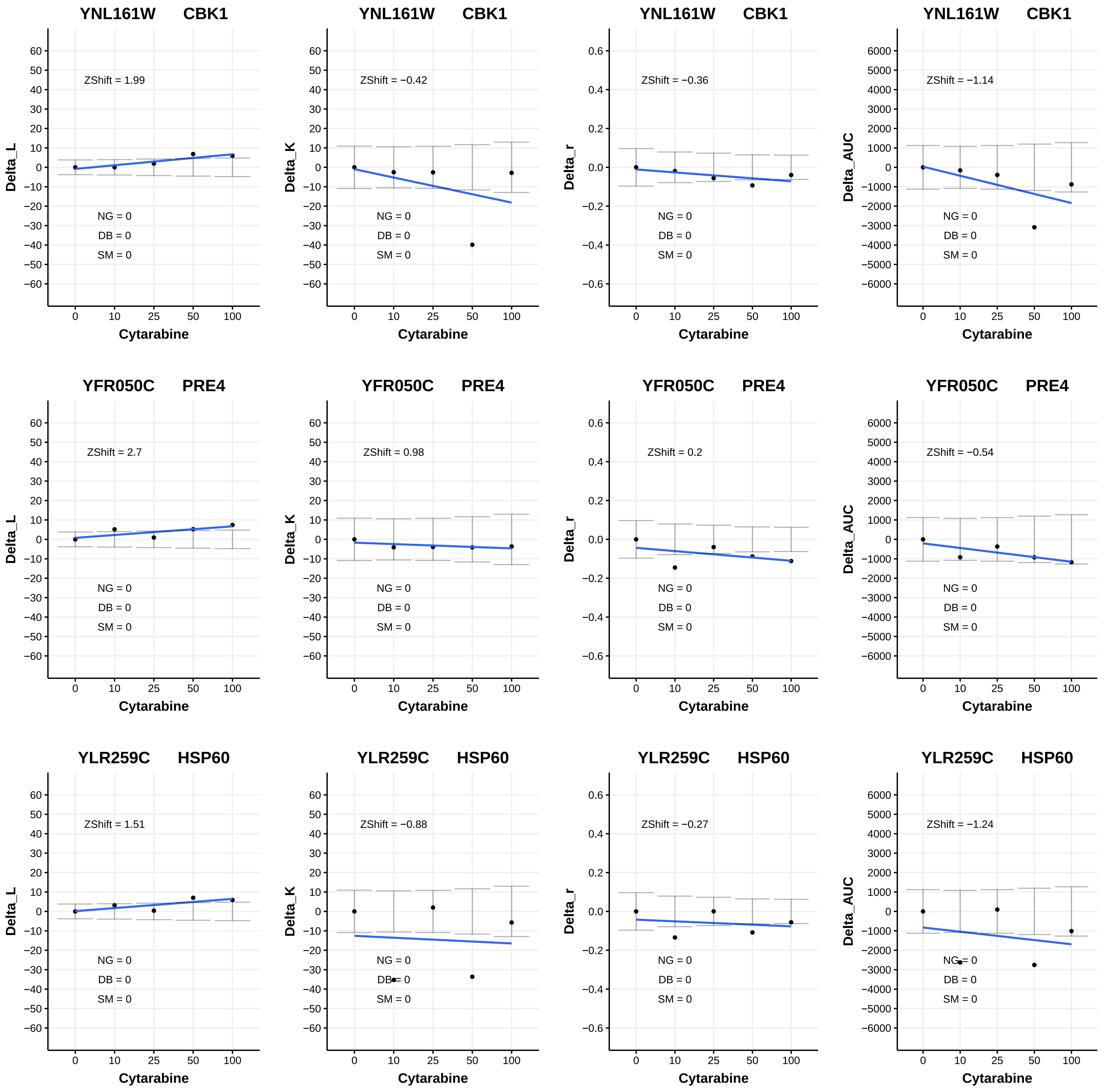
YNL161W CBK1
ZShift = 1.99
ZShift = −0.42
ZShift = −0.36
ZShift = −1.14
YFR050C PRE4
ZShift = 2.7
ZShift = 0.98
ZShift = 0.2
ZShift = −0.54
YLR259C HSP60
ZShift = 1.51
ZShift = −0.88
ZShift = −0.27
ZShift = −1.24
NG = 0
DB = 0
SM = 0
Delta_L
Delta_K
Delta_r
Delta_AUC
Cytarabine
0
10
25
50
100

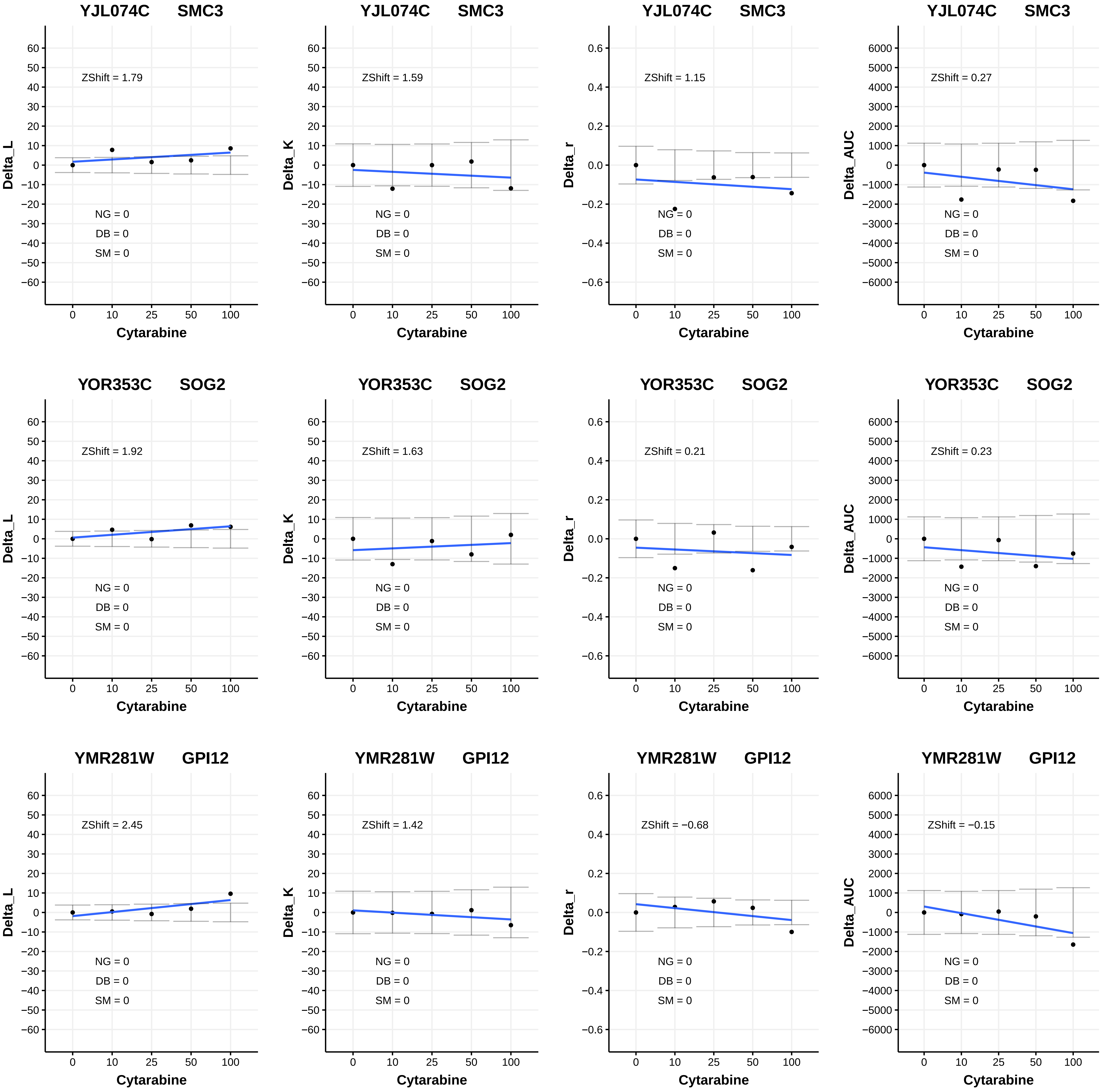
YJL074C SMC3
ZShift = 1.79
ZShift = 1.59
ZShift = 1.15
ZShift = 0.27
YOR353C SOG2
ZShift = 1.92
ZShift = 1.63
ZShift = 0.21
ZShift = 0.23
YMR281W GPI12
ZShift = 2.45
ZShift = 1.42
ZShift = −0.68
ZShift = −0.15
NG = 0
DB = 0
SM = 0
Delta_L
Delta_K
Delta_r
Delta_AUC
Cytarabine
0
10
25
50
100

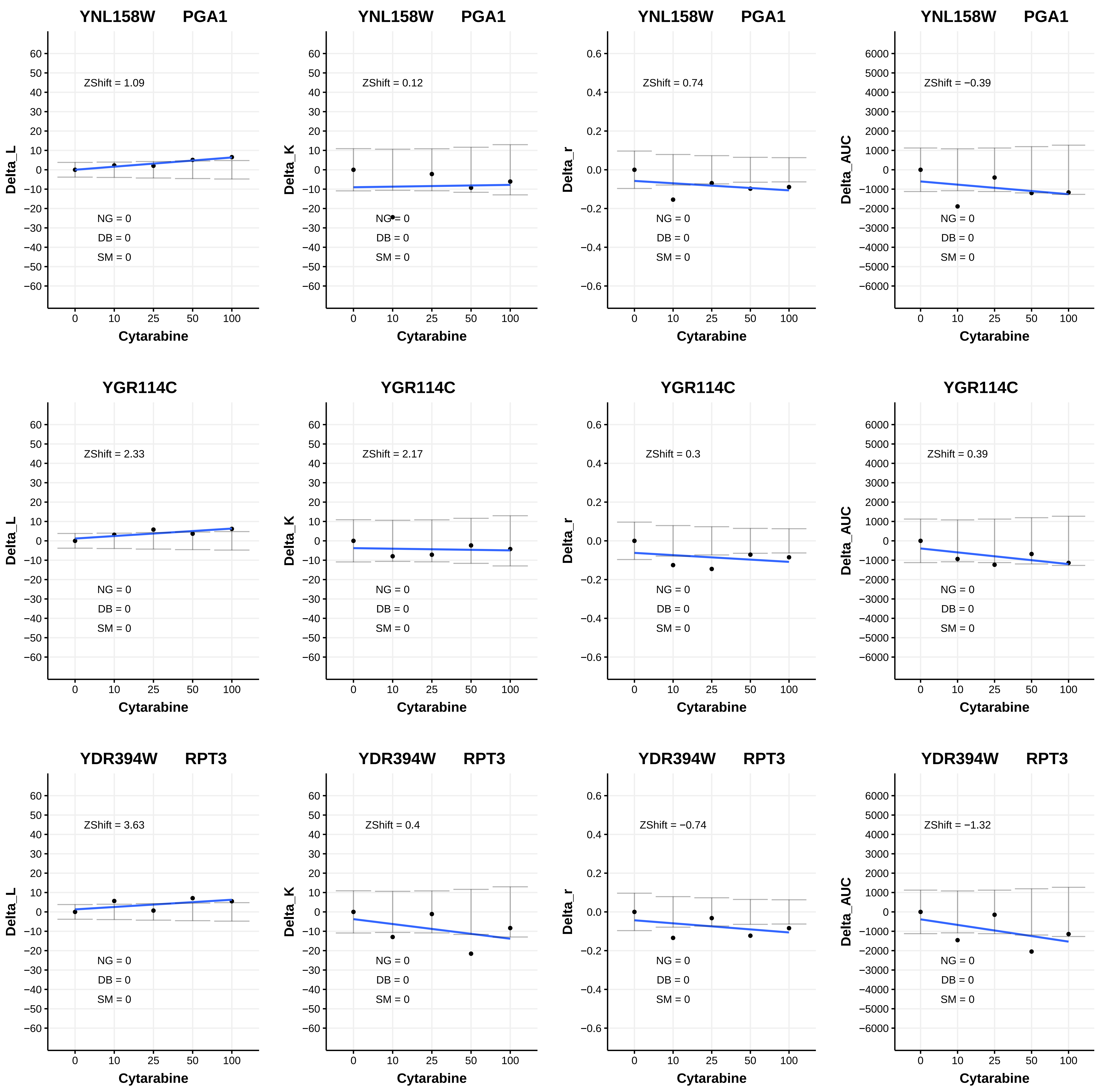

YNL158W PGA1
ZShift = 1.09
ZShift = 0.12
ZShift = 0.74
ZShift = −0.39
YGR114C
ZShift = 2.33
ZShift = 2.17
ZShift = 0.3
ZShift = 0.39
YDR394W RPT3
ZShift = 3.63
ZShift = 0.4
ZShift = −0.74
ZShift = −1.32
NG = 0
DB = 0
SM = 0
Delta_L
Delta_K
Delta_r
Delta_AUC
Cytarabine
0 10 25 50 100

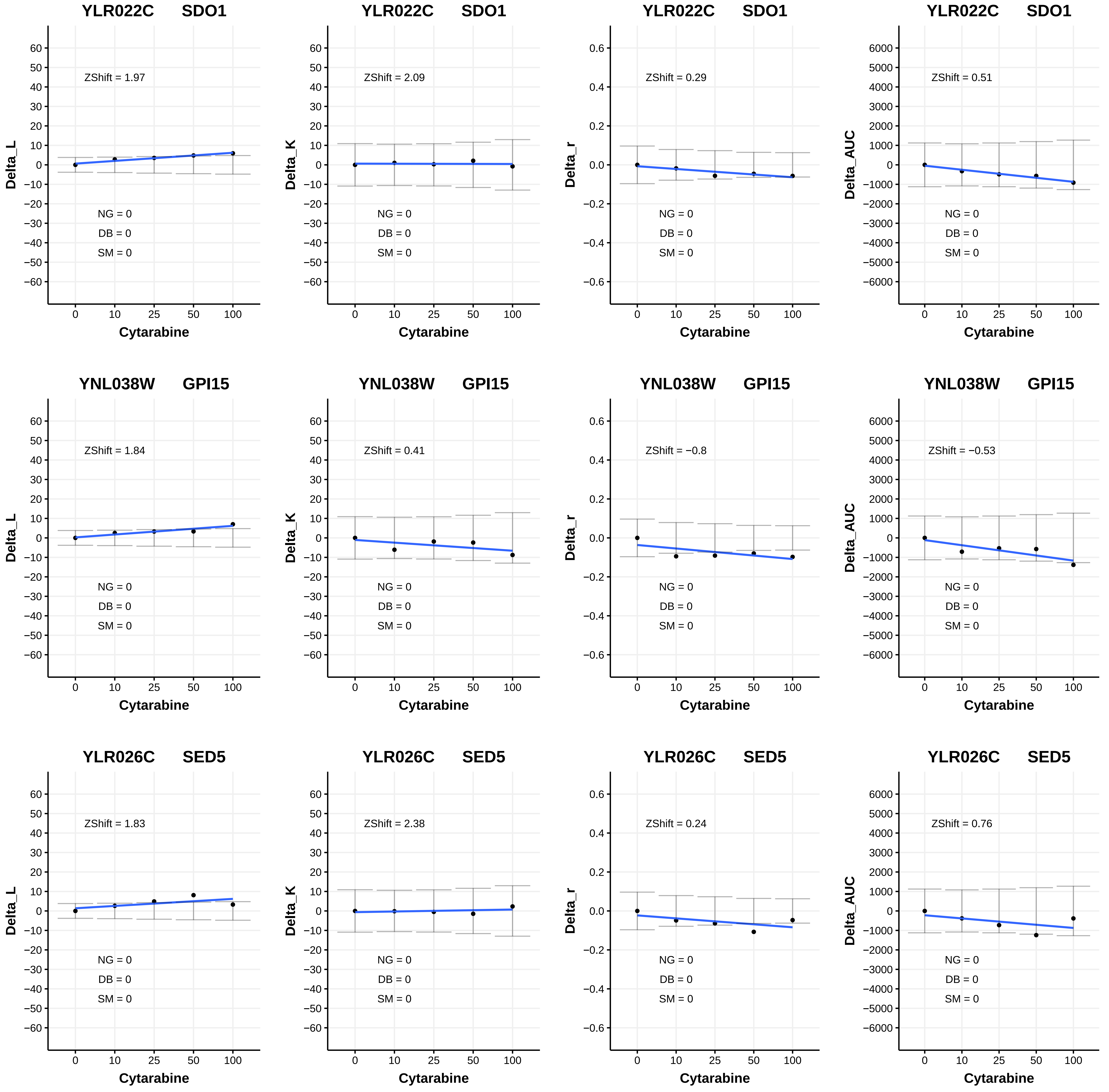
YLR022C SDO1
ZShift = 1.97
NG = 0
DB = 0
SM = 0
Delta_L
Cytarabine
YLR022C SDO1
ZShift = 2.09
NG = 0
DB = 0
SM = 0
Delta_K
Cytarabine
YLR022C SDO1
ZShift = 0.29
NG = 0
DB = 0
SM = 0
Delta_r
Cytarabine
YLR022C SDO1
ZShift = 0.51
NG = 0
DB = 0
SM = 0
Delta_AUC
Cytarabine
YNL038W GPI15
ZShift = 1.84
NG = 0
DB = 0
SM = 0
Delta_L
Cytarabine
YNL038W GPI15
ZShift = 0.41
NG = 0
DB = 0
SM = 0
Delta_K
Cytarabine
YNL038W GPI15
ZShift = −0.8
NG = 0
DB = 0
SM = 0
Delta_r
Cytarabine
YNL038W GPI15
ZShift = −0.53
NG = 0
DB = 0
SM = 0
Delta_AUC
Cytarabine
YLR026C SED5
ZShift = 1.83
NG = 0
DB = 0
SM = 0
Delta_L
Cytarabine
YLR026C SED5
ZShift = 2.38
NG = 0
DB = 0
SM = 0
Delta_K
Cytarabine
YLR026C SED5
ZShift = 0.24
NG = 0
DB = 0
SM = 0
Delta_r
Cytarabine
YLR026C SED5
ZShift = 0.76
NG = 0
DB = 0
SM = 0
Delta_AUC
Cytarabine

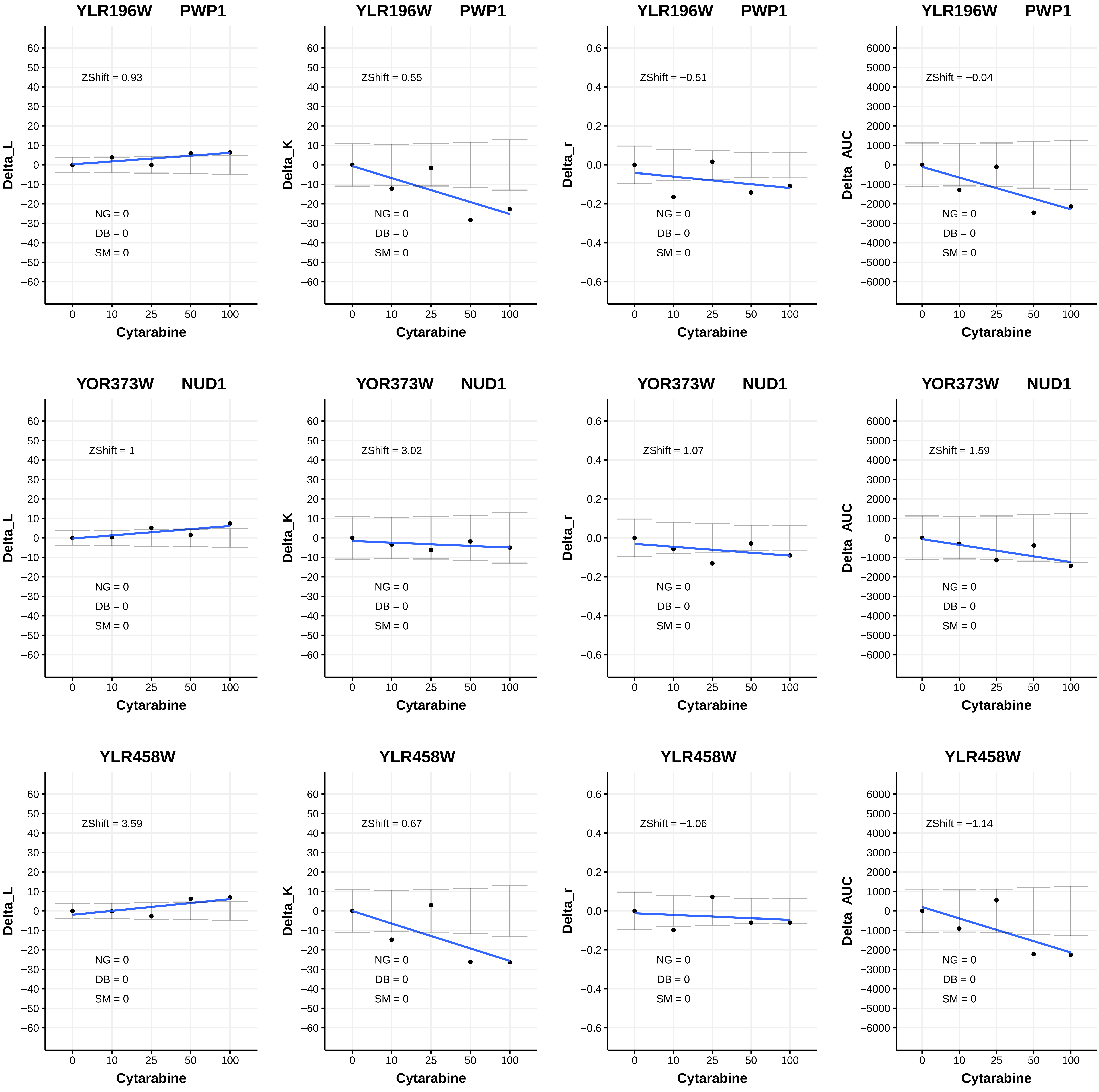

YLR196W PWP1
ZShift = 0.93
Delta_L
NG = 0
DB = 0
SM = 0
Cytarabine
YLR196W PWP1
ZShift = 0.55
Delta_K
NG = 0
DB = 0
SM = 0
Cytarabine
YLR196W PWP1
ZShift = −0.51
Delta_r
NG = 0
DB = 0
SM = 0
Cytarabine
YLR196W PWP1
ZShift = −0.04
Delta_AUC
NG = 0
DB = 0
SM = 0
Cytarabine
YOR373W NUD1
ZShift = 1
Delta_L
NG = 0
DB = 0
SM = 0
Cytarabine
YOR373W NUD1
ZShift = 3.02
Delta_K
NG = 0
DB = 0
SM = 0
Cytarabine
YOR373W NUD1
ZShift = 1.07
Delta_r
NG = 0
DB = 0
SM = 0
Cytarabine
YOR373W NUD1
ZShift = 1.59
Delta_AUC
NG = 0
DB = 0
SM = 0
Cytarabine
YLR458W
ZShift = 3.59
Delta_L
NG = 0
DB = 0
SM = 0
Cytarabine
YLR458W
ZShift = 0.67
Delta_K
NG = 0
DB = 0
SM = 0
Cytarabine
YLR458W
ZShift = −1.06
Delta_r
NG = 0
DB = 0
SM = 0
Cytarabine
YLR458W
ZShift = −1.14
Delta_AUC
NG = 0
DB = 0
SM = 0
Cytarabine

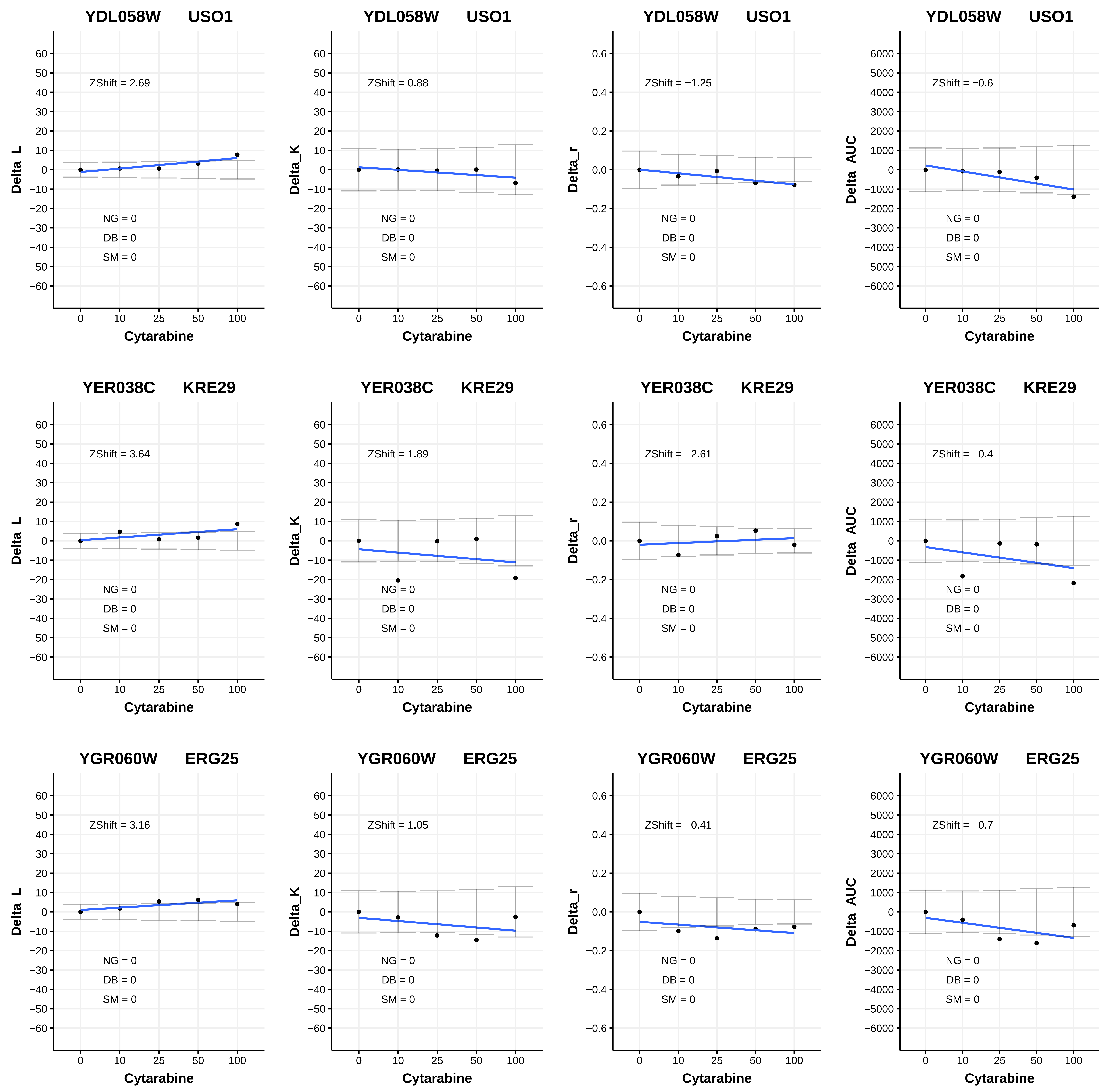
YDL058W USO1
ZShift = 2.69
NG = 0
DB = 0
SM = 0
Delta_L
Cytarabine
YDL058W USO1
ZShift = 0.88
NG = 0
DB = 0
SM = 0
Delta_K
Cytarabine
YDL058W USO1
ZShift = −1.25
NG = 0
DB = 0
SM = 0
Delta_r
Cytarabine
YDL058W USO1
ZShift = −0.6
NG = 0
DB = 0
SM = 0
Delta_AUC
Cytarabine
YER038C KRE29
ZShift = 3.64
NG = 0
DB = 0
SM = 0
Delta_L
Cytarabine
YER038C KRE29
ZShift = 1.89
NG = 0
DB = 0
SM = 0
Delta_K
Cytarabine
YER038C KRE29
ZShift = −2.61
NG = 0
DB = 0
SM = 0
Delta_r
Cytarabine
YER038C KRE29
ZShift = −0.4
NG = 0
DB = 0
SM = 0
Delta_AUC
Cytarabine
YGR060W ERG25
ZShift = 3.16
NG = 0
DB = 0
SM = 0
Delta_L
Cytarabine
YGR060W ERG25
ZShift = 1.05
NG = 0
DB = 0
SM = 0
Delta_K
Cytarabine
YGR060W ERG25
ZShift = −0.41
NG = 0
DB = 0
SM = 0
Delta_r
Cytarabine
YGR060W ERG25
ZShift = −0.7
NG = 0
DB = 0
SM = 0
Delta_AUC
Cytarabine

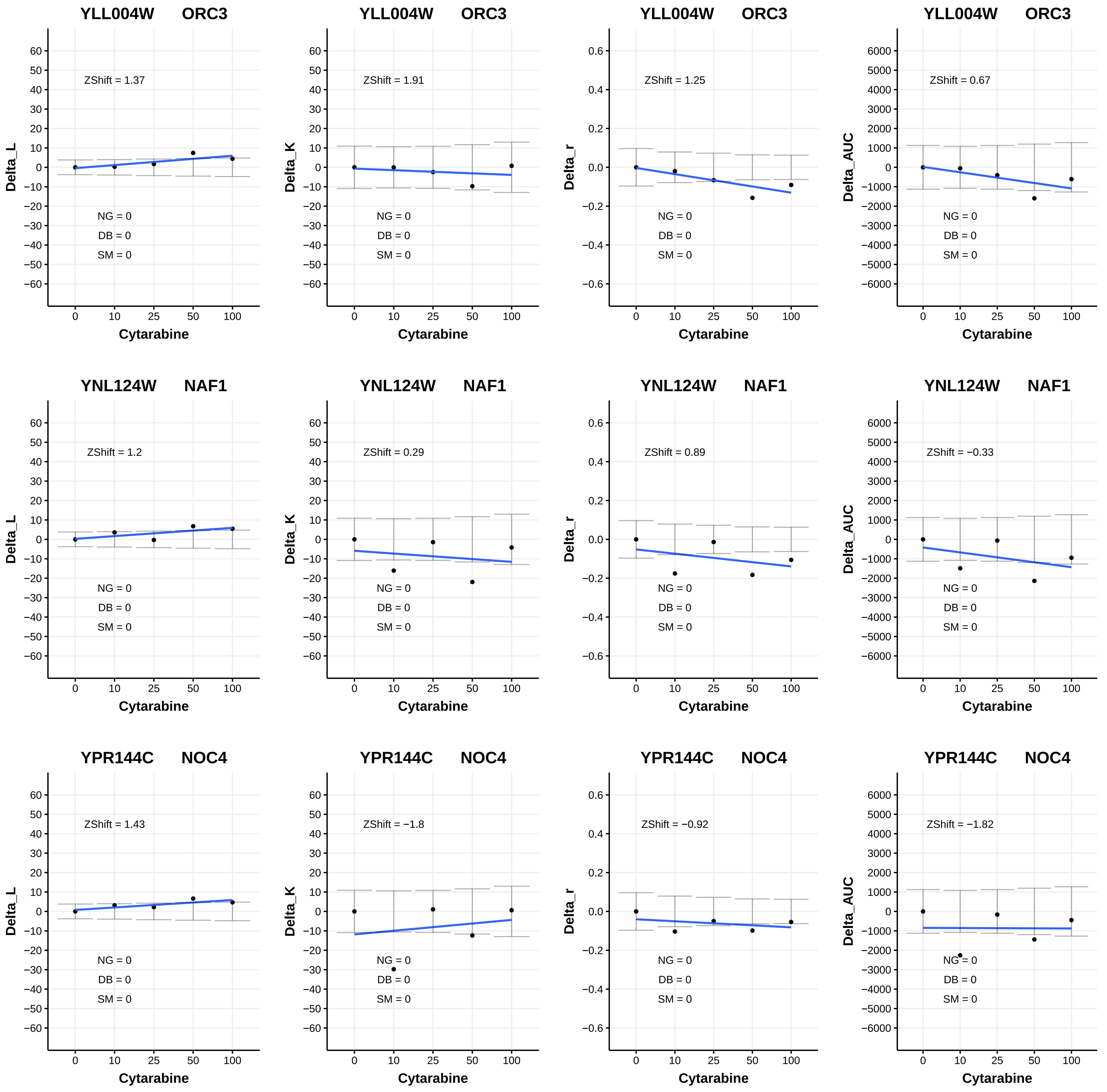
YLL004W ORC3
ZShift = 1.37
NG = 0
DB = 0
SM = 0
Delta_L
Cytarabine
YLL004W ORC3
ZShift = 1.91
NG = 0
DB = 0
SM = 0
Delta_K
Cytarabine
YLL004W ORC3
ZShift = 1.25
NG = 0
DB = 0
SM = 0
Delta_r
Cytarabine
YLL004W ORC3
ZShift = 0.67
NG = 0
DB = 0
SM = 0
Delta_AUC
Cytarabine
YNL124W NAF1
ZShift = 1.2
NG = 0
DB = 0
SM = 0
Delta_L
Cytarabine
YNL124W NAF1
ZShift = 0.29
NG = 0
DB = 0
SM = 0
Delta_K
Cytarabine
YNL124W NAF1
ZShift = 0.89
NG = 0
DB = 0
SM = 0
Delta_r
Cytarabine
YNL124W NAF1
ZShift = −0.33
NG = 0
DB = 0
SM = 0
Delta_AUC
Cytarabine
YPR144C NOC4
ZShift = 1.43
NG = 0
DB = 0
SM = 0
Delta_L
Cytarabine
YPR144C NOC4
ZShift = −1.8
NG = 0
DB = 0
SM = 0
Delta_K
Cytarabine
YPR144C NOC4
ZShift = −0.92
NG = 0
DB = 0
SM = 0
Delta_r
Cytarabine
YPR144C NOC4
ZShift = −1.82
NG = 0
DB = 0
SM = 0
Delta_AUC
Cytarabine

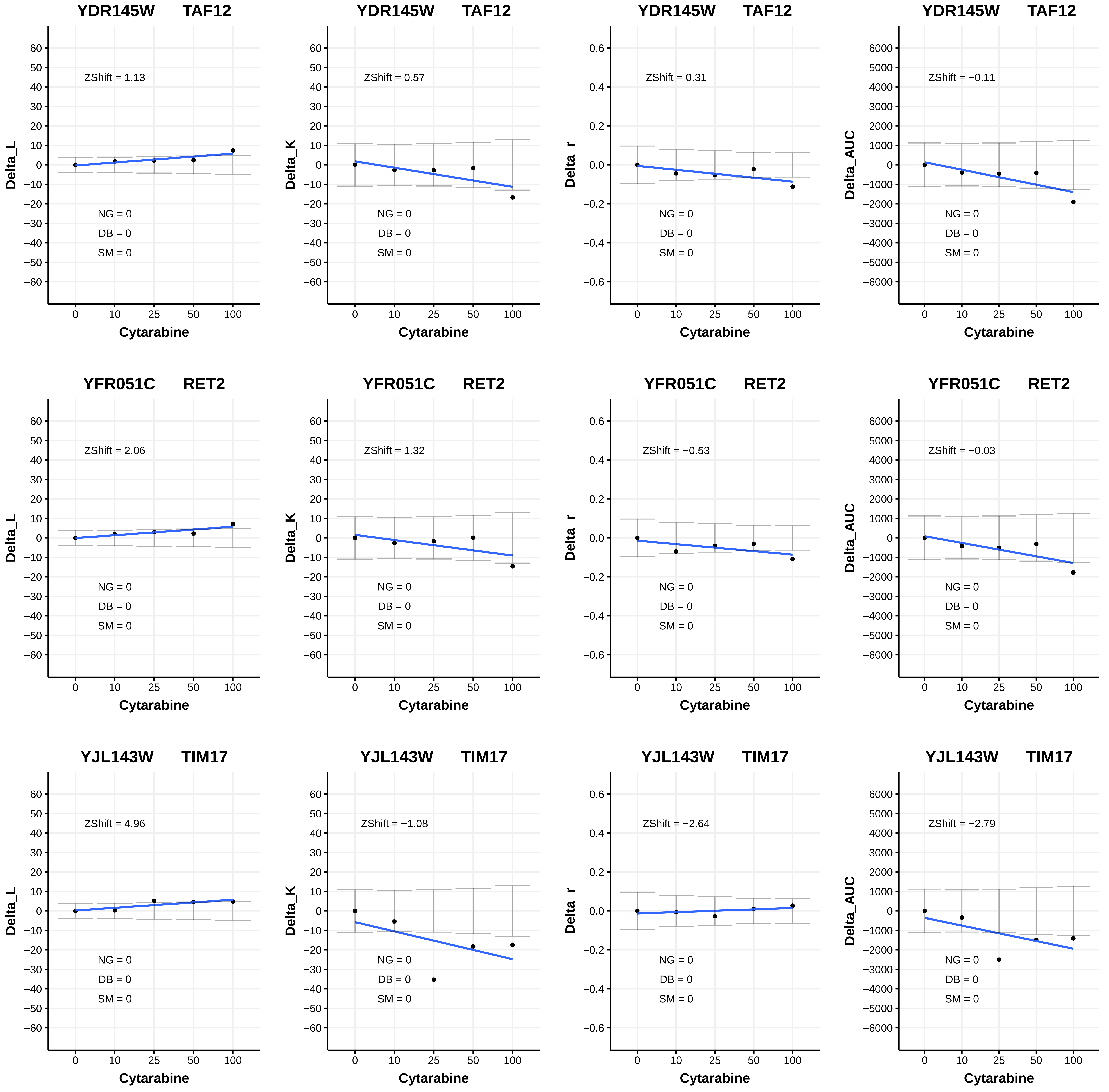

YDR145W TAF12
ZShift = 1.13
ZShift = 0.57
ZShift = 0.31
ZShift = −0.11
YFR051C RET2
ZShift = 2.06
ZShift = 1.32
ZShift = −0.53
ZShift = −0.03
YJL143W TIM17
ZShift = 4.96
ZShift = −1.08
ZShift = −2.64
ZShift = −2.79
NG = 0
DB = 0
SM = 0
Delta_L
Delta_K
Delta_r
Delta_AUC
Cytarabine
0
10
25
50
100

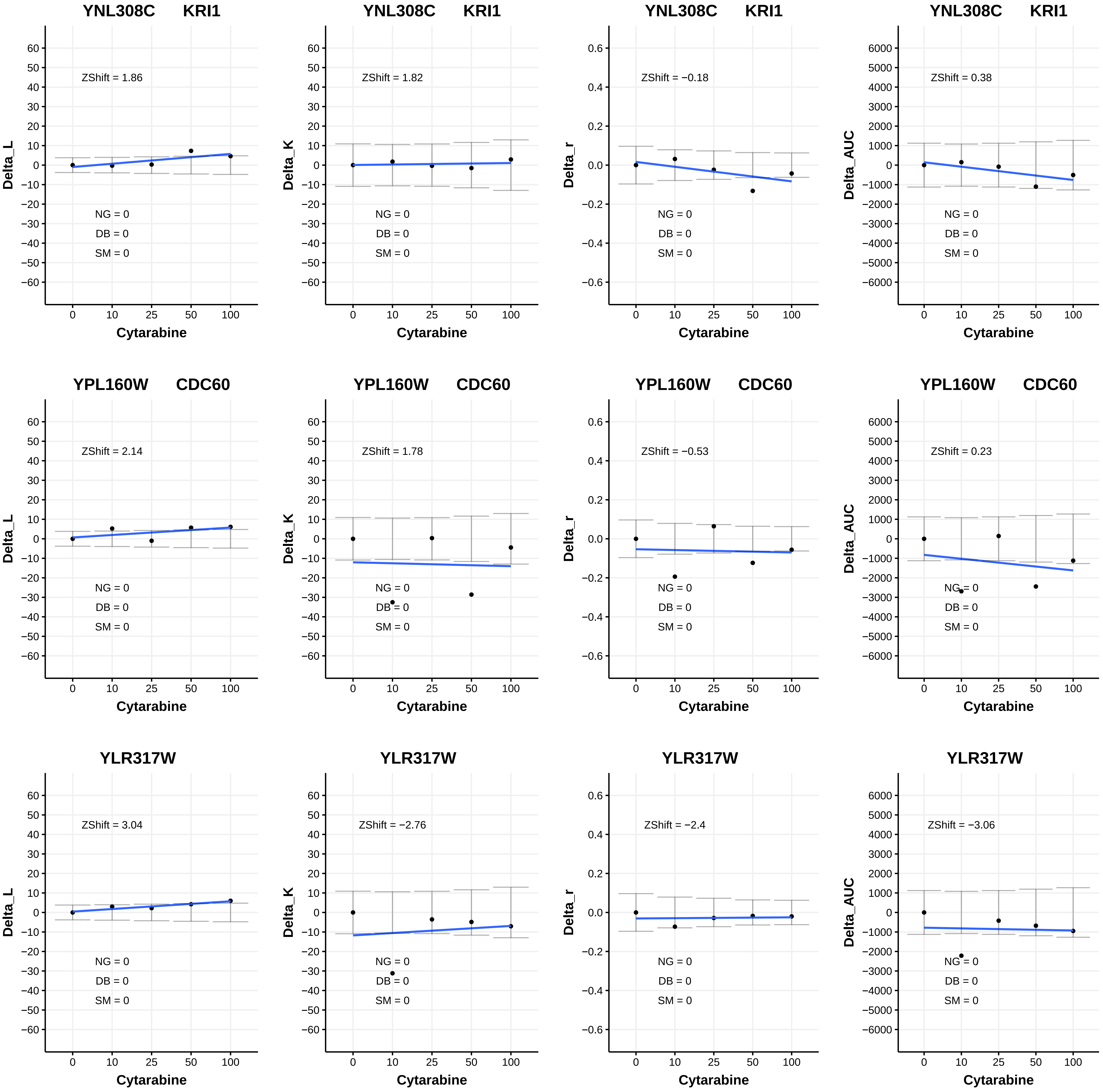

YNL308C KRI1
ZShift = 1.86
ZShift = 1.82
ZShift = −0.18
ZShift = 0.38
YPL160W CDC60
ZShift = 2.14
ZShift = 1.78
ZShift = −0.53
ZShift = 0.23
YLR317W
ZShift = 3.04
ZShift = −2.76
ZShift = −2.4
ZShift = −3.06
NG = 0
DB = 0
SM = 0
Delta_L
Delta_K
Delta_r
Delta_AUC
Cytarabine
0
10
25
50
100

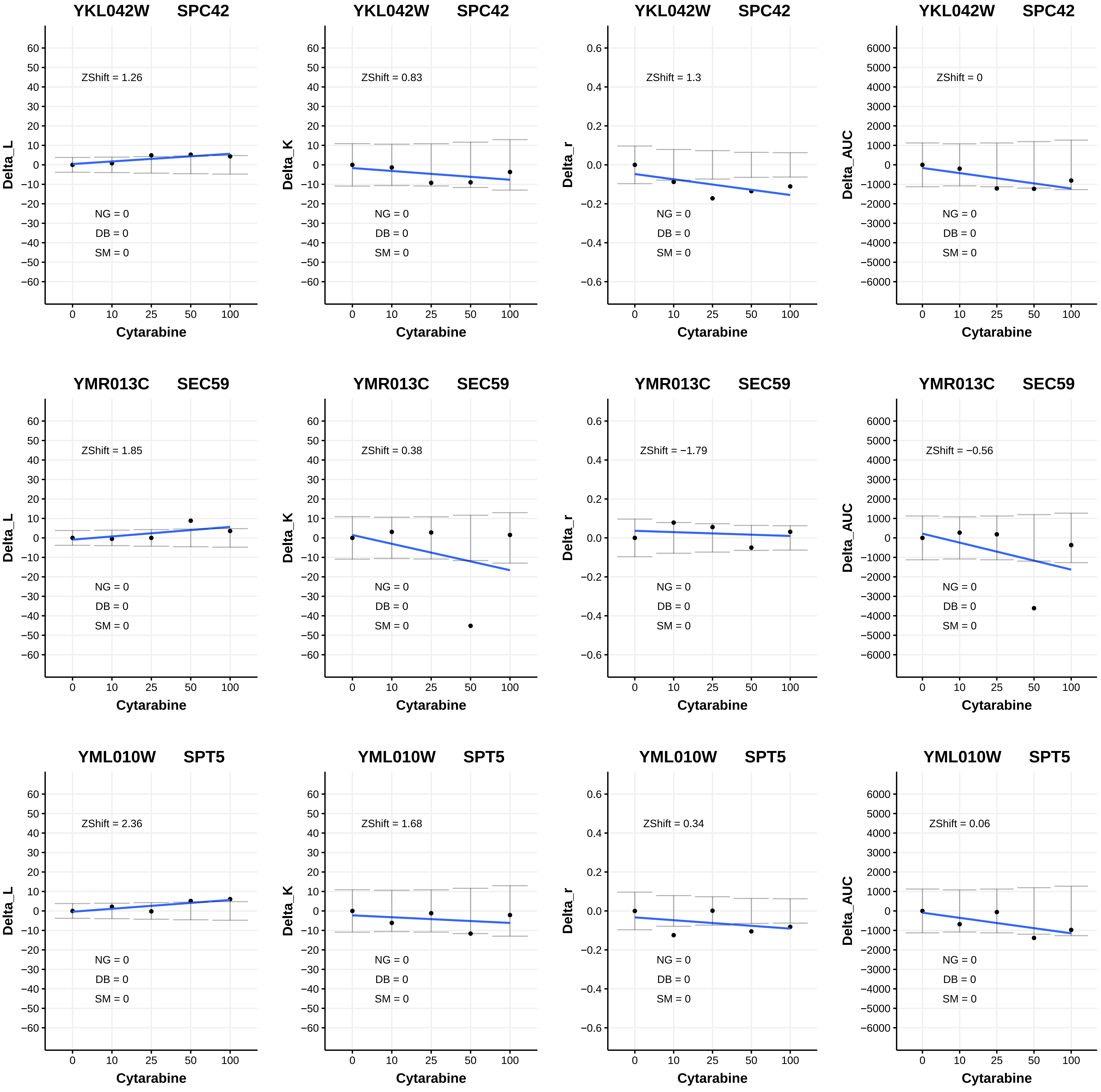
YKL042W SPC42
ZShift = 1.26
NG = 0
DB = 0
SM = 0
Delta_L
Cytarabine
YKL042W SPC42
ZShift = 0.83
NG = 0
DB = 0
SM = 0
Delta_K
Cytarabine
YKL042W SPC42
ZShift = 1.3
NG = 0
DB = 0
SM = 0
Delta_r
Cytarabine
YKL042W SPC42
ZShift = 0
NG = 0
DB = 0
SM = 0
Delta_AUC
Cytarabine
YMR013C SEC59
ZShift = 1.85
NG = 0
DB = 0
SM = 0
Delta_L
Cytarabine
YMR013C SEC59
ZShift = 0.38
NG = 0
DB = 0
SM = 0
Delta_K
Cytarabine
YMR013C SEC59
ZShift = −1.79
NG = 0
DB = 0
SM = 0
Delta_r
Cytarabine
YMR013C SEC59
ZShift = −0.56
NG = 0
DB = 0
SM = 0
Delta_AUC
Cytarabine
YML010W SPT5
ZShift = 2.36
NG = 0
DB = 0
SM = 0
Delta_L
Cytarabine
YML010W SPT5
ZShift = 1.68
NG = 0
DB = 0
SM = 0
Delta_K
Cytarabine
YML010W SPT5
ZShift = 0.34
NG = 0
DB = 0
SM = 0
Delta_r
Cytarabine
YML010W SPT5
ZShift = 0.06
NG = 0
DB = 0
SM = 0
Delta_AUC
Cytarabine

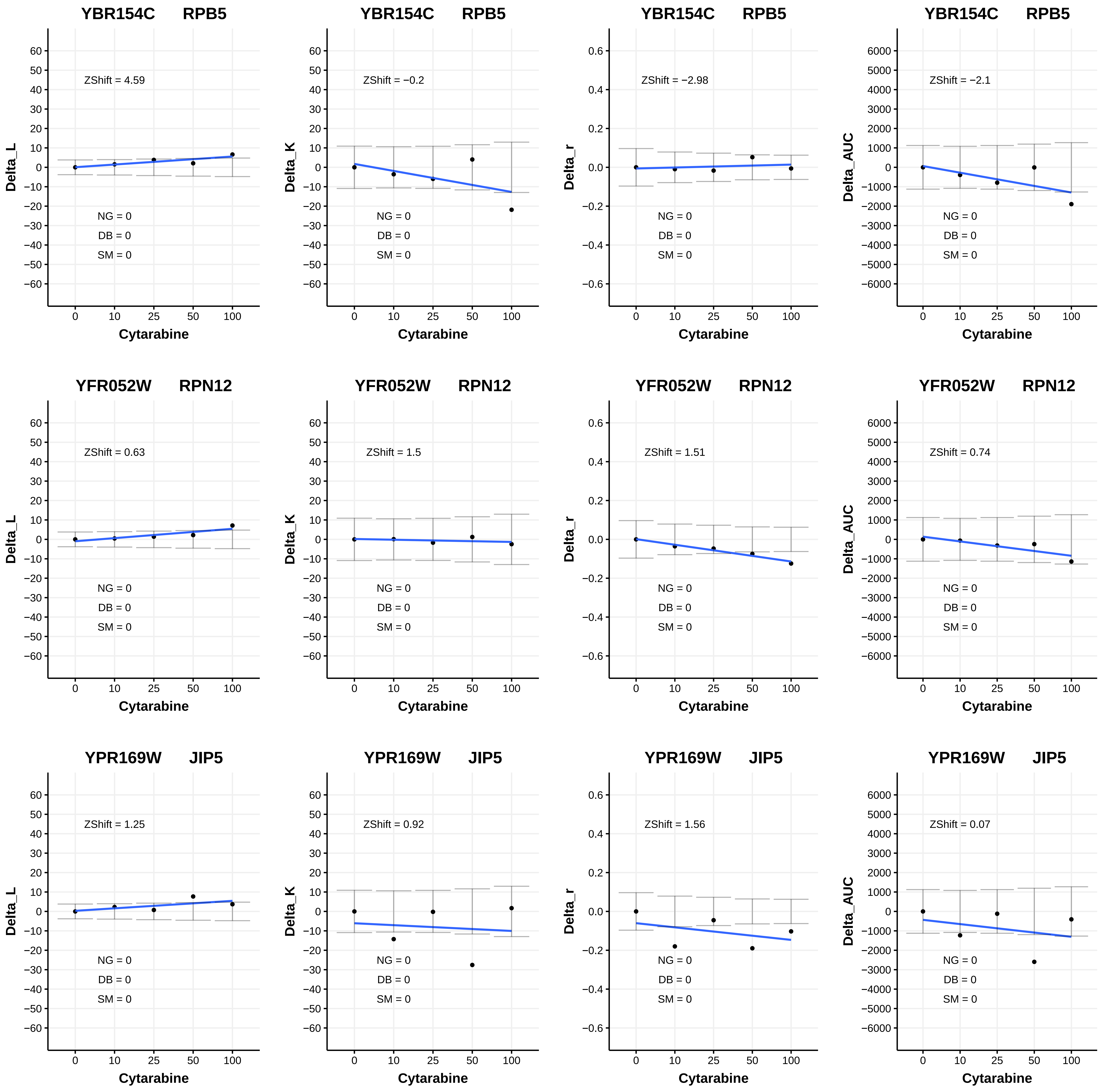

YBR154C RPB5
ZShift = 4.59
Delta_L
NG = 0
DB = 0
SM = 0
Cytarabine
YBR154C RPB5
ZShift = −0.2
Delta_K
NG = 0
DB = 0
SM = 0
Cytarabine
YBR154C RPB5
ZShift = −2.98
Delta_r
NG = 0
DB = 0
SM = 0
Cytarabine
YBR154C RPB5
ZShift = −2.1
Delta_AUC
NG = 0
DB = 0
SM = 0
Cytarabine
YFR052W RPN12
ZShift = 0.63
Delta_L
NG = 0
DB = 0
SM = 0
Cytarabine
YFR052W RPN12
ZShift = 1.5
Delta_K
NG = 0
DB = 0
SM = 0
Cytarabine
YFR052W RPN12
ZShift = 1.51
Delta_r
NG = 0
DB = 0
SM = 0
Cytarabine
YFR052W RPN12
ZShift = 0.74
Delta_AUC
NG = 0
DB = 0
SM = 0
Cytarabine
YPR169W JIP5
ZShift = 1.25
Delta_L
NG = 0
DB = 0
SM = 0
Cytarabine
YPR169W JIP5
ZShift = 0.92
Delta_K
NG = 0
DB = 0
SM = 0
Cytarabine
YPR169W JIP5
ZShift = 1.56
Delta_r
NG = 0
DB = 0
SM = 0
Cytarabine
YPR169W JIP5
ZShift = 0.07
Delta_AUC
NG = 0
DB = 0
SM = 0
Cytarabine

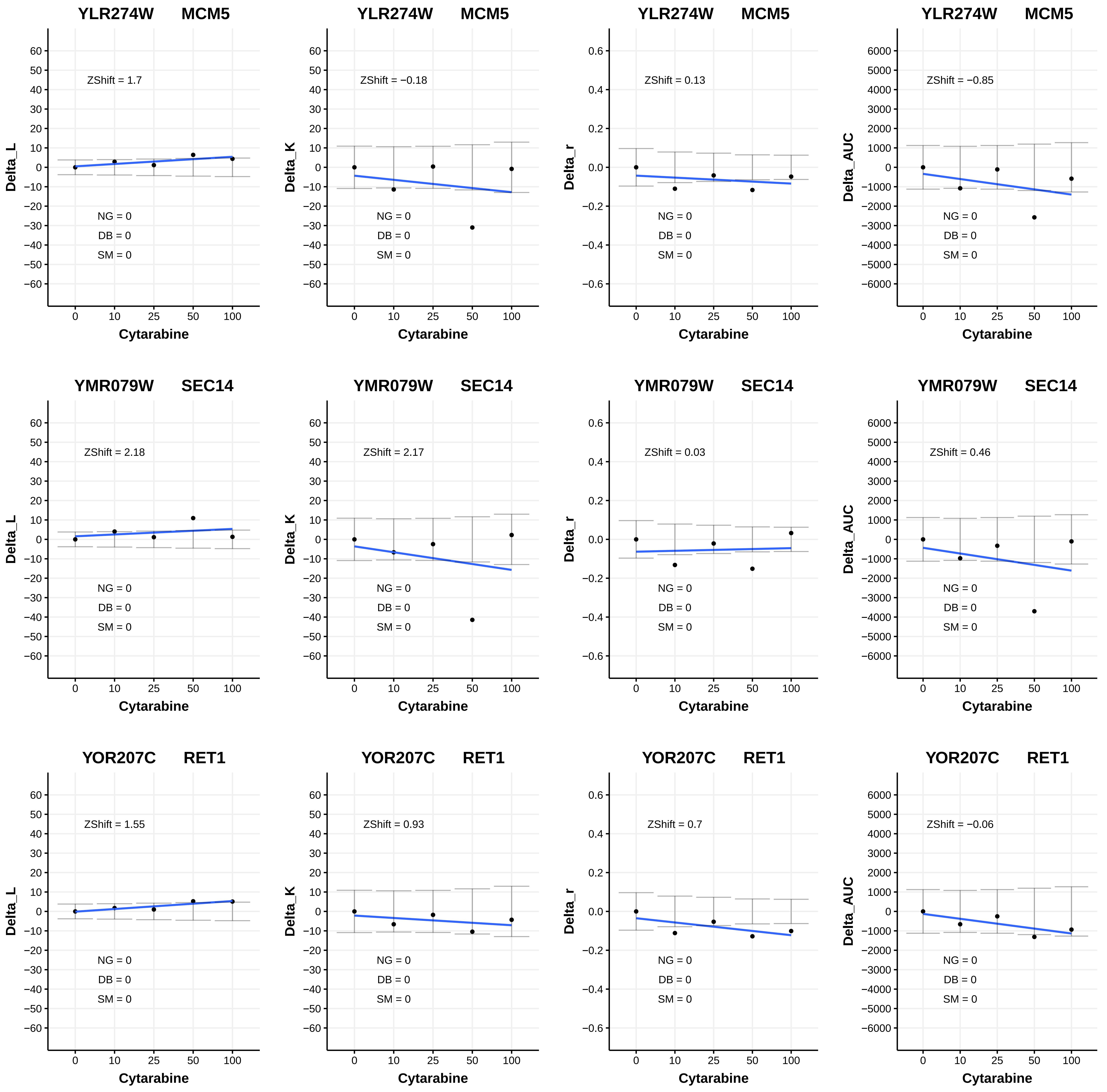
YLR274W MCM5
ZShift = 1.7
NG = 0
DB = 0
SM = 0
Delta_L
Cytarabine
YLR274W MCM5
ZShift = −0.18
NG = 0
DB = 0
SM = 0
Delta_K
Cytarabine
YLR274W MCM5
ZShift = 0.13
NG = 0
DB = 0
SM = 0
Delta_r
Cytarabine
YLR274W MCM5
ZShift = −0.85
NG = 0
DB = 0
SM = 0
Delta_AUC
Cytarabine
YMR079W SEC14
ZShift = 2.18
NG = 0
DB = 0
SM = 0
Delta_L
Cytarabine
YMR079W SEC14
ZShift = 2.17
NG = 0
DB = 0
SM = 0
Delta_K
Cytarabine
YMR079W SEC14
ZShift = 0.03
NG = 0
DB = 0
SM = 0
Delta_r
Cytarabine
YMR079W SEC14
ZShift = 0.46
NG = 0
DB = 0
SM = 0
Delta_AUC
Cytarabine
YOR207C RET1
ZShift = 1.55
NG = 0
DB = 0
SM = 0
Delta_L
Cytarabine
YOR207C RET1
ZShift = 0.93
NG = 0
DB = 0
SM = 0
Delta_K
Cytarabine
YOR207C RET1
ZShift = 0.7
NG = 0
DB = 0
SM = 0
Delta_r
Cytarabine
YOR207C RET1
ZShift = −0.06
NG = 0
DB = 0
SM = 0
Delta_AUC
Cytarabine

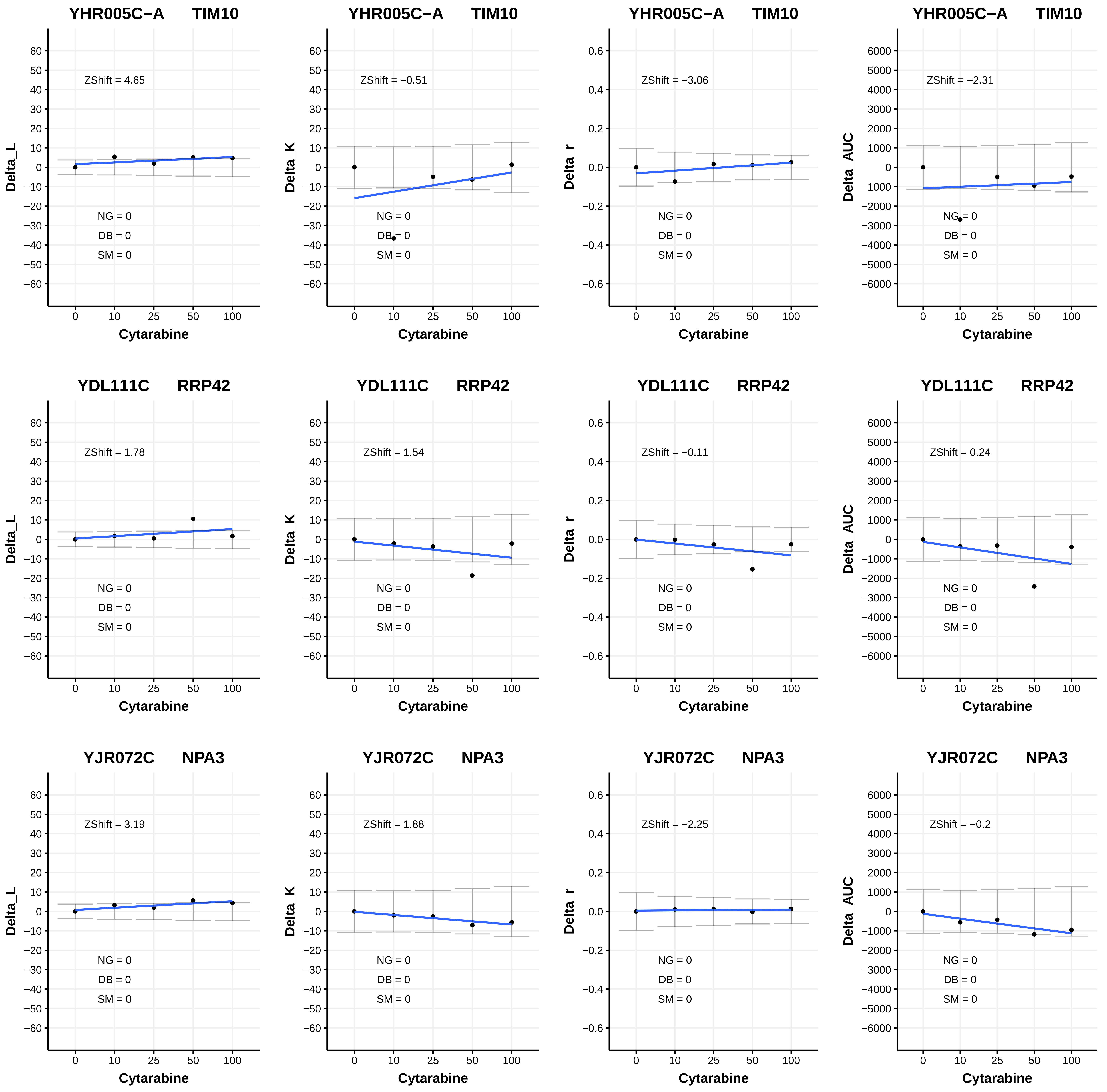
YHR005C−A TIM10
ZShift = 4.65
ZShift = −0.51
ZShift = −3.06
ZShift = −2.31
YDL111C RRP42
ZShift = 1.78
ZShift = 1.54
ZShift = −0.11
ZShift = 0.24
YJR072C NPA3
ZShift = 3.19
ZShift = 1.88
ZShift = −2.25
ZShift = −0.2
NG = 0
DB = 0
SM = 0
Delta_L
Delta_K
Delta_r
Delta_AUC
Cytarabine
0 10 25 50 100

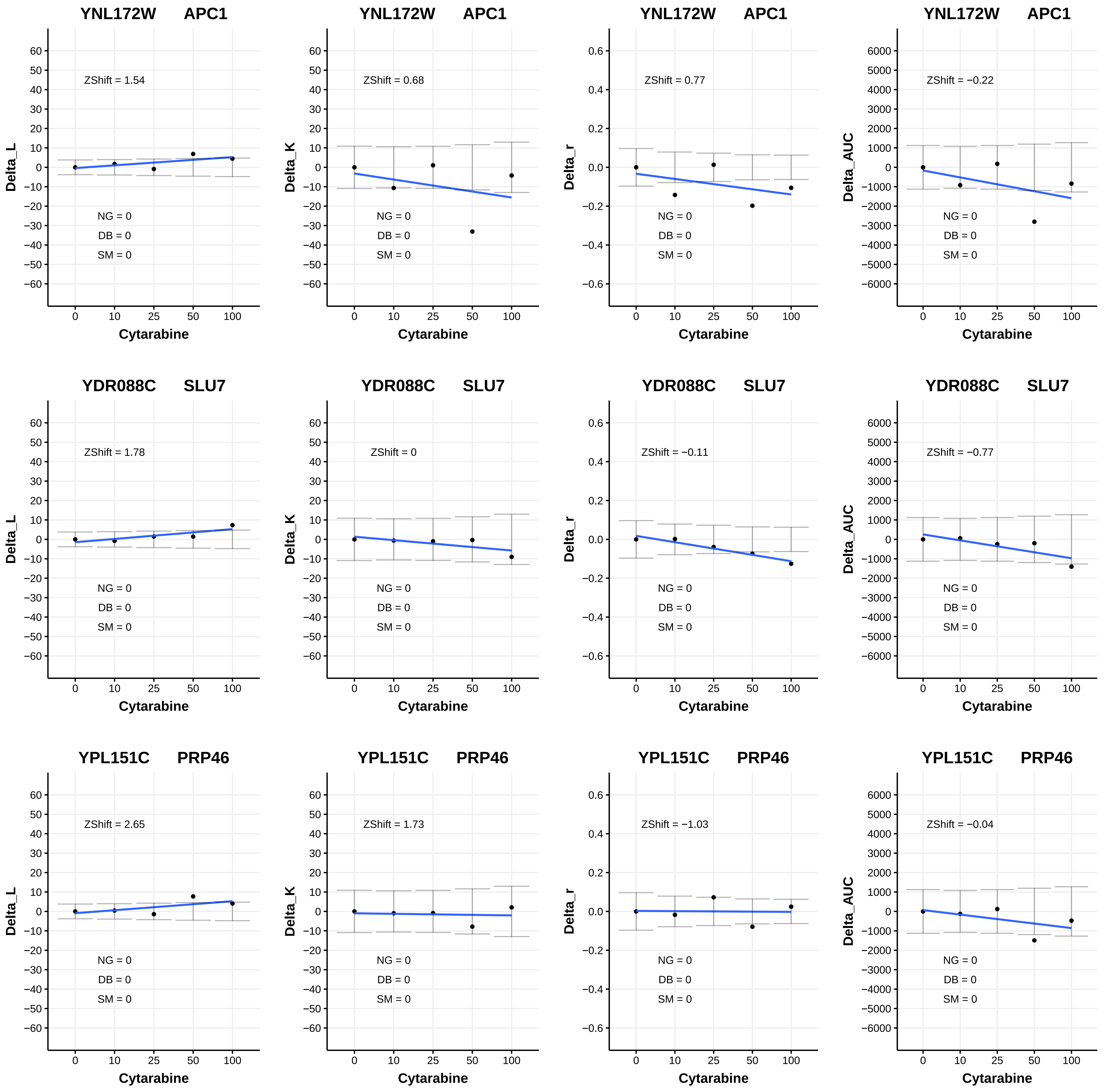
YNL172W APC1
ZShift = 1.54
NG = 0
DB = 0
SM = 0
Delta_L
Cytarabine
YNL172W APC1
ZShift = 0.68
NG = 0
DB = 0
SM = 0
Delta_K
Cytarabine
YNL172W APC1
ZShift = 0.77
NG = 0
DB = 0
SM = 0
Delta_r
Cytarabine
YNL172W APC1
ZShift = −0.22
NG = 0
DB = 0
SM = 0
Delta_AUC
Cytarabine
YDR088C SLU7
ZShift = 1.78
NG = 0
DB = 0
SM = 0
Delta_L
Cytarabine
YDR088C SLU7
ZShift = 0
NG = 0
DB = 0
SM = 0
Delta_K
Cytarabine
YDR088C SLU7
ZShift = −0.11
NG = 0
DB = 0
SM = 0
Delta_r
Cytarabine
YDR088C SLU7
ZShift = −0.77
NG = 0
DB = 0
SM = 0
Delta_AUC
Cytarabine
YPL151C PRP46
ZShift = 2.65
NG = 0
DB = 0
SM = 0
Delta_L
Cytarabine
YPL151C PRP46
ZShift = 1.73
NG = 0
DB = 0
SM = 0
Delta_K
Cytarabine
YPL151C PRP46
ZShift = −1.03
NG = 0
DB = 0
SM = 0
Delta_r
Cytarabine
YPL151C PRP46
ZShift = −0.04
NG = 0
DB = 0
SM = 0
Delta_AUC
Cytarabine

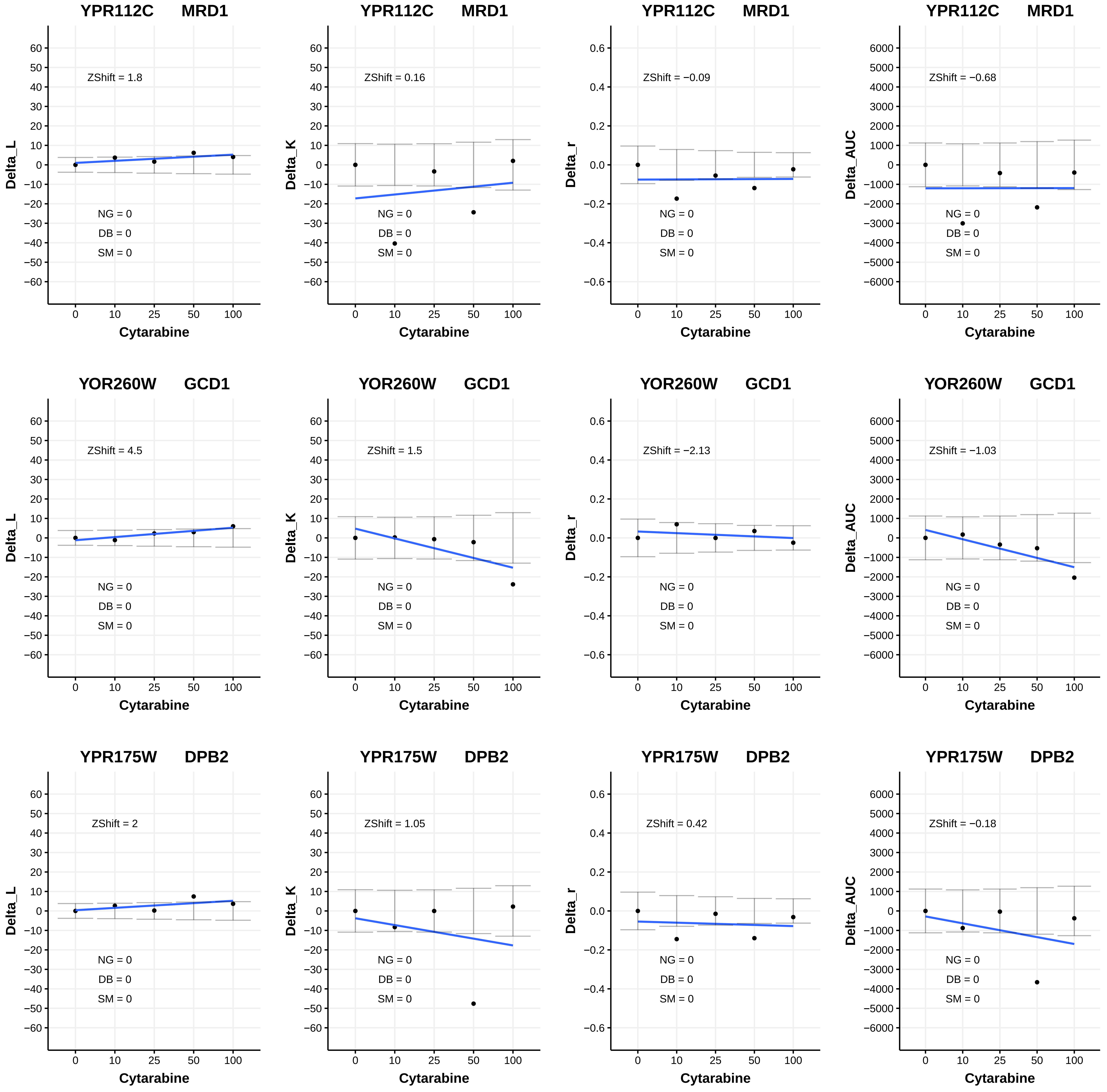
YPR112C MRD1
ZShift = 1.8
ZShift = 0.16
ZShift = −0.09
ZShift = −0.68
YOR260W GCD1
ZShift = 4.5
ZShift = 1.5
ZShift = −2.13
ZShift = −1.03
YPR175W DPB2
ZShift = 2
ZShift = 1.05
ZShift = 0.42
ZShift = −0.18
NG = 0
DB = 0
SM = 0
Delta_L
Delta_K
Delta_r
Delta_AUC
Cytarabine
0
10
25
50
100

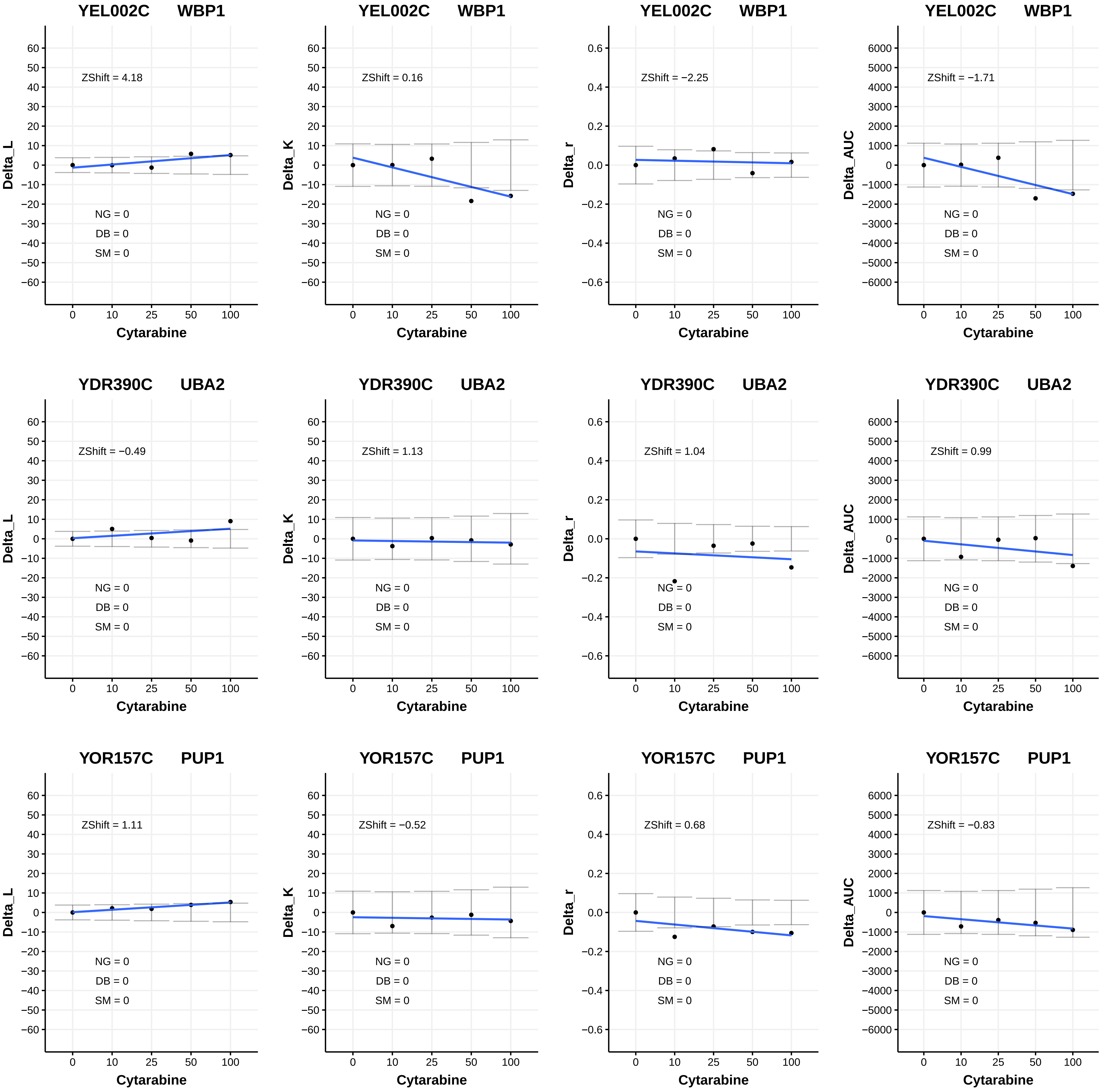
YEL002C WBP1
ZShift = 4.18
ZShift = 0.16
ZShift = −2.25
ZShift = −1.71
YDR390C UBA2
ZShift = −0.49
ZShift = 1.13
ZShift = 1.04
ZShift = 0.99
YOR157C PUP1
ZShift = 1.11
ZShift = −0.52
ZShift = 0.68
ZShift = −0.83
NG = 0
DB = 0
SM = 0
Delta_L
Delta_K
Delta_r
Delta_AUC
Cytarabine
0
10
25
50
100

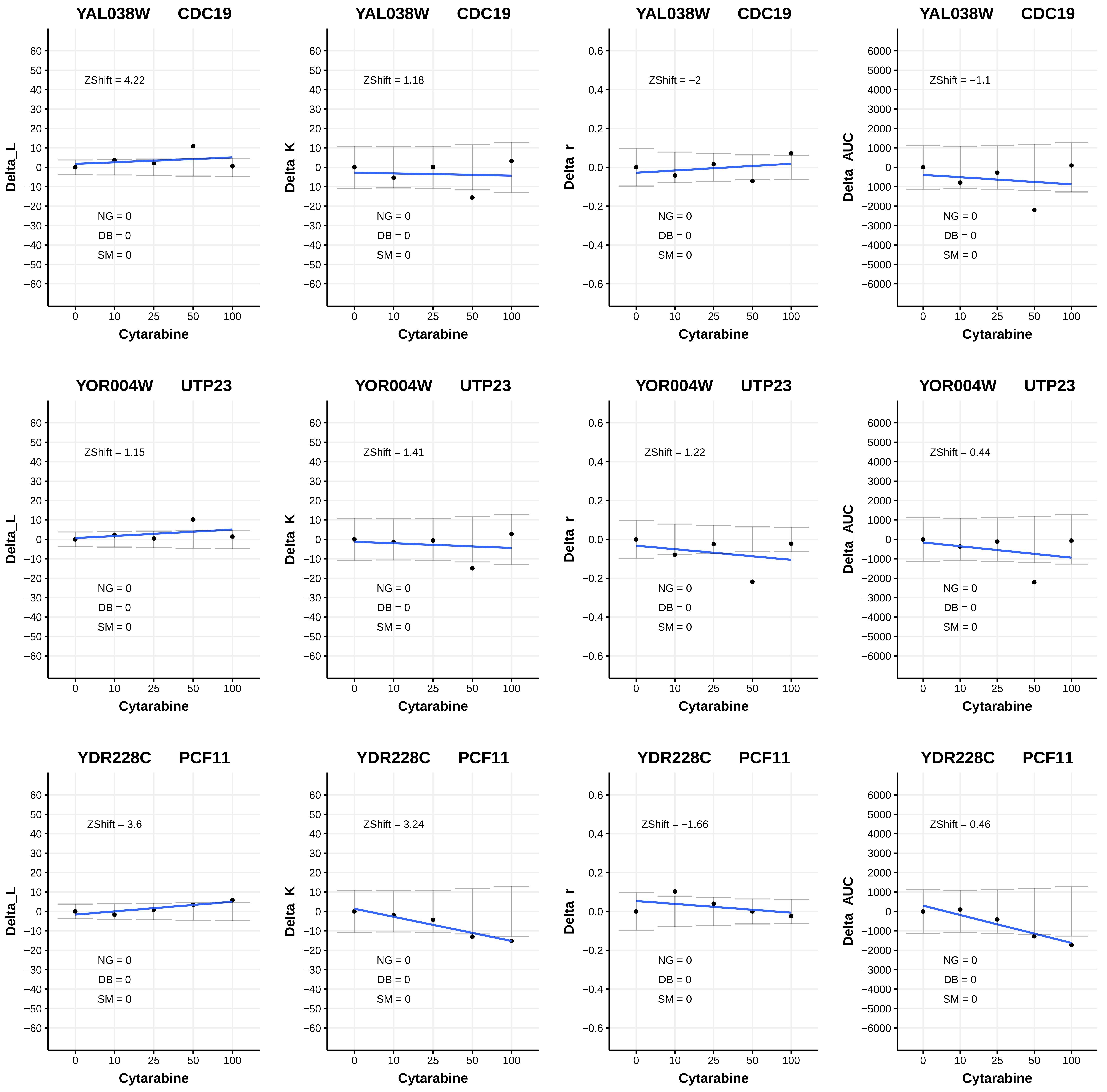
YAL038W CDC19
Delta_L
ZShift = 4.22
NG = 0
DB = 0
SM = 0
Cytarabine
YAL038W CDC19
Delta_K
ZShift = 1.18
NG = 0
DB = 0
SM = 0
Cytarabine
YAL038W CDC19
Delta_r
ZShift = −2
NG = 0
DB = 0
SM = 0
Cytarabine
YAL038W CDC19
Delta_AUC
ZShift = −1.1
NG = 0
DB = 0
SM = 0
Cytarabine
YOR004W UTP23
Delta_L
ZShift = 1.15
NG = 0
DB = 0
SM = 0
Cytarabine
YOR004W UTP23
Delta_K
ZShift = 1.41
NG = 0
DB = 0
SM = 0
Cytarabine
YOR004W UTP23
Delta_r
ZShift = 1.22
NG = 0
DB = 0
SM = 0
Cytarabine
YOR004W UTP23
Delta_AUC
ZShift = 0.44
NG = 0
DB = 0
SM = 0
Cytarabine
YDR228C PCF11
Delta_L
ZShift = 3.6
NG = 0
DB = 0
SM = 0
Cytarabine
YDR228C PCF11
Delta_K
ZShift = 3.24
NG = 0
DB = 0
SM = 0
Cytarabine
YDR228C PCF11
Delta_r
ZShift = −1.66
NG = 0
DB = 0
SM = 0
Cytarabine
YDR228C PCF11
Delta_AUC
ZShift = 0.46
NG = 0
DB = 0
SM = 0
Cytarabine

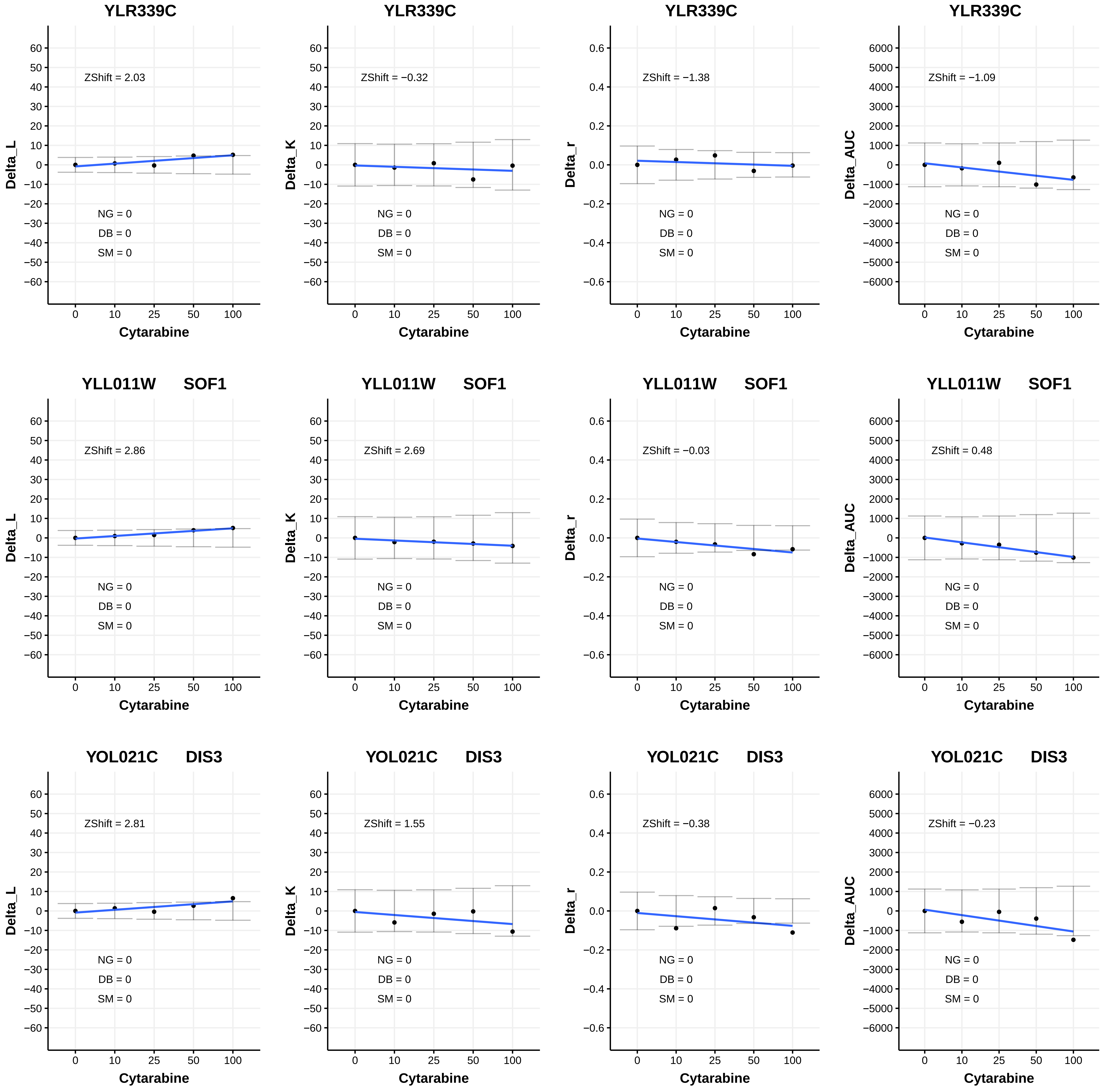

YLR339C
ZShift = 2.03
Delta_L
NG = 0
DB = 0
SM = 0
Cytarabine
YLR339C
ZShift = −0.32
Delta_K
NG = 0
DB = 0
SM = 0
Cytarabine
YLR339C
ZShift = −1.38
Delta_r
NG = 0
DB = 0
SM = 0
Cytarabine
YLR339C
ZShift = −1.09
Delta_AUC
NG = 0
DB = 0
SM = 0
Cytarabine
YLL011W SOF1
ZShift = 2.86
Delta_L
NG = 0
DB = 0
SM = 0
Cytarabine
YLL011W SOF1
ZShift = 2.69
Delta_K
NG = 0
DB = 0
SM = 0
Cytarabine
YLL011W SOF1
ZShift = −0.03
Delta_r
NG = 0
DB = 0
SM = 0
Cytarabine
YLL011W SOF1
ZShift = 0.48
Delta_AUC
NG = 0
DB = 0
SM = 0
Cytarabine
YOL021C DIS3
ZShift = 2.81
Delta_L
NG = 0
DB = 0
SM = 0
Cytarabine
YOL021C DIS3
ZShift = 1.55
Delta_K
NG = 0
DB = 0
SM = 0
Cytarabine
YOL021C DIS3
ZShift = −0.38
Delta_r
NG = 0
DB = 0
SM = 0
Cytarabine
YOL021C DIS3
ZShift = −0.23
Delta_AUC
NG = 0
DB = 0
SM = 0
Cytarabine

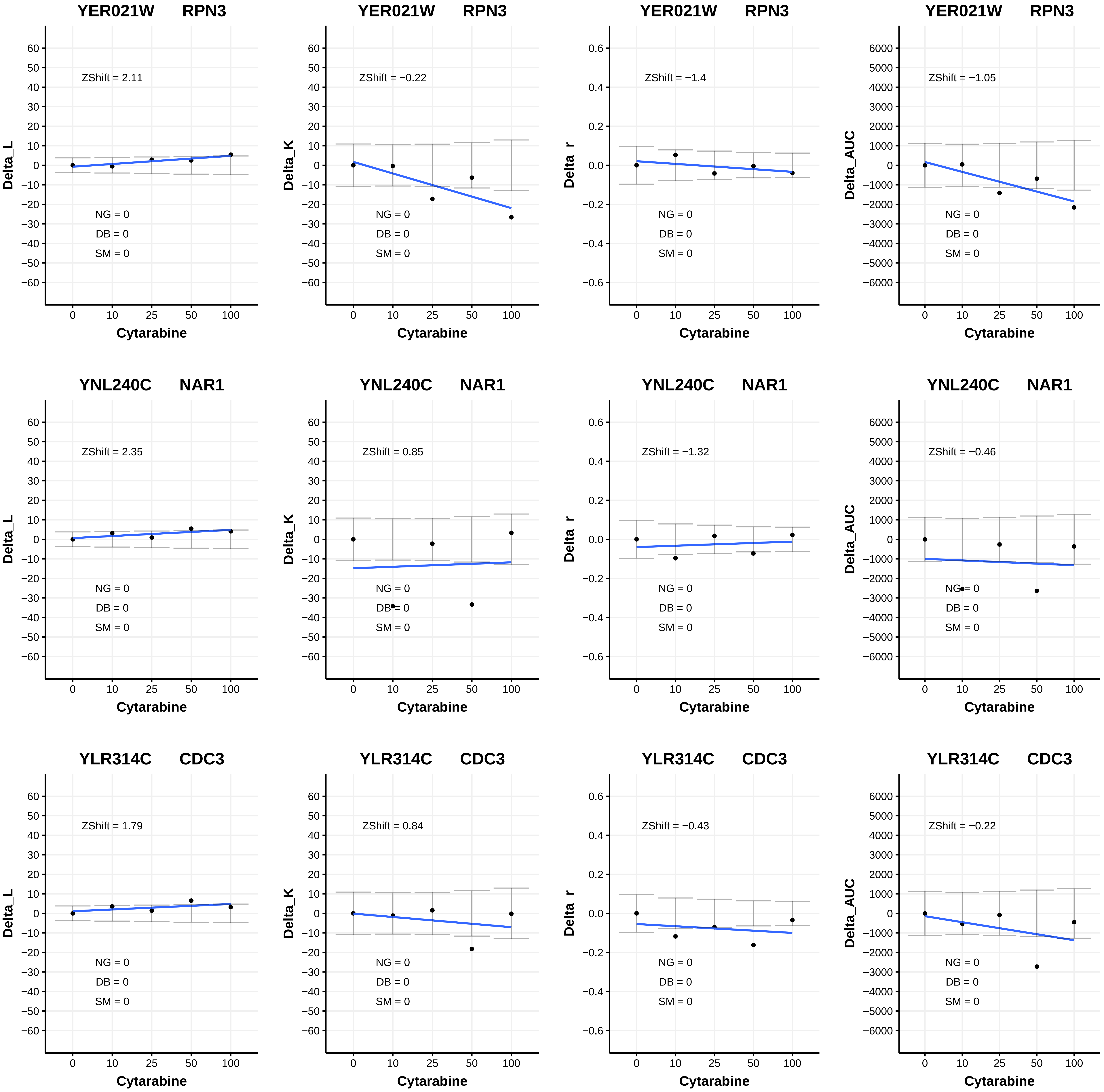
YER021W RPN3
ZShift = 2.11
ZShift = −0.22
ZShift = −1.4
ZShift = −1.05
YNL240C NAR1
ZShift = 2.35
ZShift = 0.85
ZShift = −1.32
ZShift = −0.46
YLR314C CDC3
ZShift = 1.79
ZShift = 0.84
ZShift = −0.43
ZShift = −0.22
NG = 0
DB = 0
SM = 0
Delta_L
Delta_K
Delta_r
Delta_AUC
Cytarabine
0
10
25
50
100

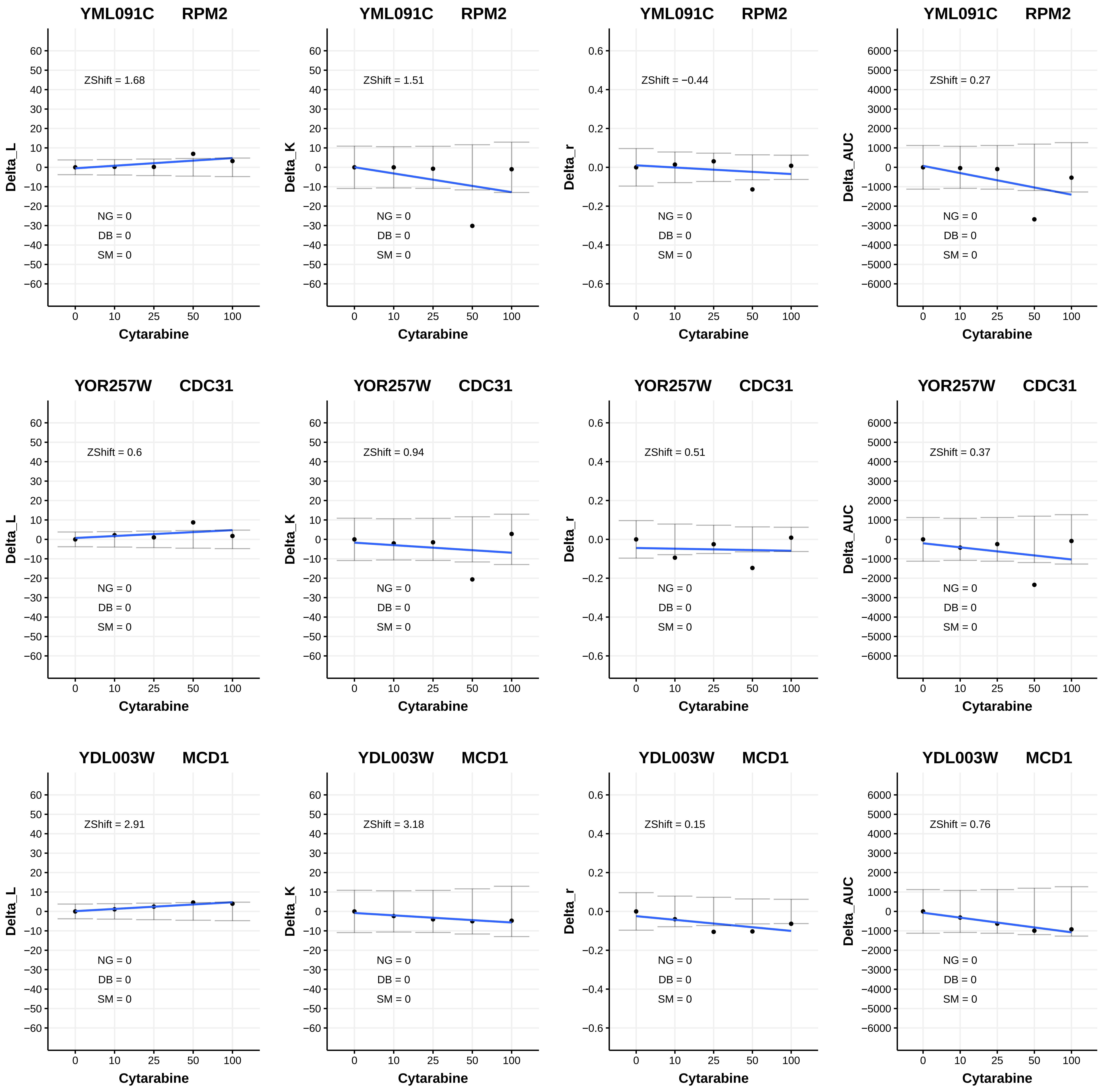
YML091C RPM2
ZShift = 1.68
NG = 0
DB = 0
SM = 0
Delta_L
Cytarabine
YML091C RPM2
ZShift = 1.51
NG = 0
DB = 0
SM = 0
Delta_K
Cytarabine
YML091C RPM2
ZShift = −0.44
NG = 0
DB = 0
SM = 0
Delta_r
Cytarabine
YML091C RPM2
ZShift = 0.27
NG = 0
DB = 0
SM = 0
Delta_AUC
Cytarabine
YOR257W CDC31
ZShift = 0.6
NG = 0
DB = 0
SM = 0
Delta_L
Cytarabine
YOR257W CDC31
ZShift = 0.94
NG = 0
DB = 0
SM = 0
Delta_K
Cytarabine
YOR257W CDC31
ZShift = 0.51
NG = 0
DB = 0
SM = 0
Delta_r
Cytarabine
YOR257W CDC31
ZShift = 0.37
NG = 0
DB = 0
SM = 0
Delta_AUC
Cytarabine
YDL003W MCD1
ZShift = 2.91
NG = 0
DB = 0
SM = 0
Delta_L
Cytarabine
YDL003W MCD1
ZShift = 3.18
NG = 0
DB = 0
SM = 0
Delta_K
Cytarabine
YDL003W MCD1
ZShift = 0.15
NG = 0
DB = 0
SM = 0
Delta_r
Cytarabine
YDL003W MCD1
ZShift = 0.76
NG = 0
DB = 0
SM = 0
Delta_AUC
Cytarabine

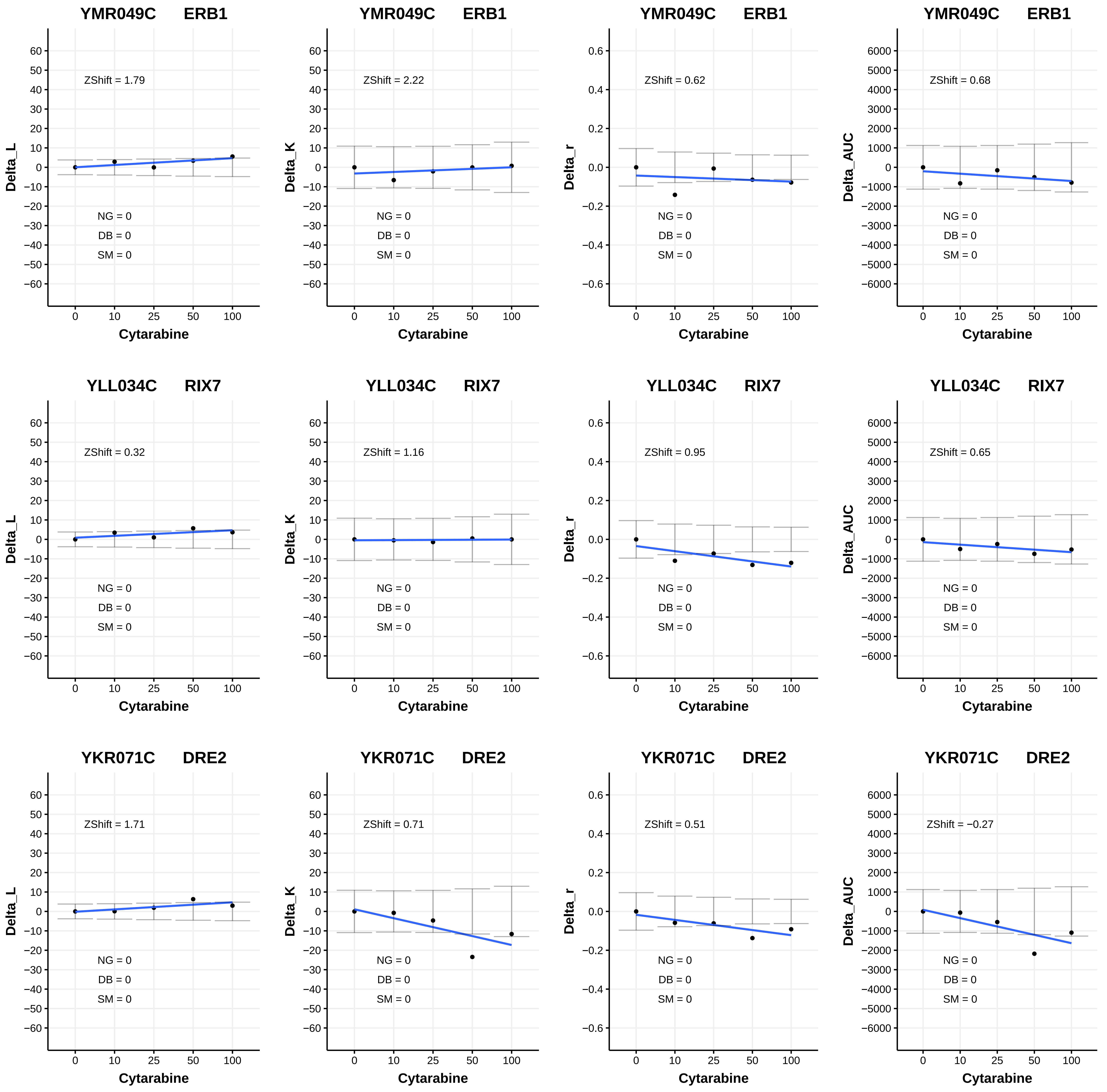
YMR049C ERB1
ZShift = 1.79
NG = 0
DB = 0
SM = 0
Delta_L
Cytarabine
YMR049C ERB1
ZShift = 2.22
NG = 0
DB = 0
SM = 0
Delta_K
Cytarabine
YMR049C ERB1
ZShift = 0.62
NG = 0
DB = 0
SM = 0
Delta_r
Cytarabine
YMR049C ERB1
ZShift = 0.68
NG = 0
DB = 0
SM = 0
Delta_AUC
Cytarabine
YLL034C RIX7
ZShift = 0.32
NG = 0
DB = 0
SM = 0
Delta_L
Cytarabine
YLL034C RIX7
ZShift = 1.16
NG = 0
DB = 0
SM = 0
Delta_K
Cytarabine
YLL034C RIX7
ZShift = 0.95
NG = 0
DB = 0
SM = 0
Delta_r
Cytarabine
YLL034C RIX7
ZShift = 0.65
NG = 0
DB = 0
SM = 0
Delta_AUC
Cytarabine
YKR071C DRE2
ZShift = 1.71
NG = 0
DB = 0
SM = 0
Delta_L
Cytarabine
YKR071C DRE2
ZShift = 0.71
NG = 0
DB = 0
SM = 0
Delta_K
Cytarabine
YKR071C DRE2
ZShift = 0.51
NG = 0
DB = 0
SM = 0
Delta_r
Cytarabine
YKR071C DRE2
ZShift = −0.27
NG = 0
DB = 0
SM = 0
Delta_AUC
Cytarabine

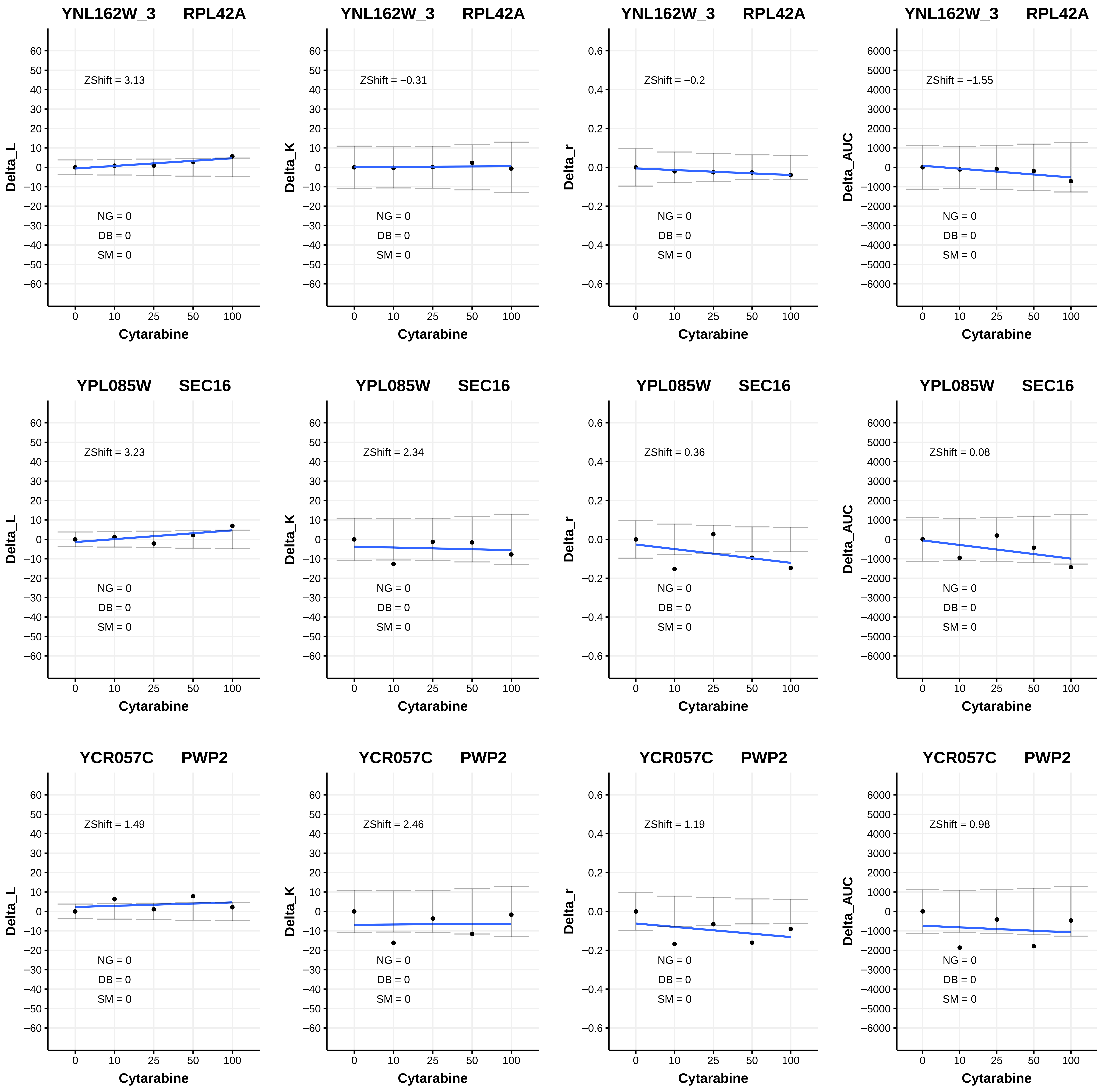

YNL162W_3 RPL42A
ZShift = 3.13
NG = 0
DB = 0
SM = 0
Delta_L
Cytarabine
YNL162W_3 RPL42A
ZShift = −0.31
NG = 0
DB = 0
SM = 0
Delta_K
Cytarabine
YNL162W_3 RPL42A
ZShift = −0.2
NG = 0
DB = 0
SM = 0
Delta_r
Cytarabine
YNL162W_3 RPL42A
ZShift = −1.55
NG = 0
DB = 0
SM = 0
Delta_AUC
Cytarabine
YPL085W SEC16
ZShift = 3.23
NG = 0
DB = 0
SM = 0
Delta_L
Cytarabine
YPL085W SEC16
ZShift = 2.34
NG = 0
DB = 0
SM = 0
Delta_K
Cytarabine
YPL085W SEC16
ZShift = 0.36
NG = 0
DB = 0
SM = 0
Delta_r
Cytarabine
YPL085W SEC16
ZShift = 0.08
NG = 0
DB = 0
SM = 0
Delta_AUC
Cytarabine
YCR057C PWP2
ZShift = 1.49
NG = 0
DB = 0
SM = 0
Delta_L
Cytarabine
YCR057C PWP2
ZShift = 2.46
NG = 0
DB = 0
SM = 0
Delta_K
Cytarabine
YCR057C PWP2
ZShift = 1.19
NG = 0
DB = 0
SM = 0
Delta_r
Cytarabine
YCR057C PWP2
ZShift = 0.98
NG = 0
DB = 0
SM = 0
Delta_AUC
Cytarabine
0
10
25
50
100

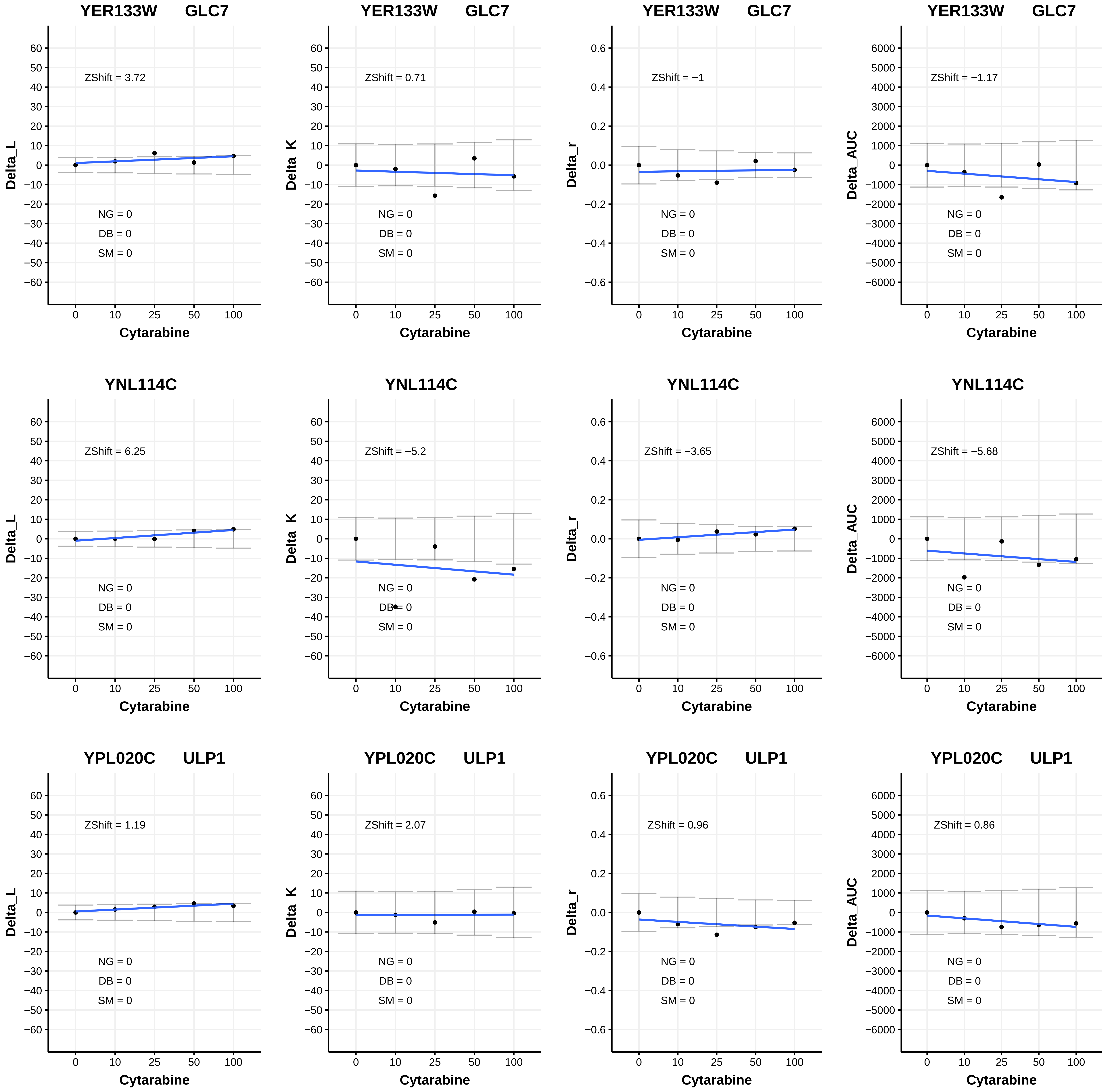

YER133W GLC7
ZShift = 3.72
ZShift = 0.71
ZShift = −1
ZShift = −1.17
YNL114C
ZShift = 6.25
ZShift = −5.2
ZShift = −3.65
ZShift = −5.68
YPL020C ULP1
ZShift = 1.19
ZShift = 2.07
ZShift = 0.96
ZShift = 0.86
NG = 0
DB = 0
SM = 0
Delta_L
Delta_K
Delta_r
Delta_AUC
Cytarabine

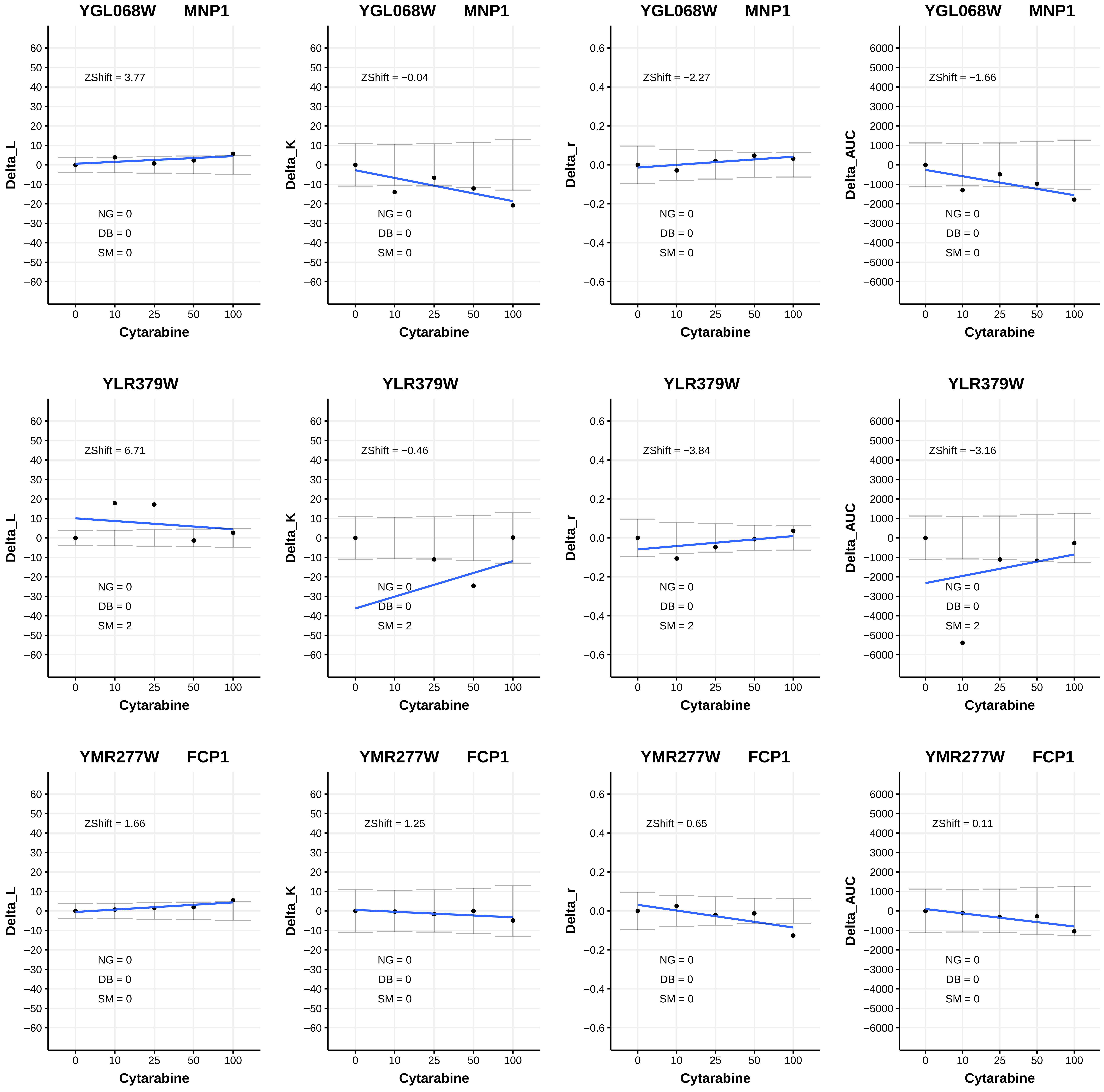
YGL068W MNP1
ZShift = 3.77
NG = 0
DB = 0
SM = 0
Delta_L
Cytarabine
YGL068W MNP1
ZShift = −0.04
NG = 0
DB = 0
SM = 0
Delta_K
Cytarabine
YGL068W MNP1
ZShift = −2.27
NG = 0
DB = 0
SM = 0
Delta_r
Cytarabine
YGL068W MNP1
ZShift = −1.66
NG = 0
DB = 0
SM = 0
Delta_AUC
Cytarabine
YLR379W
ZShift = 6.71
NG = 0
DB = 0
SM = 2
Delta_L
Cytarabine
YLR379W
ZShift = −0.46
NG = 0
DB = 0
SM = 2
Delta_K
Cytarabine
YLR379W
ZShift = −3.84
NG = 0
DB = 0
SM = 2
Delta_r
Cytarabine
YLR379W
ZShift = −3.16
NG = 0
DB = 0
SM = 2
Delta_AUC
Cytarabine
YMR277W FCP1
ZShift = 1.66
NG = 0
DB = 0
SM = 0
Delta_L
Cytarabine
YMR277W FCP1
ZShift = 1.25
NG = 0
DB = 0
SM = 0
Delta_K
Cytarabine
YMR277W FCP1
ZShift = 0.65
NG = 0
DB = 0
SM = 0
Delta_r
Cytarabine
YMR277W FCP1
ZShift = 0.11
NG = 0
DB = 0
SM = 0
Delta_AUC
Cytarabine

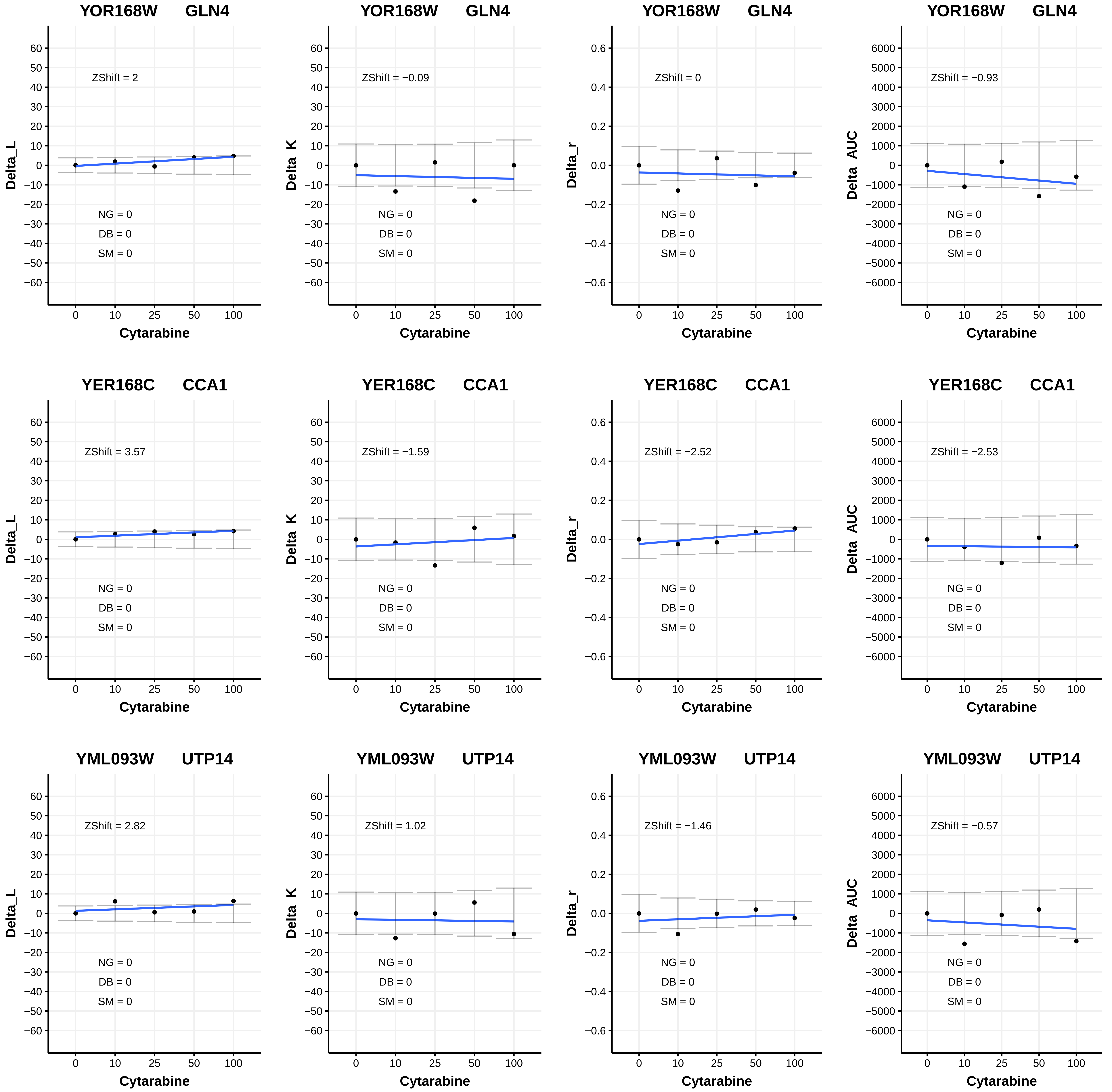
YOR168W GLN4
ZShift = 2
ZShift = −0.09
ZShift = 0
ZShift = −0.93
YER168C CCA1
ZShift = 3.57
ZShift = −1.59
ZShift = −2.52
ZShift = −2.53
YML093W UTP14
ZShift = 2.82
ZShift = 1.02
ZShift = −1.46
ZShift = −0.57
NG = 0
DB = 0
SM = 0
Delta_L
Delta_K
Delta_r
Delta_AUC
Cytarabine
0 10 25 50 100

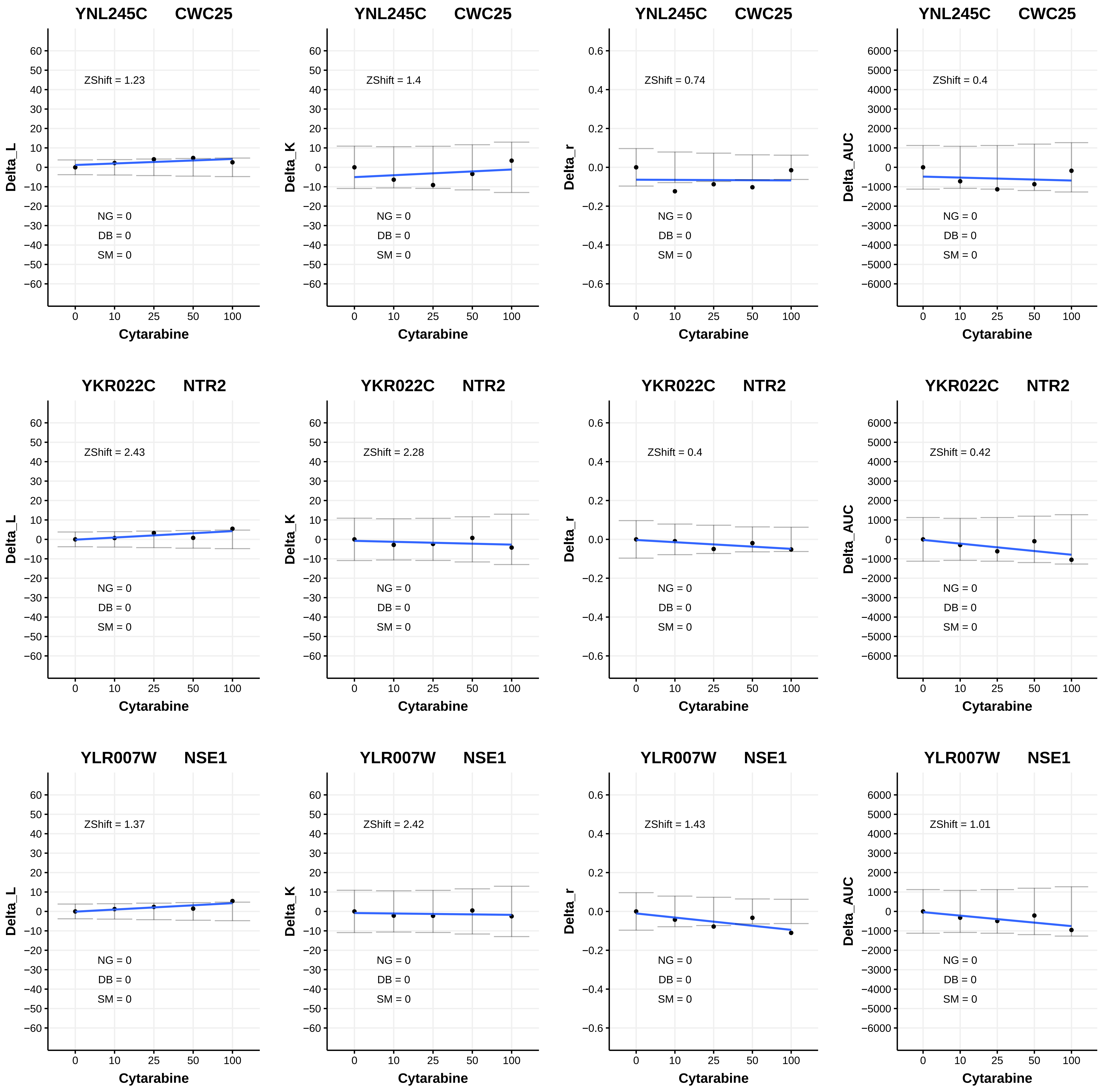

YNL245C CWC25
ZShift = 1.23
NG = 0
DB = 0
SM = 0
Delta_L
Cytarabine
YNL245C CWC25
ZShift = 1.4
NG = 0
DB = 0
SM = 0
Delta_K
Cytarabine
YNL245C CWC25
ZShift = 0.74
NG = 0
DB = 0
SM = 0
Delta_r
Cytarabine
YNL245C CWC25
ZShift = 0.4
NG = 0
DB = 0
SM = 0
Delta_AUC
Cytarabine
YKR022C NTR2
ZShift = 2.43
NG = 0
DB = 0
SM = 0
Delta_L
Cytarabine
YKR022C NTR2
ZShift = 2.28
NG = 0
DB = 0
SM = 0
Delta_K
Cytarabine
YKR022C NTR2
ZShift = 0.4
NG = 0
DB = 0
SM = 0
Delta_r
Cytarabine
YKR022C NTR2
ZShift = 0.42
NG = 0
DB = 0
SM = 0
Delta_AUC
Cytarabine
YLR007W NSE1
ZShift = 1.37
NG = 0
DB = 0
SM = 0
Delta_L
Cytarabine
YLR007W NSE1
ZShift = 2.42
NG = 0
DB = 0
SM = 0
Delta_K
Cytarabine
YLR007W NSE1
ZShift = 1.43
NG = 0
DB = 0
SM = 0
Delta_r
Cytarabine
YLR007W NSE1
ZShift = 1.01
NG = 0
DB = 0
SM = 0
Delta_AUC
Cytarabine

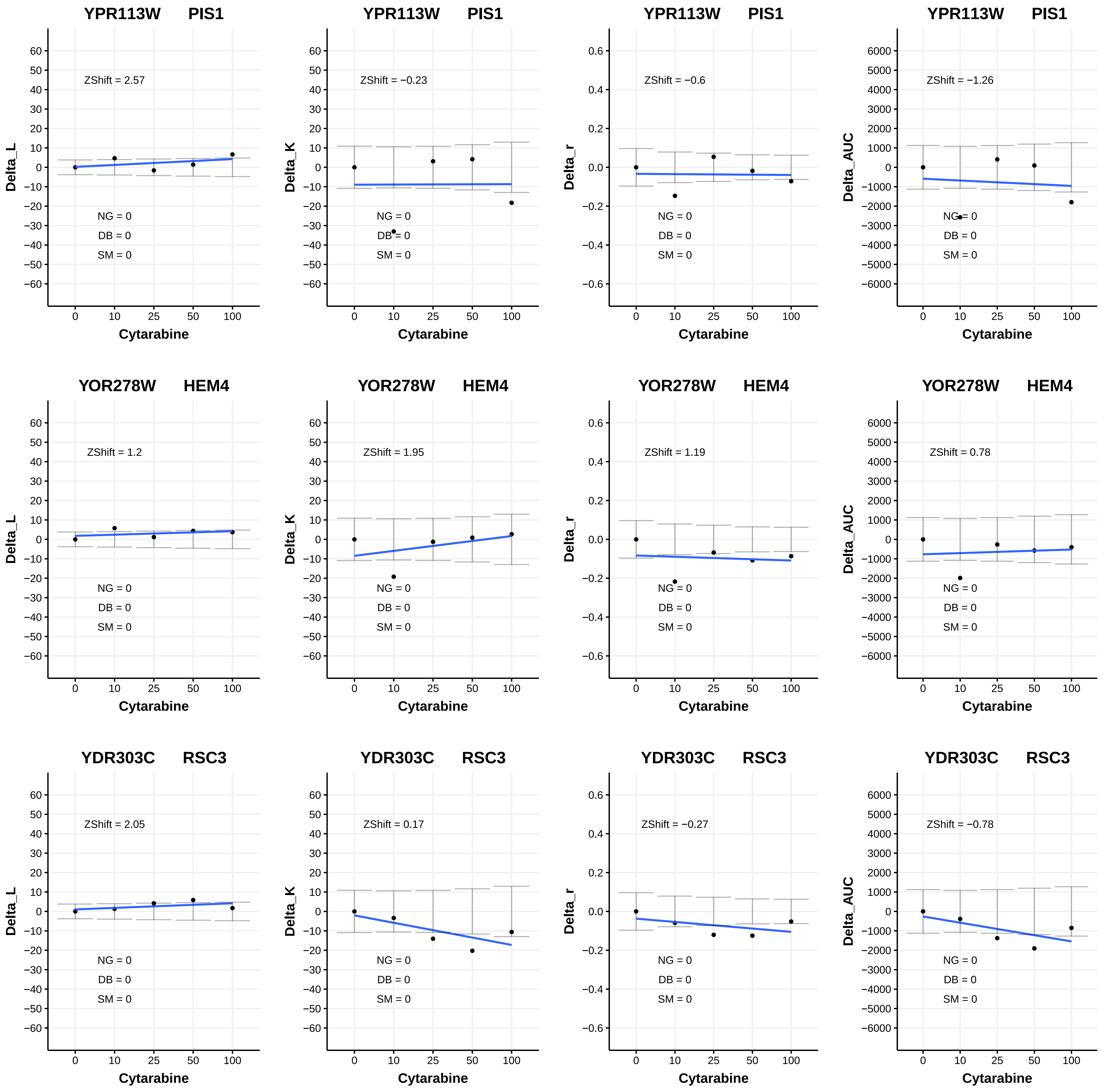
YPR113W PIS1
ZShift = 2.57
Delta_L
NG = 0
DB = 0
SM = 0
Cytarabine
YPR113W PIS1
ZShift = −0.23
Delta_K
NG = 0
DB = 0
SM = 0
Cytarabine
YPR113W PIS1
ZShift = −0.6
Delta_r
NG = 0
DB = 0
SM = 0
Cytarabine
YPR113W PIS1
ZShift = −1.26
Delta_AUC
NG = 0
DB = 0
SM = 0
Cytarabine
YOR278W HEM4
ZShift = 1.2
Delta_L
NG = 0
DB = 0
SM = 0
Cytarabine
YOR278W HEM4
ZShift = 1.95
Delta_K
NG = 0
DB = 0
SM = 0
Cytarabine
YOR278W HEM4
ZShift = 1.19
Delta_r
NG = 0
DB = 0
SM = 0
Cytarabine
YOR278W HEM4
ZShift = 0.78
Delta_AUC
NG = 0
DB = 0
SM = 0
Cytarabine
YDR303C RSC3
ZShift = 2.05
Delta_L
NG = 0
DB = 0
SM = 0
Cytarabine
YDR303C RSC3
ZShift = 0.17
Delta_K
NG = 0
DB = 0
SM = 0
Cytarabine
YDR303C RSC3
ZShift = −0.27
Delta_r
NG = 0
DB = 0
SM = 0
Cytarabine
YDR303C RSC3
ZShift = −0.78
Delta_AUC
NG = 0
DB = 0
SM = 0
Cytarabine

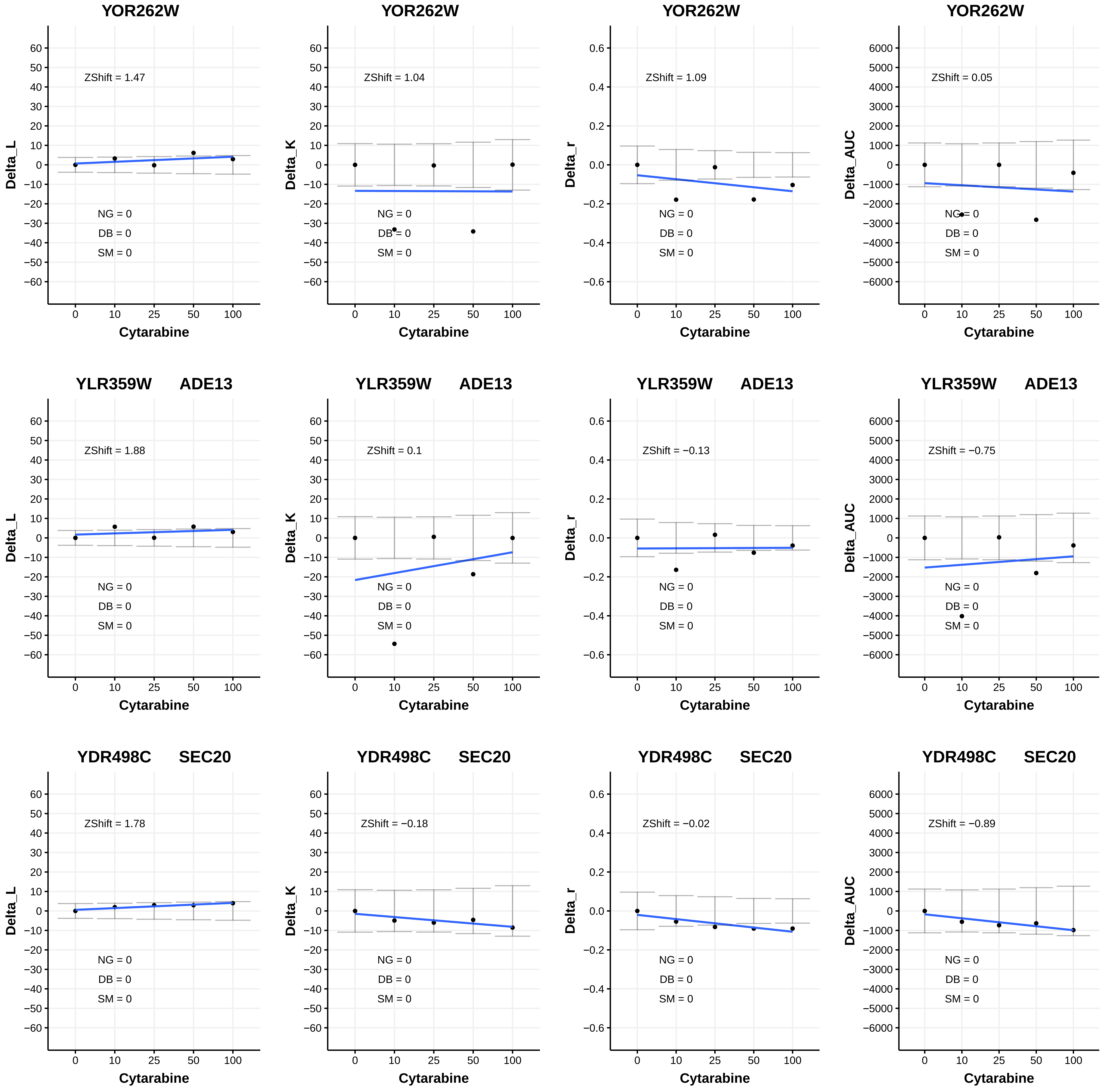
YOR262W
ZShift = 1.47
NG = 0
DB = 0
SM = 0
Delta_L
Cytarabine
YOR262W
ZShift = 1.04
NG = 0
DB = 0
SM = 0
Delta_K
Cytarabine
YOR262W
ZShift = 1.09
NG = 0
DB = 0
SM = 0
Delta_r
Cytarabine
YOR262W
ZShift = 0.05
NG = 0
DB = 0
SM = 0
Delta_AUC
Cytarabine
YLR359W ADE13
ZShift = 1.88
NG = 0
DB = 0
SM = 0
Delta_L
Cytarabine
YLR359W ADE13
ZShift = 0.1
NG = 0
DB = 0
SM = 0
Delta_K
Cytarabine
YLR359W ADE13
ZShift = −0.13
NG = 0
DB = 0
SM = 0
Delta_r
Cytarabine
YLR359W ADE13
ZShift = −0.75
NG = 0
DB = 0
SM = 0
Delta_AUC
Cytarabine
YDR498C SEC20
ZShift = 1.78
NG = 0
DB = 0
SM = 0
Delta_L
Cytarabine
YDR498C SEC20
ZShift = −0.18
NG = 0
DB = 0
SM = 0
Delta_K
Cytarabine
YDR498C SEC20
ZShift = −0.02
NG = 0
DB = 0
SM = 0
Delta_r
Cytarabine
YDR498C SEC20
ZShift = −0.89
NG = 0
DB = 0
SM = 0
Delta_AUC
Cytarabine

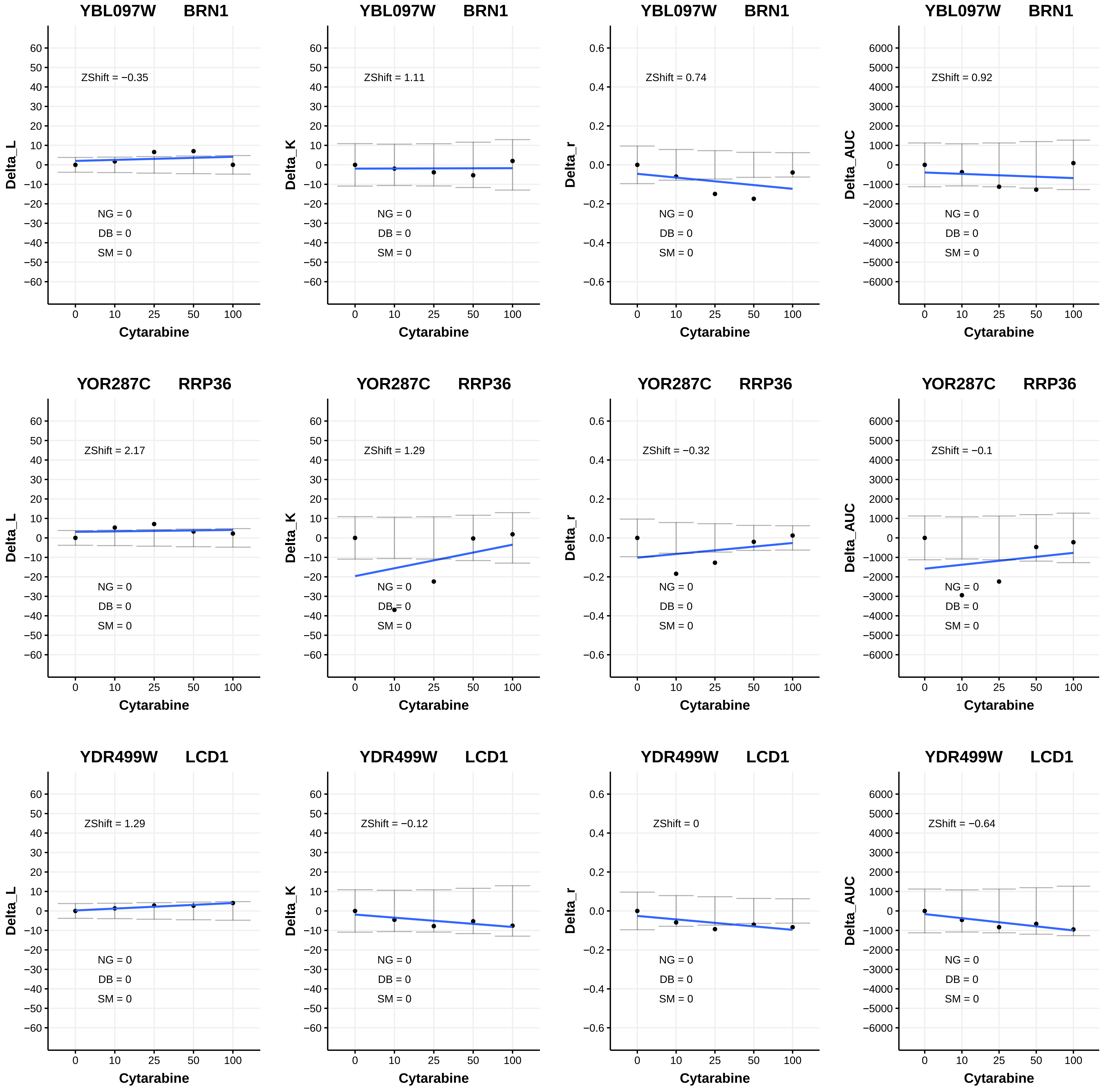
YBL097W BRN1
Delta_L
ZShift = −0.35
NG = 0
DB = 0
SM = 0
Cytarabine
YBL097W BRN1
Delta_K
ZShift = 1.11
NG = 0
DB = 0
SM = 0
Cytarabine
YBL097W BRN1
Delta_r
ZShift = 0.74
NG = 0
DB = 0
SM = 0
Cytarabine
YBL097W BRN1
Delta_AUC
ZShift = 0.92
NG = 0
DB = 0
SM = 0
Cytarabine
YOR287C RRP36
Delta_L
ZShift = 2.17
NG = 0
DB = 0
SM = 0
Cytarabine
YOR287C RRP36
Delta_K
ZShift = 1.29
NG = 0
DB = 0
SM = 0
Cytarabine
YOR287C RRP36
Delta_r
ZShift = −0.32
NG = 0
DB = 0
SM = 0
Cytarabine
YOR287C RRP36
Delta_AUC
ZShift = −0.1
NG = 0
DB = 0
SM = 0
Cytarabine
YDR499W LCD1
Delta_L
ZShift = 1.29
NG = 0
DB = 0
SM = 0
Cytarabine
YDR499W LCD1
Delta_K
ZShift = −0.12
NG = 0
DB = 0
SM = 0
Cytarabine
YDR499W LCD1
Delta_r
ZShift = 0
NG = 0
DB = 0
SM = 0
Cytarabine
YDR499W LCD1
Delta_AUC
ZShift = −0.64
NG = 0
DB = 0
SM = 0
Cytarabine

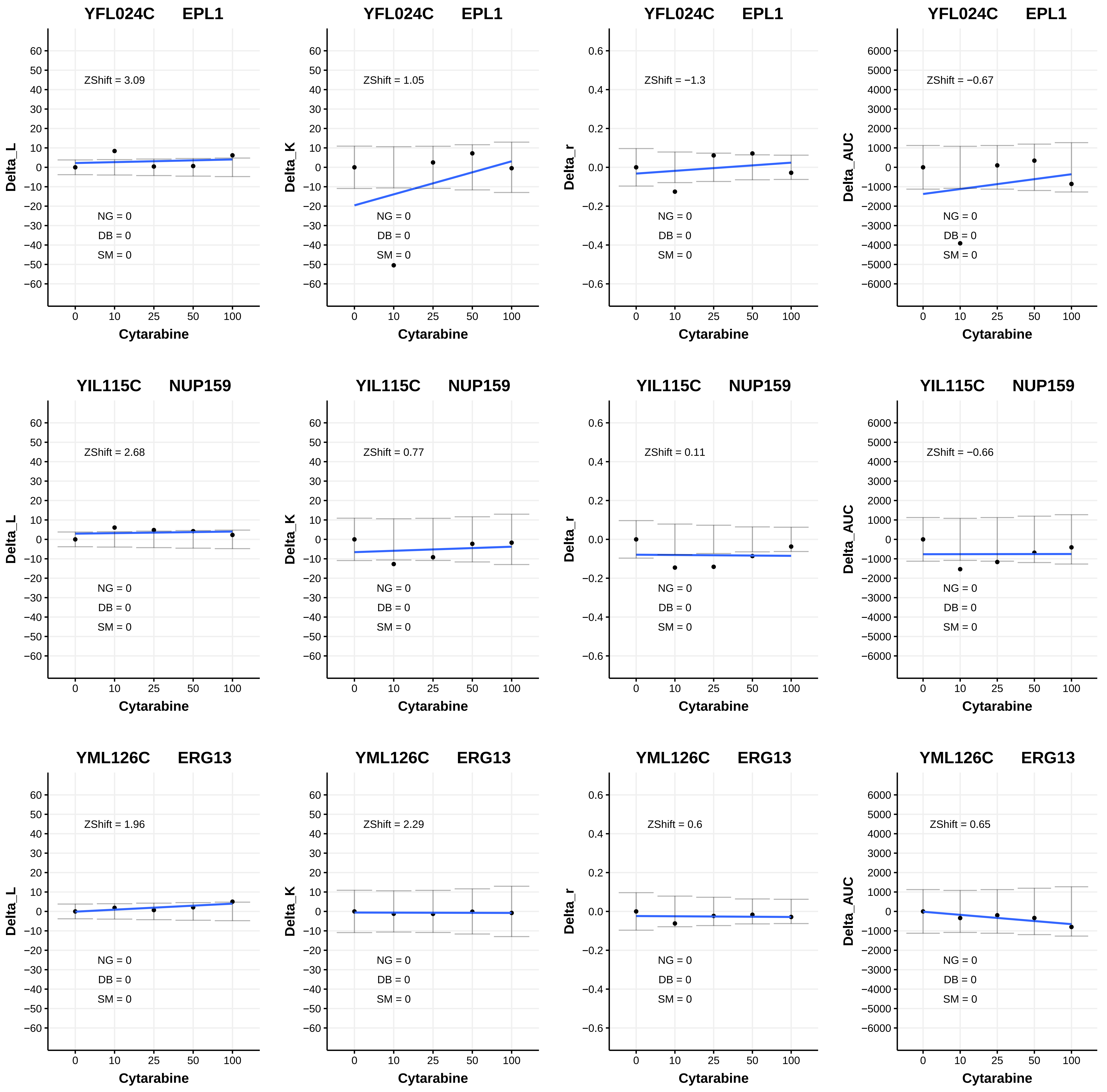
YFL024C EPL1
ZShift = 3.09
NG = 0
DB = 0
SM = 0
Delta_L
Cytarabine
YFL024C EPL1
ZShift = 1.05
NG = 0
DB = 0
SM = 0
Delta_K
Cytarabine
YFL024C EPL1
ZShift = −1.3
NG = 0
DB = 0
SM = 0
Delta_r
Cytarabine
YFL024C EPL1
ZShift = −0.67
NG = 0
DB = 0
SM = 0
Delta_AUC
Cytarabine
YIL115C NUP159
ZShift = 2.68
NG = 0
DB = 0
SM = 0
Delta_L
Cytarabine
YIL115C NUP159
ZShift = 0.77
NG = 0
DB = 0
SM = 0
Delta_K
Cytarabine
YIL115C NUP159
ZShift = 0.11
NG = 0
DB = 0
SM = 0
Delta_r
Cytarabine
YIL115C NUP159
ZShift = −0.66
NG = 0
DB = 0
SM = 0
Delta_AUC
Cytarabine
YML126C ERG13
ZShift = 1.96
NG = 0
DB = 0
SM = 0
Delta_L
Cytarabine
YML126C ERG13
ZShift = 2.29
NG = 0
DB = 0
SM = 0
Delta_K
Cytarabine
YML126C ERG13
ZShift = 0.6
NG = 0
DB = 0
SM = 0
Delta_r
Cytarabine
YML126C ERG13
ZShift = 0.65
NG = 0
DB = 0
SM = 0
Delta_AUC
Cytarabine

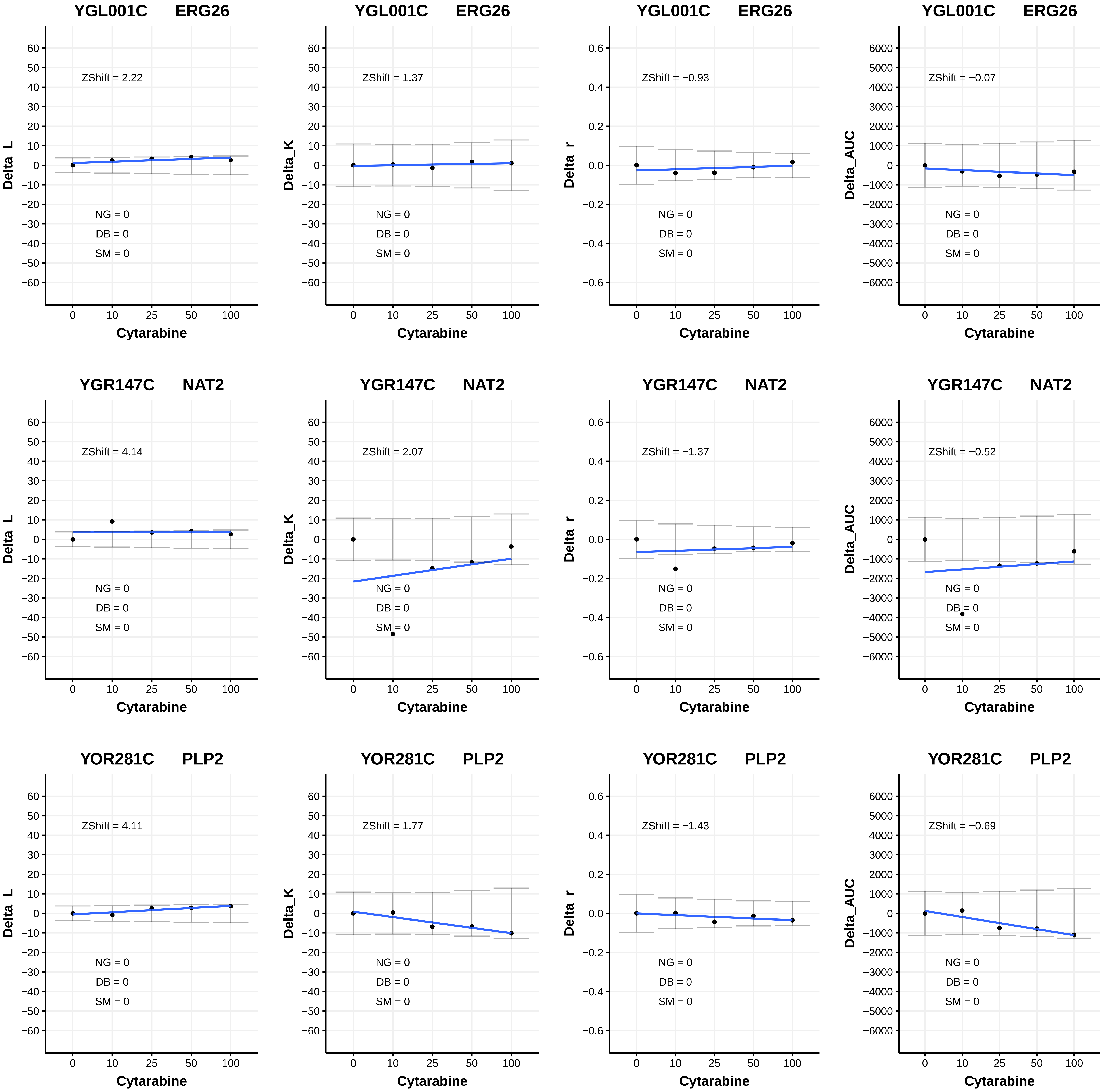
YGL001C ERG26
ZShift = 2.22
Delta_L
NG = 0
DB = 0
SM = 0
Cytarabine
YGL001C ERG26
ZShift = 1.37
Delta_K
NG = 0
DB = 0
SM = 0
Cytarabine
YGL001C ERG26
ZShift = −0.93
Delta_r
NG = 0
DB = 0
SM = 0
Cytarabine
YGL001C ERG26
ZShift = −0.07
Delta_AUC
NG = 0
DB = 0
SM = 0
Cytarabine
YGR147C NAT2
ZShift = 4.14
Delta_L
NG = 0
DB = 0
SM = 0
Cytarabine
YGR147C NAT2
ZShift = 2.07
Delta_K
NG = 0
DB = 0
SM = 0
Cytarabine
YGR147C NAT2
ZShift = −1.37
Delta_r
NG = 0
DB = 0
SM = 0
Cytarabine
YGR147C NAT2
ZShift = −0.52
Delta_AUC
NG = 0
DB = 0
SM = 0
Cytarabine
YOR281C PLP2
ZShift = 4.11
Delta_L
NG = 0
DB = 0
SM = 0
Cytarabine
YOR281C PLP2
ZShift = 1.77
Delta_K
NG = 0
DB = 0
SM = 0
Cytarabine
YOR281C PLP2
ZShift = −1.43
Delta_r
NG = 0
DB = 0
SM = 0
Cytarabine
YOR281C PLP2
ZShift = −0.69
Delta_AUC
NG = 0
DB = 0
SM = 0
Cytarabine

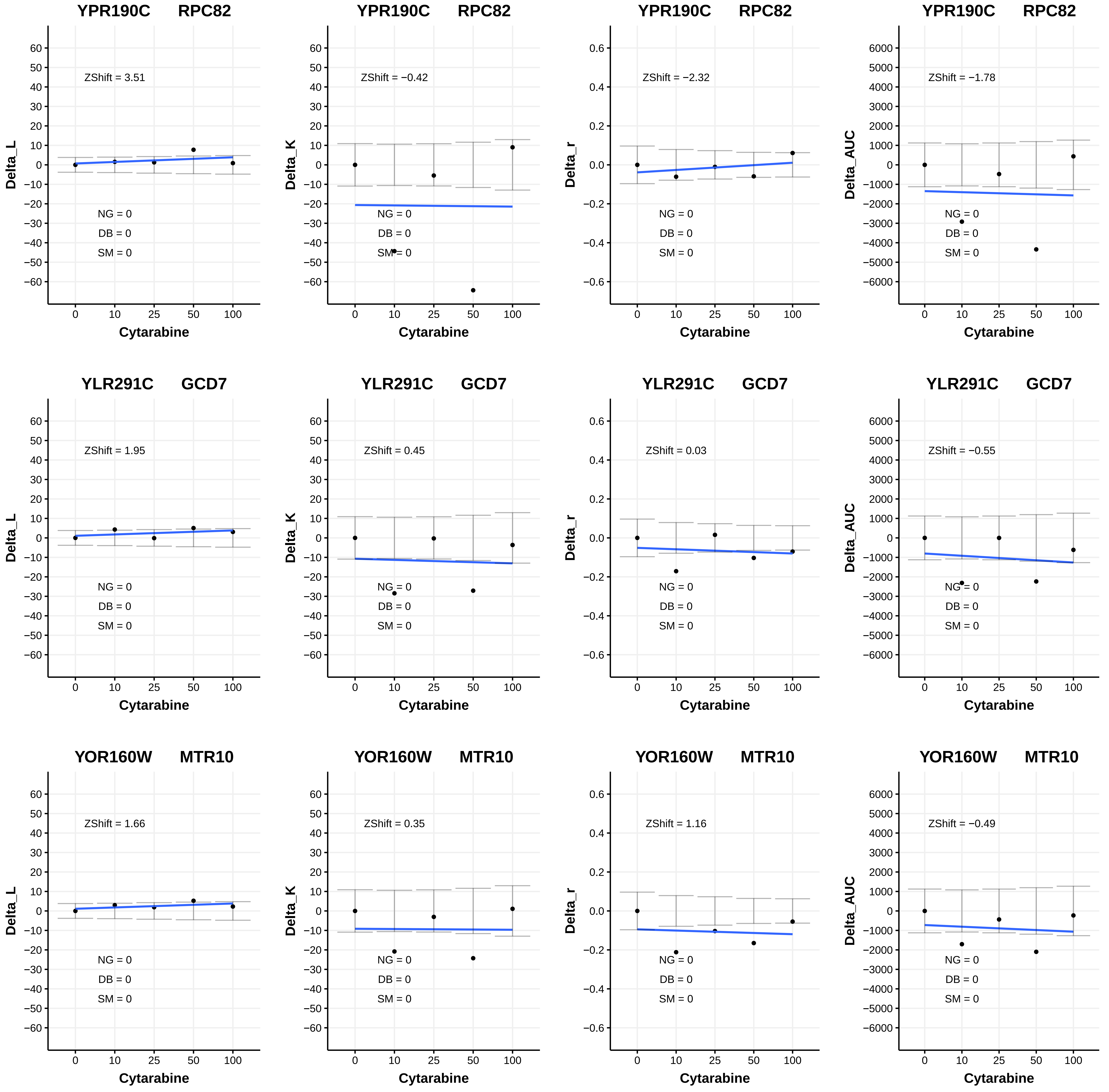
YPR190C RPC82
ZShift = 3.51
NG = 0
DB = 0
SM = 0
Delta_L
Cytarabine
YPR190C RPC82
ZShift = −0.42
NG = 0
DB = 0
SM = 0
Delta_K
Cytarabine
YPR190C RPC82
ZShift = −2.32
NG = 0
DB = 0
SM = 0
Delta_r
Cytarabine
YPR190C RPC82
ZShift = −1.78
NG = 0
DB = 0
SM = 0
Delta_AUC
Cytarabine
YLR291C GCD7
ZShift = 1.95
NG = 0
DB = 0
SM = 0
Delta_L
Cytarabine
YLR291C GCD7
ZShift = 0.45
NG = 0
DB = 0
SM = 0
Delta_K
Cytarabine
YLR291C GCD7
ZShift = 0.03
NG = 0
DB = 0
SM = 0
Delta_r
Cytarabine
YLR291C GCD7
ZShift = −0.55
NG = 0
DB = 0
SM = 0
Delta_AUC
Cytarabine
YOR160W MTR10
ZShift = 1.66
NG = 0
DB = 0
SM = 0
Delta_L
Cytarabine
YOR160W MTR10
ZShift = 0.35
NG = 0
DB = 0
SM = 0
Delta_K
Cytarabine
YOR160W MTR10
ZShift = 1.16
NG = 0
DB = 0
SM = 0
Delta_r
Cytarabine
YOR160W MTR10
ZShift = −0.49
NG = 0
DB = 0
SM = 0
Delta_AUC
Cytarabine

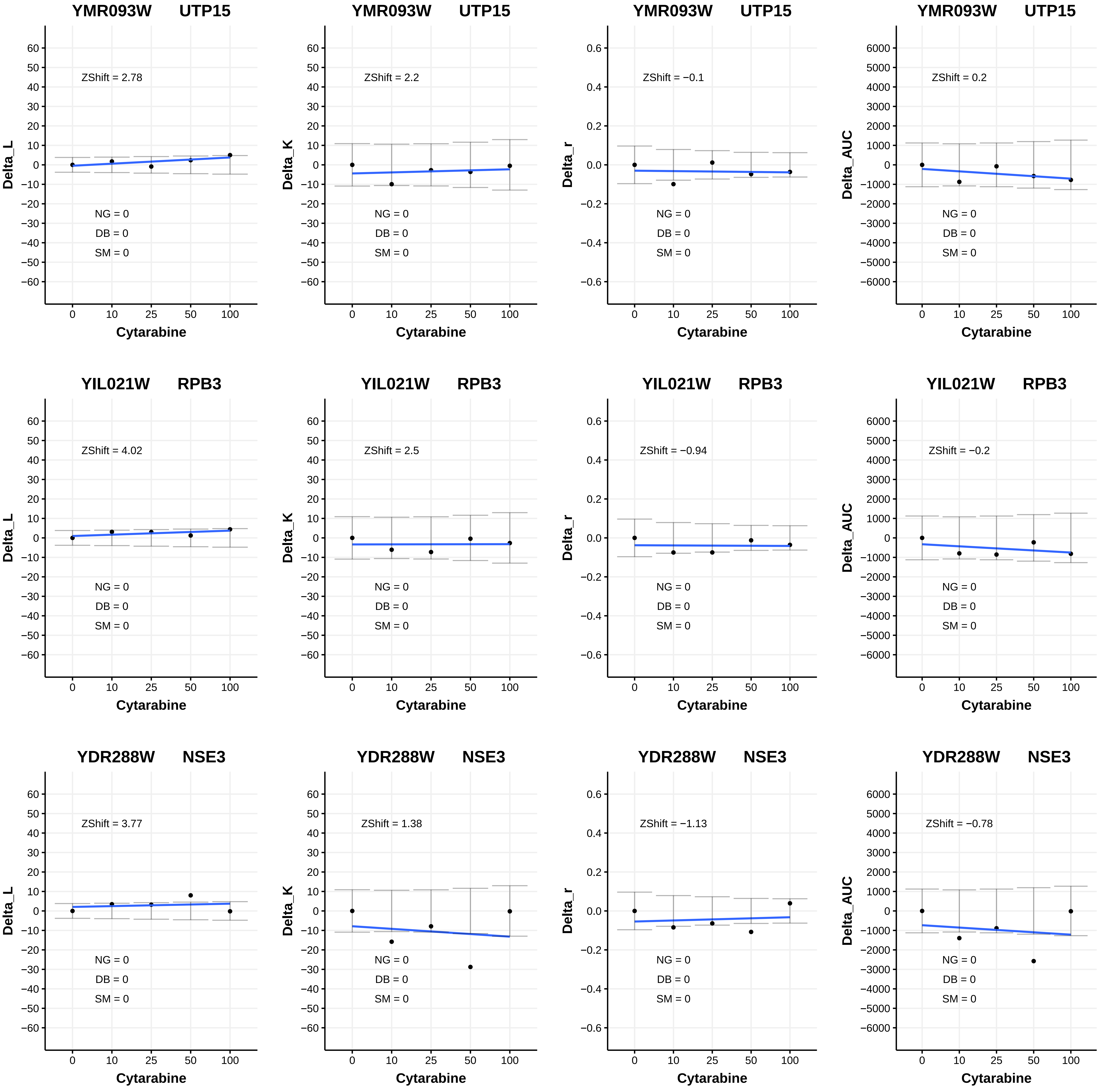

YMR093W UTP15
ZShift = 2.78
NG = 0
DB = 0
SM = 0
Delta_L
Cytarabine
YMR093W UTP15
ZShift = 2.2
NG = 0
DB = 0
SM = 0
Delta_K
Cytarabine
YMR093W UTP15
ZShift = −0.1
NG = 0
DB = 0
SM = 0
Delta_r
Cytarabine
YMR093W UTP15
ZShift = 0.2
NG = 0
DB = 0
SM = 0
Delta_AUC
Cytarabine
YIL021W RPB3
ZShift = 4.02
NG = 0
DB = 0
SM = 0
Delta_L
Cytarabine
YIL021W RPB3
ZShift = 2.5
NG = 0
DB = 0
SM = 0
Delta_K
Cytarabine
YIL021W RPB3
ZShift = −0.94
NG = 0
DB = 0
SM = 0
Delta_r
Cytarabine
YIL021W RPB3
ZShift = −0.2
NG = 0
DB = 0
SM = 0
Delta_AUC
Cytarabine
YDR288W NSE3
ZShift = 3.77
NG = 0
DB = 0
SM = 0
Delta_L
Cytarabine
YDR288W NSE3
ZShift = 1.38
NG = 0
DB = 0
SM = 0
Delta_K
Cytarabine
YDR288W NSE3
ZShift = −1.13
NG = 0
DB = 0
SM = 0
Delta_r
Cytarabine
YDR288W NSE3
ZShift = −0.78
NG = 0
DB = 0
SM = 0
Delta_AUC
Cytarabine

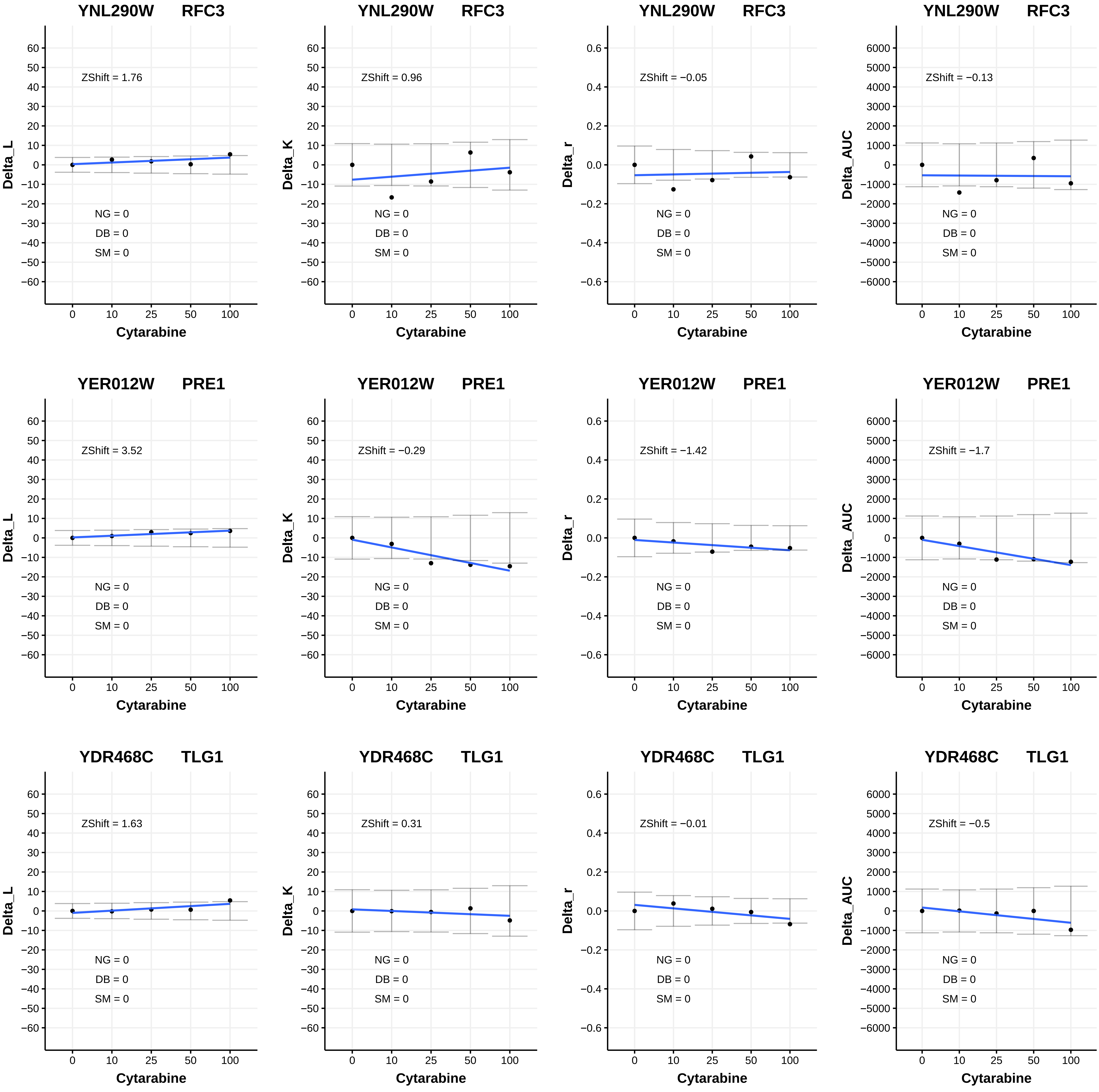
YNL290W RFC3
ZShift = 1.76
Delta_L
NG = 0
DB = 0
SM = 0
Cytarabine
YNL290W RFC3
ZShift = 0.96
Delta_K
NG = 0
DB = 0
SM = 0
Cytarabine
YNL290W RFC3
ZShift = −0.05
Delta_r
NG = 0
DB = 0
SM = 0
Cytarabine
YNL290W RFC3
ZShift = −0.13
Delta_AUC
NG = 0
DB = 0
SM = 0
Cytarabine
YER012W PRE1
ZShift = 3.52
Delta_L
NG = 0
DB = 0
SM = 0
Cytarabine
YER012W PRE1
ZShift = −0.29
Delta_K
NG = 0
DB = 0
SM = 0
Cytarabine
YER012W PRE1
ZShift = −1.42
Delta_r
NG = 0
DB = 0
SM = 0
Cytarabine
YER012W PRE1
ZShift = −1.7
Delta_AUC
NG = 0
DB = 0
SM = 0
Cytarabine
YDR468C TLG1
ZShift = 1.63
Delta_L
NG = 0
DB = 0
SM = 0
Cytarabine
YDR468C TLG1
ZShift = 0.31
Delta_K
NG = 0
DB = 0
SM = 0
Cytarabine
YDR468C TLG1
ZShift = −0.01
Delta_r
NG = 0
DB = 0
SM = 0
Cytarabine
YDR468C TLG1
ZShift = −0.5
Delta_AUC
NG = 0
DB = 0
SM = 0
Cytarabine

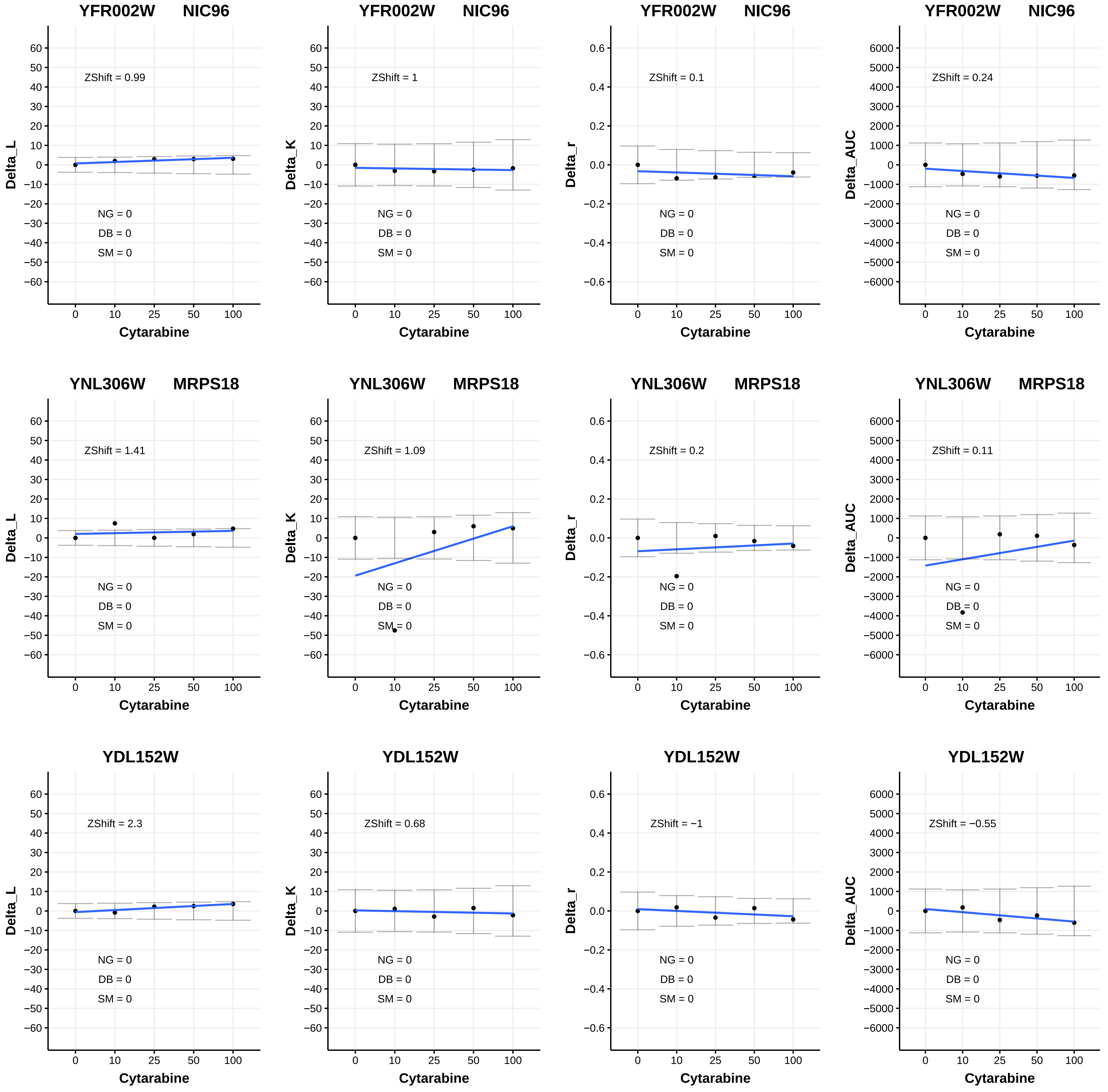
YFR002W NIC96
ZShift = 0.99
Delta_L
NG = 0
DB = 0
SM = 0
Cytarabine
YFR002W NIC96
ZShift = 1
Delta_K
NG = 0
DB = 0
SM = 0
Cytarabine
YFR002W NIC96
ZShift = 0.1
Delta_r
NG = 0
DB = 0
SM = 0
Cytarabine
YFR002W NIC96
ZShift = 0.24
Delta_AUC
NG = 0
DB = 0
SM = 0
Cytarabine
YNL306W MRPS18
ZShift = 1.41
Delta_L
NG = 0
DB = 0
SM = 0
Cytarabine
YNL306W MRPS18
ZShift = 1.09
Delta_K
NG = 0
DB = 0
SM = 0
Cytarabine
YNL306W MRPS18
ZShift = 0.2
Delta_r
NG = 0
DB = 0
SM = 0
Cytarabine
YNL306W MRPS18
ZShift = 0.11
Delta_AUC
NG = 0
DB = 0
SM = 0
Cytarabine
YDL152W
ZShift = 2.3
Delta_L
NG = 0
DB = 0
SM = 0
Cytarabine
YDL152W
ZShift = 0.68
Delta_K
NG = 0
DB = 0
SM = 0
Cytarabine
YDL152W
ZShift = −1
Delta_r
NG = 0
DB = 0
SM = 0
Cytarabine
YDL152W
ZShift = −0.55
Delta_AUC
NG = 0
DB = 0
SM = 0
Cytarabine

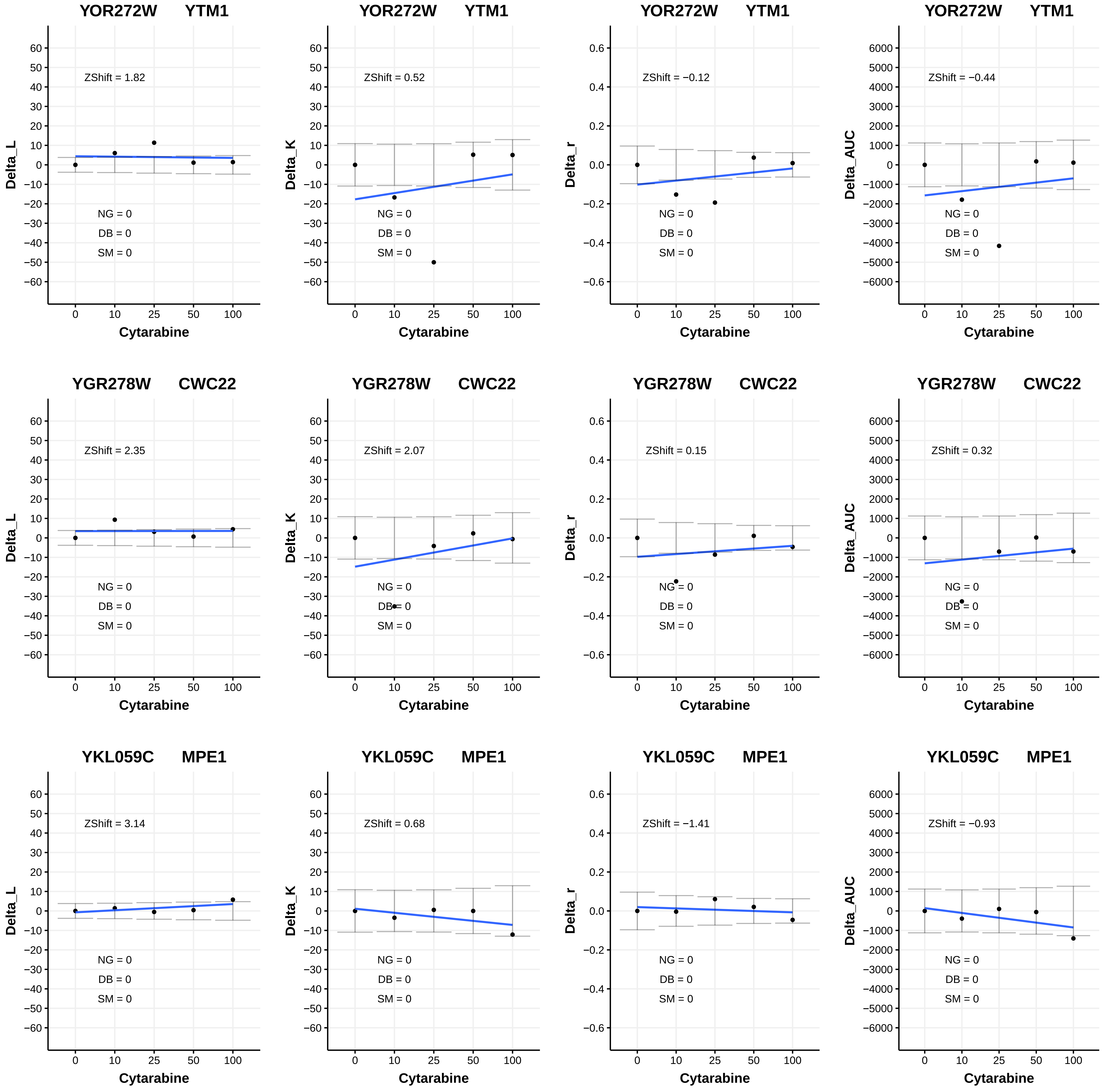

YOR272W YTM1
ZShift = 1.82
NG = 0
DB = 0
SM = 0
Delta_L
Cytarabine
YOR272W YTM1
ZShift = 0.52
NG = 0
DB = 0
SM = 0
Delta_K
Cytarabine
YOR272W YTM1
ZShift = −0.12
NG = 0
DB = 0
SM = 0
Delta_r
Cytarabine
YOR272W YTM1
ZShift = −0.44
NG = 0
DB = 0
SM = 0
Delta_AUC
Cytarabine
YGR278W CWC22
ZShift = 2.35
NG = 0
DB = 0
SM = 0
Delta_L
Cytarabine
YGR278W CWC22
ZShift = 2.07
NG = 0
DB = 0
SM = 0
Delta_K
Cytarabine
YGR278W CWC22
ZShift = 0.15
NG = 0
DB = 0
SM = 0
Delta_r
Cytarabine
YGR278W CWC22
ZShift = 0.32
NG = 0
DB = 0
SM = 0
Delta_AUC
Cytarabine
YKL059C MPE1
ZShift = 3.14
NG = 0
DB = 0
SM = 0
Delta_L
Cytarabine
YKL059C MPE1
ZShift = 0.68
NG = 0
DB = 0
SM = 0
Delta_K
Cytarabine
YKL059C MPE1
ZShift = −1.41
NG = 0
DB = 0
SM = 0
Delta_r
Cytarabine
YKL059C MPE1
ZShift = −0.93
NG = 0
DB = 0
SM = 0
Delta_AUC
Cytarabine

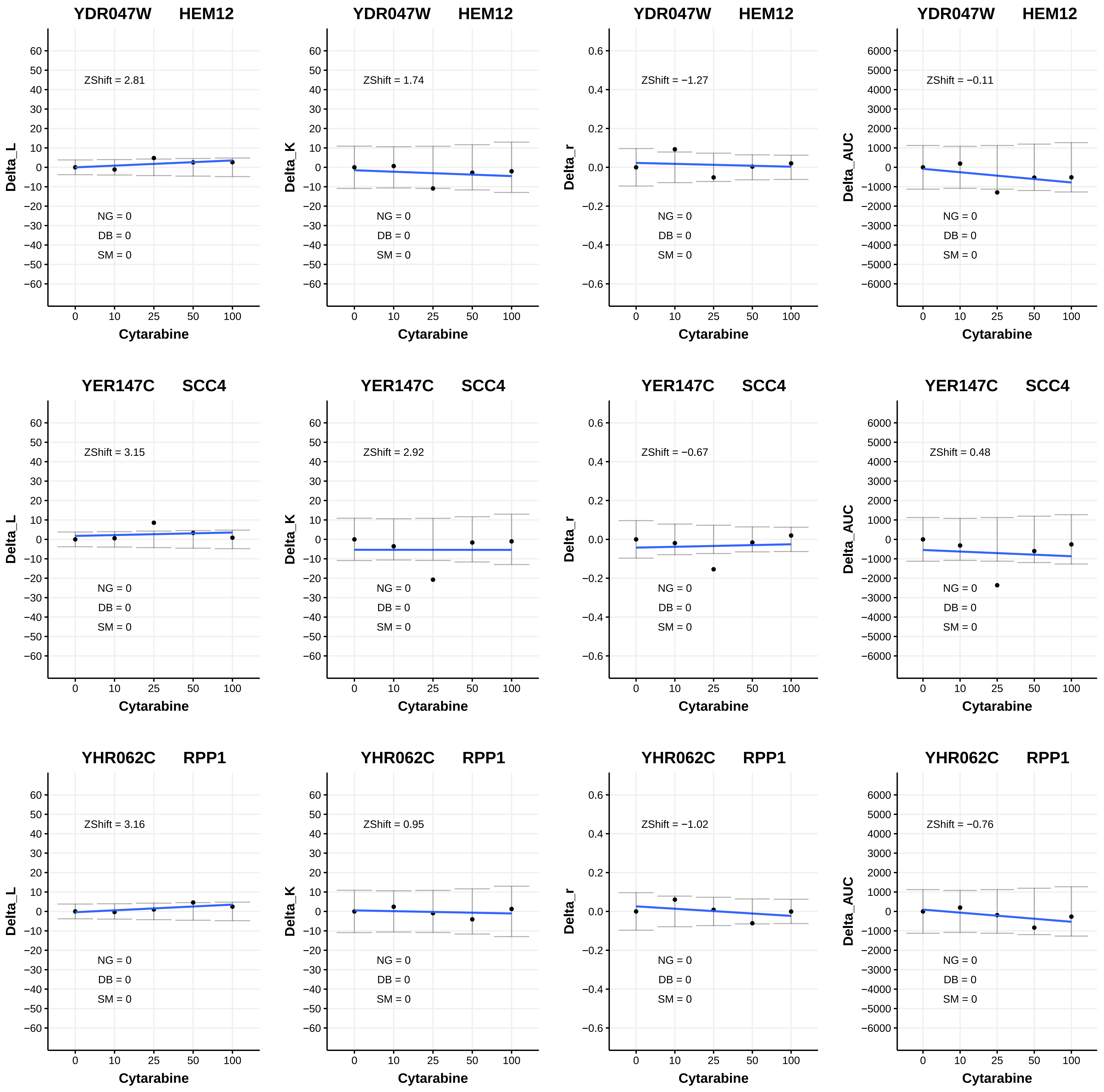
YDR047W HEM12
ZShift = 2.81
NG = 0
DB = 0
SM = 0
Delta_L
Cytarabine
YDR047W HEM12
ZShift = 1.74
NG = 0
DB = 0
SM = 0
Delta_K
Cytarabine
YDR047W HEM12
ZShift = −1.27
NG = 0
DB = 0
SM = 0
Delta_r
Cytarabine
YDR047W HEM12
ZShift = −0.11
NG = 0
DB = 0
SM = 0
Delta_AUC
Cytarabine
YER147C SCC4
ZShift = 3.15
NG = 0
DB = 0
SM = 0
Delta_L
Cytarabine
YER147C SCC4
ZShift = 2.92
NG = 0
DB = 0
SM = 0
Delta_K
Cytarabine
YER147C SCC4
ZShift = −0.67
NG = 0
DB = 0
SM = 0
Delta_r
Cytarabine
YER147C SCC4
ZShift = 0.48
NG = 0
DB = 0
SM = 0
Delta_AUC
Cytarabine
YHR062C RPP1
ZShift = 3.16
NG = 0
DB = 0
SM = 0
Delta_L
Cytarabine
YHR062C RPP1
ZShift = 0.95
NG = 0
DB = 0
SM = 0
Delta_K
Cytarabine
YHR062C RPP1
ZShift = −1.02
NG = 0
DB = 0
SM = 0
Delta_r
Cytarabine
YHR062C RPP1
ZShift = −0.76
NG = 0
DB = 0
SM = 0
Delta_AUC
Cytarabine

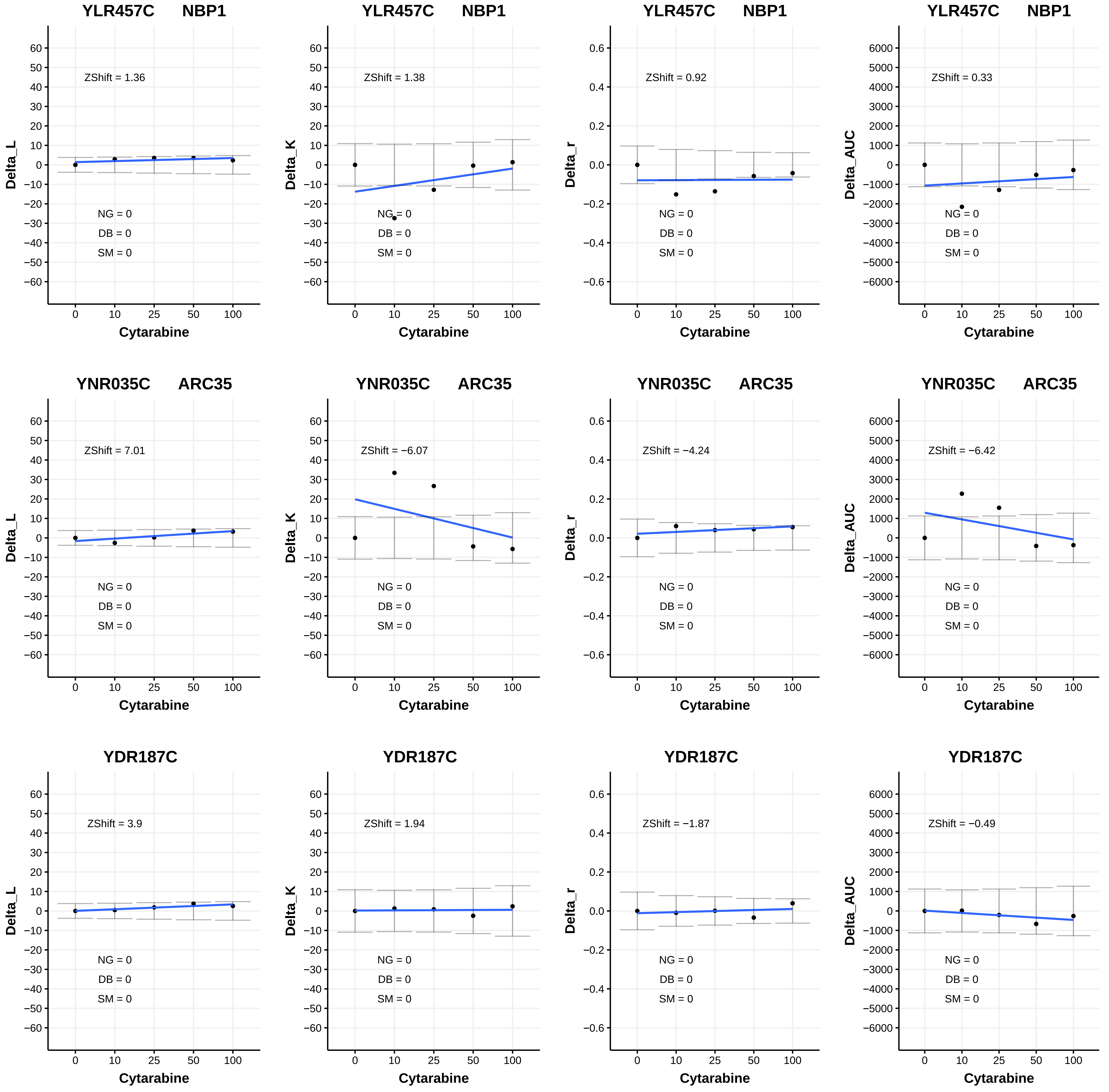
YLR457C NBP1
ZShift = 1.36
ZShift = 1.38
ZShift = 0.92
ZShift = 0.33
YNR035C ARC35
ZShift = 7.01
ZShift = −6.07
ZShift = −4.24
ZShift = −6.42
YDR187C
ZShift = 3.9
ZShift = 1.94
ZShift = −1.87
ZShift = −0.49
NG = 0
DB = 0
SM = 0
Delta_L
Delta_K
Delta_r
Delta_AUC
Cytarabine
0 10 25 50 100

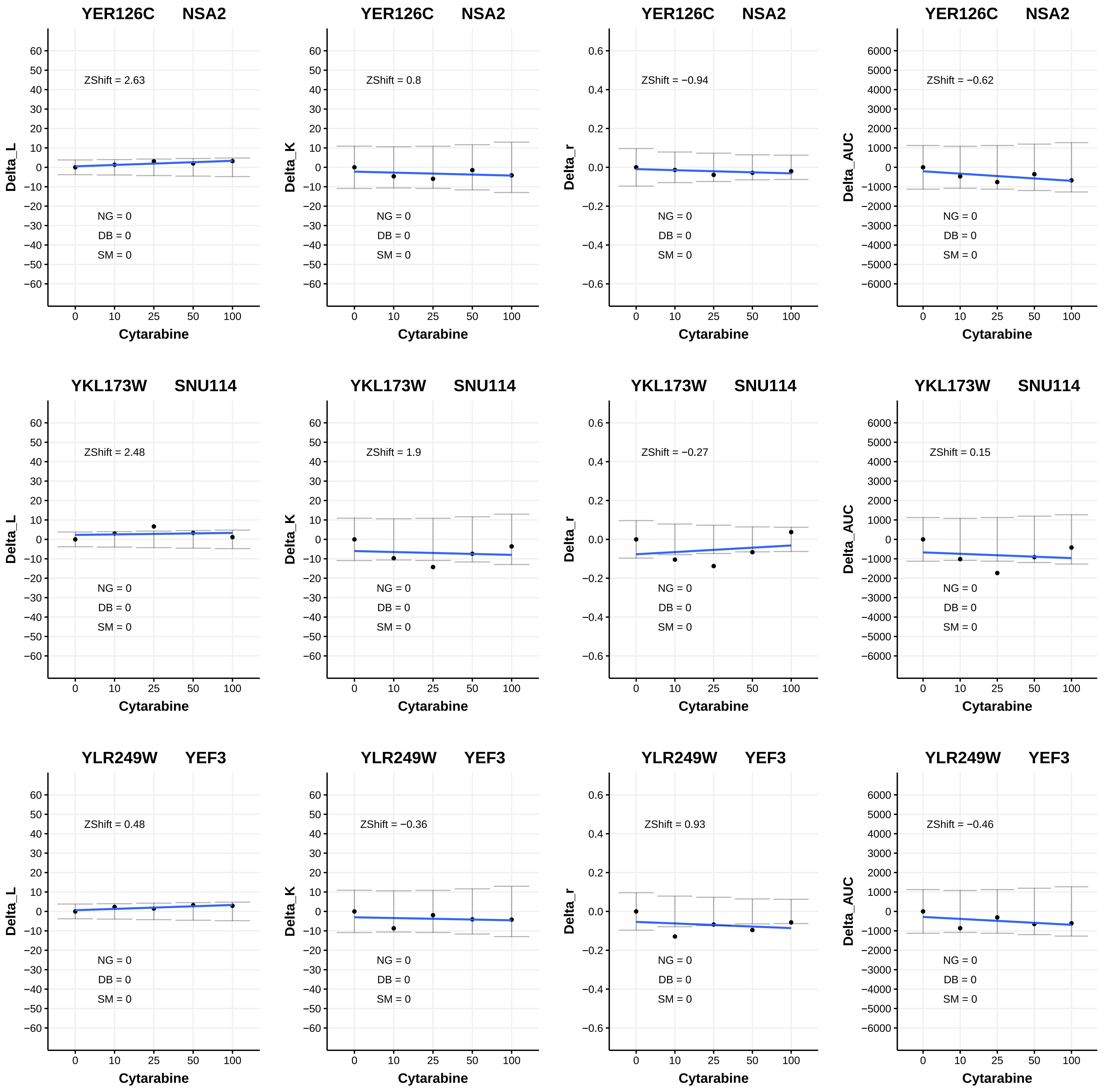
YER126C NSA2
ZShift = 2.63
ZShift = 0.8
ZShift = −0.94
ZShift = −0.62
NG = 0
DB = 0
SM = 0
Delta_L
Delta_K
Delta_r
Delta_AUC
Cytarabine
YKL173W SNU114
ZShift = 2.48
ZShift = 1.9
ZShift = −0.27
ZShift = 0.15
YLR249W YEF3
ZShift = 0.48
ZShift = −0.36
ZShift = 0.93
ZShift = −0.46

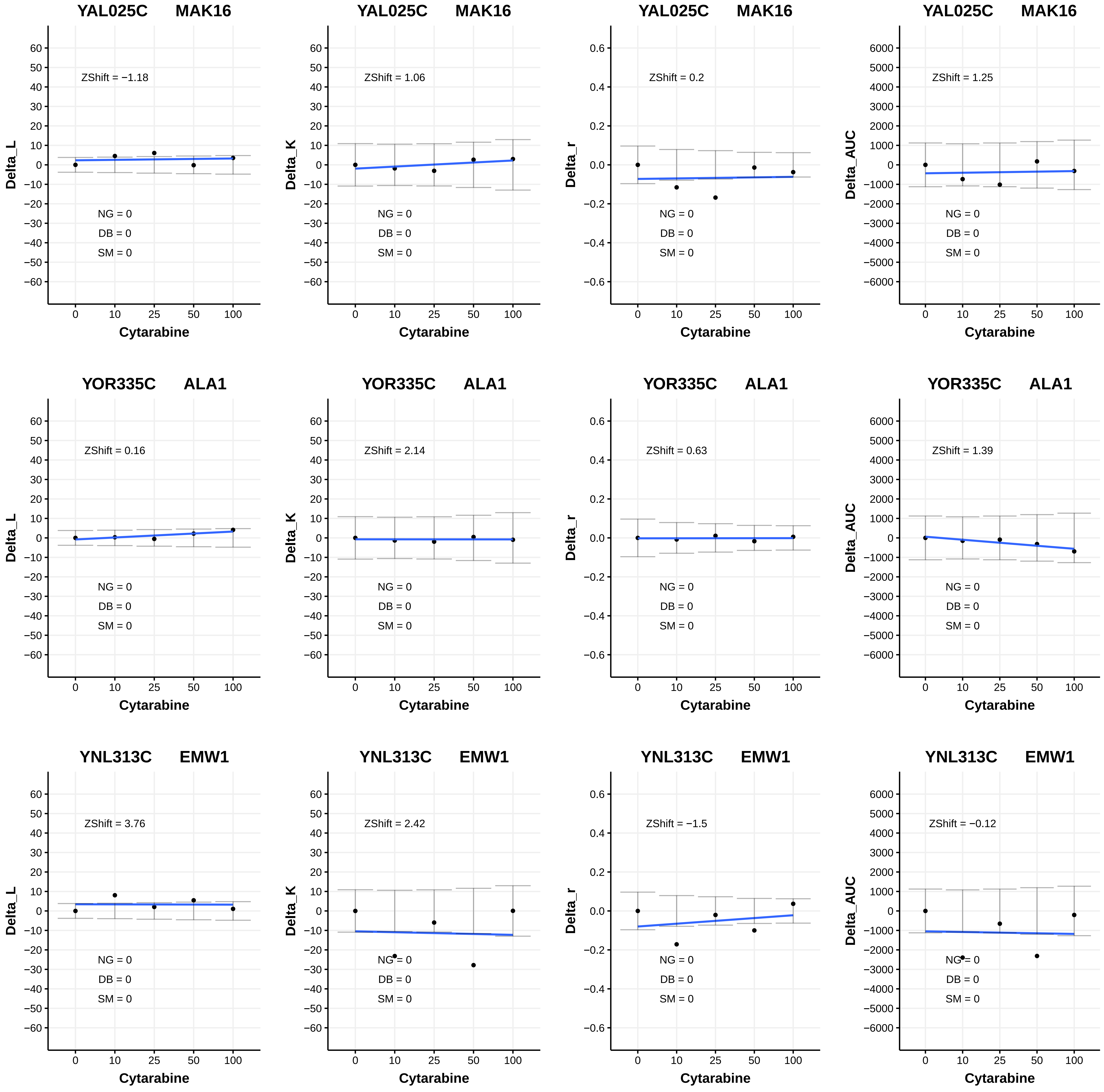
YAL025C MAK16
ZShift = −1.18
Delta_L
Cytarabine
NG = 0
DB = 0
SM = 0
YAL025C MAK16
ZShift = 1.06
Delta_K
Cytarabine
NG = 0
DB = 0
SM = 0
YAL025C MAK16
ZShift = 0.2
Delta_r
Cytarabine
NG = 0
DB = 0
SM = 0
YAL025C MAK16
ZShift = 1.25
Delta_AUC
Cytarabine
NG = 0
DB = 0
SM = 0
YOR335C ALA1
ZShift = 0.16
Delta_L
Cytarabine
NG = 0
DB = 0
SM = 0
YOR335C ALA1
ZShift = 2.14
Delta_K
Cytarabine
NG = 0
DB = 0
SM = 0
YOR335C ALA1
ZShift = 0.63
Delta_r
Cytarabine
NG = 0
DB = 0
SM = 0
YOR335C ALA1
ZShift = 1.39
Delta_AUC
Cytarabine
NG = 0
DB = 0
SM = 0
YNL313C EMW1
ZShift = 3.76
Delta_L
Cytarabine
NG = 0
DB = 0
SM = 0
YNL313C EMW1
ZShift = 2.42
Delta_K
Cytarabine
NG = 0
DB = 0
SM = 0
YNL313C EMW1
ZShift = −1.5
Delta_r
Cytarabine
NG = 0
DB = 0
SM = 0
YNL313C EMW1
ZShift = −0.12
Delta_AUC
Cytarabine
NG = 0
DB = 0
SM = 0

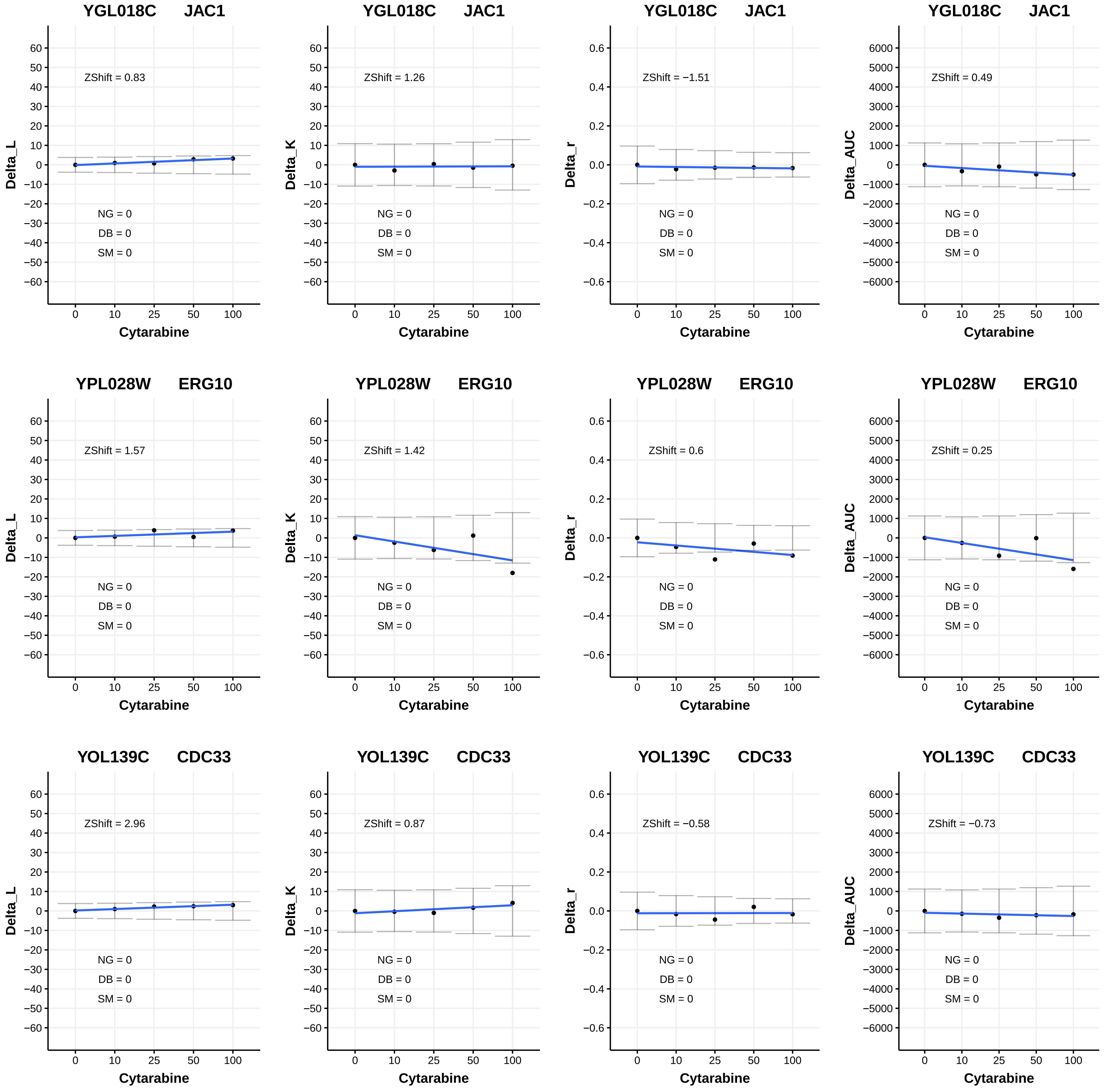
YGL018C JAC1
ZShift = 0.83
ZShift = 1.26
ZShift = −1.51
ZShift = 0.49
YPL028W ERG10
ZShift = 1.57
ZShift = 1.42
ZShift = 0.6
ZShift = 0.25
YOL139C CDC33
ZShift = 2.96
ZShift = 0.87
ZShift = −0.58
ZShift = −0.73
NG = 0
DB = 0
SM = 0
Delta_L
Delta_K
Delta_r
Delta_AUC
Cytarabine

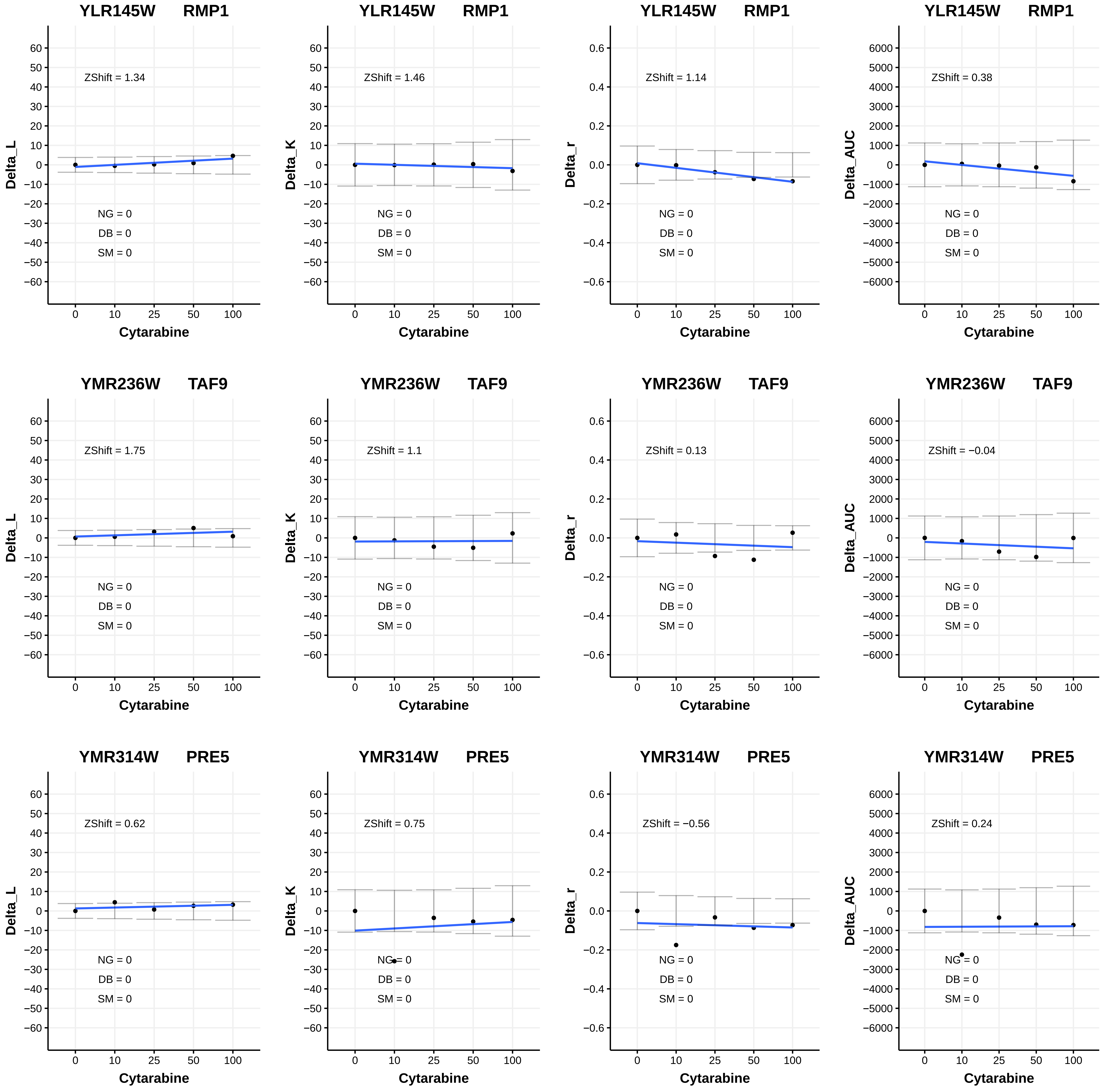

YLR145W RMP1
ZShift = 1.34
NG = 0
DB = 0
SM = 0
Delta_L
Cytarabine
YLR145W RMP1
ZShift = 1.46
NG = 0
DB = 0
SM = 0
Delta_K
Cytarabine
YLR145W RMP1
ZShift = 1.14
NG = 0
DB = 0
SM = 0
Delta_r
Cytarabine
YLR145W RMP1
ZShift = 0.38
NG = 0
DB = 0
SM = 0
Delta_AUC
Cytarabine
YMR236W TAF9
ZShift = 1.75
NG = 0
DB = 0
SM = 0
Delta_L
Cytarabine
YMR236W TAF9
ZShift = 1.1
NG = 0
DB = 0
SM = 0
Delta_K
Cytarabine
YMR236W TAF9
ZShift = 0.13
NG = 0
DB = 0
SM = 0
Delta_r
Cytarabine
YMR236W TAF9
ZShift = −0.04
NG = 0
DB = 0
SM = 0
Delta_AUC
Cytarabine
YMR314W PRE5
ZShift = 0.62
NG = 0
DB = 0
SM = 0
Delta_L
Cytarabine
YMR314W PRE5
ZShift = 0.75
NG = 0
DB = 0
SM = 0
Delta_K
Cytarabine
YMR314W PRE5
ZShift = −0.56
NG = 0
DB = 0
SM = 0
Delta_r
Cytarabine
YMR314W PRE5
ZShift = 0.24
NG = 0
DB = 0
SM = 0
Delta_AUC
Cytarabine

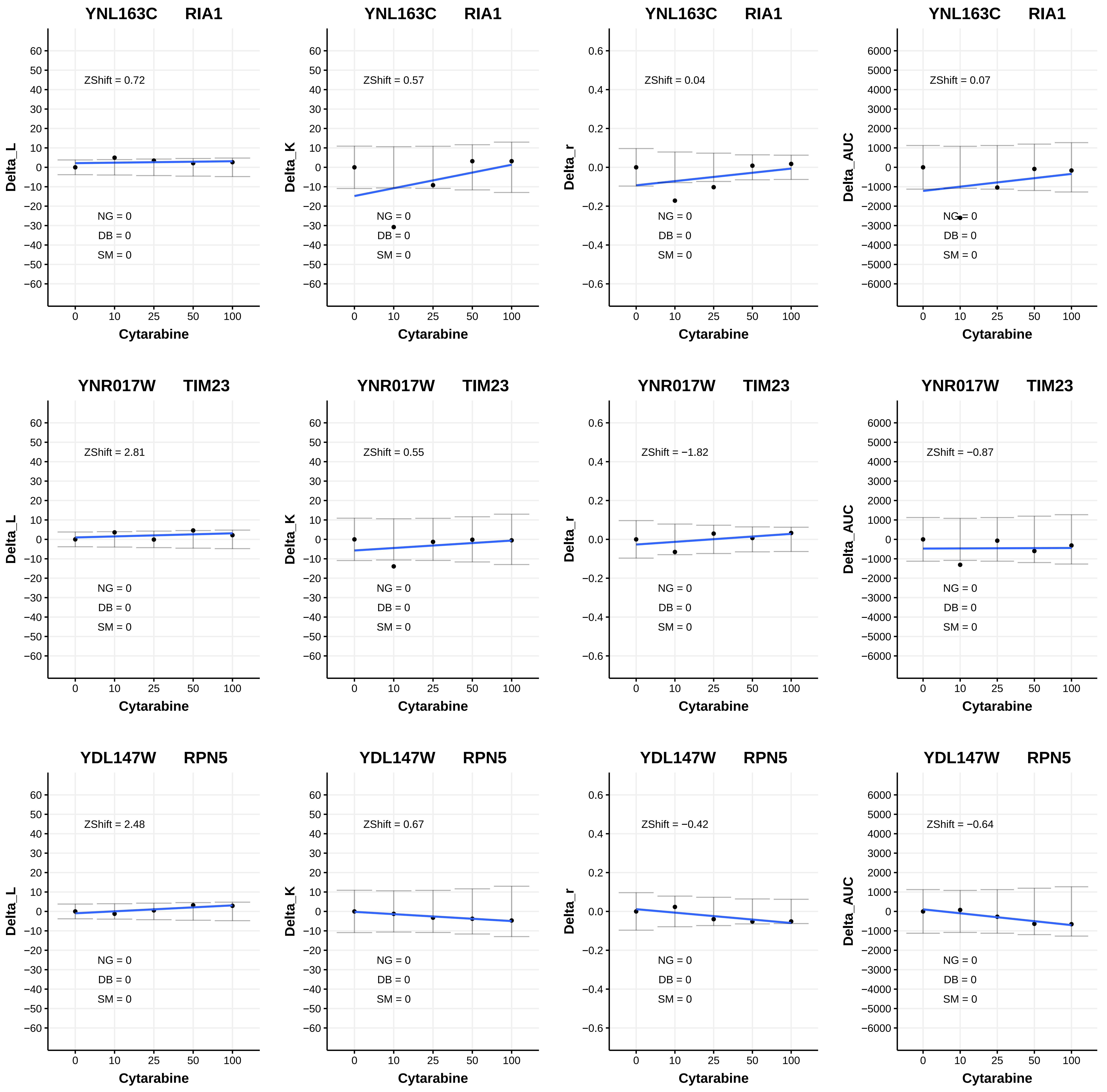
YNL163C RIA1
ZShift = 0.72
ZShift = 0.57
ZShift = 0.04
ZShift = 0.07
YNR017W TIM23
ZShift = 2.81
ZShift = 0.55
ZShift = −1.82
ZShift = −0.87
YDL147W RPN5
ZShift = 2.48
ZShift = 0.67
ZShift = −0.42
ZShift = −0.64
NG = 0
DB = 0
SM = 0
Delta_L
Delta_K
Delta_r
Delta_AUC
Cytarabine
0
10
25
50
100

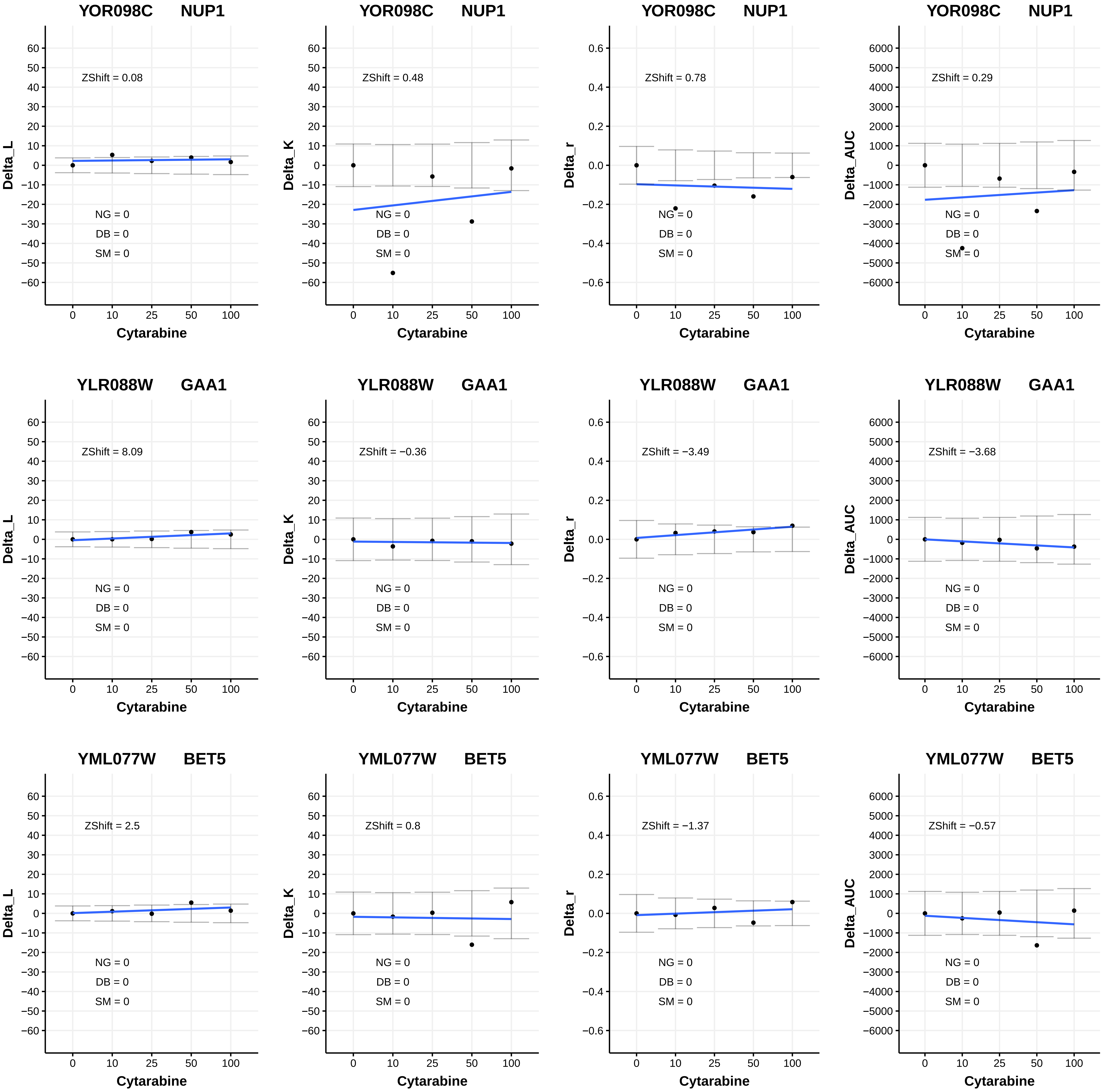
YOR098C NUP1
ZShift = 0.08
NG = 0
DB = 0
SM = 0
Delta_L
Cytarabine
YOR098C NUP1
ZShift = 0.48
NG = 0
DB = 0
SM = 0
Delta_K
Cytarabine
YOR098C NUP1
ZShift = 0.78
NG = 0
DB = 0
SM = 0
Delta_r
Cytarabine
YOR098C NUP1
ZShift = 0.29
NG = 0
DB = 0
SM = 0
Delta_AUC
Cytarabine
YLR088W GAA1
ZShift = 8.09
NG = 0
DB = 0
SM = 0
Delta_L
Cytarabine
YLR088W GAA1
ZShift = −0.36
NG = 0
DB = 0
SM = 0
Delta_K
Cytarabine
YLR088W GAA1
ZShift = −3.49
NG = 0
DB = 0
SM = 0
Delta_r
Cytarabine
YLR088W GAA1
ZShift = −3.68
NG = 0
DB = 0
SM = 0
Delta_AUC
Cytarabine
YML077W BET5
ZShift = 2.5
NG = 0
DB = 0
SM = 0
Delta_L
Cytarabine
YML077W BET5
ZShift = 0.8
NG = 0
DB = 0
SM = 0
Delta_K
Cytarabine
YML077W BET5
ZShift = −1.37
NG = 0
DB = 0
SM = 0
Delta_r
Cytarabine
YML077W BET5
ZShift = −0.57
NG = 0
DB = 0
SM = 0
Delta_AUC
Cytarabine

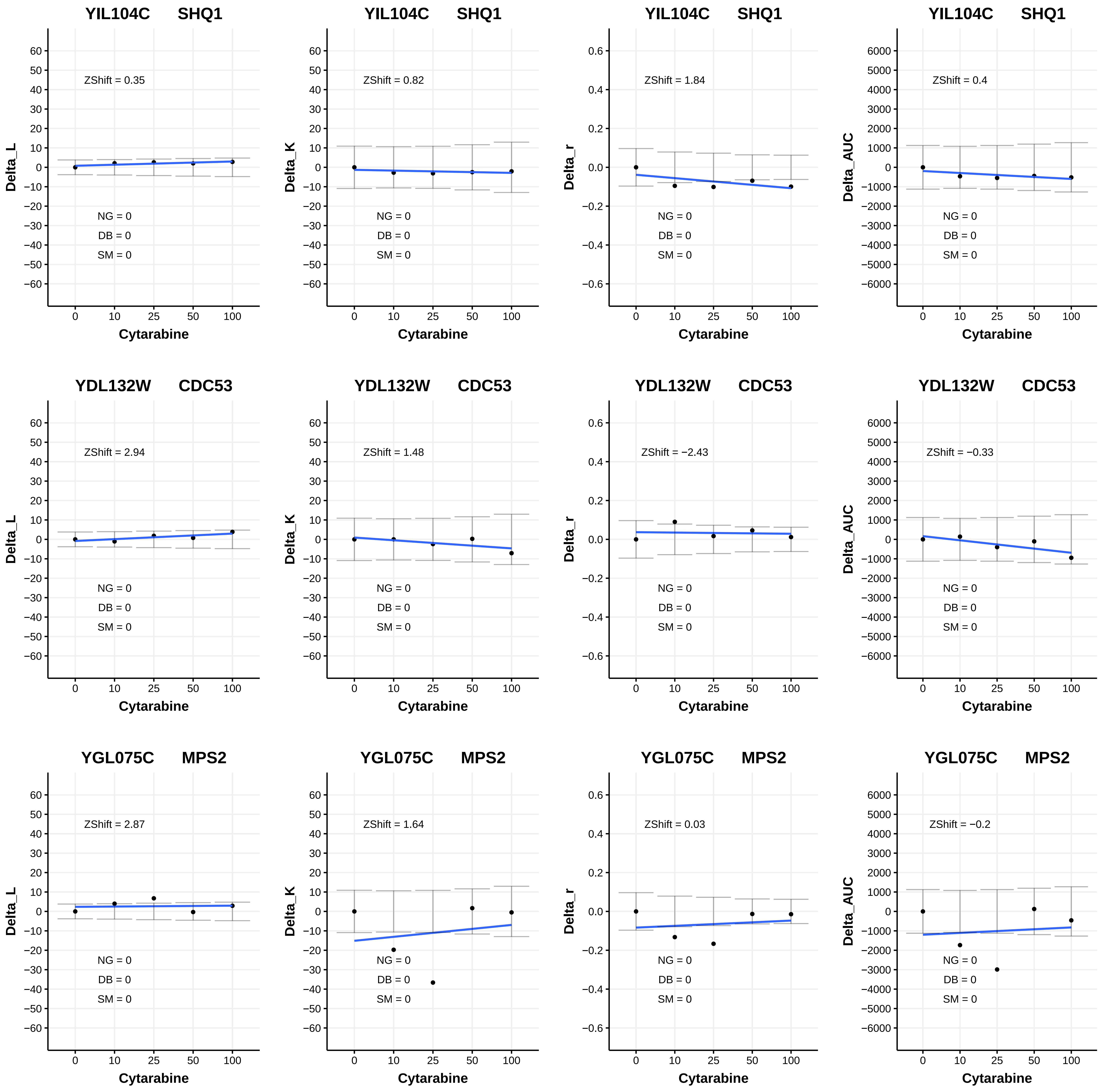

YIL104C SHQ1
ZShift = 0.35
NG = 0
DB = 0
SM = 0
Delta_L
Cytarabine
YIL104C SHQ1
ZShift = 0.82
NG = 0
DB = 0
SM = 0
Delta_K
Cytarabine
YIL104C SHQ1
ZShift = 1.84
NG = 0
DB = 0
SM = 0
Delta_r
Cytarabine
YIL104C SHQ1
ZShift = 0.4
NG = 0
DB = 0
SM = 0
Delta_AUC
Cytarabine
YDL132W CDC53
ZShift = 2.94
NG = 0
DB = 0
SM = 0
Delta_L
Cytarabine
YDL132W CDC53
ZShift = 1.48
NG = 0
DB = 0
SM = 0
Delta_K
Cytarabine
YDL132W CDC53
ZShift = −2.43
NG = 0
DB = 0
SM = 0
Delta_r
Cytarabine
YDL132W CDC53
ZShift = −0.33
NG = 0
DB = 0
SM = 0
Delta_AUC
Cytarabine
YGL075C MPS2
ZShift = 2.87
NG = 0
DB = 0
SM = 0
Delta_L
Cytarabine
YGL075C MPS2
ZShift = 1.64
NG = 0
DB = 0
SM = 0
Delta_K
Cytarabine
YGL075C MPS2
ZShift = 0.03
NG = 0
DB = 0
SM = 0
Delta_r
Cytarabine
YGL075C MPS2
ZShift = −0.2
NG = 0
DB = 0
SM = 0
Delta_AUC
Cytarabine

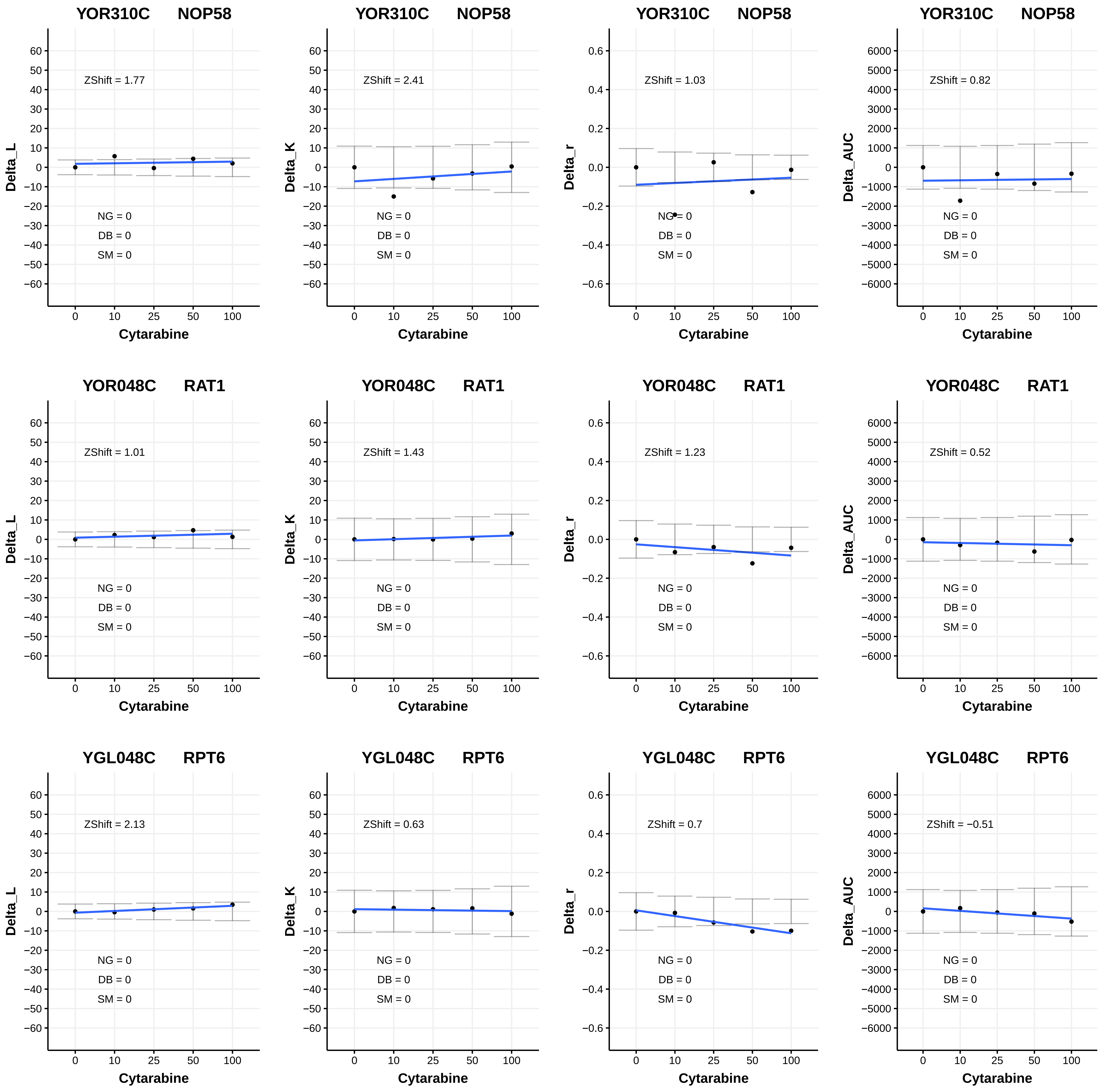
YOR310C NOP58
ZShift = 1.77
NG = 0
DB = 0
SM = 0
Delta_L
Cytarabine
YOR310C NOP58
ZShift = 2.41
NG = 0
DB = 0
SM = 0
Delta_K
Cytarabine
YOR310C NOP58
ZShift = 1.03
NG = 0
DB = 0
SM = 0
Delta_r
Cytarabine
YOR310C NOP58
ZShift = 0.82
NG = 0
DB = 0
SM = 0
Delta_AUC
Cytarabine
YOR048C RAT1
ZShift = 1.01
NG = 0
DB = 0
SM = 0
Delta_L
Cytarabine
YOR048C RAT1
ZShift = 1.43
NG = 0
DB = 0
SM = 0
Delta_K
Cytarabine
YOR048C RAT1
ZShift = 1.23
NG = 0
DB = 0
SM = 0
Delta_r
Cytarabine
YOR048C RAT1
ZShift = 0.52
NG = 0
DB = 0
SM = 0
Delta_AUC
Cytarabine
YGL048C RPT6
ZShift = 2.13
NG = 0
DB = 0
SM = 0
Delta_L
Cytarabine
YGL048C RPT6
ZShift = 0.63
NG = 0
DB = 0
SM = 0
Delta_K
Cytarabine
YGL048C RPT6
ZShift = 0.7
NG = 0
DB = 0
SM = 0
Delta_r
Cytarabine
YGL048C RPT6
ZShift = −0.51
NG = 0
DB = 0
SM = 0
Delta_AUC
Cytarabine

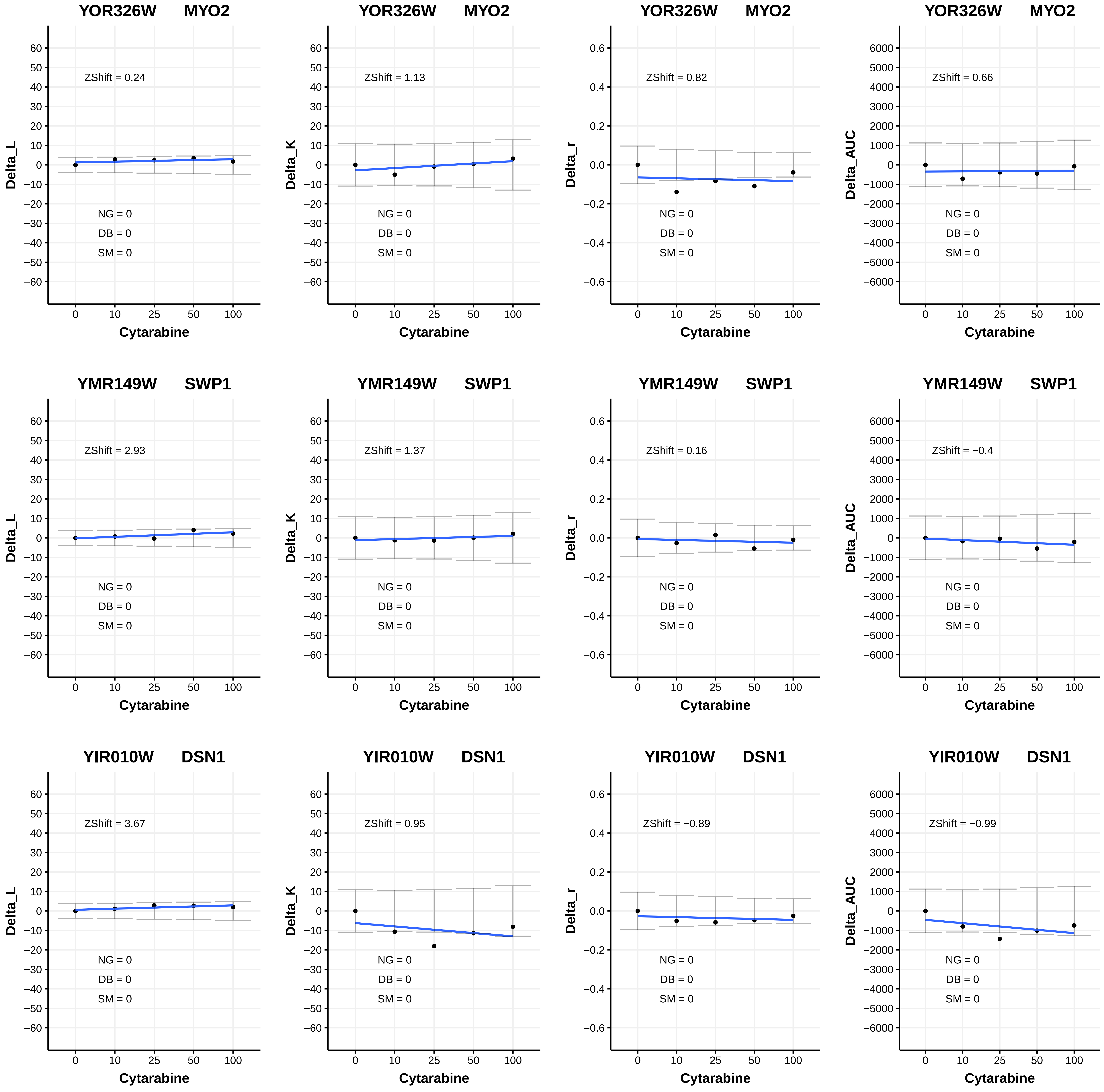
YOR326W MYO2
Delta_L
ZShift = 0.24
NG = 0
DB = 0
SM = 0
Cytarabine
YOR326W MYO2
Delta_K
ZShift = 1.13
NG = 0
DB = 0
SM = 0
Cytarabine
YOR326W MYO2
Delta_r
ZShift = 0.82
NG = 0
DB = 0
SM = 0
Cytarabine
YOR326W MYO2
Delta_AUC
ZShift = 0.66
NG = 0
DB = 0
SM = 0
Cytarabine
YMR149W SWP1
Delta_L
ZShift = 2.93
NG = 0
DB = 0
SM = 0
Cytarabine
YMR149W SWP1
Delta_K
ZShift = 1.37
NG = 0
DB = 0
SM = 0
Cytarabine
YMR149W SWP1
Delta_r
ZShift = 0.16
NG = 0
DB = 0
SM = 0
Cytarabine
YMR149W SWP1
Delta_AUC
ZShift = −0.4
NG = 0
DB = 0
SM = 0
Cytarabine
YIR010W DSN1
Delta_L
ZShift = 3.67
NG = 0
DB = 0
SM = 0
Cytarabine
YIR010W DSN1
Delta_K
ZShift = 0.95
NG = 0
DB = 0
SM = 0
Cytarabine
YIR010W DSN1
Delta_r
ZShift = −0.89
NG = 0
DB = 0
SM = 0
Cytarabine
YIR010W DSN1
Delta_AUC
ZShift = −0.99
NG = 0
DB = 0
SM = 0
Cytarabine

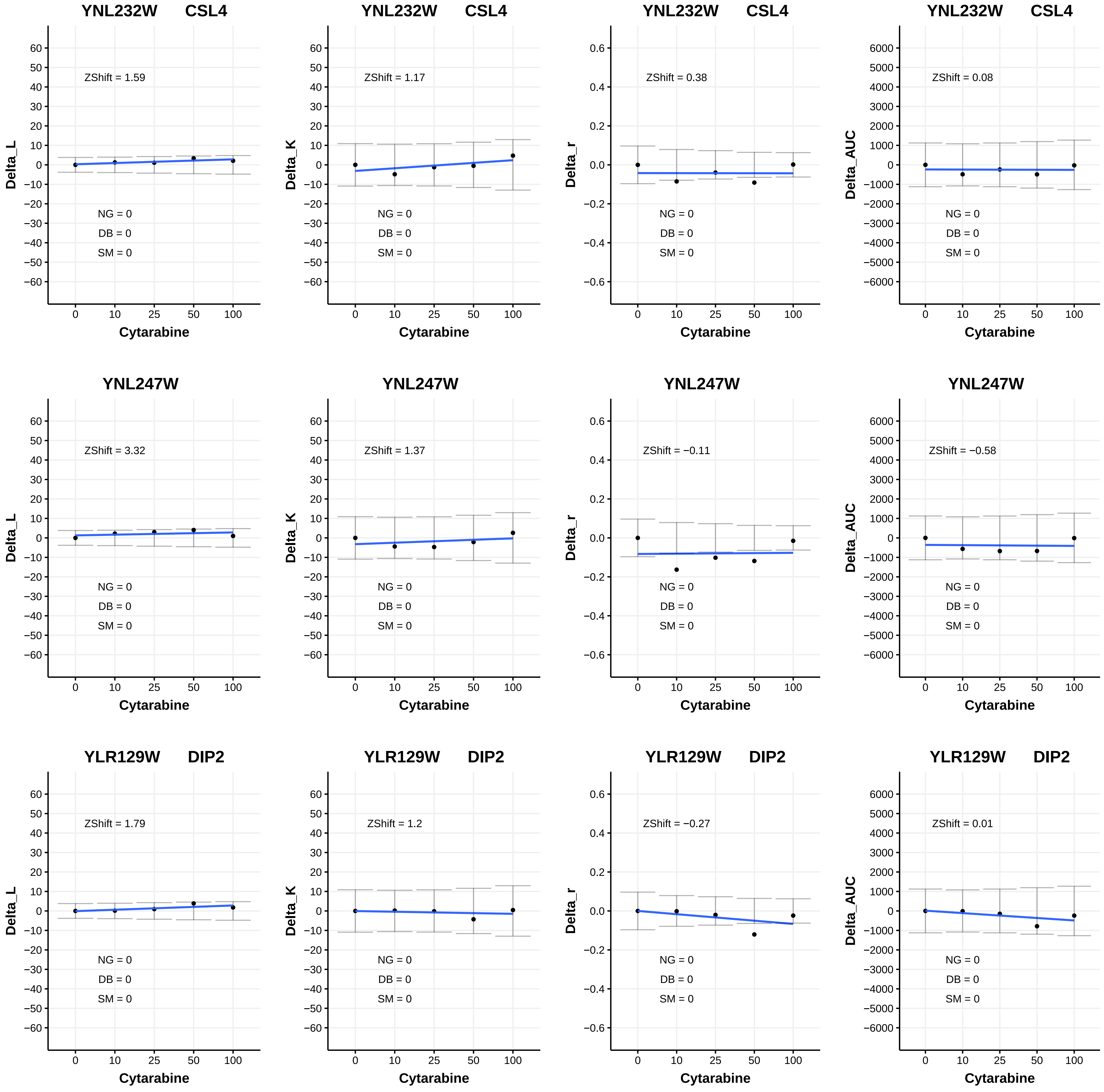

YNL232W CSL4
ZShift = 1.59
ZShift = 1.17
ZShift = 0.38
ZShift = 0.08
YNL247W
ZShift = 3.32
ZShift = 1.37
ZShift = −0.11
ZShift = −0.58
YLR129W DIP2
ZShift = 1.79
ZShift = 1.2
ZShift = −0.27
ZShift = 0.01
NG = 0
DB = 0
SM = 0
Delta_L
Delta_K
Delta_r
Delta_AUC
Cytarabine
0
10
25
50
100

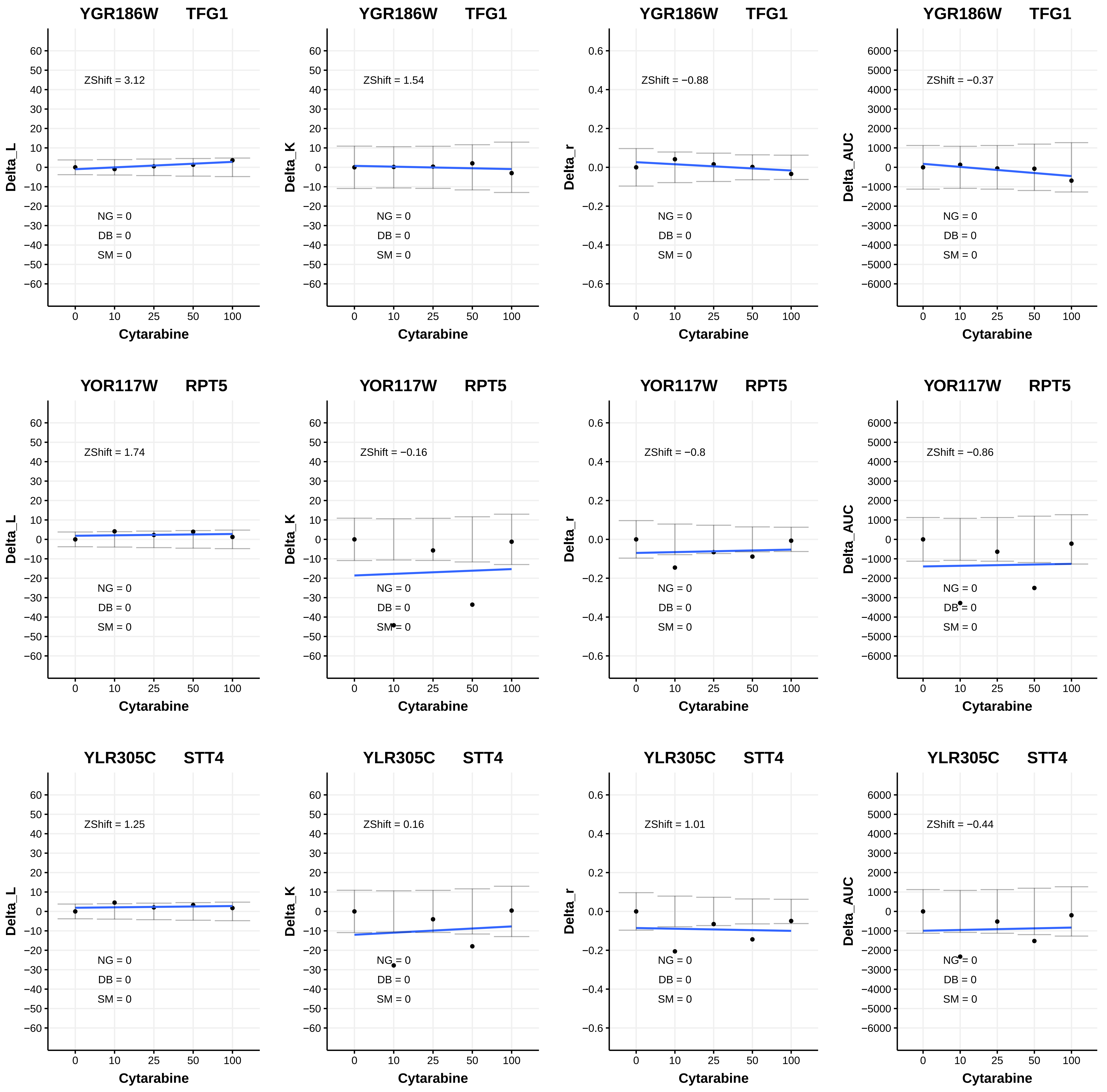

YGR186W TFG1
ZShift = 3.12
Delta_L
NG = 0
DB = 0
SM = 0
Cytarabine
YGR186W TFG1
ZShift = 1.54
Delta_K
NG = 0
DB = 0
SM = 0
Cytarabine
YGR186W TFG1
ZShift = −0.88
Delta_r
NG = 0
DB = 0
SM = 0
Cytarabine
YGR186W TFG1
ZShift = −0.37
Delta_AUC
NG = 0
DB = 0
SM = 0
Cytarabine
YOR117W RPT5
ZShift = 1.74
Delta_L
NG = 0
DB = 0
SM = 0
Cytarabine
YOR117W RPT5
ZShift = −0.16
Delta_K
NG = 0
DB = 0
SM = 0
Cytarabine
YOR117W RPT5
ZShift = −0.8
Delta_r
NG = 0
DB = 0
SM = 0
Cytarabine
YOR117W RPT5
ZShift = −0.86
Delta_AUC
NG = 0
DB = 0
SM = 0
Cytarabine
YLR305C STT4
ZShift = 1.25
Delta_L
NG = 0
DB = 0
SM = 0
Cytarabine
YLR305C STT4
ZShift = 0.16
Delta_K
NG = 0
DB = 0
SM = 0
Cytarabine
YLR305C STT4
ZShift = 1.01
Delta_r
NG = 0
DB = 0
SM = 0
Cytarabine
YLR305C STT4
ZShift = −0.44
Delta_AUC
NG = 0
DB = 0
SM = 0
Cytarabine

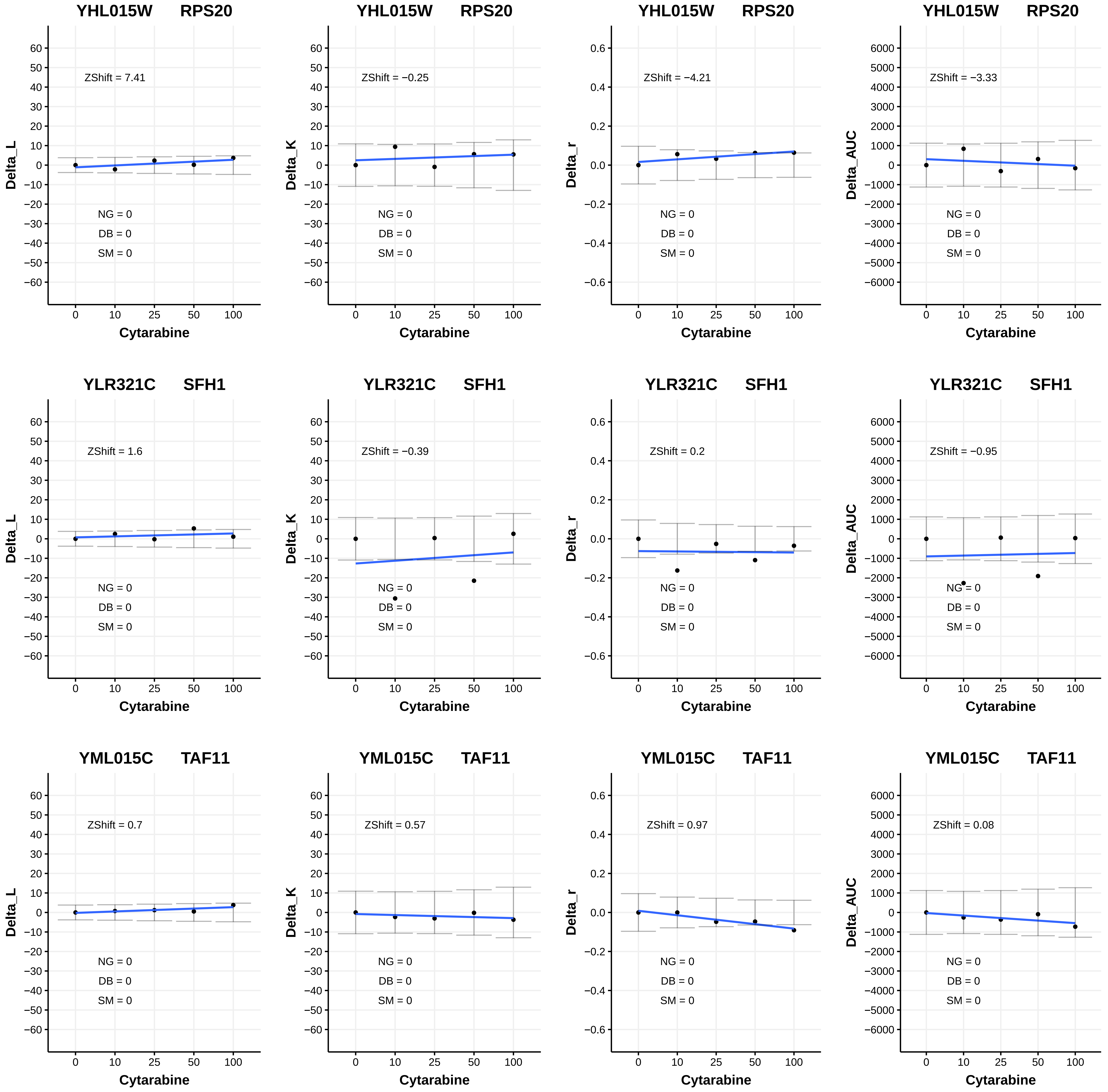
YHL015W RPS20
ZShift = 7.41
NG = 0
DB = 0
SM = 0
Delta_L
Cytarabine
YHL015W RPS20
ZShift = −0.25
NG = 0
DB = 0
SM = 0
Delta_K
Cytarabine
YHL015W RPS20
ZShift = −4.21
NG = 0
DB = 0
SM = 0
Delta_r
Cytarabine
YHL015W RPS20
ZShift = −3.33
NG = 0
DB = 0
SM = 0
Delta_AUC
Cytarabine
YLR321C SFH1
ZShift = 1.6
NG = 0
DB = 0
SM = 0
Delta_L
Cytarabine
YLR321C SFH1
ZShift = −0.39
NG = 0
DB = 0
SM = 0
Delta_K
Cytarabine
YLR321C SFH1
ZShift = 0.2
NG = 0
DB = 0
SM = 0
Delta_r
Cytarabine
YLR321C SFH1
ZShift = −0.95
NG = 0
DB = 0
SM = 0
Delta_AUC
Cytarabine
YML015C TAF11
ZShift = 0.7
NG = 0
DB = 0
SM = 0
Delta_L
Cytarabine
YML015C TAF11
ZShift = 0.57
NG = 0
DB = 0
SM = 0
Delta_K
Cytarabine
YML015C TAF11
ZShift = 0.97
NG = 0
DB = 0
SM = 0
Delta_r
Cytarabine
YML015C TAF11
ZShift = 0.08
NG = 0
DB = 0
SM = 0
Delta_AUC
Cytarabine
0 10 25 50 100

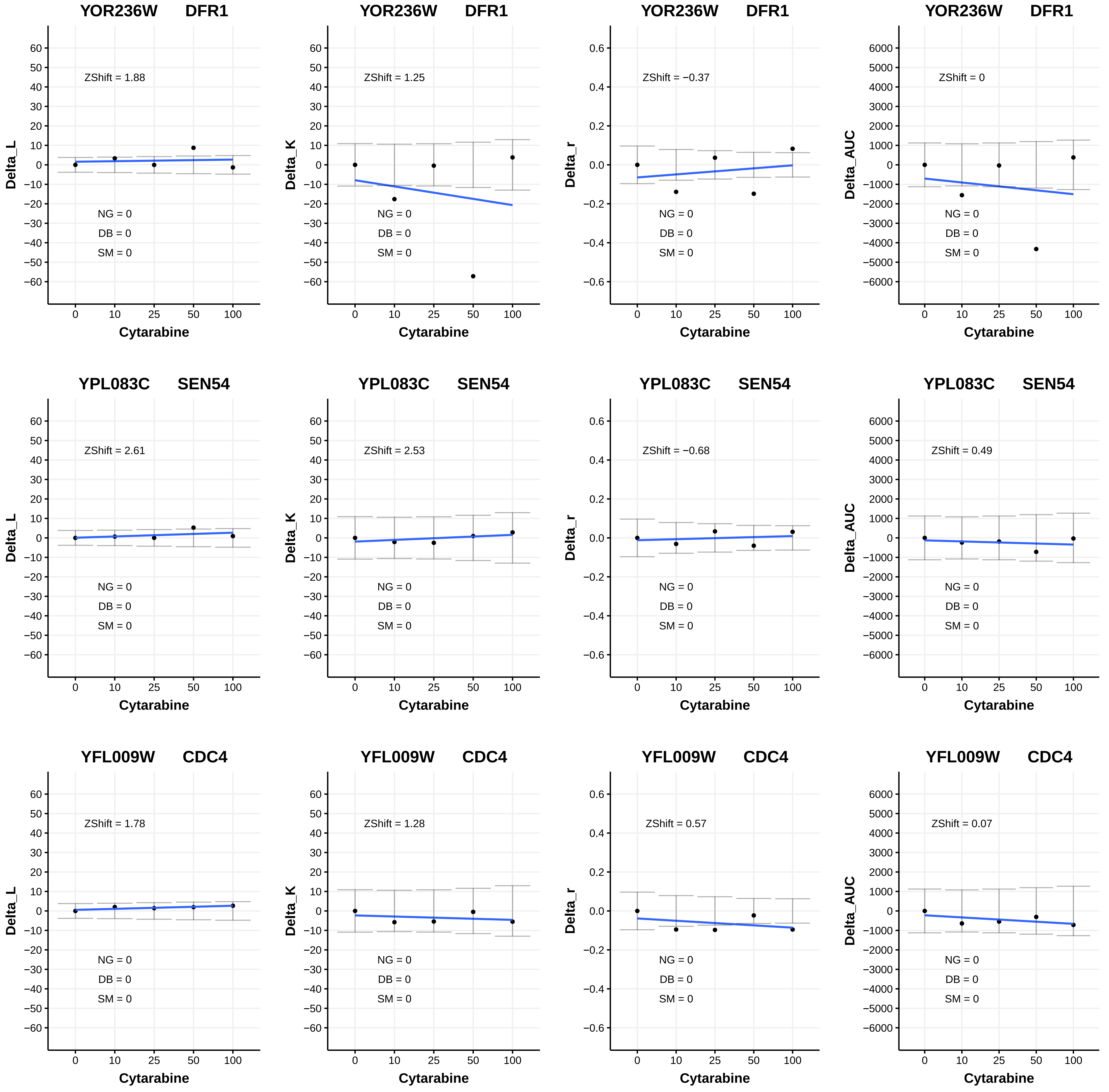
YOR236W DFR1
Delta_L
ZShift = 1.88
NG = 0
DB = 0
SM = 0
Cytarabine
YOR236W DFR1
Delta_K
ZShift = 1.25
NG = 0
DB = 0
SM = 0
Cytarabine
YOR236W DFR1
Delta_r
ZShift = −0.37
NG = 0
DB = 0
SM = 0
Cytarabine
YOR236W DFR1
Delta_AUC
ZShift = 0
NG = 0
DB = 0
SM = 0
Cytarabine
YPL083C SEN54
Delta_L
ZShift = 2.61
NG = 0
DB = 0
SM = 0
Cytarabine
YPL083C SEN54
Delta_K
ZShift = 2.53
NG = 0
DB = 0
SM = 0
Cytarabine
YPL083C SEN54
Delta_r
ZShift = −0.68
NG = 0
DB = 0
SM = 0
Cytarabine
YPL083C SEN54
Delta_AUC
ZShift = 0.49
NG = 0
DB = 0
SM = 0
Cytarabine
YFL009W CDC4
Delta_L
ZShift = 1.78
NG = 0
DB = 0
SM = 0
Cytarabine
YFL009W CDC4
Delta_K
ZShift = 1.28
NG = 0
DB = 0
SM = 0
Cytarabine
YFL009W CDC4
Delta_r
ZShift = 0.57
NG = 0
DB = 0
SM = 0
Cytarabine
YFL009W CDC4
Delta_AUC
ZShift = 0.07
NG = 0
DB = 0
SM = 0
Cytarabine

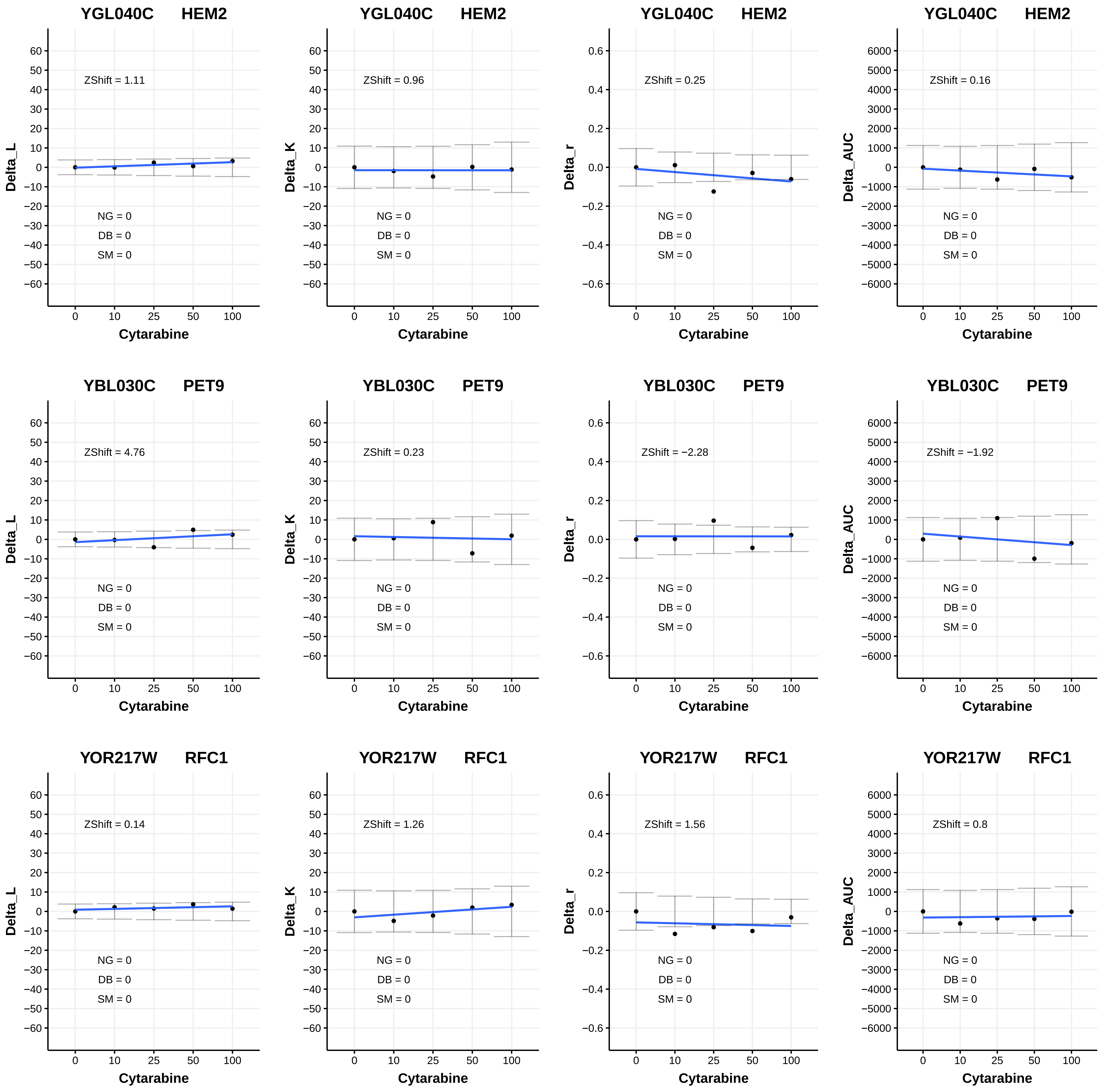

YGL040C HEM2
ZShift = 1.11
NG = 0
DB = 0
SM = 0
Delta_L
Cytarabine
YGL040C HEM2
ZShift = 0.96
NG = 0
DB = 0
SM = 0
Delta_K
Cytarabine
YGL040C HEM2
ZShift = 0.25
NG = 0
DB = 0
SM = 0
Delta_r
Cytarabine
YGL040C HEM2
ZShift = 0.16
NG = 0
DB = 0
SM = 0
Delta_AUC
Cytarabine
YBL030C PET9
ZShift = 4.76
NG = 0
DB = 0
SM = 0
Delta_L
Cytarabine
YBL030C PET9
ZShift = 0.23
NG = 0
DB = 0
SM = 0
Delta_K
Cytarabine
YBL030C PET9
ZShift = −2.28
NG = 0
DB = 0
SM = 0
Delta_r
Cytarabine
YBL030C PET9
ZShift = −1.92
NG = 0
DB = 0
SM = 0
Delta_AUC
Cytarabine
YOR217W RFC1
ZShift = 0.14
NG = 0
DB = 0
SM = 0
Delta_L
Cytarabine
YOR217W RFC1
ZShift = 1.26
NG = 0
DB = 0
SM = 0
Delta_K
Cytarabine
YOR217W RFC1
ZShift = 1.56
NG = 0
DB = 0
SM = 0
Delta_r
Cytarabine
YOR217W RFC1
ZShift = 0.8
NG = 0
DB = 0
SM = 0
Delta_AUC
Cytarabine

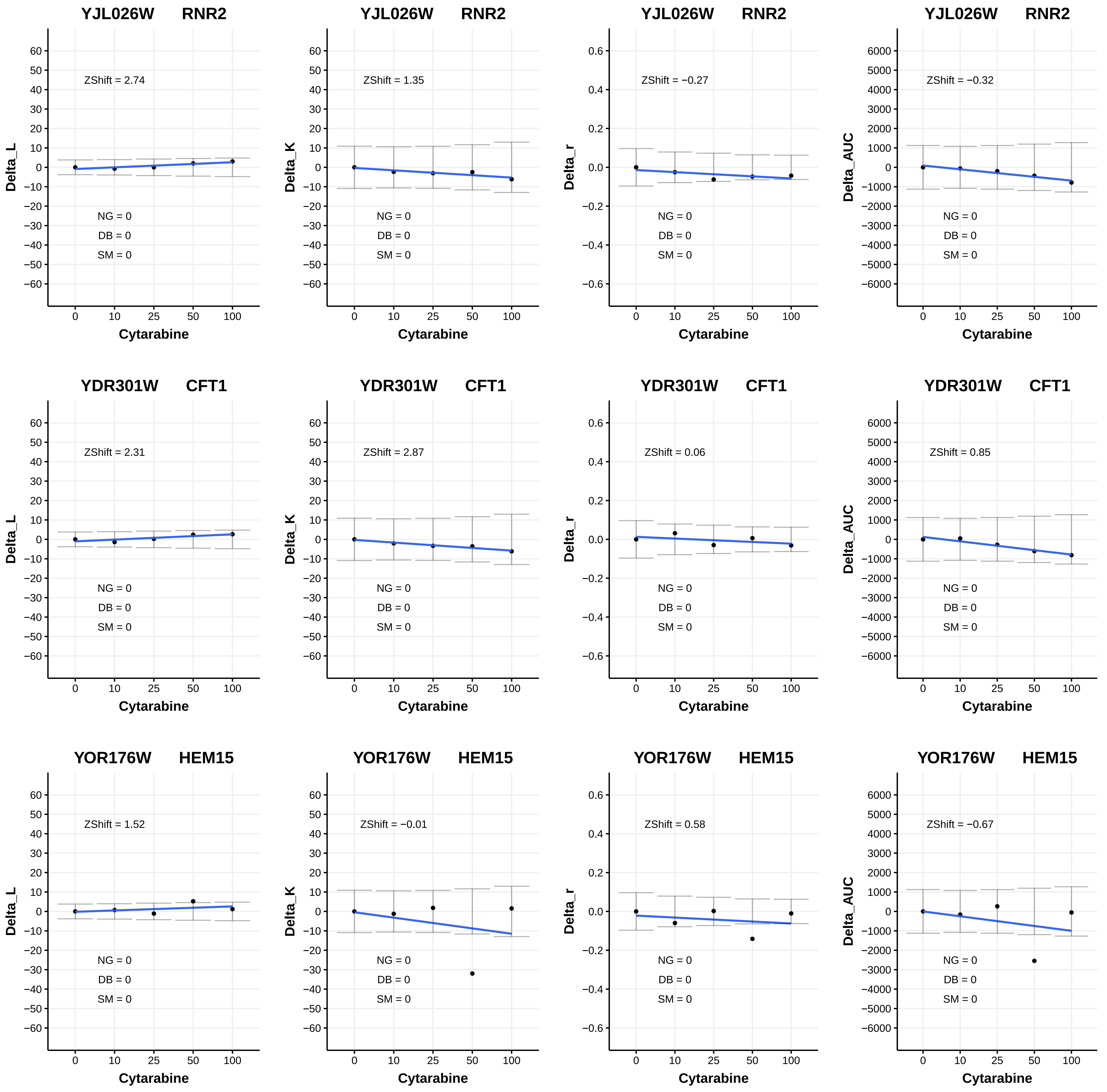
YJL026W RNR2
ZShift = 2.74
NG = 0
DB = 0
SM = 0
Delta_L
Cytarabine
YJL026W RNR2
ZShift = 1.35
NG = 0
DB = 0
SM = 0
Delta_K
Cytarabine
YJL026W RNR2
ZShift = −0.27
NG = 0
DB = 0
SM = 0
Delta_r
Cytarabine
YJL026W RNR2
ZShift = −0.32
NG = 0
DB = 0
SM = 0
Delta_AUC
Cytarabine
YDR301W CFT1
ZShift = 2.31
NG = 0
DB = 0
SM = 0
Delta_L
Cytarabine
YDR301W CFT1
ZShift = 2.87
NG = 0
DB = 0
SM = 0
Delta_K
Cytarabine
YDR301W CFT1
ZShift = 0.06
NG = 0
DB = 0
SM = 0
Delta_r
Cytarabine
YDR301W CFT1
ZShift = 0.85
NG = 0
DB = 0
SM = 0
Delta_AUC
Cytarabine
YOR176W HEM15
ZShift = 1.52
NG = 0
DB = 0
SM = 0
Delta_L
Cytarabine
YOR176W HEM15
ZShift = −0.01
NG = 0
DB = 0
SM = 0
Delta_K
Cytarabine
YOR176W HEM15
ZShift = 0.58
NG = 0
DB = 0
SM = 0
Delta_r
Cytarabine
YOR176W HEM15
ZShift = −0.67
NG = 0
DB = 0
SM = 0
Delta_AUC
Cytarabine

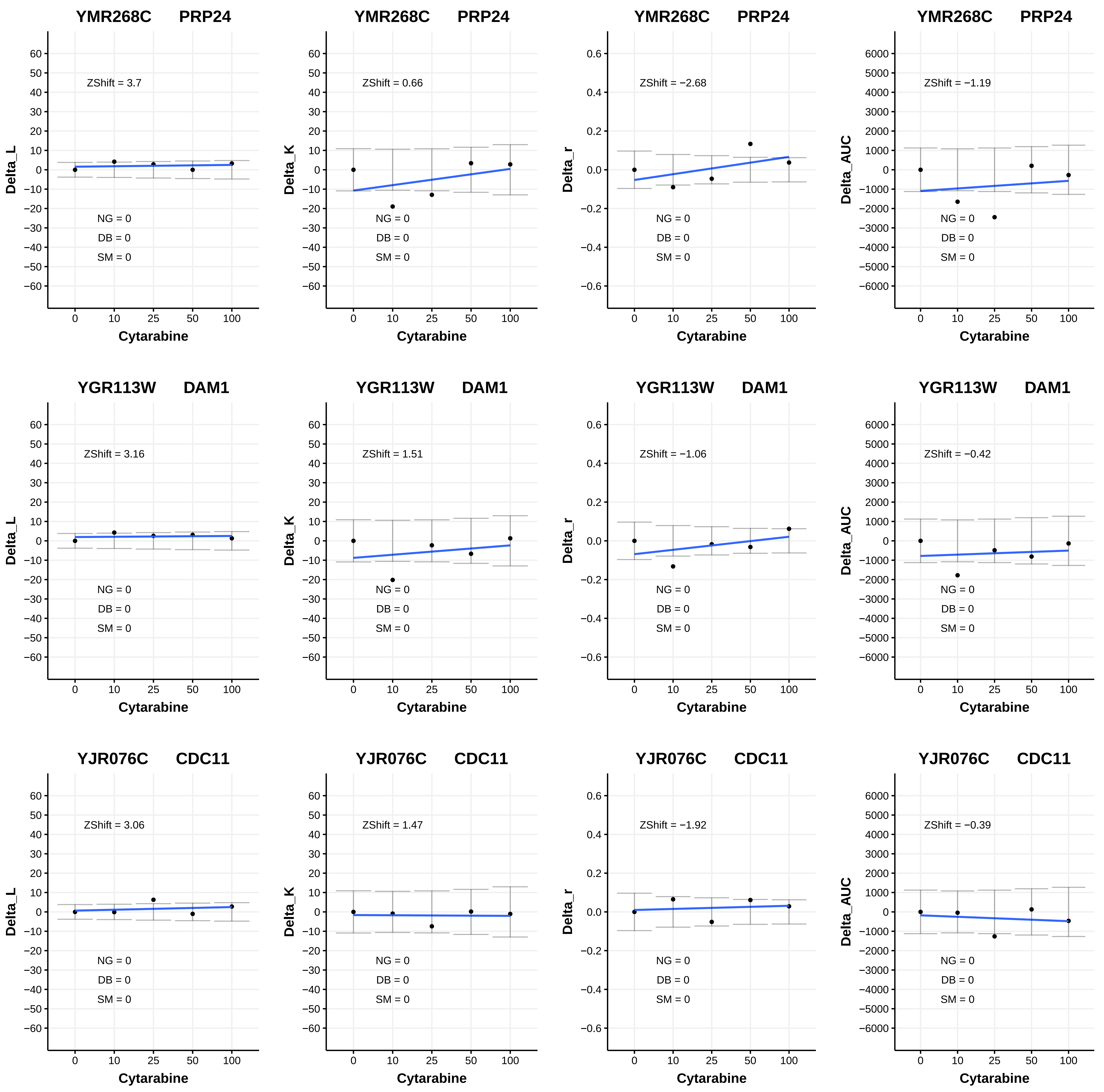
YMR268C PRP24
ZShift = 3.7
NG = 0
DB = 0
SM = 0
Delta_L
Cytarabine
YMR268C PRP24
ZShift = 0.66
NG = 0
DB = 0
SM = 0
Delta_K
Cytarabine
YMR268C PRP24
ZShift = −2.68
NG = 0
DB = 0
SM = 0
Delta_r
Cytarabine
YMR268C PRP24
ZShift = −1.19
NG = 0
DB = 0
SM = 0
Delta_AUC
Cytarabine
YGR113W DAM1
ZShift = 3.16
NG = 0
DB = 0
SM = 0
Delta_L
Cytarabine
YGR113W DAM1
ZShift = 1.51
NG = 0
DB = 0
SM = 0
Delta_K
Cytarabine
YGR113W DAM1
ZShift = −1.06
NG = 0
DB = 0
SM = 0
Delta_r
Cytarabine
YGR113W DAM1
ZShift = −0.42
NG = 0
DB = 0
SM = 0
Delta_AUC
Cytarabine
YJR076C CDC11
ZShift = 3.06
NG = 0
DB = 0
SM = 0
Delta_L
Cytarabine
YJR076C CDC11
ZShift = 1.47
NG = 0
DB = 0
SM = 0
Delta_K
Cytarabine
YJR076C CDC11
ZShift = −1.92
NG = 0
DB = 0
SM = 0
Delta_r
Cytarabine
YJR076C CDC11
ZShift = −0.39
NG = 0
DB = 0
SM = 0
Delta_AUC
Cytarabine

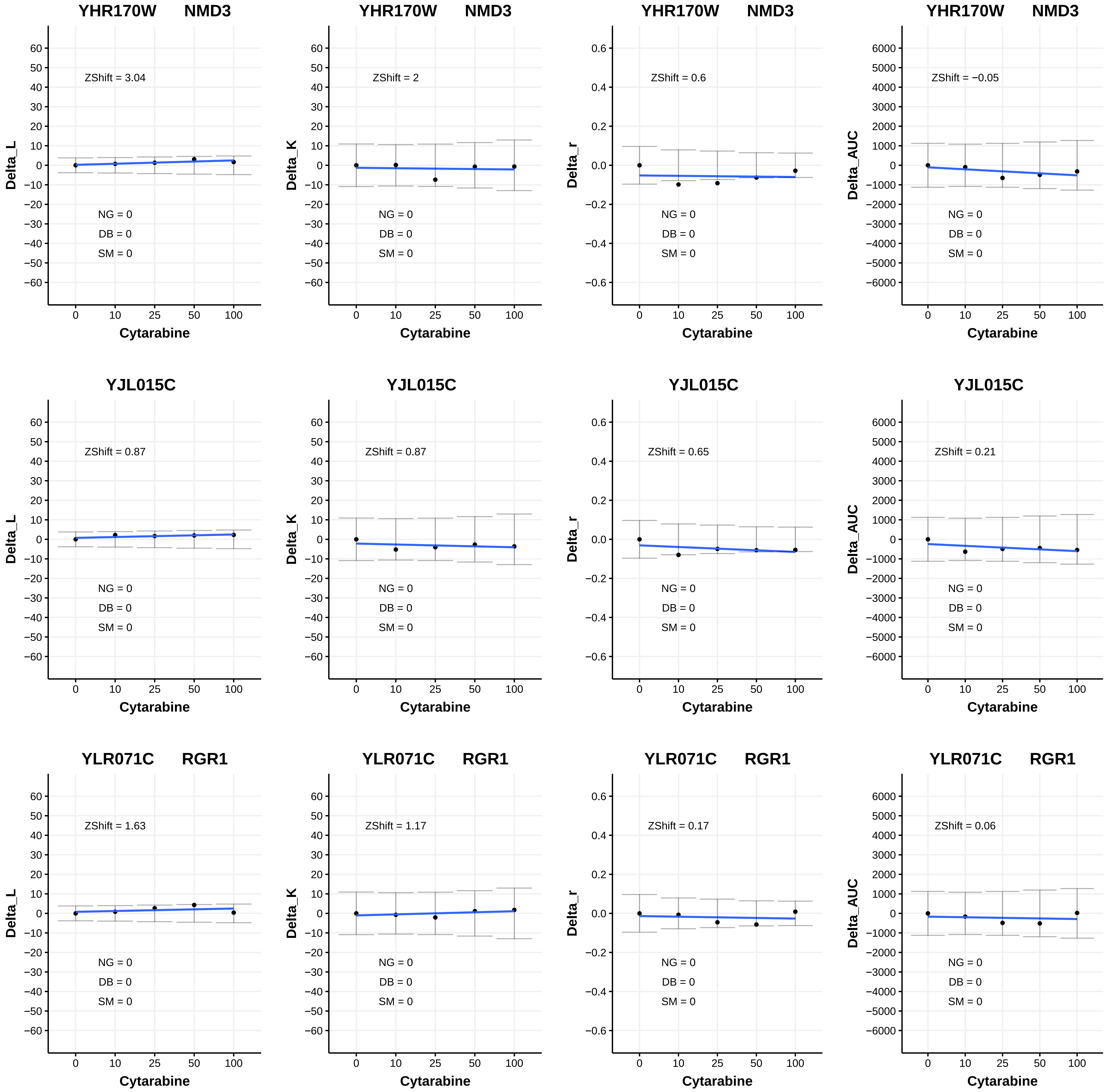
YHR170W NMD3
ZShift = 3.04
Delta_L
NG = 0
DB = 0
SM = 0
Cytarabine
YHR170W NMD3
ZShift = 2
Delta_K
NG = 0
DB = 0
SM = 0
Cytarabine
YHR170W NMD3
ZShift = 0.6
Delta_r
NG = 0
DB = 0
SM = 0
Cytarabine
YHR170W NMD3
ZShift = −0.05
Delta_AUC
NG = 0
DB = 0
SM = 0
Cytarabine
YJL015C
ZShift = 0.87
Delta_L
NG = 0
DB = 0
SM = 0
Cytarabine
YJL015C
ZShift = 0.87
Delta_K
NG = 0
DB = 0
SM = 0
Cytarabine
YJL015C
ZShift = 0.65
Delta_r
NG = 0
DB = 0
SM = 0
Cytarabine
YJL015C
ZShift = 0.21
Delta_AUC
NG = 0
DB = 0
SM = 0
Cytarabine
YLR071C RGR1
ZShift = 1.63
Delta_L
NG = 0
DB = 0
SM = 0
Cytarabine
YLR071C RGR1
ZShift = 1.17
Delta_K
NG = 0
DB = 0
SM = 0
Cytarabine
YLR071C RGR1
ZShift = 0.17
Delta_r
NG = 0
DB = 0
SM = 0
Cytarabine
YLR071C RGR1
ZShift = 0.06
Delta_AUC
NG = 0
DB = 0
SM = 0
Cytarabine
0 10 25 50 100

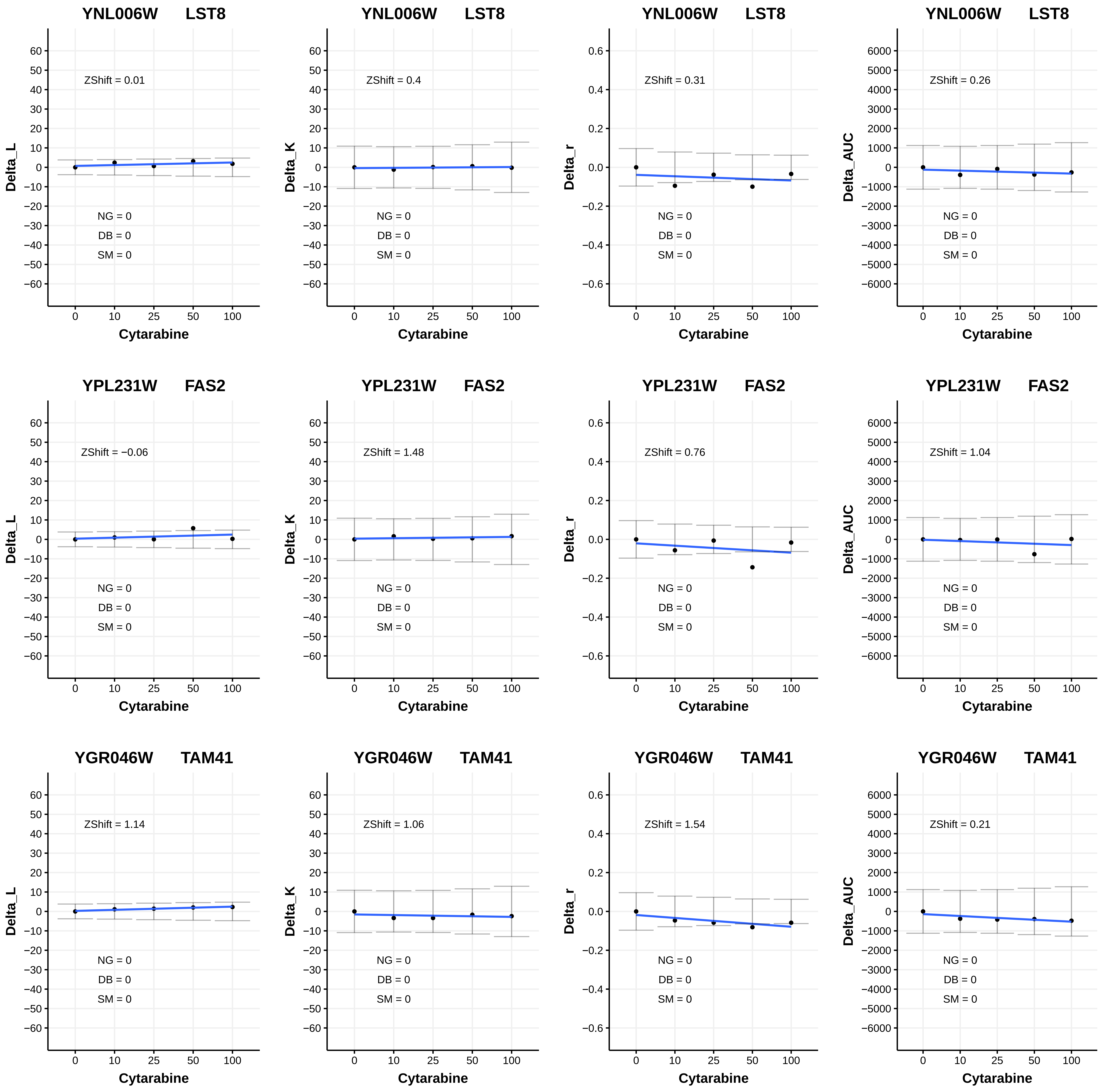
YNL006W LST8
ZShift = 0.01
ZShift = 0.4
ZShift = 0.31
ZShift = 0.26
YPL231W FAS2
ZShift = −0.06
ZShift = 1.48
ZShift = 0.76
ZShift = 1.04
YGR046W TAM41
ZShift = 1.14
ZShift = 1.06
ZShift = 1.54
ZShift = 0.21
NG = 0
DB = 0
SM = 0
Delta_L
Delta_K
Delta_r
Delta_AUC
Cytarabine
0
10
25
50
100

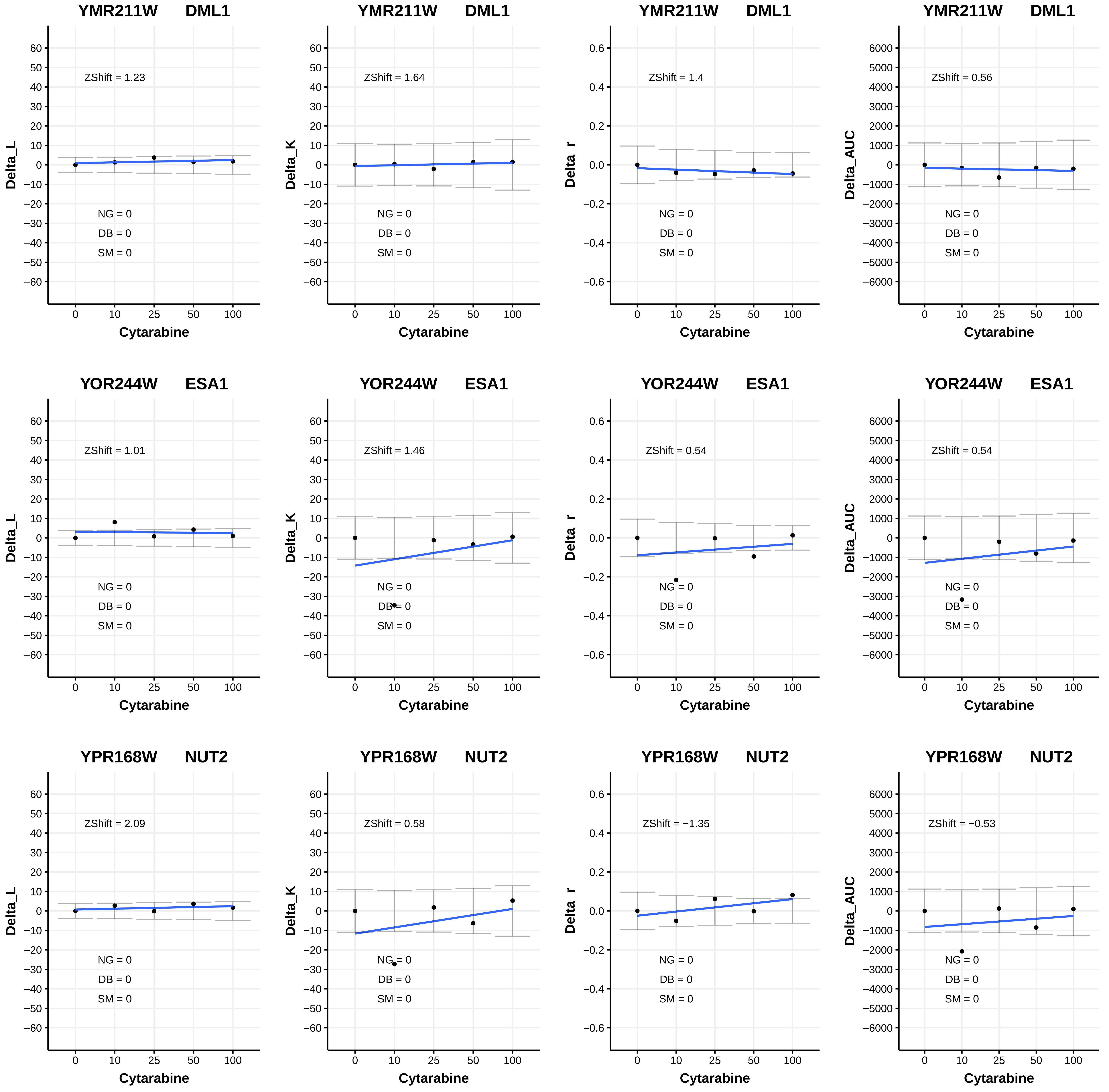
YMR211W DML1
ZShift = 1.23
ZShift = 1.64
ZShift = 1.4
ZShift = 0.56
YOR244W ESA1
ZShift = 1.01
ZShift = 1.46
ZShift = 0.54
ZShift = 0.54
YPR168W NUT2
ZShift = 2.09
ZShift = 0.58
ZShift = −1.35
ZShift = −0.53
NG = 0
DB = 0
SM = 0
Delta_L
Delta_K
Delta_r
Delta_AUC
Cytarabine
0
10
25
50
100

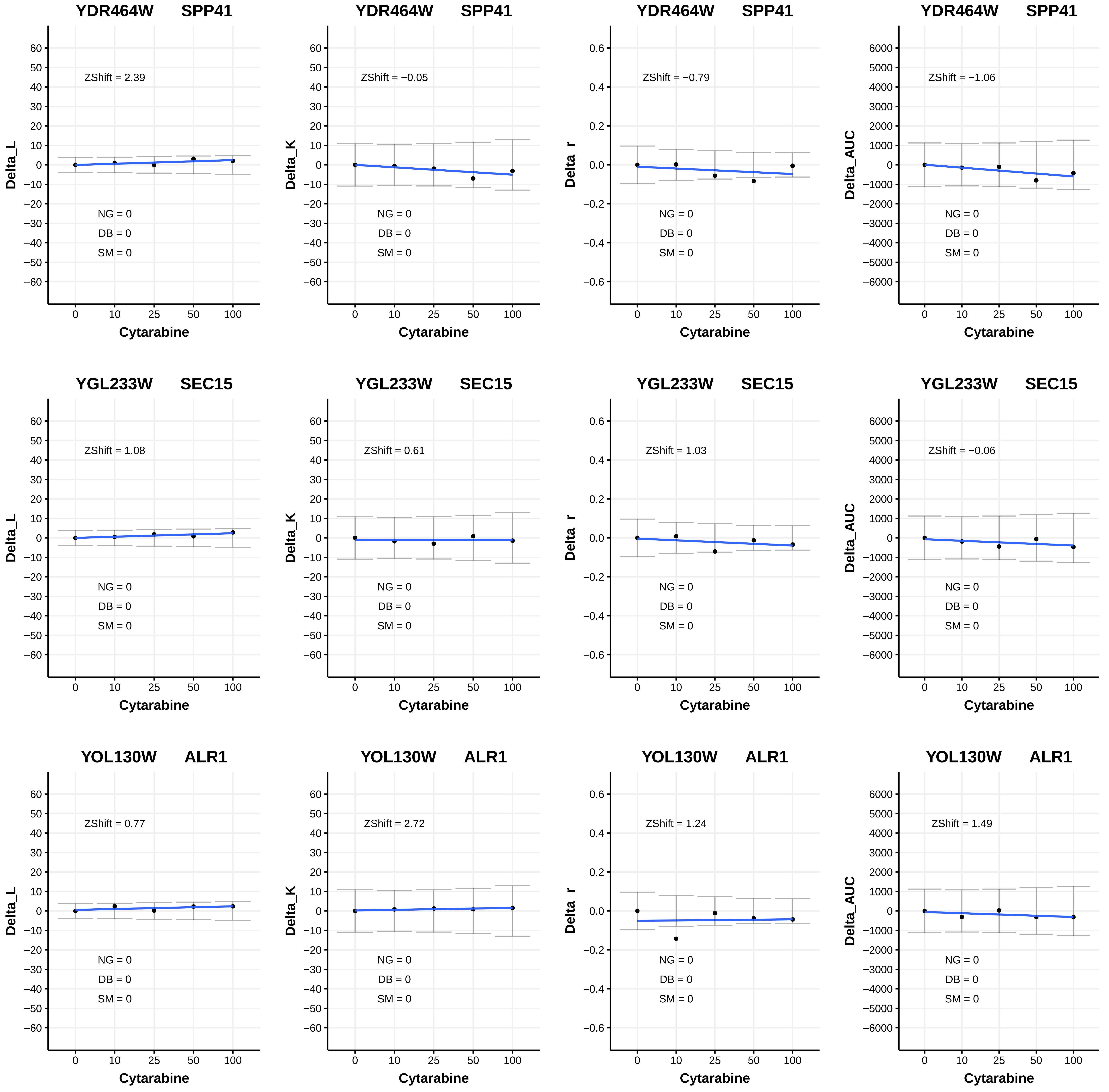
YDR464W SPP41
Delta_L
ZShift = 2.39
NG = 0
DB = 0
SM = 0
Cytarabine
YDR464W SPP41
Delta_K
ZShift = −0.05
NG = 0
DB = 0
SM = 0
Cytarabine
YDR464W SPP41
Delta_r
ZShift = −0.79
NG = 0
DB = 0
SM = 0
Cytarabine
YDR464W SPP41
Delta_AUC
ZShift = −1.06
NG = 0
DB = 0
SM = 0
Cytarabine
YGL233W SEC15
Delta_L
ZShift = 1.08
NG = 0
DB = 0
SM = 0
Cytarabine
YGL233W SEC15
Delta_K
ZShift = 0.61
NG = 0
DB = 0
SM = 0
Cytarabine
YGL233W SEC15
Delta_r
ZShift = 1.03
NG = 0
DB = 0
SM = 0
Cytarabine
YGL233W SEC15
Delta_AUC
ZShift = −0.06
NG = 0
DB = 0
SM = 0
Cytarabine
YOL130W ALR1
Delta_L
ZShift = 0.77
NG = 0
DB = 0
SM = 0
Cytarabine
YOL130W ALR1
Delta_K
ZShift = 2.72
NG = 0
DB = 0
SM = 0
Cytarabine
YOL130W ALR1
Delta_r
ZShift = 1.24
NG = 0
DB = 0
SM = 0
Cytarabine
YOL130W ALR1
Delta_AUC
ZShift = 1.49
NG = 0
DB = 0
SM = 0
Cytarabine

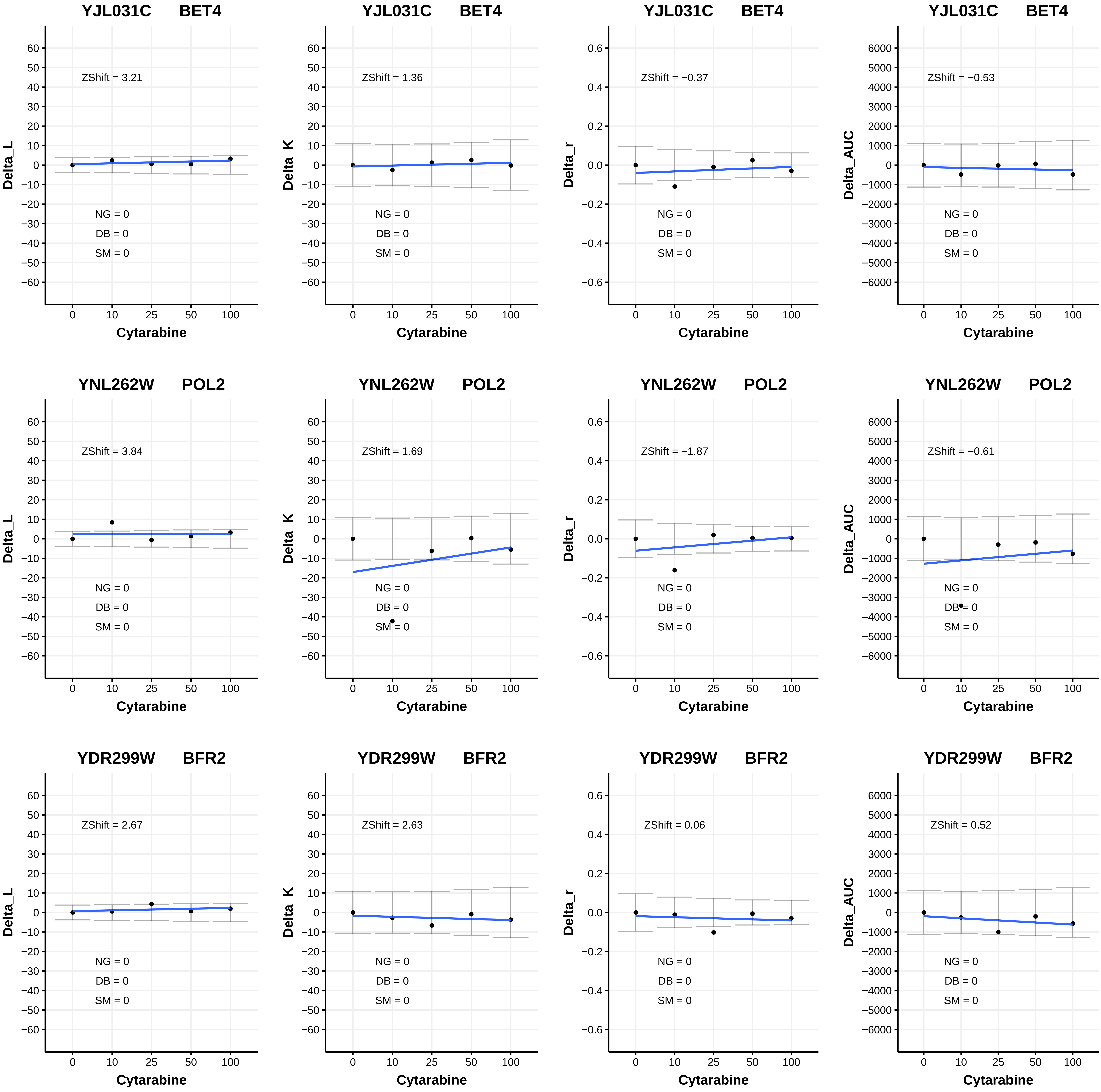
YJL031C BET4
Delta_L
ZShift = 3.21
NG = 0
DB = 0
SM = 0
Cytarabine
YJL031C BET4
Delta_K
ZShift = 1.36
NG = 0
DB = 0
SM = 0
Cytarabine
YJL031C BET4
Delta_r
ZShift = −0.37
NG = 0
DB = 0
SM = 0
Cytarabine
YJL031C BET4
Delta_AUC
ZShift = −0.53
NG = 0
DB = 0
SM = 0
Cytarabine
YNL262W POL2
Delta_L
ZShift = 3.84
NG = 0
DB = 0
SM = 0
Cytarabine
YNL262W POL2
Delta_K
ZShift = 1.69
NG = 0
DB = 0
SM = 0
Cytarabine
YNL262W POL2
Delta_r
ZShift = −1.87
NG = 0
DB = 0
SM = 0
Cytarabine
YNL262W POL2
Delta_AUC
ZShift = −0.61
NG = 0
DB = 0
SM = 0
Cytarabine
YDR299W BFR2
Delta_L
ZShift = 2.67
NG = 0
DB = 0
SM = 0
Cytarabine
YDR299W BFR2
Delta_K
ZShift = 2.63
NG = 0
DB = 0
SM = 0
Cytarabine
YDR299W BFR2
Delta_r
ZShift = 0.06
NG = 0
DB = 0
SM = 0
Cytarabine
YDR299W BFR2
Delta_AUC
ZShift = 0.52
NG = 0
DB = 0
SM = 0
Cytarabine

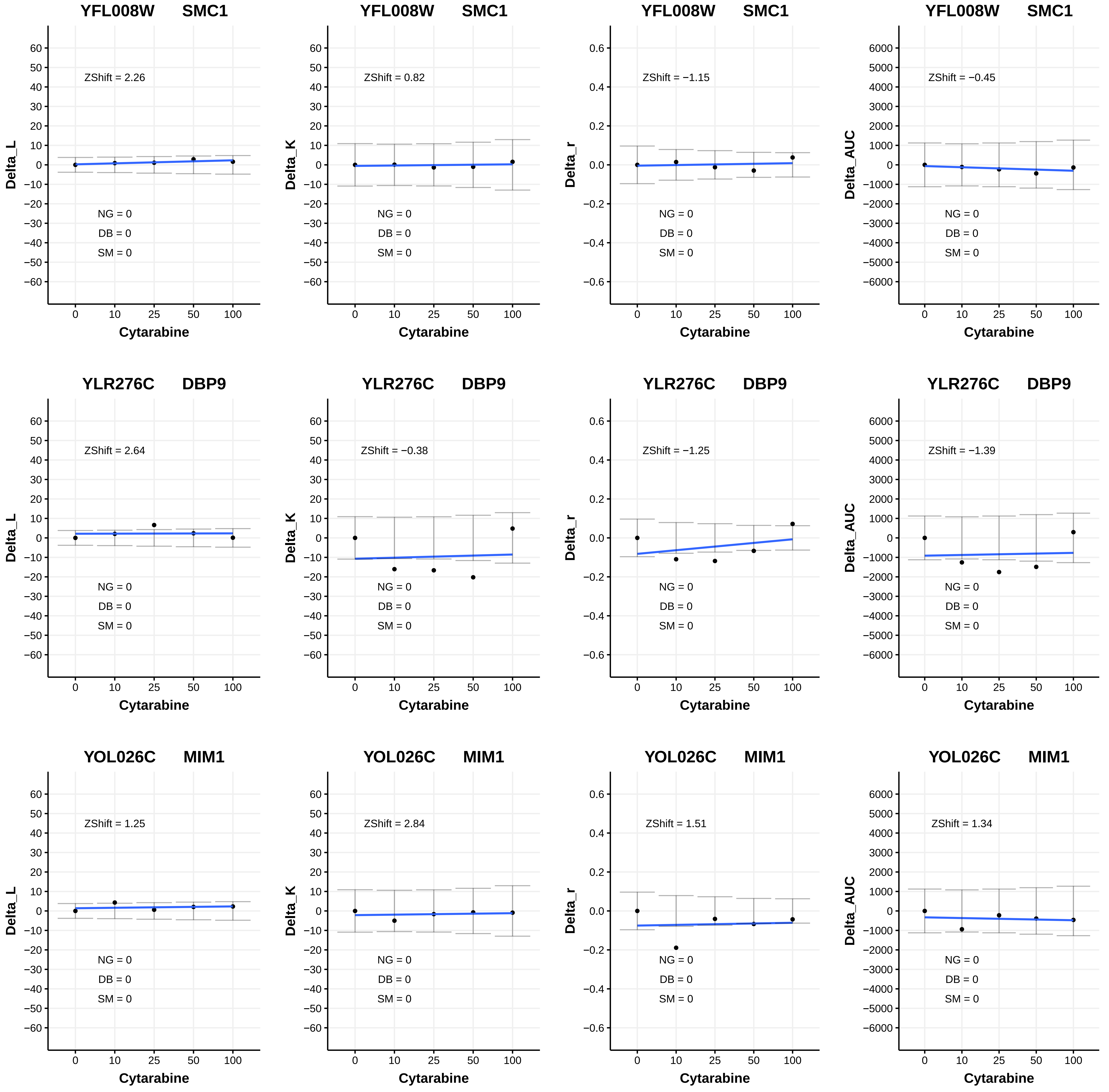

YFL008W SMC1
Delta_L
ZShift = 2.26
NG = 0
DB = 0
SM = 0
Cytarabine
YFL008W SMC1
Delta_K
ZShift = 0.82
NG = 0
DB = 0
SM = 0
Cytarabine
YFL008W SMC1
Delta_r
ZShift = −1.15
NG = 0
DB = 0
SM = 0
Cytarabine
YFL008W SMC1
Delta_AUC
ZShift = −0.45
NG = 0
DB = 0
SM = 0
Cytarabine
YLR276C DBP9
Delta_L
ZShift = 2.64
NG = 0
DB = 0
SM = 0
Cytarabine
YLR276C DBP9
Delta_K
ZShift = −0.38
NG = 0
DB = 0
SM = 0
Cytarabine
YLR276C DBP9
Delta_r
ZShift = −1.25
NG = 0
DB = 0
SM = 0
Cytarabine
YLR276C DBP9
Delta_AUC
ZShift = −1.39
NG = 0
DB = 0
SM = 0
Cytarabine
YOL026C MIM1
Delta_L
ZShift = 1.25
NG = 0
DB = 0
SM = 0
Cytarabine
YOL026C MIM1
Delta_K
ZShift = 2.84
NG = 0
DB = 0
SM = 0
Cytarabine
YOL026C MIM1
Delta_r
ZShift = 1.51
NG = 0
DB = 0
SM = 0
Cytarabine
YOL026C MIM1
Delta_AUC
ZShift = 1.34
NG = 0
DB = 0
SM = 0
Cytarabine

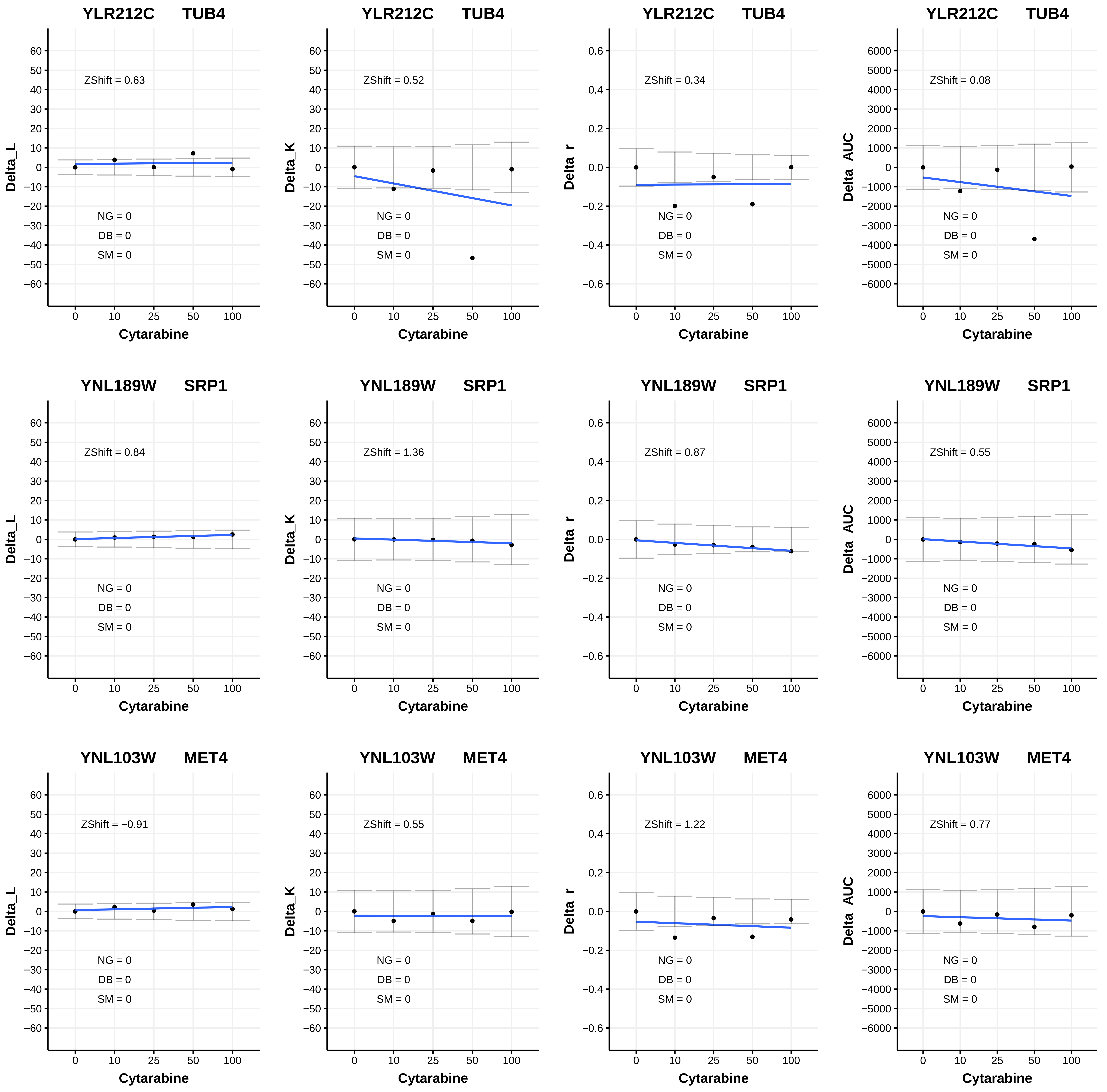
YLR212C TUB4
ZShift = 0.63
NG = 0
DB = 0
SM = 0
Delta_L
Cytarabine
YLR212C TUB4
ZShift = 0.52
NG = 0
DB = 0
SM = 0
Delta_K
Cytarabine
YLR212C TUB4
ZShift = 0.34
NG = 0
DB = 0
SM = 0
Delta_r
Cytarabine
YLR212C TUB4
ZShift = 0.08
NG = 0
DB = 0
SM = 0
Delta_AUC
Cytarabine
YNL189W SRP1
ZShift = 0.84
NG = 0
DB = 0
SM = 0
Delta_L
Cytarabine
YNL189W SRP1
ZShift = 1.36
NG = 0
DB = 0
SM = 0
Delta_K
Cytarabine
YNL189W SRP1
ZShift = 0.87
NG = 0
DB = 0
SM = 0
Delta_r
Cytarabine
YNL189W SRP1
ZShift = 0.55
NG = 0
DB = 0
SM = 0
Delta_AUC
Cytarabine
YNL103W MET4
ZShift = −0.91
NG = 0
DB = 0
SM = 0
Delta_L
Cytarabine
YNL103W MET4
ZShift = 0.55
NG = 0
DB = 0
SM = 0
Delta_K
Cytarabine
YNL103W MET4
ZShift = 1.22
NG = 0
DB = 0
SM = 0
Delta_r
Cytarabine
YNL103W MET4
ZShift = 0.77
NG = 0
DB = 0
SM = 0
Delta_AUC
Cytarabine

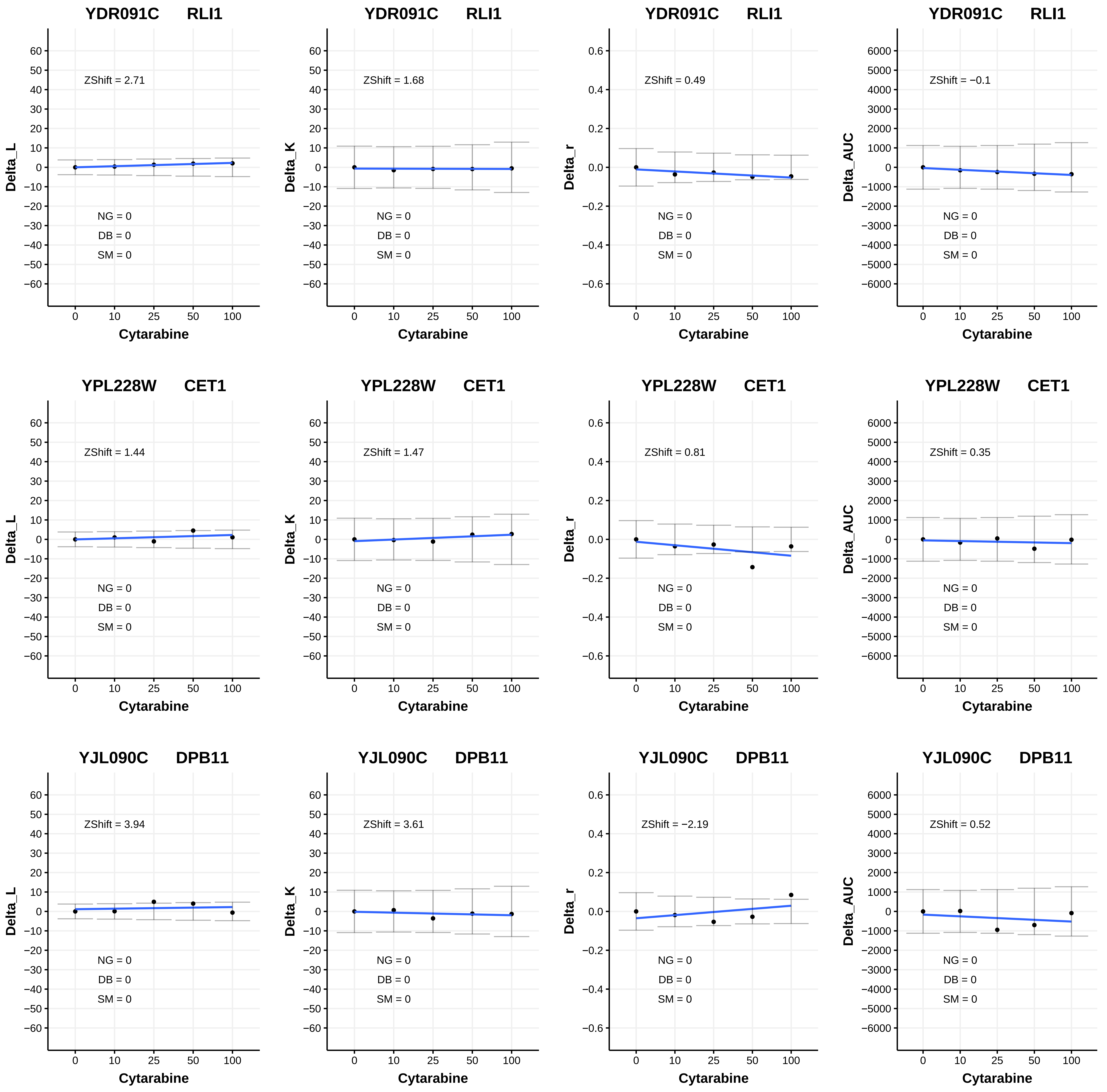
YDR091C RLI1
ZShift = 2.71
ZShift = 1.68
ZShift = 0.49
ZShift = −0.1
YPL228W CET1
ZShift = 1.44
ZShift = 1.47
ZShift = 0.81
ZShift = 0.35
YJL090C DPB11
ZShift = 3.94
ZShift = 3.61
ZShift = −2.19
ZShift = 0.52
NG = 0
DB = 0
SM = 0
Delta_L
Delta_K
Delta_r
Delta_AUC
Cytarabine
0
10
25
50
100

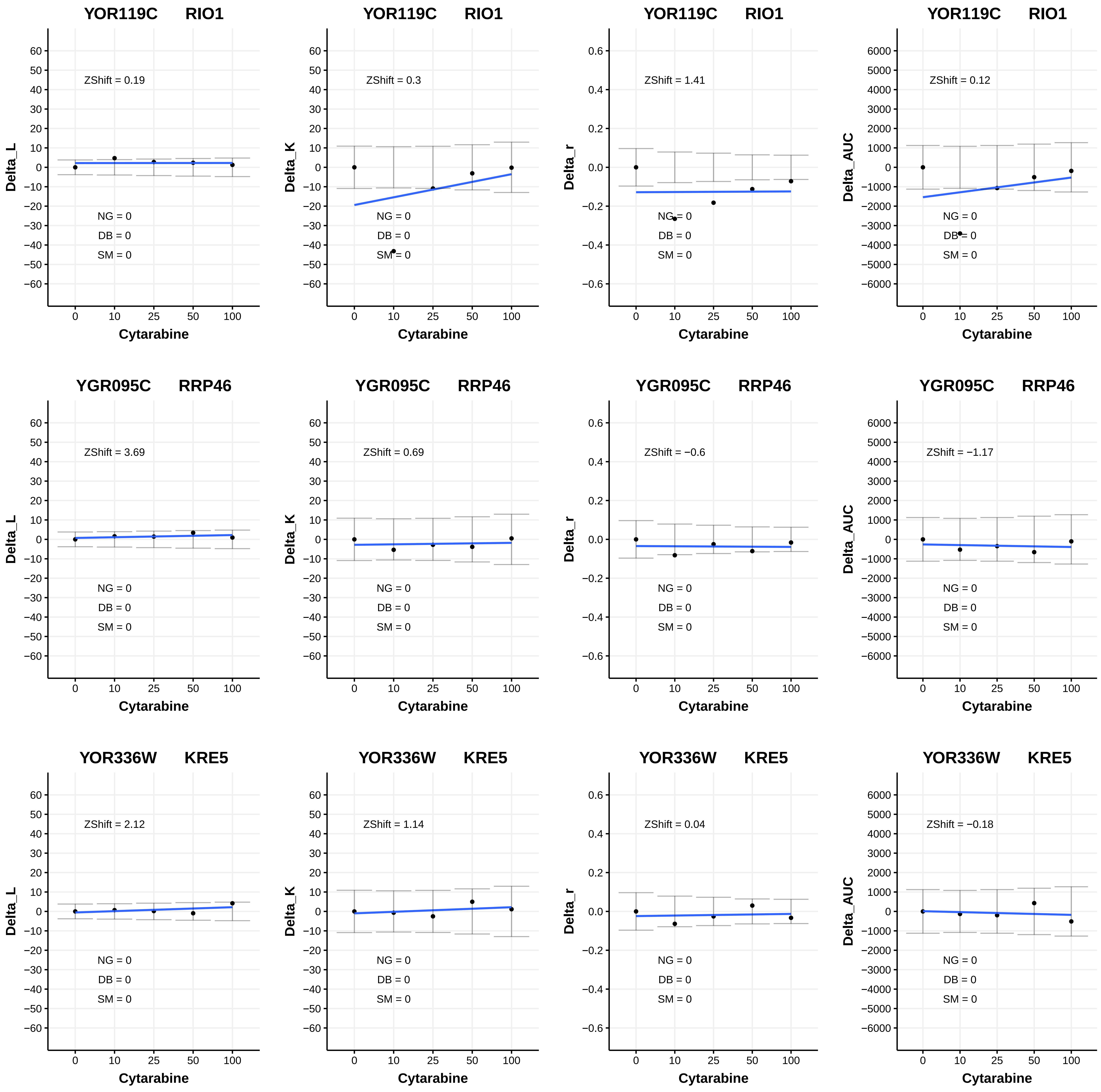
YOR119C RIO1
ZShift = 0.19
NG = 0
DB = 0
SM = 0
Delta_L
Cytarabine
YOR119C RIO1
ZShift = 0.3
NG = 0
DB = 0
SM = 0
Delta_K
Cytarabine
YOR119C RIO1
ZShift = 1.41
NG = 0
DB = 0
SM = 0
Delta_r
Cytarabine
YOR119C RIO1
ZShift = 0.12
NG = 0
DB = 0
SM = 0
Delta_AUC
Cytarabine
YGR095C RRP46
ZShift = 3.69
NG = 0
DB = 0
SM = 0
Delta_L
Cytarabine
YGR095C RRP46
ZShift = 0.69
NG = 0
DB = 0
SM = 0
Delta_K
Cytarabine
YGR095C RRP46
ZShift = −0.6
NG = 0
DB = 0
SM = 0
Delta_r
Cytarabine
YGR095C RRP46
ZShift = −1.17
NG = 0
DB = 0
SM = 0
Delta_AUC
Cytarabine
YOR336W KRE5
ZShift = 2.12
NG = 0
DB = 0
SM = 0
Delta_L
Cytarabine
YOR336W KRE5
ZShift = 1.14
NG = 0
DB = 0
SM = 0
Delta_K
Cytarabine
YOR336W KRE5
ZShift = 0.04
NG = 0
DB = 0
SM = 0
Delta_r
Cytarabine
YOR336W KRE5
ZShift = −0.18
NG = 0
DB = 0
SM = 0
Delta_AUC
Cytarabine

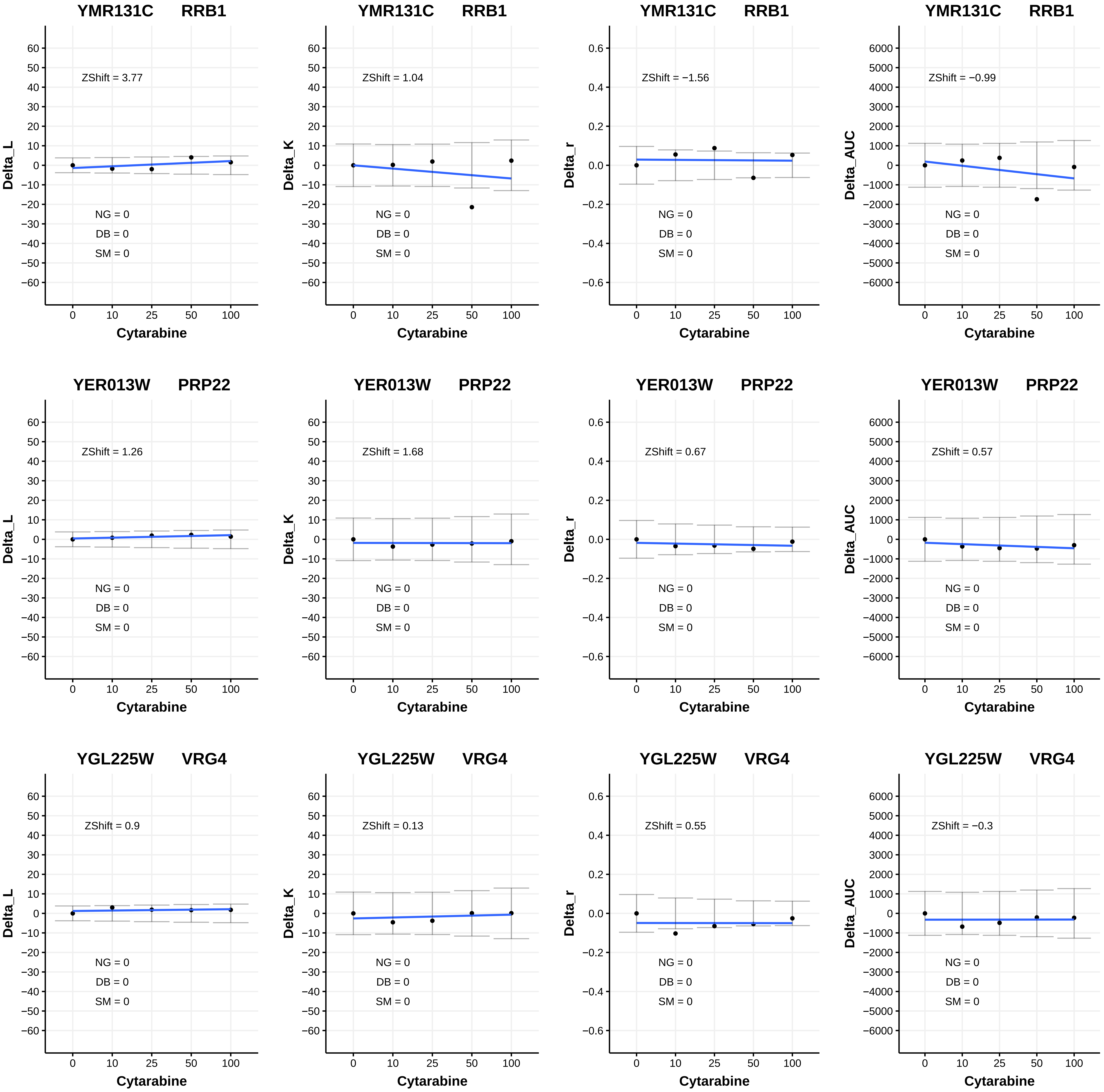
YMR131C RRB1
ZShift = 3.77
NG = 0
DB = 0
SM = 0
Delta_L
Cytarabine
YMR131C RRB1
ZShift = 1.04
NG = 0
DB = 0
SM = 0
Delta_K
Cytarabine
YMR131C RRB1
ZShift = −1.56
NG = 0
DB = 0
SM = 0
Delta_r
Cytarabine
YMR131C RRB1
ZShift = −0.99
NG = 0
DB = 0
SM = 0
Delta_AUC
Cytarabine
YER013W PRP22
ZShift = 1.26
NG = 0
DB = 0
SM = 0
Delta_L
Cytarabine
YER013W PRP22
ZShift = 1.68
NG = 0
DB = 0
SM = 0
Delta_K
Cytarabine
YER013W PRP22
ZShift = 0.67
NG = 0
DB = 0
SM = 0
Delta_r
Cytarabine
YER013W PRP22
ZShift = 0.57
NG = 0
DB = 0
SM = 0
Delta_AUC
Cytarabine
YGL225W VRG4
ZShift = 0.9
NG = 0
DB = 0
SM = 0
Delta_L
Cytarabine
YGL225W VRG4
ZShift = 0.13
NG = 0
DB = 0
SM = 0
Delta_K
Cytarabine
YGL225W VRG4
ZShift = 0.55
NG = 0
DB = 0
SM = 0
Delta_r
Cytarabine
YGL225W VRG4
ZShift = −0.3
NG = 0
DB = 0
SM = 0
Delta_AUC
Cytarabine

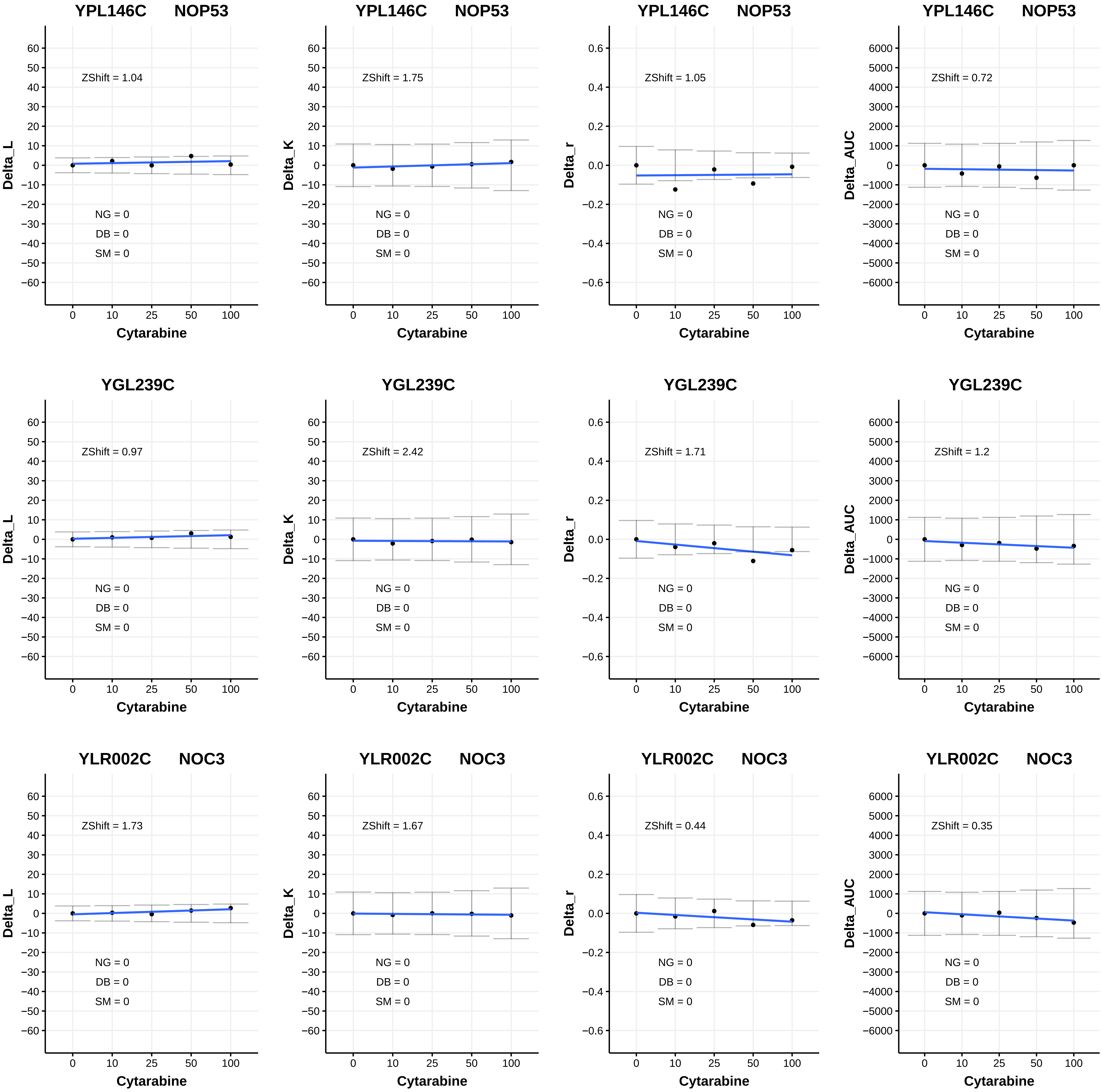
YPL146C NOP53
ZShift = 1.04
Delta_L
NG = 0
DB = 0
SM = 0
Cytarabine
YPL146C NOP53
ZShift = 1.75
Delta_K
NG = 0
DB = 0
SM = 0
Cytarabine
YPL146C NOP53
ZShift = 1.05
Delta_r
NG = 0
DB = 0
SM = 0
Cytarabine
YPL146C NOP53
ZShift = 0.72
Delta_AUC
NG = 0
DB = 0
SM = 0
Cytarabine
YGL239C
ZShift = 0.97
Delta_L
NG = 0
DB = 0
SM = 0
Cytarabine
YGL239C
ZShift = 2.42
Delta_K
NG = 0
DB = 0
SM = 0
Cytarabine
YGL239C
ZShift = 1.71
Delta_r
NG = 0
DB = 0
SM = 0
Cytarabine
YGL239C
ZShift = 1.2
Delta_AUC
NG = 0
DB = 0
SM = 0
Cytarabine
YLR002C NOC3
ZShift = 1.73
Delta_L
NG = 0
DB = 0
SM = 0
Cytarabine
YLR002C NOC3
ZShift = 1.67
Delta_K
NG = 0
DB = 0
SM = 0
Cytarabine
YLR002C NOC3
ZShift = 0.44
Delta_r
NG = 0
DB = 0
SM = 0
Cytarabine
YLR002C NOC3
ZShift = 0.35
Delta_AUC
NG = 0
DB = 0
SM = 0
Cytarabine

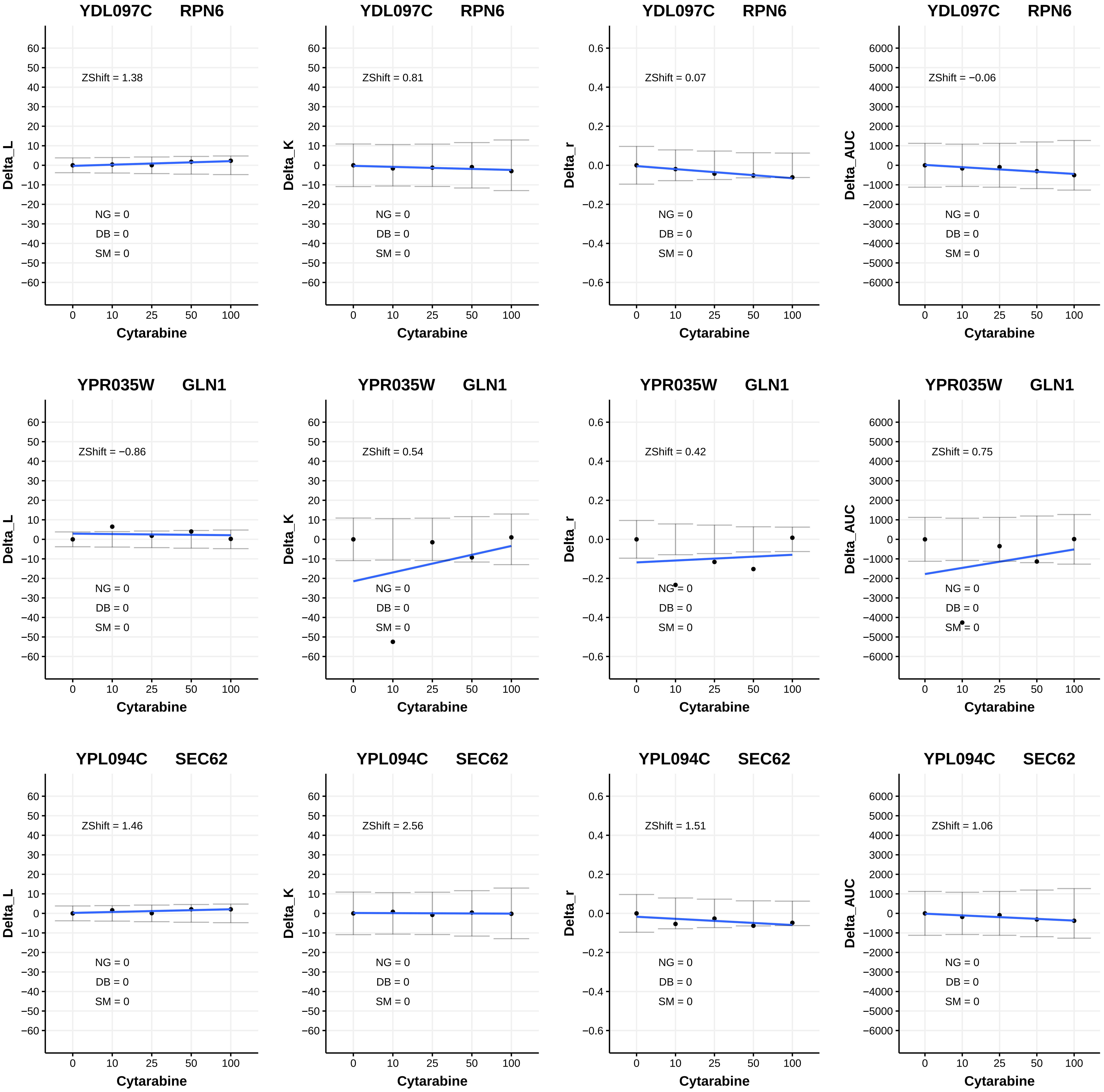
YDL097C RPN6
ZShift = 1.38
Delta_L
NG = 0
DB = 0
SM = 0
Cytarabine
YDL097C RPN6
ZShift = 0.81
Delta_K
NG = 0
DB = 0
SM = 0
Cytarabine
YDL097C RPN6
ZShift = 0.07
Delta_r
NG = 0
DB = 0
SM = 0
Cytarabine
YDL097C RPN6
ZShift = −0.06
Delta_AUC
NG = 0
DB = 0
SM = 0
Cytarabine
YPR035W GLN1
ZShift = −0.86
Delta_L
NG = 0
DB = 0
SM = 0
Cytarabine
YPR035W GLN1
ZShift = 0.54
Delta_K
NG = 0
DB = 0
SM = 0
Cytarabine
YPR035W GLN1
ZShift = 0.42
Delta_r
NG = 0
DB = 0
SM = 0
Cytarabine
YPR035W GLN1
ZShift = 0.75
Delta_AUC
NG = 0
DB = 0
SM = 0
Cytarabine
YPL094C SEC62
ZShift = 1.46
Delta_L
NG = 0
DB = 0
SM = 0
Cytarabine
YPL094C SEC62
ZShift = 2.56
Delta_K
NG = 0
DB = 0
SM = 0
Cytarabine
YPL094C SEC62
ZShift = 1.51
Delta_r
NG = 0
DB = 0
SM = 0
Cytarabine
YPL094C SEC62
ZShift = 1.06
Delta_AUC
NG = 0
DB = 0
SM = 0
Cytarabine

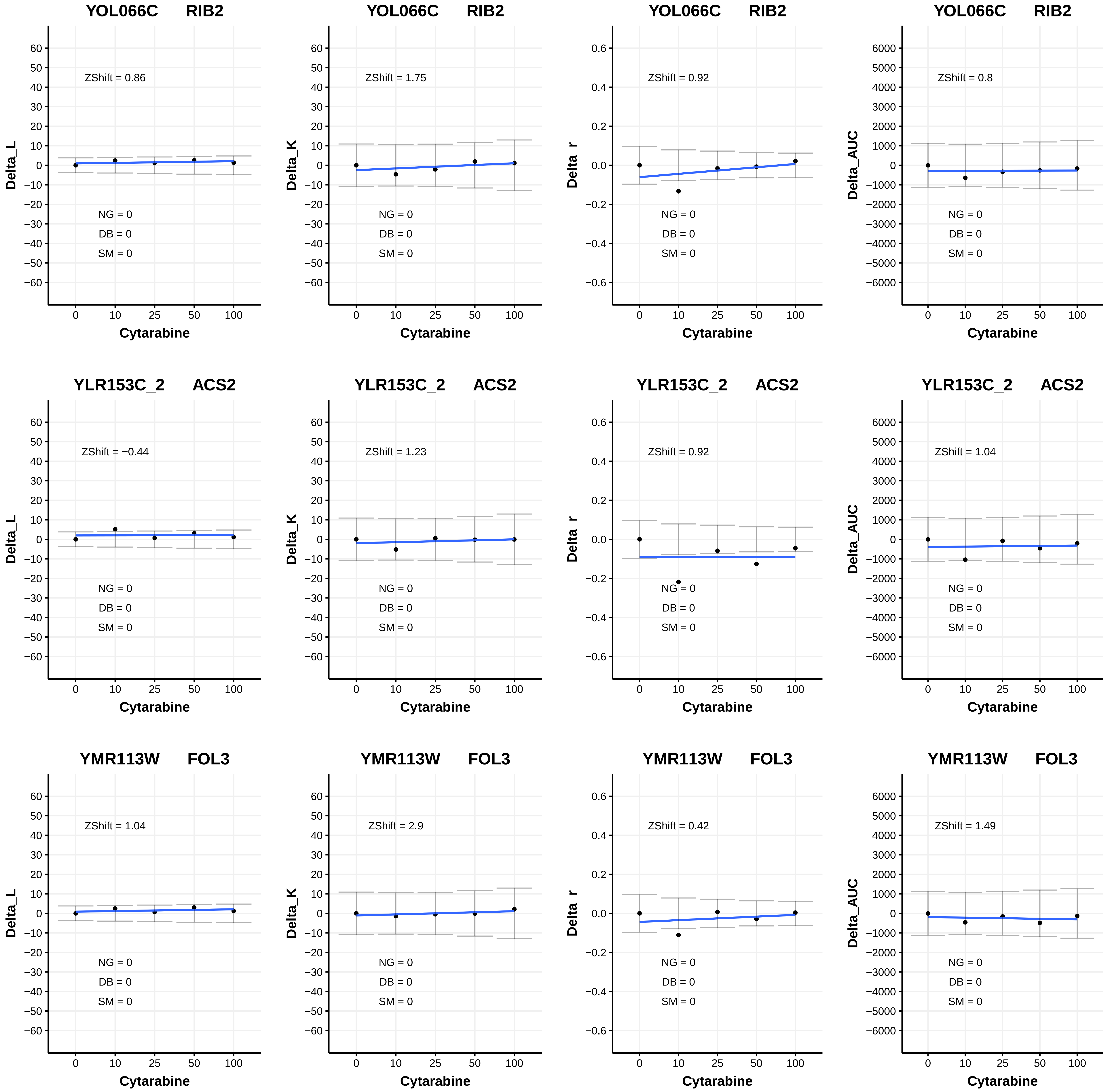
YOL066C RIB2
ZShift = 0.86
ZShift = 1.75
ZShift = 0.92
ZShift = 0.8
YLR153C_2 ACS2
ZShift = −0.44
ZShift = 1.23
ZShift = 0.92
ZShift = 1.04
YMR113W FOL3
ZShift = 1.04
ZShift = 2.9
ZShift = 0.42
ZShift = 1.49
NG = 0
DB = 0
SM = 0
Delta_L
Delta_K
Delta_r
Delta_AUC
Cytarabine

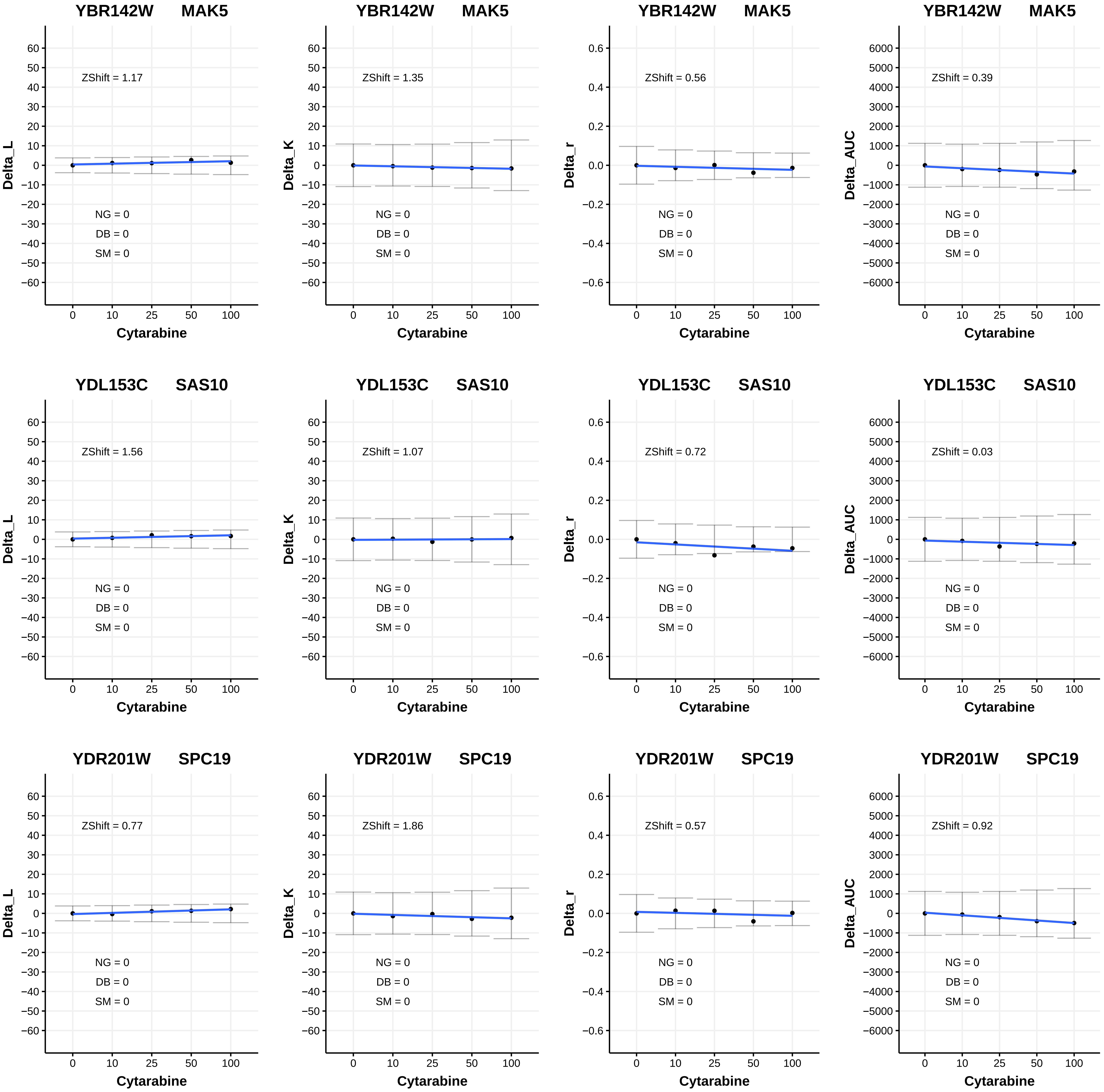
YBR142W MAK5
ZShift = 1.17
NG = 0
DB = 0
SM = 0
Delta_L
Cytarabine
YBR142W MAK5
ZShift = 1.35
NG = 0
DB = 0
SM = 0
Delta_K
Cytarabine
YBR142W MAK5
ZShift = 0.56
NG = 0
DB = 0
SM = 0
Delta_r
Cytarabine
YBR142W MAK5
ZShift = 0.39
NG = 0
DB = 0
SM = 0
Delta_AUC
Cytarabine
YDL153C SAS10
ZShift = 1.56
NG = 0
DB = 0
SM = 0
Delta_L
Cytarabine
YDL153C SAS10
ZShift = 1.07
NG = 0
DB = 0
SM = 0
Delta_K
Cytarabine
YDL153C SAS10
ZShift = 0.72
NG = 0
DB = 0
SM = 0
Delta_r
Cytarabine
YDL153C SAS10
ZShift = 0.03
NG = 0
DB = 0
SM = 0
Delta_AUC
Cytarabine
YDR201W SPC19
ZShift = 0.77
NG = 0
DB = 0
SM = 0
Delta_L
Cytarabine
YDR201W SPC19
ZShift = 1.86
NG = 0
DB = 0
SM = 0
Delta_K
Cytarabine
YDR201W SPC19
ZShift = 0.57
NG = 0
DB = 0
SM = 0
Delta_r
Cytarabine
YDR201W SPC19
ZShift = 0.92
NG = 0
DB = 0
SM = 0
Delta_AUC
Cytarabine

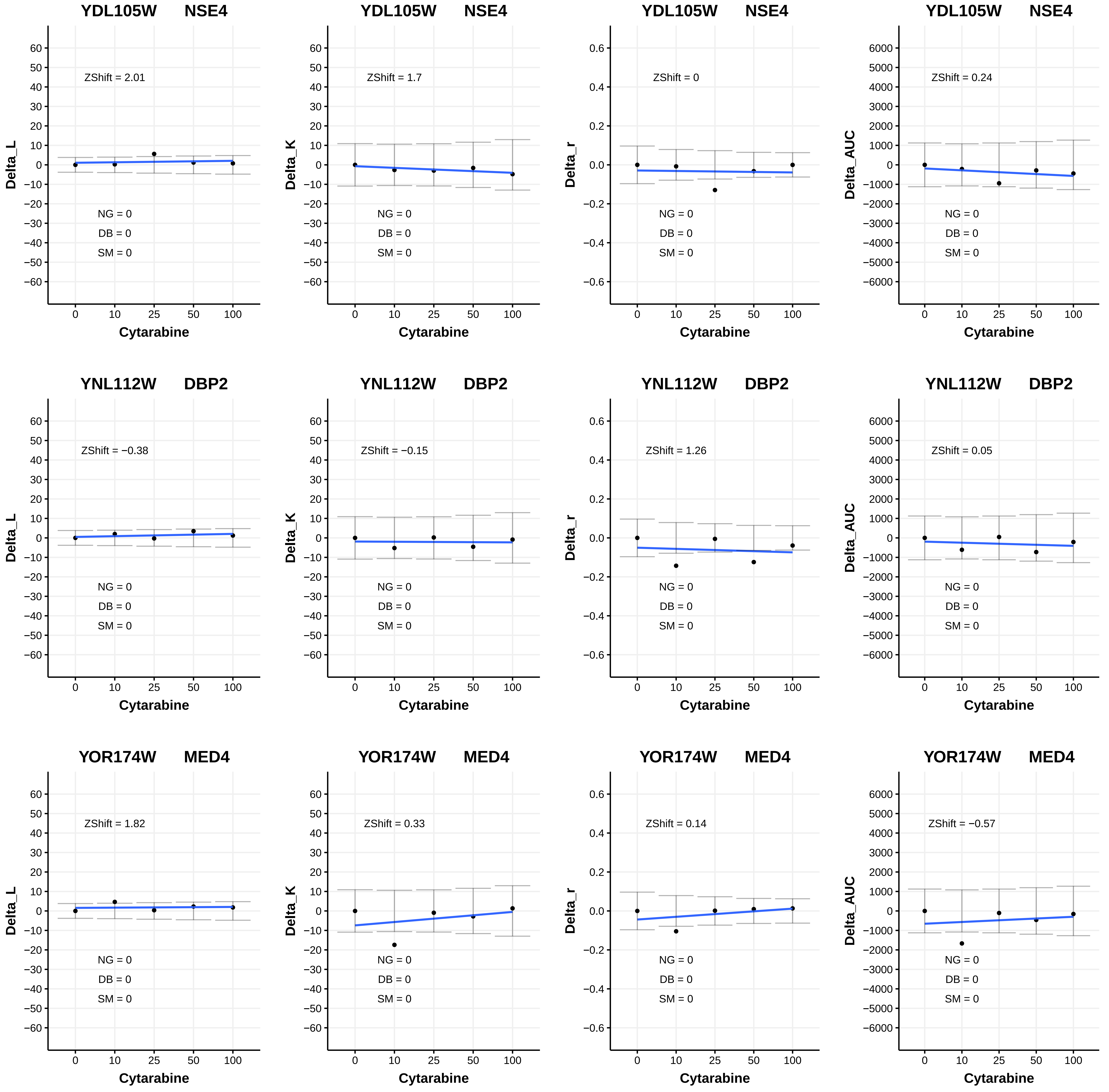
YDL105W NSE4
ZShift = 2.01
ZShift = 1.7
ZShift = 0
ZShift = 0.24
Delta_L
Delta_K
Delta_r
Delta_AUC
NG = 0
DB = 0
SM = 0
Cytarabine
0 10 25 50 100
YNL112W DBP2
ZShift = −0.38
ZShift = −0.15
ZShift = 1.26
ZShift = 0.05
YOR174W MED4
ZShift = 1.82
ZShift = 0.33
ZShift = 0.14
ZShift = −0.57

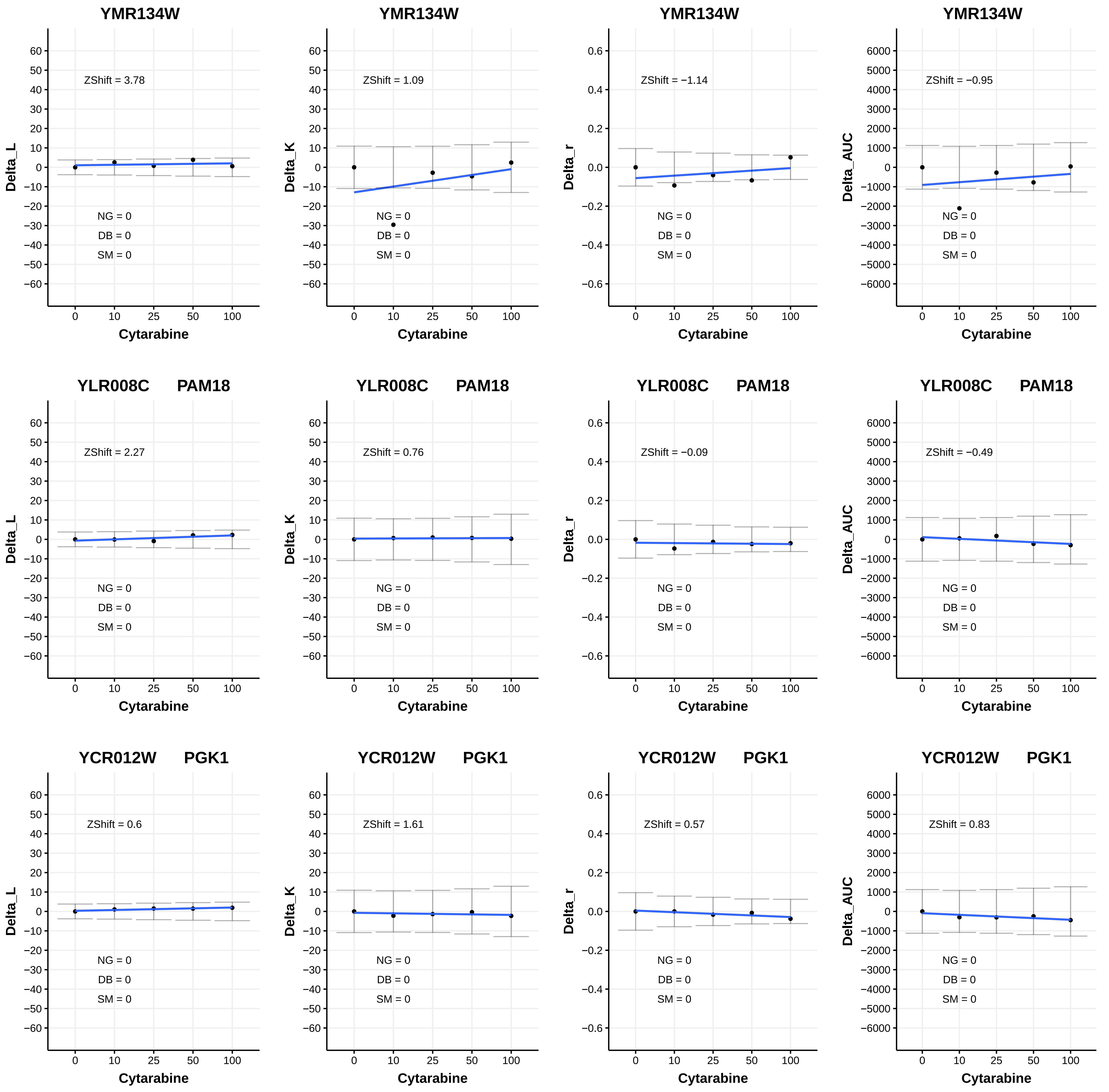
YMR134W
ZShift = 3.78
NG = 0
DB = 0
SM = 0
Delta_L
Cytarabine
YMR134W
ZShift = 1.09
NG = 0
DB = 0
SM = 0
Delta_K
Cytarabine
YMR134W
ZShift = −1.14
NG = 0
DB = 0
SM = 0
Delta_r
Cytarabine
YMR134W
ZShift = −0.95
NG = 0
DB = 0
SM = 0
Delta_AUC
Cytarabine
YLR008C PAM18
ZShift = 2.27
NG = 0
DB = 0
SM = 0
Delta_L
Cytarabine
YLR008C PAM18
ZShift = 0.76
NG = 0
DB = 0
SM = 0
Delta_K
Cytarabine
YLR008C PAM18
ZShift = −0.09
NG = 0
DB = 0
SM = 0
Delta_r
Cytarabine
YLR008C PAM18
ZShift = −0.49
NG = 0
DB = 0
SM = 0
Delta_AUC
Cytarabine
YCR012W PGK1
ZShift = 0.6
NG = 0
DB = 0
SM = 0
Delta_L
Cytarabine
YCR012W PGK1
ZShift = 1.61
NG = 0
DB = 0
SM = 0
Delta_K
Cytarabine
YCR012W PGK1
ZShift = 0.57
NG = 0
DB = 0
SM = 0
Delta_r
Cytarabine
YCR012W PGK1
ZShift = 0.83
NG = 0
DB = 0
SM = 0
Delta_AUC
Cytarabine
0
10
25
50
100

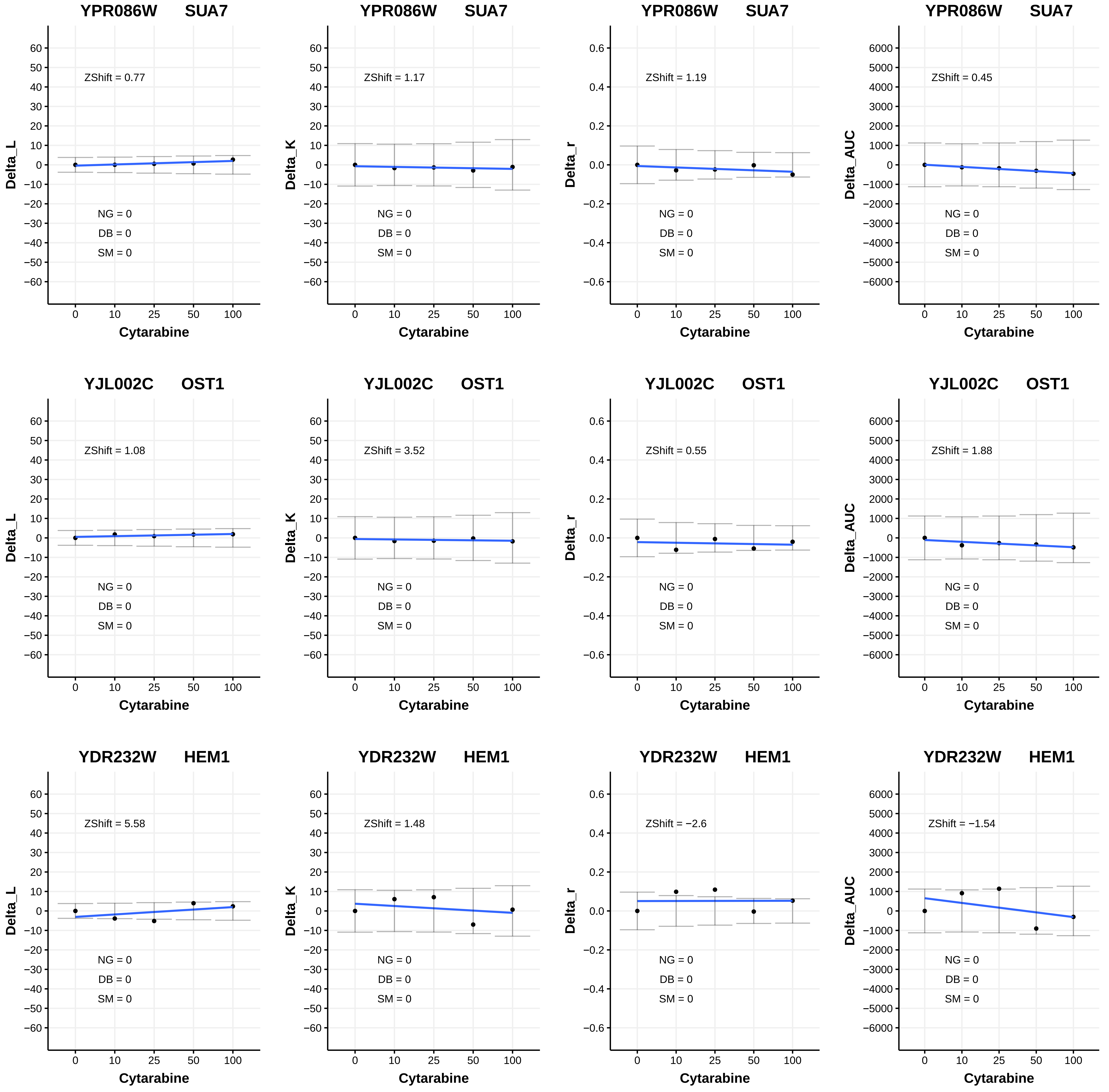
YPR086W SUA7
ZShift = 0.77
NG = 0
DB = 0
SM = 0
Delta_L
Cytarabine
YPR086W SUA7
ZShift = 1.17
NG = 0
DB = 0
SM = 0
Delta_K
Cytarabine
YPR086W SUA7
ZShift = 1.19
NG = 0
DB = 0
SM = 0
Delta_r
Cytarabine
YPR086W SUA7
ZShift = 0.45
NG = 0
DB = 0
SM = 0
Delta_AUC
Cytarabine
YJL002C OST1
ZShift = 1.08
NG = 0
DB = 0
SM = 0
Delta_L
Cytarabine
YJL002C OST1
ZShift = 3.52
NG = 0
DB = 0
SM = 0
Delta_K
Cytarabine
YJL002C OST1
ZShift = 0.55
NG = 0
DB = 0
SM = 0
Delta_r
Cytarabine
YJL002C OST1
ZShift = 1.88
NG = 0
DB = 0
SM = 0
Delta_AUC
Cytarabine
YDR232W HEM1
ZShift = 5.58
NG = 0
DB = 0
SM = 0
Delta_L
Cytarabine
YDR232W HEM1
ZShift = 1.48
NG = 0
DB = 0
SM = 0
Delta_K
Cytarabine
YDR232W HEM1
ZShift = −2.6
NG = 0
DB = 0
SM = 0
Delta_r
Cytarabine
YDR232W HEM1
ZShift = −1.54
NG = 0
DB = 0
SM = 0
Delta_AUC
Cytarabine

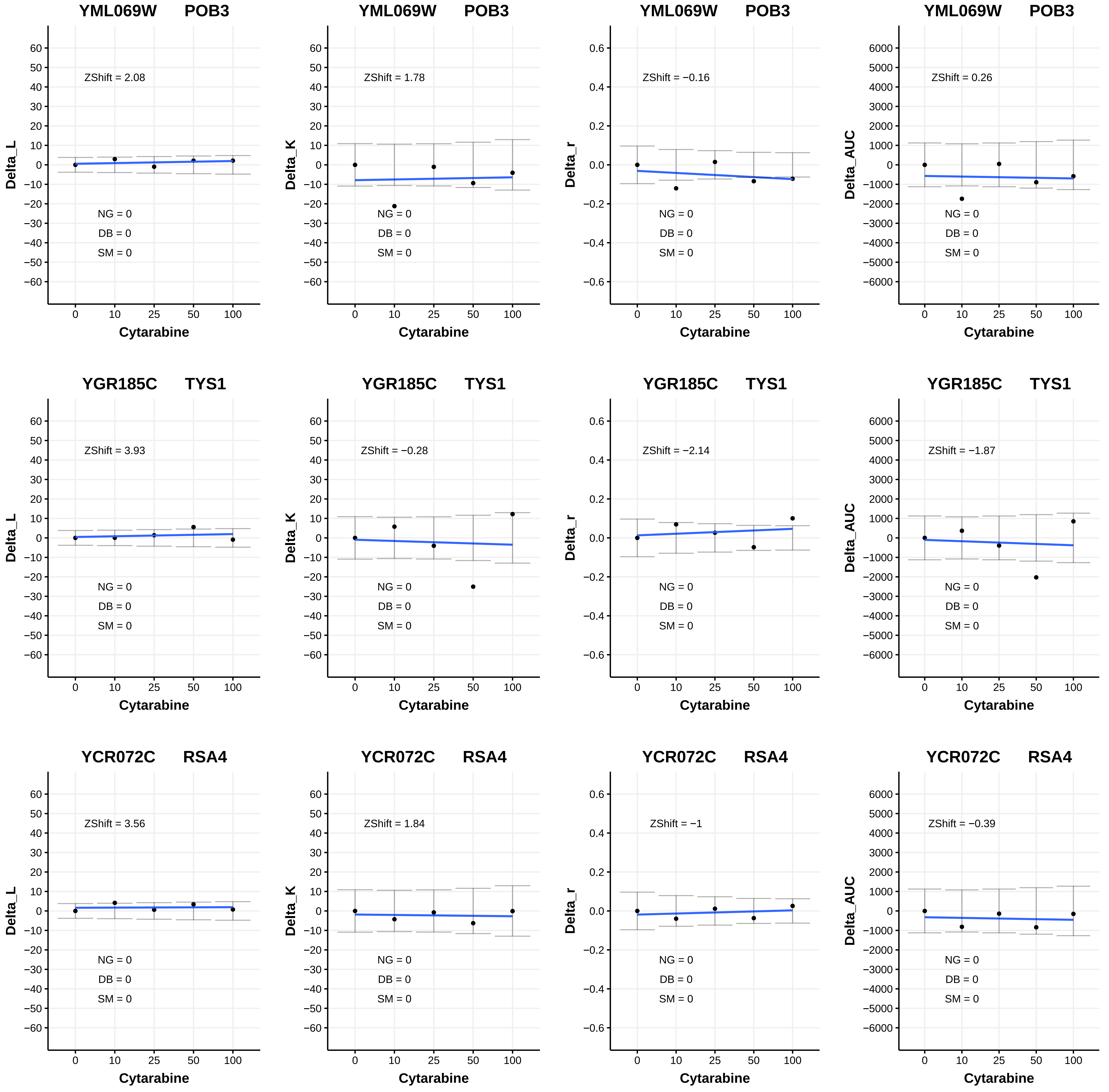
YML069W POB3
ZShift = 2.08
ZShift = 1.78
ZShift = −0.16
ZShift = 0.26
YGR185C TYS1
ZShift = 3.93
ZShift = −0.28
ZShift = −2.14
ZShift = −1.87
YCR072C RSA4
ZShift = 3.56
ZShift = 1.84
ZShift = −1
ZShift = −0.39
NG = 0
DB = 0
SM = 0
Delta_L
Delta_K
Delta_r
Delta_AUC
Cytarabine
0 10 25 50 100

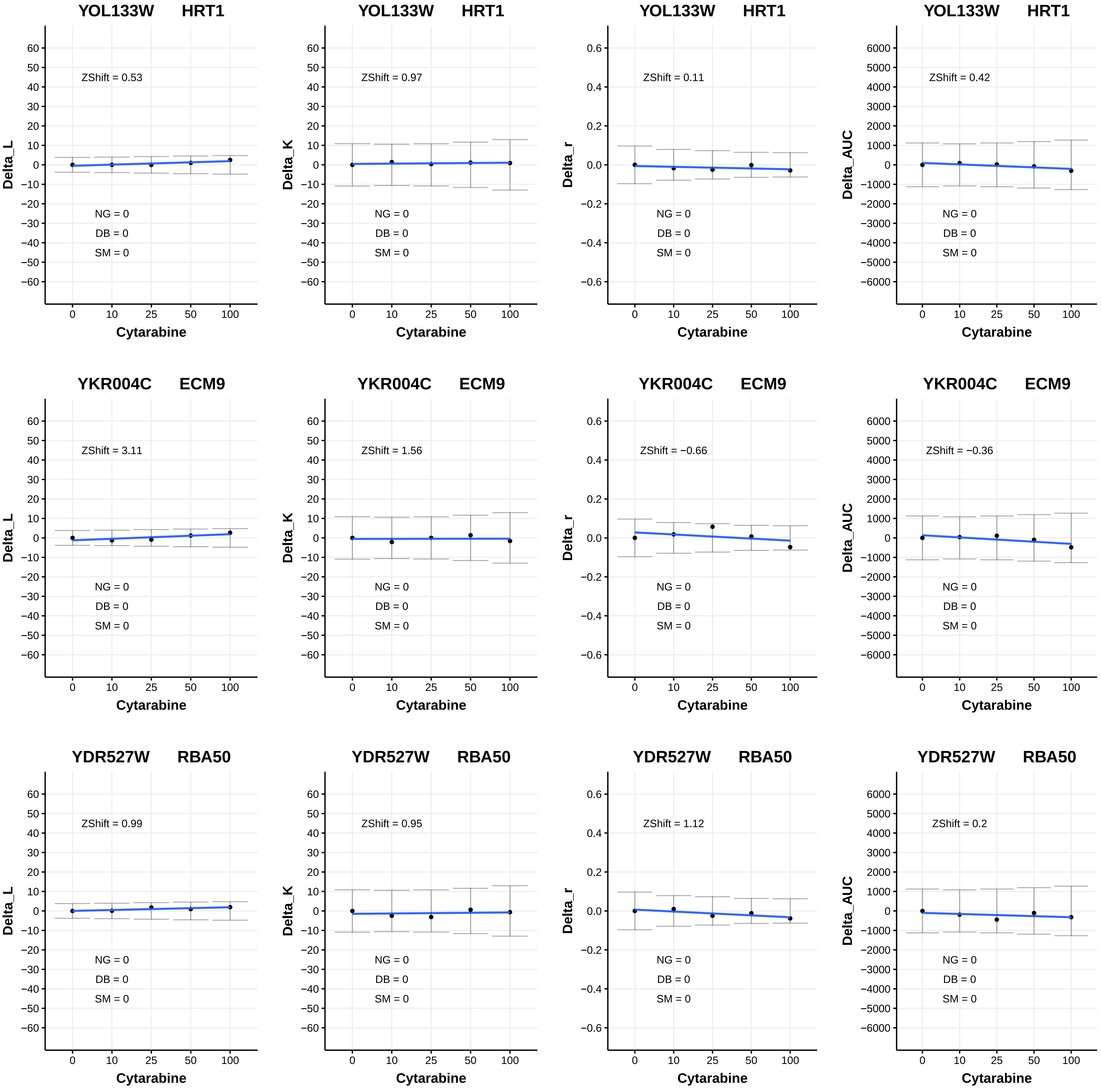

YOL133W HRT1
Delta_L
ZShift = 0.53
NG = 0
DB = 0
SM = 0
Cytarabine
YOL133W HRT1
Delta_K
ZShift = 0.97
NG = 0
DB = 0
SM = 0
Cytarabine
YOL133W HRT1
Delta_r
ZShift = 0.11
NG = 0
DB = 0
SM = 0
Cytarabine
YOL133W HRT1
Delta_AUC
ZShift = 0.42
NG = 0
DB = 0
SM = 0
Cytarabine
YKR004C ECM9
Delta_L
ZShift = 3.11
NG = 0
DB = 0
SM = 0
Cytarabine
YKR004C ECM9
Delta_K
ZShift = 1.56
NG = 0
DB = 0
SM = 0
Cytarabine
YKR004C ECM9
Delta_r
ZShift = −0.66
NG = 0
DB = 0
SM = 0
Cytarabine
YKR004C ECM9
Delta_AUC
ZShift = −0.36
NG = 0
DB = 0
SM = 0
Cytarabine
YDR527W RBA50
Delta_L
ZShift = 0.99
NG = 0
DB = 0
SM = 0
Cytarabine
YDR527W RBA50
Delta_K
ZShift = 0.95
NG = 0
DB = 0
SM = 0
Cytarabine
YDR527W RBA50
Delta_r
ZShift = 1.12
NG = 0
DB = 0
SM = 0
Cytarabine
YDR527W RBA50
Delta_AUC
ZShift = 0.2
NG = 0
DB = 0
SM = 0
Cytarabine

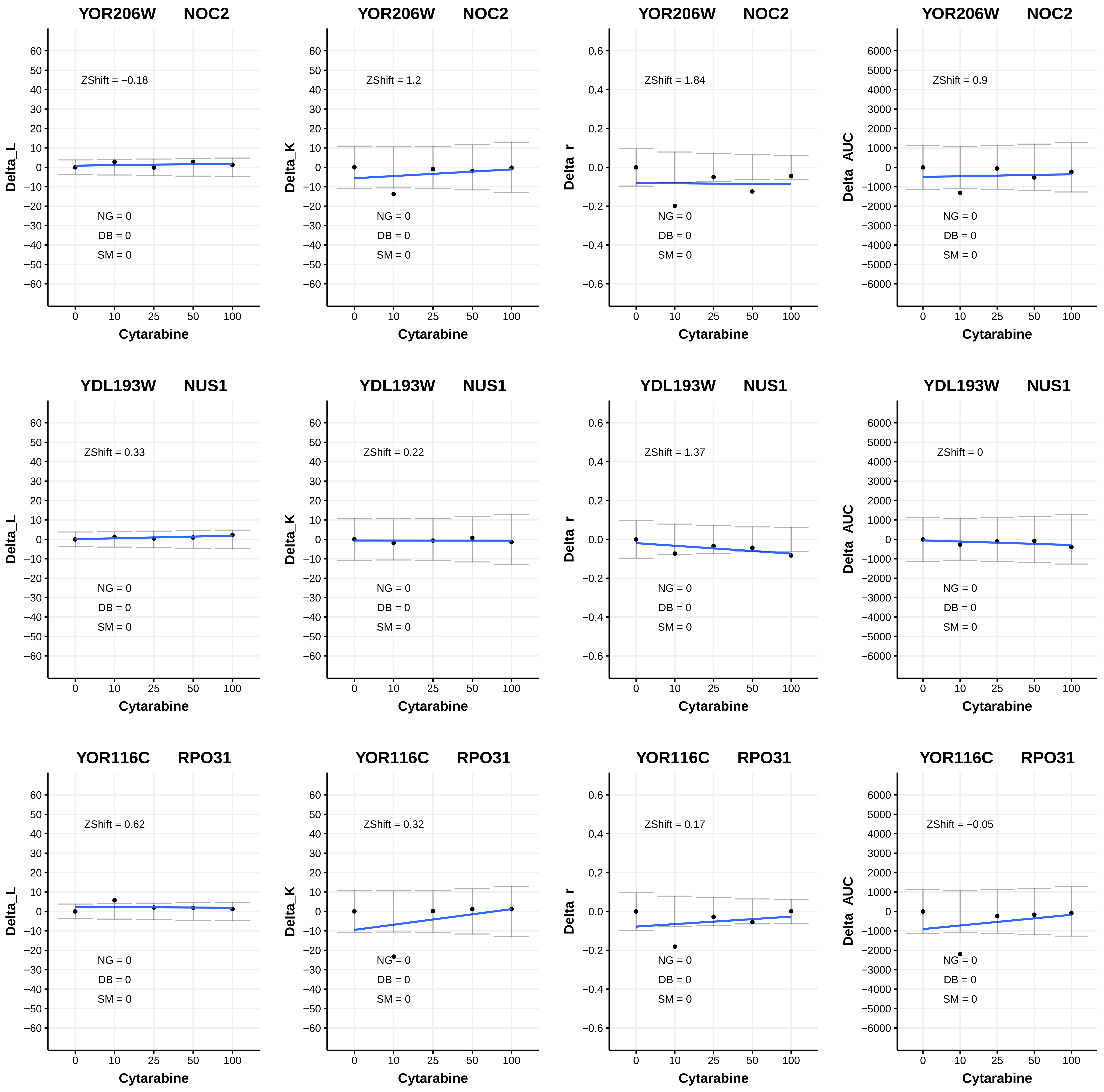
YOR206W NOC2
ZShift = −0.18
ZShift = 1.2
ZShift = 1.84
ZShift = 0.9
YDL193W NUS1
ZShift = 0.33
ZShift = 0.22
ZShift = 1.37
ZShift = 0
YOR116C RPO31
ZShift = 0.62
ZShift = 0.32
ZShift = 0.17
ZShift = −0.05
NG = 0
DB = 0
SM = 0
Delta_L
Delta_K
Delta_r
Delta_AUC
Cytarabine
0 10 25 50 100

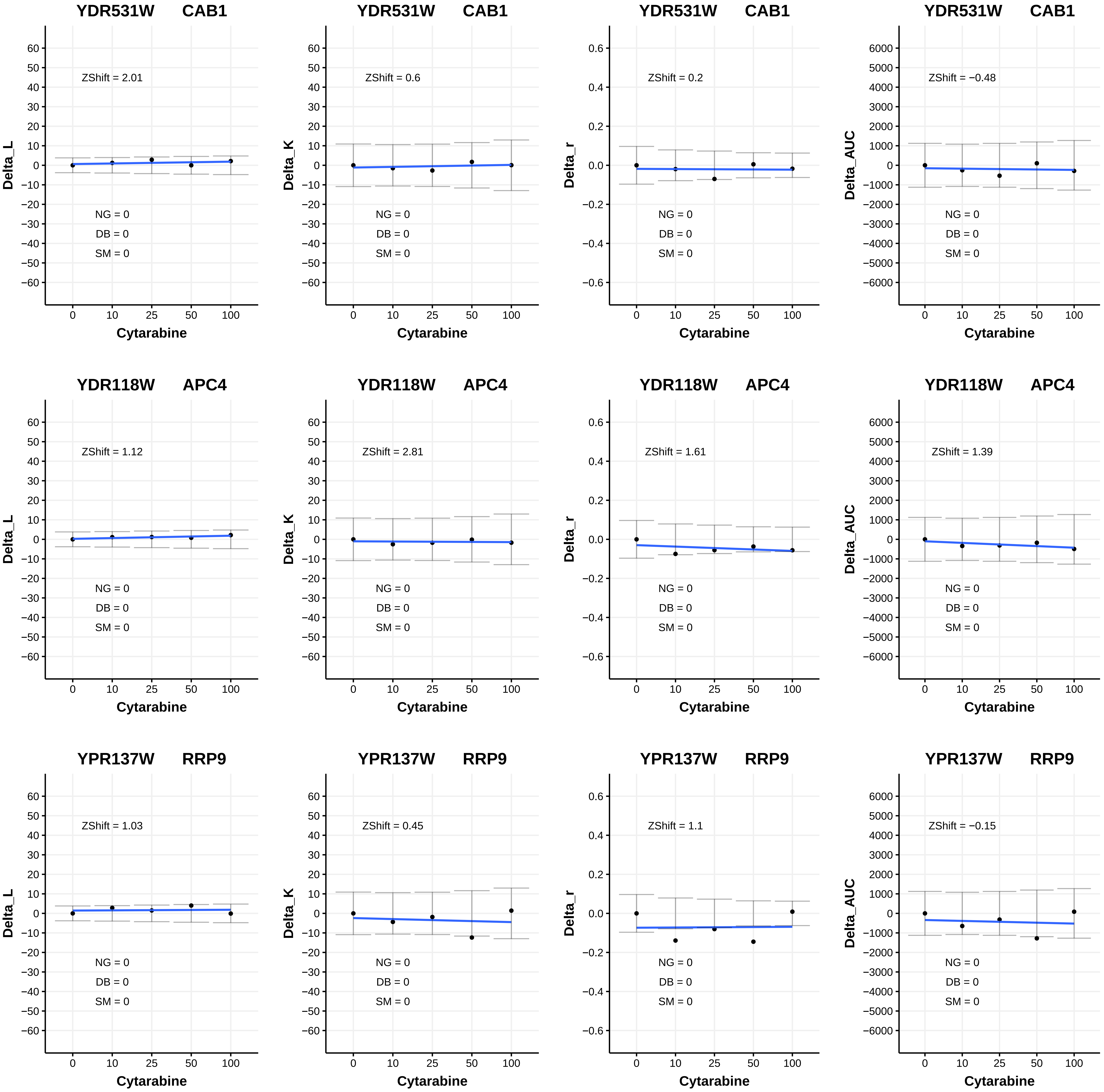
YDR531W CAB1
ZShift = 2.01
NG = 0
DB = 0
SM = 0
Delta_L
Cytarabine
YDR531W CAB1
ZShift = 0.6
NG = 0
DB = 0
SM = 0
Delta_K
Cytarabine
YDR531W CAB1
ZShift = 0.2
NG = 0
DB = 0
SM = 0
Delta_r
Cytarabine
YDR531W CAB1
ZShift = −0.48
NG = 0
DB = 0
SM = 0
Delta_AUC
Cytarabine
YDR118W APC4
ZShift = 1.12
NG = 0
DB = 0
SM = 0
Delta_L
Cytarabine
YDR118W APC4
ZShift = 2.81
NG = 0
DB = 0
SM = 0
Delta_K
Cytarabine
YDR118W APC4
ZShift = 1.61
NG = 0
DB = 0
SM = 0
Delta_r
Cytarabine
YDR118W APC4
ZShift = 1.39
NG = 0
DB = 0
SM = 0
Delta_AUC
Cytarabine
YPR137W RRP9
ZShift = 1.03
NG = 0
DB = 0
SM = 0
Delta_L
Cytarabine
YPR137W RRP9
ZShift = 0.45
NG = 0
DB = 0
SM = 0
Delta_K
Cytarabine
YPR137W RRP9
ZShift = 1.1
NG = 0
DB = 0
SM = 0
Delta_r
Cytarabine
YPR137W RRP9
ZShift = −0.15
NG = 0
DB = 0
SM = 0
Delta_AUC
Cytarabine

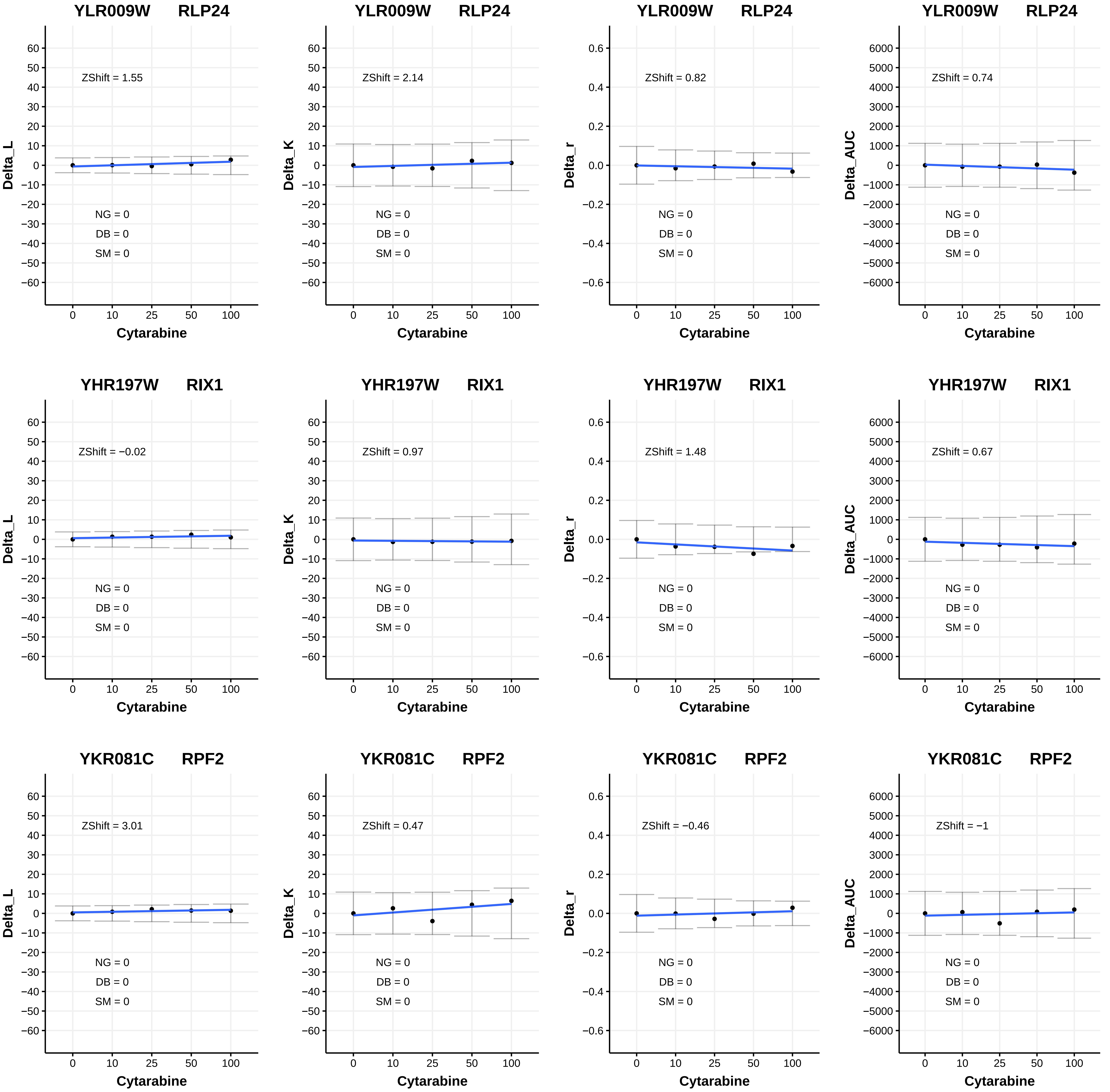
YLR009W RLP24
ZShift = 1.55
NG = 0
DB = 0
SM = 0
Delta_L
Cytarabine
YLR009W RLP24
ZShift = 2.14
NG = 0
DB = 0
SM = 0
Delta_K
Cytarabine
YLR009W RLP24
ZShift = 0.82
NG = 0
DB = 0
SM = 0
Delta_r
Cytarabine
YLR009W RLP24
ZShift = 0.74
NG = 0
DB = 0
SM = 0
Delta_AUC
Cytarabine
YHR197W RIX1
ZShift = −0.02
NG = 0
DB = 0
SM = 0
Delta_L
Cytarabine
YHR197W RIX1
ZShift = 0.97
NG = 0
DB = 0
SM = 0
Delta_K
Cytarabine
YHR197W RIX1
ZShift = 1.48
NG = 0
DB = 0
SM = 0
Delta_r
Cytarabine
YHR197W RIX1
ZShift = 0.67
NG = 0
DB = 0
SM = 0
Delta_AUC
Cytarabine
YKR081C RPF2
ZShift = 3.01
NG = 0
DB = 0
SM = 0
Delta_L
Cytarabine
YKR081C RPF2
ZShift = 0.47
NG = 0
DB = 0
SM = 0
Delta_K
Cytarabine
YKR081C RPF2
ZShift = −0.46
NG = 0
DB = 0
SM = 0
Delta_r
Cytarabine
YKR081C RPF2
ZShift = −1
NG = 0
DB = 0
SM = 0
Delta_AUC
Cytarabine

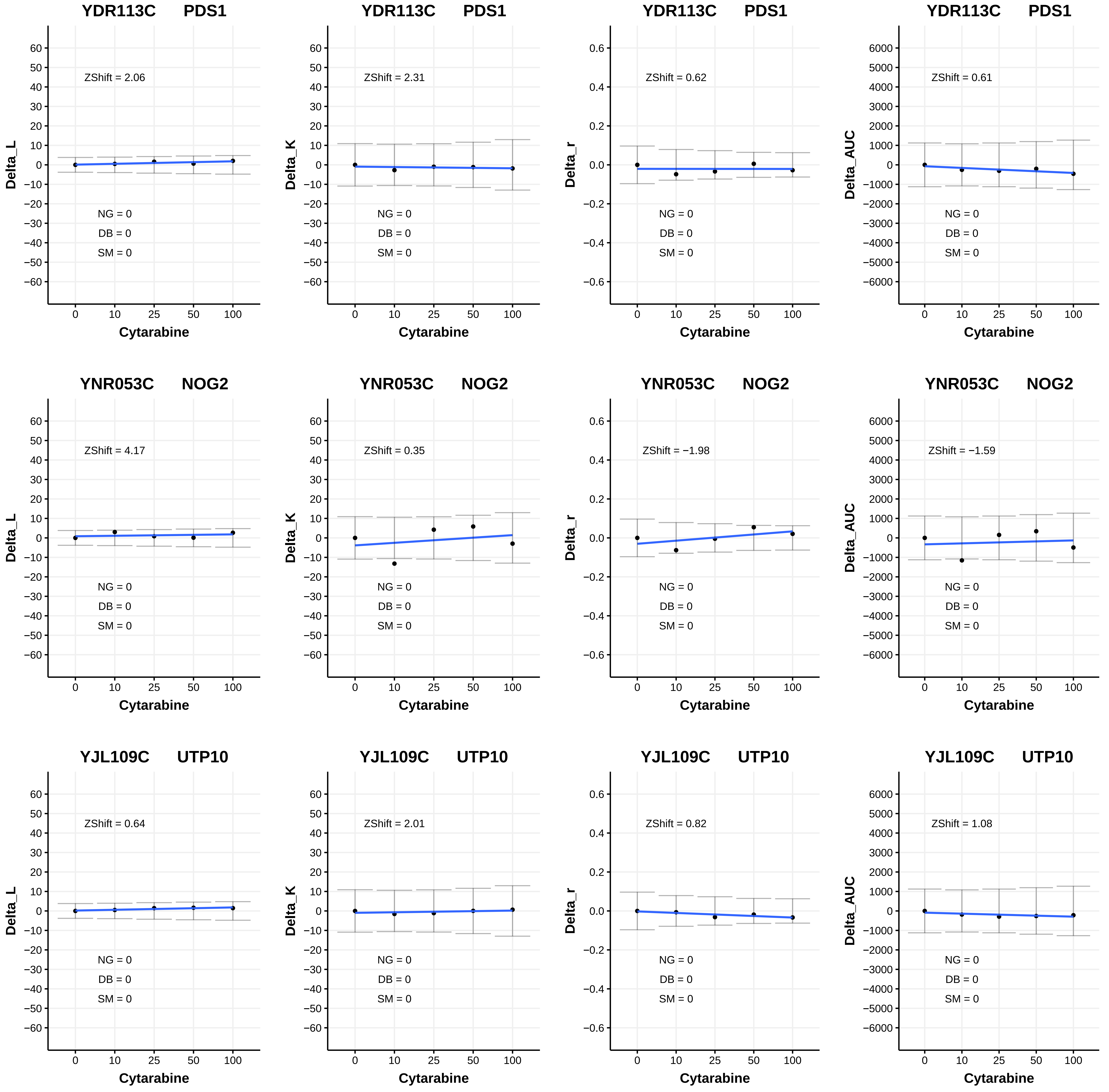
YDR113C PDS1
ZShift = 2.06
ZShift = 2.31
ZShift = 0.62
ZShift = 0.61
YNR053C NOG2
ZShift = 4.17
ZShift = 0.35
ZShift = −1.98
ZShift = −1.59
YJL109C UTP10
ZShift = 0.64
ZShift = 2.01
ZShift = 0.82
ZShift = 1.08
NG = 0
DB = 0
SM = 0
Delta_L
Delta_K
Delta_r
Delta_AUC
Cytarabine
0 10 25 50 100

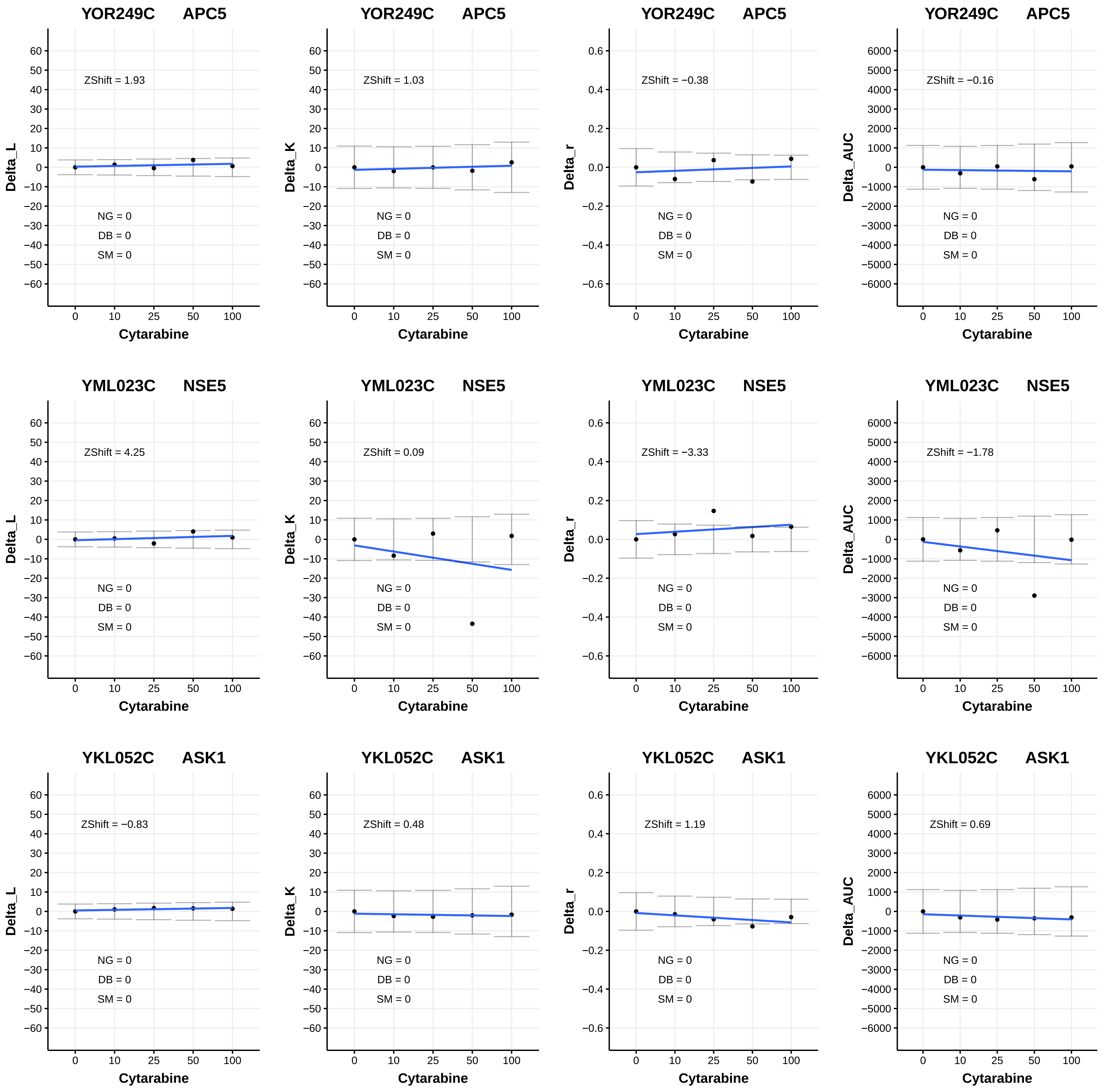
YOR249C APC5
ZShift = 1.93
NG = 0
DB = 0
SM = 0
Delta_L
Cytarabine
YOR249C APC5
ZShift = 1.03
NG = 0
DB = 0
SM = 0
Delta_K
Cytarabine
YOR249C APC5
ZShift = −0.38
NG = 0
DB = 0
SM = 0
Delta_r
Cytarabine
YOR249C APC5
ZShift = −0.16
NG = 0
DB = 0
SM = 0
Delta_AUC
Cytarabine
YML023C NSE5
ZShift = 4.25
NG = 0
DB = 0
SM = 0
Delta_L
Cytarabine
YML023C NSE5
ZShift = 0.09
NG = 0
DB = 0
SM = 0
Delta_K
Cytarabine
YML023C NSE5
ZShift = −3.33
NG = 0
DB = 0
SM = 0
Delta_r
Cytarabine
YML023C NSE5
ZShift = −1.78
NG = 0
DB = 0
SM = 0
Delta_AUC
Cytarabine
YKL052C ASK1
ZShift = −0.83
NG = 0
DB = 0
SM = 0
Delta_L
Cytarabine
YKL052C ASK1
ZShift = 0.48
NG = 0
DB = 0
SM = 0
Delta_K
Cytarabine
YKL052C ASK1
ZShift = 1.19
NG = 0
DB = 0
SM = 0
Delta_r
Cytarabine
YKL052C ASK1
ZShift = 0.69
NG = 0
DB = 0
SM = 0
Delta_AUC
Cytarabine
0
10
25
50
100

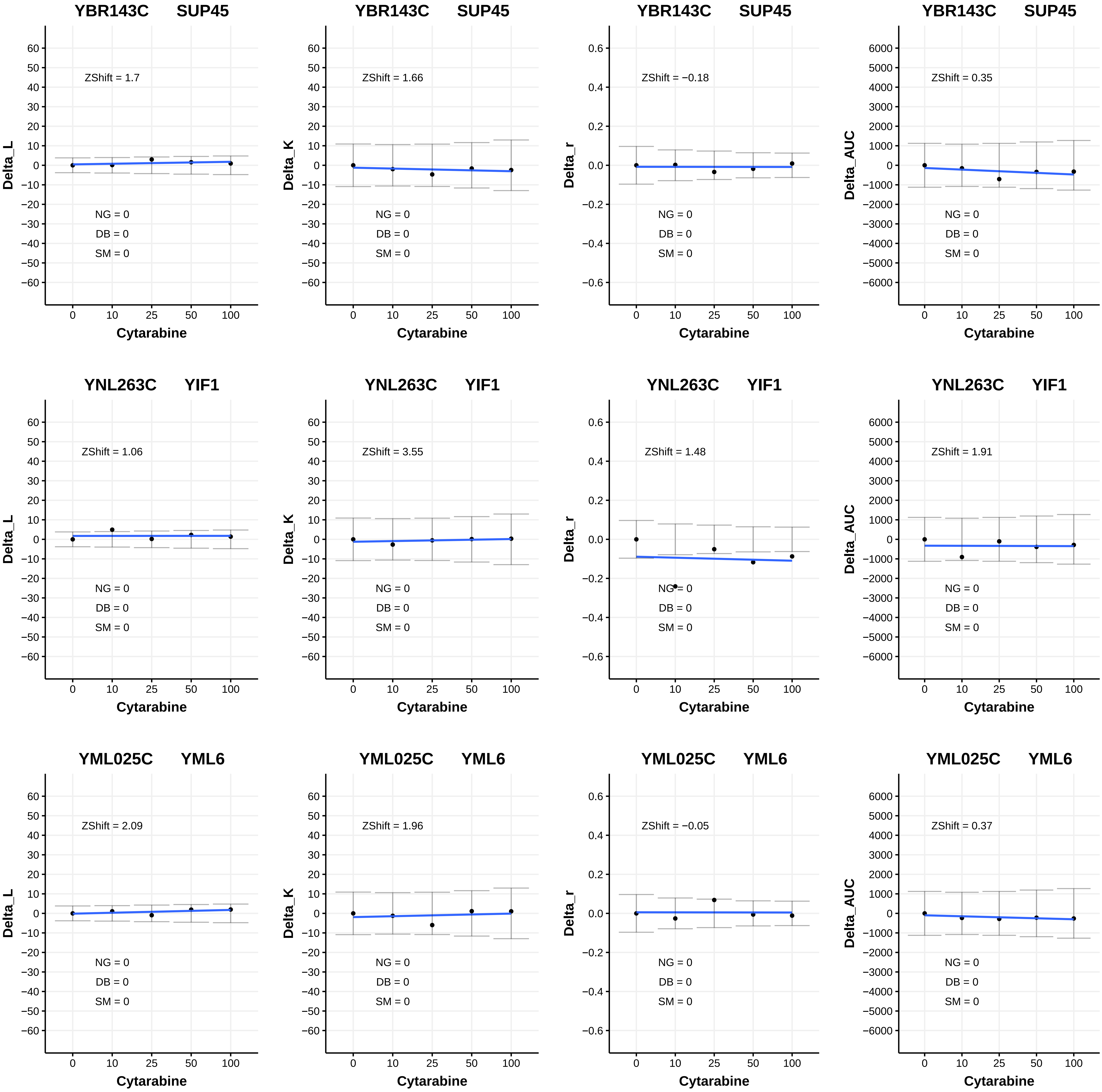
YBR143C SUP45
ZShift = 1.7
Delta_L
NG = 0
DB = 0
SM = 0
Cytarabine
YBR143C SUP45
ZShift = 1.66
Delta_K
NG = 0
DB = 0
SM = 0
Cytarabine
YBR143C SUP45
ZShift = −0.18
Delta_r
NG = 0
DB = 0
SM = 0
Cytarabine
YBR143C SUP45
ZShift = 0.35
Delta_AUC
NG = 0
DB = 0
SM = 0
Cytarabine
YNL263C YIF1
ZShift = 1.06
Delta_L
NG = 0
DB = 0
SM = 0
Cytarabine
YNL263C YIF1
ZShift = 3.55
Delta_K
NG = 0
DB = 0
SM = 0
Cytarabine
YNL263C YIF1
ZShift = 1.48
Delta_r
NG = 0
DB = 0
SM = 0
Cytarabine
YNL263C YIF1
ZShift = 1.91
Delta_AUC
NG = 0
DB = 0
SM = 0
Cytarabine
YML025C YML6
ZShift = 2.09
Delta_L
NG = 0
DB = 0
SM = 0
Cytarabine
YML025C YML6
ZShift = 1.96
Delta_K
NG = 0
DB = 0
SM = 0
Cytarabine
YML025C YML6
ZShift = −0.05
Delta_r
NG = 0
DB = 0
SM = 0
Cytarabine
YML025C YML6
ZShift = 0.37
Delta_AUC
NG = 0
DB = 0
SM = 0
Cytarabine

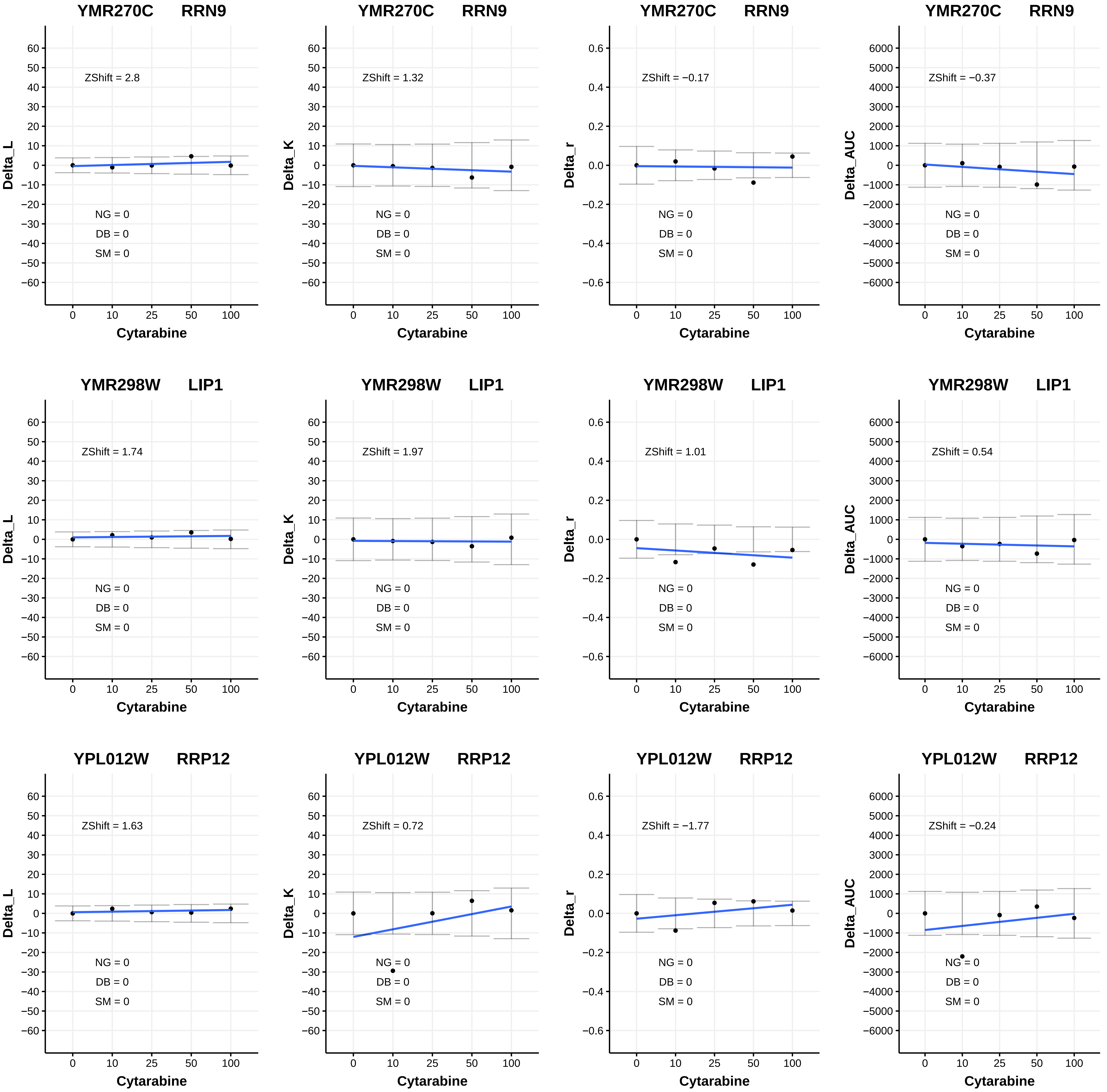

YMR270C RRN9
ZShift = 2.8
Delta_L
NG = 0
DB = 0
SM = 0
Cytarabine
YMR270C RRN9
ZShift = 1.32
Delta_K
NG = 0
DB = 0
SM = 0
Cytarabine
YMR270C RRN9
ZShift = −0.17
Delta_r
NG = 0
DB = 0
SM = 0
Cytarabine
YMR270C RRN9
ZShift = −0.37
Delta_AUC
NG = 0
DB = 0
SM = 0
Cytarabine
YMR298W LIP1
ZShift = 1.74
Delta_L
NG = 0
DB = 0
SM = 0
Cytarabine
YMR298W LIP1
ZShift = 1.97
Delta_K
NG = 0
DB = 0
SM = 0
Cytarabine
YMR298W LIP1
ZShift = 1.01
Delta_r
NG = 0
DB = 0
SM = 0
Cytarabine
YMR298W LIP1
ZShift = 0.54
Delta_AUC
NG = 0
DB = 0
SM = 0
Cytarabine
YPL012W RRP12
ZShift = 1.63
Delta_L
NG = 0
DB = 0
SM = 0
Cytarabine
YPL012W RRP12
ZShift = 0.72
Delta_K
NG = 0
DB = 0
SM = 0
Cytarabine
YPL012W RRP12
ZShift = −1.77
Delta_r
NG = 0
DB = 0
SM = 0
Cytarabine
YPL012W RRP12
ZShift = −0.24
Delta_AUC
NG = 0
DB = 0
SM = 0
Cytarabine

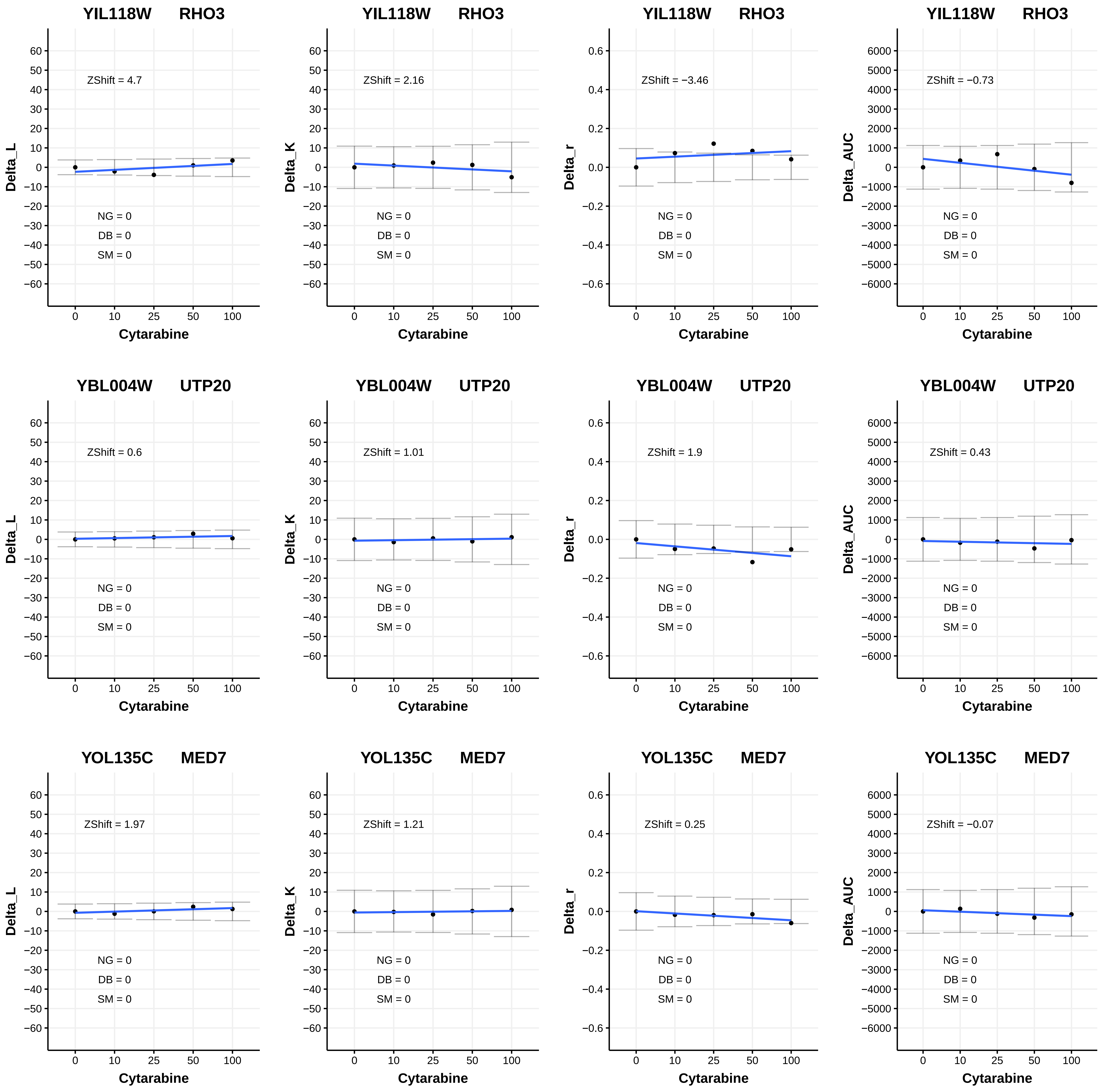

YIL118W RHO3
ZShift = 4.7
NG = 0
DB = 0
SM = 0
Delta_L
Cytarabine
YIL118W RHO3
ZShift = 2.16
NG = 0
DB = 0
SM = 0
Delta_K
Cytarabine
YIL118W RHO3
ZShift = −3.46
NG = 0
DB = 0
SM = 0
Delta_r
Cytarabine
YIL118W RHO3
ZShift = −0.73
NG = 0
DB = 0
SM = 0
Delta_AUC
Cytarabine
YBL004W UTP20
ZShift = 0.6
NG = 0
DB = 0
SM = 0
Delta_L
Cytarabine
YBL004W UTP20
ZShift = 1.01
NG = 0
DB = 0
SM = 0
Delta_K
Cytarabine
YBL004W UTP20
ZShift = 1.9
NG = 0
DB = 0
SM = 0
Delta_r
Cytarabine
YBL004W UTP20
ZShift = 0.43
NG = 0
DB = 0
SM = 0
Delta_AUC
Cytarabine
YOL135C MED7
ZShift = 1.97
NG = 0
DB = 0
SM = 0
Delta_L
Cytarabine
YOL135C MED7
ZShift = 1.21
NG = 0
DB = 0
SM = 0
Delta_K
Cytarabine
YOL135C MED7
ZShift = 0.25
NG = 0
DB = 0
SM = 0
Delta_r
Cytarabine
YOL135C MED7
ZShift = −0.07
NG = 0
DB = 0
SM = 0
Delta_AUC
Cytarabine

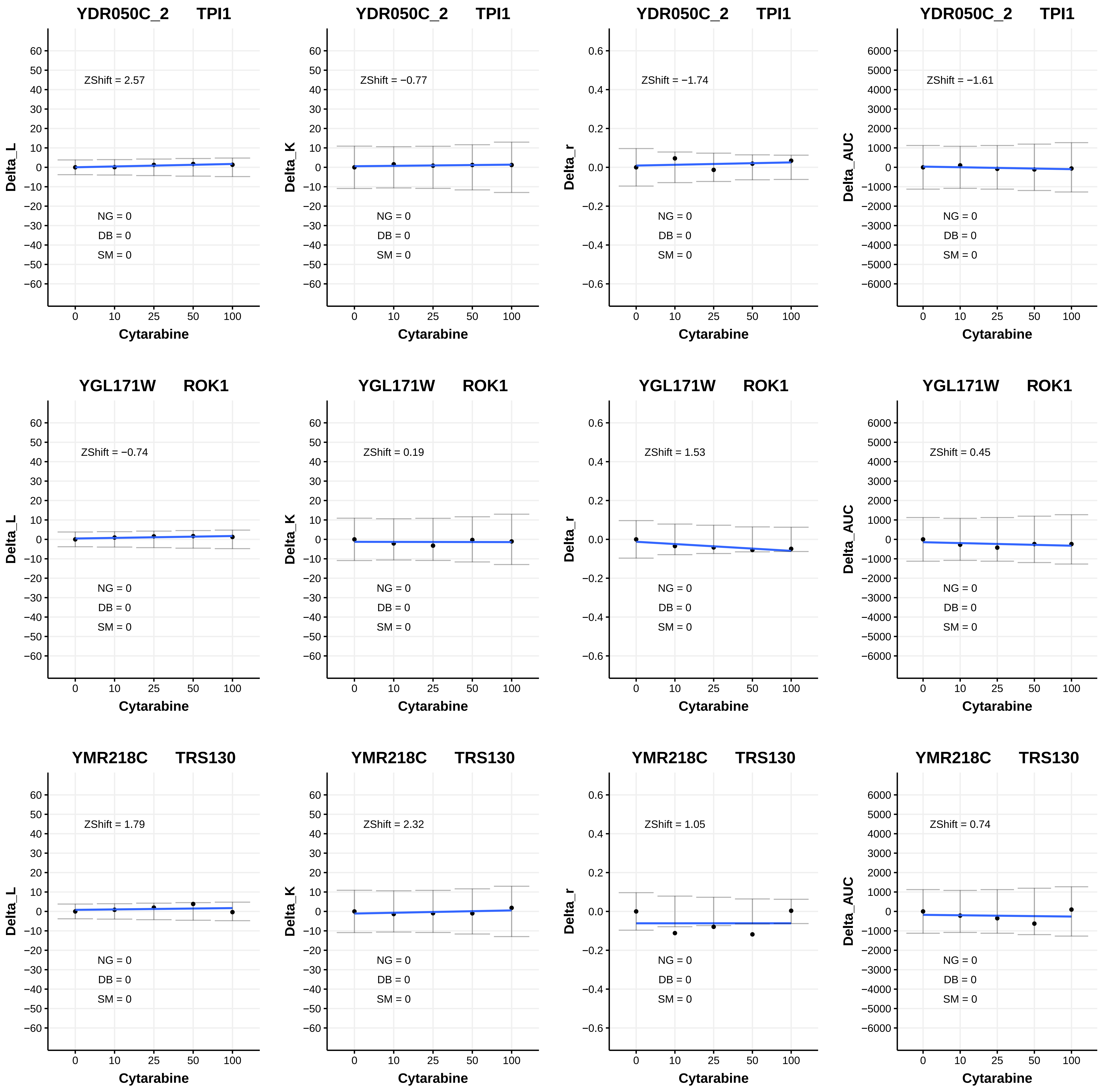

YDR050C_2 TPI1
ZShift = 2.57
ZShift = −0.77
ZShift = −1.74
ZShift = −1.61
YGL171W ROK1
ZShift = −0.74
ZShift = 0.19
ZShift = 1.53
ZShift = 0.45
YMR218C TRS130
ZShift = 1.79
ZShift = 2.32
ZShift = 1.05
ZShift = 0.74
NG = 0
DB = 0
SM = 0
Delta_L
Delta_K
Delta_r
Delta_AUC
Cytarabine
0 10 25 50 100

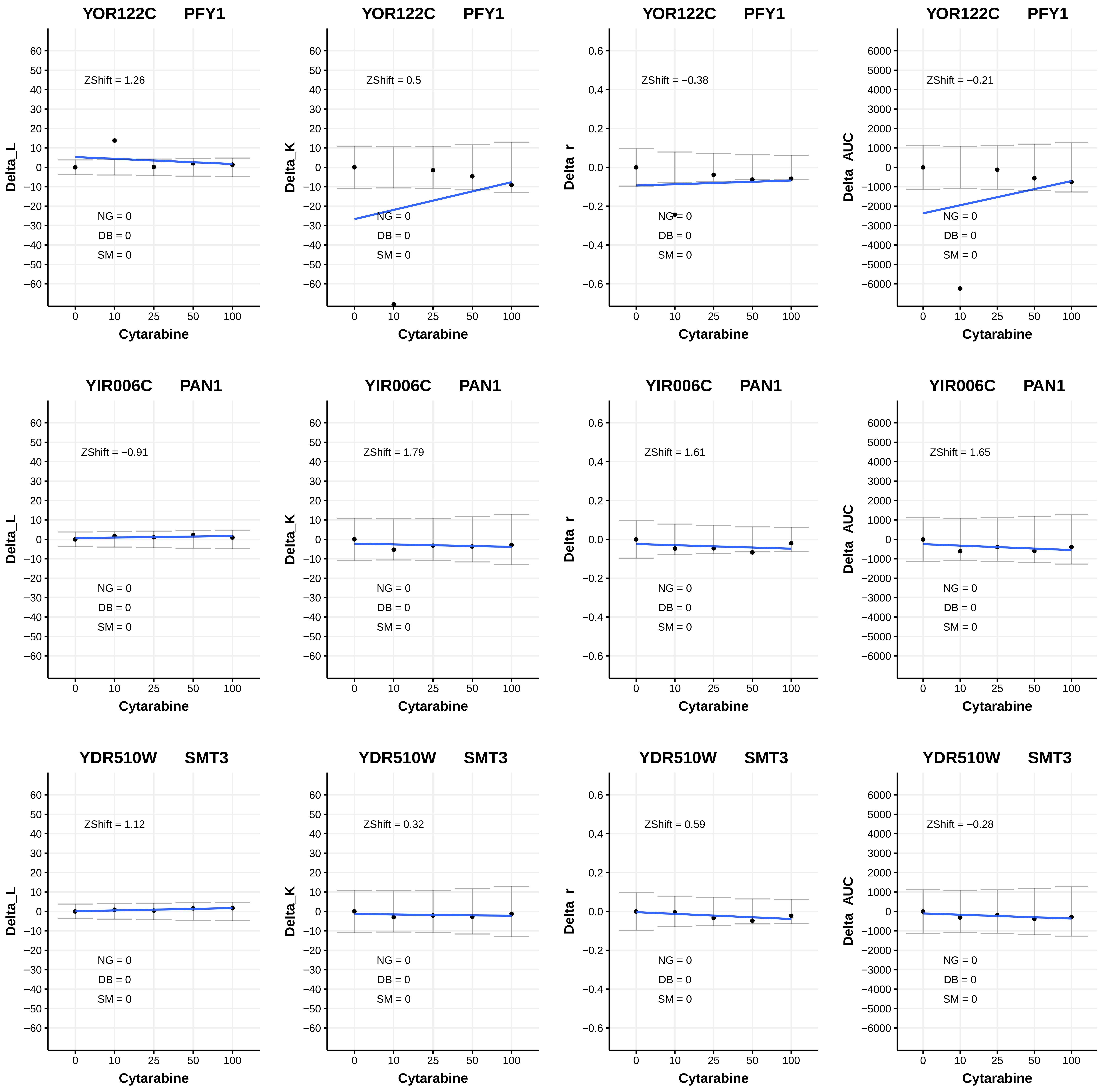

YOR122C PFY1
ZShift = 1.26
NG = 0
DB = 0
SM = 0
Delta_L
Cytarabine
YOR122C PFY1
ZShift = 0.5
NG = 0
DB = 0
SM = 0
Delta_K
Cytarabine
YOR122C PFY1
ZShift = −0.38
NG = 0
DB = 0
SM = 0
Delta_r
Cytarabine
YOR122C PFY1
ZShift = −0.21
NG = 0
DB = 0
SM = 0
Delta_AUC
Cytarabine
YIR006C PAN1
ZShift = −0.91
NG = 0
DB = 0
SM = 0
Delta_L
Cytarabine
YIR006C PAN1
ZShift = 1.79
NG = 0
DB = 0
SM = 0
Delta_K
Cytarabine
YIR006C PAN1
ZShift = 1.61
NG = 0
DB = 0
SM = 0
Delta_r
Cytarabine
YIR006C PAN1
ZShift = 1.65
NG = 0
DB = 0
SM = 0
Delta_AUC
Cytarabine
YDR510W SMT3
ZShift = 1.12
NG = 0
DB = 0
SM = 0
Delta_L
Cytarabine
YDR510W SMT3
ZShift = 0.32
NG = 0
DB = 0
SM = 0
Delta_K
Cytarabine
YDR510W SMT3
ZShift = 0.59
NG = 0
DB = 0
SM = 0
Delta_r
Cytarabine
YDR510W SMT3
ZShift = −0.28
NG = 0
DB = 0
SM = 0
Delta_AUC
Cytarabine

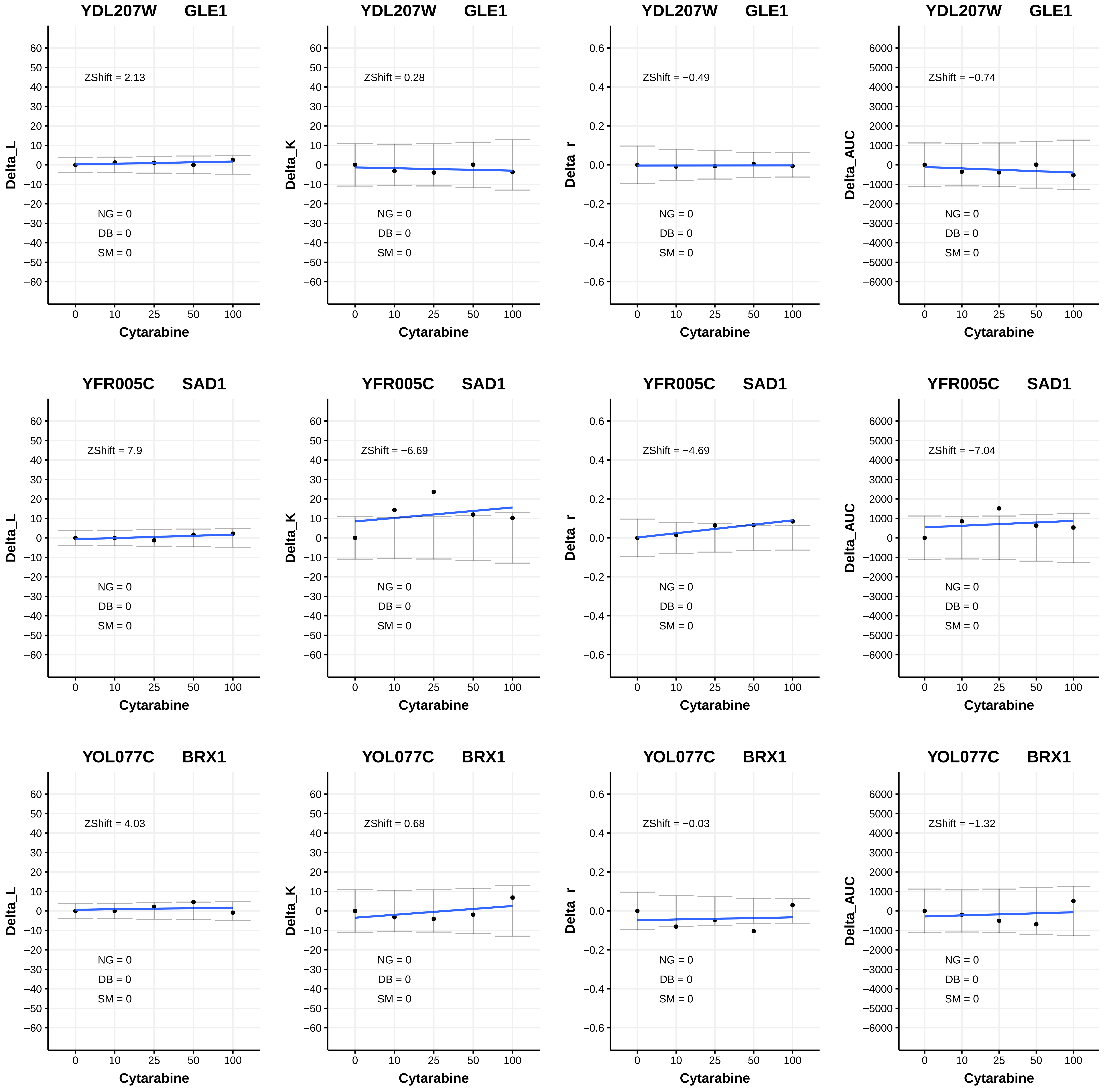
YDL207W GLE1
ZShift = 2.13
NG = 0
DB = 0
SM = 0
Delta_L
Cytarabine
YDL207W GLE1
ZShift = 0.28
NG = 0
DB = 0
SM = 0
Delta_K
Cytarabine
YDL207W GLE1
ZShift = −0.49
NG = 0
DB = 0
SM = 0
Delta_r
Cytarabine
YDL207W GLE1
ZShift = −0.74
NG = 0
DB = 0
SM = 0
Delta_AUC
Cytarabine
YFR005C SAD1
ZShift = 7.9
NG = 0
DB = 0
SM = 0
Delta_L
Cytarabine
YFR005C SAD1
ZShift = −6.69
NG = 0
DB = 0
SM = 0
Delta_K
Cytarabine
YFR005C SAD1
ZShift = −4.69
NG = 0
DB = 0
SM = 0
Delta_r
Cytarabine
YFR005C SAD1
ZShift = −7.04
NG = 0
DB = 0
SM = 0
Delta_AUC
Cytarabine
YOL077C BRX1
ZShift = 4.03
NG = 0
DB = 0
SM = 0
Delta_L
Cytarabine
YOL077C BRX1
ZShift = 0.68
NG = 0
DB = 0
SM = 0
Delta_K
Cytarabine
YOL077C BRX1
ZShift = −0.03
NG = 0
DB = 0
SM = 0
Delta_r
Cytarabine
YOL077C BRX1
ZShift = −1.32
NG = 0
DB = 0
SM = 0
Delta_AUC
Cytarabine

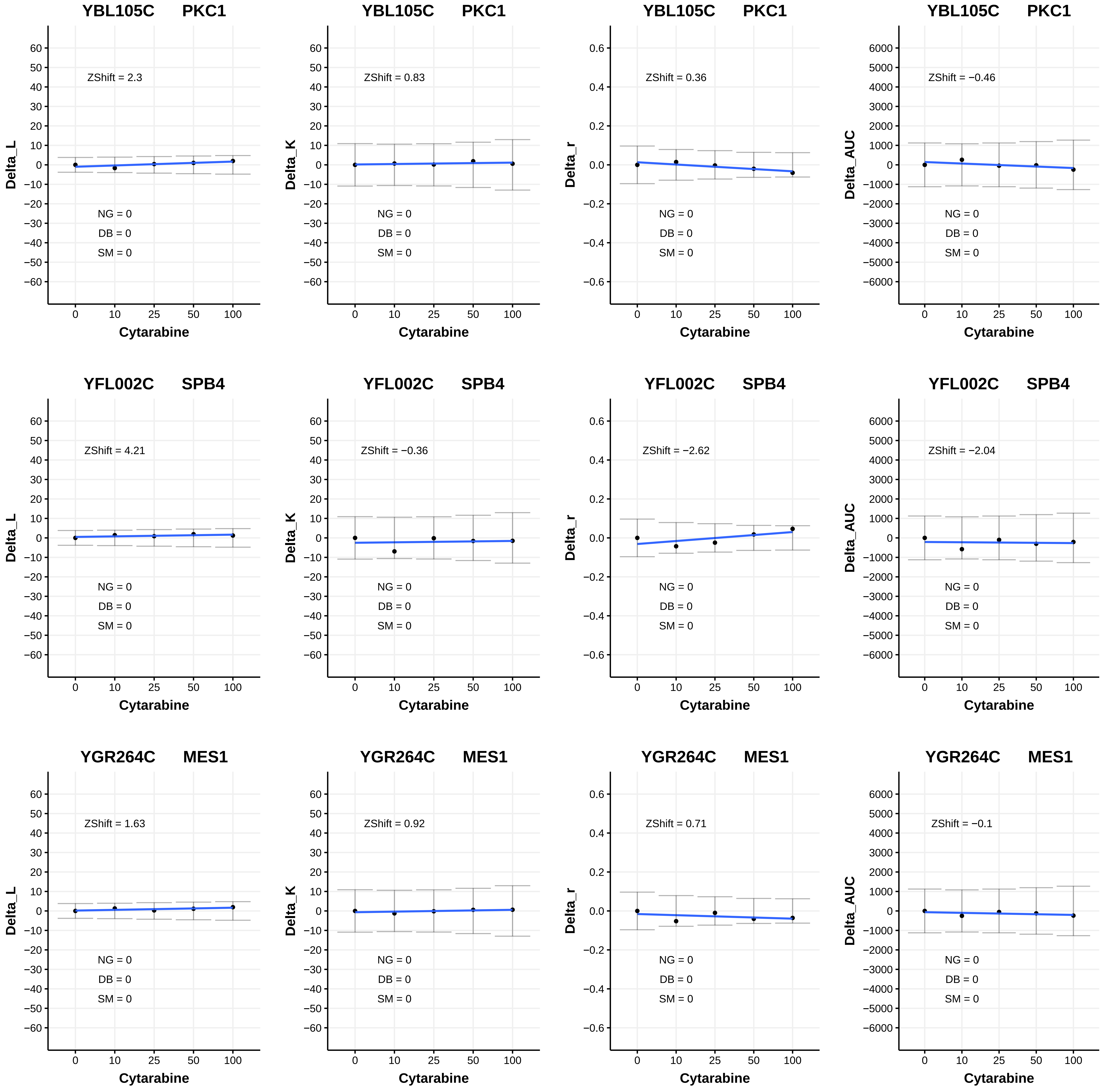
YBL105C PKC1
ZShift = 2.3
ZShift = 0.83
ZShift = 0.36
ZShift = −0.46
YFL002C SPB4
ZShift = 4.21
ZShift = −0.36
ZShift = −2.62
ZShift = −2.04
YGR264C MES1
ZShift = 1.63
ZShift = 0.92
ZShift = 0.71
ZShift = −0.1
NG = 0
DB = 0
SM = 0
Delta_L
Delta_K
Delta_r
Delta_AUC
Cytarabine

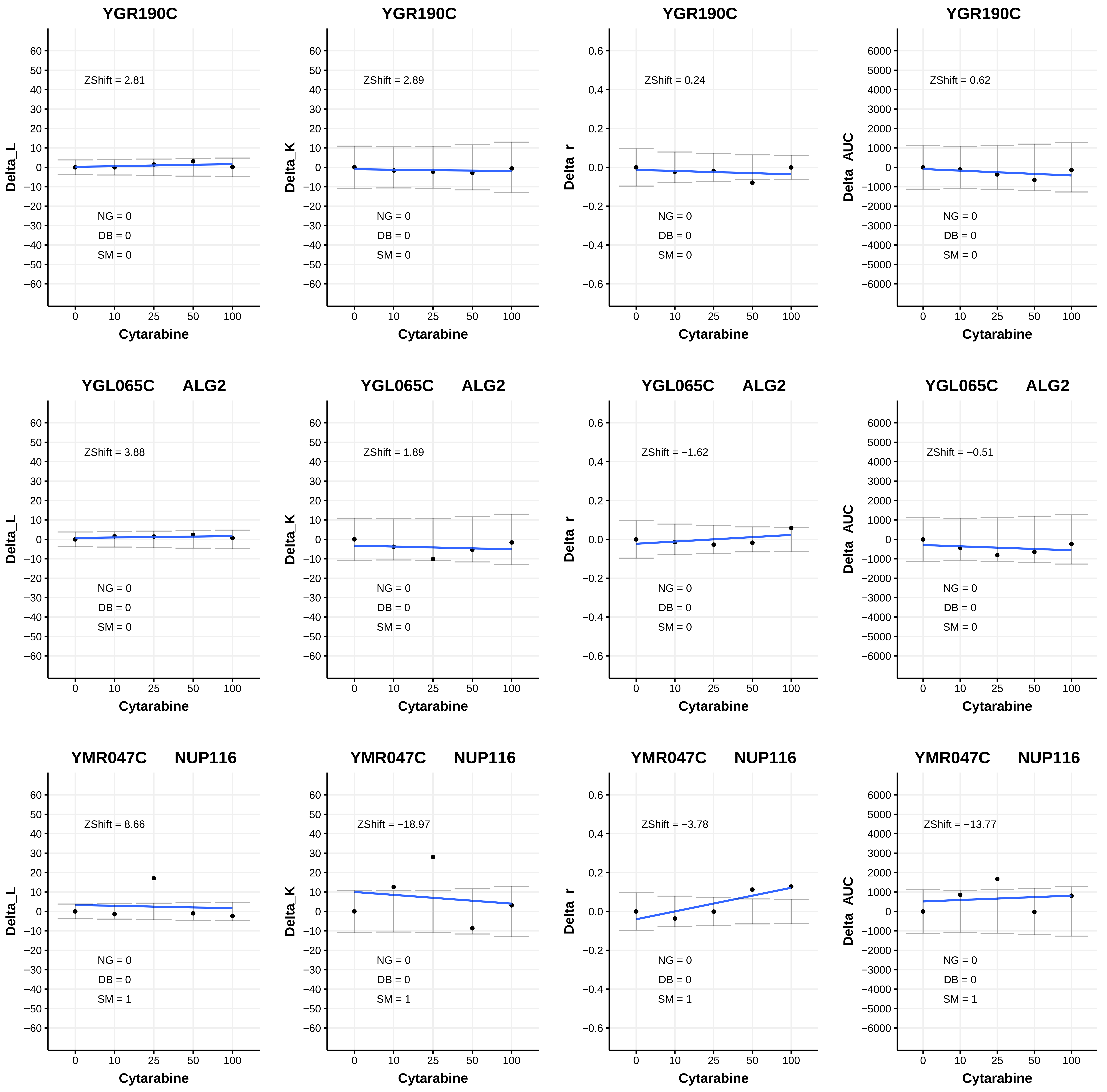
YGR190C
ZShift = 2.81
NG = 0
DB = 0
SM = 0
Delta_L
Cytarabine
YGR190C
ZShift = 2.89
NG = 0
DB = 0
SM = 0
Delta_K
Cytarabine
YGR190C
ZShift = 0.24
NG = 0
DB = 0
SM = 0
Delta_r
Cytarabine
YGR190C
ZShift = 0.62
NG = 0
DB = 0
SM = 0
Delta_AUC
Cytarabine
YGL065C ALG2
ZShift = 3.88
NG = 0
DB = 0
SM = 0
Delta_L
Cytarabine
YGL065C ALG2
ZShift = 1.89
NG = 0
DB = 0
SM = 0
Delta_K
Cytarabine
YGL065C ALG2
ZShift = −1.62
NG = 0
DB = 0
SM = 0
Delta_r
Cytarabine
YGL065C ALG2
ZShift = −0.51
NG = 0
DB = 0
SM = 0
Delta_AUC
Cytarabine
YMR047C NUP116
ZShift = 8.66
NG = 0
DB = 0
SM = 1
Delta_L
Cytarabine
YMR047C NUP116
ZShift = −18.97
NG = 0
DB = 0
SM = 1
Delta_K
Cytarabine
YMR047C NUP116
ZShift = −3.78
NG = 0
DB = 0
SM = 1
Delta_r
Cytarabine
YMR047C NUP116
ZShift = −13.77
NG = 0
DB = 0
SM = 1
Delta_AUC
Cytarabine

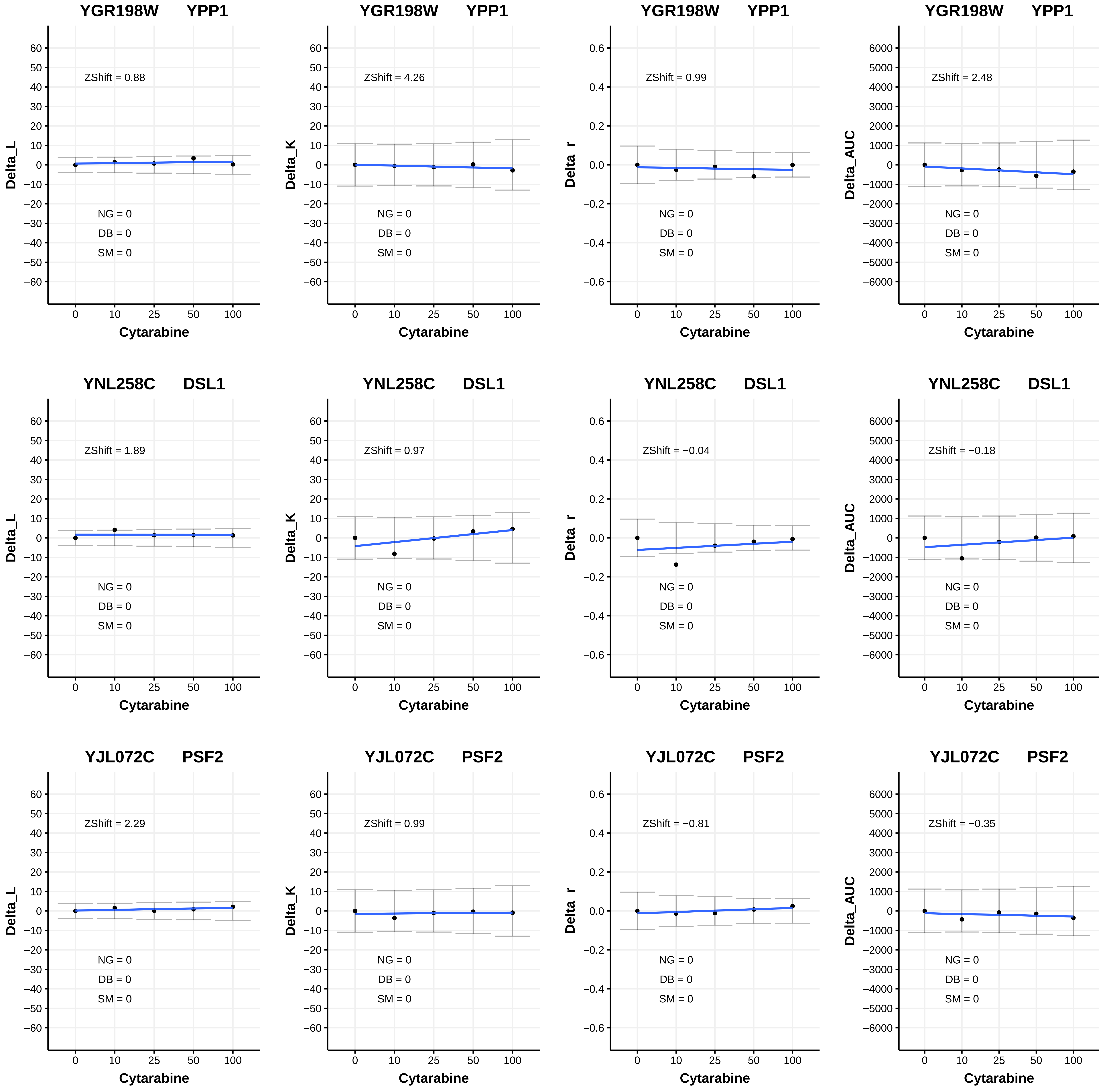
YGR198W YPP1
ZShift = 0.88
Delta_L
NG = 0
DB = 0
SM = 0
Cytarabine
YGR198W YPP1
ZShift = 4.26
Delta_K
NG = 0
DB = 0
SM = 0
Cytarabine
YGR198W YPP1
ZShift = 0.99
Delta_r
NG = 0
DB = 0
SM = 0
Cytarabine
YGR198W YPP1
ZShift = 2.48
Delta_AUC
NG = 0
DB = 0
SM = 0
Cytarabine
YNL258C DSL1
ZShift = 1.89
Delta_L
NG = 0
DB = 0
SM = 0
Cytarabine
YNL258C DSL1
ZShift = 0.97
Delta_K
NG = 0
DB = 0
SM = 0
Cytarabine
YNL258C DSL1
ZShift = −0.04
Delta_r
NG = 0
DB = 0
SM = 0
Cytarabine
YNL258C DSL1
ZShift = −0.18
Delta_AUC
NG = 0
DB = 0
SM = 0
Cytarabine
YJL072C PSF2
ZShift = 2.29
Delta_L
NG = 0
DB = 0
SM = 0
Cytarabine
YJL072C PSF2
ZShift = 0.99
Delta_K
NG = 0
DB = 0
SM = 0
Cytarabine
YJL072C PSF2
ZShift = −0.81
Delta_r
NG = 0
DB = 0
SM = 0
Cytarabine
YJL072C PSF2
ZShift = −0.35
Delta_AUC
NG = 0
DB = 0
SM = 0
Cytarabine

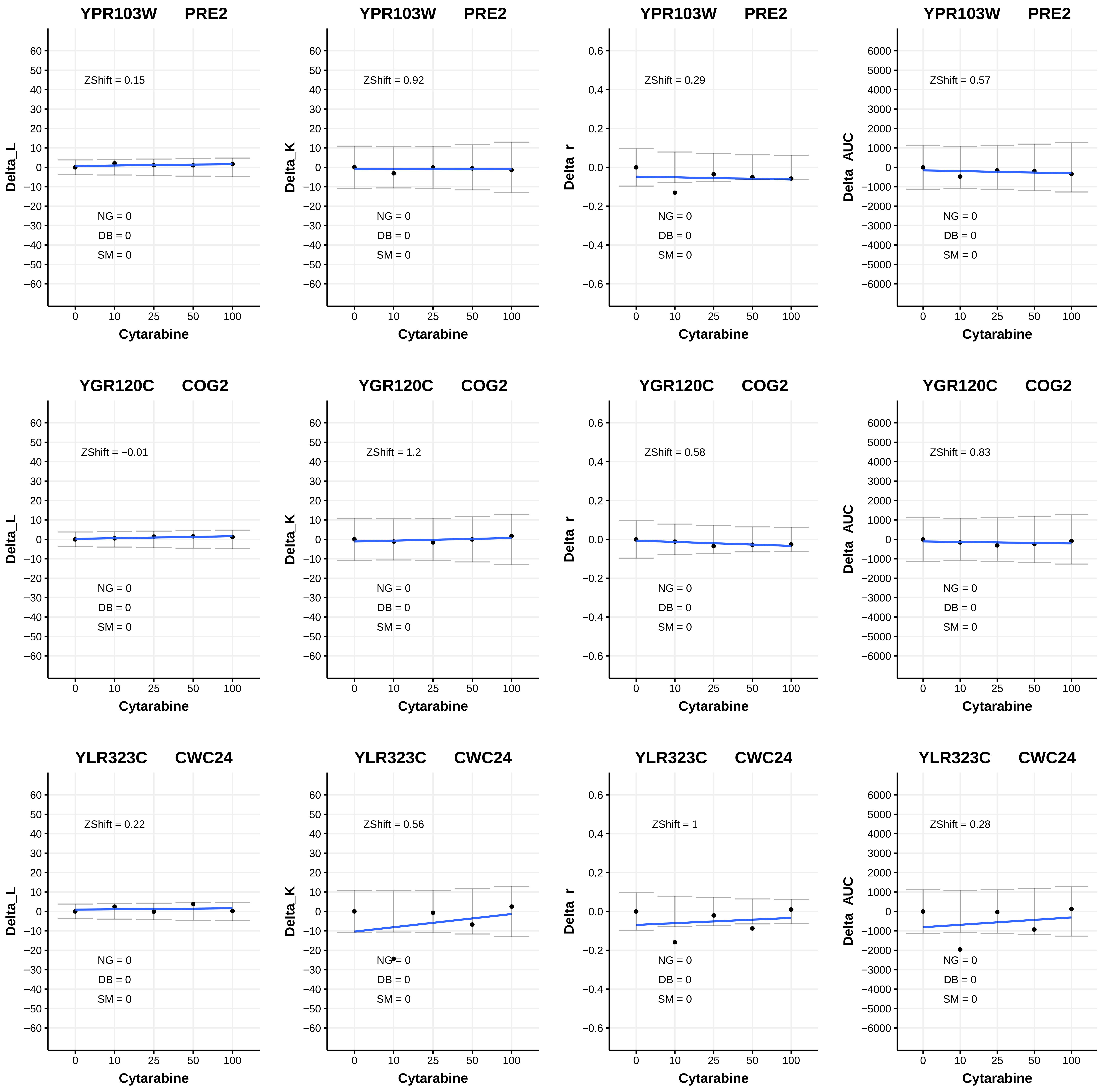
YPR103W PRE2
ZShift = 0.15
Delta_L
NG = 0
DB = 0
SM = 0
Cytarabine
0 10 25 50 100
YPR103W PRE2
ZShift = 0.92
Delta_K
NG = 0
DB = 0
SM = 0
Cytarabine
YPR103W PRE2
ZShift = 0.29
Delta_r
NG = 0
DB = 0
SM = 0
Cytarabine
YPR103W PRE2
ZShift = 0.57
Delta_AUC
NG = 0
DB = 0
SM = 0
Cytarabine
YGR120C COG2
ZShift = −0.01
Delta_L
NG = 0
DB = 0
SM = 0
Cytarabine
YGR120C COG2
ZShift = 1.2
Delta_K
NG = 0
DB = 0
SM = 0
Cytarabine
YGR120C COG2
ZShift = 0.58
Delta_r
NG = 0
DB = 0
SM = 0
Cytarabine
YGR120C COG2
ZShift = 0.83
Delta_AUC
NG = 0
DB = 0
SM = 0
Cytarabine
YLR323C CWC24
ZShift = 0.22
Delta_L
NG = 0
DB = 0
SM = 0
Cytarabine
YLR323C CWC24
ZShift = 0.56
Delta_K
NG = 0
DB = 0
SM = 0
Cytarabine
YLR323C CWC24
ZShift = 1
Delta_r
NG = 0
DB = 0
SM = 0
Cytarabine
YLR323C CWC24
ZShift = 0.28
Delta_AUC
NG = 0
DB = 0
SM = 0
Cytarabine

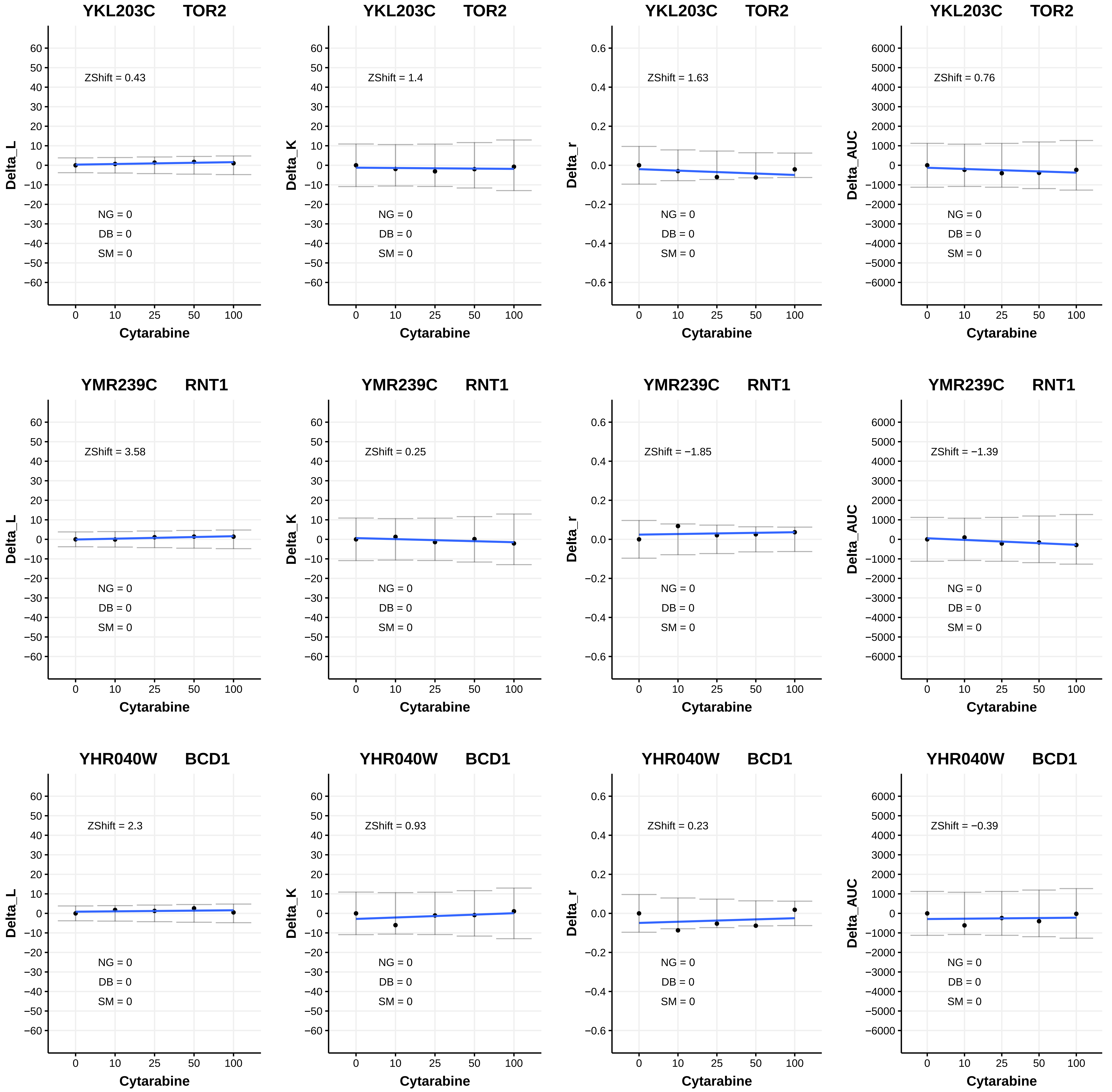
YKL203C TOR2
ZShift = 0.43
NG = 0
DB = 0
SM = 0
Delta_L
Cytarabine
YKL203C TOR2
ZShift = 1.4
NG = 0
DB = 0
SM = 0
Delta_K
Cytarabine
YKL203C TOR2
ZShift = 1.63
NG = 0
DB = 0
SM = 0
Delta_r
Cytarabine
YKL203C TOR2
ZShift = 0.76
NG = 0
DB = 0
SM = 0
Delta_AUC
Cytarabine
YMR239C RNT1
ZShift = 3.58
NG = 0
DB = 0
SM = 0
Delta_L
Cytarabine
YMR239C RNT1
ZShift = 0.25
NG = 0
DB = 0
SM = 0
Delta_K
Cytarabine
YMR239C RNT1
ZShift = −1.85
NG = 0
DB = 0
SM = 0
Delta_r
Cytarabine
YMR239C RNT1
ZShift = −1.39
NG = 0
DB = 0
SM = 0
Delta_AUC
Cytarabine
YHR040W BCD1
ZShift = 2.3
NG = 0
DB = 0
SM = 0
Delta_L
Cytarabine
YHR040W BCD1
ZShift = 0.93
NG = 0
DB = 0
SM = 0
Delta_K
Cytarabine
YHR040W BCD1
ZShift = 0.23
NG = 0
DB = 0
SM = 0
Delta_r
Cytarabine
YHR040W BCD1
ZShift = −0.39
NG = 0
DB = 0
SM = 0
Delta_AUC
Cytarabine

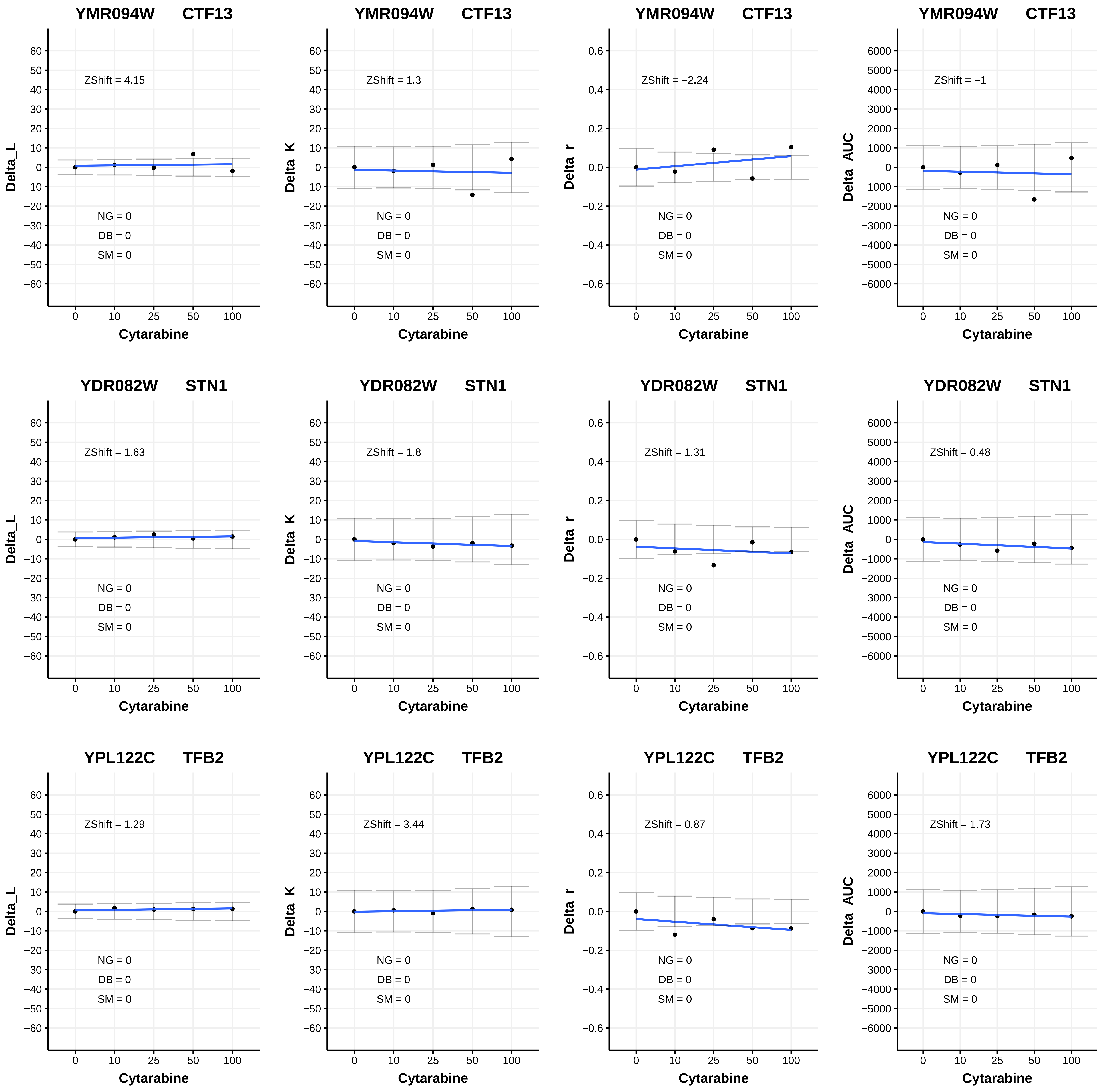

YMR094W CTF13
ZShift = 4.15
Delta_L
ZShift = 1.3
Delta_K
ZShift = −2.24
Delta_r
ZShift = −1
Delta_AUC
YDR082W STN1
ZShift = 1.63
Delta_L
ZShift = 1.8
Delta_K
ZShift = 1.31
Delta_r
ZShift = 0.48
Delta_AUC
YPL122C TFB2
ZShift = 1.29
Delta_L
ZShift = 3.44
Delta_K
ZShift = 0.87
Delta_r
ZShift = 1.73
Delta_AUC
NG = 0
DB = 0
SM = 0
Cytarabine
0 10 25 50 100

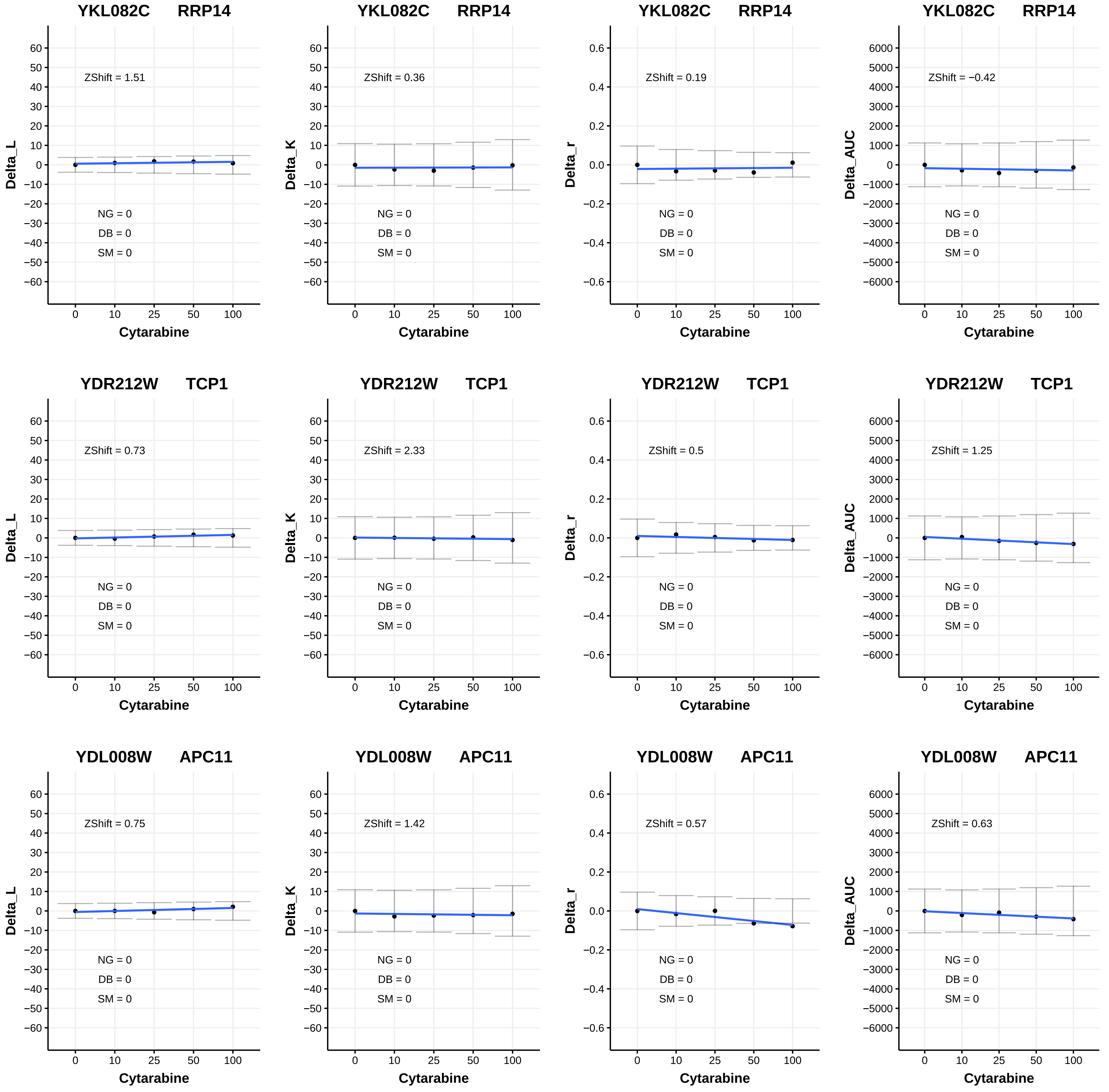
YKL082C RRP14
ZShift = 1.51
ZShift = 0.36
ZShift = 0.19
ZShift = −0.42
YDR212W TCP1
ZShift = 0.73
ZShift = 2.33
ZShift = 0.5
ZShift = 1.25
YDL008W APC11
ZShift = 0.75
ZShift = 1.42
ZShift = 0.57
ZShift = 0.63
NG = 0
DB = 0
SM = 0
Delta_L
Delta_K
Delta_r
Delta_AUC
Cytarabine
0
10
25
50
100

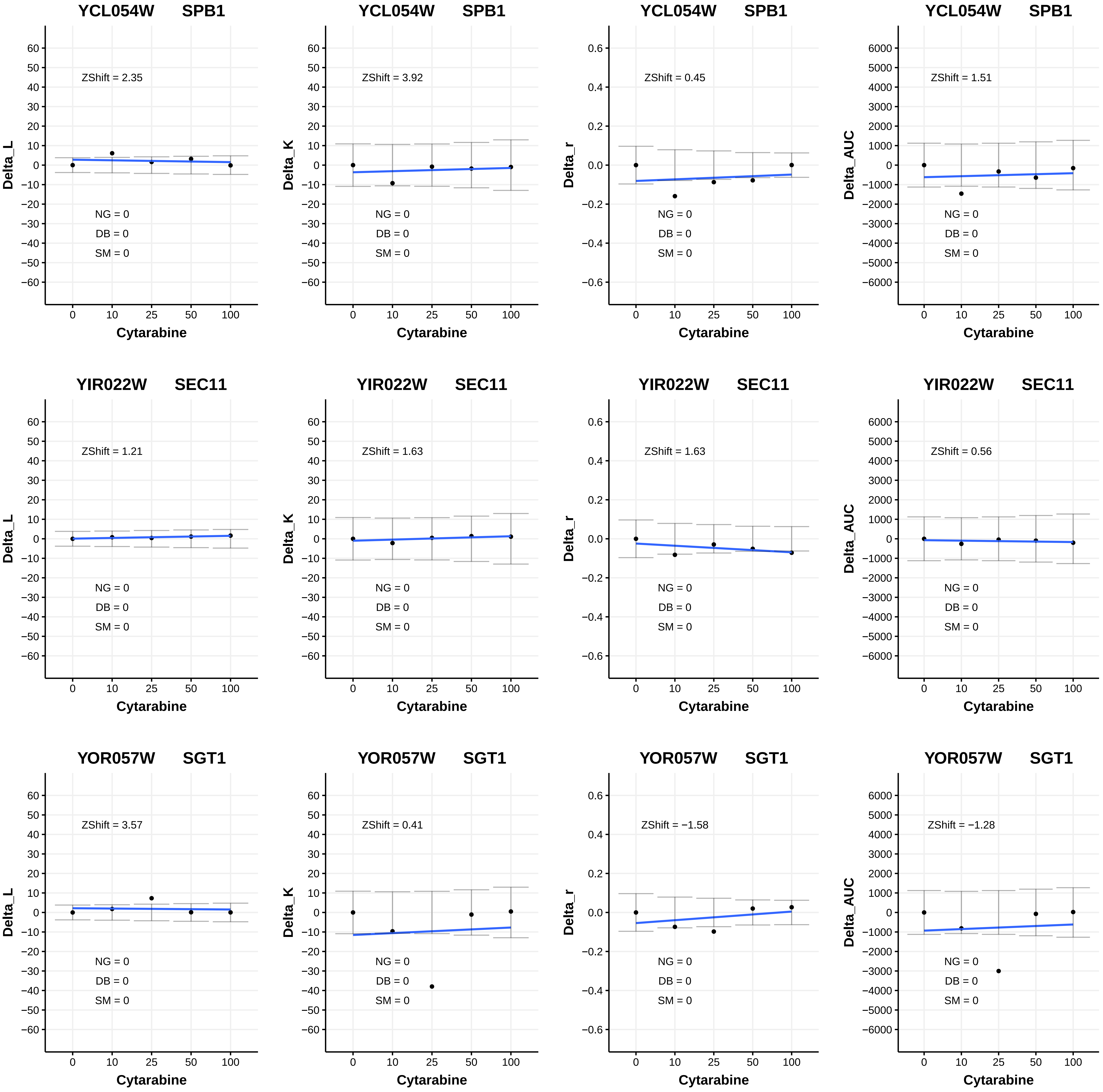

YCL054W SPB1
ZShift = 2.35
NG = 0
DB = 0
SM = 0
Delta_L
Cytarabine
YCL054W SPB1
ZShift = 3.92
NG = 0
DB = 0
SM = 0
Delta_K
Cytarabine
YCL054W SPB1
ZShift = 0.45
NG = 0
DB = 0
SM = 0
Delta_r
Cytarabine
YCL054W SPB1
ZShift = 1.51
NG = 0
DB = 0
SM = 0
Delta_AUC
Cytarabine
YIR022W SEC11
ZShift = 1.21
NG = 0
DB = 0
SM = 0
Delta_L
Cytarabine
YIR022W SEC11
ZShift = 1.63
NG = 0
DB = 0
SM = 0
Delta_K
Cytarabine
YIR022W SEC11
ZShift = 1.63
NG = 0
DB = 0
SM = 0
Delta_r
Cytarabine
YIR022W SEC11
ZShift = 0.56
NG = 0
DB = 0
SM = 0
Delta_AUC
Cytarabine
YOR057W SGT1
ZShift = 3.57
NG = 0
DB = 0
SM = 0
Delta_L
Cytarabine
YOR057W SGT1
ZShift = 0.41
NG = 0
DB = 0
SM = 0
Delta_K
Cytarabine
YOR057W SGT1
ZShift = −1.58
NG = 0
DB = 0
SM = 0
Delta_r
Cytarabine
YOR057W SGT1
ZShift = −1.28
NG = 0
DB = 0
SM = 0
Delta_AUC
Cytarabine

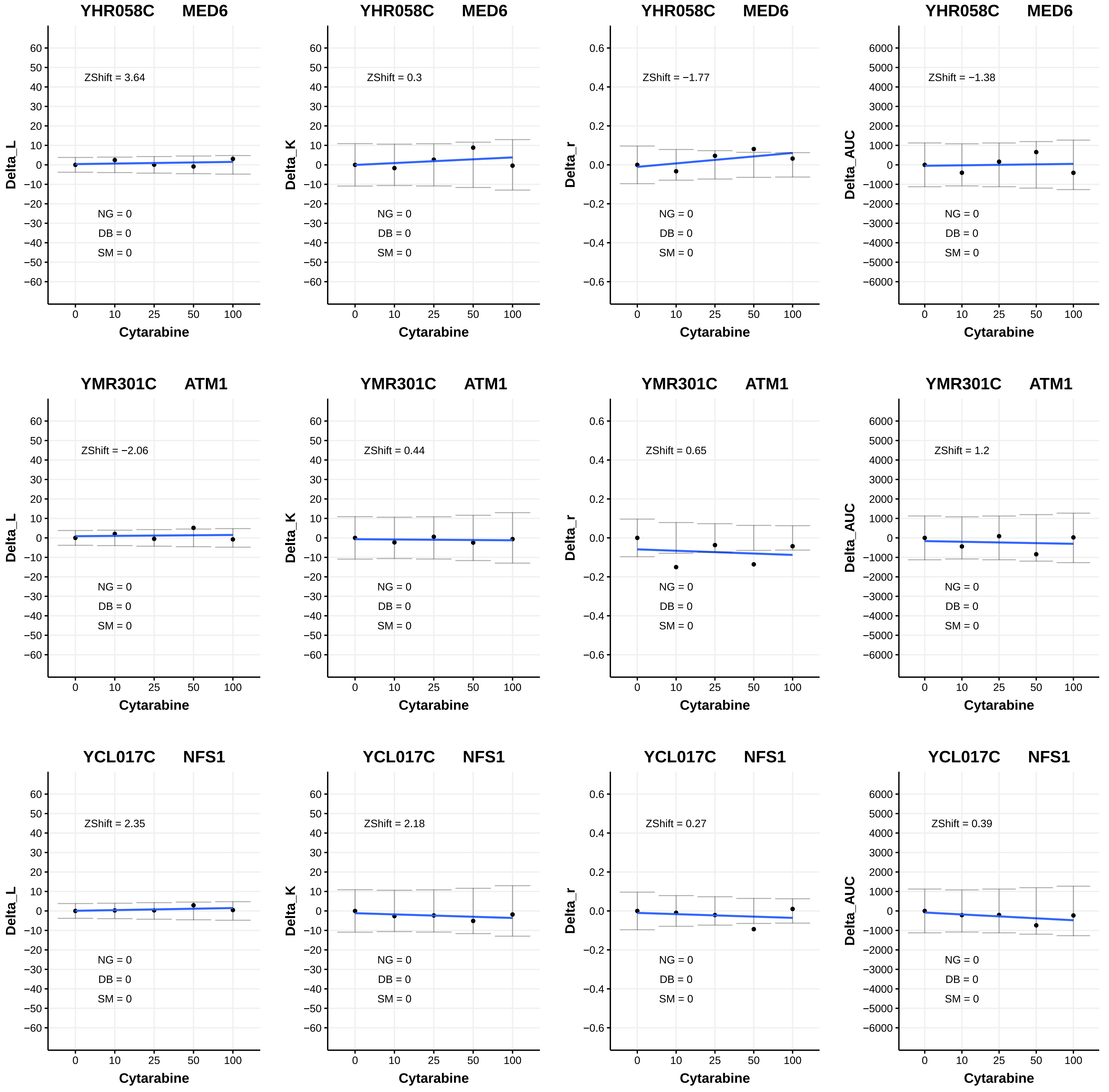
YHR058C MED6
ZShift = 3.64
ZShift = 0.3
ZShift = −1.77
ZShift = −1.38
YMR301C ATM1
ZShift = −2.06
ZShift = 0.44
ZShift = 0.65
ZShift = 1.2
YCL017C NFS1
ZShift = 2.35
ZShift = 2.18
ZShift = 0.27
ZShift = 0.39
NG = 0
DB = 0
SM = 0
Delta_L
Delta_K
Delta_r
Delta_AUC
Cytarabine
0 10 25 50 100

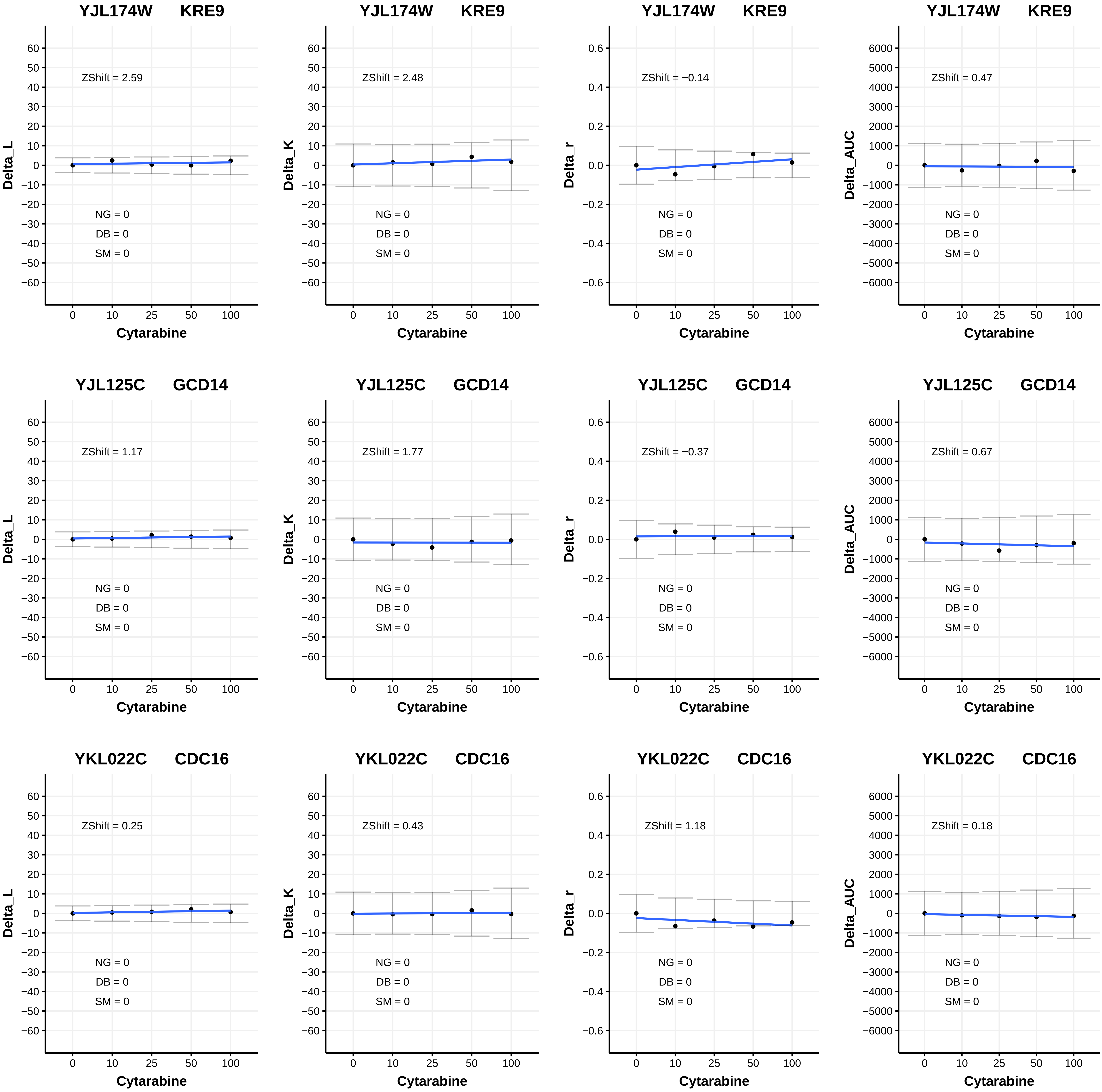
YJL174W KRE9
ZShift = 2.59
NG = 0
DB = 0
SM = 0
Delta_L
Cytarabine
YJL174W KRE9
ZShift = 2.48
NG = 0
DB = 0
SM = 0
Delta_K
Cytarabine
YJL174W KRE9
ZShift = −0.14
NG = 0
DB = 0
SM = 0
Delta_r
Cytarabine
YJL174W KRE9
ZShift = 0.47
NG = 0
DB = 0
SM = 0
Delta_AUC
Cytarabine
YJL125C GCD14
ZShift = 1.17
NG = 0
DB = 0
SM = 0
Delta_L
Cytarabine
YJL125C GCD14
ZShift = 1.77
NG = 0
DB = 0
SM = 0
Delta_K
Cytarabine
YJL125C GCD14
ZShift = −0.37
NG = 0
DB = 0
SM = 0
Delta_r
Cytarabine
YJL125C GCD14
ZShift = 0.67
NG = 0
DB = 0
SM = 0
Delta_AUC
Cytarabine
YKL022C CDC16
ZShift = 0.25
NG = 0
DB = 0
SM = 0
Delta_L
Cytarabine
YKL022C CDC16
ZShift = 0.43
NG = 0
DB = 0
SM = 0
Delta_K
Cytarabine
YKL022C CDC16
ZShift = 1.18
NG = 0
DB = 0
SM = 0
Delta_r
Cytarabine
YKL022C CDC16
ZShift = 0.18
NG = 0
DB = 0
SM = 0
Delta_AUC
Cytarabine

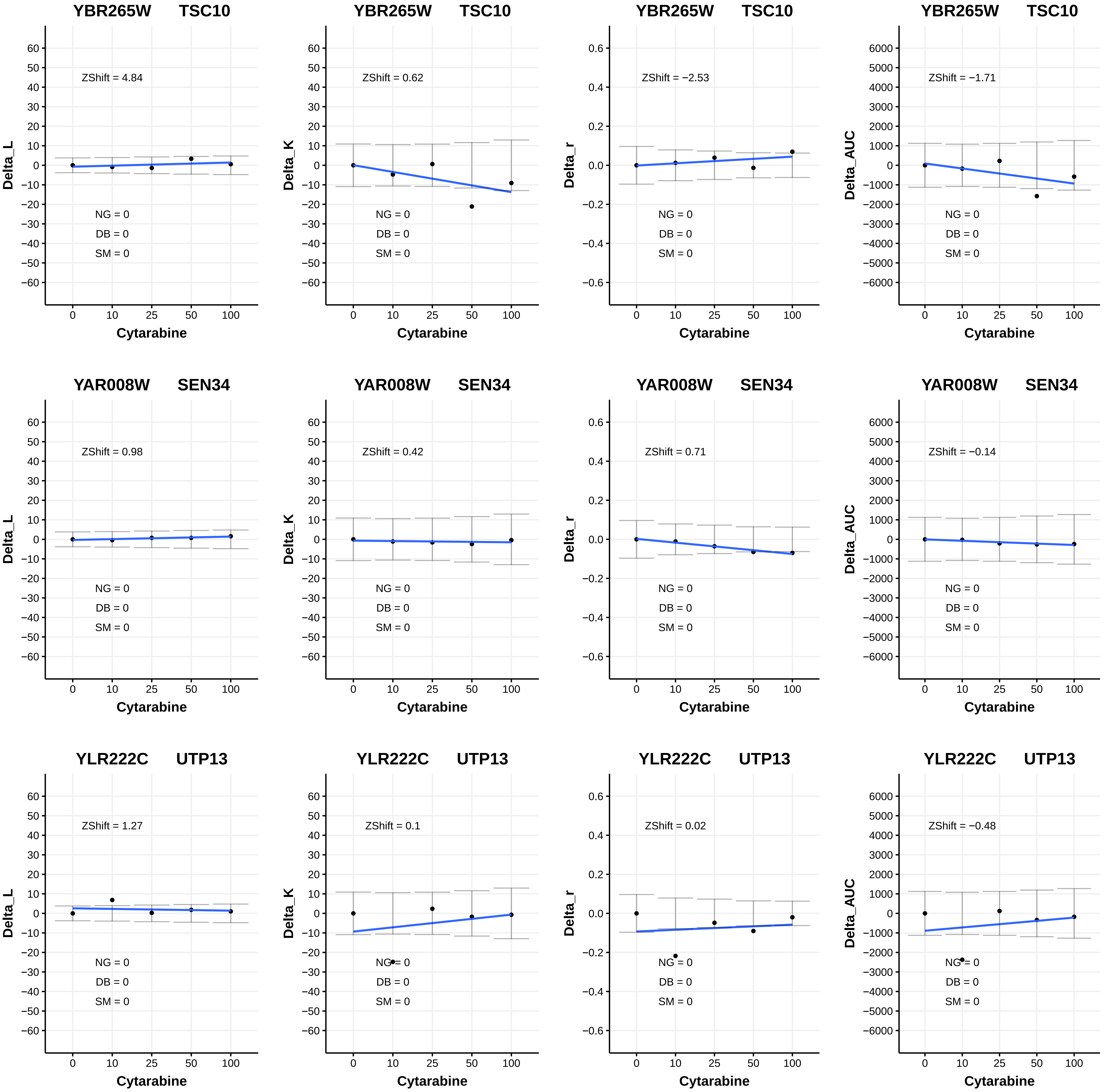

YBR265W TSC10
ZShift = 4.84
ZShift = 0.62
ZShift = −2.53
ZShift = −1.71
YAR008W SEN34
ZShift = 0.98
ZShift = 0.42
ZShift = 0.71
ZShift = −0.14
YLR222C UTP13
ZShift = 1.27
ZShift = 0.1
ZShift = 0.02
ZShift = −0.48
NG = 0
DB = 0
SM = 0
Delta_L
Delta_K
Delta_r
Delta_AUC
Cytarabine

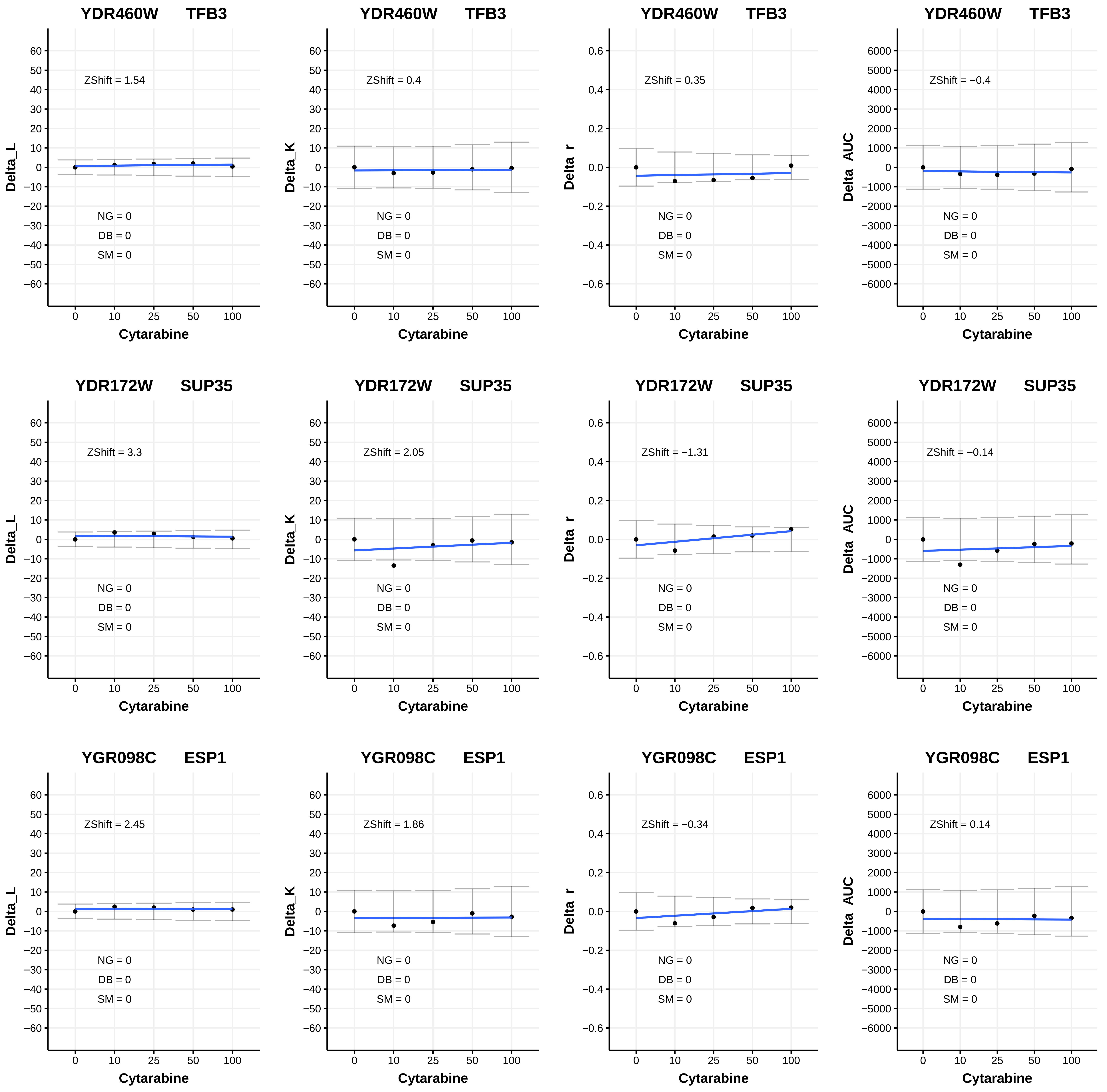
YDR460W TFB3
ZShift = 1.54
NG = 0
DB = 0
SM = 0
Delta_L
Cytarabine
YDR460W TFB3
ZShift = 0.4
NG = 0
DB = 0
SM = 0
Delta_K
Cytarabine
YDR460W TFB3
ZShift = 0.35
NG = 0
DB = 0
SM = 0
Delta_r
Cytarabine
YDR460W TFB3
ZShift = −0.4
NG = 0
DB = 0
SM = 0
Delta_AUC
Cytarabine
YDR172W SUP35
ZShift = 3.3
NG = 0
DB = 0
SM = 0
Delta_L
Cytarabine
YDR172W SUP35
ZShift = 2.05
NG = 0
DB = 0
SM = 0
Delta_K
Cytarabine
YDR172W SUP35
ZShift = −1.31
NG = 0
DB = 0
SM = 0
Delta_r
Cytarabine
YDR172W SUP35
ZShift = −0.14
NG = 0
DB = 0
SM = 0
Delta_AUC
Cytarabine
YGR098C ESP1
ZShift = 2.45
NG = 0
DB = 0
SM = 0
Delta_L
Cytarabine
YGR098C ESP1
ZShift = 1.86
NG = 0
DB = 0
SM = 0
Delta_K
Cytarabine
YGR098C ESP1
ZShift = −0.34
NG = 0
DB = 0
SM = 0
Delta_r
Cytarabine
YGR098C ESP1
ZShift = 0.14
NG = 0
DB = 0
SM = 0
Delta_AUC
Cytarabine

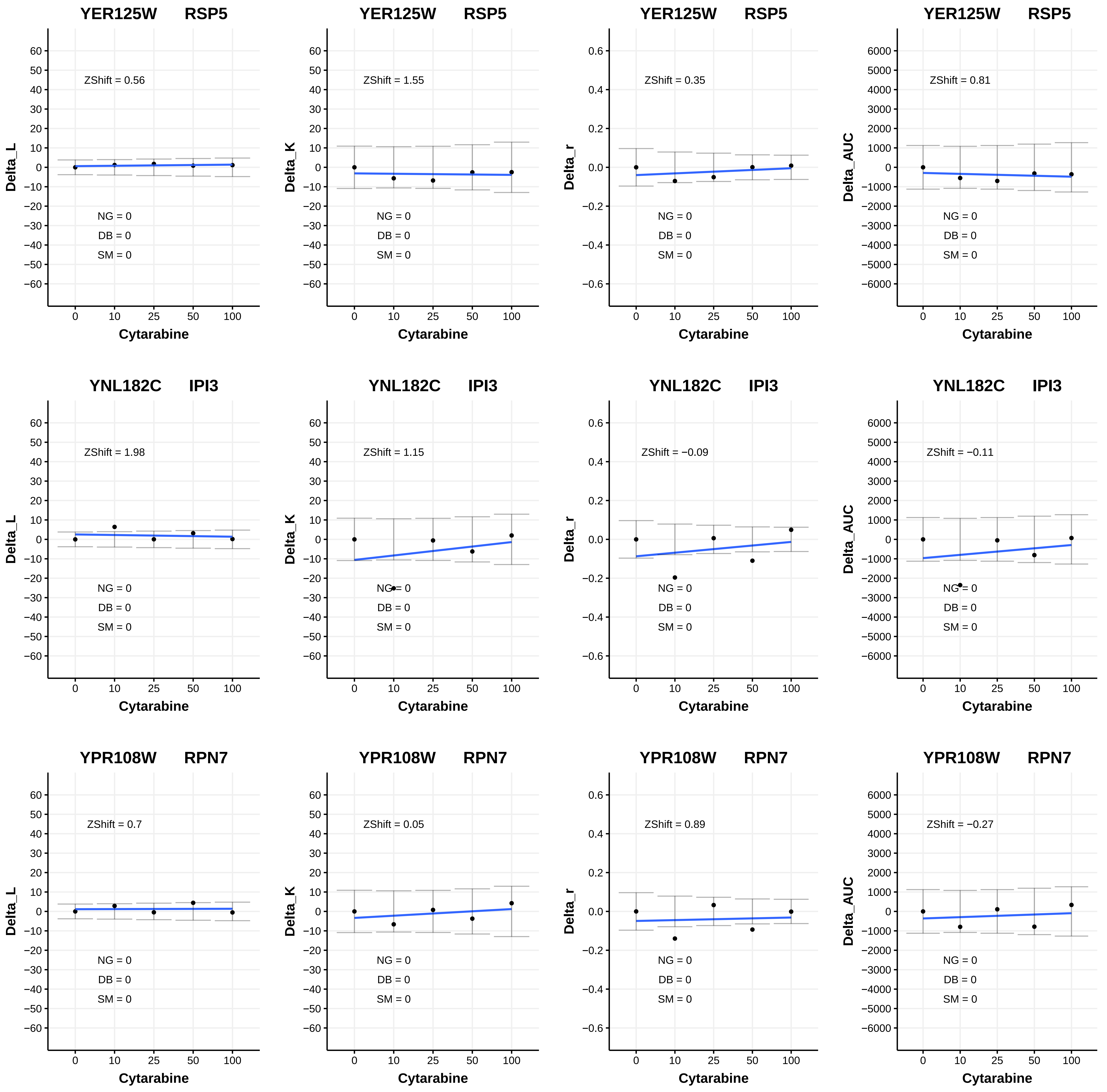

YER125W RSP5
ZShift = 0.56
NG = 0
DB = 0
SM = 0
Delta_L
Cytarabine
YER125W RSP5
ZShift = 1.55
NG = 0
DB = 0
SM = 0
Delta_K
Cytarabine
YER125W RSP5
ZShift = 0.35
NG = 0
DB = 0
SM = 0
Delta_r
Cytarabine
YER125W RSP5
ZShift = 0.81
NG = 0
DB = 0
SM = 0
Delta_AUC
Cytarabine
YNL182C IPI3
ZShift = 1.98
NG = 0
DB = 0
SM = 0
Delta_L
Cytarabine
YNL182C IPI3
ZShift = 1.15
NG = 0
DB = 0
SM = 0
Delta_K
Cytarabine
YNL182C IPI3
ZShift = −0.09
NG = 0
DB = 0
SM = 0
Delta_r
Cytarabine
YNL182C IPI3
ZShift = −0.11
NG = 0
DB = 0
SM = 0
Delta_AUC
Cytarabine
YPR108W RPN7
ZShift = 0.7
NG = 0
DB = 0
SM = 0
Delta_L
Cytarabine
YPR108W RPN7
ZShift = 0.05
NG = 0
DB = 0
SM = 0
Delta_K
Cytarabine
YPR108W RPN7
ZShift = 0.89
NG = 0
DB = 0
SM = 0
Delta_r
Cytarabine
YPR108W RPN7
ZShift = −0.27
NG = 0
DB = 0
SM = 0
Delta_AUC
Cytarabine

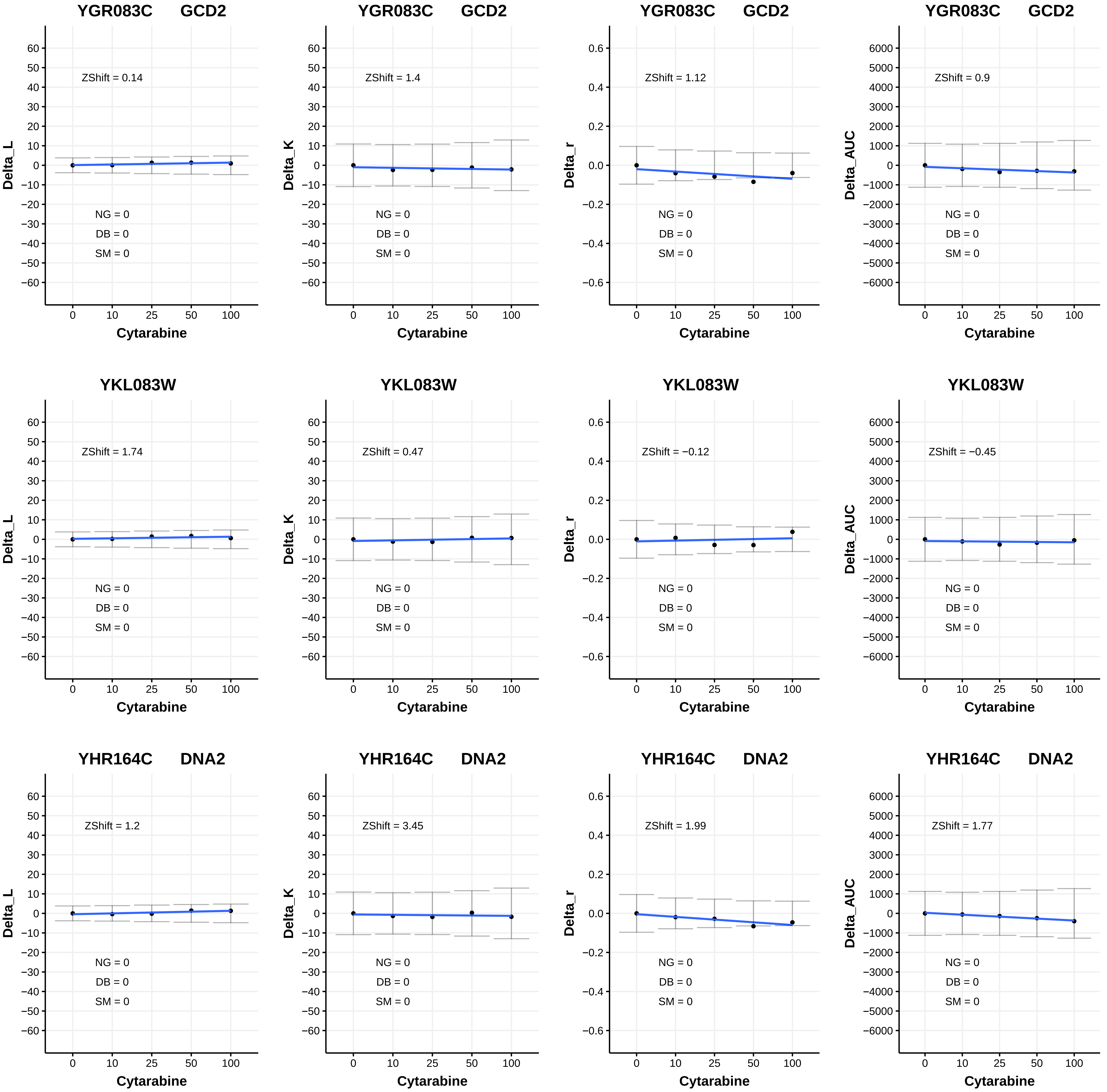

YGR083C GCD2
ZShift = 0.14
ZShift = 1.4
ZShift = 1.12
ZShift = 0.9
YKL083W
ZShift = 1.74
ZShift = 0.47
ZShift = −0.12
ZShift = −0.45
YHR164C DNA2
ZShift = 1.2
ZShift = 3.45
ZShift = 1.99
ZShift = 1.77
NG = 0
DB = 0
SM = 0
Delta_L
Delta_K
Delta_r
Delta_AUC
Cytarabine
0
10
25
50
100

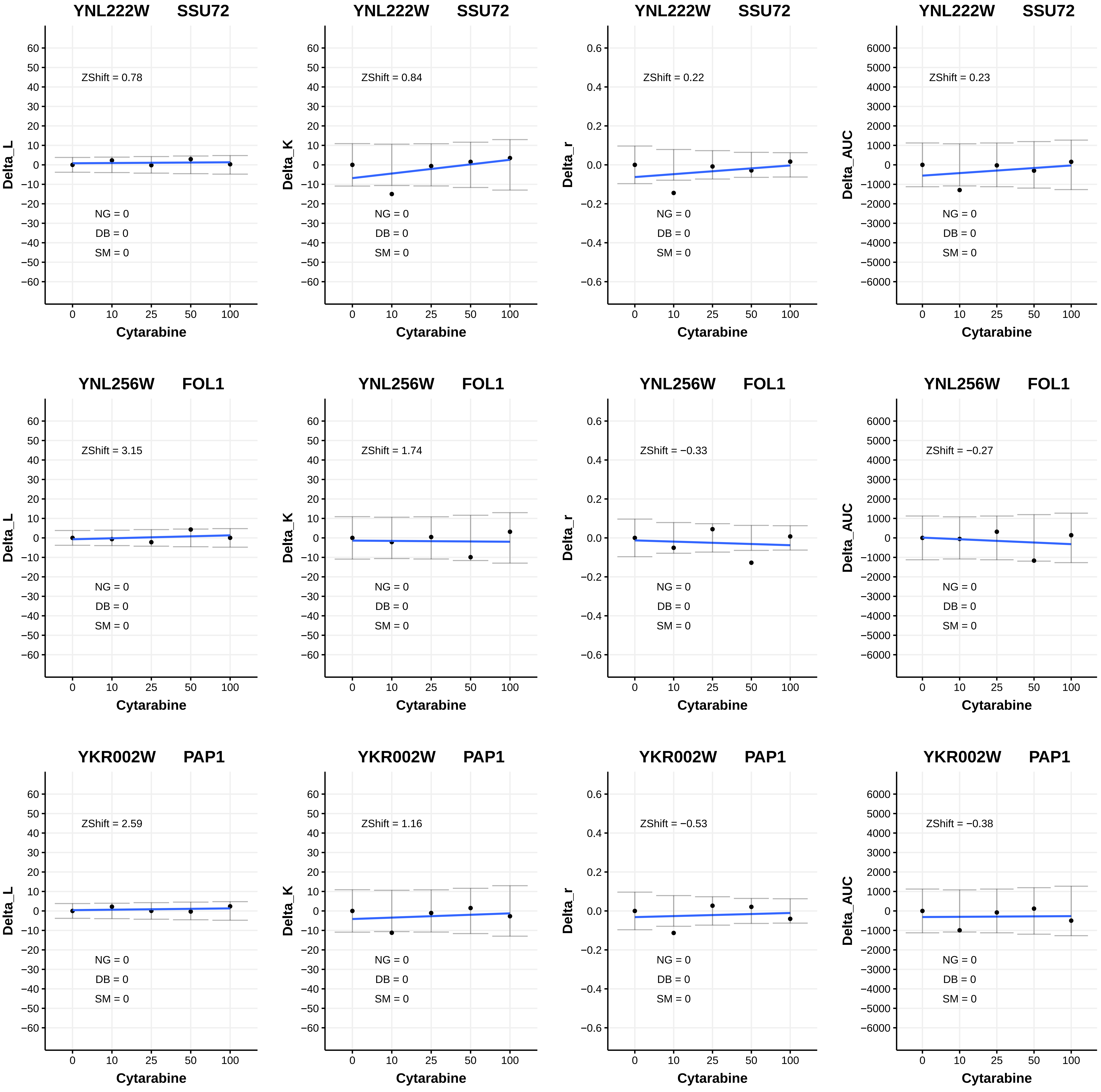
YNL222W SSU72
ZShift = 0.78
NG = 0
DB = 0
SM = 0
Delta_L
Cytarabine
YNL222W SSU72
ZShift = 0.84
NG = 0
DB = 0
SM = 0
Delta_K
Cytarabine
YNL222W SSU72
ZShift = 0.22
NG = 0
DB = 0
SM = 0
Delta_r
Cytarabine
YNL222W SSU72
ZShift = 0.23
NG = 0
DB = 0
SM = 0
Delta_AUC
Cytarabine
YNL256W FOL1
ZShift = 3.15
NG = 0
DB = 0
SM = 0
Delta_L
Cytarabine
YNL256W FOL1
ZShift = 1.74
NG = 0
DB = 0
SM = 0
Delta_K
Cytarabine
YNL256W FOL1
ZShift = −0.33
NG = 0
DB = 0
SM = 0
Delta_r
Cytarabine
YNL256W FOL1
ZShift = −0.27
NG = 0
DB = 0
SM = 0
Delta_AUC
Cytarabine
YKR002W PAP1
ZShift = 2.59
NG = 0
DB = 0
SM = 0
Delta_L
Cytarabine
YKR002W PAP1
ZShift = 1.16
NG = 0
DB = 0
SM = 0
Delta_K
Cytarabine
YKR002W PAP1
ZShift = −0.53
NG = 0
DB = 0
SM = 0
Delta_r
Cytarabine
YKR002W PAP1
ZShift = −0.38
NG = 0
DB = 0
SM = 0
Delta_AUC
Cytarabine
0 10 25 50 100

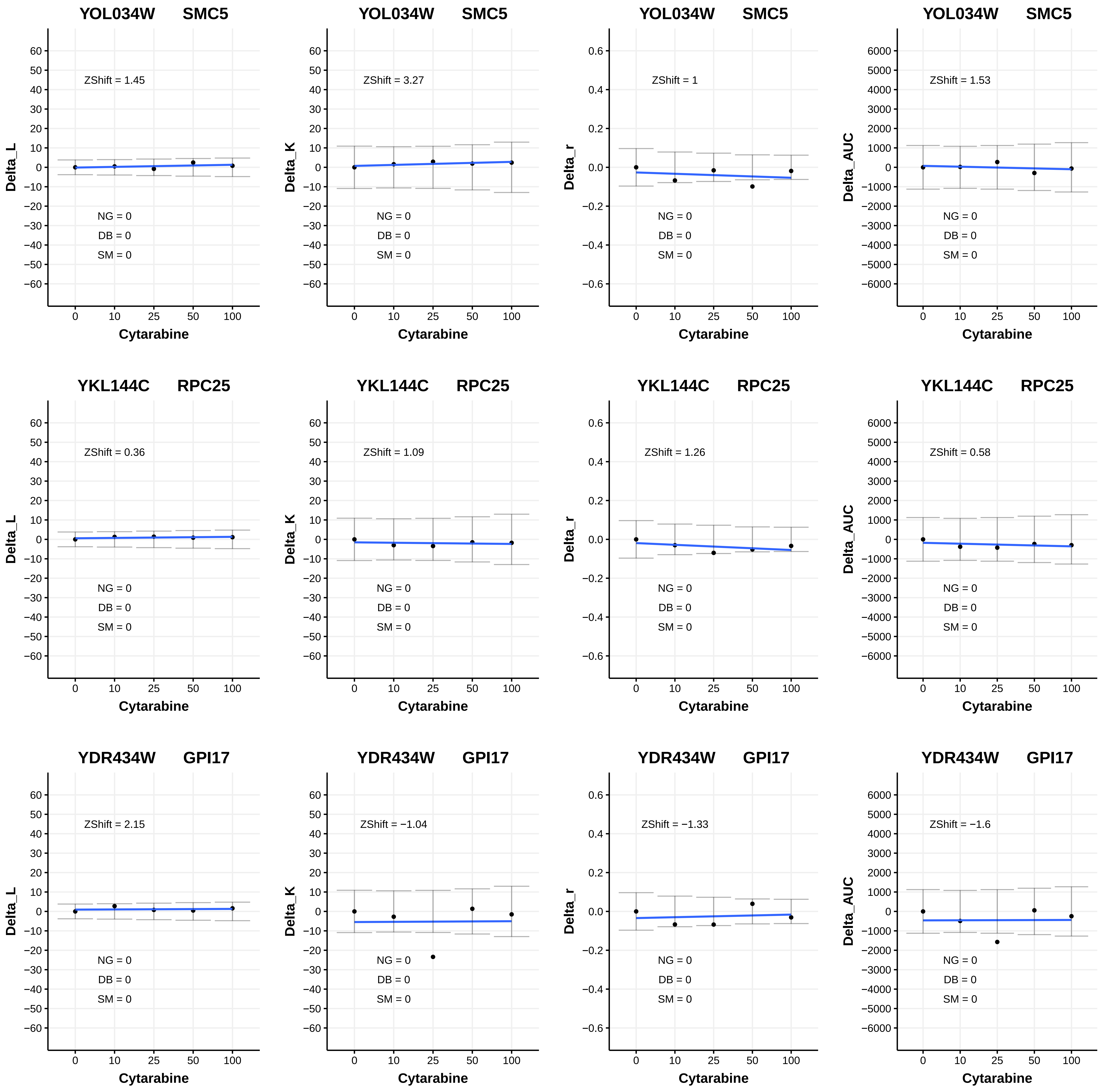
YOL034W SMC5
ZShift = 1.45
NG = 0
DB = 0
SM = 0
Delta_L
Cytarabine
YOL034W SMC5
ZShift = 3.27
NG = 0
DB = 0
SM = 0
Delta_K
Cytarabine
YOL034W SMC5
ZShift = 1
NG = 0
DB = 0
SM = 0
Delta_r
Cytarabine
YOL034W SMC5
ZShift = 1.53
NG = 0
DB = 0
SM = 0
Delta_AUC
Cytarabine
YKL144C RPC25
ZShift = 0.36
NG = 0
DB = 0
SM = 0
Delta_L
Cytarabine
YKL144C RPC25
ZShift = 1.09
NG = 0
DB = 0
SM = 0
Delta_K
Cytarabine
YKL144C RPC25
ZShift = 1.26
NG = 0
DB = 0
SM = 0
Delta_r
Cytarabine
YKL144C RPC25
ZShift = 0.58
NG = 0
DB = 0
SM = 0
Delta_AUC
Cytarabine
YDR434W GPI17
ZShift = 2.15
NG = 0
DB = 0
SM = 0
Delta_L
Cytarabine
YDR434W GPI17
ZShift = −1.04
NG = 0
DB = 0
SM = 0
Delta_K
Cytarabine
YDR434W GPI17
ZShift = −1.33
NG = 0
DB = 0
SM = 0
Delta_r
Cytarabine
YDR434W GPI17
ZShift = −1.6
NG = 0
DB = 0
SM = 0
Delta_AUC
Cytarabine

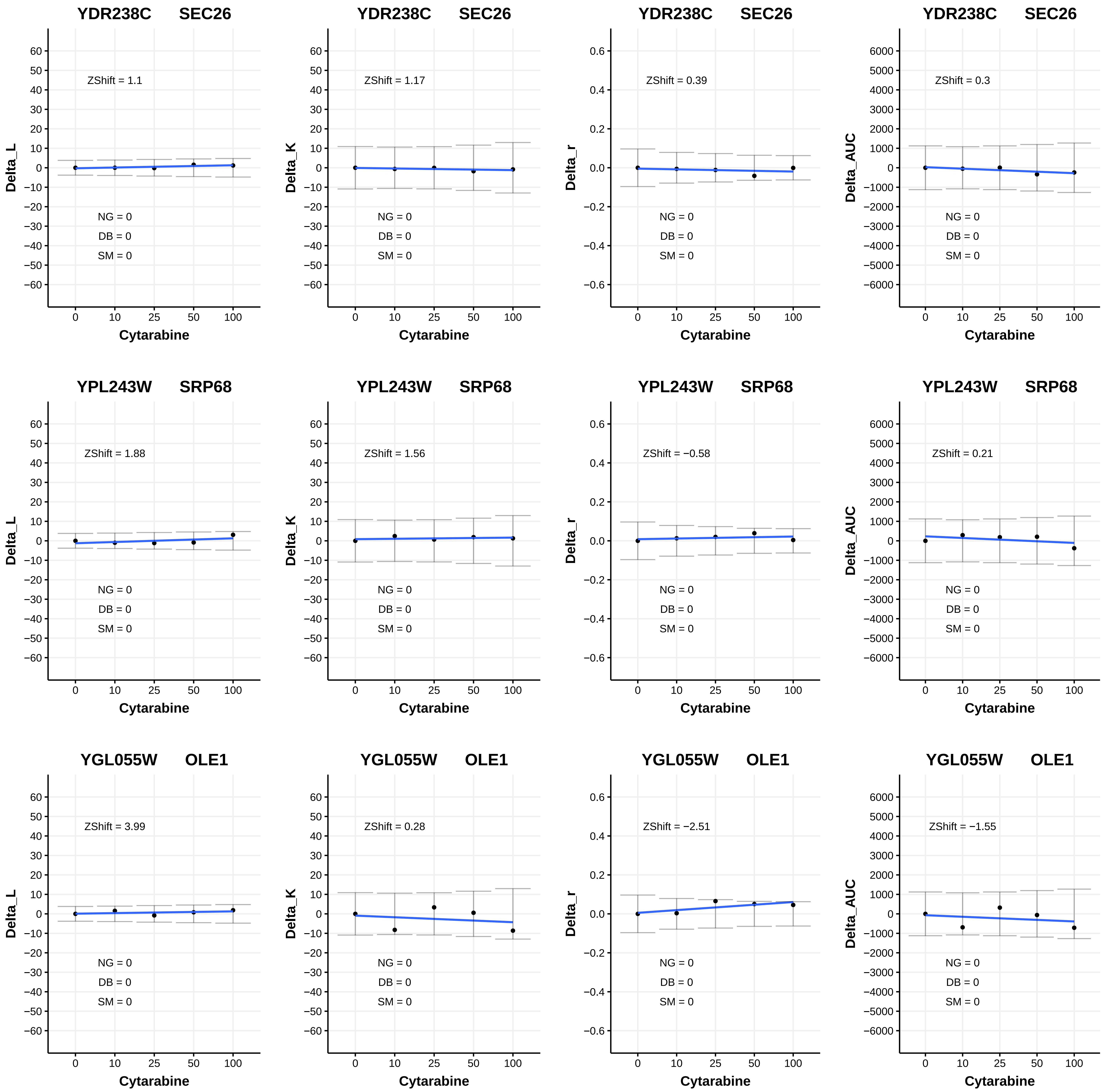
YDR238C SEC26
ZShift = 1.1
NG = 0
DB = 0
SM = 0
Delta_L
Cytarabine
YDR238C SEC26
ZShift = 1.17
NG = 0
DB = 0
SM = 0
Delta_K
Cytarabine
YDR238C SEC26
ZShift = 0.39
NG = 0
DB = 0
SM = 0
Delta_r
Cytarabine
YDR238C SEC26
ZShift = 0.3
NG = 0
DB = 0
SM = 0
Delta_AUC
Cytarabine
YPL243W SRP68
ZShift = 1.88
NG = 0
DB = 0
SM = 0
Delta_L
Cytarabine
YPL243W SRP68
ZShift = 1.56
NG = 0
DB = 0
SM = 0
Delta_K
Cytarabine
YPL243W SRP68
ZShift = −0.58
NG = 0
DB = 0
SM = 0
Delta_r
Cytarabine
YPL243W SRP68
ZShift = 0.21
NG = 0
DB = 0
SM = 0
Delta_AUC
Cytarabine
YGL055W OLE1
ZShift = 3.99
NG = 0
DB = 0
SM = 0
Delta_L
Cytarabine
YGL055W OLE1
ZShift = 0.28
NG = 0
DB = 0
SM = 0
Delta_K
Cytarabine
YGL055W OLE1
ZShift = −2.51
NG = 0
DB = 0
SM = 0
Delta_r
Cytarabine
YGL055W OLE1
ZShift = −1.55
NG = 0
DB = 0
SM = 0
Delta_AUC
Cytarabine
0 10 25 50 100

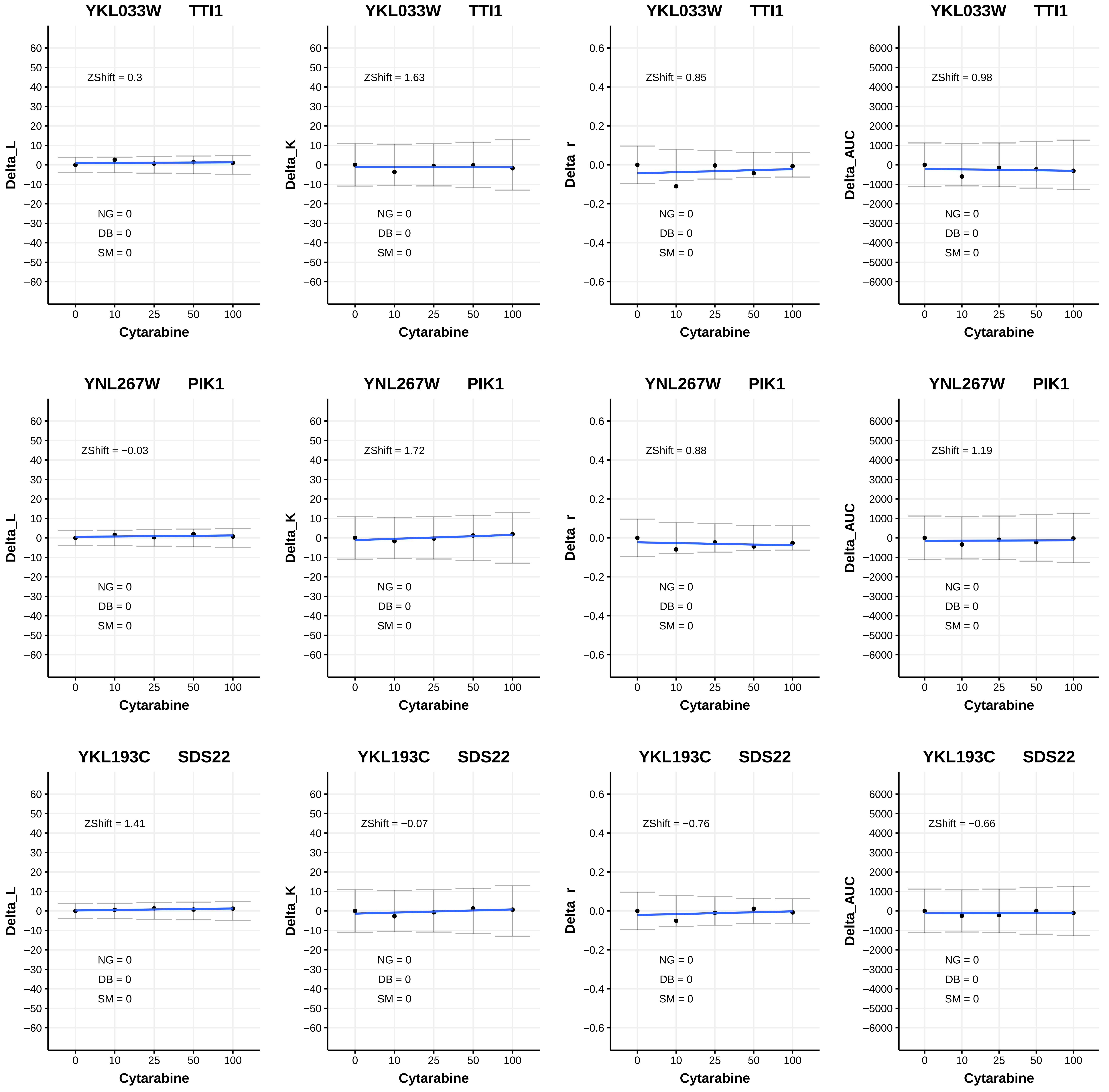
YKL033W TTI1
ZShift = 0.3
NG = 0
DB = 0
SM = 0
Delta_L
Cytarabine
YKL033W TTI1
ZShift = 1.63
NG = 0
DB = 0
SM = 0
Delta_K
Cytarabine
YKL033W TTI1
ZShift = 0.85
NG = 0
DB = 0
SM = 0
Delta_r
Cytarabine
YKL033W TTI1
ZShift = 0.98
NG = 0
DB = 0
SM = 0
Delta_AUC
Cytarabine
YNL267W PIK1
ZShift = −0.03
NG = 0
DB = 0
SM = 0
Delta_L
Cytarabine
YNL267W PIK1
ZShift = 1.72
NG = 0
DB = 0
SM = 0
Delta_K
Cytarabine
YNL267W PIK1
ZShift = 0.88
NG = 0
DB = 0
SM = 0
Delta_r
Cytarabine
YNL267W PIK1
ZShift = 1.19
NG = 0
DB = 0
SM = 0
Delta_AUC
Cytarabine
YKL193C SDS22
ZShift = 1.41
NG = 0
DB = 0
SM = 0
Delta_L
Cytarabine
YKL193C SDS22
ZShift = −0.07
NG = 0
DB = 0
SM = 0
Delta_K
Cytarabine
YKL193C SDS22
ZShift = −0.76
NG = 0
DB = 0
SM = 0
Delta_r
Cytarabine
YKL193C SDS22
ZShift = −0.66
NG = 0
DB = 0
SM = 0
Delta_AUC
Cytarabine

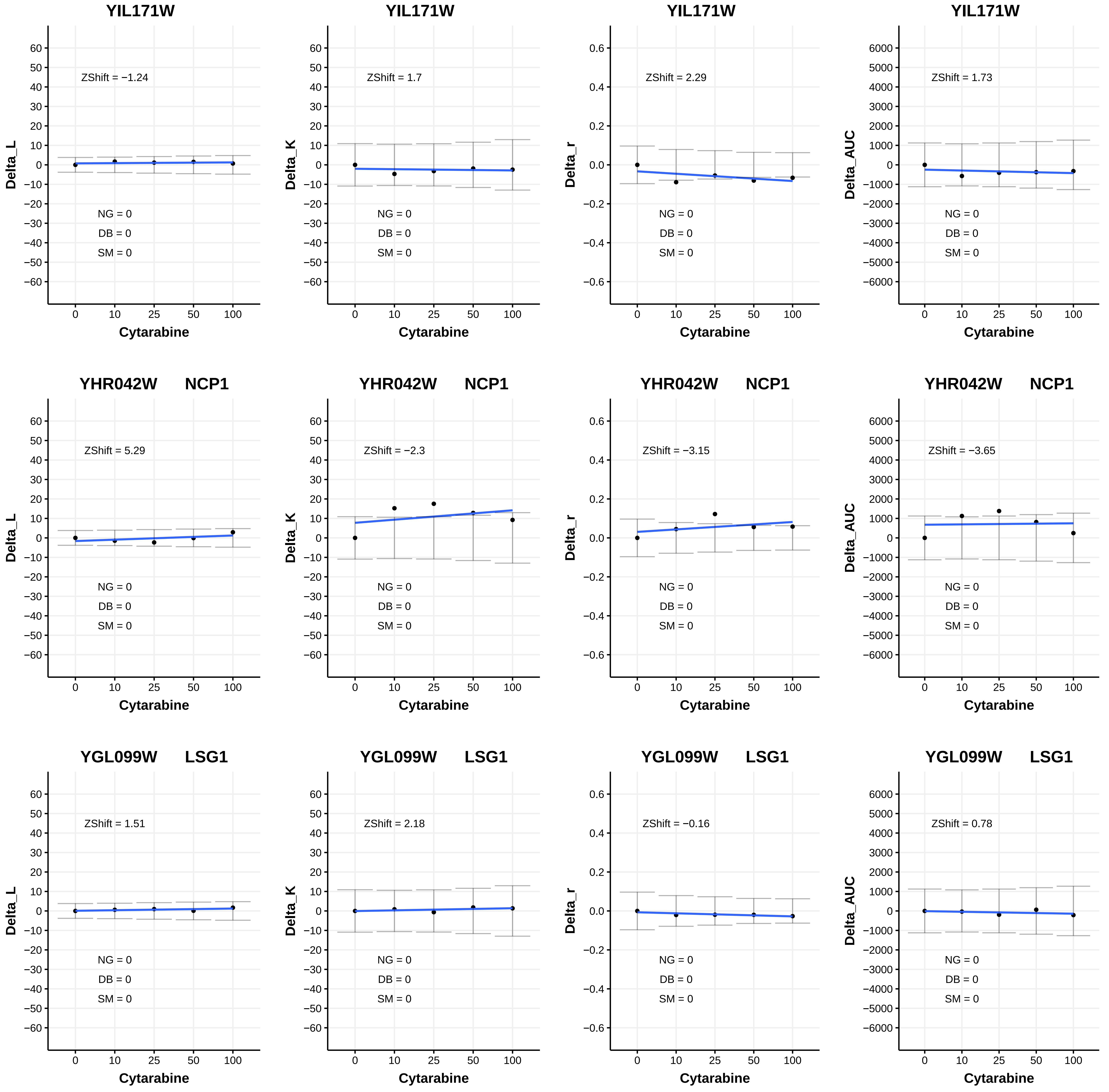

YIL171W
ZShift = −1.24
NG = 0
DB = 0
SM = 0
Delta_L
Cytarabine
YIL171W
ZShift = 1.7
NG = 0
DB = 0
SM = 0
Delta_K
Cytarabine
YIL171W
ZShift = 2.29
NG = 0
DB = 0
SM = 0
Delta_r
Cytarabine
YIL171W
ZShift = 1.73
NG = 0
DB = 0
SM = 0
Delta_AUC
Cytarabine
YHR042W NCP1
ZShift = 5.29
NG = 0
DB = 0
SM = 0
Delta_L
Cytarabine
YHR042W NCP1
ZShift = −2.3
NG = 0
DB = 0
SM = 0
Delta_K
Cytarabine
YHR042W NCP1
ZShift = −3.15
NG = 0
DB = 0
SM = 0
Delta_r
Cytarabine
YHR042W NCP1
ZShift = −3.65
NG = 0
DB = 0
SM = 0
Delta_AUC
Cytarabine
YGL099W LSG1
ZShift = 1.51
NG = 0
DB = 0
SM = 0
Delta_L
Cytarabine
YGL099W LSG1
ZShift = 2.18
NG = 0
DB = 0
SM = 0
Delta_K
Cytarabine
YGL099W LSG1
ZShift = −0.16
NG = 0
DB = 0
SM = 0
Delta_r
Cytarabine
YGL099W LSG1
ZShift = 0.78
NG = 0
DB = 0
SM = 0
Delta_AUC
Cytarabine

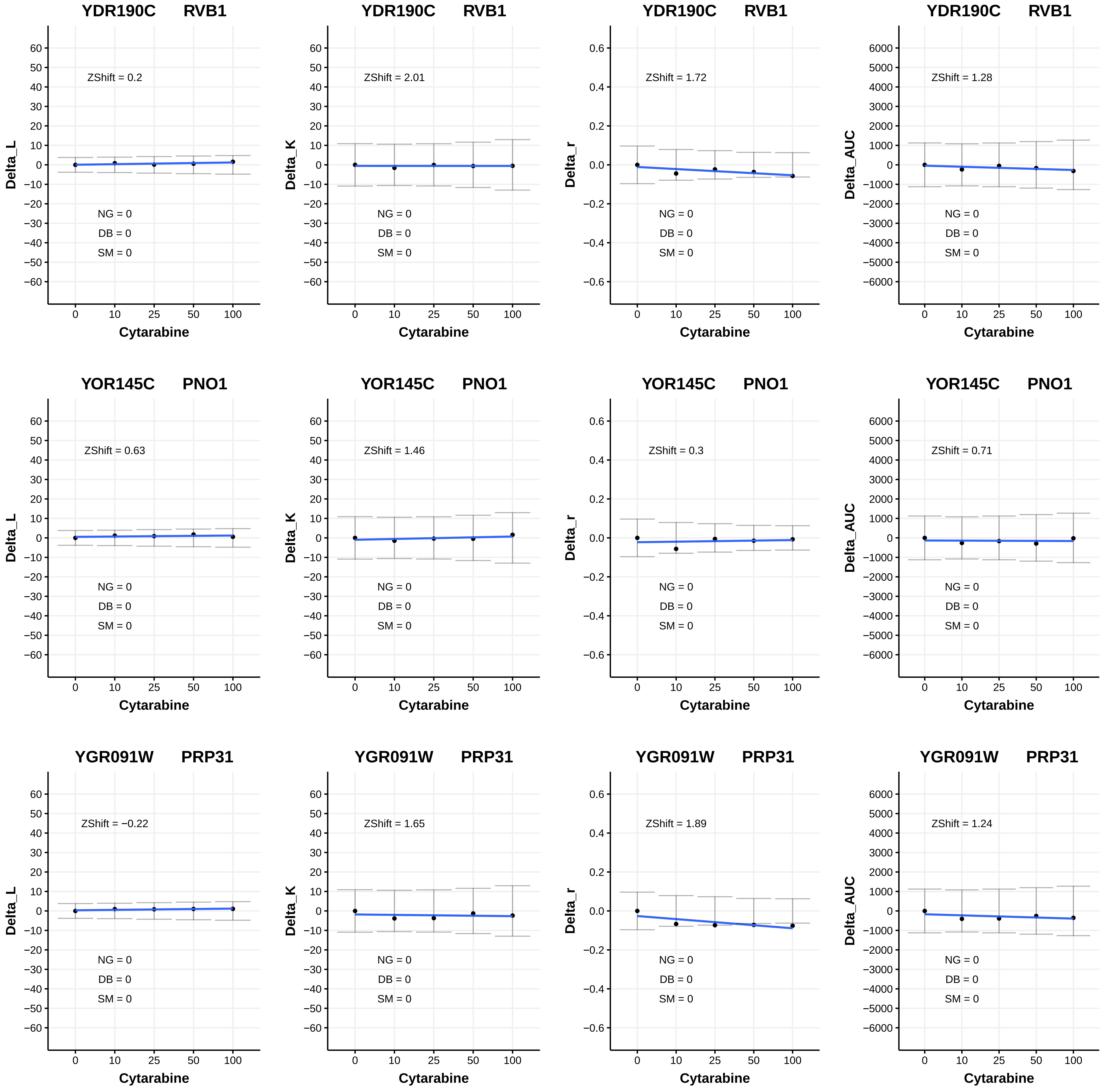
YDR190C RVB1
ZShift = 0.2
NG = 0
DB = 0
SM = 0
Delta_L
Cytarabine
YDR190C RVB1
ZShift = 2.01
NG = 0
DB = 0
SM = 0
Delta_K
Cytarabine
YDR190C RVB1
ZShift = 1.72
NG = 0
DB = 0
SM = 0
Delta_r
Cytarabine
YDR190C RVB1
ZShift = 1.28
NG = 0
DB = 0
SM = 0
Delta_AUC
Cytarabine
YOR145C PNO1
ZShift = 0.63
NG = 0
DB = 0
SM = 0
Delta_L
Cytarabine
YOR145C PNO1
ZShift = 1.46
NG = 0
DB = 0
SM = 0
Delta_K
Cytarabine
YOR145C PNO1
ZShift = 0.3
NG = 0
DB = 0
SM = 0
Delta_r
Cytarabine
YOR145C PNO1
ZShift = 0.71
NG = 0
DB = 0
SM = 0
Delta_AUC
Cytarabine
YGR091W PRP31
ZShift = −0.22
NG = 0
DB = 0
SM = 0
Delta_L
Cytarabine
YGR091W PRP31
ZShift = 1.65
NG = 0
DB = 0
SM = 0
Delta_K
Cytarabine
YGR091W PRP31
ZShift = 1.89
NG = 0
DB = 0
SM = 0
Delta_r
Cytarabine
YGR091W PRP31
ZShift = 1.24
NG = 0
DB = 0
SM = 0
Delta_AUC
Cytarabine

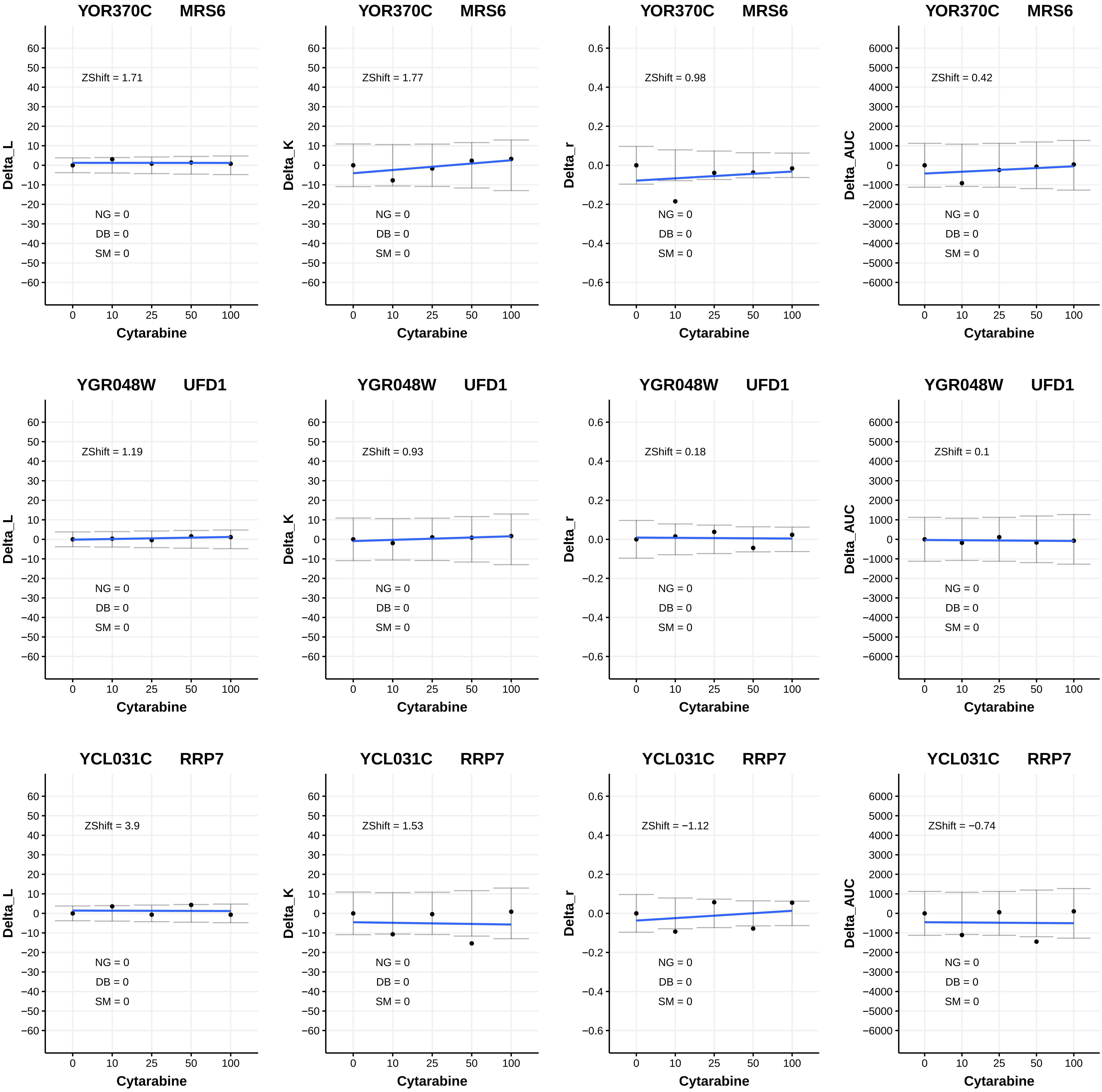
YOR370C MRS6
ZShift = 1.71
ZShift = 1.77
ZShift = 0.98
ZShift = 0.42
YGR048W UFD1
ZShift = 1.19
ZShift = 0.93
ZShift = 0.18
ZShift = 0.1
YCL031C RRP7
ZShift = 3.9
ZShift = 1.53
ZShift = −1.12
ZShift = −0.74
NG = 0
DB = 0
SM = 0
Delta_L
Delta_K
Delta_r
Delta_AUC
Cytarabine
0
10
25
50
100

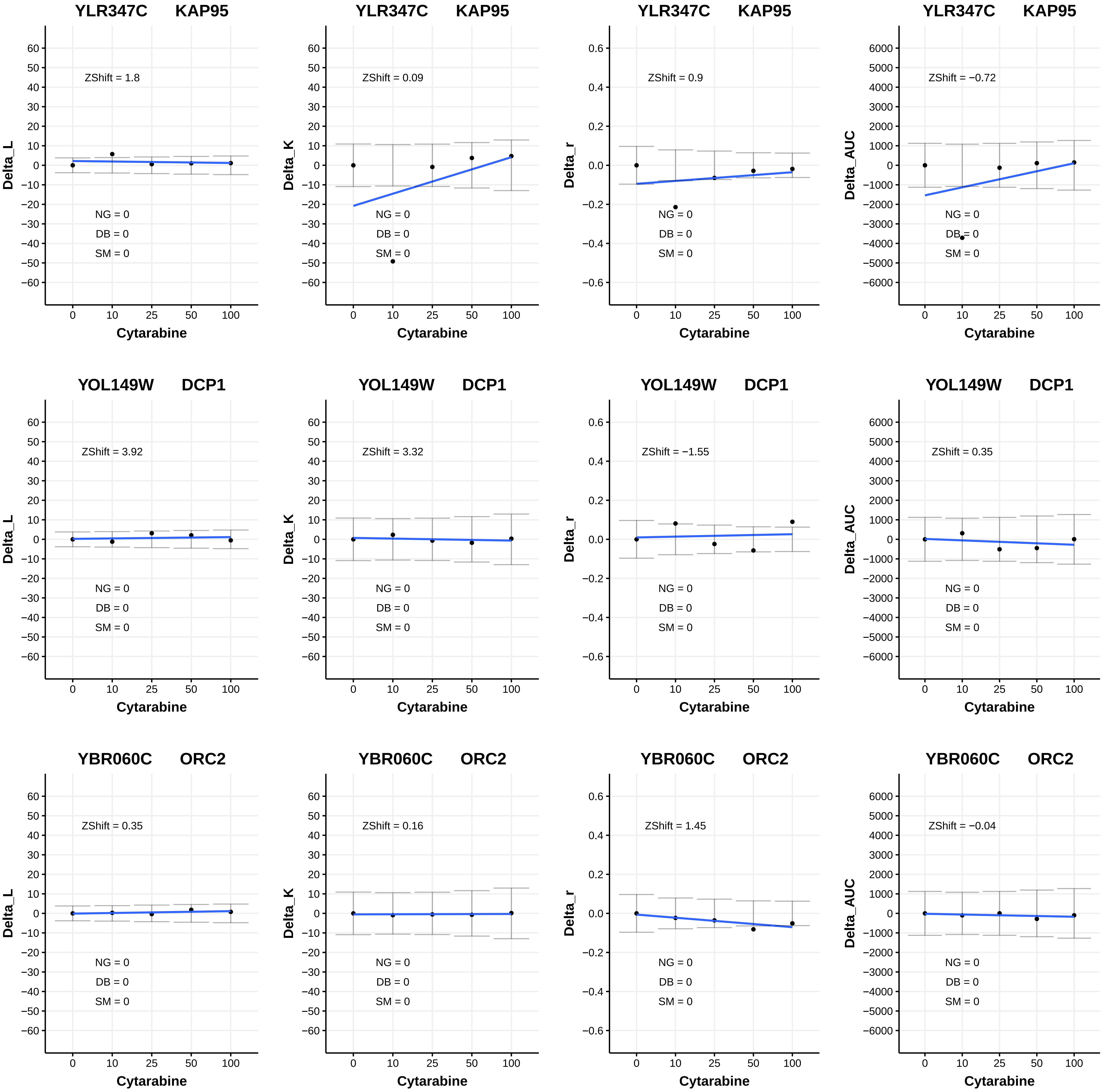

YLR347C KAP95
ZShift = 1.8
ZShift = 0.09
ZShift = 0.9
ZShift = −0.72
YOL149W DCP1
ZShift = 3.92
ZShift = 3.32
ZShift = −1.55
ZShift = 0.35
YBR060C ORC2
ZShift = 0.35
ZShift = 0.16
ZShift = 1.45
ZShift = −0.04
NG = 0
DB = 0
SM = 0
Delta_L
Delta_K
Delta_r
Delta_AUC
Cytarabine
0
10
25
50
100

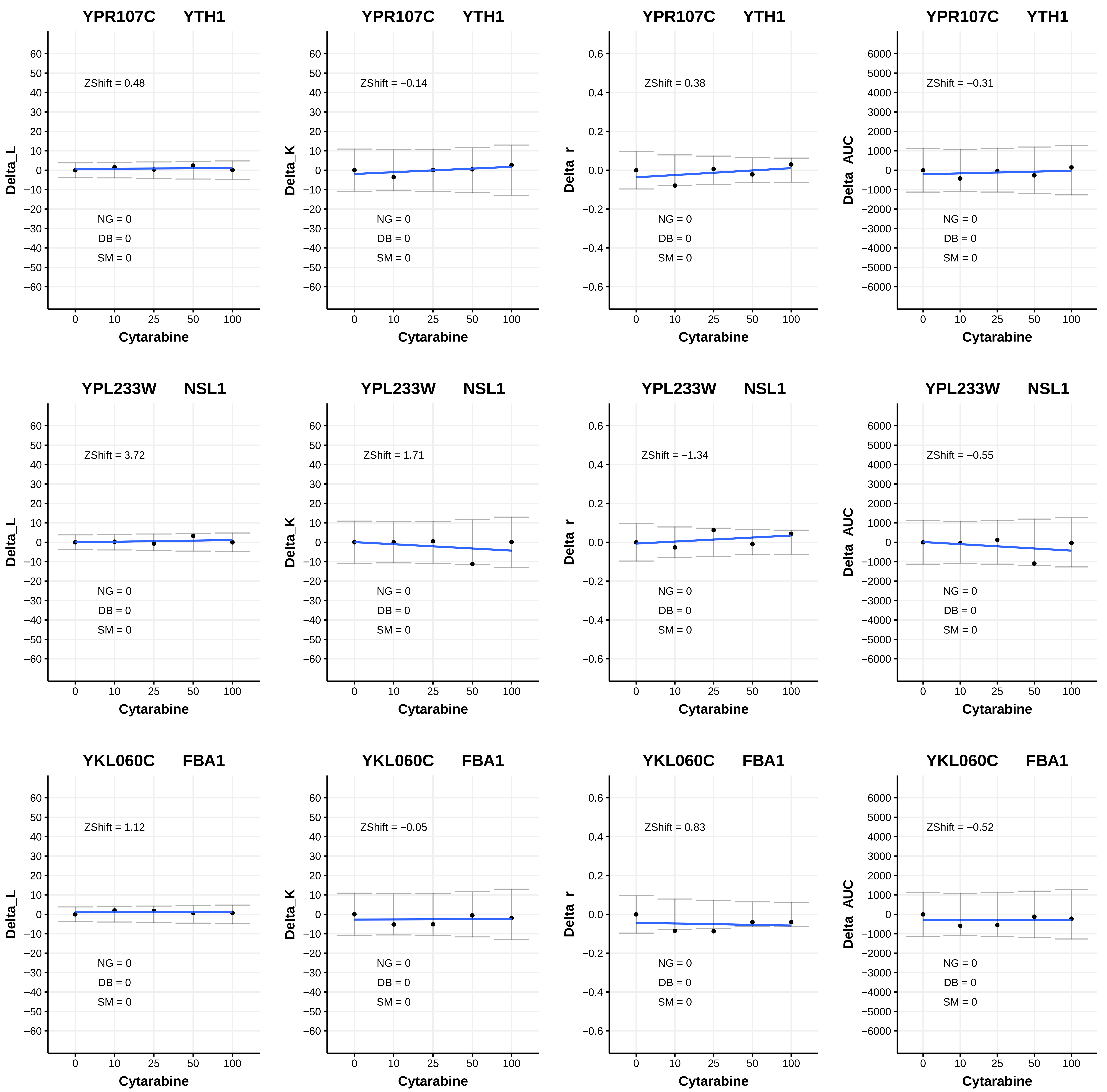
YPR107C YTH1
ZShift = 0.48
NG = 0
DB = 0
SM = 0
Delta_L
Cytarabine
YPR107C YTH1
ZShift = −0.14
NG = 0
DB = 0
SM = 0
Delta_K
Cytarabine
YPR107C YTH1
ZShift = 0.38
NG = 0
DB = 0
SM = 0
Delta_r
Cytarabine
YPR107C YTH1
ZShift = −0.31
NG = 0
DB = 0
SM = 0
Delta_AUC
Cytarabine
YPL233W NSL1
ZShift = 3.72
NG = 0
DB = 0
SM = 0
Delta_L
Cytarabine
YPL233W NSL1
ZShift = 1.71
NG = 0
DB = 0
SM = 0
Delta_K
Cytarabine
YPL233W NSL1
ZShift = −1.34
NG = 0
DB = 0
SM = 0
Delta_r
Cytarabine
YPL233W NSL1
ZShift = −0.55
NG = 0
DB = 0
SM = 0
Delta_AUC
Cytarabine
YKL060C FBA1
ZShift = 1.12
NG = 0
DB = 0
SM = 0
Delta_L
Cytarabine
YKL060C FBA1
ZShift = −0.05
NG = 0
DB = 0
SM = 0
Delta_K
Cytarabine
YKL060C FBA1
ZShift = 0.83
NG = 0
DB = 0
SM = 0
Delta_r
Cytarabine
YKL060C FBA1
ZShift = −0.52
NG = 0
DB = 0
SM = 0
Delta_AUC
Cytarabine

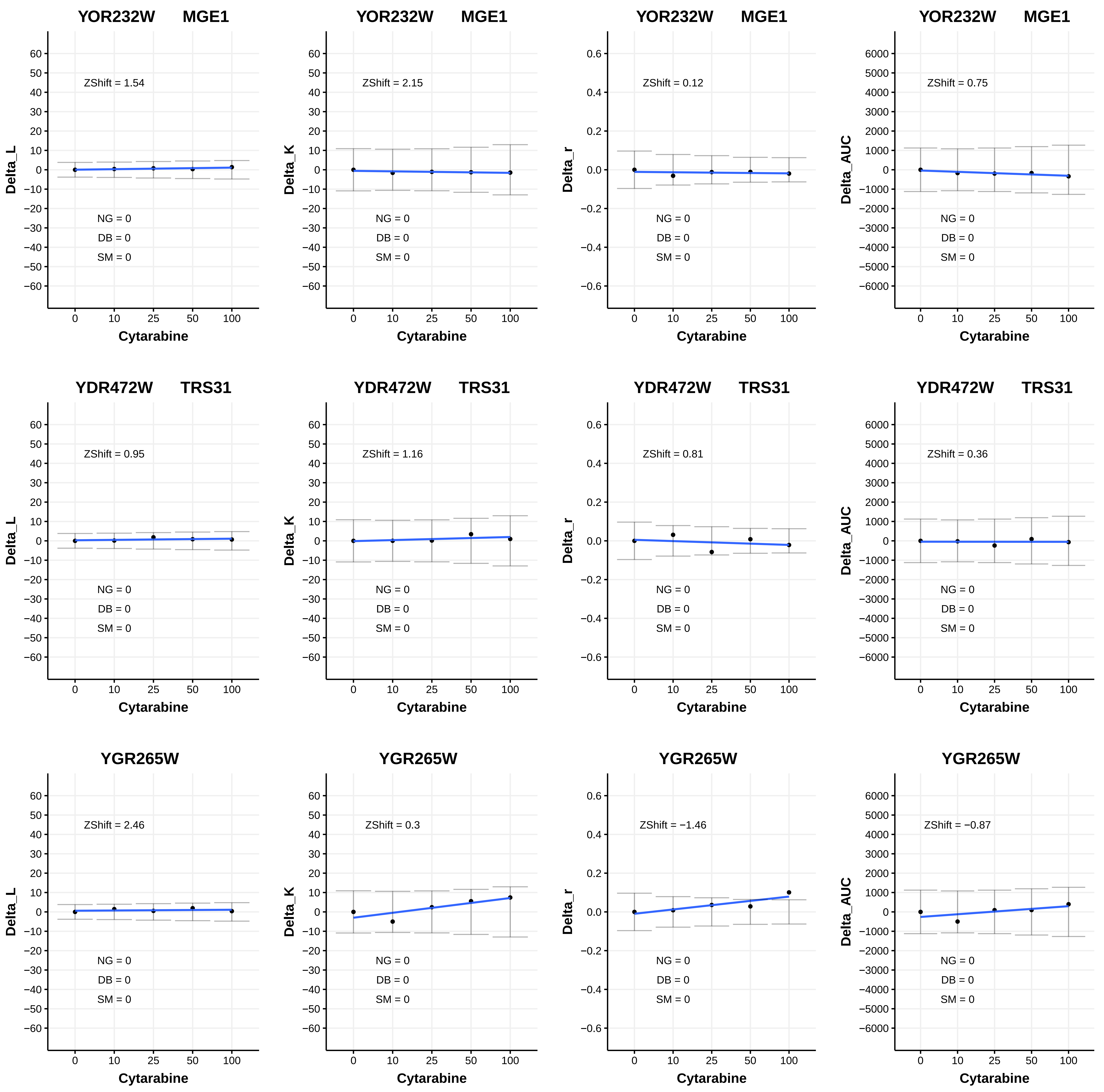
YOR232W MGE1
ZShift = 1.54
ZShift = 2.15
ZShift = 0.12
ZShift = 0.75
YDR472W TRS31
ZShift = 0.95
ZShift = 1.16
ZShift = 0.81
ZShift = 0.36
YGR265W
ZShift = 2.46
ZShift = 0.3
ZShift = −1.46
ZShift = −0.87
NG = 0
DB = 0
SM = 0
Delta_L
Delta_K
Delta_r
Delta_AUC
Cytarabine

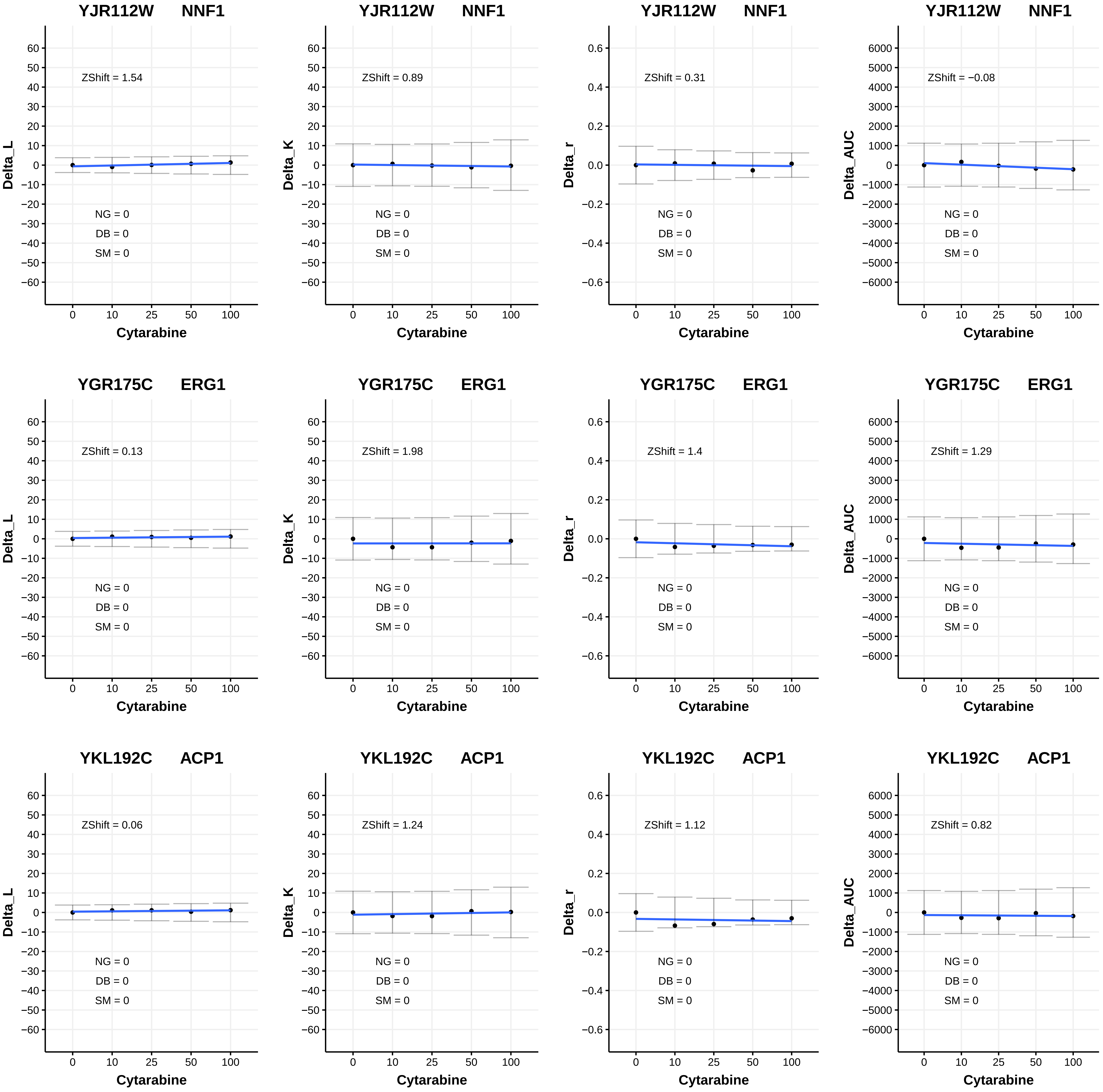

YJR112W NNF1
ZShift = 1.54
Delta_L
NG = 0
DB = 0
SM = 0
Cytarabine
YJR112W NNF1
ZShift = 0.89
Delta_K
NG = 0
DB = 0
SM = 0
Cytarabine
YJR112W NNF1
ZShift = 0.31
Delta_r
NG = 0
DB = 0
SM = 0
Cytarabine
YJR112W NNF1
ZShift = −0.08
Delta_AUC
NG = 0
DB = 0
SM = 0
Cytarabine
YGR175C ERG1
ZShift = 0.13
Delta_L
NG = 0
DB = 0
SM = 0
Cytarabine
YGR175C ERG1
ZShift = 1.98
Delta_K
NG = 0
DB = 0
SM = 0
Cytarabine
YGR175C ERG1
ZShift = 1.4
Delta_r
NG = 0
DB = 0
SM = 0
Cytarabine
YGR175C ERG1
ZShift = 1.29
Delta_AUC
NG = 0
DB = 0
SM = 0
Cytarabine
YKL192C ACP1
ZShift = 0.06
Delta_L
NG = 0
DB = 0
SM = 0
Cytarabine
YKL192C ACP1
ZShift = 1.24
Delta_K
NG = 0
DB = 0
SM = 0
Cytarabine
YKL192C ACP1
ZShift = 1.12
Delta_r
NG = 0
DB = 0
SM = 0
Cytarabine
YKL192C ACP1
ZShift = 0.82
Delta_AUC
NG = 0
DB = 0
SM = 0
Cytarabine

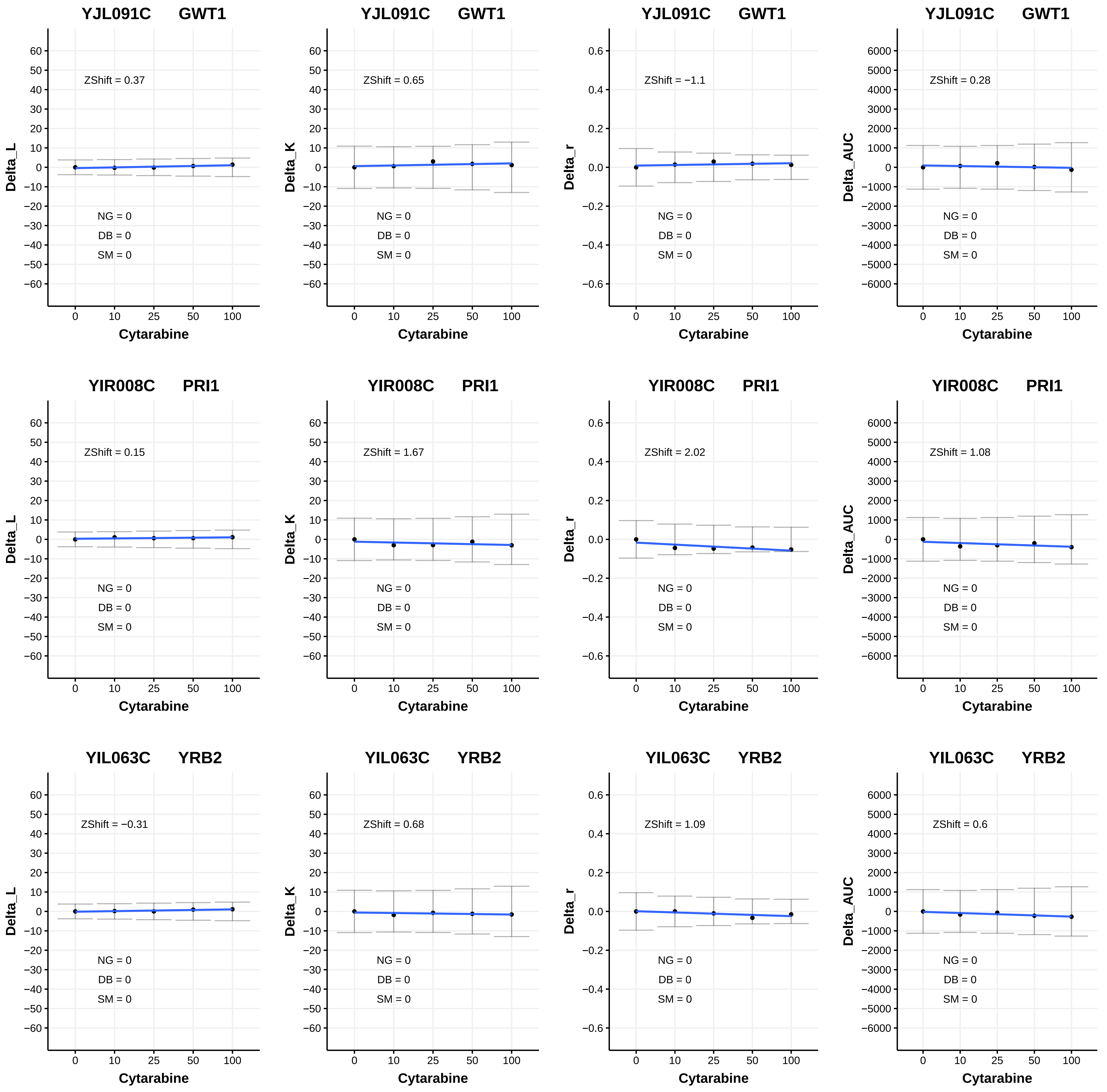
YJL091C GWT1
ZShift = 0.37
NG = 0
DB = 0
SM = 0
Delta_L
Cytarabine
YJL091C GWT1
ZShift = 0.65
NG = 0
DB = 0
SM = 0
Delta_K
Cytarabine
YJL091C GWT1
ZShift = −1.1
NG = 0
DB = 0
SM = 0
Delta_r
Cytarabine
YJL091C GWT1
ZShift = 0.28
NG = 0
DB = 0
SM = 0
Delta_AUC
Cytarabine
YIR008C PRI1
ZShift = 0.15
NG = 0
DB = 0
SM = 0
Delta_L
Cytarabine
YIR008C PRI1
ZShift = 1.67
NG = 0
DB = 0
SM = 0
Delta_K
Cytarabine
YIR008C PRI1
ZShift = 2.02
NG = 0
DB = 0
SM = 0
Delta_r
Cytarabine
YIR008C PRI1
ZShift = 1.08
NG = 0
DB = 0
SM = 0
Delta_AUC
Cytarabine
YIL063C YRB2
ZShift = −0.31
NG = 0
DB = 0
SM = 0
Delta_L
Cytarabine
YIL063C YRB2
ZShift = 0.68
NG = 0
DB = 0
SM = 0
Delta_K
Cytarabine
YIL063C YRB2
ZShift = 1.09
NG = 0
DB = 0
SM = 0
Delta_r
Cytarabine
YIL063C YRB2
ZShift = 0.6
NG = 0
DB = 0
SM = 0
Delta_AUC
Cytarabine

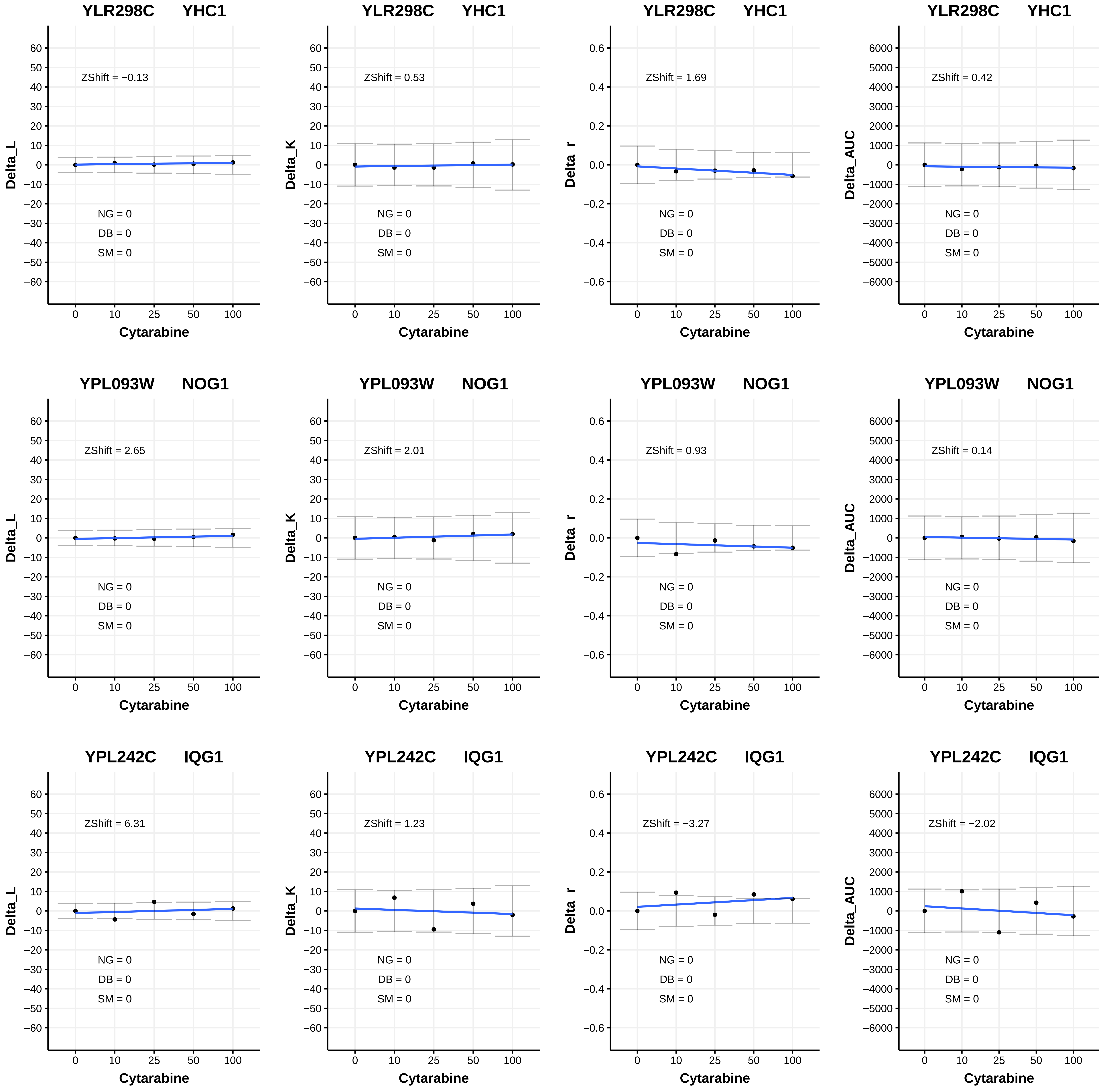

YLR298C YHC1
ZShift = −0.13
ZShift = 0.53
ZShift = 1.69
ZShift = 0.42
YPL093W NOG1
ZShift = 2.65
ZShift = 2.01
ZShift = 0.93
ZShift = 0.14
YPL242C IQG1
ZShift = 6.31
ZShift = 1.23
ZShift = −3.27
ZShift = −2.02
NG = 0
DB = 0
SM = 0
Delta_L
Delta_K
Delta_r
Delta_AUC
Cytarabine
0
10
25
50
100

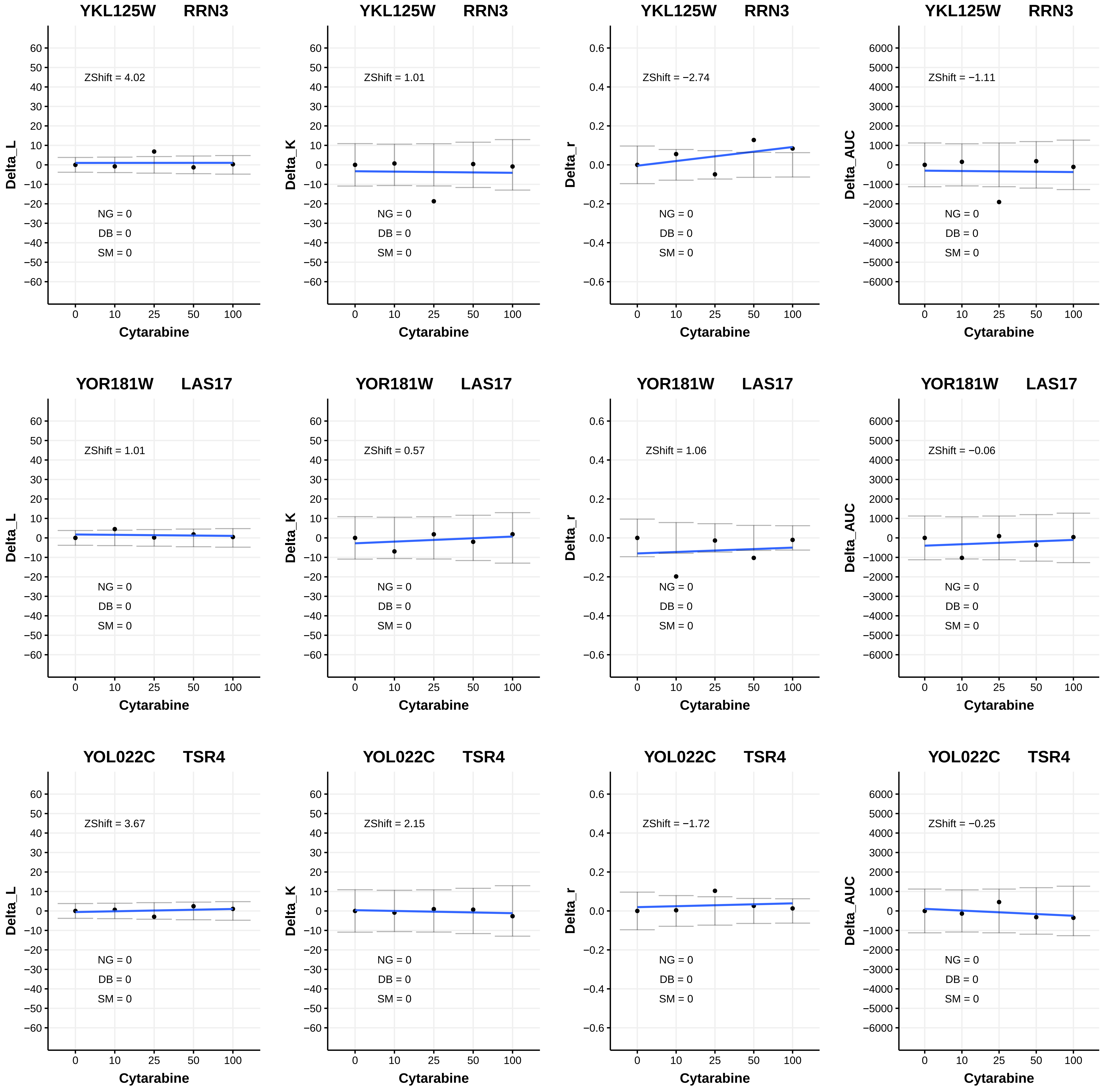
YKL125W RRN3
ZShift = 4.02
NG = 0
DB = 0
SM = 0
Delta_L
Cytarabine
YKL125W RRN3
ZShift = 1.01
NG = 0
DB = 0
SM = 0
Delta_K
Cytarabine
YKL125W RRN3
ZShift = −2.74
NG = 0
DB = 0
SM = 0
Delta_r
Cytarabine
YKL125W RRN3
ZShift = −1.11
NG = 0
DB = 0
SM = 0
Delta_AUC
Cytarabine
YOR181W LAS17
ZShift = 1.01
NG = 0
DB = 0
SM = 0
Delta_L
Cytarabine
YOR181W LAS17
ZShift = 0.57
NG = 0
DB = 0
SM = 0
Delta_K
Cytarabine
YOR181W LAS17
ZShift = 1.06
NG = 0
DB = 0
SM = 0
Delta_r
Cytarabine
YOR181W LAS17
ZShift = −0.06
NG = 0
DB = 0
SM = 0
Delta_AUC
Cytarabine
YOL022C TSR4
ZShift = 3.67
NG = 0
DB = 0
SM = 0
Delta_L
Cytarabine
YOL022C TSR4
ZShift = 2.15
NG = 0
DB = 0
SM = 0
Delta_K
Cytarabine
YOL022C TSR4
ZShift = −1.72
NG = 0
DB = 0
SM = 0
Delta_r
Cytarabine
YOL022C TSR4
ZShift = −0.25
NG = 0
DB = 0
SM = 0
Delta_AUC
Cytarabine

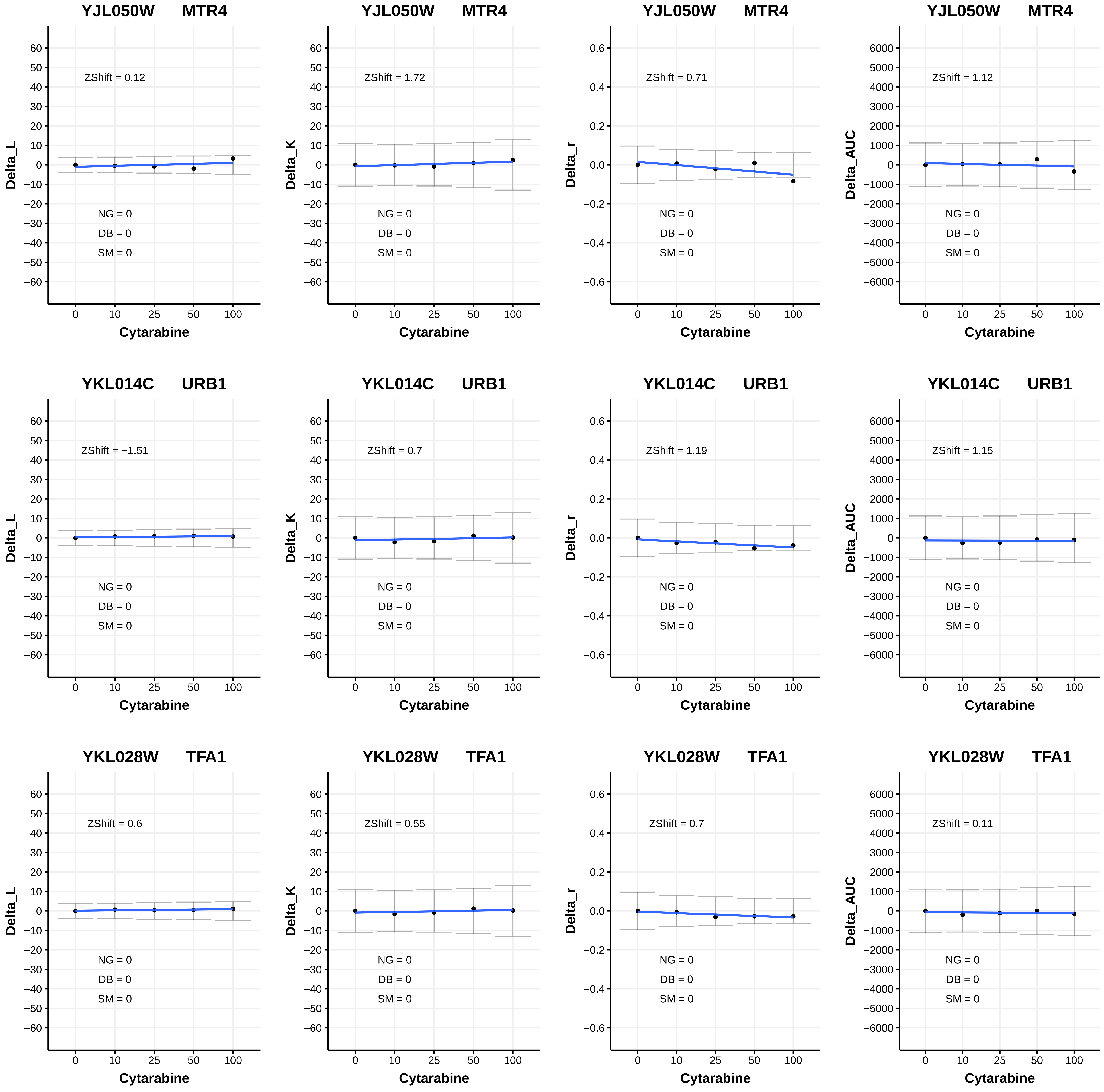

YJL050W MTR4
ZShift = 0.12
NG = 0
DB = 0
SM = 0
Delta_L
Cytarabine
YJL050W MTR4
ZShift = 1.72
NG = 0
DB = 0
SM = 0
Delta_K
Cytarabine
YJL050W MTR4
ZShift = 0.71
NG = 0
DB = 0
SM = 0
Delta_r
Cytarabine
YJL050W MTR4
ZShift = 1.12
NG = 0
DB = 0
SM = 0
Delta_AUC
Cytarabine
YKL014C URB1
ZShift = −1.51
NG = 0
DB = 0
SM = 0
Delta_L
Cytarabine
YKL014C URB1
ZShift = 0.7
NG = 0
DB = 0
SM = 0
Delta_K
Cytarabine
YKL014C URB1
ZShift = 1.19
NG = 0
DB = 0
SM = 0
Delta_r
Cytarabine
YKL014C URB1
ZShift = 1.15
NG = 0
DB = 0
SM = 0
Delta_AUC
Cytarabine
YKL028W TFA1
ZShift = 0.6
NG = 0
DB = 0
SM = 0
Delta_L
Cytarabine
YKL028W TFA1
ZShift = 0.55
NG = 0
DB = 0
SM = 0
Delta_K
Cytarabine
YKL028W TFA1
ZShift = 0.7
NG = 0
DB = 0
SM = 0
Delta_r
Cytarabine
YKL028W TFA1
ZShift = 0.11
NG = 0
DB = 0
SM = 0
Delta_AUC
Cytarabine

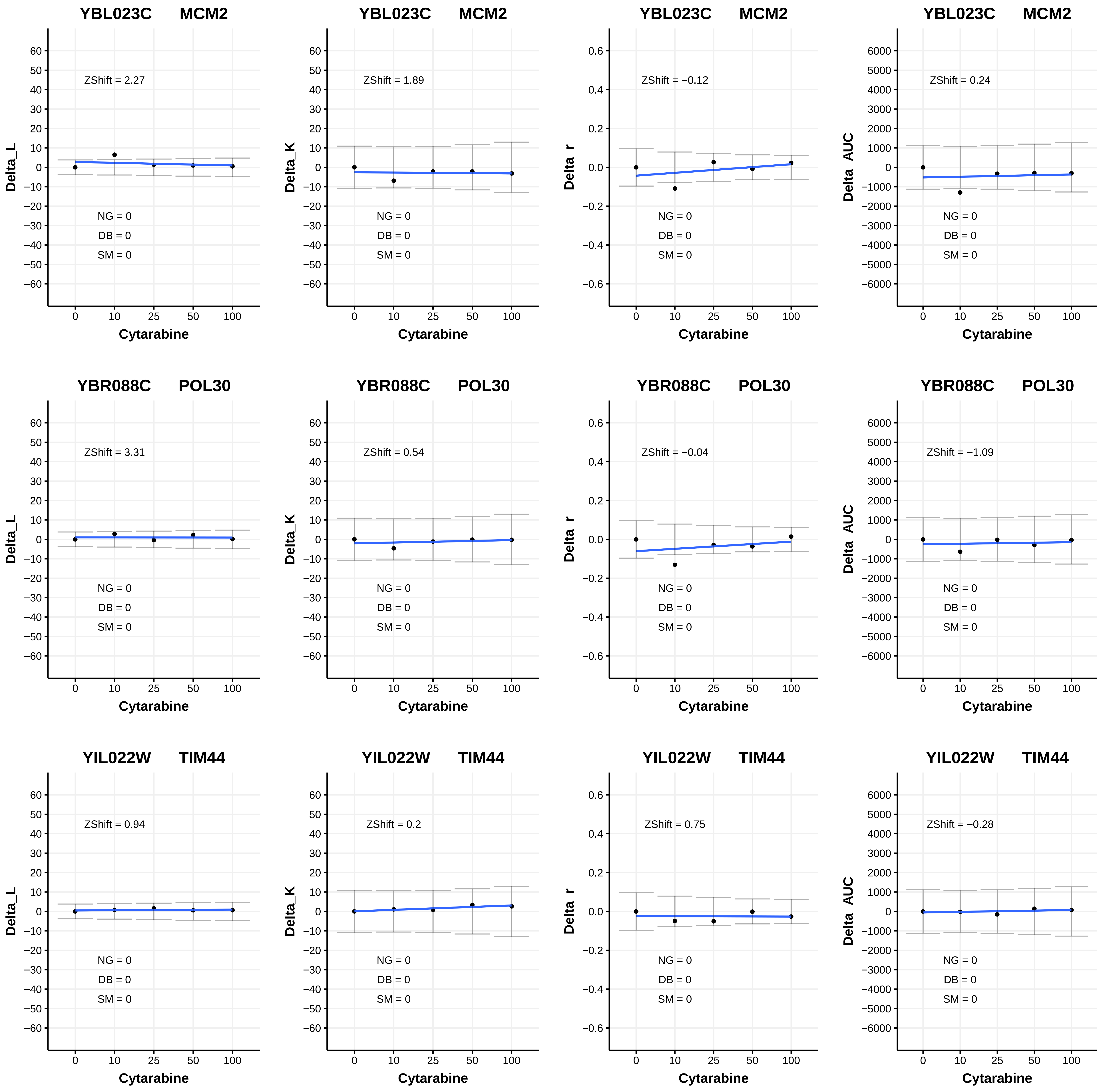
YBL023C MCM2
Delta_L
ZShift = 2.27
NG = 0
DB = 0
SM = 0
Cytarabine
YBL023C MCM2
Delta_K
ZShift = 1.89
NG = 0
DB = 0
SM = 0
Cytarabine
YBL023C MCM2
Delta_r
ZShift = −0.12
NG = 0
DB = 0
SM = 0
Cytarabine
YBL023C MCM2
Delta_AUC
ZShift = 0.24
NG = 0
DB = 0
SM = 0
Cytarabine
YBR088C POL30
Delta_L
ZShift = 3.31
NG = 0
DB = 0
SM = 0
Cytarabine
YBR088C POL30
Delta_K
ZShift = 0.54
NG = 0
DB = 0
SM = 0
Cytarabine
YBR088C POL30
Delta_r
ZShift = −0.04
NG = 0
DB = 0
SM = 0
Cytarabine
YBR088C POL30
Delta_AUC
ZShift = −1.09
NG = 0
DB = 0
SM = 0
Cytarabine
YIL022W TIM44
Delta_L
ZShift = 0.94
NG = 0
DB = 0
SM = 0
Cytarabine
YIL022W TIM44
Delta_K
ZShift = 0.2
NG = 0
DB = 0
SM = 0
Cytarabine
YIL022W TIM44
Delta_r
ZShift = 0.75
NG = 0
DB = 0
SM = 0
Cytarabine
YIL022W TIM44
Delta_AUC
ZShift = −0.28
NG = 0
DB = 0
SM = 0
Cytarabine

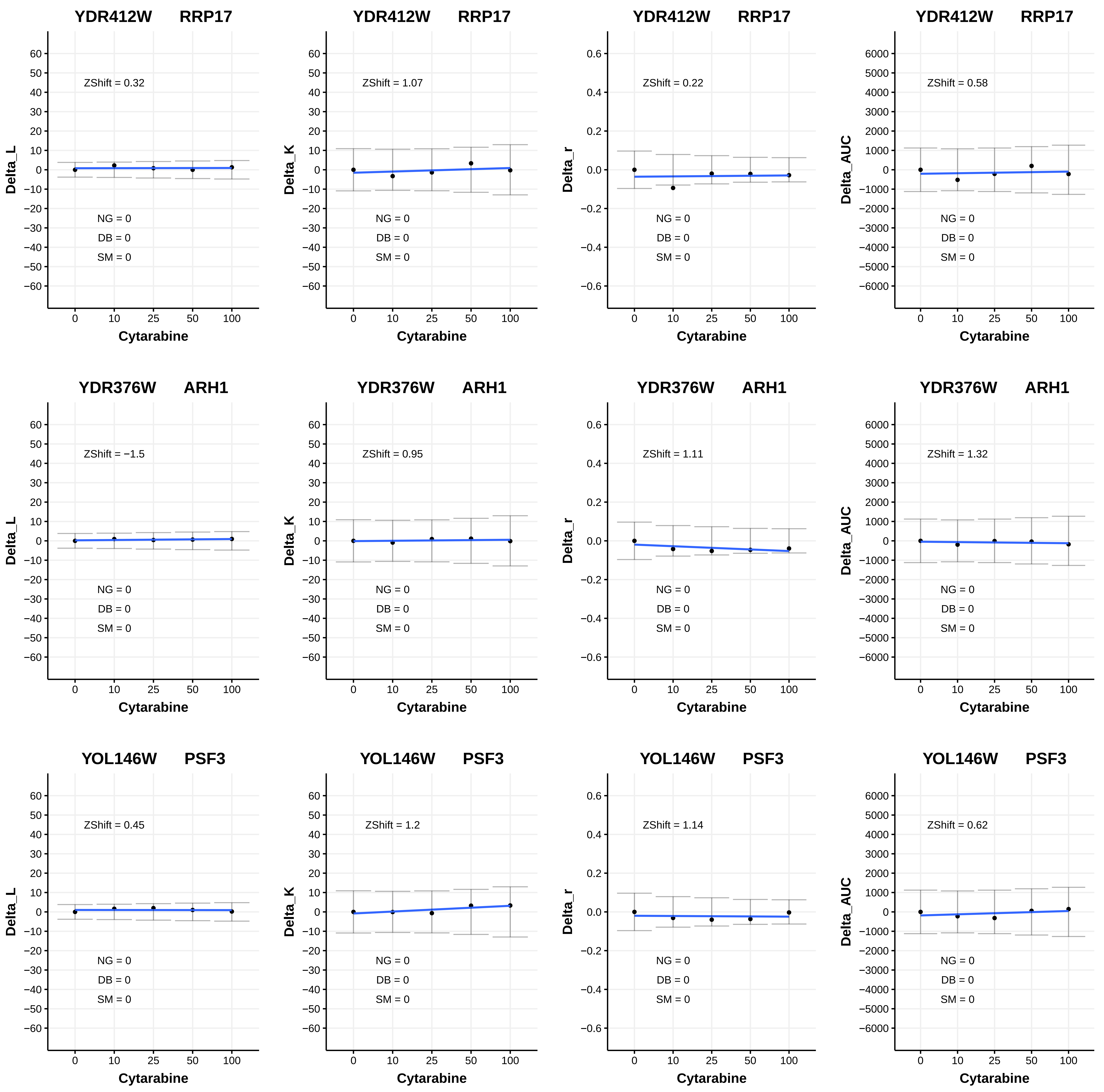

YDR412W RRP17
ZShift = 0.32
NG = 0
DB = 0
SM = 0
Delta_L
Cytarabine
YDR412W RRP17
ZShift = 1.07
NG = 0
DB = 0
SM = 0
Delta_K
Cytarabine
YDR412W RRP17
ZShift = 0.22
NG = 0
DB = 0
SM = 0
Delta_r
Cytarabine
YDR412W RRP17
ZShift = 0.58
NG = 0
DB = 0
SM = 0
Delta_AUC
Cytarabine
YDR376W ARH1
ZShift = −1.5
NG = 0
DB = 0
SM = 0
Delta_L
Cytarabine
YDR376W ARH1
ZShift = 0.95
NG = 0
DB = 0
SM = 0
Delta_K
Cytarabine
YDR376W ARH1
ZShift = 1.11
NG = 0
DB = 0
SM = 0
Delta_r
Cytarabine
YDR376W ARH1
ZShift = 1.32
NG = 0
DB = 0
SM = 0
Delta_AUC
Cytarabine
YOL146W PSF3
ZShift = 0.45
NG = 0
DB = 0
SM = 0
Delta_L
Cytarabine
YOL146W PSF3
ZShift = 1.2
NG = 0
DB = 0
SM = 0
Delta_K
Cytarabine
YOL146W PSF3
ZShift = 1.14
NG = 0
DB = 0
SM = 0
Delta_r
Cytarabine
YOL146W PSF3
ZShift = 0.62
NG = 0
DB = 0
SM = 0
Delta_AUC
Cytarabine

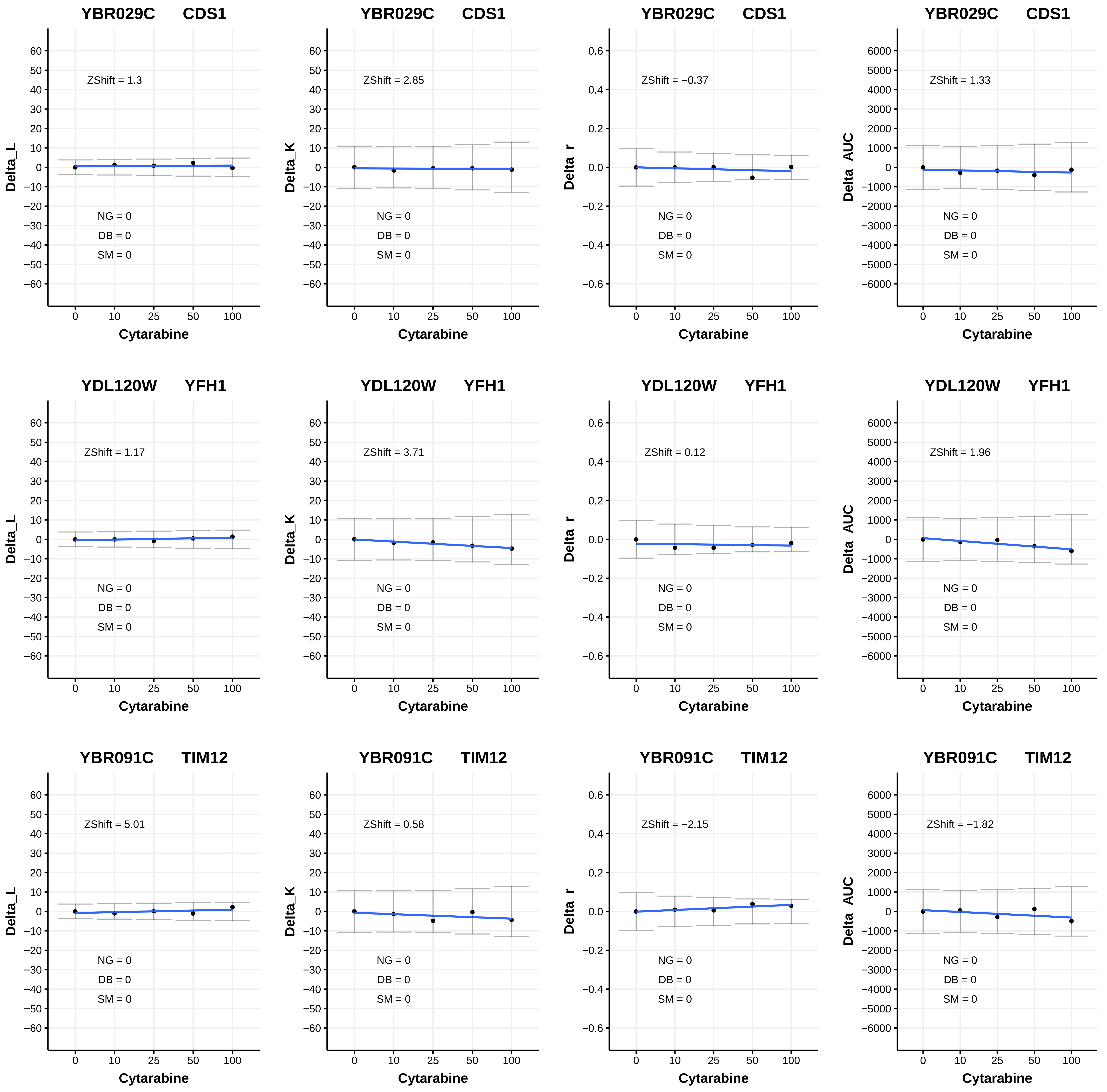

YBR029C CDS1
ZShift = 1.3
NG = 0
DB = 0
SM = 0
Delta_L
Cytarabine
YBR029C CDS1
ZShift = 2.85
NG = 0
DB = 0
SM = 0
Delta_K
Cytarabine
YBR029C CDS1
ZShift = −0.37
NG = 0
DB = 0
SM = 0
Delta_r
Cytarabine
YBR029C CDS1
ZShift = 1.33
NG = 0
DB = 0
SM = 0
Delta_AUC
Cytarabine
YDL120W YFH1
ZShift = 1.17
NG = 0
DB = 0
SM = 0
Delta_L
Cytarabine
YDL120W YFH1
ZShift = 3.71
NG = 0
DB = 0
SM = 0
Delta_K
Cytarabine
YDL120W YFH1
ZShift = 0.12
NG = 0
DB = 0
SM = 0
Delta_r
Cytarabine
YDL120W YFH1
ZShift = 1.96
NG = 0
DB = 0
SM = 0
Delta_AUC
Cytarabine
YBR091C TIM12
ZShift = 5.01
NG = 0
DB = 0
SM = 0
Delta_L
Cytarabine
YBR091C TIM12
ZShift = 0.58
NG = 0
DB = 0
SM = 0
Delta_K
Cytarabine
YBR091C TIM12
ZShift = −2.15
NG = 0
DB = 0
SM = 0
Delta_r
Cytarabine
YBR091C TIM12
ZShift = −1.82
NG = 0
DB = 0
SM = 0
Delta_AUC
Cytarabine

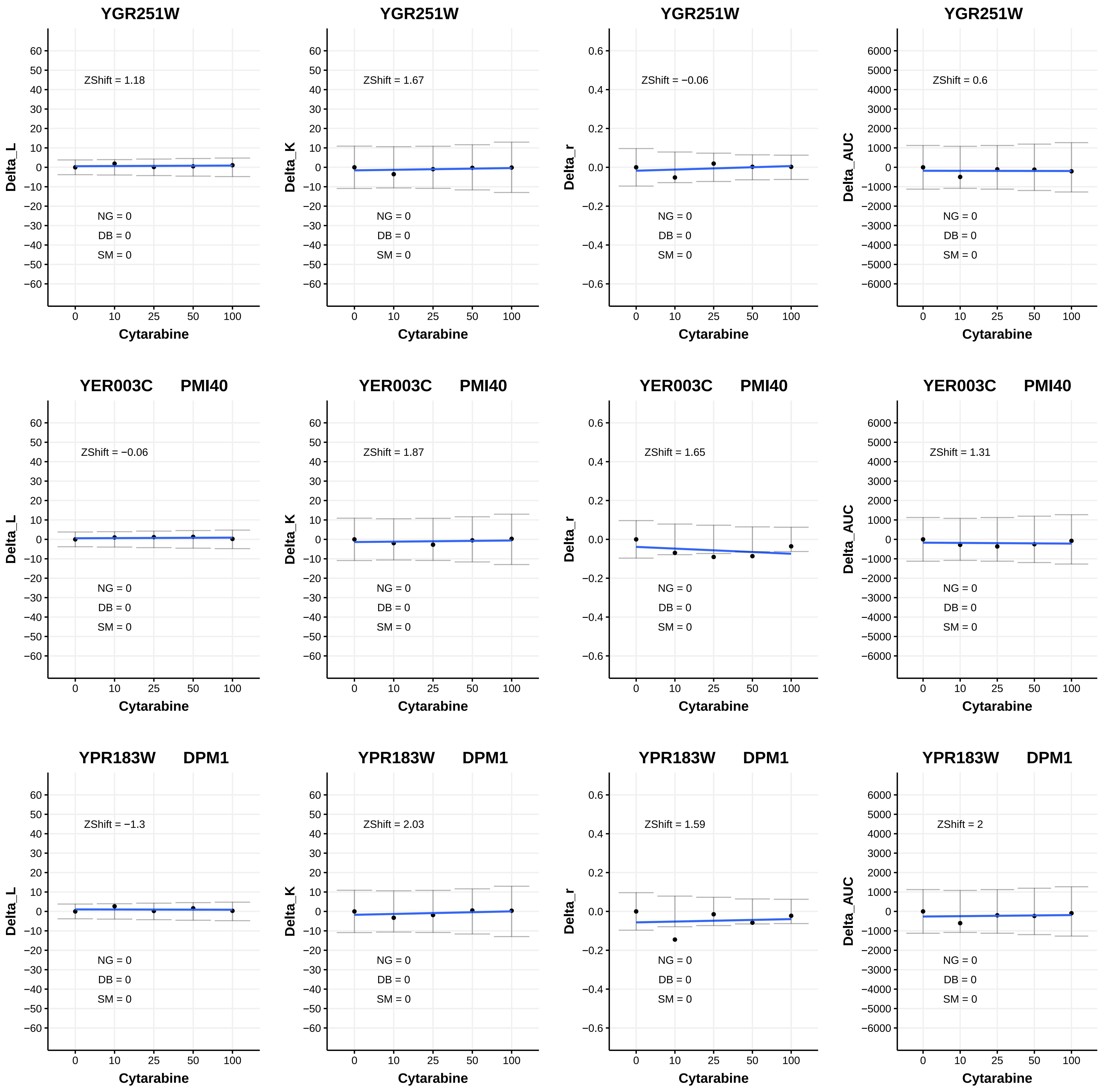
YGR251W
ZShift = 1.18
Delta_L
NG = 0
DB = 0
SM = 0
Cytarabine
YGR251W
ZShift = 1.67
Delta_K
NG = 0
DB = 0
SM = 0
Cytarabine
YGR251W
ZShift = −0.06
Delta_r
NG = 0
DB = 0
SM = 0
Cytarabine
YGR251W
ZShift = 0.6
Delta_AUC
NG = 0
DB = 0
SM = 0
Cytarabine
YER003C PMI40
ZShift = −0.06
Delta_L
NG = 0
DB = 0
SM = 0
Cytarabine
YER003C PMI40
ZShift = 1.87
Delta_K
NG = 0
DB = 0
SM = 0
Cytarabine
YER003C PMI40
ZShift = 1.65
Delta_r
NG = 0
DB = 0
SM = 0
Cytarabine
YER003C PMI40
ZShift = 1.31
Delta_AUC
NG = 0
DB = 0
SM = 0
Cytarabine
YPR183W DPM1
ZShift = −1.3
Delta_L
NG = 0
DB = 0
SM = 0
Cytarabine
YPR183W DPM1
ZShift = 2.03
Delta_K
NG = 0
DB = 0
SM = 0
Cytarabine
YPR183W DPM1
ZShift = 1.59
Delta_r
NG = 0
DB = 0
SM = 0
Cytarabine
YPR183W DPM1
ZShift = 2
Delta_AUC
NG = 0
DB = 0
SM = 0
Cytarabine

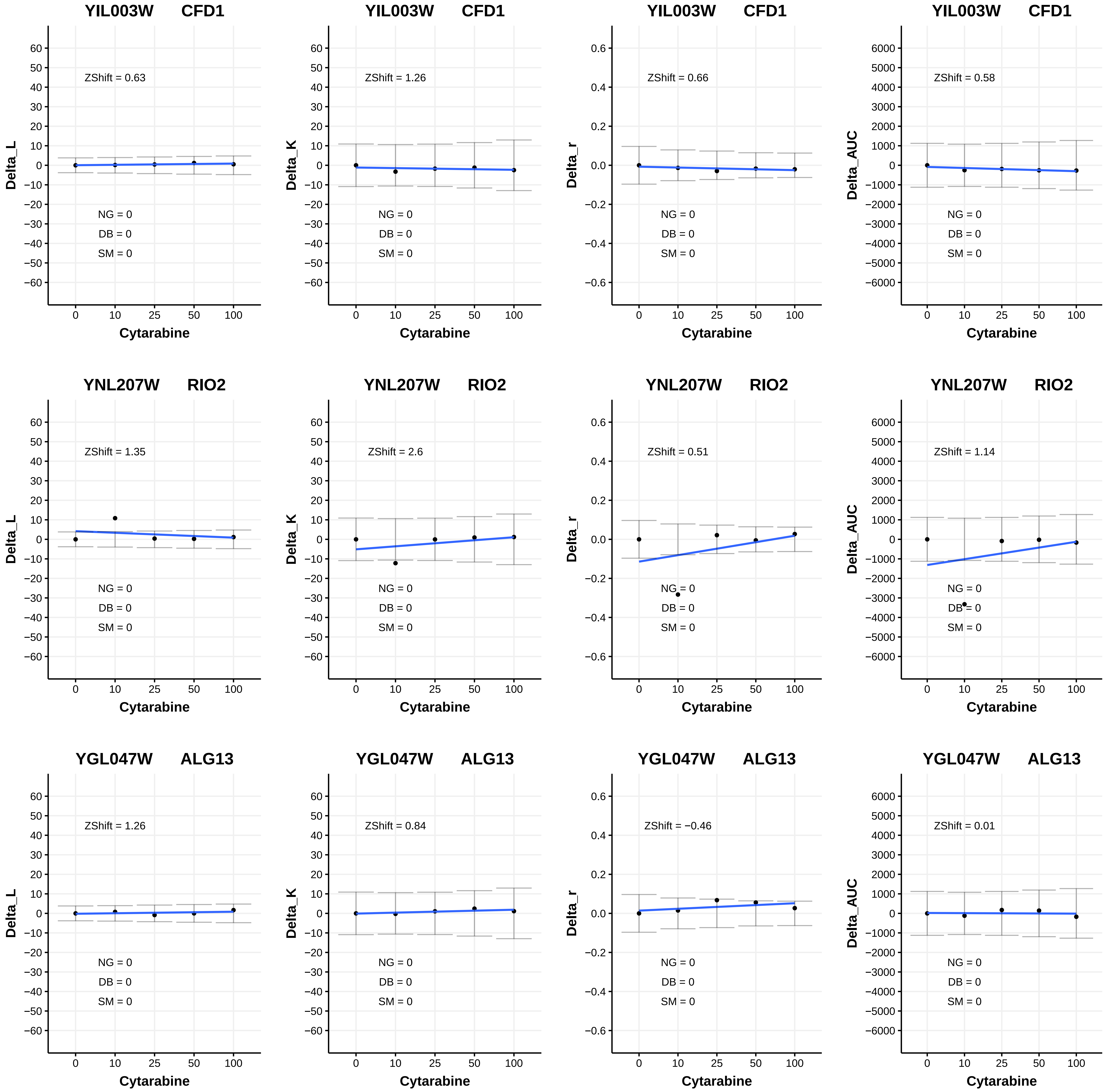
YIL003W CFD1
ZShift = 0.63
NG = 0
DB = 0
SM = 0
Delta_L
Cytarabine
YIL003W CFD1
ZShift = 1.26
NG = 0
DB = 0
SM = 0
Delta_K
Cytarabine
YIL003W CFD1
ZShift = 0.66
NG = 0
DB = 0
SM = 0
Delta_r
Cytarabine
YIL003W CFD1
ZShift = 0.58
NG = 0
DB = 0
SM = 0
Delta_AUC
Cytarabine
YNL207W RIO2
ZShift = 1.35
NG = 0
DB = 0
SM = 0
Delta_L
Cytarabine
YNL207W RIO2
ZShift = 2.6
NG = 0
DB = 0
SM = 0
Delta_K
Cytarabine
YNL207W RIO2
ZShift = 0.51
NG = 0
DB = 0
SM = 0
Delta_r
Cytarabine
YNL207W RIO2
ZShift = 1.14
NG = 0
DB = 0
SM = 0
Delta_AUC
Cytarabine
YGL047W ALG13
ZShift = 1.26
NG = 0
DB = 0
SM = 0
Delta_L
Cytarabine
YGL047W ALG13
ZShift = 0.84
NG = 0
DB = 0
SM = 0
Delta_K
Cytarabine
YGL047W ALG13
ZShift = −0.46
NG = 0
DB = 0
SM = 0
Delta_r
Cytarabine
YGL047W ALG13
ZShift = 0.01
NG = 0
DB = 0
SM = 0
Delta_AUC
Cytarabine

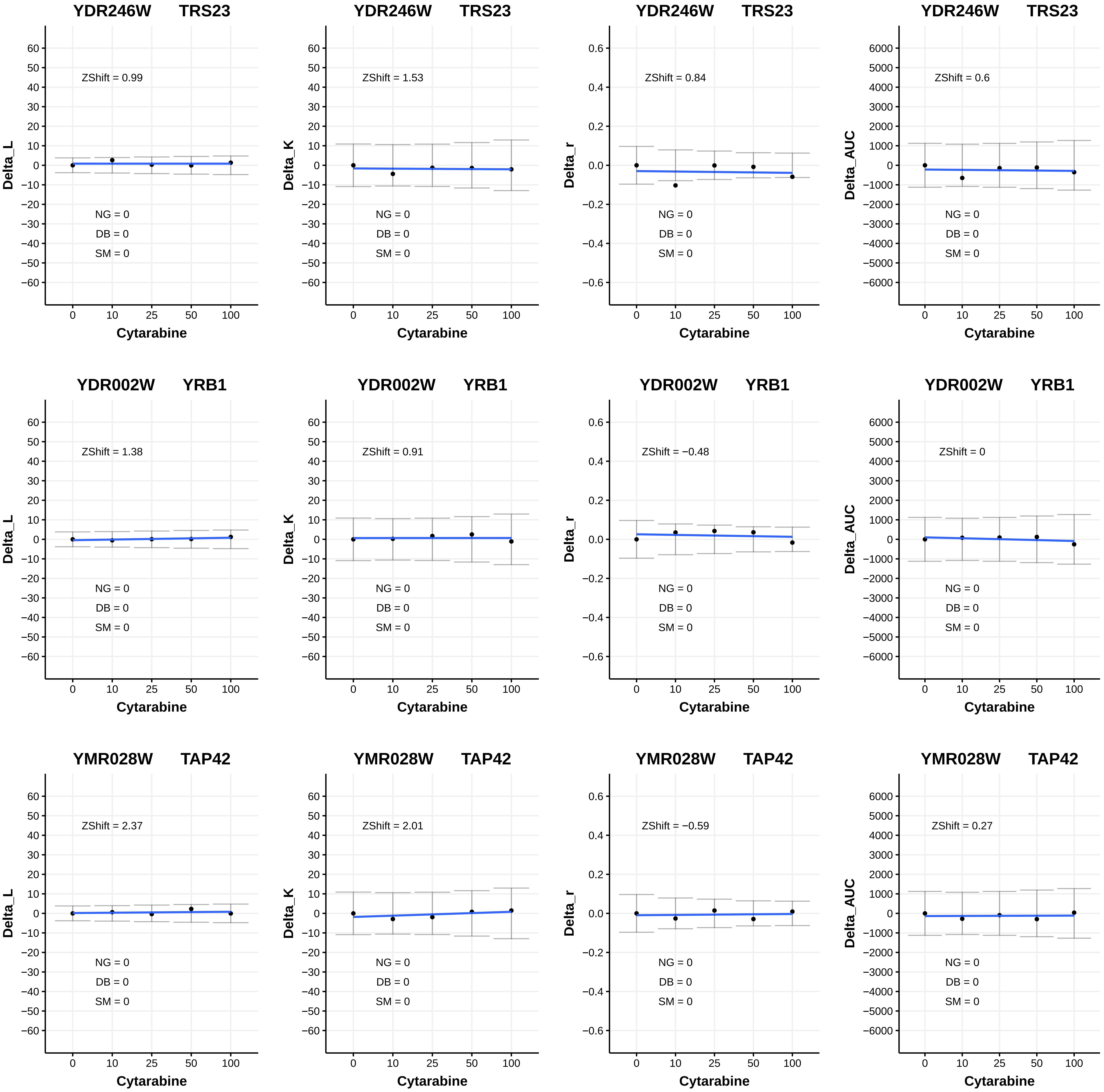
YDR246W TRS23
ZShift = 0.99
ZShift = 1.53
ZShift = 0.84
ZShift = 0.6
YDR002W YRB1
ZShift = 1.38
ZShift = 0.91
ZShift = −0.48
ZShift = 0
YMR028W TAP42
ZShift = 2.37
ZShift = 2.01
ZShift = −0.59
ZShift = 0.27
NG = 0
DB = 0
SM = 0
Delta_L
Delta_K
Delta_r
Delta_AUC
Cytarabine
0
10
25
50
100

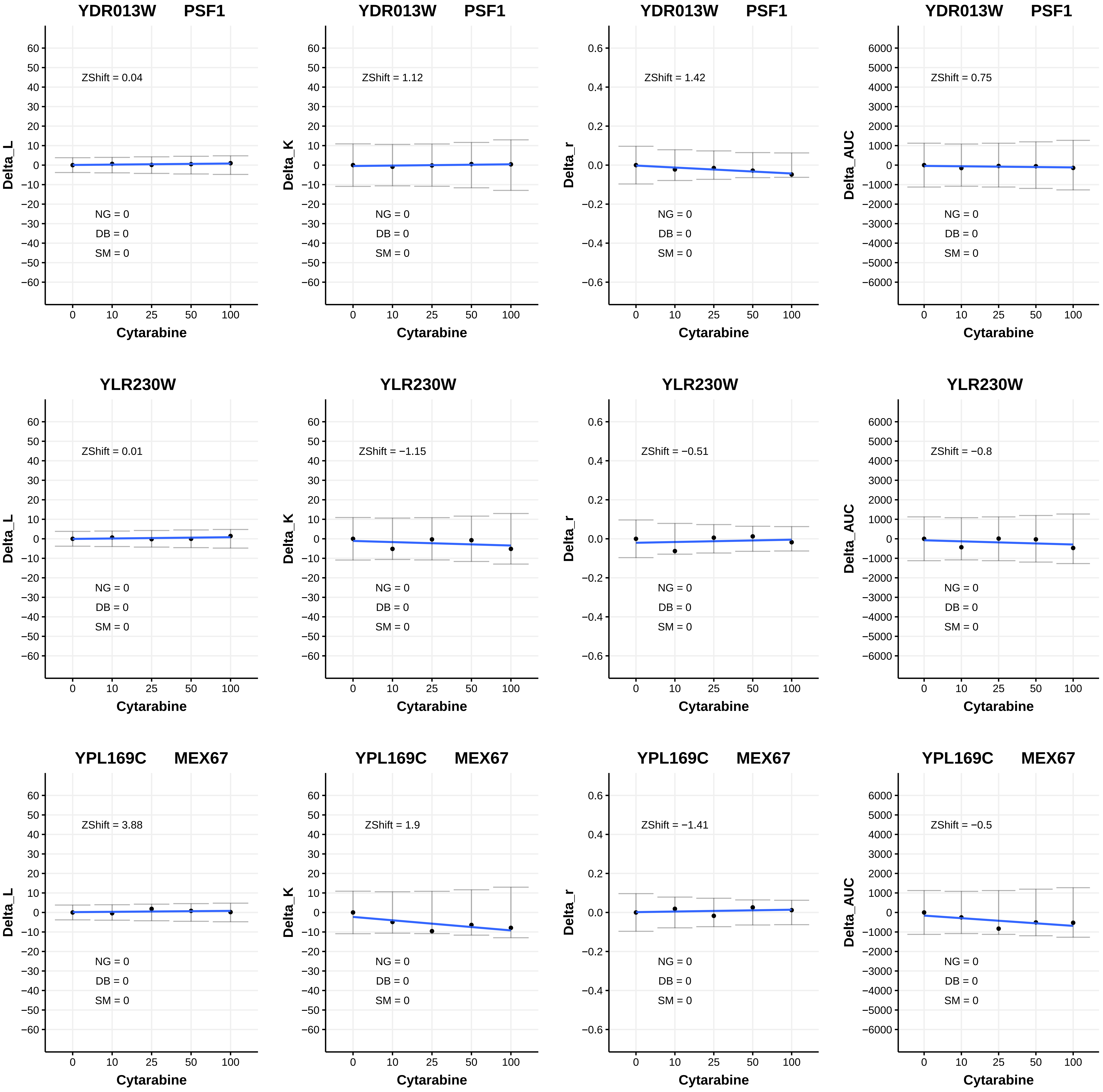

YDR013W PSF1
ZShift = 0.04
NG = 0
DB = 0
SM = 0
Delta_L
Cytarabine
YDR013W PSF1
ZShift = 1.12
NG = 0
DB = 0
SM = 0
Delta_K
Cytarabine
YDR013W PSF1
ZShift = 1.42
NG = 0
DB = 0
SM = 0
Delta_r
Cytarabine
YDR013W PSF1
ZShift = 0.75
NG = 0
DB = 0
SM = 0
Delta_AUC
Cytarabine
YLR230W
ZShift = 0.01
NG = 0
DB = 0
SM = 0
Delta_L
Cytarabine
YLR230W
ZShift = −1.15
NG = 0
DB = 0
SM = 0
Delta_K
Cytarabine
YLR230W
ZShift = −0.51
NG = 0
DB = 0
SM = 0
Delta_r
Cytarabine
YLR230W
ZShift = −0.8
NG = 0
DB = 0
SM = 0
Delta_AUC
Cytarabine
YPL169C MEX67
ZShift = 3.88
NG = 0
DB = 0
SM = 0
Delta_L
Cytarabine
YPL169C MEX67
ZShift = 1.9
NG = 0
DB = 0
SM = 0
Delta_K
Cytarabine
YPL169C MEX67
ZShift = −1.41
NG = 0
DB = 0
SM = 0
Delta_r
Cytarabine
YPL169C MEX67
ZShift = −0.5
NG = 0
DB = 0
SM = 0
Delta_AUC
Cytarabine

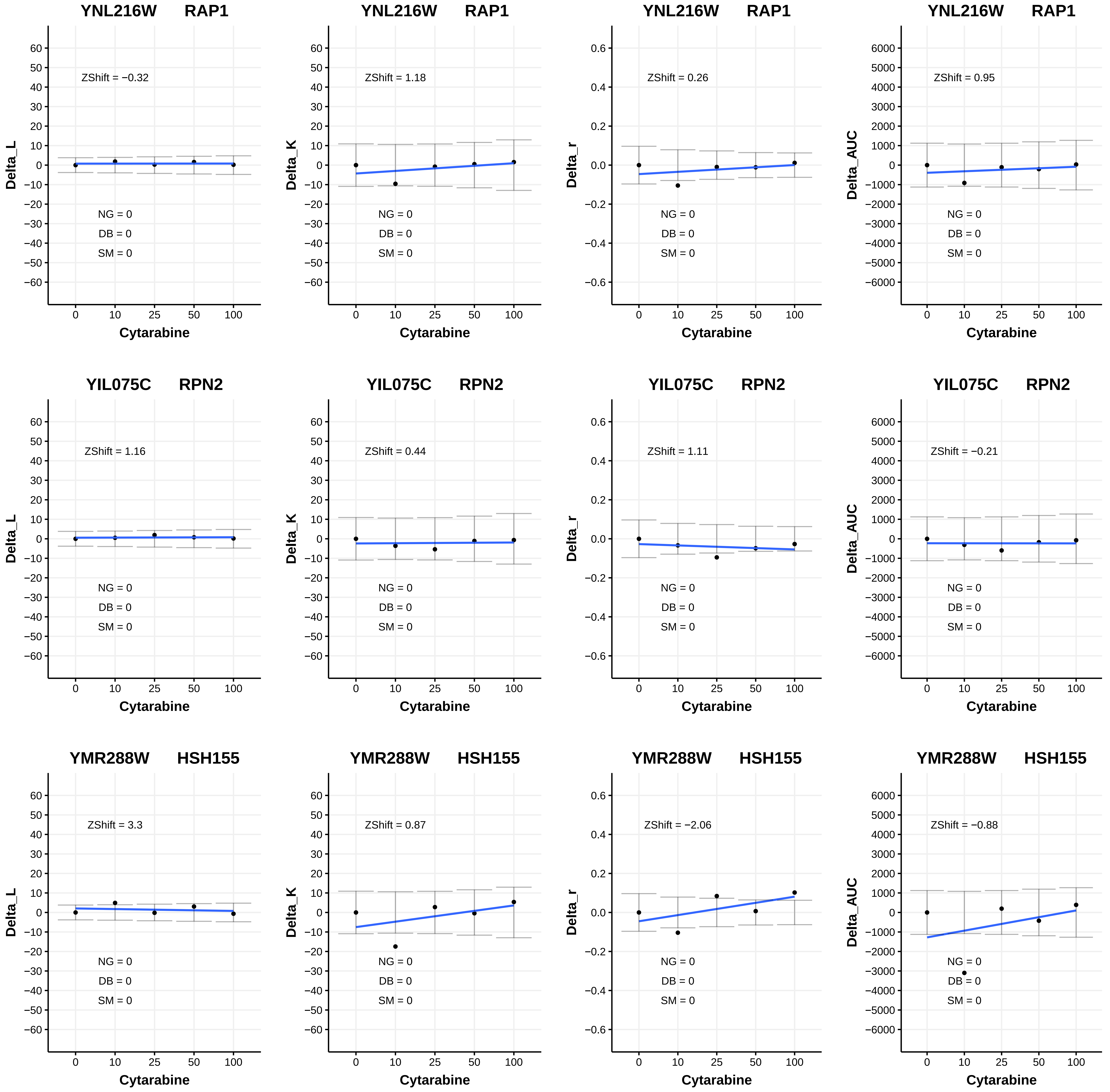
YNL216W RAP1
ZShift = −0.32
NG = 0
DB = 0
SM = 0
Delta_L
Cytarabine
YNL216W RAP1
ZShift = 1.18
NG = 0
DB = 0
SM = 0
Delta_K
Cytarabine
YNL216W RAP1
ZShift = 0.26
NG = 0
DB = 0
SM = 0
Delta_r
Cytarabine
YNL216W RAP1
ZShift = 0.95
NG = 0
DB = 0
SM = 0
Delta_AUC
Cytarabine
YIL075C RPN2
ZShift = 1.16
NG = 0
DB = 0
SM = 0
Delta_L
Cytarabine
YIL075C RPN2
ZShift = 0.44
NG = 0
DB = 0
SM = 0
Delta_K
Cytarabine
YIL075C RPN2
ZShift = 1.11
NG = 0
DB = 0
SM = 0
Delta_r
Cytarabine
YIL075C RPN2
ZShift = −0.21
NG = 0
DB = 0
SM = 0
Delta_AUC
Cytarabine
YMR288W HSH155
ZShift = 3.3
NG = 0
DB = 0
SM = 0
Delta_L
Cytarabine
YMR288W HSH155
ZShift = 0.87
NG = 0
DB = 0
SM = 0
Delta_K
Cytarabine
YMR288W HSH155
ZShift = −2.06
NG = 0
DB = 0
SM = 0
Delta_r
Cytarabine
YMR288W HSH155
ZShift = −0.88
NG = 0
DB = 0
SM = 0
Delta_AUC
Cytarabine

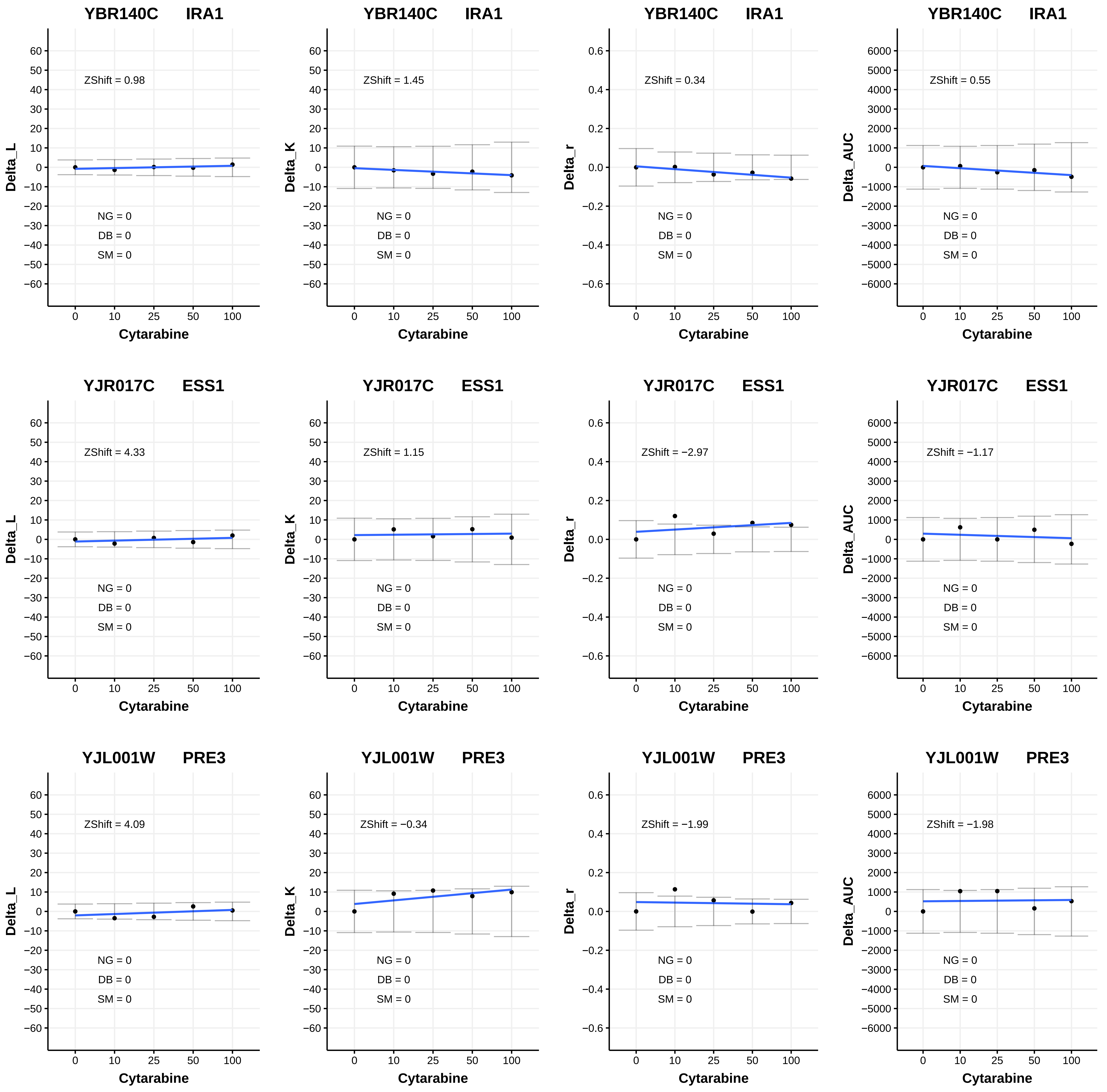
YBR140C IRA1
ZShift = 0.98
NG = 0
DB = 0
SM = 0
Delta_L
Cytarabine
YBR140C IRA1
ZShift = 1.45
NG = 0
DB = 0
SM = 0
Delta_K
Cytarabine
YBR140C IRA1
ZShift = 0.34
NG = 0
DB = 0
SM = 0
Delta_r
Cytarabine
YBR140C IRA1
ZShift = 0.55
NG = 0
DB = 0
SM = 0
Delta_AUC
Cytarabine
YJR017C ESS1
ZShift = 4.33
NG = 0
DB = 0
SM = 0
Delta_L
Cytarabine
YJR017C ESS1
ZShift = 1.15
NG = 0
DB = 0
SM = 0
Delta_K
Cytarabine
YJR017C ESS1
ZShift = −2.97
NG = 0
DB = 0
SM = 0
Delta_r
Cytarabine
YJR017C ESS1
ZShift = −1.17
NG = 0
DB = 0
SM = 0
Delta_AUC
Cytarabine
YJL001W PRE3
ZShift = 4.09
NG = 0
DB = 0
SM = 0
Delta_L
Cytarabine
YJL001W PRE3
ZShift = −0.34
NG = 0
DB = 0
SM = 0
Delta_K
Cytarabine
YJL001W PRE3
ZShift = −1.99
NG = 0
DB = 0
SM = 0
Delta_r
Cytarabine
YJL001W PRE3
ZShift = −1.98
NG = 0
DB = 0
SM = 0
Delta_AUC
Cytarabine

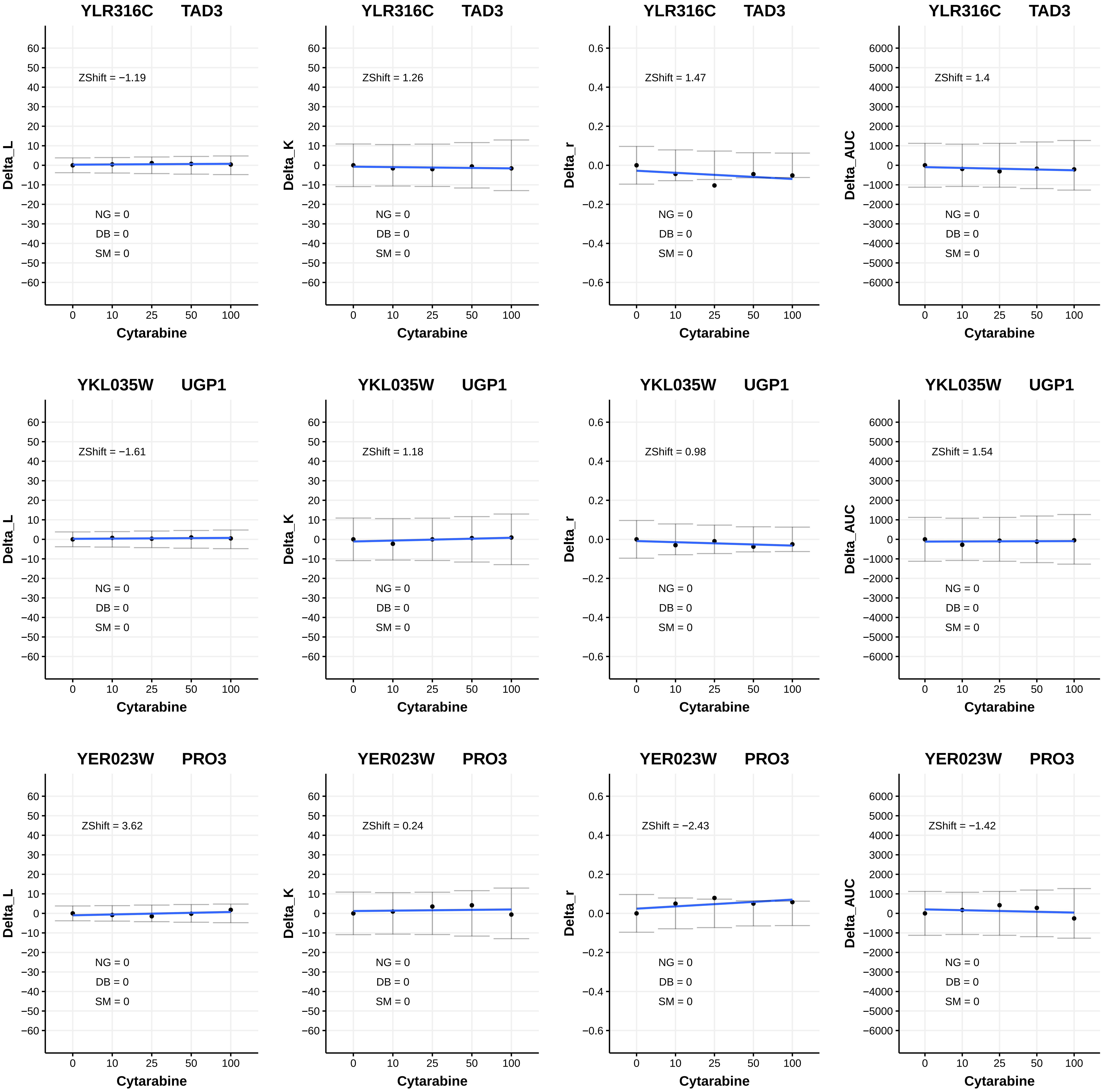
YLR316C TAD3
ZShift = −1.19
NG = 0
DB = 0
SM = 0
Delta_L
Cytarabine
YLR316C TAD3
ZShift = 1.26
NG = 0
DB = 0
SM = 0
Delta_K
Cytarabine
YLR316C TAD3
ZShift = 1.47
NG = 0
DB = 0
SM = 0
Delta_r
Cytarabine
YLR316C TAD3
ZShift = 1.4
NG = 0
DB = 0
SM = 0
Delta_AUC
Cytarabine
YKL035W UGP1
ZShift = −1.61
NG = 0
DB = 0
SM = 0
Delta_L
Cytarabine
YKL035W UGP1
ZShift = 1.18
NG = 0
DB = 0
SM = 0
Delta_K
Cytarabine
YKL035W UGP1
ZShift = 0.98
NG = 0
DB = 0
SM = 0
Delta_r
Cytarabine
YKL035W UGP1
ZShift = 1.54
NG = 0
DB = 0
SM = 0
Delta_AUC
Cytarabine
YER023W PRO3
ZShift = 3.62
NG = 0
DB = 0
SM = 0
Delta_L
Cytarabine
YER023W PRO3
ZShift = 0.24
NG = 0
DB = 0
SM = 0
Delta_K
Cytarabine
YER023W PRO3
ZShift = −2.43
NG = 0
DB = 0
SM = 0
Delta_r
Cytarabine
YER023W PRO3
ZShift = −1.42
NG = 0
DB = 0
SM = 0
Delta_AUC
Cytarabine

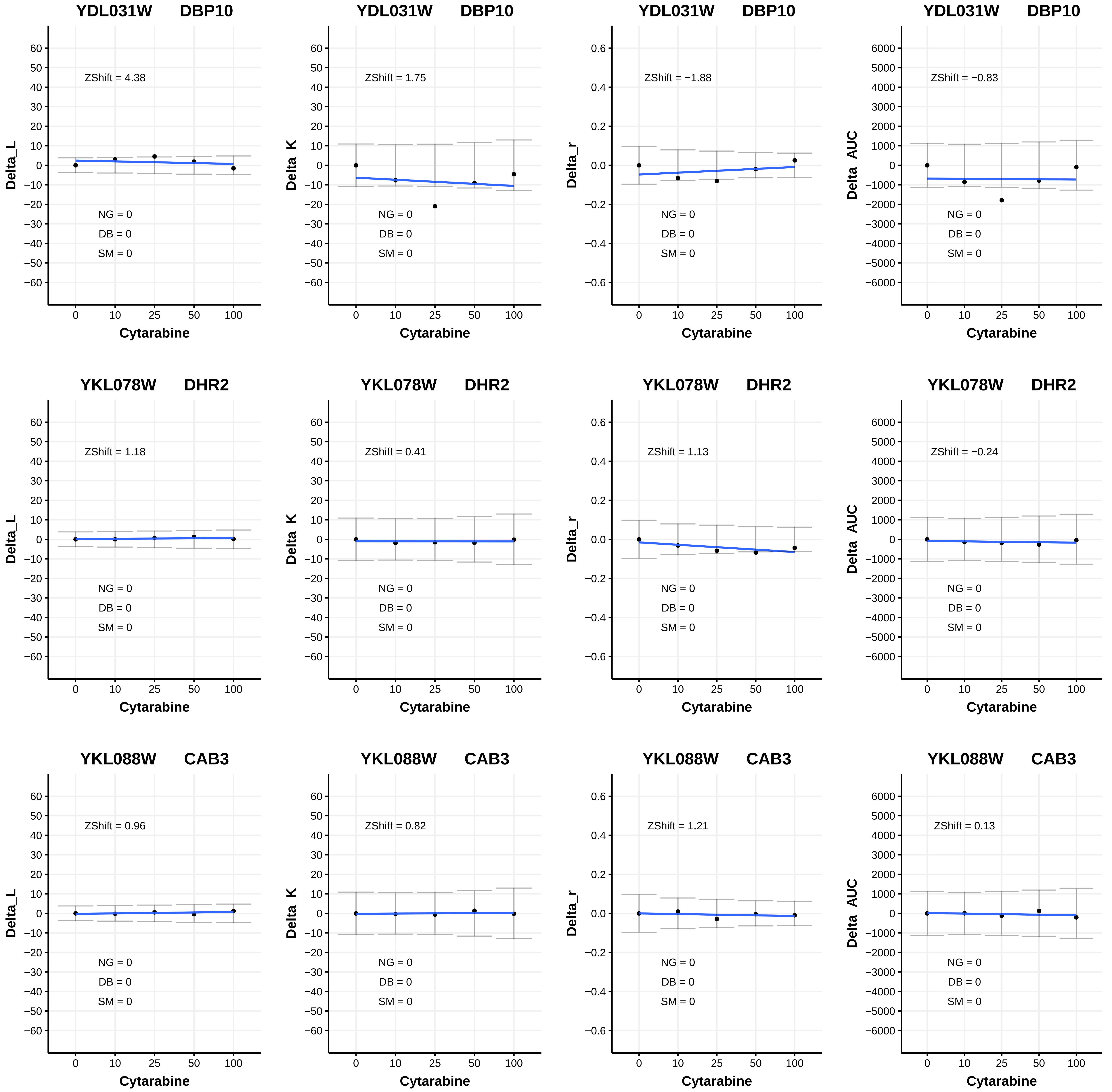

YDL031W DBP10
ZShift = 4.38
NG = 0
DB = 0
SM = 0
Delta_L
Cytarabine
YDL031W DBP10
ZShift = 1.75
NG = 0
DB = 0
SM = 0
Delta_K
Cytarabine
YDL031W DBP10
ZShift = −1.88
NG = 0
DB = 0
SM = 0
Delta_r
Cytarabine
YDL031W DBP10
ZShift = −0.83
NG = 0
DB = 0
SM = 0
Delta_AUC
Cytarabine
YKL078W DHR2
ZShift = 1.18
NG = 0
DB = 0
SM = 0
Delta_L
Cytarabine
YKL078W DHR2
ZShift = 0.41
NG = 0
DB = 0
SM = 0
Delta_K
Cytarabine
YKL078W DHR2
ZShift = 1.13
NG = 0
DB = 0
SM = 0
Delta_r
Cytarabine
YKL078W DHR2
ZShift = −0.24
NG = 0
DB = 0
SM = 0
Delta_AUC
Cytarabine
YKL088W CAB3
ZShift = 0.96
NG = 0
DB = 0
SM = 0
Delta_L
Cytarabine
YKL088W CAB3
ZShift = 0.82
NG = 0
DB = 0
SM = 0
Delta_K
Cytarabine
YKL088W CAB3
ZShift = 1.21
NG = 0
DB = 0
SM = 0
Delta_r
Cytarabine
YKL088W CAB3
ZShift = 0.13
NG = 0
DB = 0
SM = 0
Delta_AUC
Cytarabine

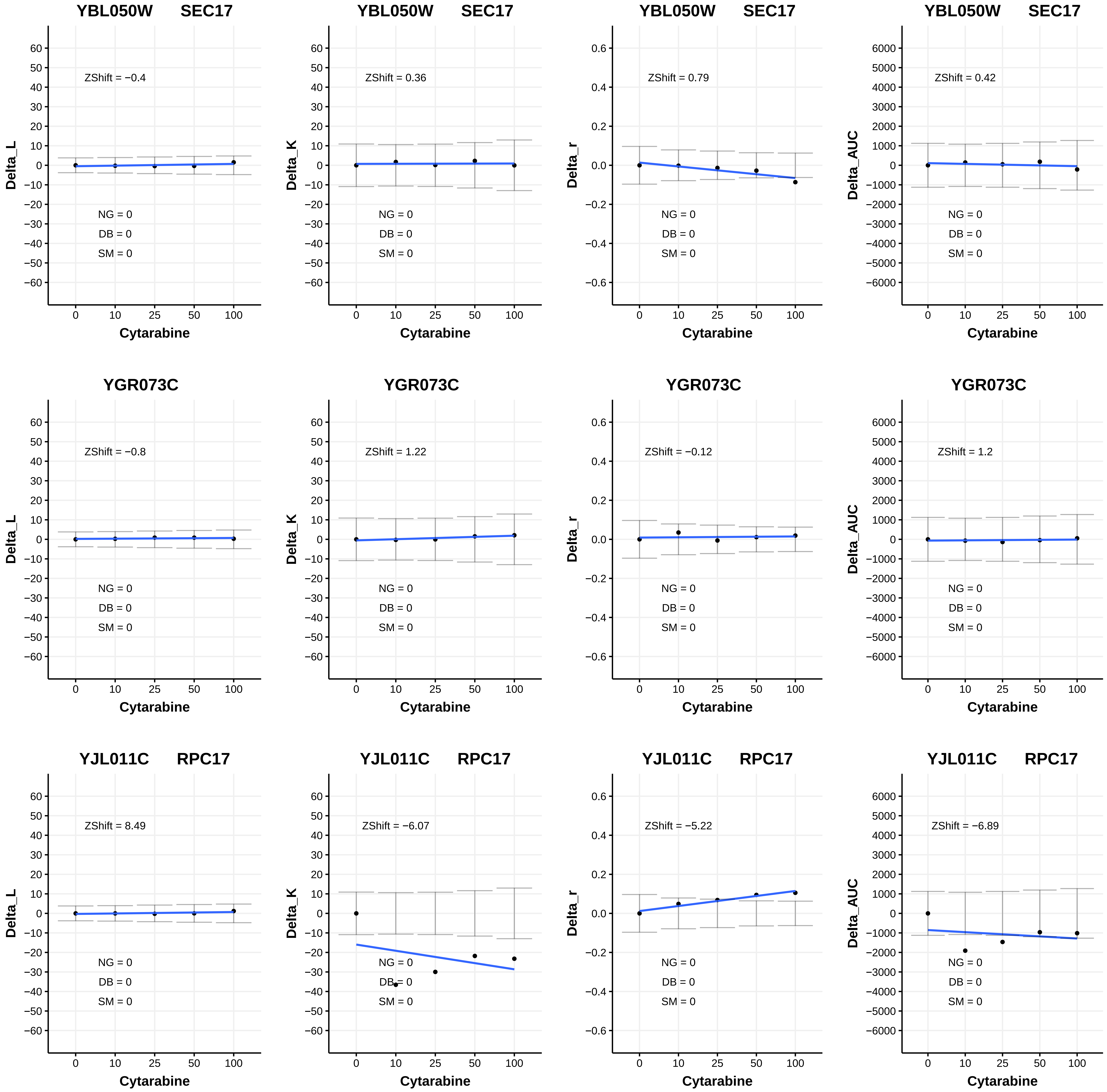
YBL050W SEC17
ZShift = −0.4
ZShift = 0.36
ZShift = 0.79
ZShift = 0.42
YGR073C
ZShift = −0.8
ZShift = 1.22
ZShift = −0.12
ZShift = 1.2
YJL011C RPC17
ZShift = 8.49
ZShift = −6.07
ZShift = −5.22
ZShift = −6.89
NG = 0
DB = 0
SM = 0
Delta_L
Delta_K
Delta_r
Delta_AUC
Cytarabine
0
10
25
50
100

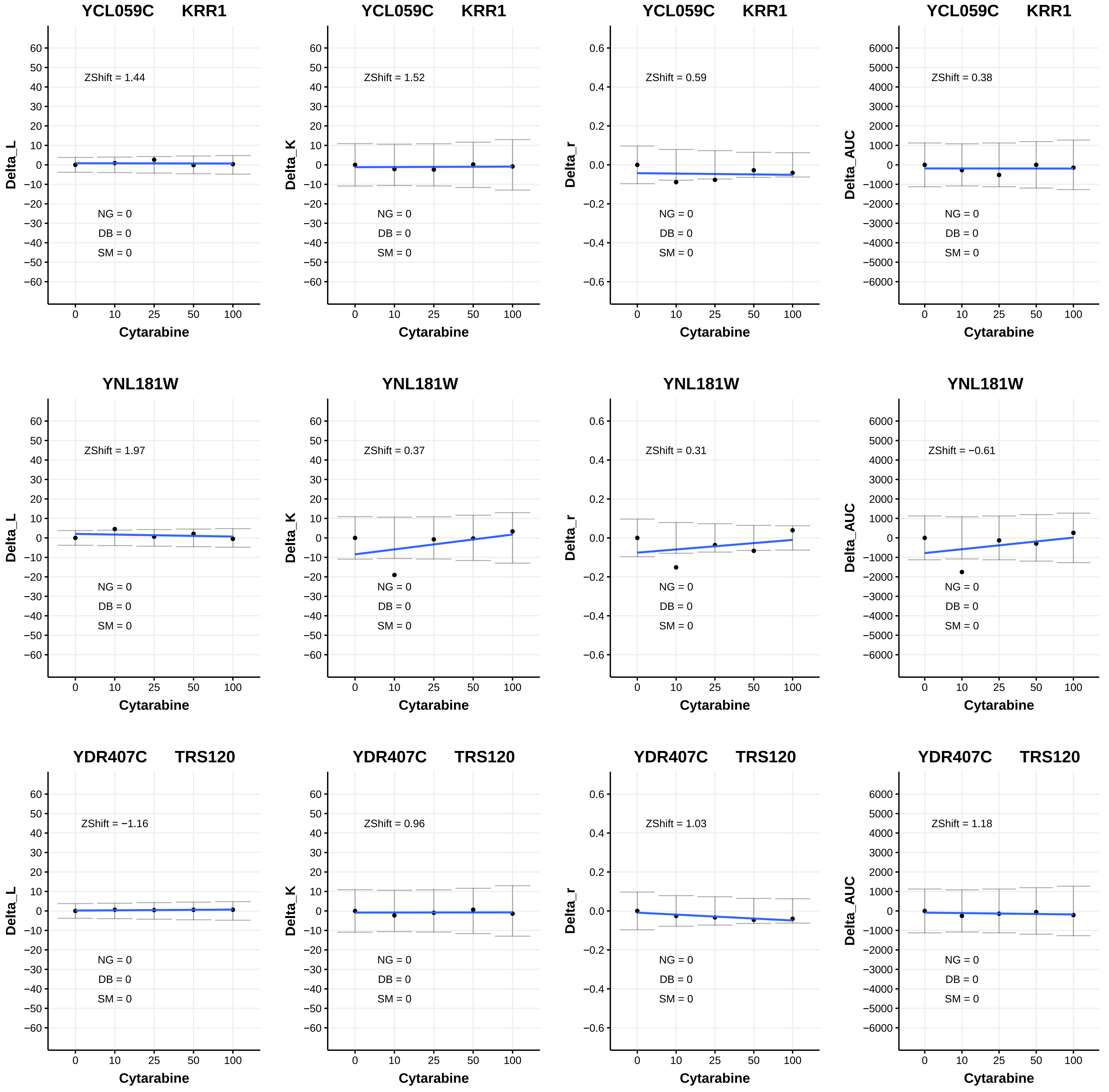

YCL059C KRR1
ZShift = 1.44
NG = 0
DB = 0
SM = 0
Delta_L
Cytarabine
YCL059C KRR1
ZShift = 1.52
NG = 0
DB = 0
SM = 0
Delta_K
Cytarabine
YCL059C KRR1
ZShift = 0.59
NG = 0
DB = 0
SM = 0
Delta_r
Cytarabine
YCL059C KRR1
ZShift = 0.38
NG = 0
DB = 0
SM = 0
Delta_AUC
Cytarabine
YNL181W
ZShift = 1.97
NG = 0
DB = 0
SM = 0
Delta_L
Cytarabine
YNL181W
ZShift = 0.37
NG = 0
DB = 0
SM = 0
Delta_K
Cytarabine
YNL181W
ZShift = 0.31
NG = 0
DB = 0
SM = 0
Delta_r
Cytarabine
YNL181W
ZShift = −0.61
NG = 0
DB = 0
SM = 0
Delta_AUC
Cytarabine
YDR407C TRS120
ZShift = −1.16
NG = 0
DB = 0
SM = 0
Delta_L
Cytarabine
YDR407C TRS120
ZShift = 0.96
NG = 0
DB = 0
SM = 0
Delta_K
Cytarabine
YDR407C TRS120
ZShift = 1.03
NG = 0
DB = 0
SM = 0
Delta_r
Cytarabine
YDR407C TRS120
ZShift = 1.18
NG = 0
DB = 0
SM = 0
Delta_AUC
Cytarabine
0 10 25 50 100

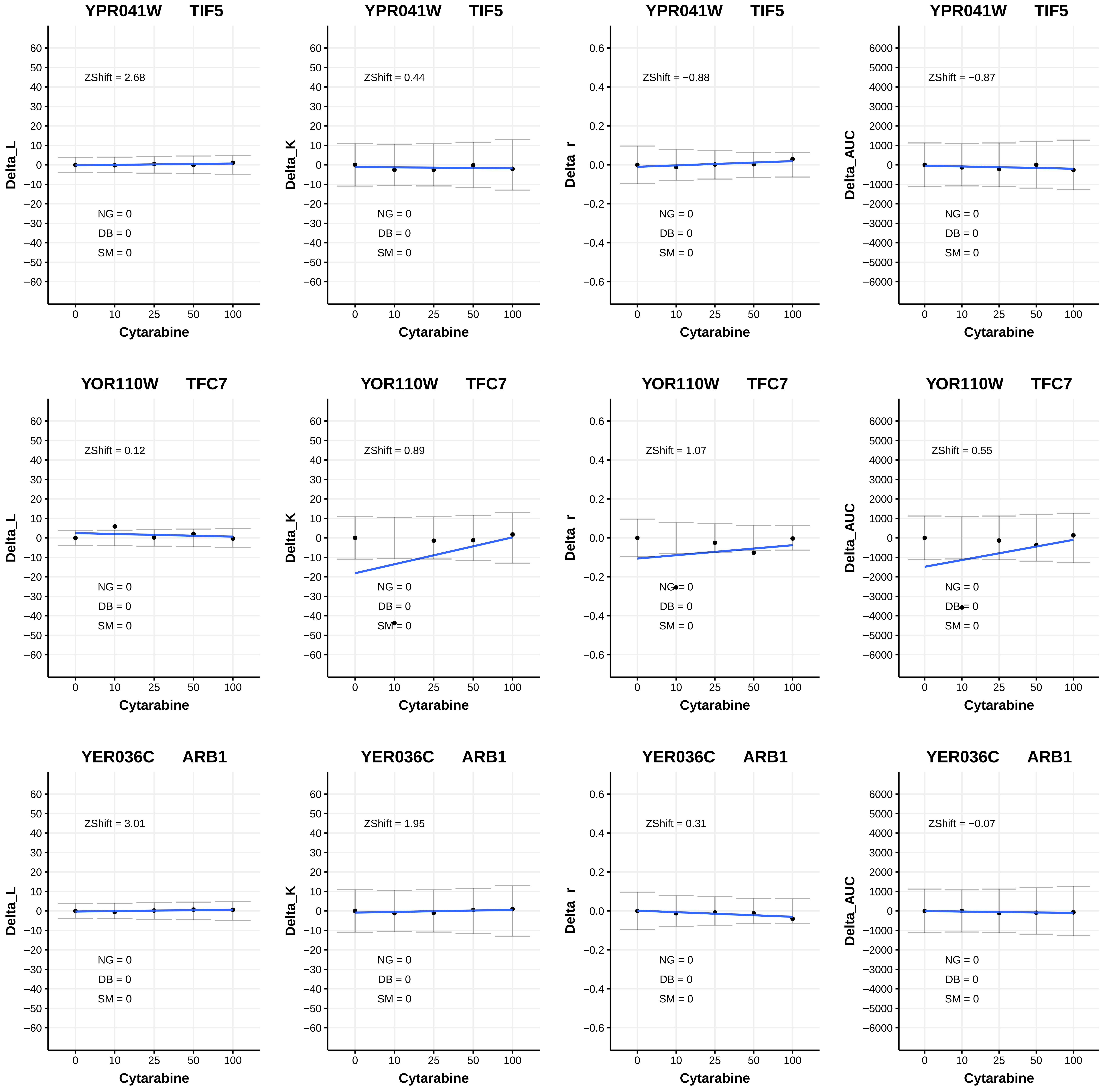

YPR041W TIF5
ZShift = 2.68
ZShift = 0.44
ZShift = −0.88
ZShift = −0.87
YOR110W TFC7
ZShift = 0.12
ZShift = 0.89
ZShift = 1.07
ZShift = 0.55
YER036C ARB1
ZShift = 3.01
ZShift = 1.95
ZShift = 0.31
ZShift = −0.07
NG = 0
DB = 0
SM = 0
Delta_L
Delta_K
Delta_r
Delta_AUC
Cytarabine
0
10
25
50
100

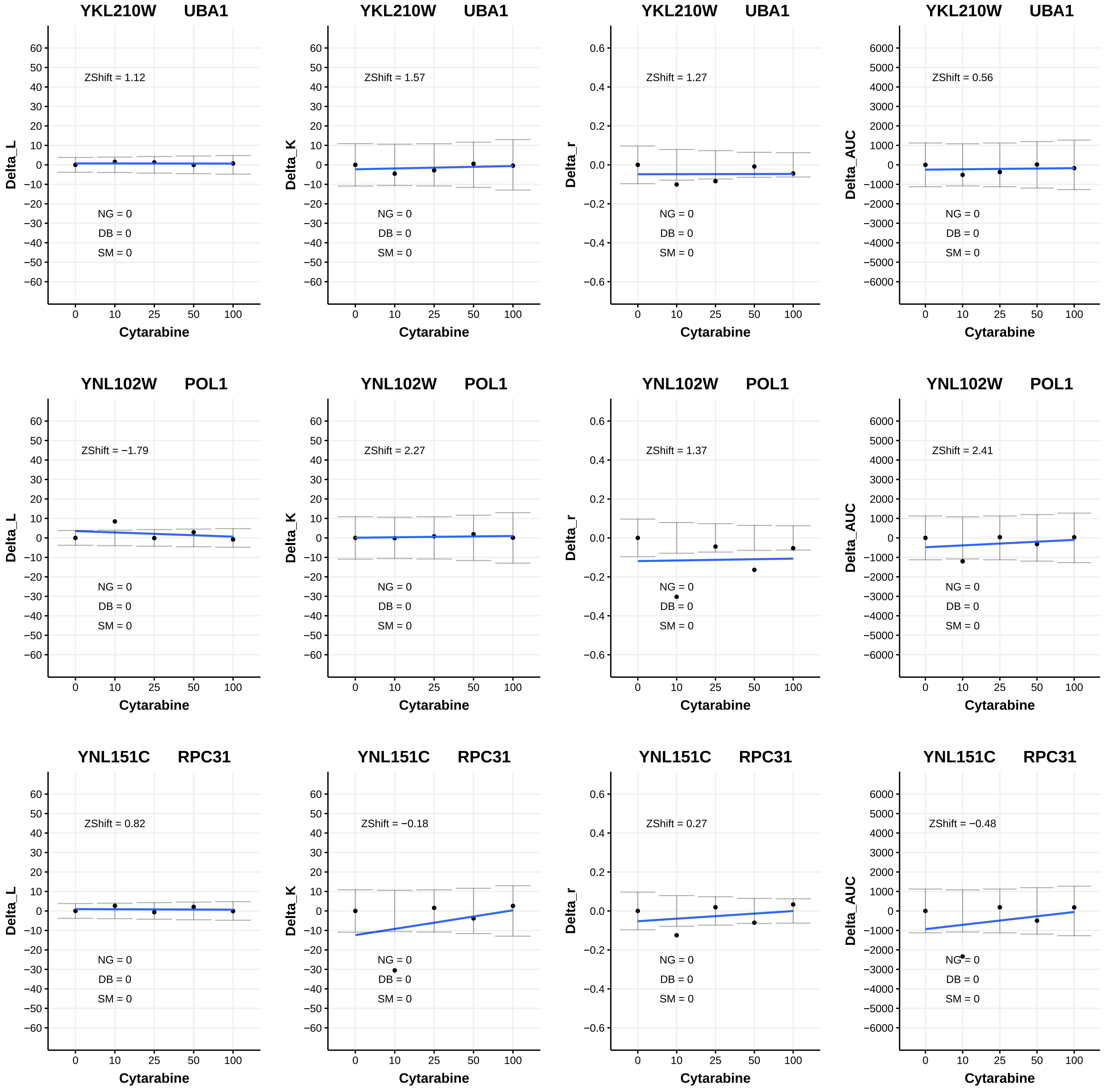

YKL210W UBA1
ZShift = 1.12
NG = 0
DB = 0
SM = 0
Delta_L
60
50
40
30
20
10
0
−10
−20
−30
−40
−50
−60
0
10
25
50
100
Cytarabine
YKL210W UBA1
ZShift = 1.57
NG = 0
DB = 0
SM = 0
Delta_K
YKL210W UBA1
ZShift = 1.27
NG = 0
DB = 0
SM = 0
Delta_r
0.6
0.4
0.2
0.0
−0.2
−0.4
−0.6
YKL210W UBA1
ZShift = 0.56
NG = 0
DB = 0
SM = 0
Delta_AUC
6000
5000
4000
3000
2000
1000
0
−1000
−2000
−3000
−4000
−5000
−6000
YNL102W POL1
ZShift = −1.79
NG = 0
DB = 0
SM = 0
Delta_L
YNL102W POL1
ZShift = 2.27
NG = 0
DB = 0
SM = 0
Delta_K
YNL102W POL1
ZShift = 1.37
NG = 0
DB = 0
SM = 0
Delta_r
YNL102W POL1
ZShift = 2.41
NG = 0
DB = 0
SM = 0
Delta_AUC
YNL151C RPC31
ZShift = 0.82
NG = 0
DB = 0
SM = 0
Delta_L
YNL151C RPC31
ZShift = −0.18
NG = 0
DB = 0
SM = 0
Delta_K
YNL151C RPC31
ZShift = 0.27
NG = 0
DB = 0
SM = 0
Delta_r
YNL151C RPC31
ZShift = −0.48
NG = 0
DB = 0
SM = 0
Delta_AUC

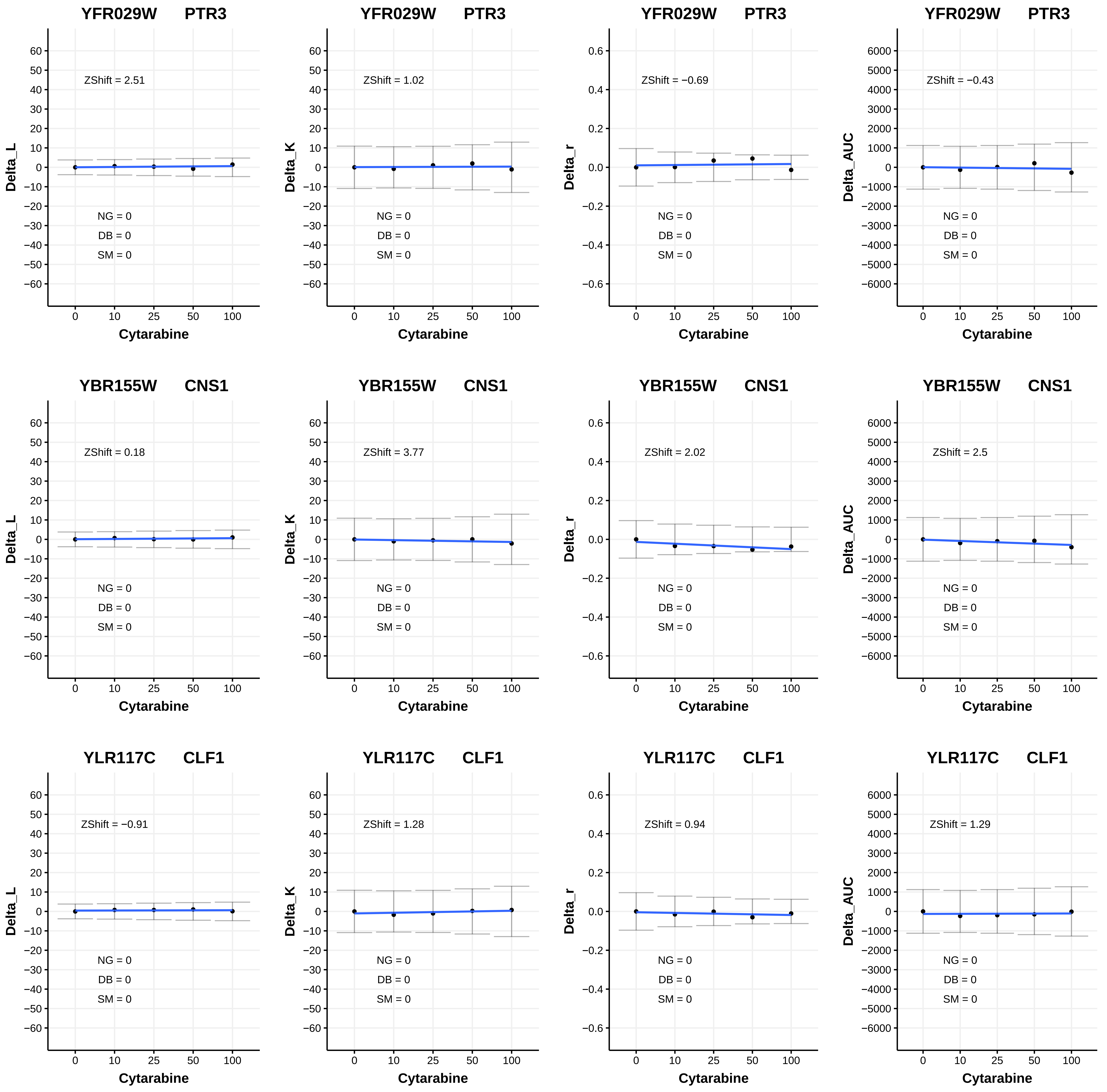
YFR029W PTR3
ZShift = 2.51
ZShift = 1.02
ZShift = −0.69
ZShift = −0.43
YBR155W CNS1
ZShift = 0.18
ZShift = 3.77
ZShift = 2.02
ZShift = 2.5
YLR117C CLF1
ZShift = −0.91
ZShift = 1.28
ZShift = 0.94
ZShift = 1.29
NG = 0
DB = 0
SM = 0
Delta_L
Delta_K
Delta_r
Delta_AUC
Cytarabine
0
10
25
50
100

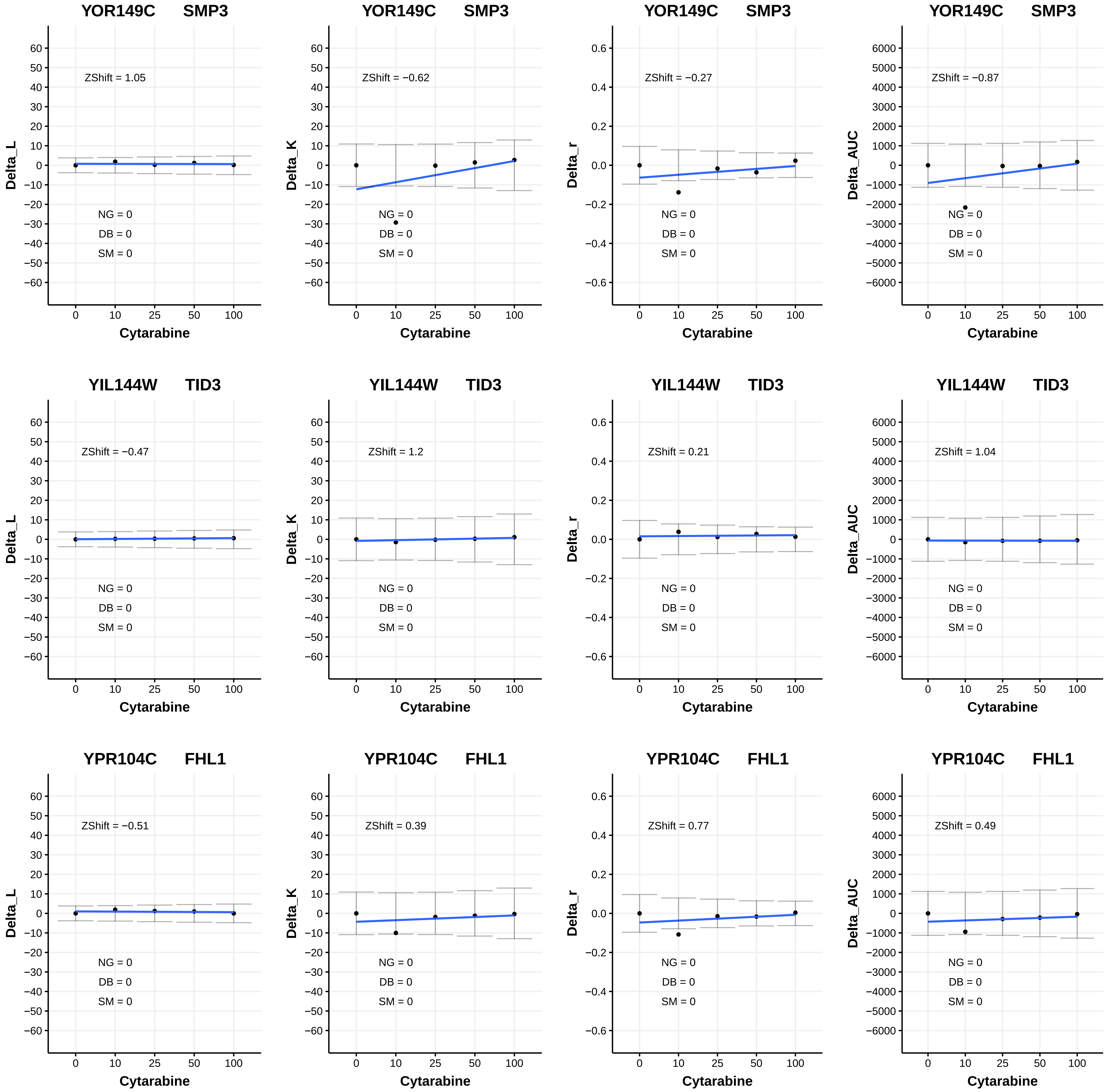

YOR149C SMP3
ZShift = 1.05
ZShift = −0.62
ZShift = −0.27
ZShift = −0.87
YIL144W TID3
ZShift = −0.47
ZShift = 1.2
ZShift = 0.21
ZShift = 1.04
YPR104C FHL1
ZShift = −0.51
ZShift = 0.39
ZShift = 0.77
ZShift = 0.49
NG = 0
DB = 0
SM = 0
Delta_L
Delta_K
Delta_r
Delta_AUC
Cytarabine
0
10
25
50
100

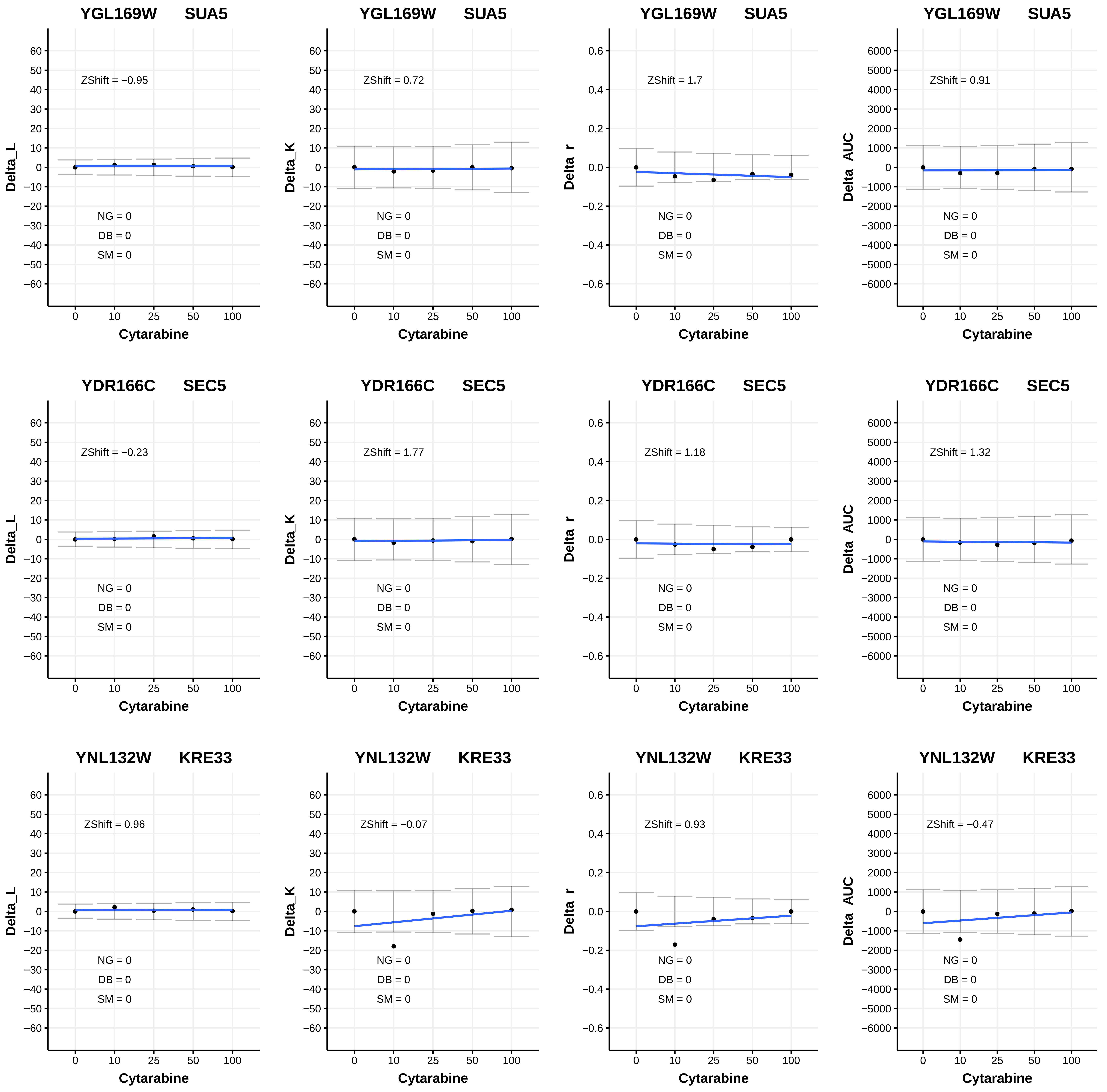
YGL169W SUA5
ZShift = −0.95
NG = 0
DB = 0
SM = 0
Delta_L
Cytarabine
YGL169W SUA5
ZShift = 0.72
NG = 0
DB = 0
SM = 0
Delta_K
Cytarabine
YGL169W SUA5
ZShift = 1.7
NG = 0
DB = 0
SM = 0
Delta_r
Cytarabine
YGL169W SUA5
ZShift = 0.91
NG = 0
DB = 0
SM = 0
Delta_AUC
Cytarabine
YDR166C SEC5
ZShift = −0.23
NG = 0
DB = 0
SM = 0
Delta_L
Cytarabine
YDR166C SEC5
ZShift = 1.77
NG = 0
DB = 0
SM = 0
Delta_K
Cytarabine
YDR166C SEC5
ZShift = 1.18
NG = 0
DB = 0
SM = 0
Delta_r
Cytarabine
YDR166C SEC5
ZShift = 1.32
NG = 0
DB = 0
SM = 0
Delta_AUC
Cytarabine
YNL132W KRE33
ZShift = 0.96
NG = 0
DB = 0
SM = 0
Delta_L
Cytarabine
YNL132W KRE33
ZShift = −0.07
NG = 0
DB = 0
SM = 0
Delta_K
Cytarabine
YNL132W KRE33
ZShift = 0.93
NG = 0
DB = 0
SM = 0
Delta_r
Cytarabine
YNL132W KRE33
ZShift = −0.47
NG = 0
DB = 0
SM = 0
Delta_AUC
Cytarabine

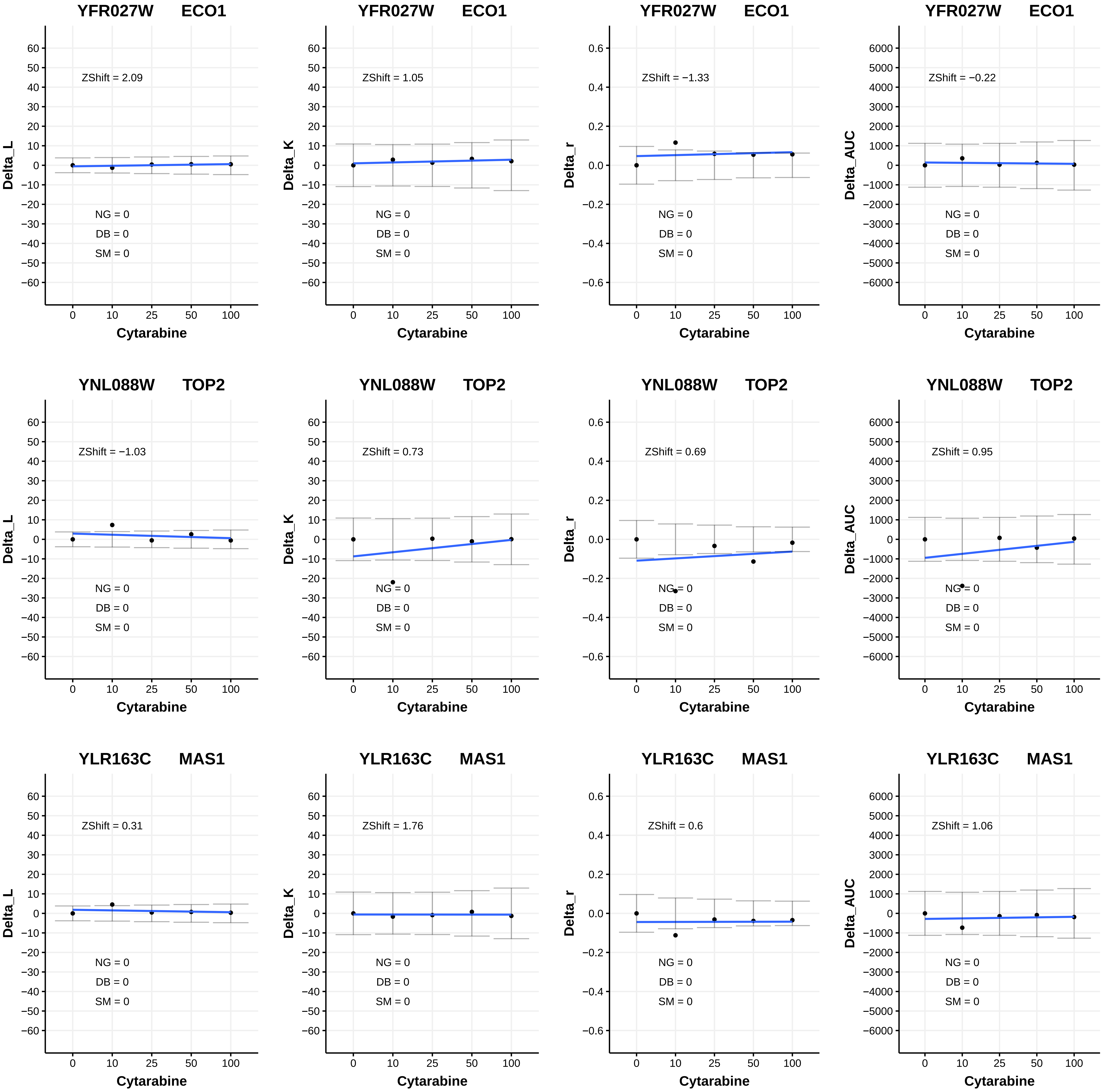
YFR027W ECO1
ZShift = 2.09
Delta_L
NG = 0
DB = 0
SM = 0
Cytarabine
YFR027W ECO1
ZShift = 1.05
Delta_K
NG = 0
DB = 0
SM = 0
Cytarabine
YFR027W ECO1
ZShift = −1.33
Delta_r
NG = 0
DB = 0
SM = 0
Cytarabine
YFR027W ECO1
ZShift = −0.22
Delta_AUC
NG = 0
DB = 0
SM = 0
Cytarabine
YNL088W TOP2
ZShift = −1.03
Delta_L
NG = 0
DB = 0
SM = 0
Cytarabine
YNL088W TOP2
ZShift = 0.73
Delta_K
NG = 0
DB = 0
SM = 0
Cytarabine
YNL088W TOP2
ZShift = 0.69
Delta_r
NG = 0
DB = 0
SM = 0
Cytarabine
YNL088W TOP2
ZShift = 0.95
Delta_AUC
NG = 0
DB = 0
SM = 0
Cytarabine
YLR163C MAS1
ZShift = 0.31
Delta_L
NG = 0
DB = 0
SM = 0
Cytarabine
YLR163C MAS1
ZShift = 1.76
Delta_K
NG = 0
DB = 0
SM = 0
Cytarabine
YLR163C MAS1
ZShift = 0.6
Delta_r
NG = 0
DB = 0
SM = 0
Cytarabine
YLR163C MAS1
ZShift = 1.06
Delta_AUC
NG = 0
DB = 0
SM = 0
Cytarabine

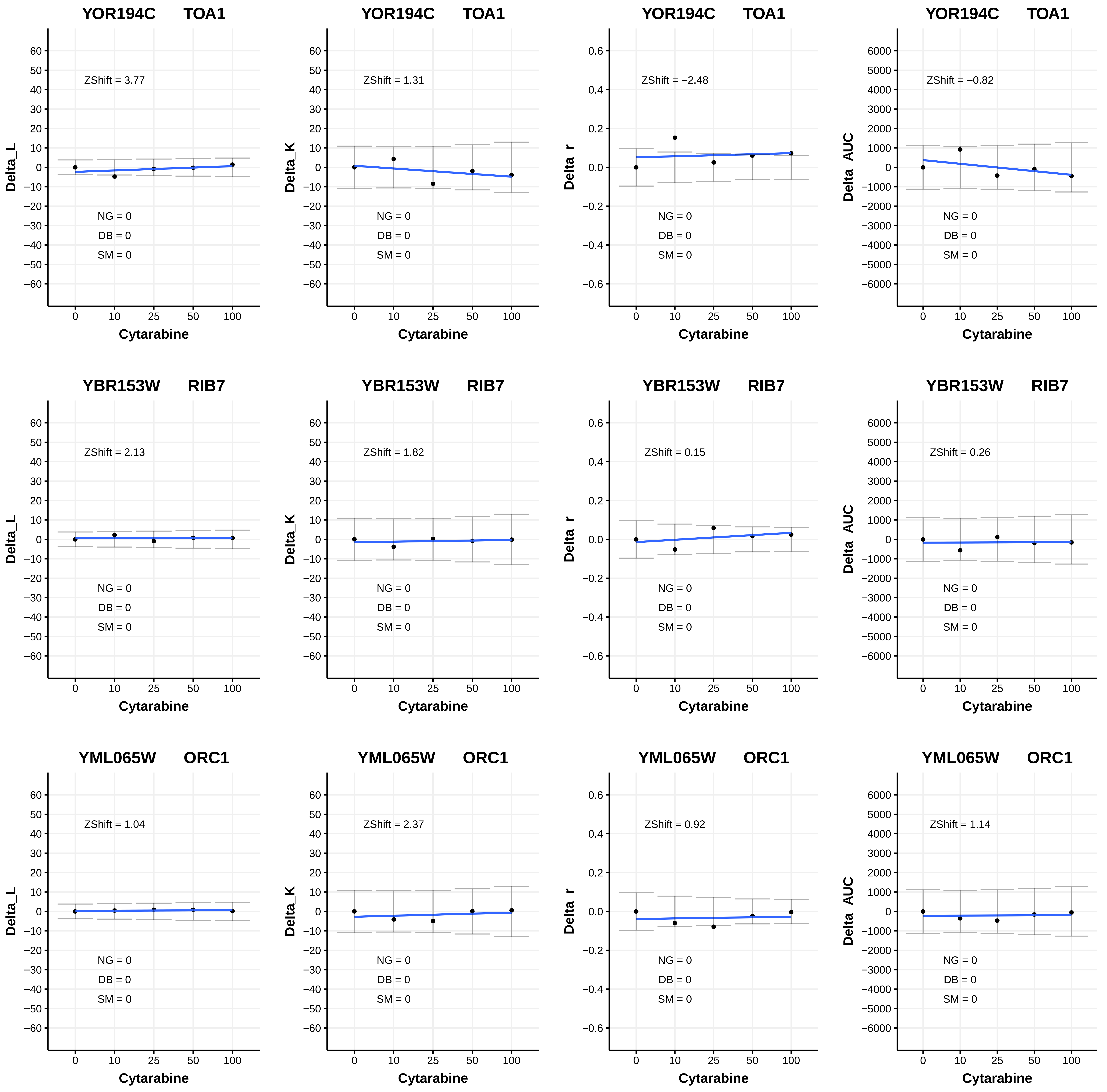
YOR194C TOA1
ZShift = 3.77
ZShift = 1.31
ZShift = −2.48
ZShift = −0.82
YBR153W RIB7
ZShift = 2.13
ZShift = 1.82
ZShift = 0.15
ZShift = 0.26
YML065W ORC1
ZShift = 1.04
ZShift = 2.37
ZShift = 0.92
ZShift = 1.14
NG = 0
DB = 0
SM = 0
Delta_L
Delta_K
Delta_r
Delta_AUC
Cytarabine
0
10
25
50
100

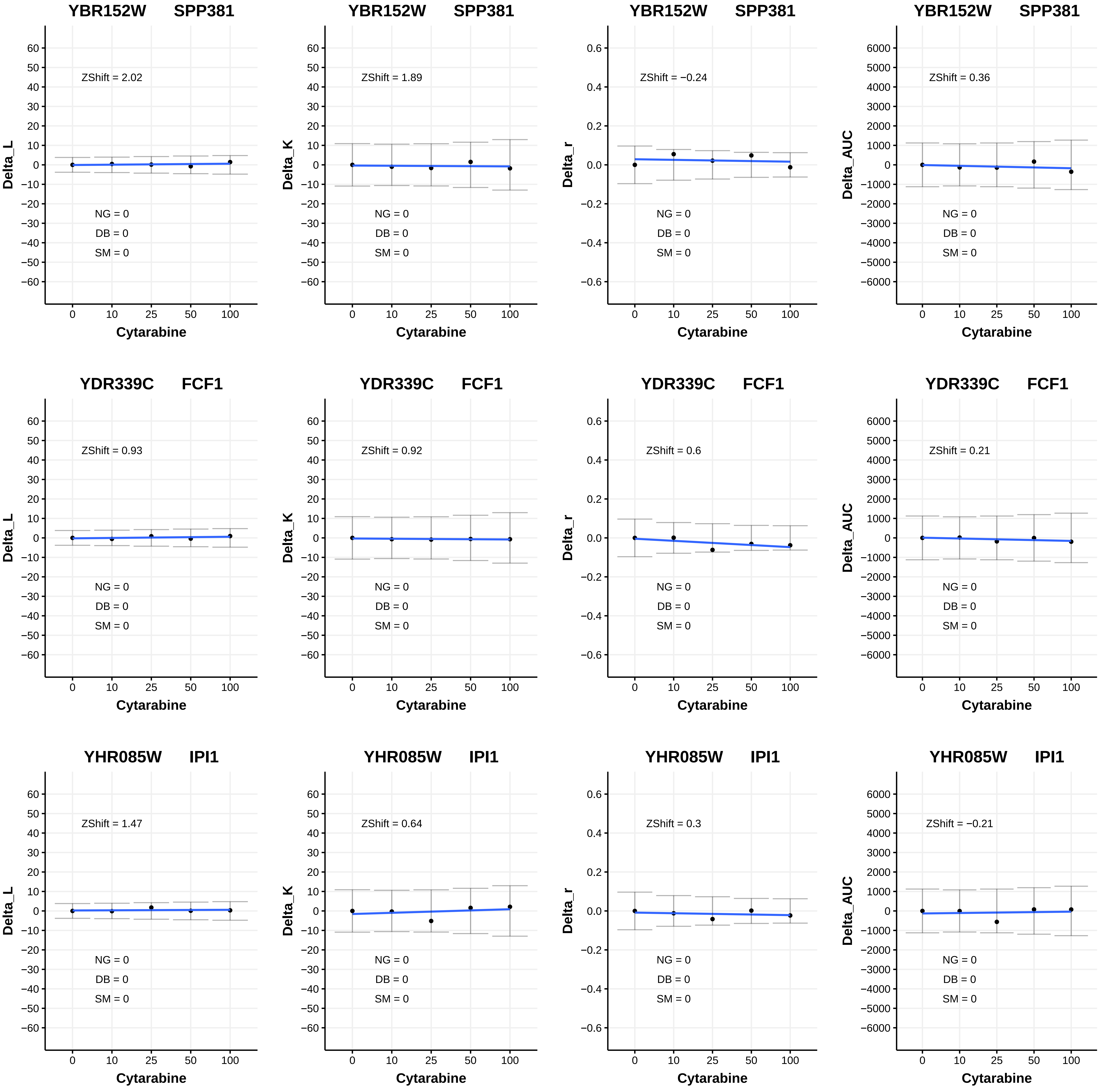
YBR152W SPP381
ZShift = 2.02
Delta_L
Cytarabine
0 10 25 50 100
NG = 0
DB = 0
SM = 0
YBR152W SPP381
ZShift = 1.89
Delta_K
Cytarabine
NG = 0
DB = 0
SM = 0
YBR152W SPP381
ZShift = −0.24
Delta_r
Cytarabine
NG = 0
DB = 0
SM = 0
YBR152W SPP381
ZShift = 0.36
Delta_AUC
Cytarabine
NG = 0
DB = 0
SM = 0
YDR339C FCF1
ZShift = 0.93
Delta_L
Cytarabine
NG = 0
DB = 0
SM = 0
YDR339C FCF1
ZShift = 0.92
Delta_K
Cytarabine
NG = 0
DB = 0
SM = 0
YDR339C FCF1
ZShift = 0.6
Delta_r
Cytarabine
NG = 0
DB = 0
SM = 0
YDR339C FCF1
ZShift = 0.21
Delta_AUC
Cytarabine
NG = 0
DB = 0
SM = 0
YHR085W IPI1
ZShift = 1.47
Delta_L
Cytarabine
NG = 0
DB = 0
SM = 0
YHR085W IPI1
ZShift = 0.64
Delta_K
Cytarabine
NG = 0
DB = 0
SM = 0
YHR085W IPI1
ZShift = 0.3
Delta_r
Cytarabine
NG = 0
DB = 0
SM = 0
YHR085W IPI1
ZShift = −0.21
Delta_AUC
Cytarabine
NG = 0
DB = 0
SM = 0

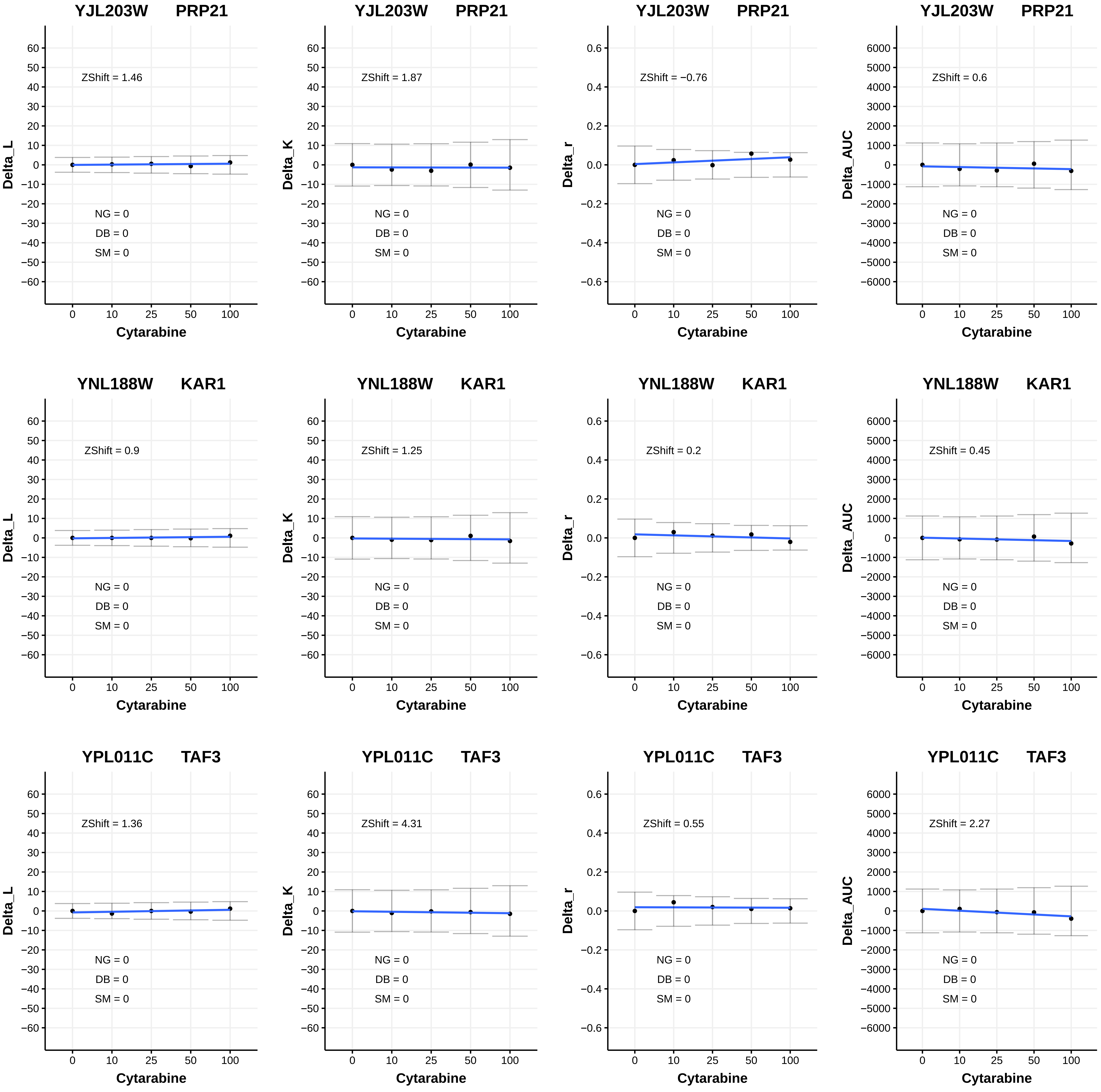
YJL203W PRP21
ZShift = 1.46
NG = 0
DB = 0
SM = 0
Delta_L
Cytarabine
YJL203W PRP21
ZShift = 1.87
NG = 0
DB = 0
SM = 0
Delta_K
Cytarabine
YJL203W PRP21
ZShift = −0.76
NG = 0
DB = 0
SM = 0
Delta_r
Cytarabine
YJL203W PRP21
ZShift = 0.6
NG = 0
DB = 0
SM = 0
Delta_AUC
Cytarabine
YNL188W KAR1
ZShift = 0.9
NG = 0
DB = 0
SM = 0
Delta_L
Cytarabine
YNL188W KAR1
ZShift = 1.25
NG = 0
DB = 0
SM = 0
Delta_K
Cytarabine
YNL188W KAR1
ZShift = 0.2
NG = 0
DB = 0
SM = 0
Delta_r
Cytarabine
YNL188W KAR1
ZShift = 0.45
NG = 0
DB = 0
SM = 0
Delta_AUC
Cytarabine
YPL011C TAF3
ZShift = 1.36
NG = 0
DB = 0
SM = 0
Delta_L
Cytarabine
YPL011C TAF3
ZShift = 4.31
NG = 0
DB = 0
SM = 0
Delta_K
Cytarabine
YPL011C TAF3
ZShift = 0.55
NG = 0
DB = 0
SM = 0
Delta_r
Cytarabine
YPL011C TAF3
ZShift = 2.27
NG = 0
DB = 0
SM = 0
Delta_AUC
Cytarabine
0 10 25 50 100

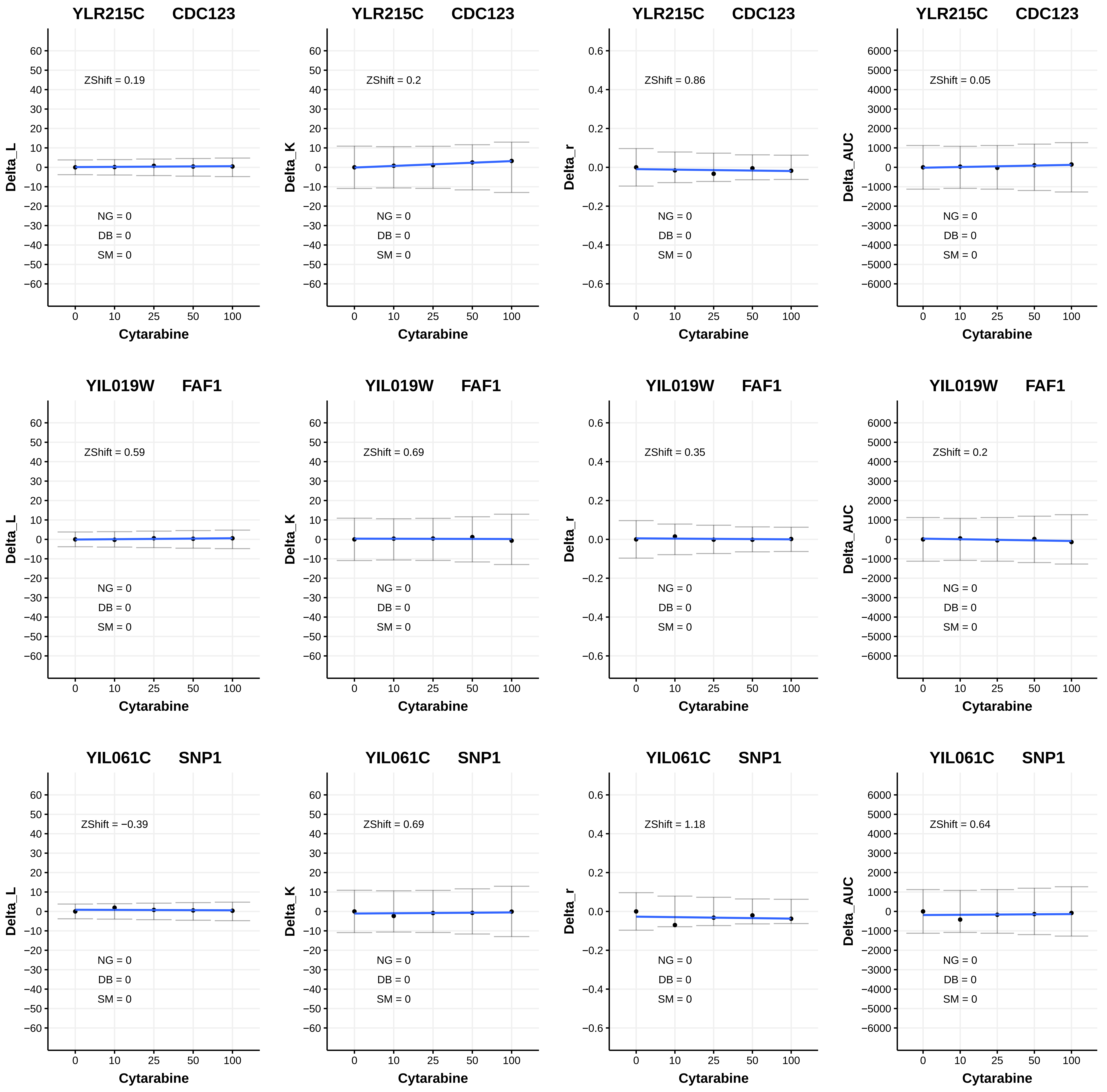

YLR215C CDC123
ZShift = 0.19
NG = 0
DB = 0
SM = 0
Delta_L
Cytarabine
YLR215C CDC123
ZShift = 0.2
NG = 0
DB = 0
SM = 0
Delta_K
Cytarabine
YLR215C CDC123
ZShift = 0.86
NG = 0
DB = 0
SM = 0
Delta_r
Cytarabine
YLR215C CDC123
ZShift = 0.05
NG = 0
DB = 0
SM = 0
Delta_AUC
Cytarabine
YIL019W FAF1
ZShift = 0.59
NG = 0
DB = 0
SM = 0
Delta_L
Cytarabine
YIL019W FAF1
ZShift = 0.69
NG = 0
DB = 0
SM = 0
Delta_K
Cytarabine
YIL019W FAF1
ZShift = 0.35
NG = 0
DB = 0
SM = 0
Delta_r
Cytarabine
YIL019W FAF1
ZShift = 0.2
NG = 0
DB = 0
SM = 0
Delta_AUC
Cytarabine
YIL061C SNP1
ZShift = −0.39
NG = 0
DB = 0
SM = 0
Delta_L
Cytarabine
YIL061C SNP1
ZShift = 0.69
NG = 0
DB = 0
SM = 0
Delta_K
Cytarabine
YIL061C SNP1
ZShift = 1.18
NG = 0
DB = 0
SM = 0
Delta_r
Cytarabine
YIL061C SNP1
ZShift = 0.64
NG = 0
DB = 0
SM = 0
Delta_AUC
Cytarabine

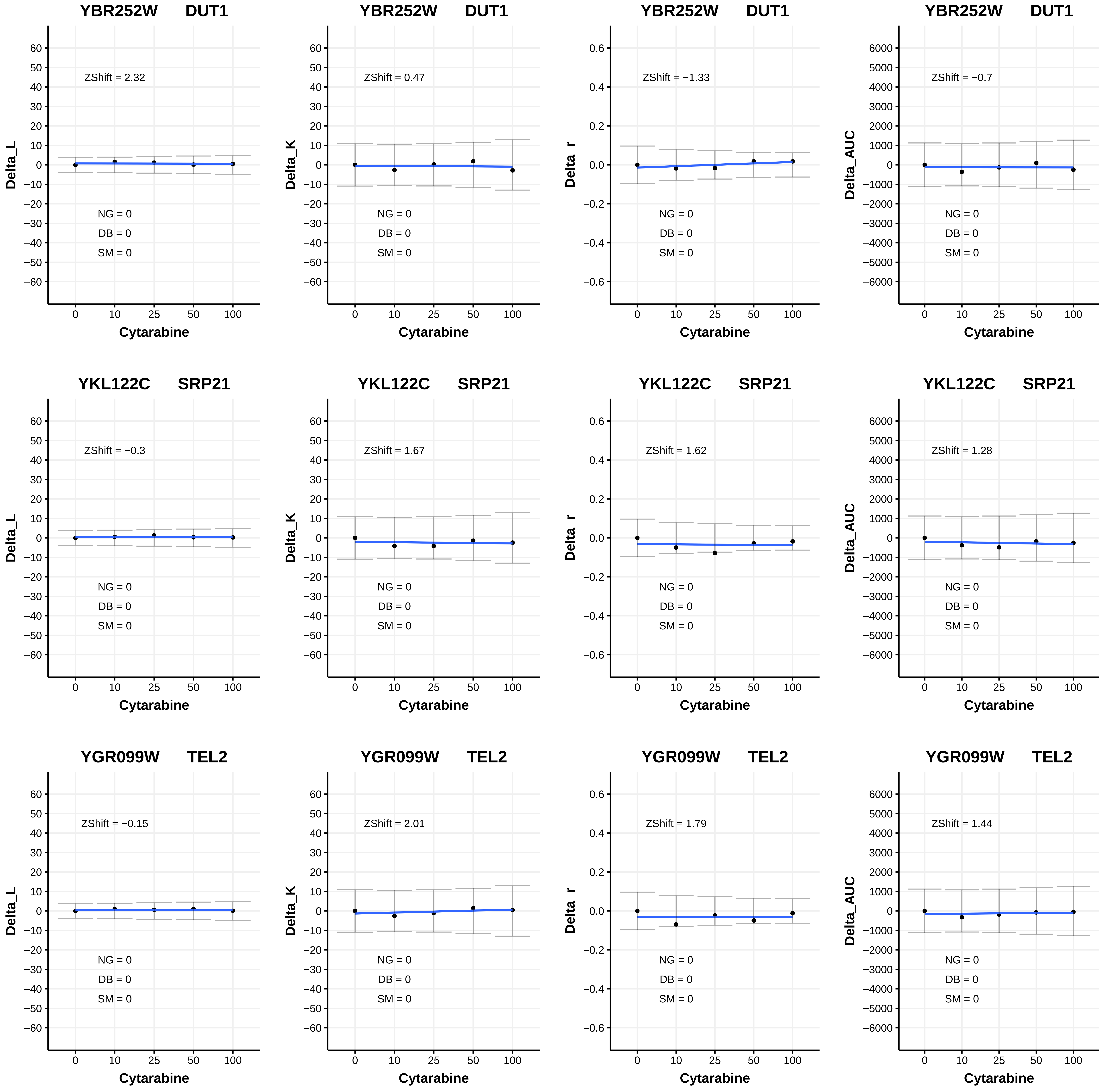
YBR252W DUT1
ZShift = 2.32
ZShift = 0.47
ZShift = −1.33
ZShift = −0.7
YKL122C SRP21
ZShift = −0.3
ZShift = 1.67
ZShift = 1.62
ZShift = 1.28
YGR099W TEL2
ZShift = −0.15
ZShift = 2.01
ZShift = 1.79
ZShift = 1.44
NG = 0
DB = 0
SM = 0
Delta_L
Delta_K
Delta_r
Delta_AUC
Cytarabine
0
10
25
50
100

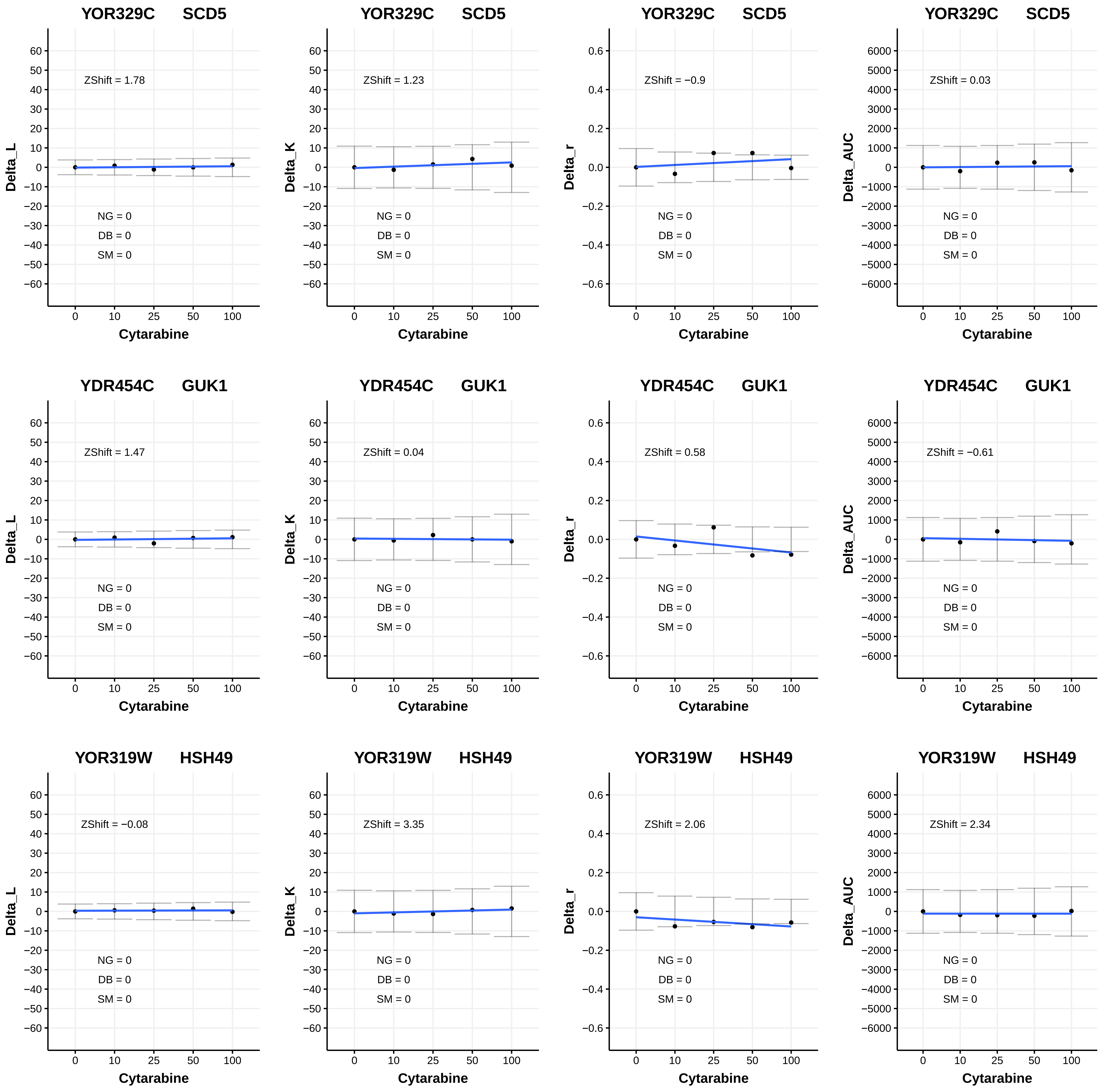
YOR329C SCD5
ZShift = 1.78
ZShift = 1.23
ZShift = −0.9
ZShift = 0.03
YDR454C GUK1
ZShift = 1.47
ZShift = 0.04
ZShift = 0.58
ZShift = −0.61
YOR319W HSH49
ZShift = −0.08
ZShift = 3.35
ZShift = 2.06
ZShift = 2.34
NG = 0
DB = 0
SM = 0
Delta_L
Delta_K
Delta_r
Delta_AUC
Cytarabine
0 10 25 50 100

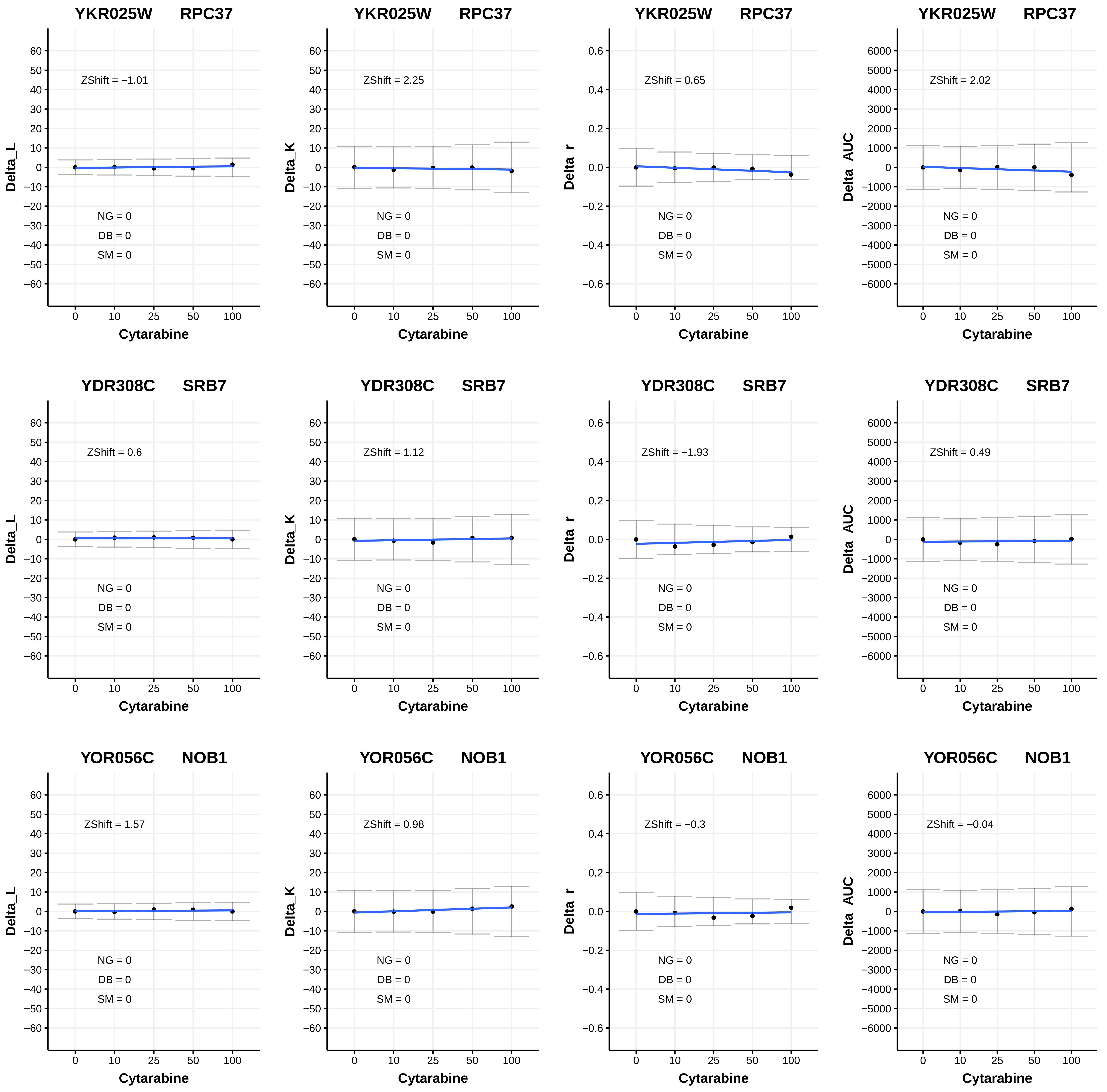
YKR025W RPC37
ZShift = −1.01
NG = 0
DB = 0
SM = 0
Delta_L
Cytarabine
YKR025W RPC37
ZShift = 2.25
NG = 0
DB = 0
SM = 0
Delta_K
Cytarabine
YKR025W RPC37
ZShift = 0.65
NG = 0
DB = 0
SM = 0
Delta_r
Cytarabine
YKR025W RPC37
ZShift = 2.02
NG = 0
DB = 0
SM = 0
Delta_AUC
Cytarabine
YDR308C SRB7
ZShift = 0.6
NG = 0
DB = 0
SM = 0
Delta_L
Cytarabine
YDR308C SRB7
ZShift = 1.12
NG = 0
DB = 0
SM = 0
Delta_K
Cytarabine
YDR308C SRB7
ZShift = −1.93
NG = 0
DB = 0
SM = 0
Delta_r
Cytarabine
YDR308C SRB7
ZShift = 0.49
NG = 0
DB = 0
SM = 0
Delta_AUC
Cytarabine
YOR056C NOB1
ZShift = 1.57
NG = 0
DB = 0
SM = 0
Delta_L
Cytarabine
YOR056C NOB1
ZShift = 0.98
NG = 0
DB = 0
SM = 0
Delta_K
Cytarabine
YOR056C NOB1
ZShift = −0.3
NG = 0
DB = 0
SM = 0
Delta_r
Cytarabine
YOR056C NOB1
ZShift = −0.04
NG = 0
DB = 0
SM = 0
Delta_AUC
Cytarabine

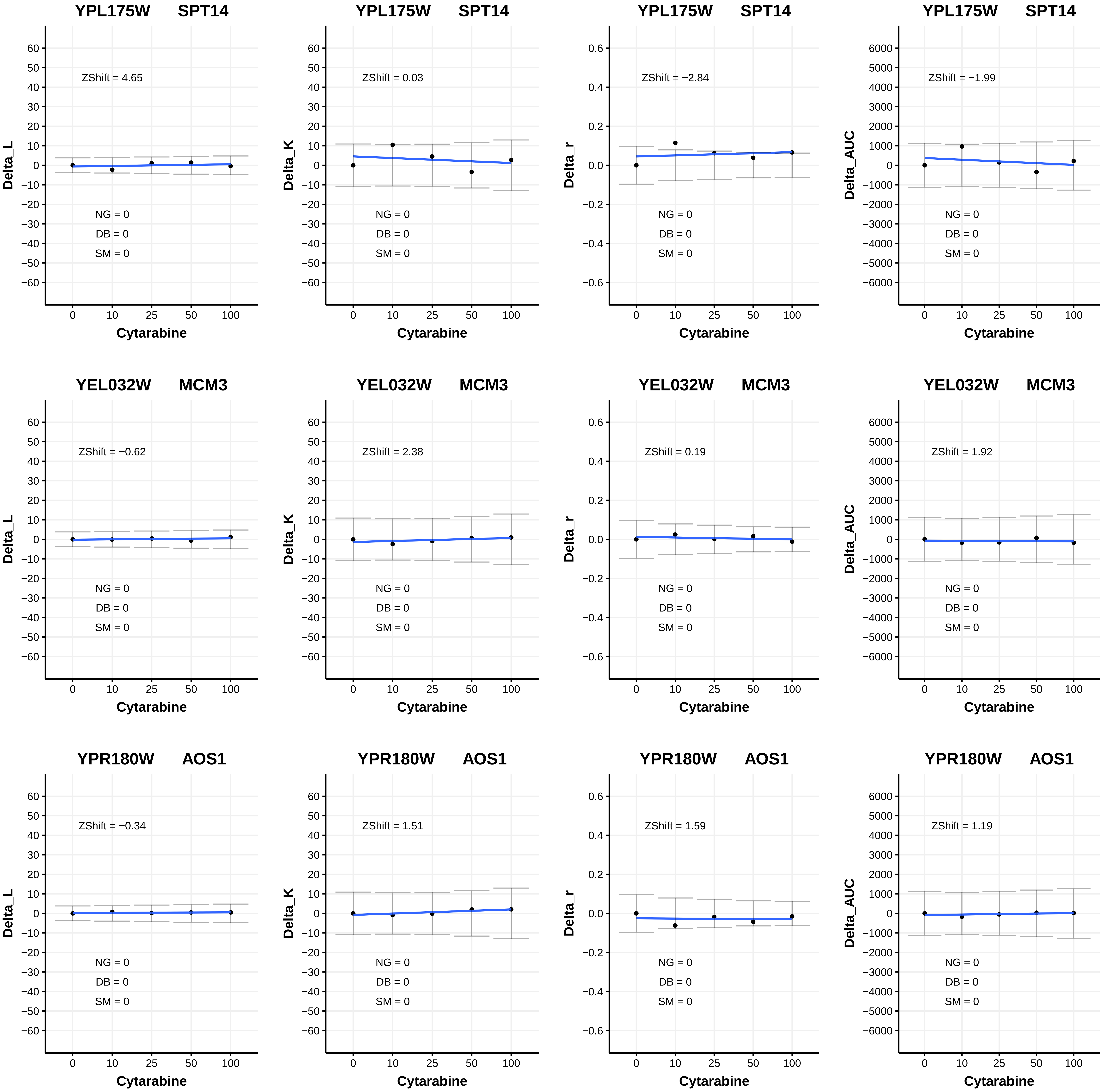

YPL175W SPT14
Delta_L
ZShift = 4.65
NG = 0
DB = 0
SM = 0
Cytarabine
YPL175W SPT14
Delta_K
ZShift = 0.03
NG = 0
DB = 0
SM = 0
Cytarabine
YPL175W SPT14
Delta_r
ZShift = −2.84
NG = 0
DB = 0
SM = 0
Cytarabine
YPL175W SPT14
Delta_AUC
ZShift = −1.99
NG = 0
DB = 0
SM = 0
Cytarabine
YEL032W MCM3
Delta_L
ZShift = −0.62
NG = 0
DB = 0
SM = 0
Cytarabine
YEL032W MCM3
Delta_K
ZShift = 2.38
NG = 0
DB = 0
SM = 0
Cytarabine
YEL032W MCM3
Delta_r
ZShift = 0.19
NG = 0
DB = 0
SM = 0
Cytarabine
YEL032W MCM3
Delta_AUC
ZShift = 1.92
NG = 0
DB = 0
SM = 0
Cytarabine
YPR180W AOS1
Delta_L
ZShift = −0.34
NG = 0
DB = 0
SM = 0
Cytarabine
YPR180W AOS1
Delta_K
ZShift = 1.51
NG = 0
DB = 0
SM = 0
Cytarabine
YPR180W AOS1
Delta_r
ZShift = 1.59
NG = 0
DB = 0
SM = 0
Cytarabine
YPR180W AOS1
Delta_AUC
ZShift = 1.19
NG = 0
DB = 0
SM = 0
Cytarabine

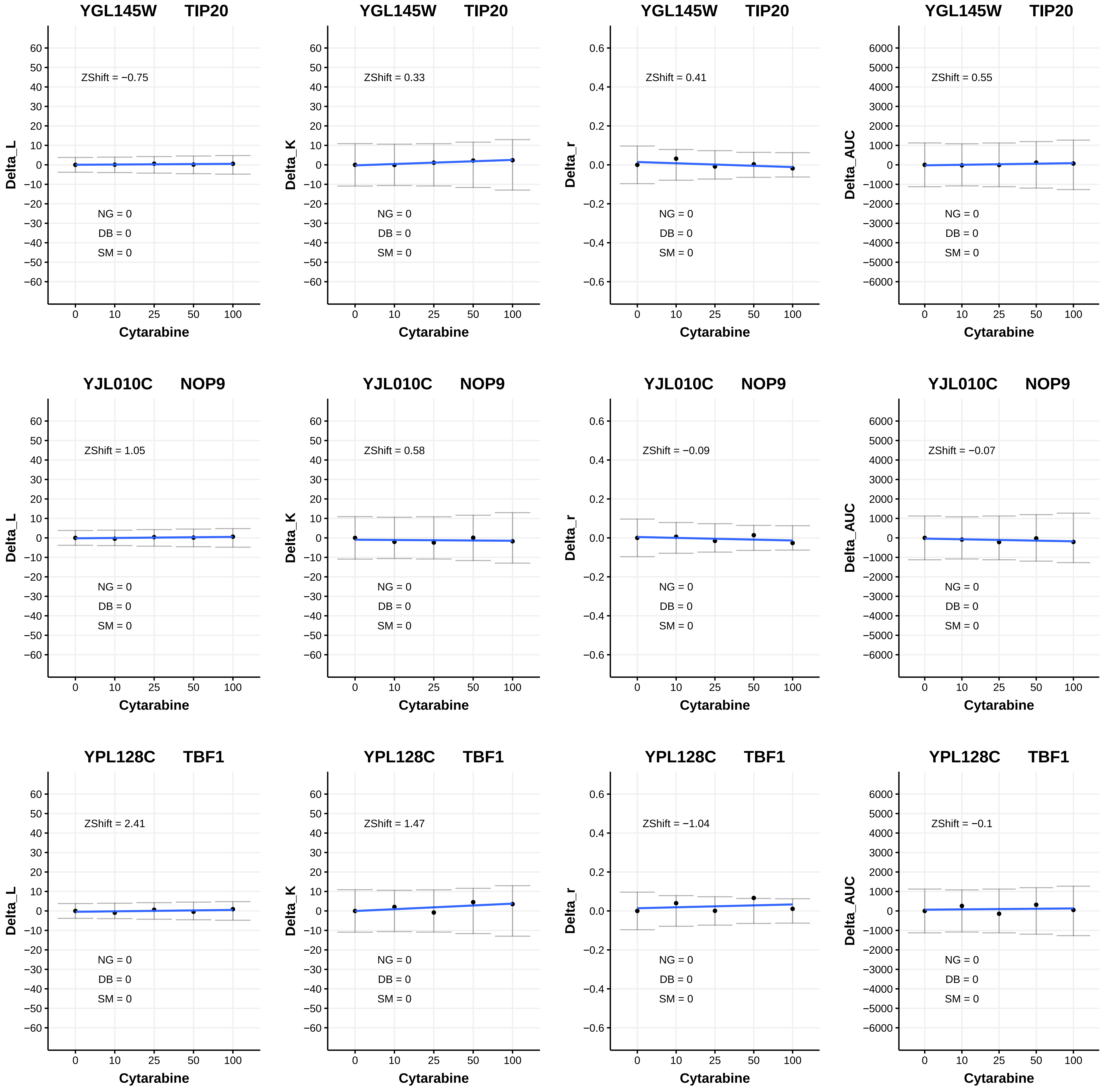
YGL145W TIP20
ZShift = −0.75
ZShift = 0.33
ZShift = 0.41
ZShift = 0.55
YJL010C NOP9
ZShift = 1.05
ZShift = 0.58
ZShift = −0.09
ZShift = −0.07
YPL128C TBF1
ZShift = 2.41
ZShift = 1.47
ZShift = −1.04
ZShift = −0.1
NG = 0
DB = 0
SM = 0
Delta_L
Delta_K
Delta_r
Delta_AUC
Cytarabine
0
10
25
50
100

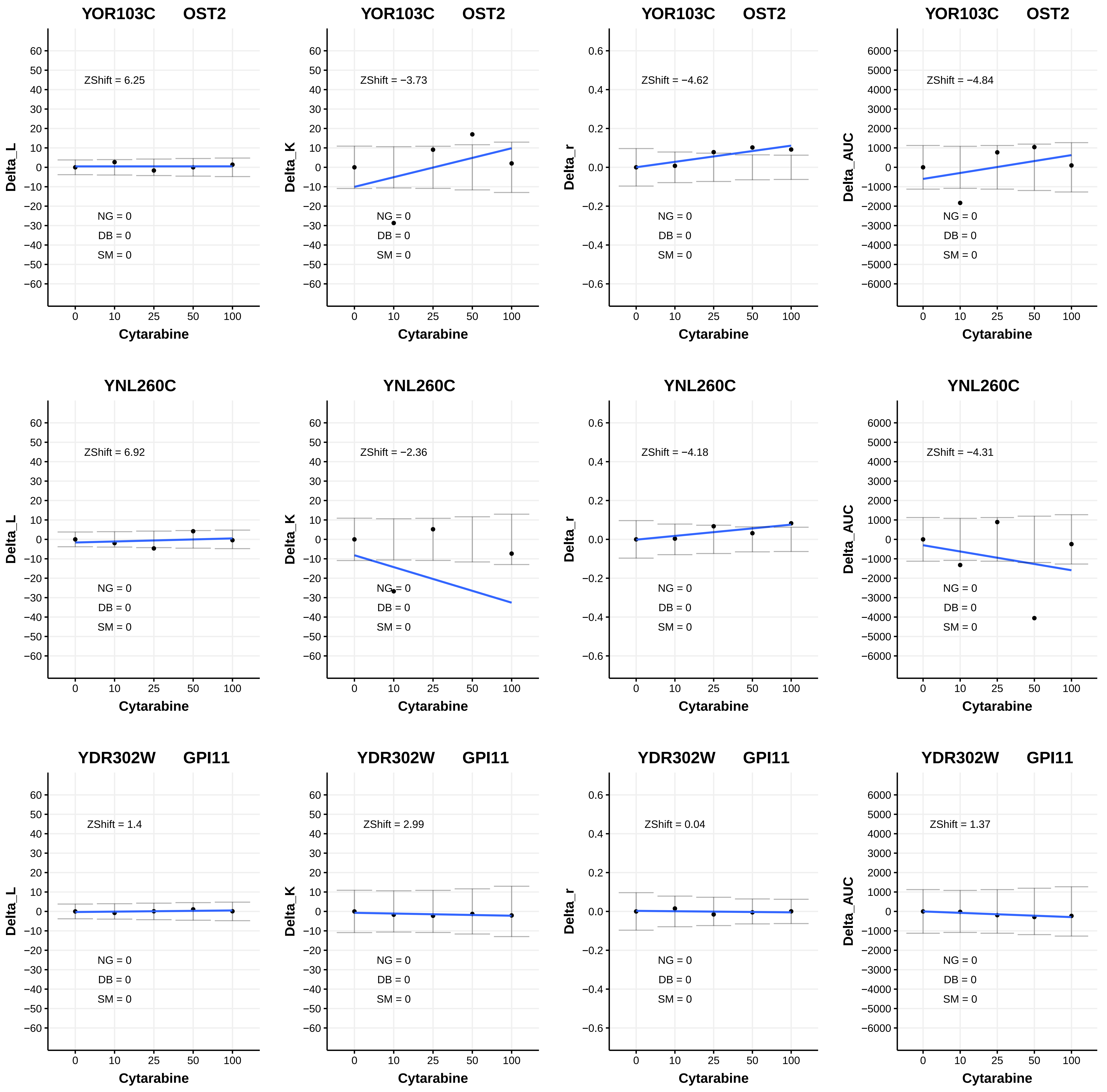
YOR103C OST2
ZShift = 6.25
NG = 0
DB = 0
SM = 0
Delta_L
Cytarabine
YOR103C OST2
ZShift = −3.73
NG = 0
DB = 0
SM = 0
Delta_K
Cytarabine
YOR103C OST2
ZShift = −4.62
NG = 0
DB = 0
SM = 0
Delta_r
Cytarabine
YOR103C OST2
ZShift = −4.84
NG = 0
DB = 0
SM = 0
Delta_AUC
Cytarabine
YNL260C
ZShift = 6.92
NG = 0
DB = 0
SM = 0
Delta_L
Cytarabine
YNL260C
ZShift = −2.36
NG = 0
DB = 0
SM = 0
Delta_K
Cytarabine
YNL260C
ZShift = −4.18
NG = 0
DB = 0
SM = 0
Delta_r
Cytarabine
YNL260C
ZShift = −4.31
NG = 0
DB = 0
SM = 0
Delta_AUC
Cytarabine
YDR302W GPI11
ZShift = 1.4
NG = 0
DB = 0
SM = 0
Delta_L
Cytarabine
YDR302W GPI11
ZShift = 2.99
NG = 0
DB = 0
SM = 0
Delta_K
Cytarabine
YDR302W GPI11
ZShift = 0.04
NG = 0
DB = 0
SM = 0
Delta_r
Cytarabine
YDR302W GPI11
ZShift = 1.37
NG = 0
DB = 0
SM = 0
Delta_AUC
Cytarabine

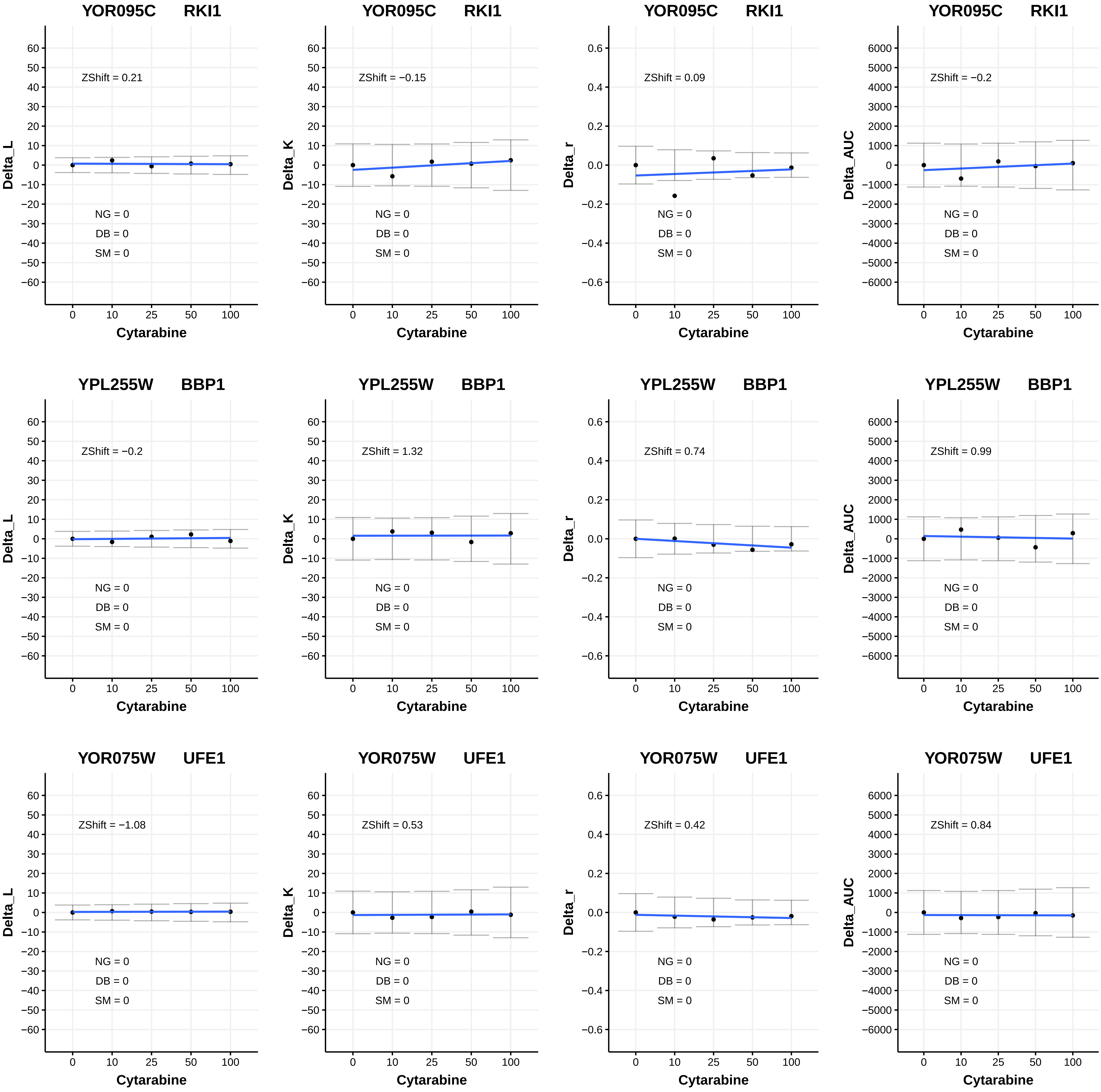

YOR095C RKI1
ZShift = 0.21
NG = 0
DB = 0
SM = 0
Delta_L
Cytarabine
YOR095C RKI1
ZShift = −0.15
NG = 0
DB = 0
SM = 0
Delta_K
Cytarabine
YOR095C RKI1
ZShift = 0.09
NG = 0
DB = 0
SM = 0
Delta_r
Cytarabine
YOR095C RKI1
ZShift = −0.2
NG = 0
DB = 0
SM = 0
Delta_AUC
Cytarabine
YPL255W BBP1
ZShift = −0.2
NG = 0
DB = 0
SM = 0
Delta_L
Cytarabine
YPL255W BBP1
ZShift = 1.32
NG = 0
DB = 0
SM = 0
Delta_K
Cytarabine
YPL255W BBP1
ZShift = 0.74
NG = 0
DB = 0
SM = 0
Delta_r
Cytarabine
YPL255W BBP1
ZShift = 0.99
NG = 0
DB = 0
SM = 0
Delta_AUC
Cytarabine
YOR075W UFE1
ZShift = −1.08
NG = 0
DB = 0
SM = 0
Delta_L
Cytarabine
YOR075W UFE1
ZShift = 0.53
NG = 0
DB = 0
SM = 0
Delta_K
Cytarabine
YOR075W UFE1
ZShift = 0.42
NG = 0
DB = 0
SM = 0
Delta_r
Cytarabine
YOR075W UFE1
ZShift = 0.84
NG = 0
DB = 0
SM = 0
Delta_AUC
Cytarabine

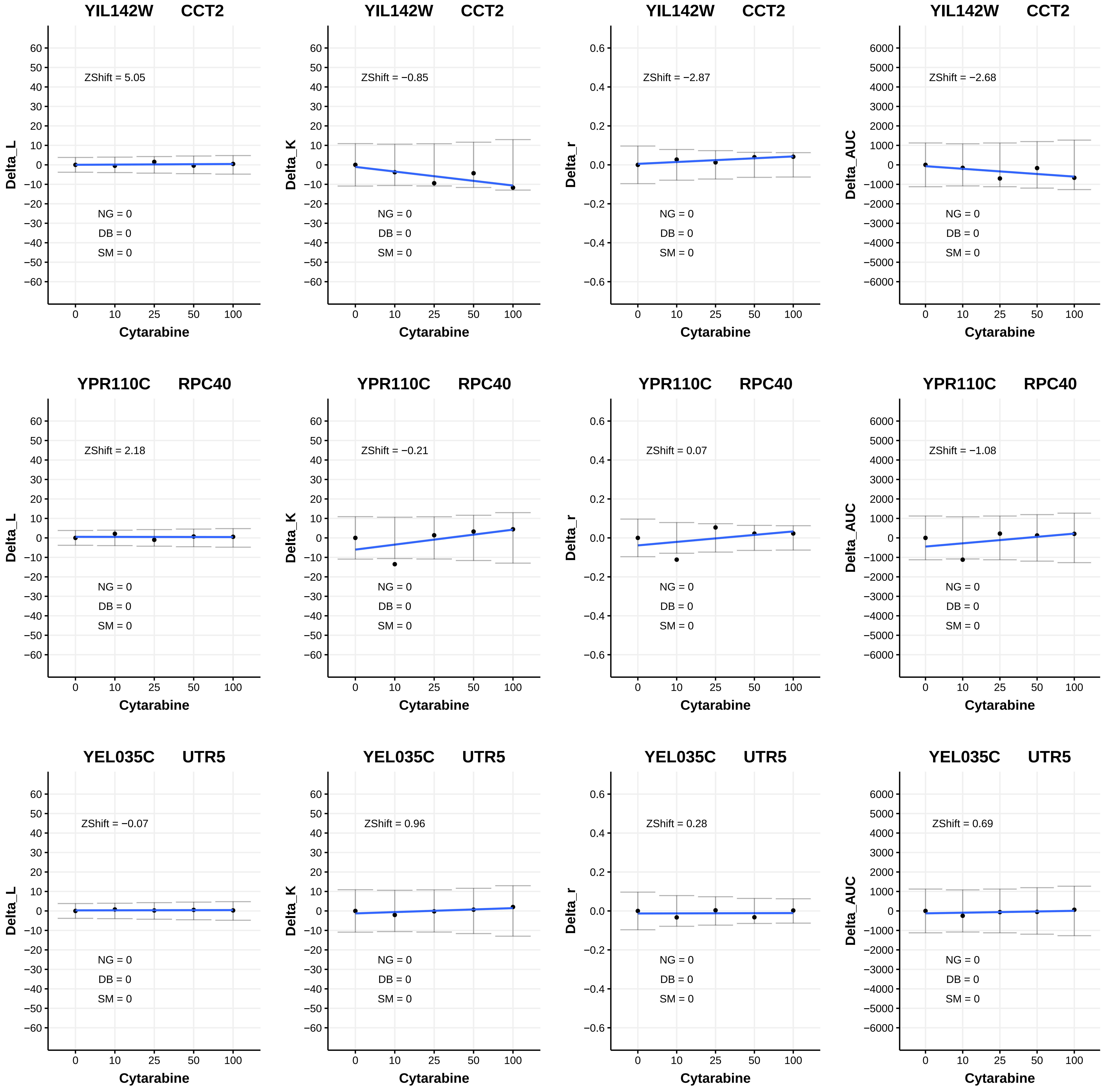
YIL142W CCT2
Delta_L
ZShift = 5.05
NG = 0
DB = 0
SM = 0
Cytarabine
YIL142W CCT2
Delta_K
ZShift = −0.85
NG = 0
DB = 0
SM = 0
Cytarabine
YIL142W CCT2
Delta_r
ZShift = −2.87
NG = 0
DB = 0
SM = 0
Cytarabine
YIL142W CCT2
Delta_AUC
ZShift = −2.68
NG = 0
DB = 0
SM = 0
Cytarabine
YPR110C RPC40
Delta_L
ZShift = 2.18
NG = 0
DB = 0
SM = 0
Cytarabine
YPR110C RPC40
Delta_K
ZShift = −0.21
NG = 0
DB = 0
SM = 0
Cytarabine
YPR110C RPC40
Delta_r
ZShift = 0.07
NG = 0
DB = 0
SM = 0
Cytarabine
YPR110C RPC40
Delta_AUC
ZShift = −1.08
NG = 0
DB = 0
SM = 0
Cytarabine
YEL035C UTR5
Delta_L
ZShift = −0.07
NG = 0
DB = 0
SM = 0
Cytarabine
YEL035C UTR5
Delta_K
ZShift = 0.96
NG = 0
DB = 0
SM = 0
Cytarabine
YEL035C UTR5
Delta_r
ZShift = 0.28
NG = 0
DB = 0
SM = 0
Cytarabine
YEL035C UTR5
Delta_AUC
ZShift = 0.69
NG = 0
DB = 0
SM = 0
Cytarabine

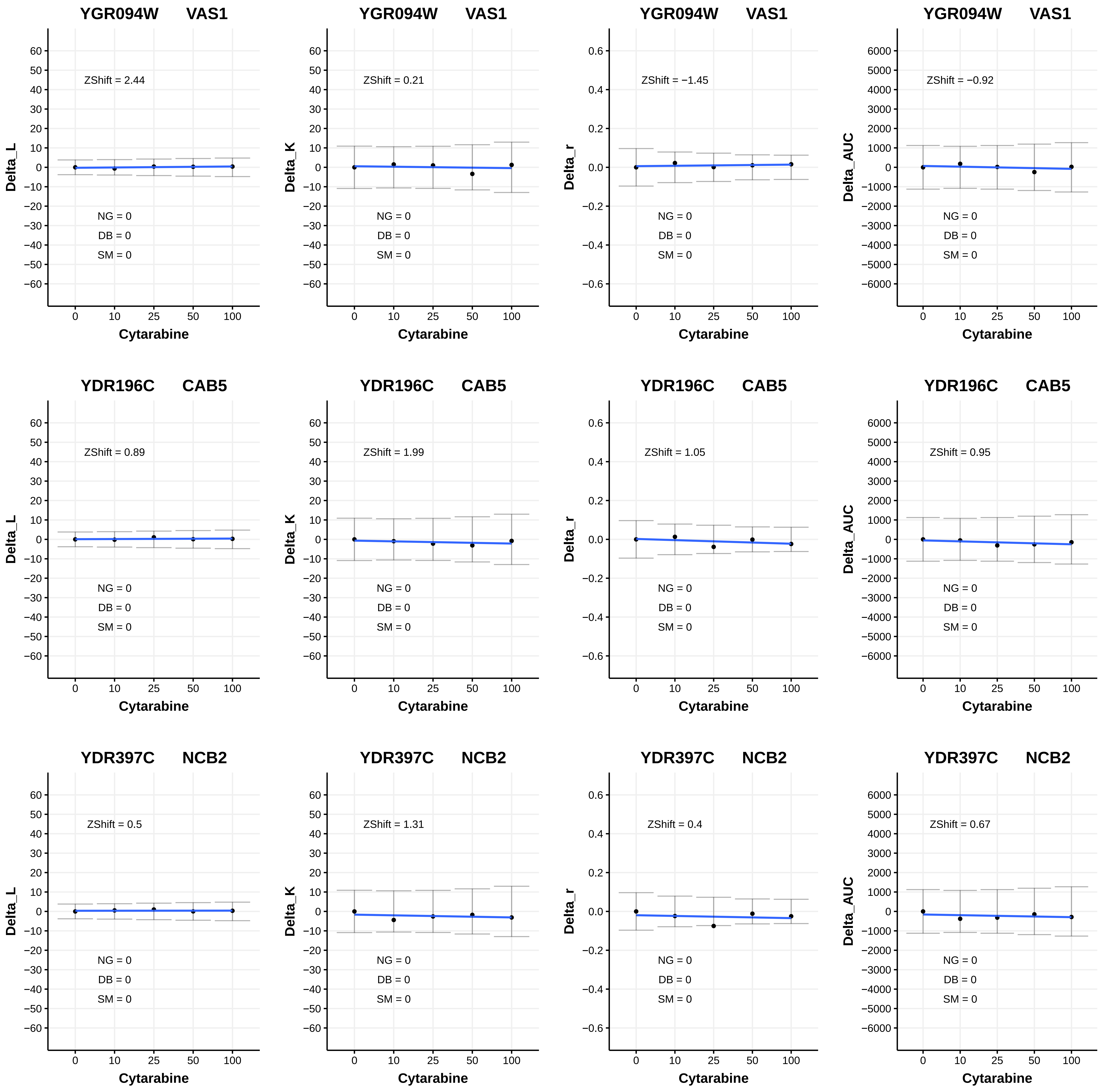
YGR094W VAS1
ZShift = 2.44
Delta_L
NG = 0
DB = 0
SM = 0
Cytarabine
YGR094W VAS1
ZShift = 0.21
Delta_K
NG = 0
DB = 0
SM = 0
Cytarabine
YGR094W VAS1
ZShift = −1.45
Delta_r
NG = 0
DB = 0
SM = 0
Cytarabine
YGR094W VAS1
ZShift = −0.92
Delta_AUC
NG = 0
DB = 0
SM = 0
Cytarabine
YDR196C CAB5
ZShift = 0.89
Delta_L
NG = 0
DB = 0
SM = 0
Cytarabine
YDR196C CAB5
ZShift = 1.99
Delta_K
NG = 0
DB = 0
SM = 0
Cytarabine
YDR196C CAB5
ZShift = 1.05
Delta_r
NG = 0
DB = 0
SM = 0
Cytarabine
YDR196C CAB5
ZShift = 0.95
Delta_AUC
NG = 0
DB = 0
SM = 0
Cytarabine
YDR397C NCB2
ZShift = 0.5
Delta_L
NG = 0
DB = 0
SM = 0
Cytarabine
YDR397C NCB2
ZShift = 1.31
Delta_K
NG = 0
DB = 0
SM = 0
Cytarabine
YDR397C NCB2
ZShift = 0.4
Delta_r
NG = 0
DB = 0
SM = 0
Cytarabine
YDR397C NCB2
ZShift = 0.67
Delta_AUC
NG = 0
DB = 0
SM = 0
Cytarabine

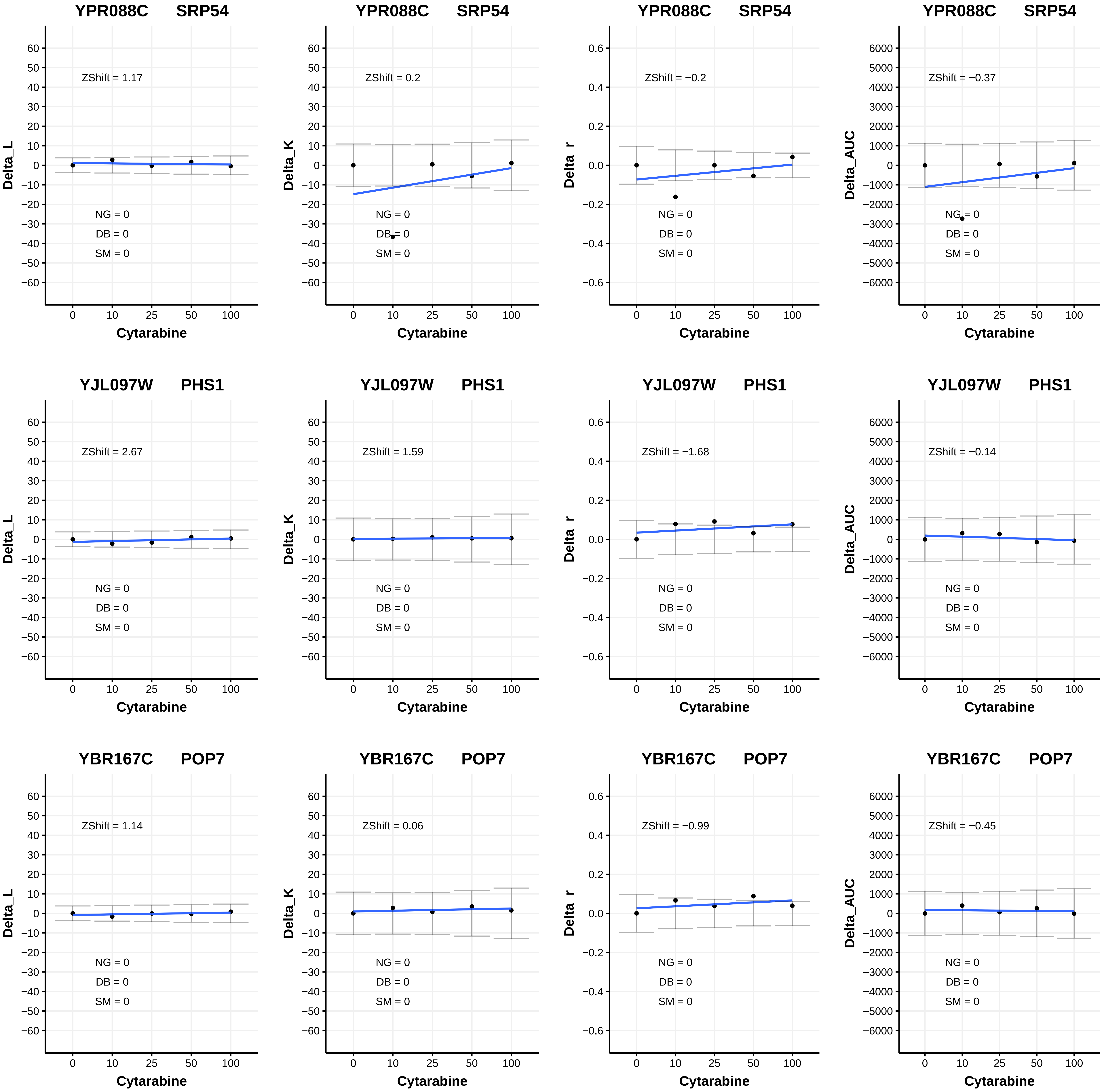
YPR088C SRP54
ZShift = 1.17
NG = 0
DB = 0
SM = 0
Delta_L
Cytarabine
YPR088C SRP54
ZShift = 0.2
NG = 0
DB = 0
SM = 0
Delta_K
Cytarabine
YPR088C SRP54
ZShift = −0.2
NG = 0
DB = 0
SM = 0
Delta_r
Cytarabine
YPR088C SRP54
ZShift = −0.37
NG = 0
DB = 0
SM = 0
Delta_AUC
Cytarabine
YJL097W PHS1
ZShift = 2.67
NG = 0
DB = 0
SM = 0
Delta_L
Cytarabine
YJL097W PHS1
ZShift = 1.59
NG = 0
DB = 0
SM = 0
Delta_K
Cytarabine
YJL097W PHS1
ZShift = −1.68
NG = 0
DB = 0
SM = 0
Delta_r
Cytarabine
YJL097W PHS1
ZShift = −0.14
NG = 0
DB = 0
SM = 0
Delta_AUC
Cytarabine
YBR167C POP7
ZShift = 1.14
NG = 0
DB = 0
SM = 0
Delta_L
Cytarabine
YBR167C POP7
ZShift = 0.06
NG = 0
DB = 0
SM = 0
Delta_K
Cytarabine
YBR167C POP7
ZShift = −0.99
NG = 0
DB = 0
SM = 0
Delta_r
Cytarabine
YBR167C POP7
ZShift = −0.45
NG = 0
DB = 0
SM = 0
Delta_AUC
Cytarabine

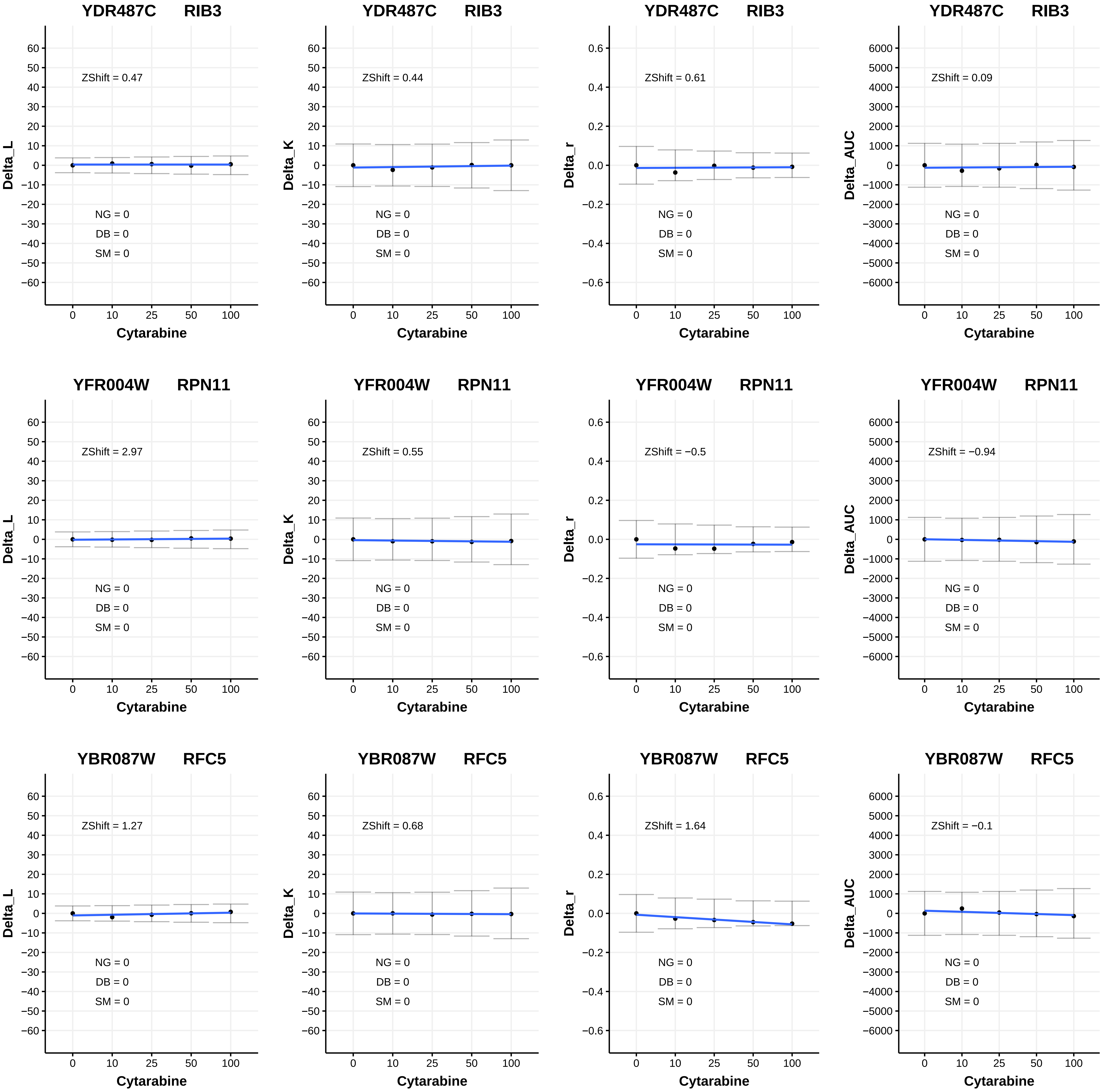

YDR487C RIB3
ZShift = 0.47
NG = 0
DB = 0
SM = 0
Delta_L
Cytarabine
YDR487C RIB3
ZShift = 0.44
NG = 0
DB = 0
SM = 0
Delta_K
Cytarabine
YDR487C RIB3
ZShift = 0.61
NG = 0
DB = 0
SM = 0
Delta_r
Cytarabine
YDR487C RIB3
ZShift = 0.09
NG = 0
DB = 0
SM = 0
Delta_AUC
Cytarabine
YFR004W RPN11
ZShift = 2.97
NG = 0
DB = 0
SM = 0
Delta_L
Cytarabine
YFR004W RPN11
ZShift = 0.55
NG = 0
DB = 0
SM = 0
Delta_K
Cytarabine
YFR004W RPN11
ZShift = −0.5
NG = 0
DB = 0
SM = 0
Delta_r
Cytarabine
YFR004W RPN11
ZShift = −0.94
NG = 0
DB = 0
SM = 0
Delta_AUC
Cytarabine
YBR087W RFC5
ZShift = 1.27
NG = 0
DB = 0
SM = 0
Delta_L
Cytarabine
YBR087W RFC5
ZShift = 0.68
NG = 0
DB = 0
SM = 0
Delta_K
Cytarabine
YBR087W RFC5
ZShift = 1.64
NG = 0
DB = 0
SM = 0
Delta_r
Cytarabine
YBR087W RFC5
ZShift = −0.1
NG = 0
DB = 0
SM = 0
Delta_AUC
Cytarabine

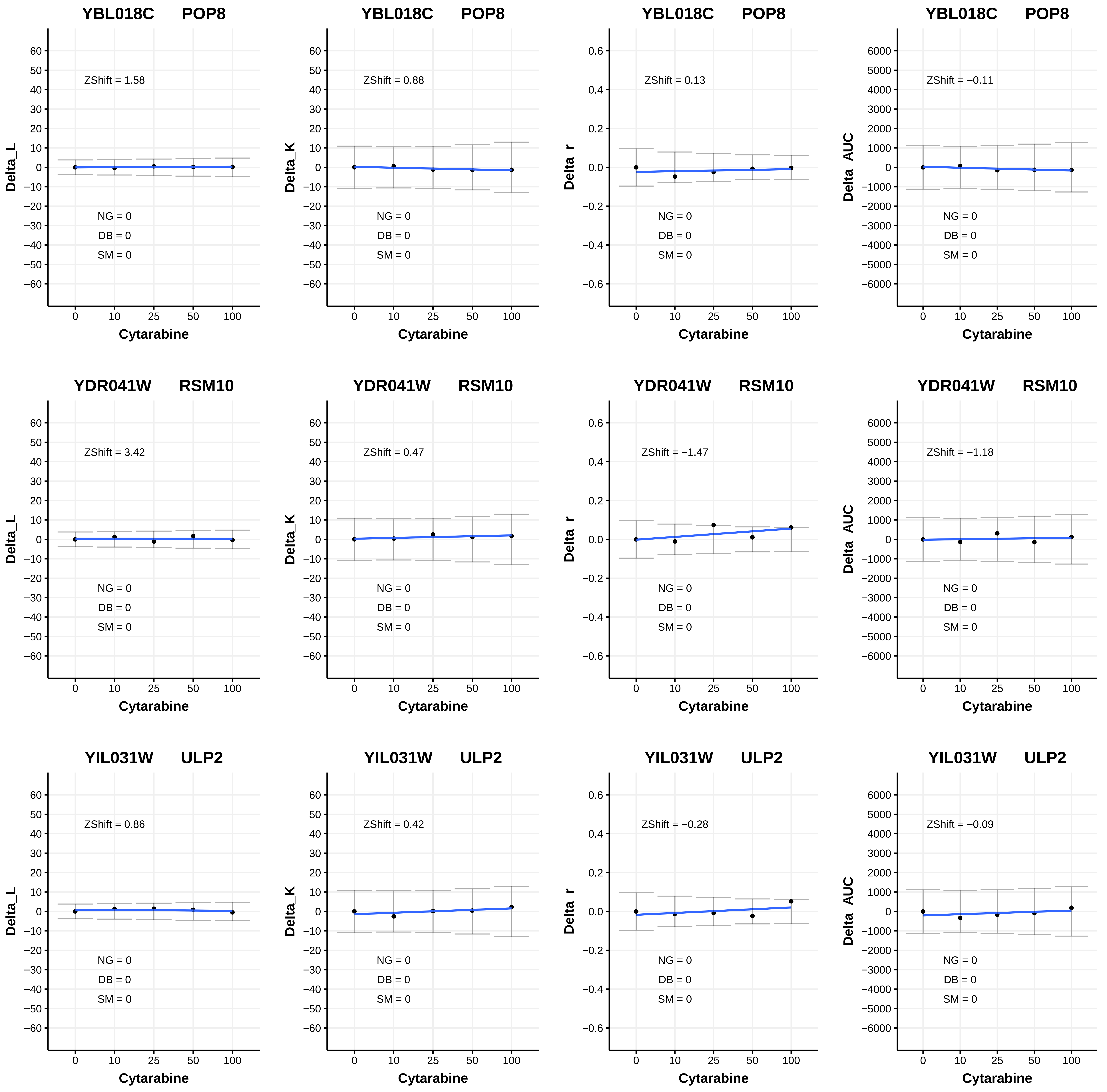
YBL018C POP8
ZShift = 1.58
NG = 0
DB = 0
SM = 0
Delta_L
Cytarabine
YBL018C POP8
ZShift = 0.88
NG = 0
DB = 0
SM = 0
Delta_K
Cytarabine
YBL018C POP8
ZShift = 0.13
NG = 0
DB = 0
SM = 0
Delta_r
Cytarabine
YBL018C POP8
ZShift = −0.11
NG = 0
DB = 0
SM = 0
Delta_AUC
Cytarabine
YDR041W RSM10
ZShift = 3.42
NG = 0
DB = 0
SM = 0
Delta_L
Cytarabine
YDR041W RSM10
ZShift = 0.47
NG = 0
DB = 0
SM = 0
Delta_K
Cytarabine
YDR041W RSM10
ZShift = −1.47
NG = 0
DB = 0
SM = 0
Delta_r
Cytarabine
YDR041W RSM10
ZShift = −1.18
NG = 0
DB = 0
SM = 0
Delta_AUC
Cytarabine
YIL031W ULP2
ZShift = 0.86
NG = 0
DB = 0
SM = 0
Delta_L
Cytarabine
YIL031W ULP2
ZShift = 0.42
NG = 0
DB = 0
SM = 0
Delta_K
Cytarabine
YIL031W ULP2
ZShift = −0.28
NG = 0
DB = 0
SM = 0
Delta_r
Cytarabine
YIL031W ULP2
ZShift = −0.09
NG = 0
DB = 0
SM = 0
Delta_AUC
Cytarabine

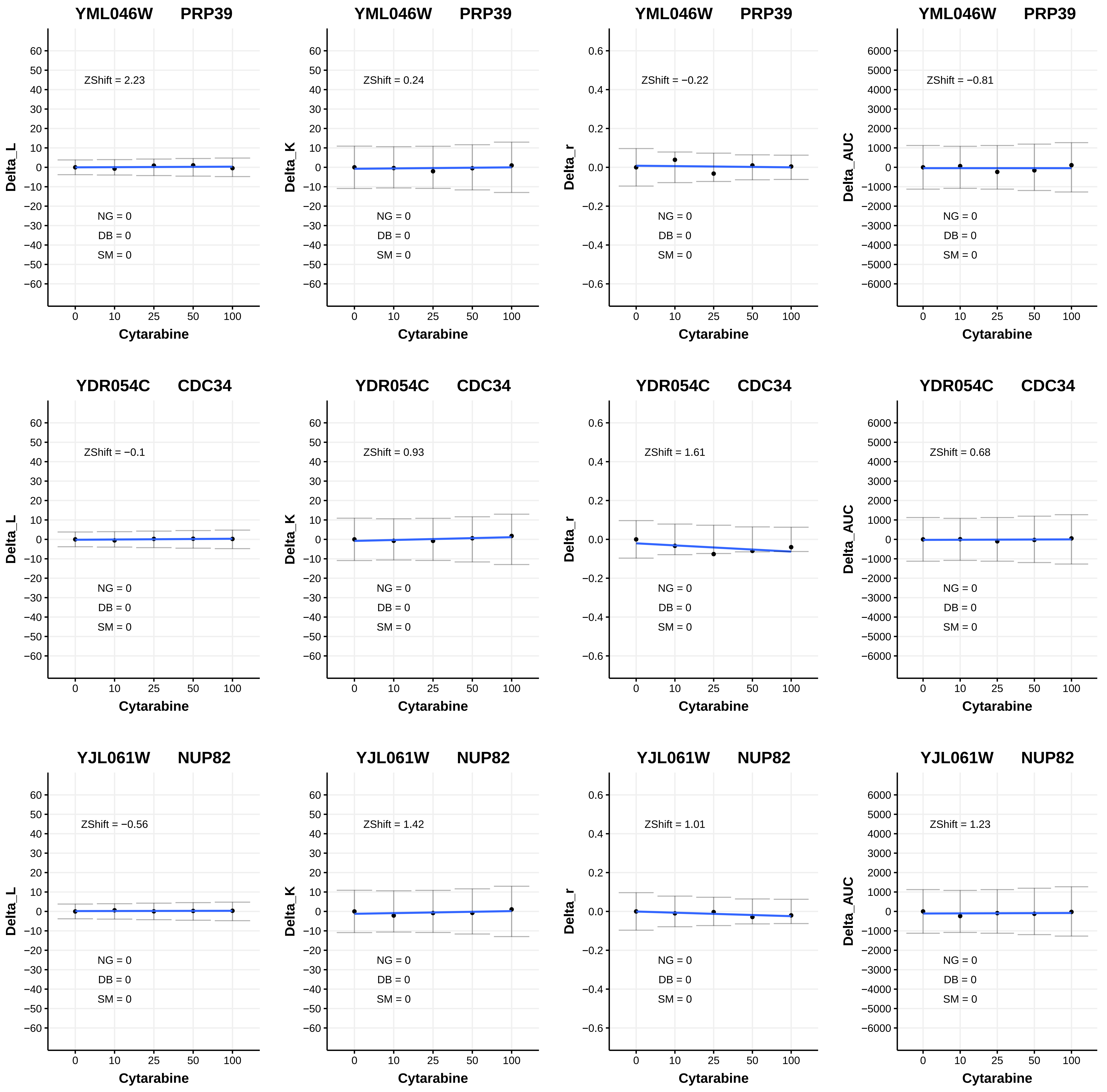
YML046W PRP39
ZShift = 2.23
NG = 0
DB = 0
SM = 0
Delta_L
Cytarabine
YML046W PRP39
ZShift = 0.24
NG = 0
DB = 0
SM = 0
Delta_K
Cytarabine
YML046W PRP39
ZShift = −0.22
NG = 0
DB = 0
SM = 0
Delta_r
Cytarabine
YML046W PRP39
ZShift = −0.81
NG = 0
DB = 0
SM = 0
Delta_AUC
Cytarabine
YDR054C CDC34
ZShift = −0.1
NG = 0
DB = 0
SM = 0
Delta_L
Cytarabine
YDR054C CDC34
ZShift = 0.93
NG = 0
DB = 0
SM = 0
Delta_K
Cytarabine
YDR054C CDC34
ZShift = 1.61
NG = 0
DB = 0
SM = 0
Delta_r
Cytarabine
YDR054C CDC34
ZShift = 0.68
NG = 0
DB = 0
SM = 0
Delta_AUC
Cytarabine
YJL061W NUP82
ZShift = −0.56
NG = 0
DB = 0
SM = 0
Delta_L
Cytarabine
YJL061W NUP82
ZShift = 1.42
NG = 0
DB = 0
SM = 0
Delta_K
Cytarabine
YJL061W NUP82
ZShift = 1.01
NG = 0
DB = 0
SM = 0
Delta_r
Cytarabine
YJL061W NUP82
ZShift = 1.23
NG = 0
DB = 0
SM = 0
Delta_AUC
Cytarabine

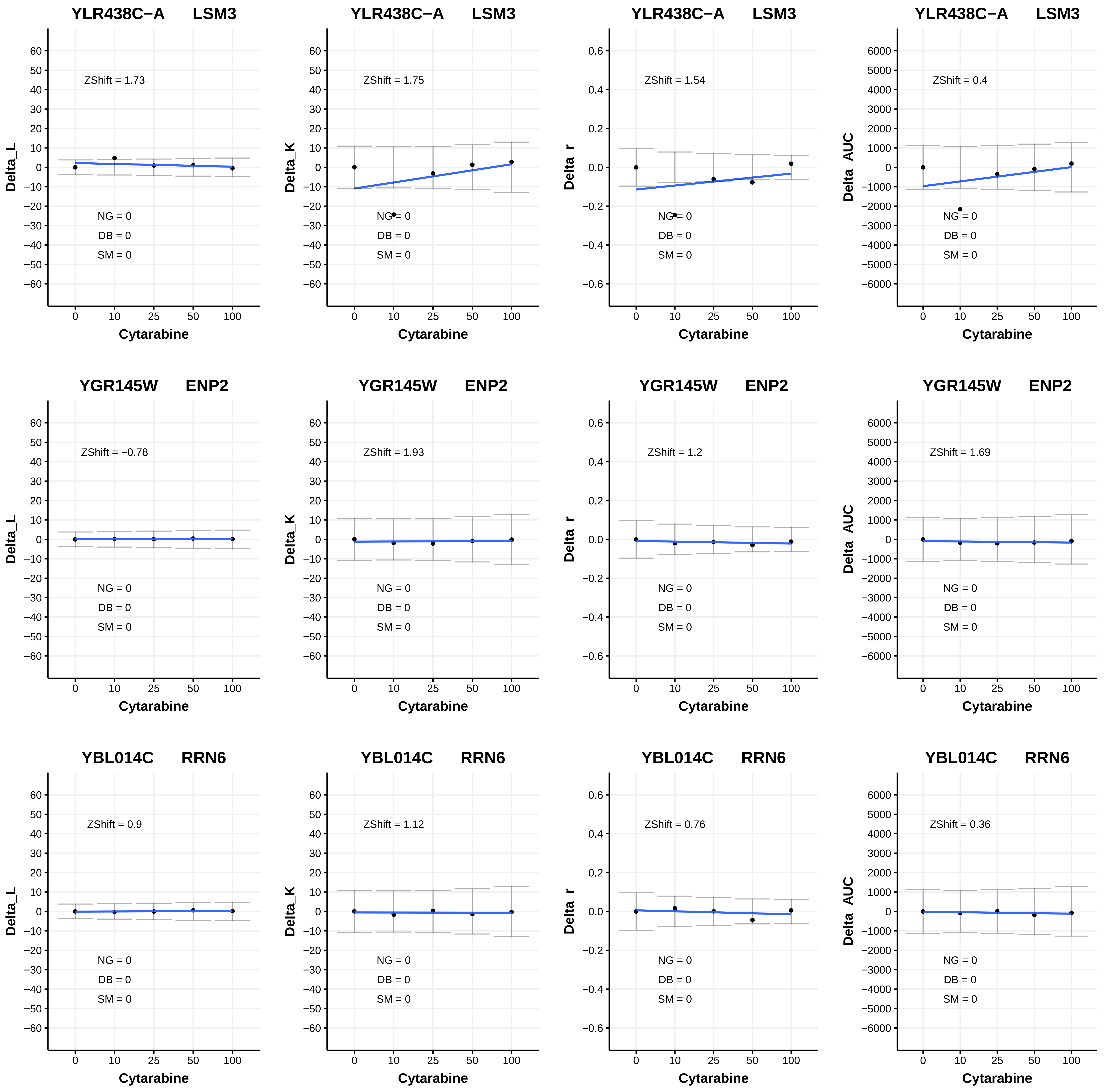
YLR438C−A LSM3
ZShift = 1.73
ZShift = 1.75
ZShift = 1.54
ZShift = 0.4
NG = 0
DB = 0
SM = 0
Delta_L
Delta_K
Delta_r
Delta_AUC
Cytarabine
YGR145W ENP2
ZShift = −0.78
ZShift = 1.93
ZShift = 1.2
ZShift = 1.69
YBL014C RRN6
ZShift = 0.9
ZShift = 1.12
ZShift = 0.76
ZShift = 0.36

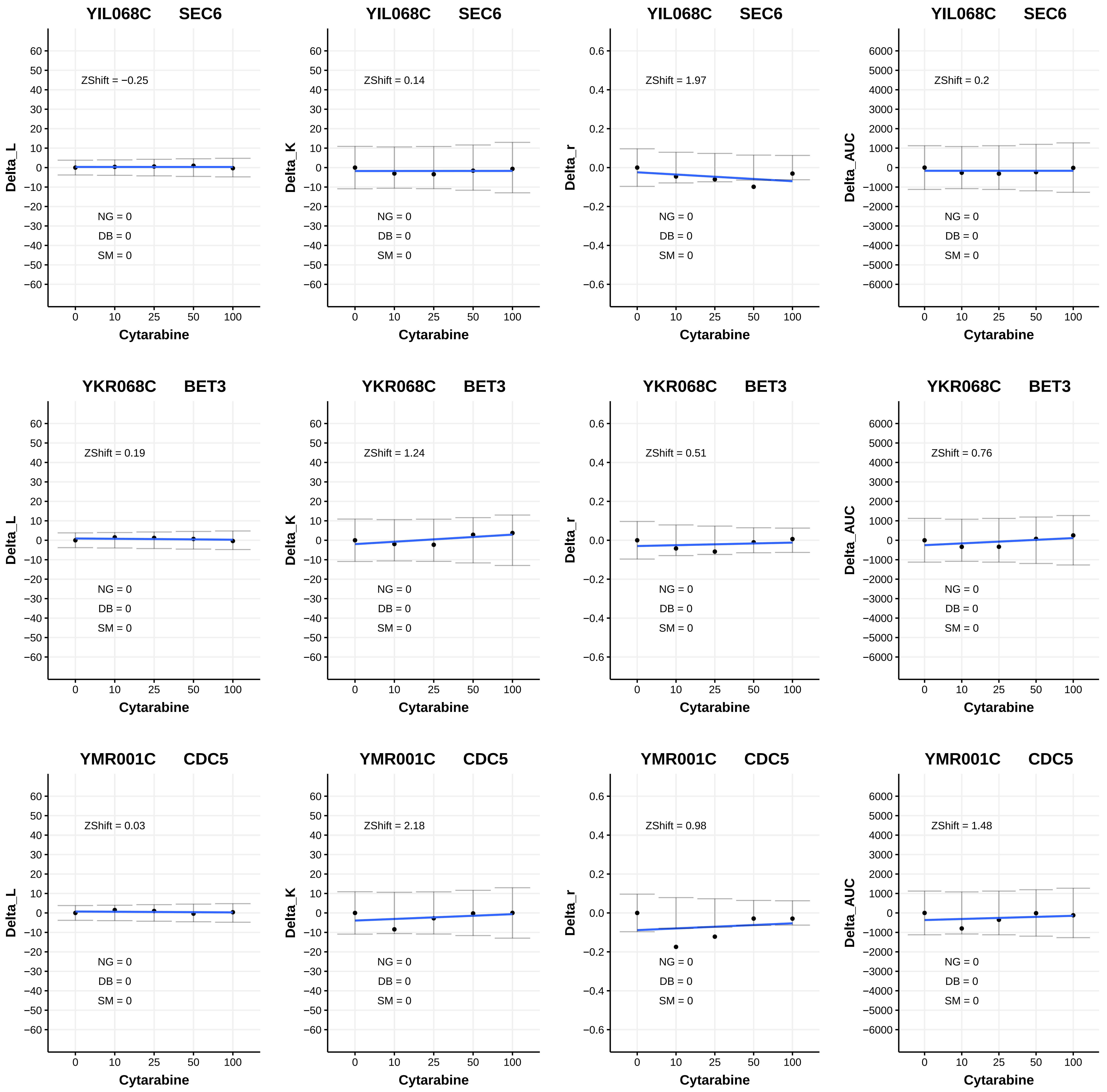
YIL068C SEC6
ZShift = −0.25
NG = 0
DB = 0
SM = 0
Delta_L
Cytarabine
YIL068C SEC6
ZShift = 0.14
NG = 0
DB = 0
SM = 0
Delta_K
Cytarabine
YIL068C SEC6
ZShift = 1.97
NG = 0
DB = 0
SM = 0
Delta_r
Cytarabine
YIL068C SEC6
ZShift = 0.2
NG = 0
DB = 0
SM = 0
Delta_AUC
Cytarabine
YKR068C BET3
ZShift = 0.19
NG = 0
DB = 0
SM = 0
Delta_L
Cytarabine
YKR068C BET3
ZShift = 1.24
NG = 0
DB = 0
SM = 0
Delta_K
Cytarabine
YKR068C BET3
ZShift = 0.51
NG = 0
DB = 0
SM = 0
Delta_r
Cytarabine
YKR068C BET3
ZShift = 0.76
NG = 0
DB = 0
SM = 0
Delta_AUC
Cytarabine
YMR001C CDC5
ZShift = 0.03
NG = 0
DB = 0
SM = 0
Delta_L
Cytarabine
YMR001C CDC5
ZShift = 2.18
NG = 0
DB = 0
SM = 0
Delta_K
Cytarabine
YMR001C CDC5
ZShift = 0.98
NG = 0
DB = 0
SM = 0
Delta_r
Cytarabine
YMR001C CDC5
ZShift = 1.48
NG = 0
DB = 0
SM = 0
Delta_AUC
Cytarabine

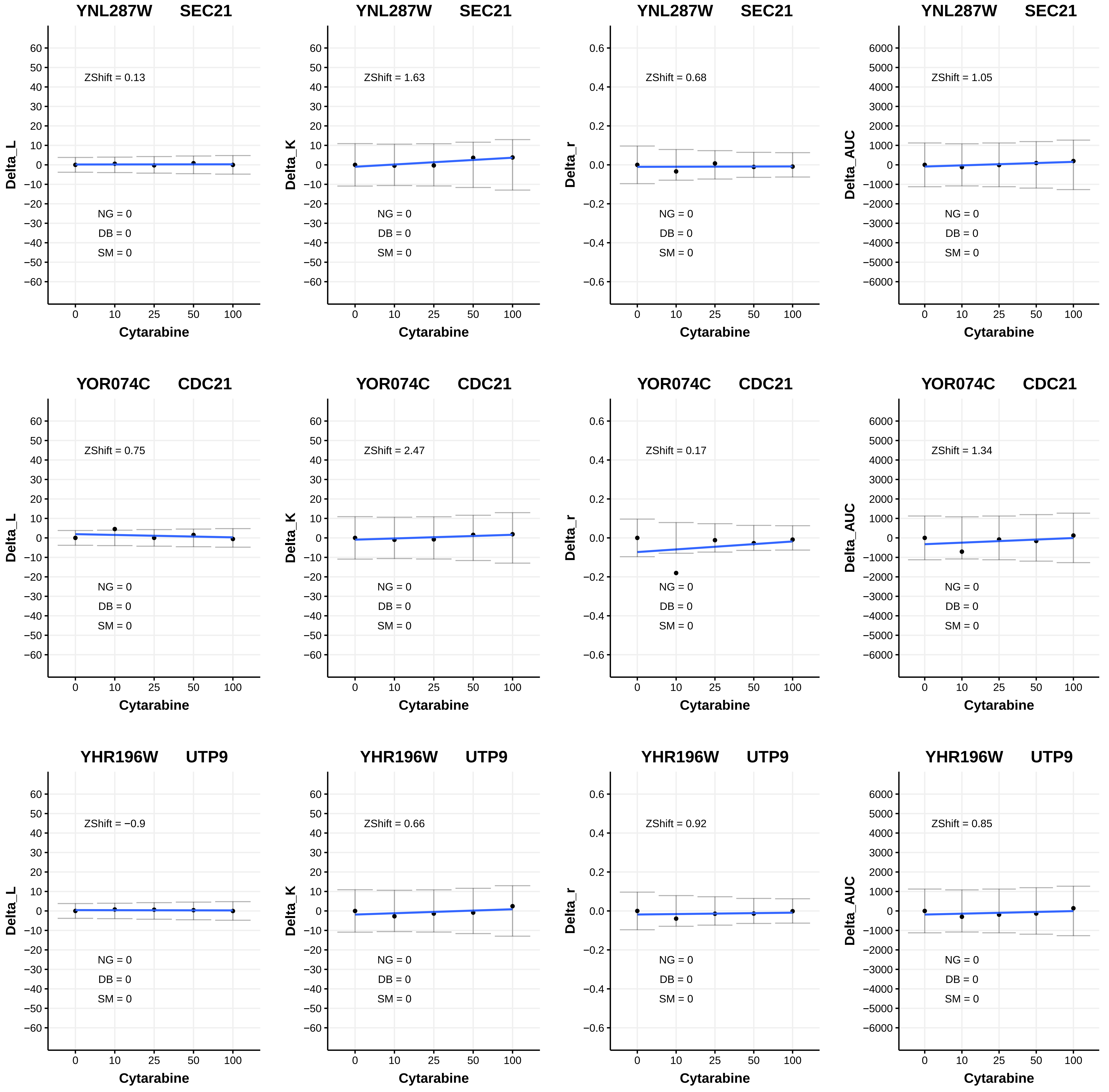

YNL287W SEC21
ZShift = 0.13
NG = 0
DB = 0
SM = 0
Delta_L
Cytarabine
YNL287W SEC21
ZShift = 1.63
Delta_K
YNL287W SEC21
ZShift = 0.68
Delta_r
YNL287W SEC21
ZShift = 1.05
Delta_AUC
YOR074C CDC21
ZShift = 0.75
YOR074C CDC21
ZShift = 2.47
YOR074C CDC21
ZShift = 0.17
YOR074C CDC21
ZShift = 1.34
YHR196W UTP9
ZShift = −0.9
YHR196W UTP9
ZShift = 0.66
YHR196W UTP9
ZShift = 0.92
YHR196W UTP9
ZShift = 0.85

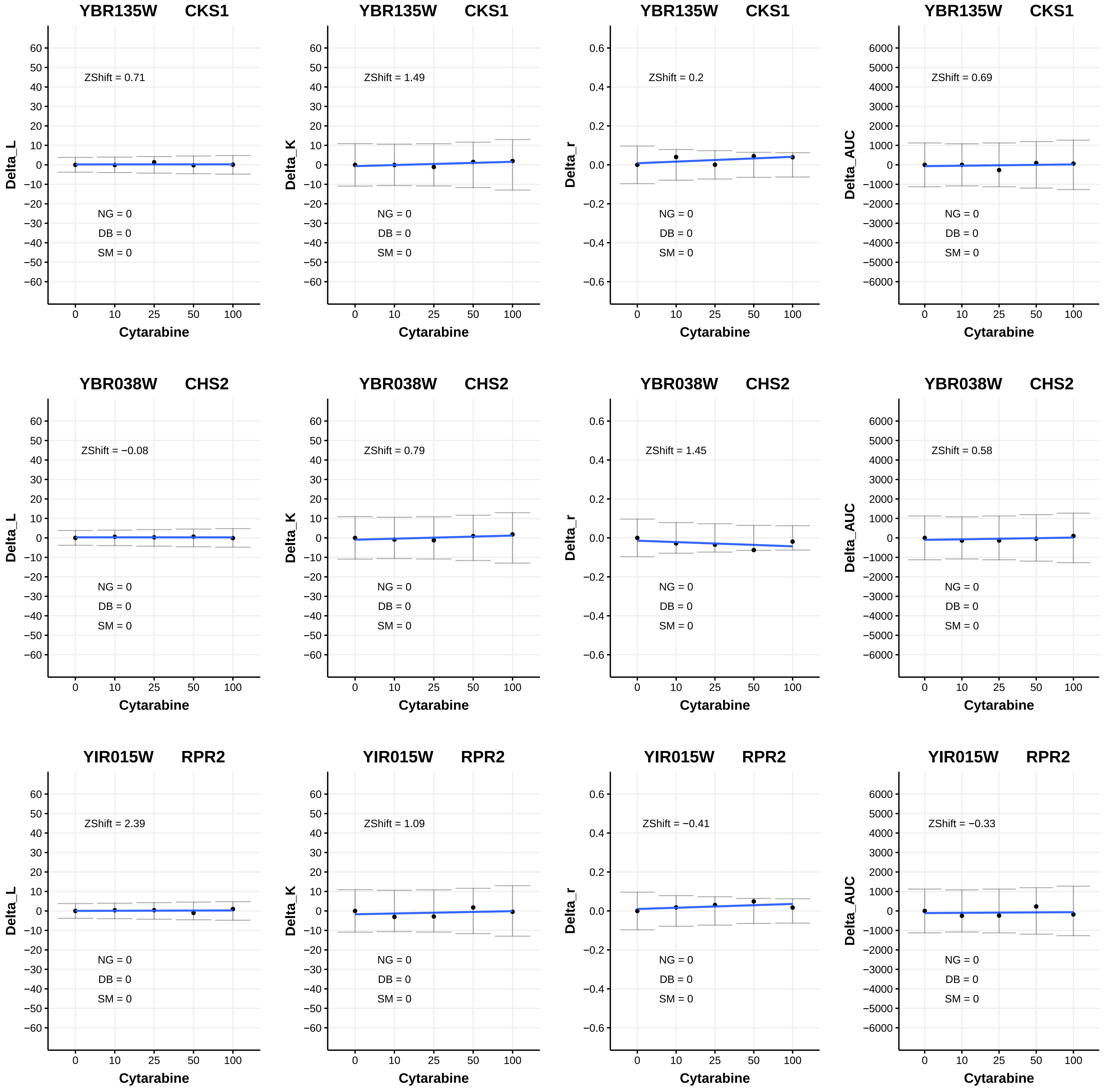
YBR135W CKS1
ZShift = 0.71
NG = 0
DB = 0
SM = 0
Delta_L
Cytarabine
YBR135W CKS1
ZShift = 1.49
NG = 0
DB = 0
SM = 0
Delta_K
Cytarabine
YBR135W CKS1
ZShift = 0.2
NG = 0
DB = 0
SM = 0
Delta_r
Cytarabine
YBR135W CKS1
ZShift = 0.69
NG = 0
DB = 0
SM = 0
Delta_AUC
Cytarabine
YBR038W CHS2
ZShift = −0.08
NG = 0
DB = 0
SM = 0
Delta_L
Cytarabine
YBR038W CHS2
ZShift = 0.79
NG = 0
DB = 0
SM = 0
Delta_K
Cytarabine
YBR038W CHS2
ZShift = 1.45
NG = 0
DB = 0
SM = 0
Delta_r
Cytarabine
YBR038W CHS2
ZShift = 0.58
NG = 0
DB = 0
SM = 0
Delta_AUC
Cytarabine
YIR015W RPR2
ZShift = 2.39
NG = 0
DB = 0
SM = 0
Delta_L
Cytarabine
YIR015W RPR2
ZShift = 1.09
NG = 0
DB = 0
SM = 0
Delta_K
Cytarabine
YIR015W RPR2
ZShift = −0.41
NG = 0
DB = 0
SM = 0
Delta_r
Cytarabine
YIR015W RPR2
ZShift = −0.33
NG = 0
DB = 0
SM = 0
Delta_AUC
Cytarabine

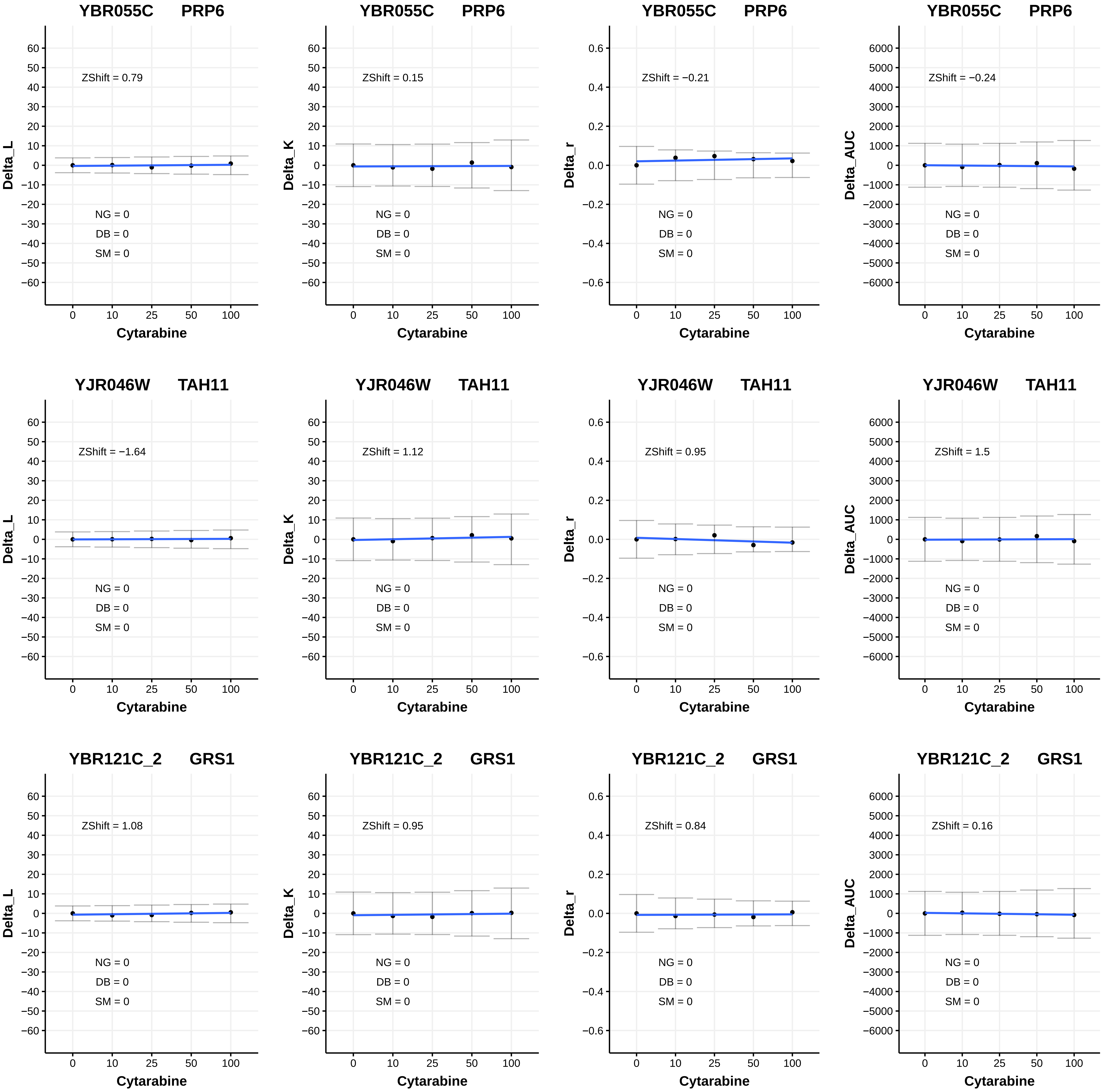
YBR055C PRP6
ZShift = 0.79
Delta_L
NG = 0
DB = 0
SM = 0
Cytarabine
YBR055C PRP6
ZShift = 0.15
Delta_K
NG = 0
DB = 0
SM = 0
Cytarabine
YBR055C PRP6
ZShift = −0.21
Delta_r
NG = 0
DB = 0
SM = 0
Cytarabine
YBR055C PRP6
ZShift = −0.24
Delta_AUC
NG = 0
DB = 0
SM = 0
Cytarabine
YJR046W TAH11
ZShift = −1.64
Delta_L
NG = 0
DB = 0
SM = 0
Cytarabine
YJR046W TAH11
ZShift = 1.12
Delta_K
NG = 0
DB = 0
SM = 0
Cytarabine
YJR046W TAH11
ZShift = 0.95
Delta_r
NG = 0
DB = 0
SM = 0
Cytarabine
YJR046W TAH11
ZShift = 1.5
Delta_AUC
NG = 0
DB = 0
SM = 0
Cytarabine
YBR121C_2 GRS1
ZShift = 1.08
Delta_L
NG = 0
DB = 0
SM = 0
Cytarabine
YBR121C_2 GRS1
ZShift = 0.95
Delta_K
NG = 0
DB = 0
SM = 0
Cytarabine
YBR121C_2 GRS1
ZShift = 0.84
Delta_r
NG = 0
DB = 0
SM = 0
Cytarabine
YBR121C_2 GRS1
ZShift = 0.16
Delta_AUC
NG = 0
DB = 0
SM = 0
Cytarabine

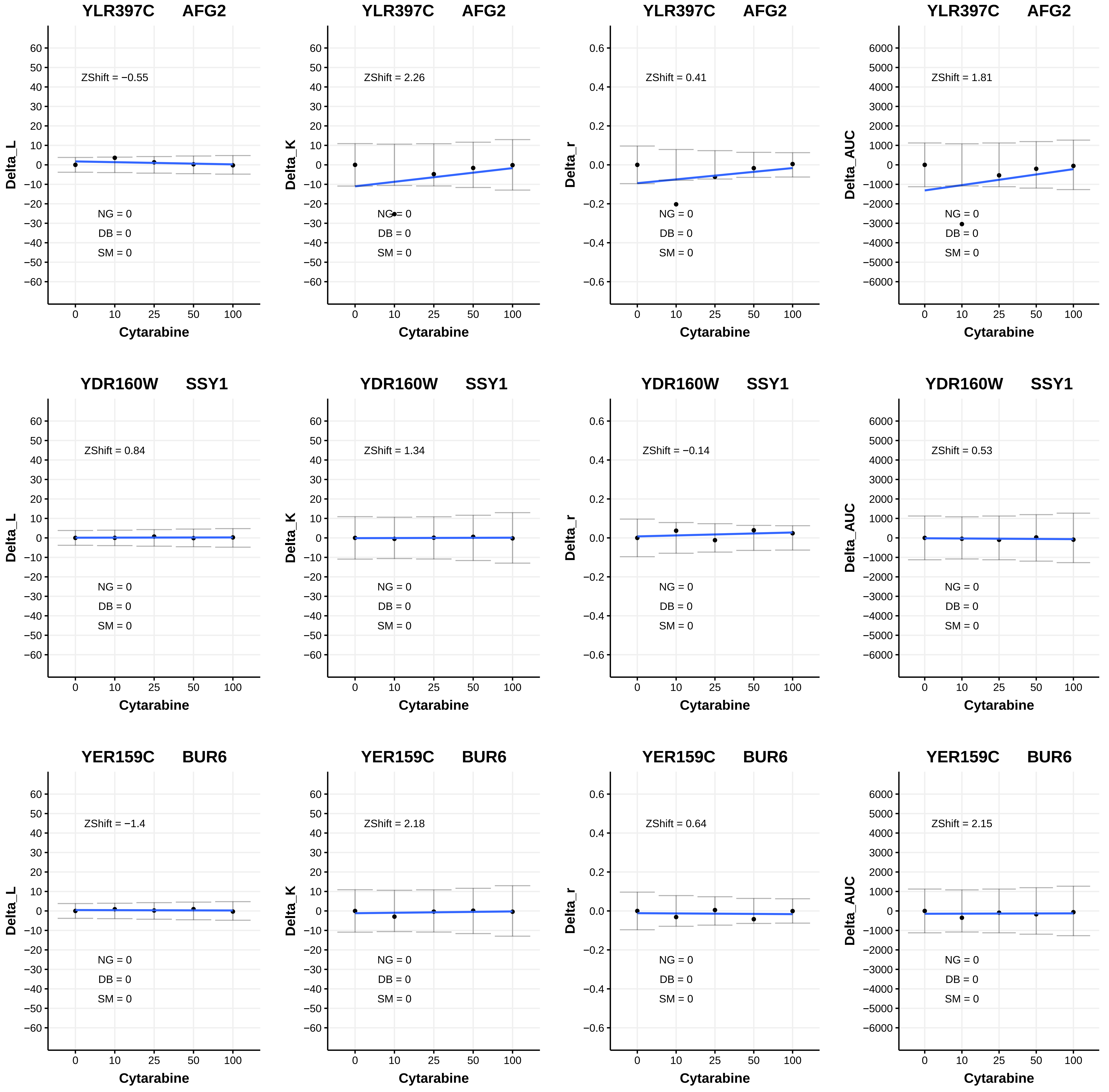

YLR397C AFG2
ZShift = −0.55
ZShift = 2.26
ZShift = 0.41
ZShift = 1.81
YDR160W SSY1
ZShift = 0.84
ZShift = 1.34
ZShift = −0.14
ZShift = 0.53
YER159C BUR6
ZShift = −1.4
ZShift = 2.18
ZShift = 0.64
ZShift = 2.15
NG = 0
DB = 0
SM = 0
Delta_L
Delta_K
Delta_r
Delta_AUC
Cytarabine
0
10
25
50
100

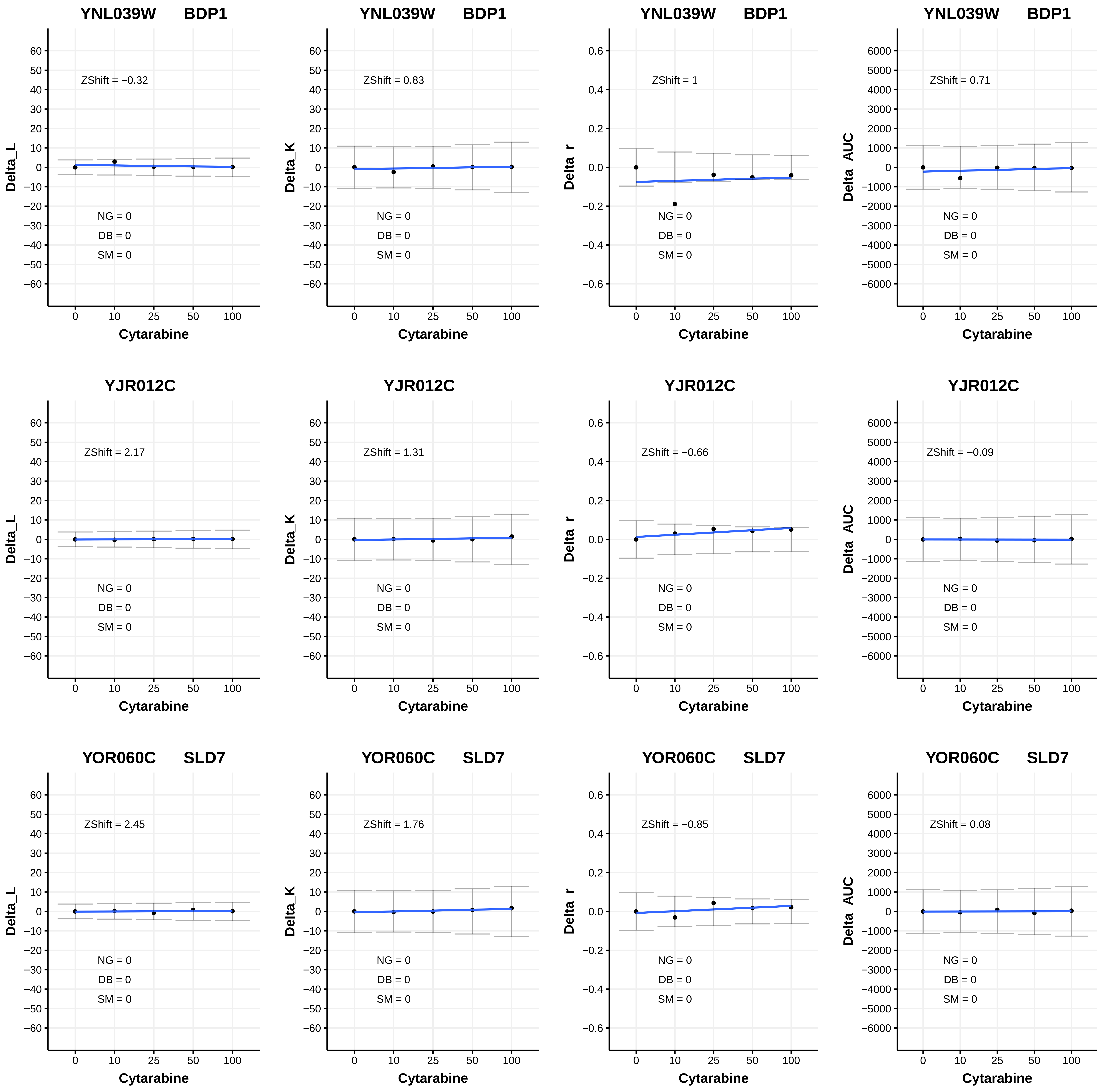

YNL039W BDP1
ZShift = −0.32
NG = 0
DB = 0
SM = 0
Delta_L
Cytarabine
YNL039W BDP1
ZShift = 0.83
NG = 0
DB = 0
SM = 0
Delta_K
Cytarabine
YNL039W BDP1
ZShift = 1
NG = 0
DB = 0
SM = 0
Delta_r
Cytarabine
YNL039W BDP1
ZShift = 0.71
NG = 0
DB = 0
SM = 0
Delta_AUC
Cytarabine
YJR012C
ZShift = 2.17
NG = 0
DB = 0
SM = 0
Delta_L
Cytarabine
YJR012C
ZShift = 1.31
NG = 0
DB = 0
SM = 0
Delta_K
Cytarabine
YJR012C
ZShift = −0.66
NG = 0
DB = 0
SM = 0
Delta_r
Cytarabine
YJR012C
ZShift = −0.09
NG = 0
DB = 0
SM = 0
Delta_AUC
Cytarabine
YOR060C SLD7
ZShift = 2.45
NG = 0
DB = 0
SM = 0
Delta_L
Cytarabine
YOR060C SLD7
ZShift = 1.76
NG = 0
DB = 0
SM = 0
Delta_K
Cytarabine
YOR060C SLD7
ZShift = −0.85
NG = 0
DB = 0
SM = 0
Delta_r
Cytarabine
YOR060C SLD7
ZShift = 0.08
NG = 0
DB = 0
SM = 0
Delta_AUC
Cytarabine

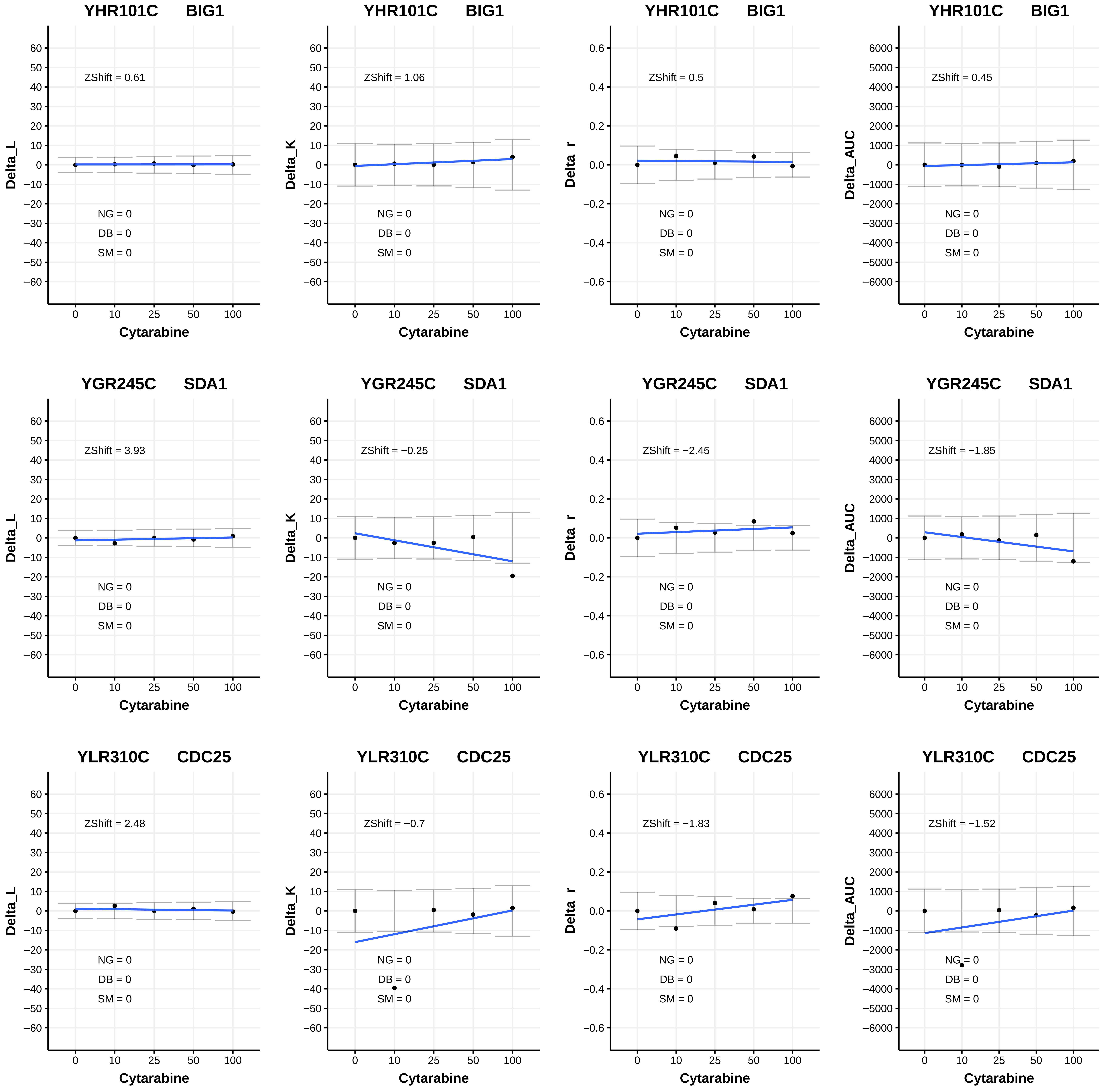
YHR101C BIG1
ZShift = 0.61
NG = 0
DB = 0
SM = 0
Delta_L
Cytarabine
YHR101C BIG1
ZShift = 1.06
NG = 0
DB = 0
SM = 0
Delta_K
Cytarabine
YHR101C BIG1
ZShift = 0.5
NG = 0
DB = 0
SM = 0
Delta_r
Cytarabine
YHR101C BIG1
ZShift = 0.45
NG = 0
DB = 0
SM = 0
Delta_AUC
Cytarabine
YGR245C SDA1
ZShift = 3.93
NG = 0
DB = 0
SM = 0
Delta_L
Cytarabine
YGR245C SDA1
ZShift = −0.25
NG = 0
DB = 0
SM = 0
Delta_K
Cytarabine
YGR245C SDA1
ZShift = −2.45
NG = 0
DB = 0
SM = 0
Delta_r
Cytarabine
YGR245C SDA1
ZShift = −1.85
NG = 0
DB = 0
SM = 0
Delta_AUC
Cytarabine
YLR310C CDC25
ZShift = 2.48
NG = 0
DB = 0
SM = 0
Delta_L
Cytarabine
YLR310C CDC25
ZShift = −0.7
NG = 0
DB = 0
SM = 0
Delta_K
Cytarabine
YLR310C CDC25
ZShift = −1.83
NG = 0
DB = 0
SM = 0
Delta_r
Cytarabine
YLR310C CDC25
ZShift = −1.52
NG = 0
DB = 0
SM = 0
Delta_AUC
Cytarabine

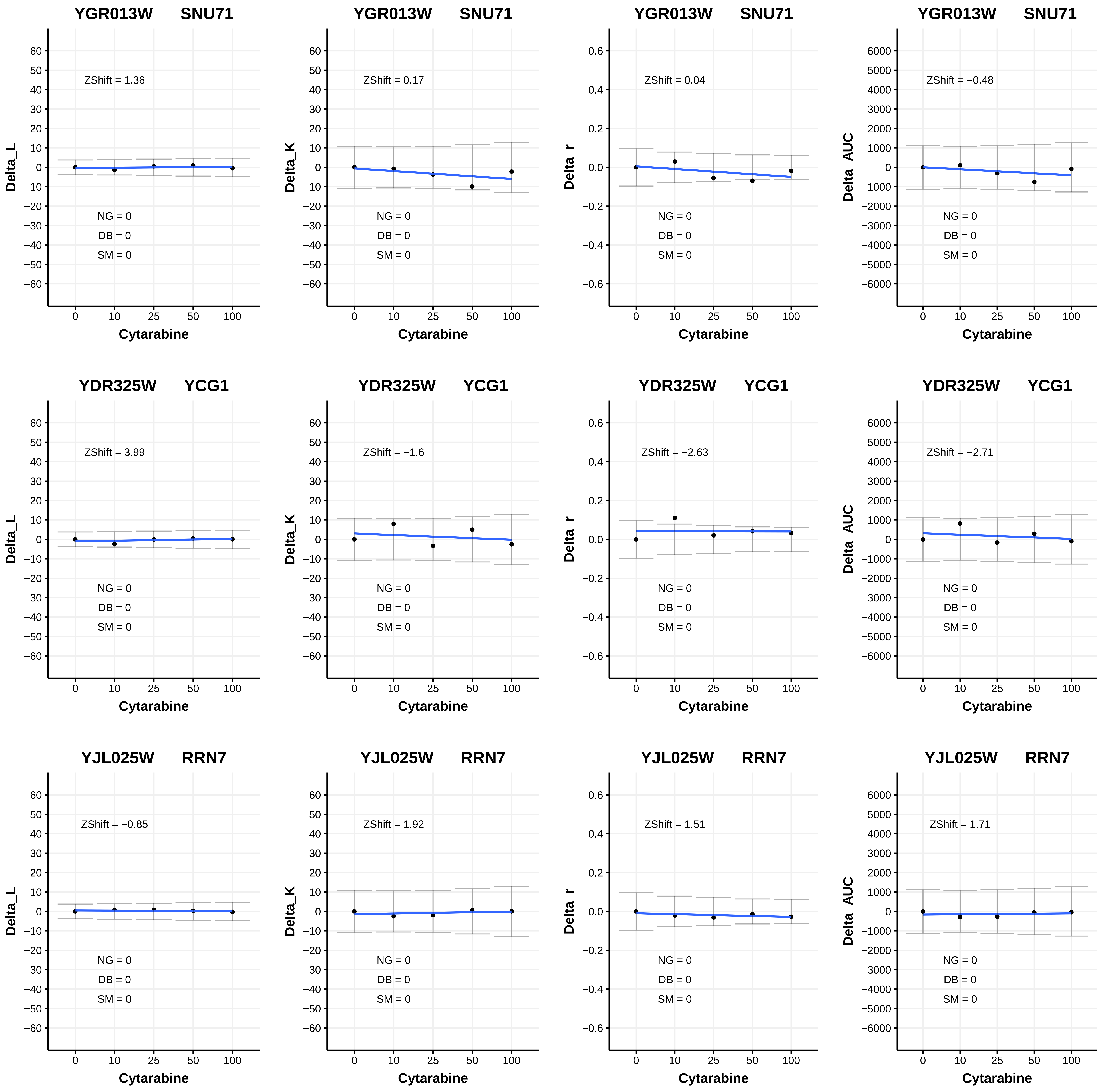

YGR013W SNU71
ZShift = 1.36
ZShift = 0.17
ZShift = 0.04
ZShift = −0.48
YDR325W YCG1
ZShift = 3.99
ZShift = −1.6
ZShift = −2.63
ZShift = −2.71
YJL025W RRN7
ZShift = −0.85
ZShift = 1.92
ZShift = 1.51
ZShift = 1.71
NG = 0
DB = 0
SM = 0
Delta_L
Delta_K
Delta_r
Delta_AUC
Cytarabine

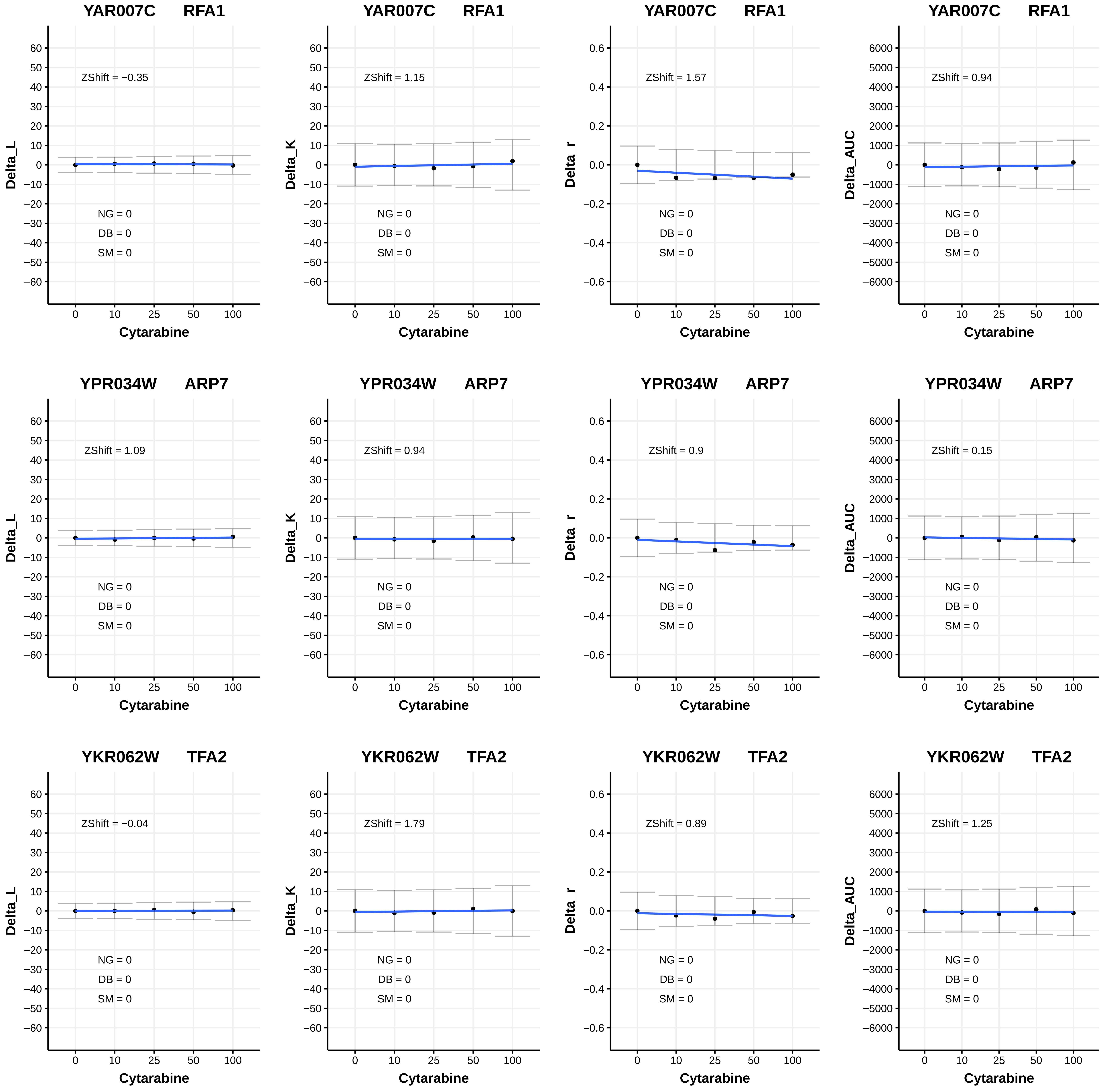
YAR007C RFA1
ZShift = −0.35
NG = 0
DB = 0
SM = 0
Delta_L
Cytarabine
YAR007C RFA1
ZShift = 1.15
NG = 0
DB = 0
SM = 0
Delta_K
Cytarabine
YAR007C RFA1
ZShift = 1.57
NG = 0
DB = 0
SM = 0
Delta_r
Cytarabine
YAR007C RFA1
ZShift = 0.94
NG = 0
DB = 0
SM = 0
Delta_AUC
Cytarabine
YPR034W ARP7
ZShift = 1.09
NG = 0
DB = 0
SM = 0
Delta_L
Cytarabine
YPR034W ARP7
ZShift = 0.94
NG = 0
DB = 0
SM = 0
Delta_K
Cytarabine
YPR034W ARP7
ZShift = 0.9
NG = 0
DB = 0
SM = 0
Delta_r
Cytarabine
YPR034W ARP7
ZShift = 0.15
NG = 0
DB = 0
SM = 0
Delta_AUC
Cytarabine
YKR062W TFA2
ZShift = −0.04
NG = 0
DB = 0
SM = 0
Delta_L
Cytarabine
YKR062W TFA2
ZShift = 1.79
NG = 0
DB = 0
SM = 0
Delta_K
Cytarabine
YKR062W TFA2
ZShift = 0.89
NG = 0
DB = 0
SM = 0
Delta_r
Cytarabine
YKR062W TFA2
ZShift = 1.25
NG = 0
DB = 0
SM = 0
Delta_AUC
Cytarabine

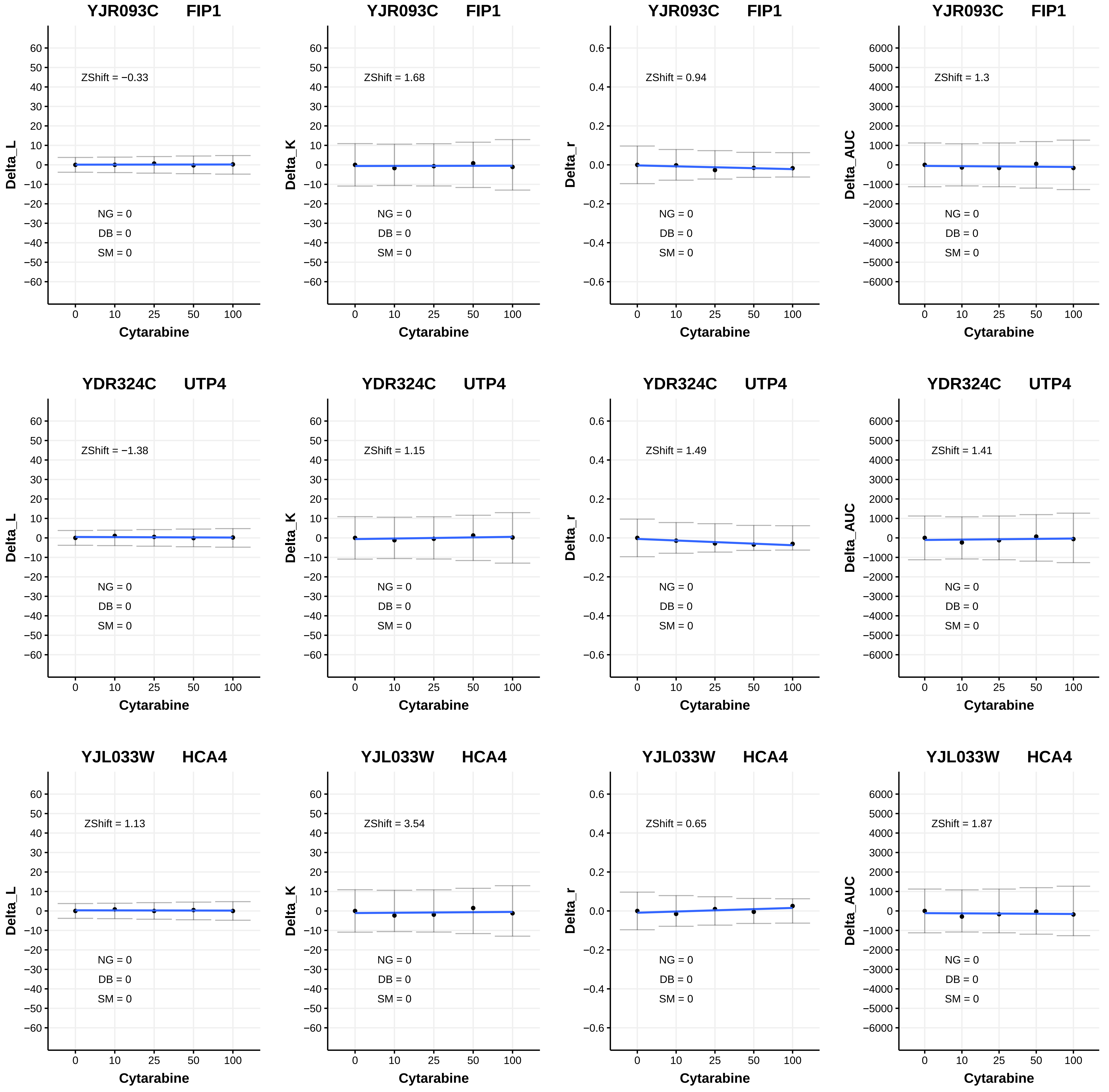

YJR093C FIP1
ZShift = −0.33
NG = 0
DB = 0
SM = 0
Delta_L
Cytarabine
YJR093C FIP1
ZShift = 1.68
NG = 0
DB = 0
SM = 0
Delta_K
Cytarabine
YJR093C FIP1
ZShift = 0.94
NG = 0
DB = 0
SM = 0
Delta_r
Cytarabine
YJR093C FIP1
ZShift = 1.3
NG = 0
DB = 0
SM = 0
Delta_AUC
Cytarabine
YDR324C UTP4
ZShift = −1.38
NG = 0
DB = 0
SM = 0
Delta_L
Cytarabine
YDR324C UTP4
ZShift = 1.15
NG = 0
DB = 0
SM = 0
Delta_K
Cytarabine
YDR324C UTP4
ZShift = 1.49
NG = 0
DB = 0
SM = 0
Delta_r
Cytarabine
YDR324C UTP4
ZShift = 1.41
NG = 0
DB = 0
SM = 0
Delta_AUC
Cytarabine
YJL033W HCA4
ZShift = 1.13
NG = 0
DB = 0
SM = 0
Delta_L
Cytarabine
YJL033W HCA4
ZShift = 3.54
NG = 0
DB = 0
SM = 0
Delta_K
Cytarabine
YJL033W HCA4
ZShift = 0.65
NG = 0
DB = 0
SM = 0
Delta_r
Cytarabine
YJL033W HCA4
ZShift = 1.87
NG = 0
DB = 0
SM = 0
Delta_AUC
Cytarabine

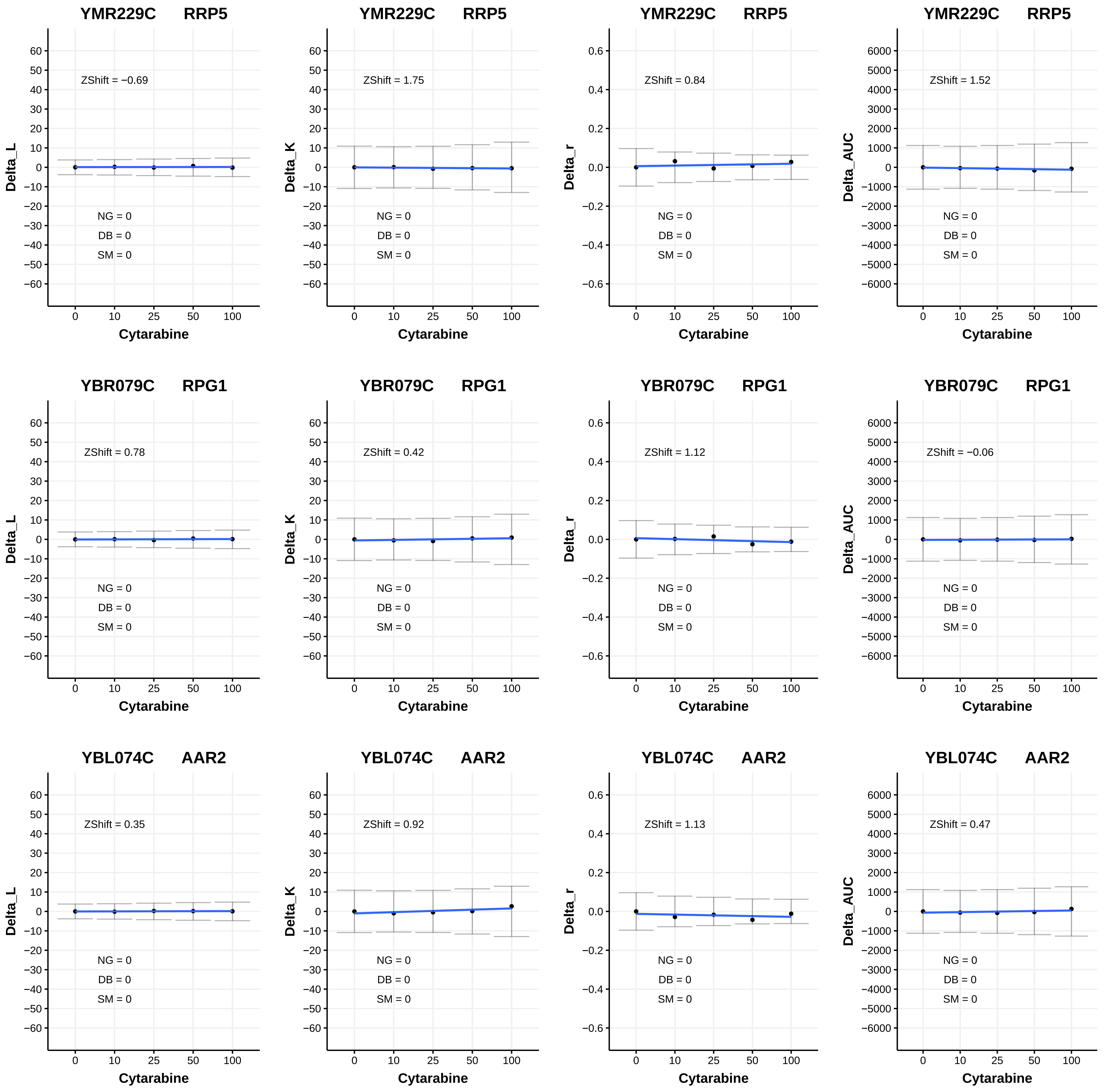
YMR229C RRP5
ZShift = −0.69
NG = 0
DB = 0
SM = 0
Delta_L
Cytarabine
YMR229C RRP5
ZShift = 1.75
NG = 0
DB = 0
SM = 0
Delta_K
Cytarabine
YMR229C RRP5
ZShift = 0.84
NG = 0
DB = 0
SM = 0
Delta_r
Cytarabine
YMR229C RRP5
ZShift = 1.52
NG = 0
DB = 0
SM = 0
Delta_AUC
Cytarabine
YBR079C RPG1
ZShift = 0.78
NG = 0
DB = 0
SM = 0
Delta_L
Cytarabine
YBR079C RPG1
ZShift = 0.42
NG = 0
DB = 0
SM = 0
Delta_K
Cytarabine
YBR079C RPG1
ZShift = 1.12
NG = 0
DB = 0
SM = 0
Delta_r
Cytarabine
YBR079C RPG1
ZShift = −0.06
NG = 0
DB = 0
SM = 0
Delta_AUC
Cytarabine
YBL074C AAR2
ZShift = 0.35
NG = 0
DB = 0
SM = 0
Delta_L
Cytarabine
YBL074C AAR2
ZShift = 0.92
NG = 0
DB = 0
SM = 0
Delta_K
Cytarabine
YBL074C AAR2
ZShift = 1.13
NG = 0
DB = 0
SM = 0
Delta_r
Cytarabine
YBL074C AAR2
ZShift = 0.47
NG = 0
DB = 0
SM = 0
Delta_AUC
Cytarabine

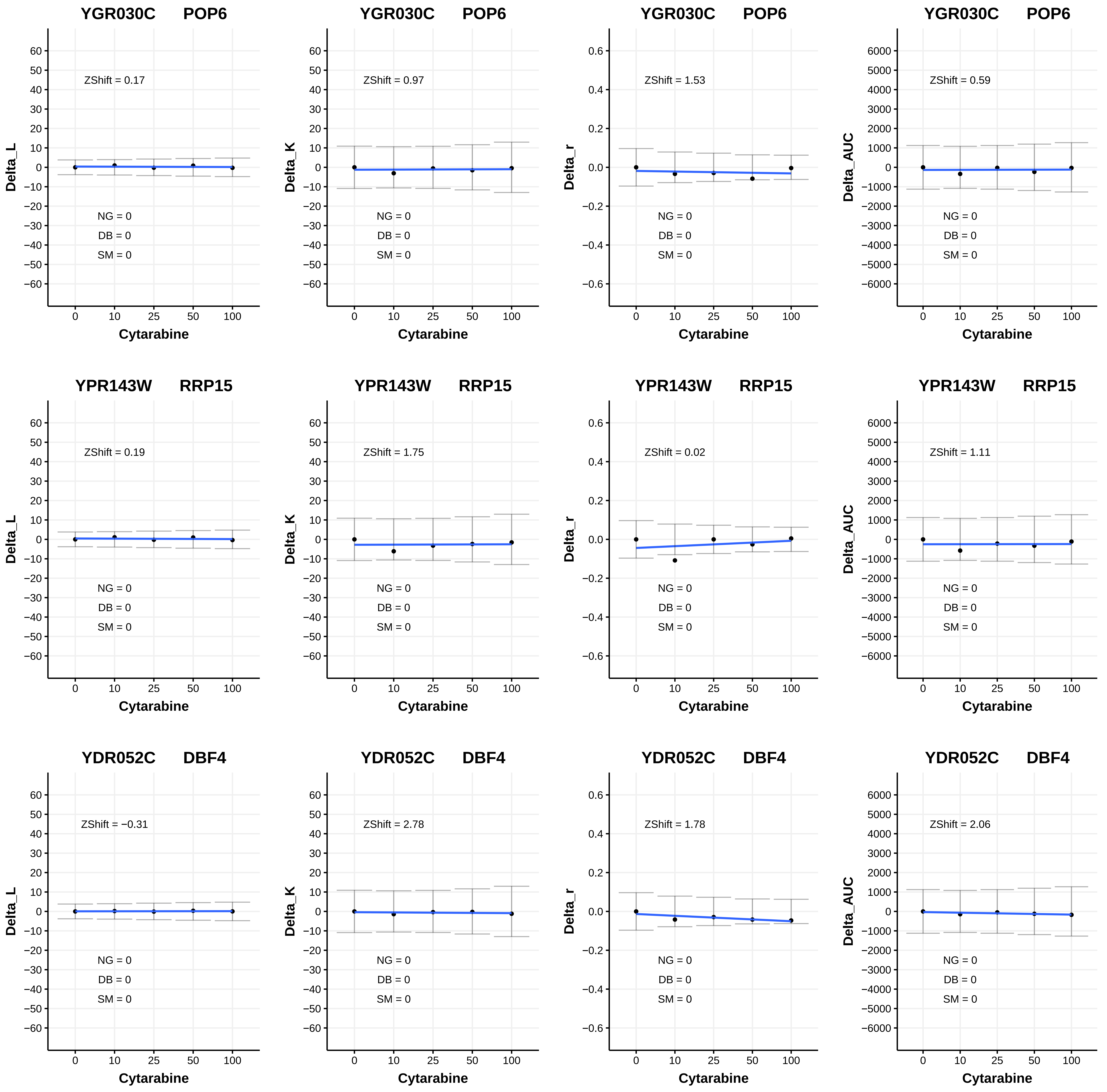
YGR030C POP6
ZShift = 0.17
NG = 0
DB = 0
SM = 0
Delta_L
Cytarabine
YGR030C POP6
ZShift = 0.97
NG = 0
DB = 0
SM = 0
Delta_K
Cytarabine
YGR030C POP6
ZShift = 1.53
NG = 0
DB = 0
SM = 0
Delta_r
Cytarabine
YGR030C POP6
ZShift = 0.59
NG = 0
DB = 0
SM = 0
Delta_AUC
Cytarabine
YPR143W RRP15
ZShift = 0.19
NG = 0
DB = 0
SM = 0
Delta_L
Cytarabine
YPR143W RRP15
ZShift = 1.75
NG = 0
DB = 0
SM = 0
Delta_K
Cytarabine
YPR143W RRP15
ZShift = 0.02
NG = 0
DB = 0
SM = 0
Delta_r
Cytarabine
YPR143W RRP15
ZShift = 1.11
NG = 0
DB = 0
SM = 0
Delta_AUC
Cytarabine
YDR052C DBF4
ZShift = −0.31
NG = 0
DB = 0
SM = 0
Delta_L
Cytarabine
YDR052C DBF4
ZShift = 2.78
NG = 0
DB = 0
SM = 0
Delta_K
Cytarabine
YDR052C DBF4
ZShift = 1.78
NG = 0
DB = 0
SM = 0
Delta_r
Cytarabine
YDR052C DBF4
ZShift = 2.06
NG = 0
DB = 0
SM = 0
Delta_AUC
Cytarabine

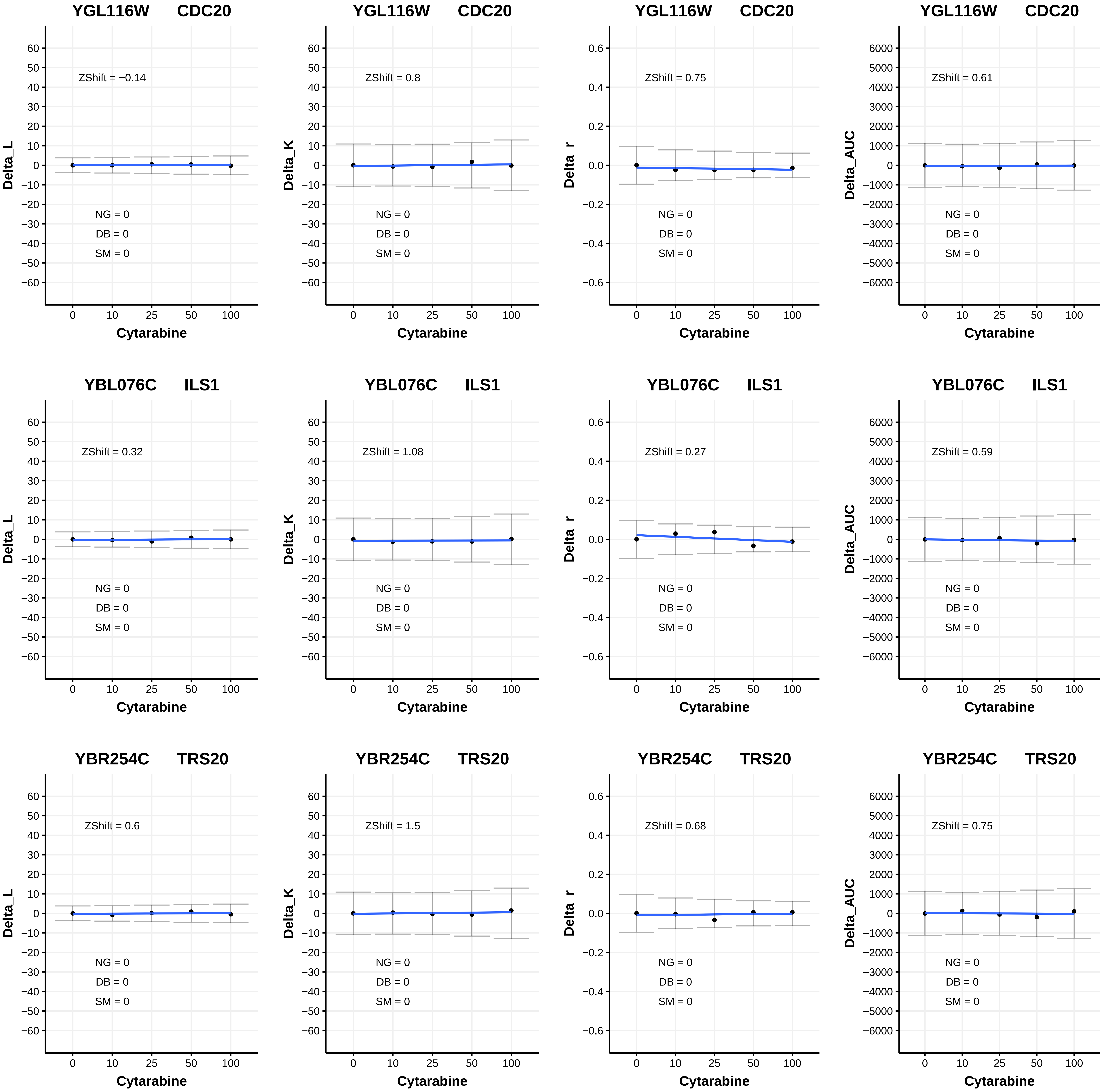
YGL116W CDC20
Delta_L
ZShift = −0.14
NG = 0
DB = 0
SM = 0
Cytarabine
YGL116W CDC20
Delta_K
ZShift = 0.8
NG = 0
DB = 0
SM = 0
Cytarabine
YGL116W CDC20
Delta_r
ZShift = 0.75
NG = 0
DB = 0
SM = 0
Cytarabine
YGL116W CDC20
Delta_AUC
ZShift = 0.61
NG = 0
DB = 0
SM = 0
Cytarabine
YBL076C ILS1
Delta_L
ZShift = 0.32
NG = 0
DB = 0
SM = 0
Cytarabine
YBL076C ILS1
Delta_K
ZShift = 1.08
NG = 0
DB = 0
SM = 0
Cytarabine
YBL076C ILS1
Delta_r
ZShift = 0.27
NG = 0
DB = 0
SM = 0
Cytarabine
YBL076C ILS1
Delta_AUC
ZShift = 0.59
NG = 0
DB = 0
SM = 0
Cytarabine
YBR254C TRS20
Delta_L
ZShift = 0.6
NG = 0
DB = 0
SM = 0
Cytarabine
YBR254C TRS20
Delta_K
ZShift = 1.5
NG = 0
DB = 0
SM = 0
Cytarabine
YBR254C TRS20
Delta_r
ZShift = 0.68
NG = 0
DB = 0
SM = 0
Cytarabine
YBR254C TRS20
Delta_AUC
ZShift = 0.75
NG = 0
DB = 0
SM = 0
Cytarabine

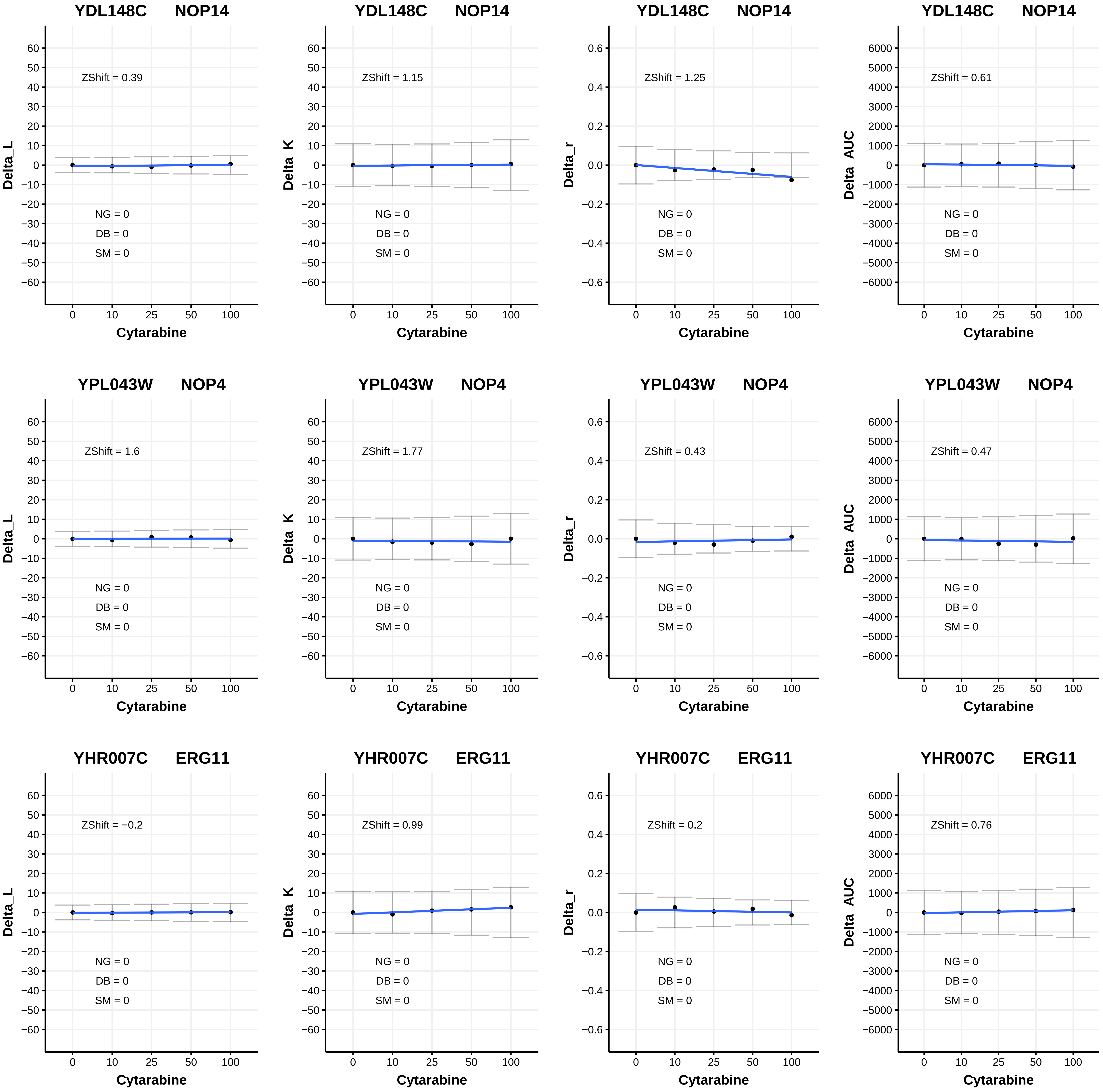

YDL148C NOP14
ZShift = 0.39
Delta_L
NG = 0
DB = 0
SM = 0
Cytarabine
YDL148C NOP14
ZShift = 1.15
Delta_K
NG = 0
DB = 0
SM = 0
Cytarabine
YDL148C NOP14
ZShift = 1.25
Delta_r
NG = 0
DB = 0
SM = 0
Cytarabine
YDL148C NOP14
ZShift = 0.61
Delta_AUC
NG = 0
DB = 0
SM = 0
Cytarabine
YPL043W NOP4
ZShift = 1.6
Delta_L
NG = 0
DB = 0
SM = 0
Cytarabine
YPL043W NOP4
ZShift = 1.77
Delta_K
NG = 0
DB = 0
SM = 0
Cytarabine
YPL043W NOP4
ZShift = 0.43
Delta_r
NG = 0
DB = 0
SM = 0
Cytarabine
YPL043W NOP4
ZShift = 0.47
Delta_AUC
NG = 0
DB = 0
SM = 0
Cytarabine
YHR007C ERG11
ZShift = −0.2
Delta_L
NG = 0
DB = 0
SM = 0
Cytarabine
YHR007C ERG11
ZShift = 0.99
Delta_K
NG = 0
DB = 0
SM = 0
Cytarabine
YHR007C ERG11
ZShift = 0.2
Delta_r
NG = 0
DB = 0
SM = 0
Cytarabine
YHR007C ERG11
ZShift = 0.76
Delta_AUC
NG = 0
DB = 0
SM = 0
Cytarabine

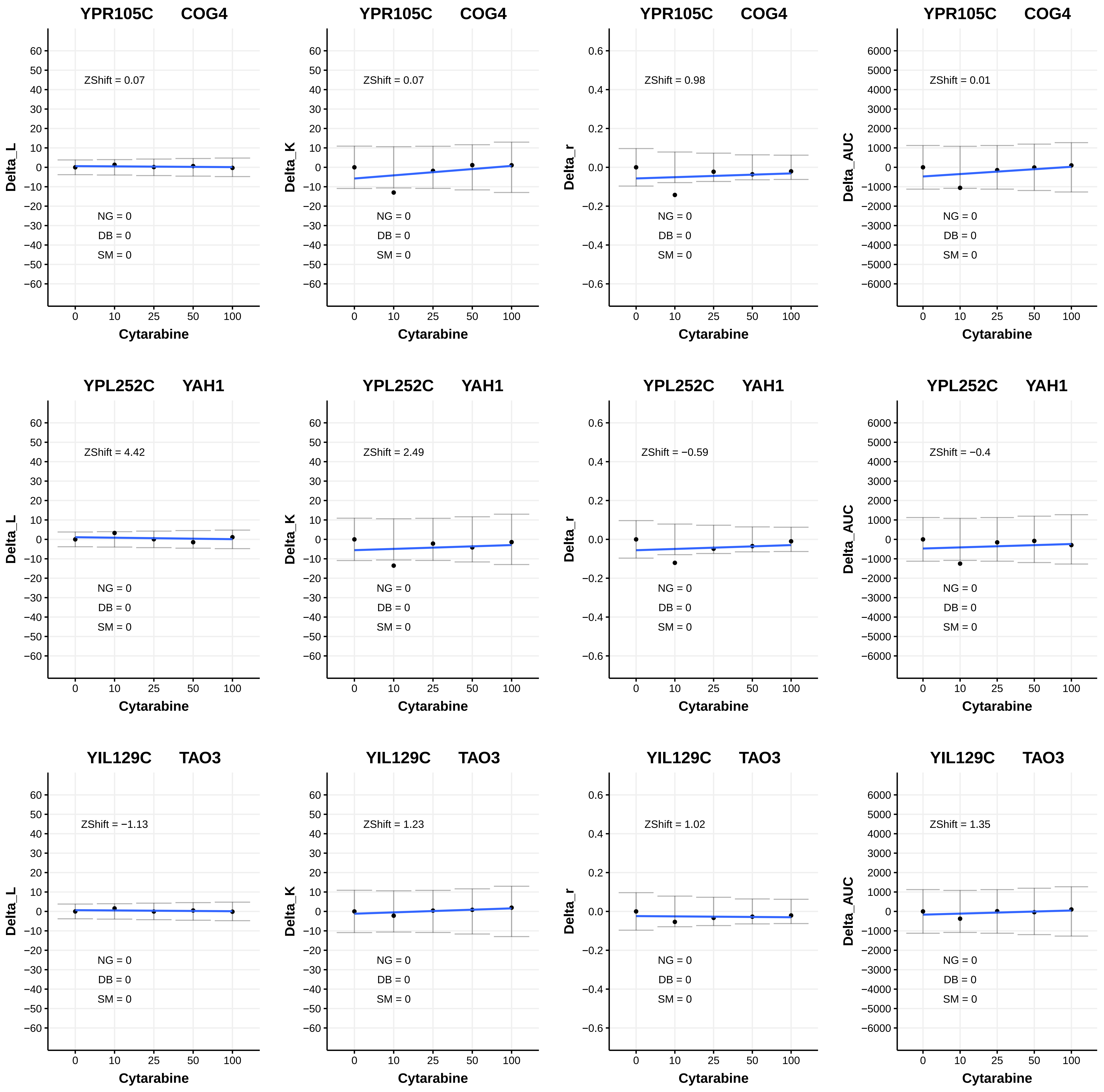
YPR105C COG4
ZShift = 0.07
NG = 0
DB = 0
SM = 0
Delta_L
Cytarabine
YPR105C COG4
ZShift = 0.07
NG = 0
DB = 0
SM = 0
Delta_K
Cytarabine
YPR105C COG4
ZShift = 0.98
NG = 0
DB = 0
SM = 0
Delta_r
Cytarabine
YPR105C COG4
ZShift = 0.01
NG = 0
DB = 0
SM = 0
Delta_AUC
Cytarabine
YPL252C YAH1
ZShift = 4.42
NG = 0
DB = 0
SM = 0
Delta_L
Cytarabine
YPL252C YAH1
ZShift = 2.49
NG = 0
DB = 0
SM = 0
Delta_K
Cytarabine
YPL252C YAH1
ZShift = −0.59
NG = 0
DB = 0
SM = 0
Delta_r
Cytarabine
YPL252C YAH1
ZShift = −0.4
NG = 0
DB = 0
SM = 0
Delta_AUC
Cytarabine
YIL129C TAO3
ZShift = −1.13
NG = 0
DB = 0
SM = 0
Delta_L
Cytarabine
YIL129C TAO3
ZShift = 1.23
NG = 0
DB = 0
SM = 0
Delta_K
Cytarabine
YIL129C TAO3
ZShift = 1.02
NG = 0
DB = 0
SM = 0
Delta_r
Cytarabine
YIL129C TAO3
ZShift = 1.35
NG = 0
DB = 0
SM = 0
Delta_AUC
Cytarabine

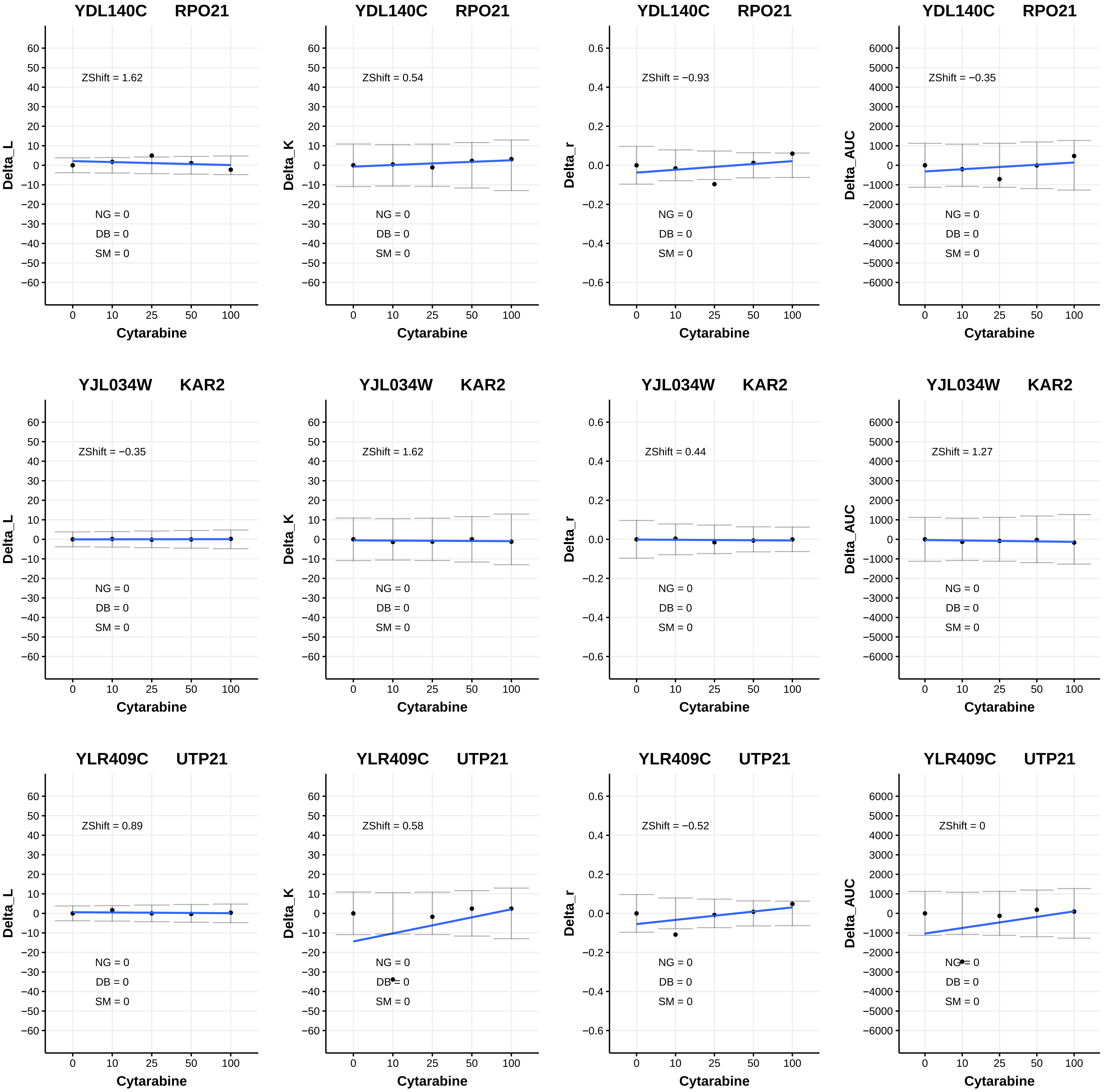

YDL140C RPO21
ZShift = 1.62
NG = 0
DB = 0
SM = 0
Delta_L
Cytarabine
YDL140C RPO21
ZShift = 0.54
NG = 0
DB = 0
SM = 0
Delta_K
Cytarabine
YDL140C RPO21
ZShift = −0.93
NG = 0
DB = 0
SM = 0
Delta_r
Cytarabine
YDL140C RPO21
ZShift = −0.35
NG = 0
DB = 0
SM = 0
Delta_AUC
Cytarabine
YJL034W KAR2
ZShift = −0.35
NG = 0
DB = 0
SM = 0
Delta_L
Cytarabine
YJL034W KAR2
ZShift = 1.62
NG = 0
DB = 0
SM = 0
Delta_K
Cytarabine
YJL034W KAR2
ZShift = 0.44
NG = 0
DB = 0
SM = 0
Delta_r
Cytarabine
YJL034W KAR2
ZShift = 1.27
NG = 0
DB = 0
SM = 0
Delta_AUC
Cytarabine
YLR409C UTP21
ZShift = 0.89
NG = 0
DB = 0
SM = 0
Delta_L
Cytarabine
YLR409C UTP21
ZShift = 0.58
NG = 0
DB = 0
SM = 0
Delta_K
Cytarabine
YLR409C UTP21
ZShift = −0.52
NG = 0
DB = 0
SM = 0
Delta_r
Cytarabine
YLR409C UTP21
ZShift = 0
NG = 0
DB = 0
SM = 0
Delta_AUC
Cytarabine

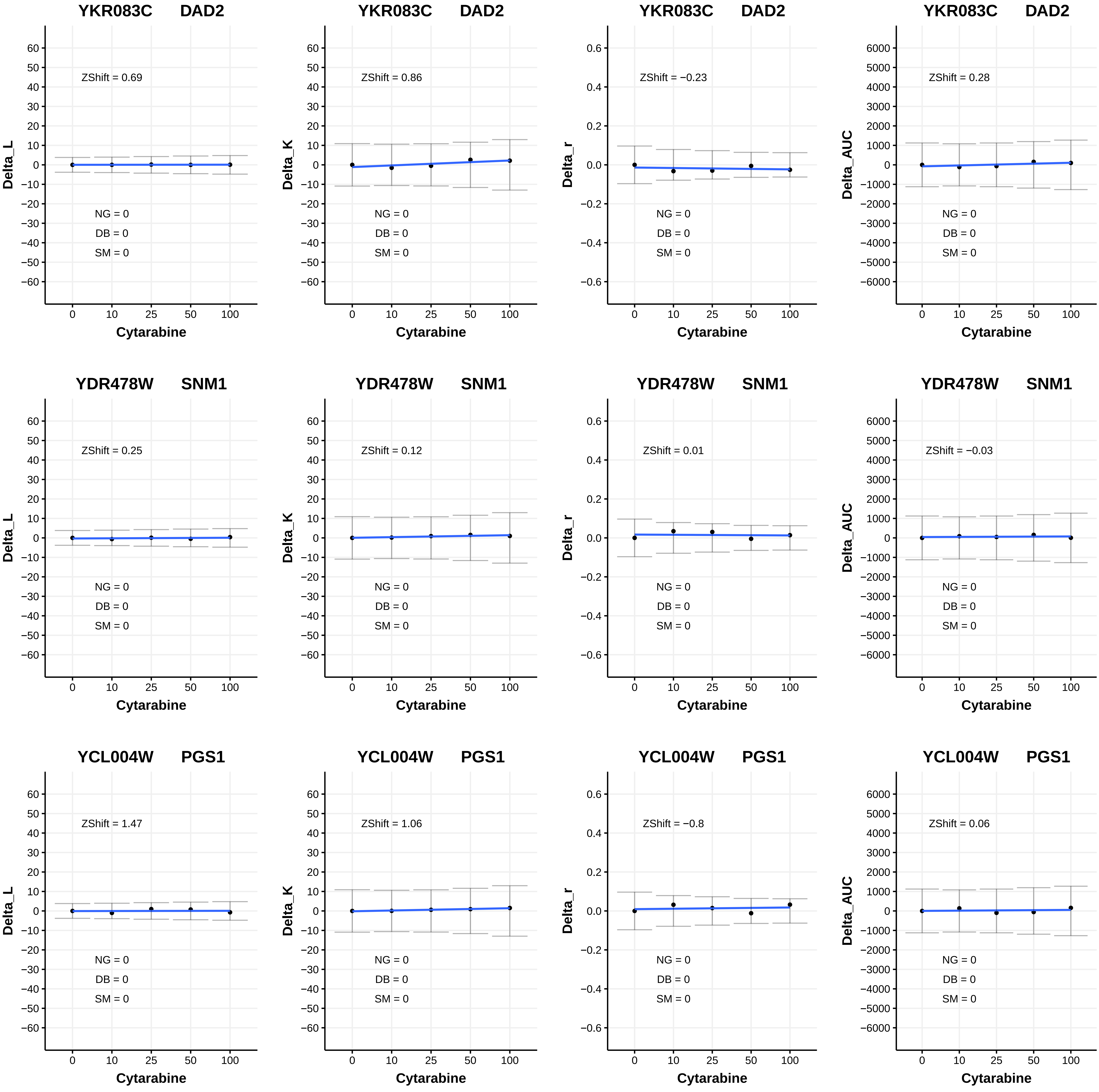
YKR083C DAD2
ZShift = 0.69
NG = 0
DB = 0
SM = 0
Delta_L
Cytarabine
YKR083C DAD2
ZShift = 0.86
NG = 0
DB = 0
SM = 0
Delta_K
Cytarabine
YKR083C DAD2
ZShift = −0.23
NG = 0
DB = 0
SM = 0
Delta_r
Cytarabine
YKR083C DAD2
ZShift = 0.28
NG = 0
DB = 0
SM = 0
Delta_AUC
Cytarabine
YDR478W SNM1
ZShift = 0.25
NG = 0
DB = 0
SM = 0
Delta_L
Cytarabine
YDR478W SNM1
ZShift = 0.12
NG = 0
DB = 0
SM = 0
Delta_K
Cytarabine
YDR478W SNM1
ZShift = 0.01
NG = 0
DB = 0
SM = 0
Delta_r
Cytarabine
YDR478W SNM1
ZShift = −0.03
NG = 0
DB = 0
SM = 0
Delta_AUC
Cytarabine
YCL004W PGS1
ZShift = 1.47
NG = 0
DB = 0
SM = 0
Delta_L
Cytarabine
YCL004W PGS1
ZShift = 1.06
NG = 0
DB = 0
SM = 0
Delta_K
Cytarabine
YCL004W PGS1
ZShift = −0.8
NG = 0
DB = 0
SM = 0
Delta_r
Cytarabine
YCL004W PGS1
ZShift = 0.06
NG = 0
DB = 0
SM = 0
Delta_AUC
Cytarabine

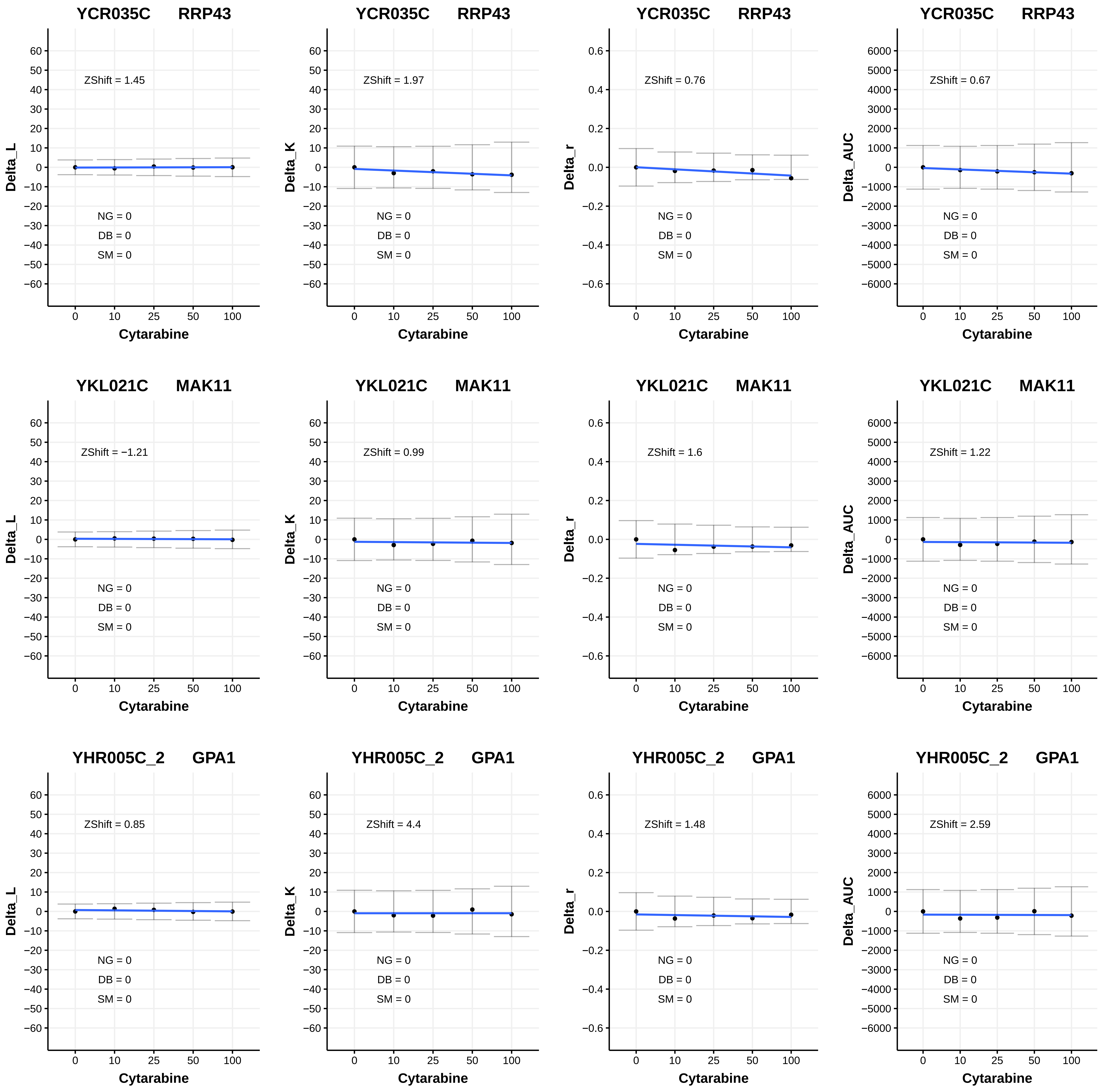
YCR035C RRP43
ZShift = 1.45
Delta_L
NG = 0
DB = 0
SM = 0
Cytarabine
YCR035C RRP43
ZShift = 1.97
Delta_K
NG = 0
DB = 0
SM = 0
Cytarabine
YCR035C RRP43
ZShift = 0.76
Delta_r
NG = 0
DB = 0
SM = 0
Cytarabine
YCR035C RRP43
ZShift = 0.67
Delta_AUC
NG = 0
DB = 0
SM = 0
Cytarabine
YKL021C MAK11
ZShift = −1.21
Delta_L
NG = 0
DB = 0
SM = 0
Cytarabine
YKL021C MAK11
ZShift = 0.99
Delta_K
NG = 0
DB = 0
SM = 0
Cytarabine
YKL021C MAK11
ZShift = 1.6
Delta_r
NG = 0
DB = 0
SM = 0
Cytarabine
YKL021C MAK11
ZShift = 1.22
Delta_AUC
NG = 0
DB = 0
SM = 0
Cytarabine
YHR005C_2 GPA1
ZShift = 0.85
Delta_L
NG = 0
DB = 0
SM = 0
Cytarabine
YHR005C_2 GPA1
ZShift = 4.4
Delta_K
NG = 0
DB = 0
SM = 0
Cytarabine
YHR005C_2 GPA1
ZShift = 1.48
Delta_r
NG = 0
DB = 0
SM = 0
Cytarabine
YHR005C_2 GPA1
ZShift = 2.59
Delta_AUC
NG = 0
DB = 0
SM = 0
Cytarabine

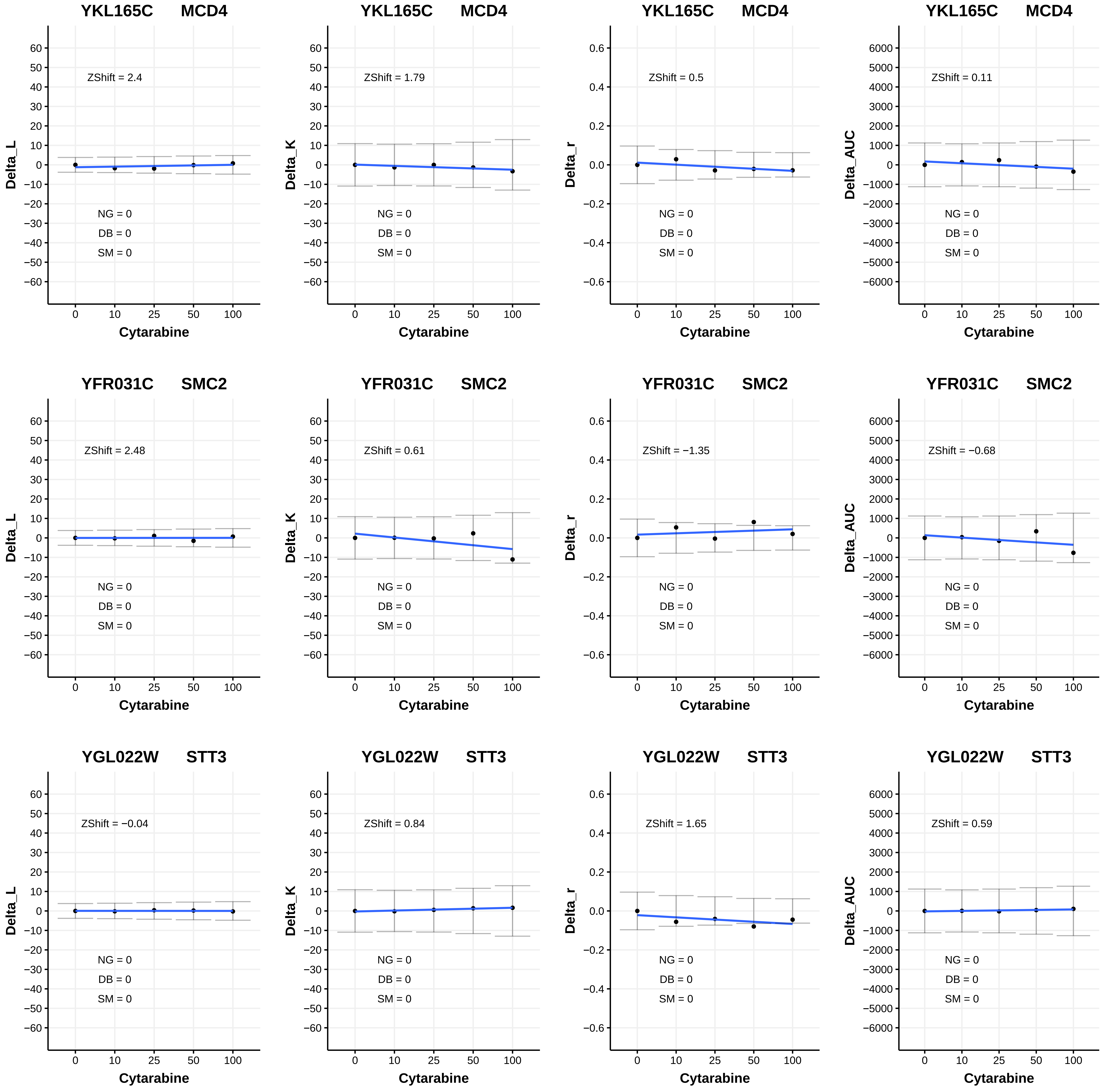
YKL165C MCD4
ZShift = 2.4
ZShift = 1.79
ZShift = 0.5
ZShift = 0.11
YFR031C SMC2
ZShift = 2.48
ZShift = 0.61
ZShift = −1.35
ZShift = −0.68
YGL022W STT3
ZShift = −0.04
ZShift = 0.84
ZShift = 1.65
ZShift = 0.59
NG = 0
DB = 0
SM = 0
Delta_L
Delta_K
Delta_r
Delta_AUC
Cytarabine

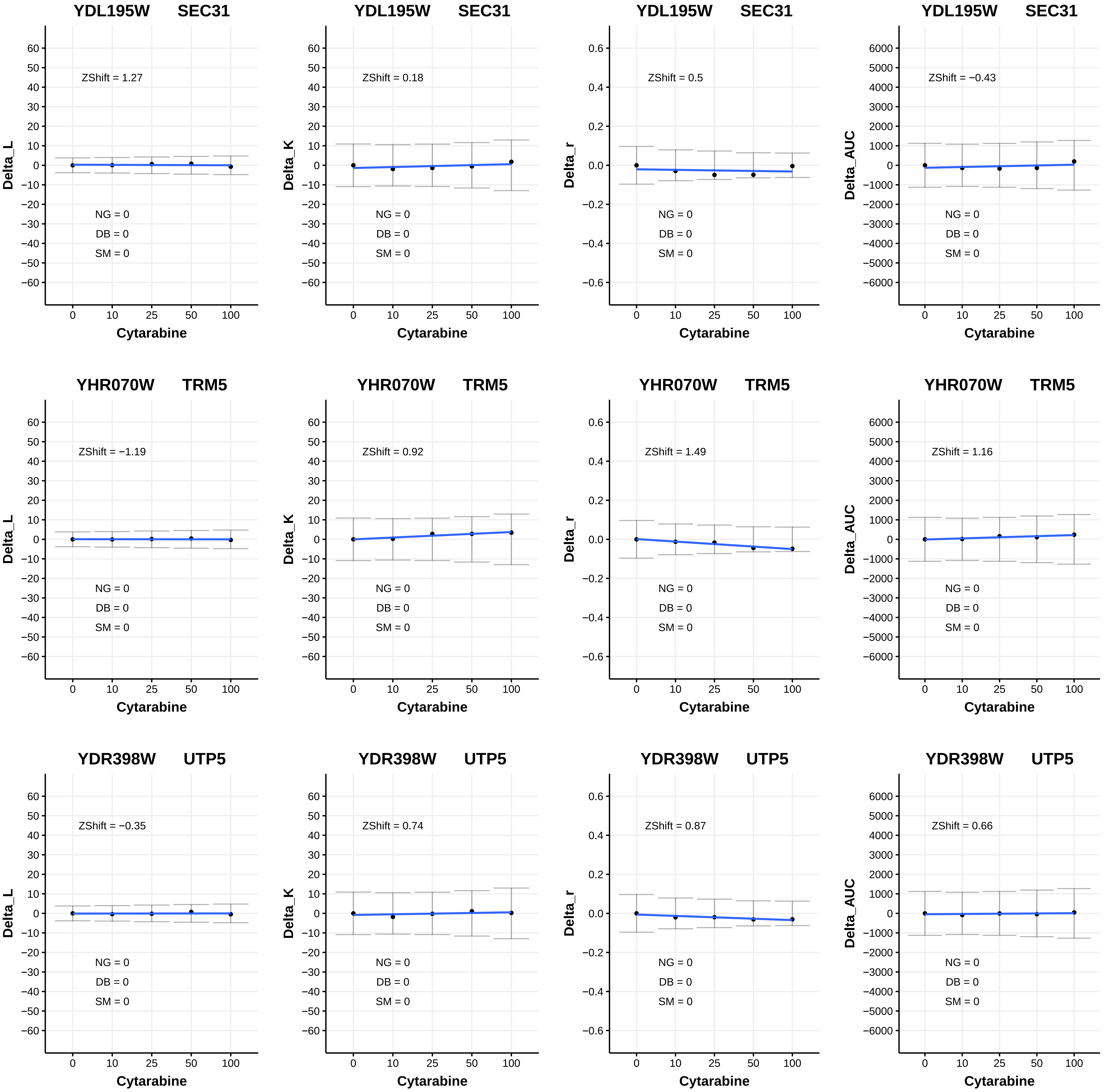
YDL195W SEC31
ZShift = 1.27
NG = 0
DB = 0
SM = 0
Delta_L
Cytarabine
YDL195W SEC31
ZShift = 0.18
NG = 0
DB = 0
SM = 0
Delta_K
Cytarabine
YDL195W SEC31
ZShift = 0.5
NG = 0
DB = 0
SM = 0
Delta_r
Cytarabine
YDL195W SEC31
ZShift = −0.43
NG = 0
DB = 0
SM = 0
Delta_AUC
Cytarabine
YHR070W TRM5
ZShift = −1.19
NG = 0
DB = 0
SM = 0
Delta_L
Cytarabine
YHR070W TRM5
ZShift = 0.92
NG = 0
DB = 0
SM = 0
Delta_K
Cytarabine
YHR070W TRM5
ZShift = 1.49
NG = 0
DB = 0
SM = 0
Delta_r
Cytarabine
YHR070W TRM5
ZShift = 1.16
NG = 0
DB = 0
SM = 0
Delta_AUC
Cytarabine
YDR398W UTP5
ZShift = −0.35
NG = 0
DB = 0
SM = 0
Delta_L
Cytarabine
YDR398W UTP5
ZShift = 0.74
NG = 0
DB = 0
SM = 0
Delta_K
Cytarabine
YDR398W UTP5
ZShift = 0.87
NG = 0
DB = 0
SM = 0
Delta_r
Cytarabine
YDR398W UTP5
ZShift = 0.66
NG = 0
DB = 0
SM = 0
Delta_AUC
Cytarabine

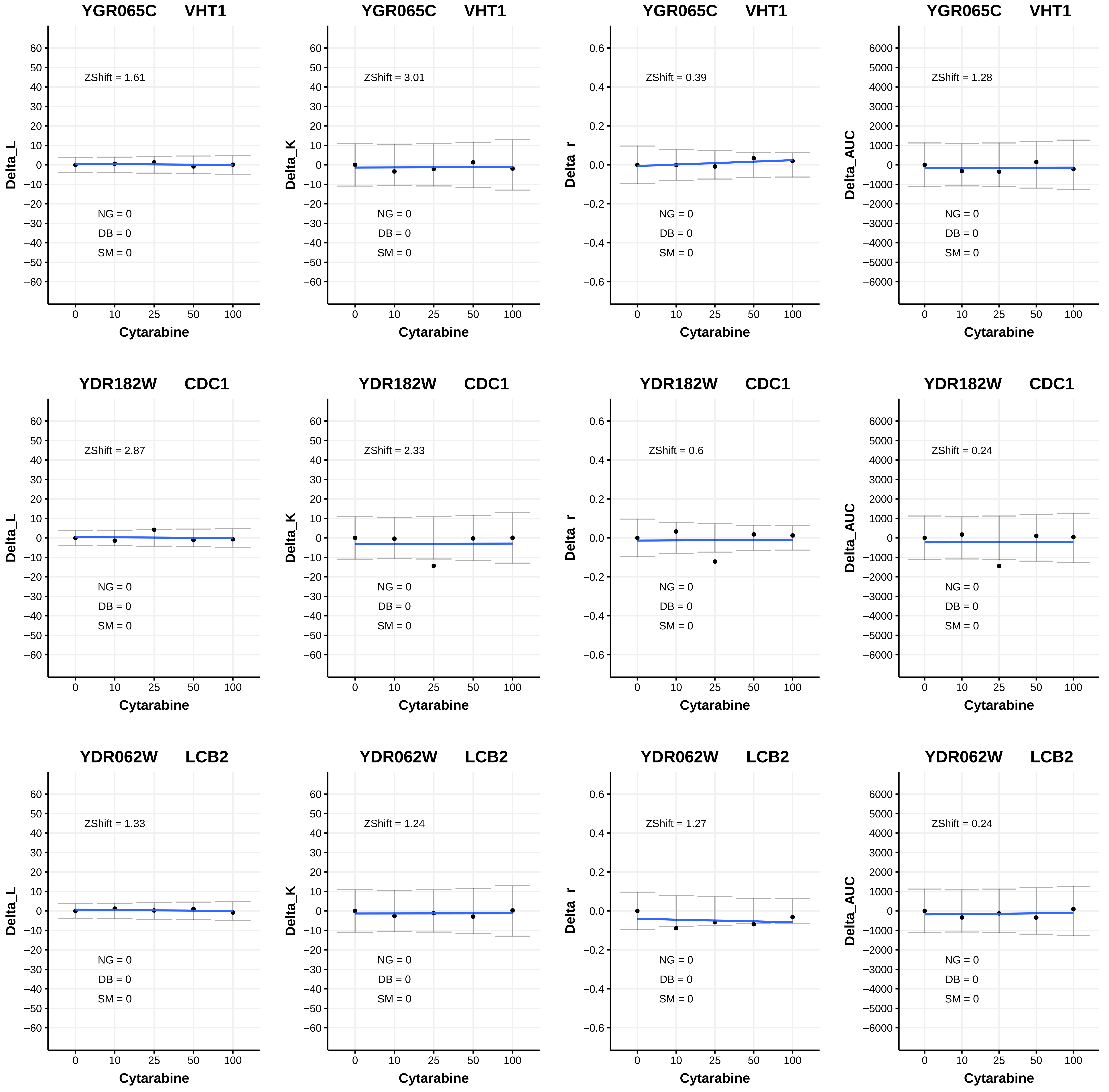
YGR065C VHT1
ZShift = 1.61
NG = 0
DB = 0
SM = 0
Delta_L
Cytarabine
YGR065C VHT1
ZShift = 3.01
NG = 0
DB = 0
SM = 0
Delta_K
Cytarabine
YGR065C VHT1
ZShift = 0.39
NG = 0
DB = 0
SM = 0
Delta_r
Cytarabine
YGR065C VHT1
ZShift = 1.28
NG = 0
DB = 0
SM = 0
Delta_AUC
Cytarabine
YDR182W CDC1
ZShift = 2.87
NG = 0
DB = 0
SM = 0
Delta_L
Cytarabine
YDR182W CDC1
ZShift = 2.33
NG = 0
DB = 0
SM = 0
Delta_K
Cytarabine
YDR182W CDC1
ZShift = 0.6
NG = 0
DB = 0
SM = 0
Delta_r
Cytarabine
YDR182W CDC1
ZShift = 0.24
NG = 0
DB = 0
SM = 0
Delta_AUC
Cytarabine
YDR062W LCB2
ZShift = 1.33
NG = 0
DB = 0
SM = 0
Delta_L
Cytarabine
YDR062W LCB2
ZShift = 1.24
NG = 0
DB = 0
SM = 0
Delta_K
Cytarabine
YDR062W LCB2
ZShift = 1.27
NG = 0
DB = 0
SM = 0
Delta_r
Cytarabine
YDR062W LCB2
ZShift = 0.24
NG = 0
DB = 0
SM = 0
Delta_AUC
Cytarabine

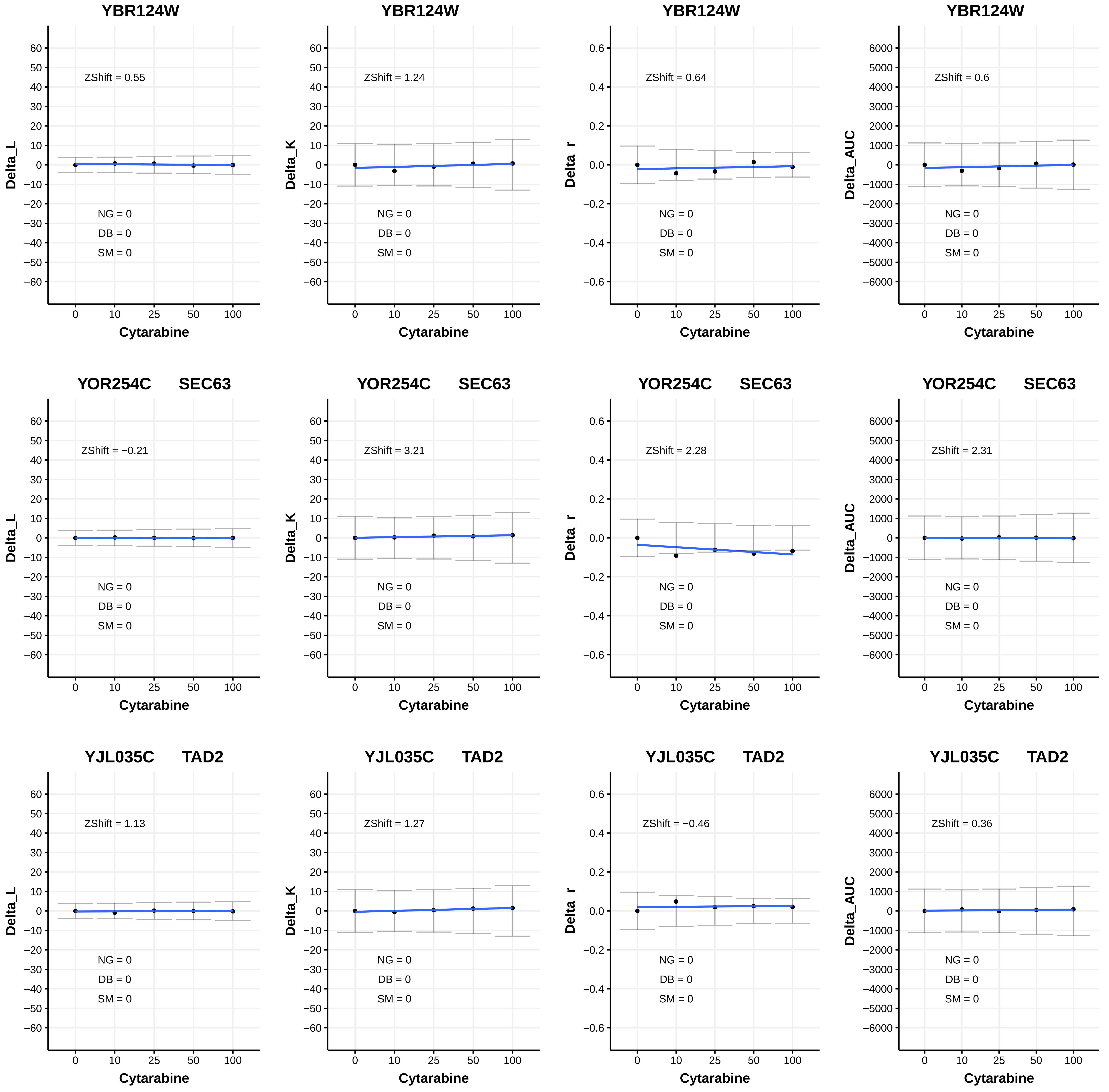
YBR124W
ZShift = 0.55
Delta_L
NG = 0
DB = 0
SM = 0
Cytarabine
YBR124W
ZShift = 1.24
Delta_K
NG = 0
DB = 0
SM = 0
Cytarabine
YBR124W
ZShift = 0.64
Delta_r
NG = 0
DB = 0
SM = 0
Cytarabine
YBR124W
ZShift = 0.6
Delta_AUC
NG = 0
DB = 0
SM = 0
Cytarabine
YOR254C SEC63
ZShift = −0.21
Delta_L
NG = 0
DB = 0
SM = 0
Cytarabine
YOR254C SEC63
ZShift = 3.21
Delta_K
NG = 0
DB = 0
SM = 0
Cytarabine
YOR254C SEC63
ZShift = 2.28
Delta_r
NG = 0
DB = 0
SM = 0
Cytarabine
YOR254C SEC63
ZShift = 2.31
Delta_AUC
NG = 0
DB = 0
SM = 0
Cytarabine
YJL035C TAD2
ZShift = 1.13
Delta_L
NG = 0
DB = 0
SM = 0
Cytarabine
YJL035C TAD2
ZShift = 1.27
Delta_K
NG = 0
DB = 0
SM = 0
Cytarabine
YJL035C TAD2
ZShift = −0.46
Delta_r
NG = 0
DB = 0
SM = 0
Cytarabine
YJL035C TAD2
ZShift = 0.36
Delta_AUC
NG = 0
DB = 0
SM = 0
Cytarabine

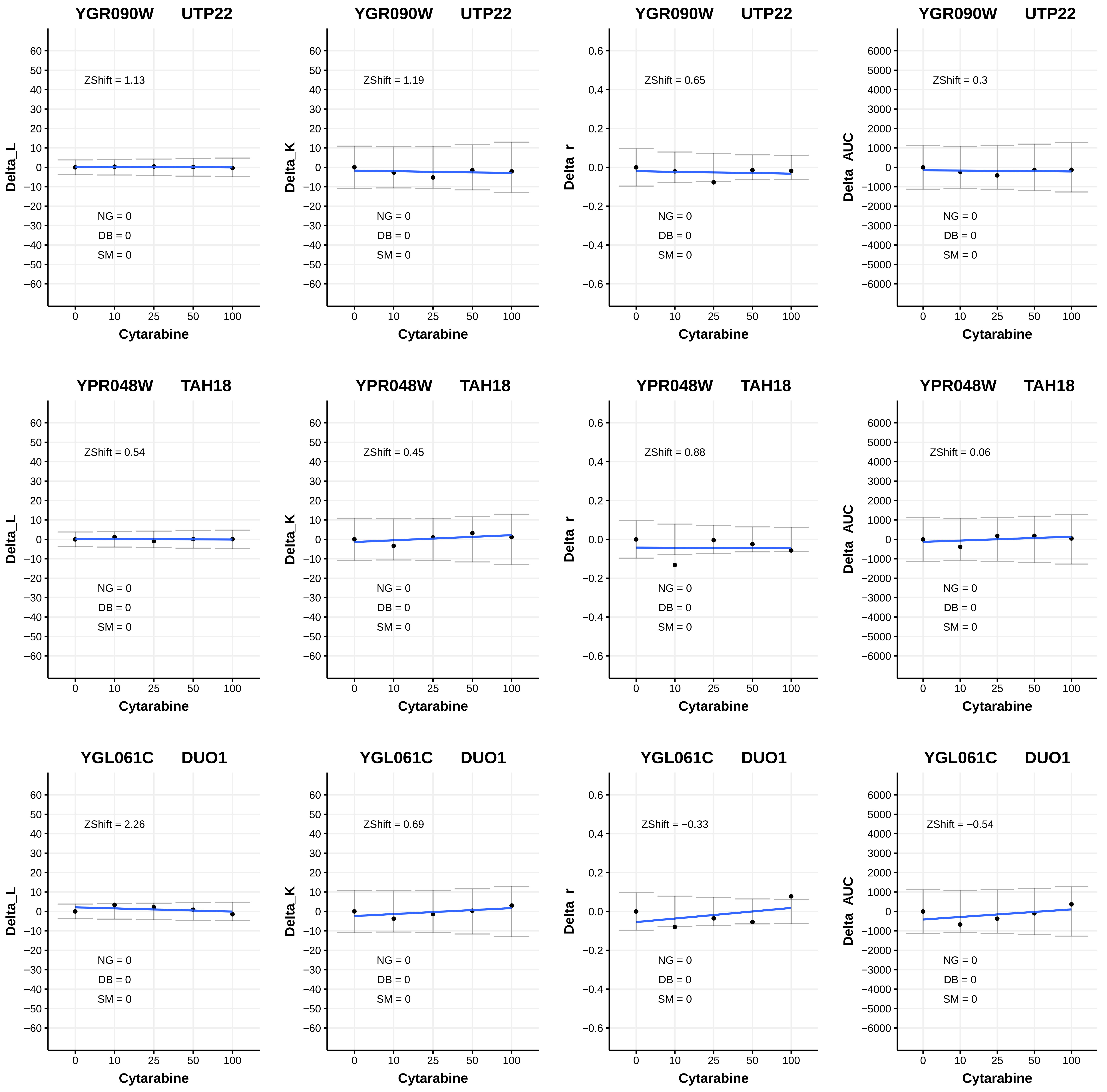
YGR090W UTP22
ZShift = 1.13
ZShift = 1.19
ZShift = 0.65
ZShift = 0.3
YPR048W TAH18
ZShift = 0.54
ZShift = 0.45
ZShift = 0.88
ZShift = 0.06
YGL061C DUO1
ZShift = 2.26
ZShift = 0.69
ZShift = −0.33
ZShift = −0.54
NG = 0
DB = 0
SM = 0
Delta_L
Delta_K
Delta_r
Delta_AUC
Cytarabine
0
10
25
50
100

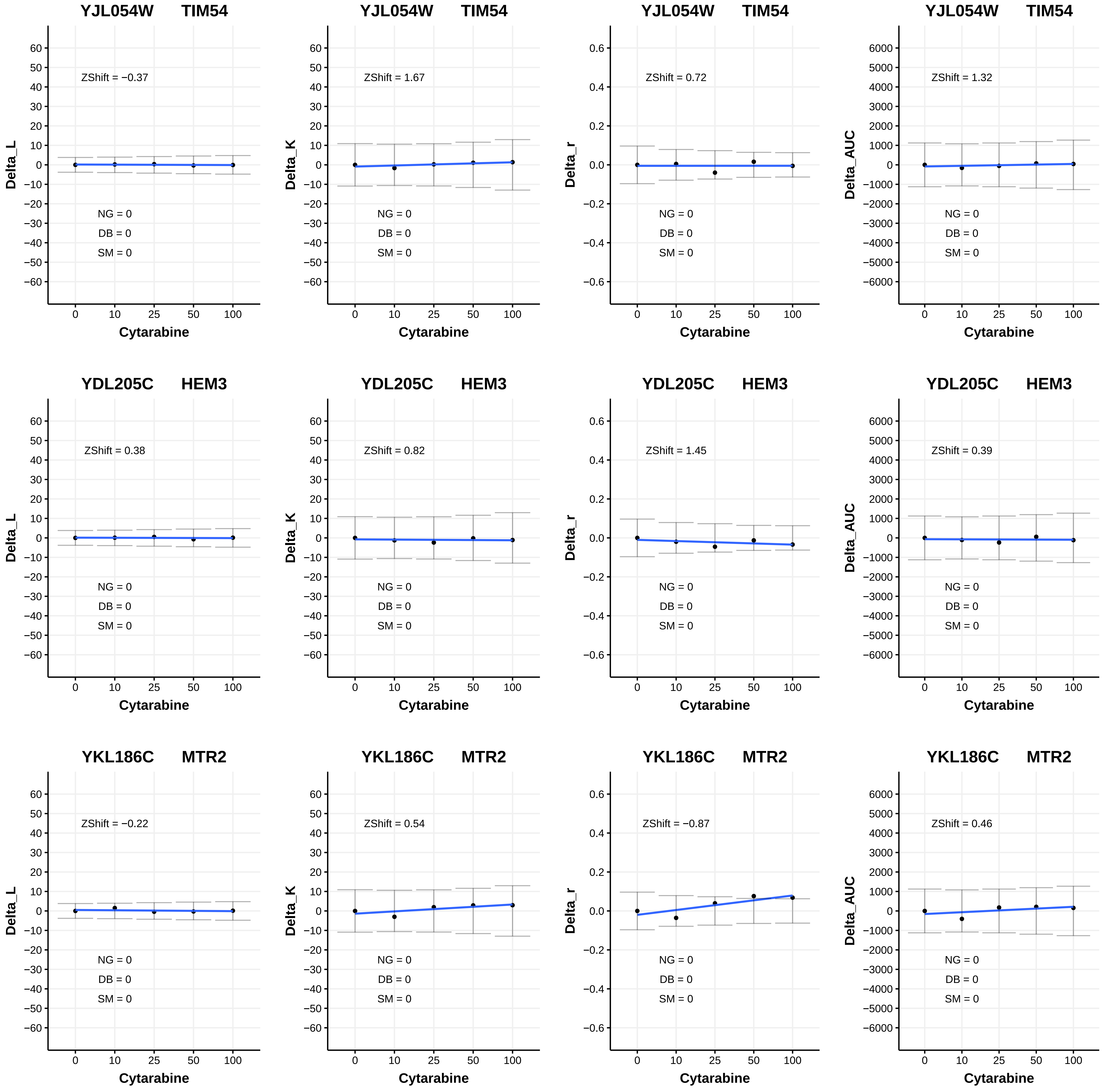
YJL054W TIM54
ZShift = −0.37
ZShift = 1.67
ZShift = 0.72
ZShift = 1.32
YDL205C HEM3
ZShift = 0.38
ZShift = 0.82
ZShift = 1.45
ZShift = 0.39
YKL186C MTR2
ZShift = −0.22
ZShift = 0.54
ZShift = −0.87
ZShift = 0.46
NG = 0
DB = 0
SM = 0
Delta_L
Delta_K
Delta_r
Delta_AUC
Cytarabine
0 10 25 50 100

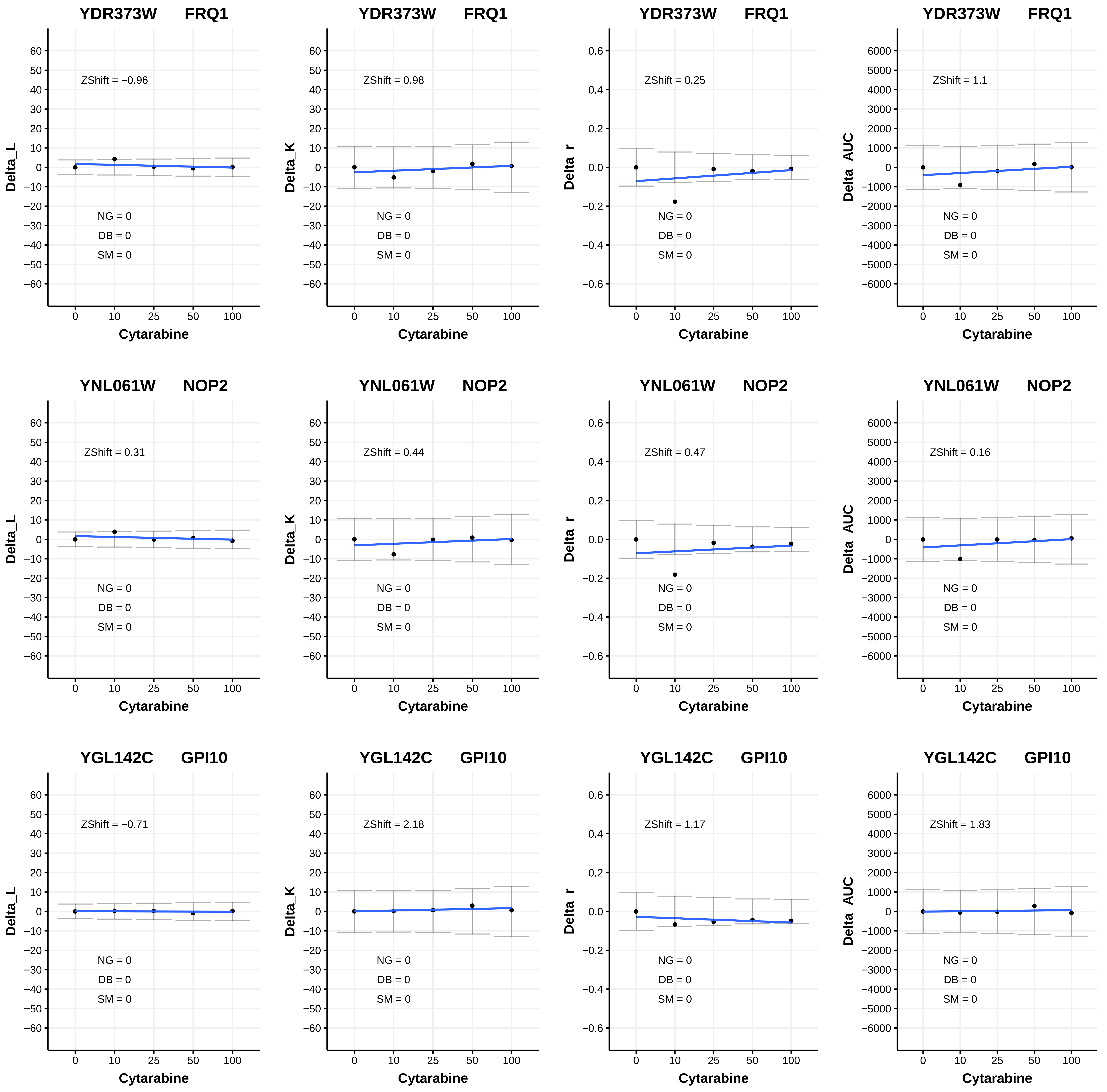
YDR373W FRQ1
ZShift = −0.96
NG = 0
DB = 0
SM = 0
Delta_L
Cytarabine
YDR373W FRQ1
ZShift = 0.98
NG = 0
DB = 0
SM = 0
Delta_K
Cytarabine
YDR373W FRQ1
ZShift = 0.25
NG = 0
DB = 0
SM = 0
Delta_r
Cytarabine
YDR373W FRQ1
ZShift = 1.1
NG = 0
DB = 0
SM = 0
Delta_AUC
Cytarabine
YNL061W NOP2
ZShift = 0.31
NG = 0
DB = 0
SM = 0
Delta_L
Cytarabine
YNL061W NOP2
ZShift = 0.44
NG = 0
DB = 0
SM = 0
Delta_K
Cytarabine
YNL061W NOP2
ZShift = 0.47
NG = 0
DB = 0
SM = 0
Delta_r
Cytarabine
YNL061W NOP2
ZShift = 0.16
NG = 0
DB = 0
SM = 0
Delta_AUC
Cytarabine
YGL142C GPI10
ZShift = −0.71
NG = 0
DB = 0
SM = 0
Delta_L
Cytarabine
YGL142C GPI10
ZShift = 2.18
NG = 0
DB = 0
SM = 0
Delta_K
Cytarabine
YGL142C GPI10
ZShift = 1.17
NG = 0
DB = 0
SM = 0
Delta_r
Cytarabine
YGL142C GPI10
ZShift = 1.83
NG = 0
DB = 0
SM = 0
Delta_AUC
Cytarabine

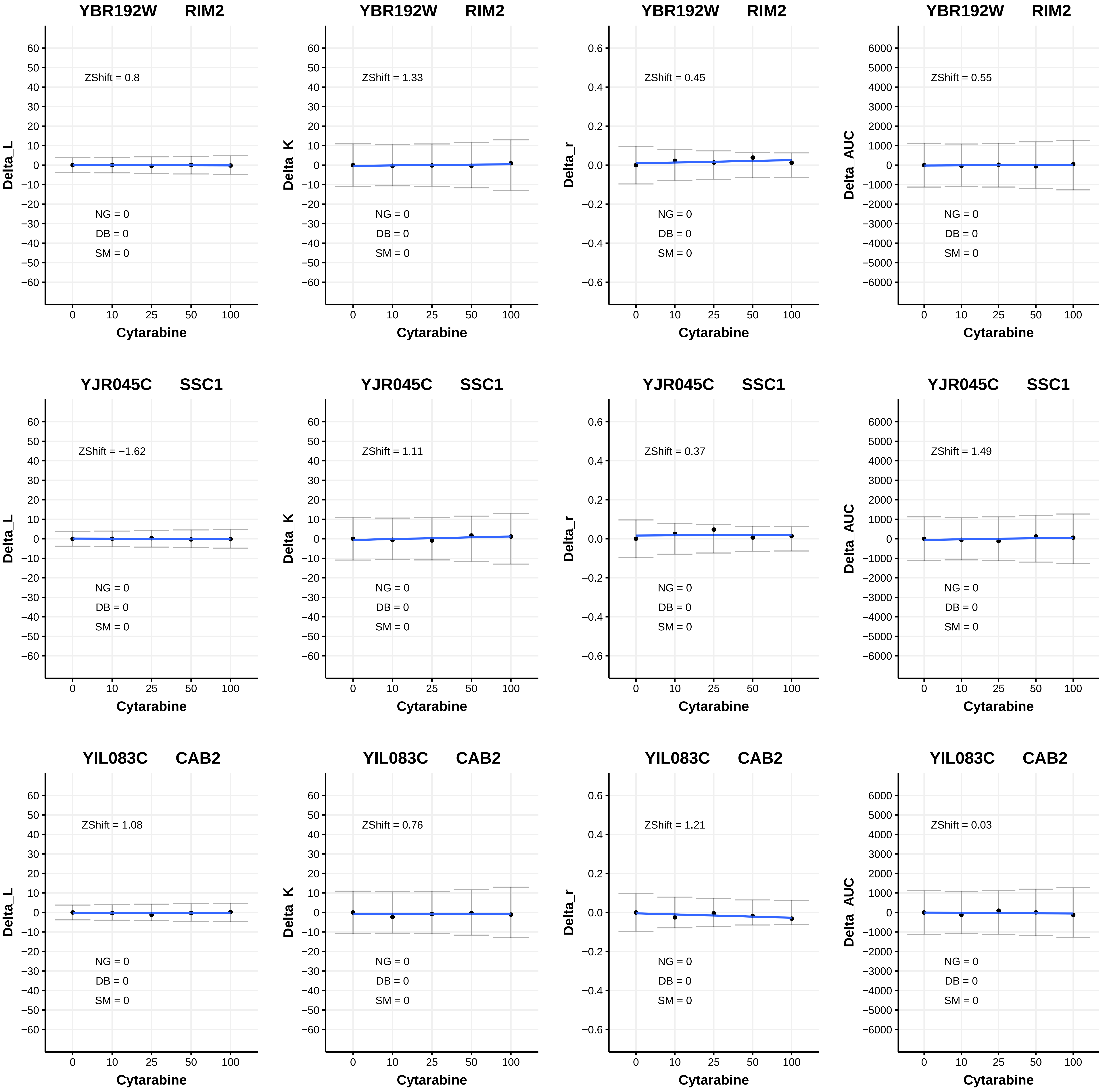
YBR192W RIM2
Delta_L
ZShift = 0.8
NG = 0
DB = 0
SM = 0
Cytarabine
YBR192W RIM2
Delta_K
ZShift = 1.33
NG = 0
DB = 0
SM = 0
Cytarabine
YBR192W RIM2
Delta_r
ZShift = 0.45
NG = 0
DB = 0
SM = 0
Cytarabine
YBR192W RIM2
Delta_AUC
ZShift = 0.55
NG = 0
DB = 0
SM = 0
Cytarabine
YJR045C SSC1
Delta_L
ZShift = −1.62
NG = 0
DB = 0
SM = 0
Cytarabine
YJR045C SSC1
Delta_K
ZShift = 1.11
NG = 0
DB = 0
SM = 0
Cytarabine
YJR045C SSC1
Delta_r
ZShift = 0.37
NG = 0
DB = 0
SM = 0
Cytarabine
YJR045C SSC1
Delta_AUC
ZShift = 1.49
NG = 0
DB = 0
SM = 0
Cytarabine
YIL083C CAB2
Delta_L
ZShift = 1.08
NG = 0
DB = 0
SM = 0
Cytarabine
YIL083C CAB2
Delta_K
ZShift = 0.76
NG = 0
DB = 0
SM = 0
Cytarabine
YIL083C CAB2
Delta_r
ZShift = 1.21
NG = 0
DB = 0
SM = 0
Cytarabine
YIL083C CAB2
Delta_AUC
ZShift = 0.03
NG = 0
DB = 0
SM = 0
Cytarabine

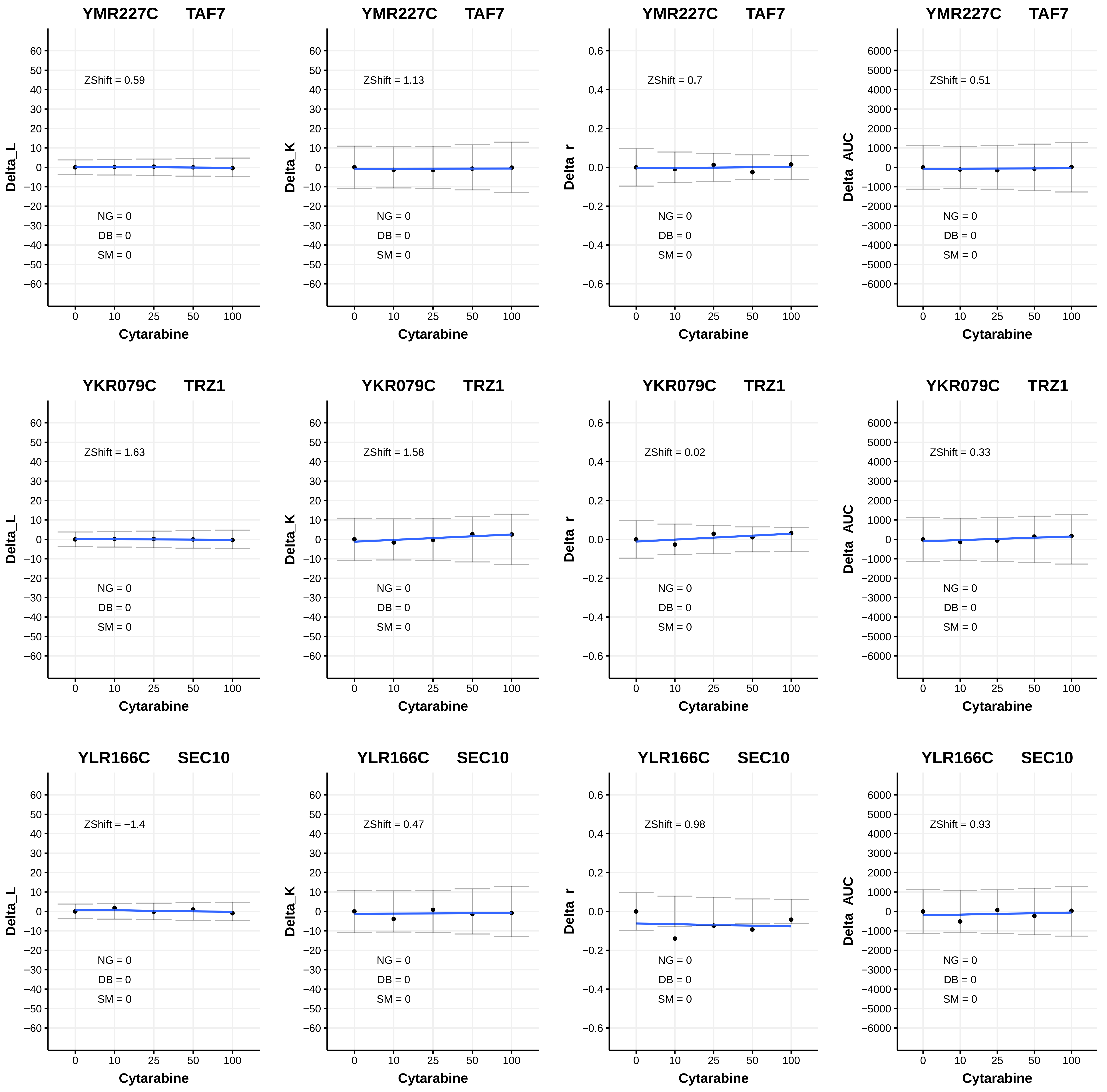

YMR227C TAF7
Delta_L
Cytarabine
ZShift = 0.59
NG = 0
DB = 0
SM = 0
YMR227C TAF7
Delta_K
Cytarabine
ZShift = 1.13
NG = 0
DB = 0
SM = 0
YMR227C TAF7
Delta_r
Cytarabine
ZShift = 0.7
NG = 0
DB = 0
SM = 0
YMR227C TAF7
Delta_AUC
Cytarabine
ZShift = 0.51
NG = 0
DB = 0
SM = 0
YKR079C TRZ1
Delta_L
Cytarabine
ZShift = 1.63
NG = 0
DB = 0
SM = 0
YKR079C TRZ1
Delta_K
Cytarabine
ZShift = 1.58
NG = 0
DB = 0
SM = 0
YKR079C TRZ1
Delta_r
Cytarabine
ZShift = 0.02
NG = 0
DB = 0
SM = 0
YKR079C TRZ1
Delta_AUC
Cytarabine
ZShift = 0.33
NG = 0
DB = 0
SM = 0
YLR166C SEC10
Delta_L
Cytarabine
ZShift = −1.4
NG = 0
DB = 0
SM = 0
YLR166C SEC10
Delta_K
Cytarabine
ZShift = 0.47
NG = 0
DB = 0
SM = 0
YLR166C SEC10
Delta_r
Cytarabine
ZShift = 0.98
NG = 0
DB = 0
SM = 0
YLR166C SEC10
Delta_AUC
Cytarabine
ZShift = 0.93
NG = 0
DB = 0
SM = 0
0 10 25 50 100

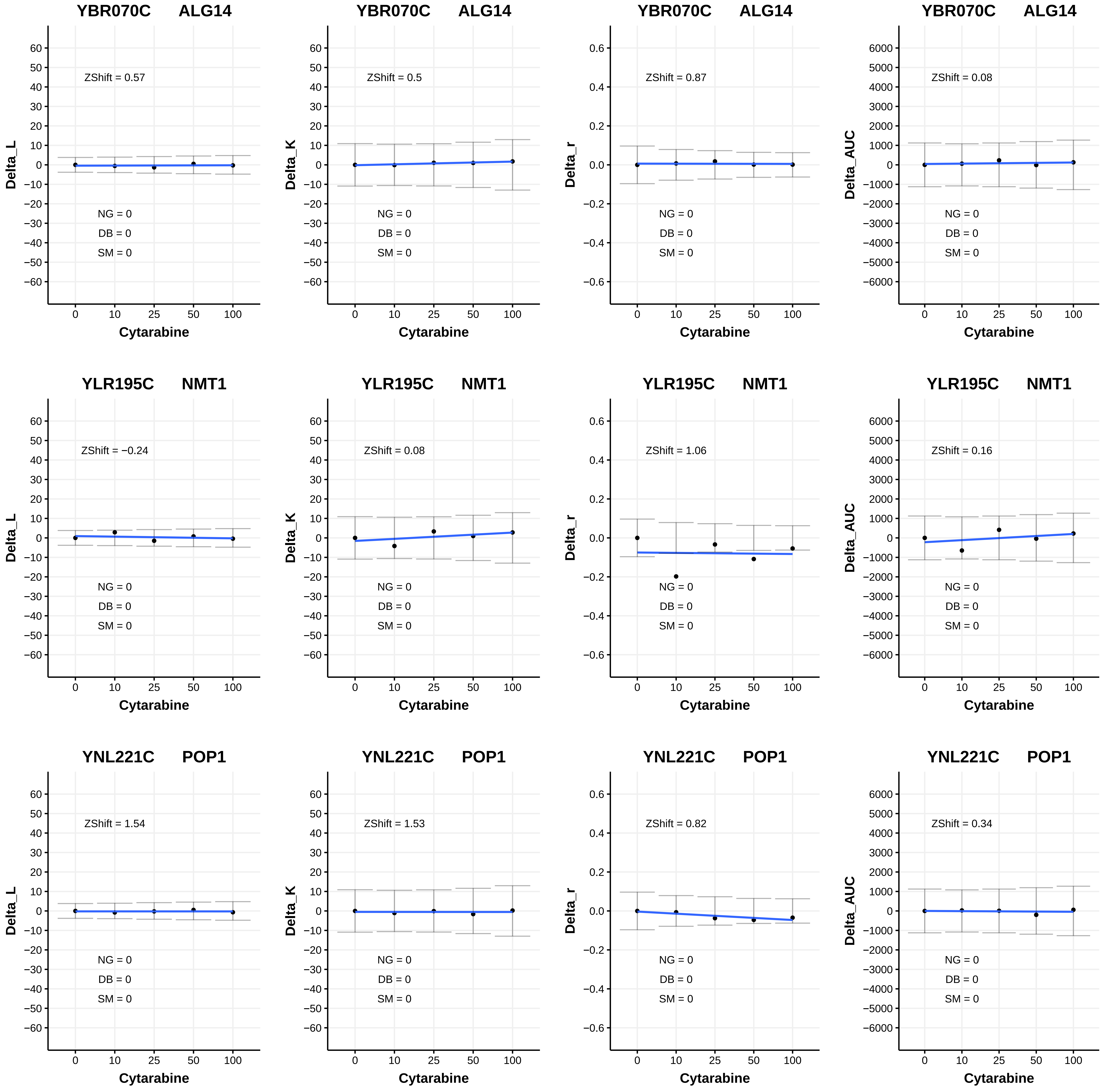

YBR070C ALG14
ZShift = 0.57
ZShift = 0.5
ZShift = 0.87
ZShift = 0.08
YLR195C NMT1
ZShift = −0.24
ZShift = 0.08
ZShift = 1.06
ZShift = 0.16
YNL221C POP1
ZShift = 1.54
ZShift = 1.53
ZShift = 0.82
ZShift = 0.34
NG = 0
DB = 0
SM = 0
Delta_L
Delta_K
Delta_r
Delta_AUC
Cytarabine
0 10 25 50 100

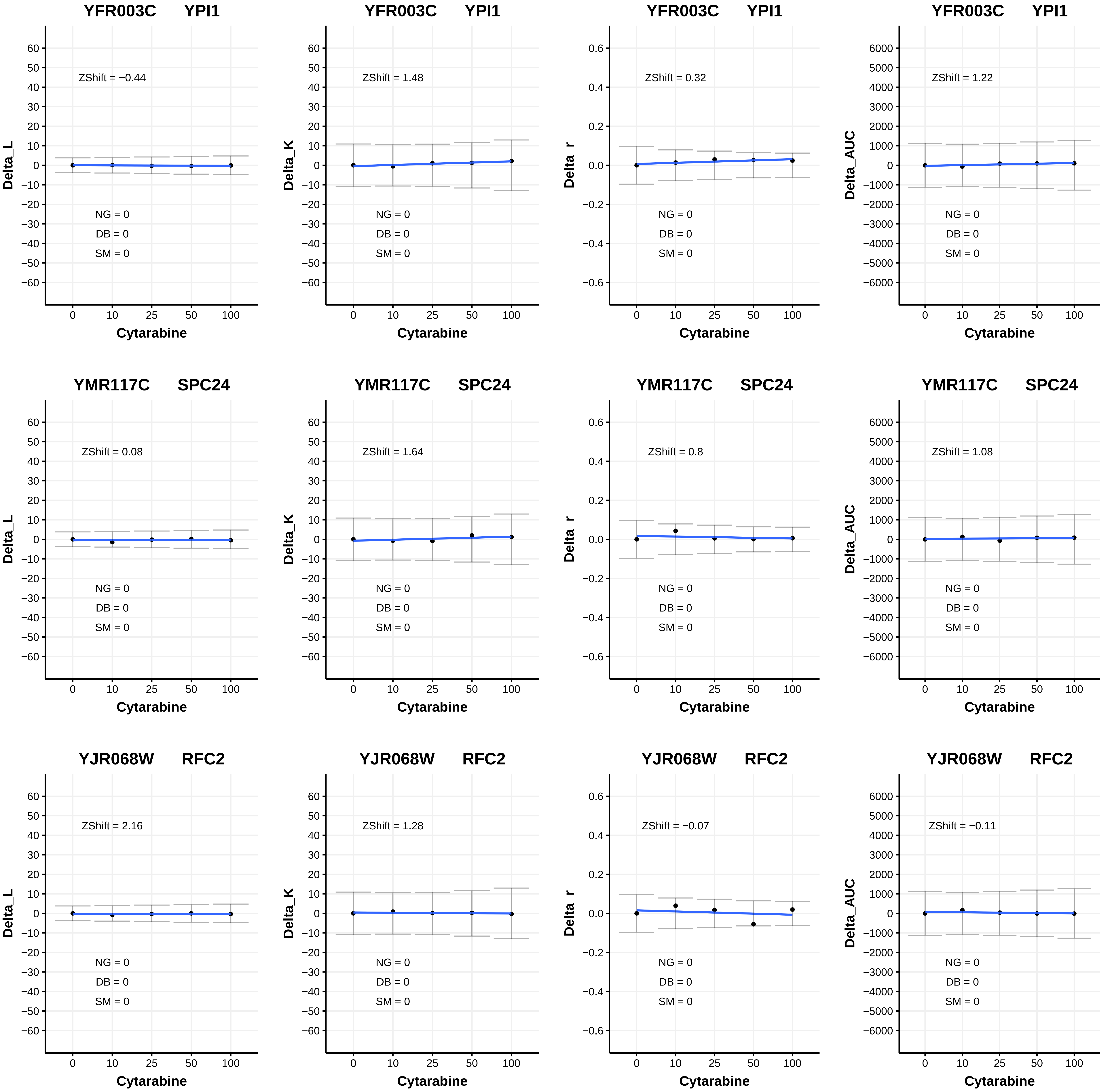

YFR003C YPI1
ZShift = −0.44
NG = 0
DB = 0
SM = 0
Delta_L
Cytarabine
YFR003C YPI1
ZShift = 1.48
NG = 0
DB = 0
SM = 0
Delta_K
Cytarabine
YFR003C YPI1
ZShift = 0.32
NG = 0
DB = 0
SM = 0
Delta_r
Cytarabine
YFR003C YPI1
ZShift = 1.22
NG = 0
DB = 0
SM = 0
Delta_AUC
Cytarabine
YMR117C SPC24
ZShift = 0.08
NG = 0
DB = 0
SM = 0
Delta_L
Cytarabine
YMR117C SPC24
ZShift = 1.64
NG = 0
DB = 0
SM = 0
Delta_K
Cytarabine
YMR117C SPC24
ZShift = 0.8
NG = 0
DB = 0
SM = 0
Delta_r
Cytarabine
YMR117C SPC24
ZShift = 1.08
NG = 0
DB = 0
SM = 0
Delta_AUC
Cytarabine
YJR068W RFC2
ZShift = 2.16
NG = 0
DB = 0
SM = 0
Delta_L
Cytarabine
YJR068W RFC2
ZShift = 1.28
NG = 0
DB = 0
SM = 0
Delta_K
Cytarabine
YJR068W RFC2
ZShift = −0.07
NG = 0
DB = 0
SM = 0
Delta_r
Cytarabine
YJR068W RFC2
ZShift = −0.11
NG = 0
DB = 0
SM = 0
Delta_AUC
Cytarabine

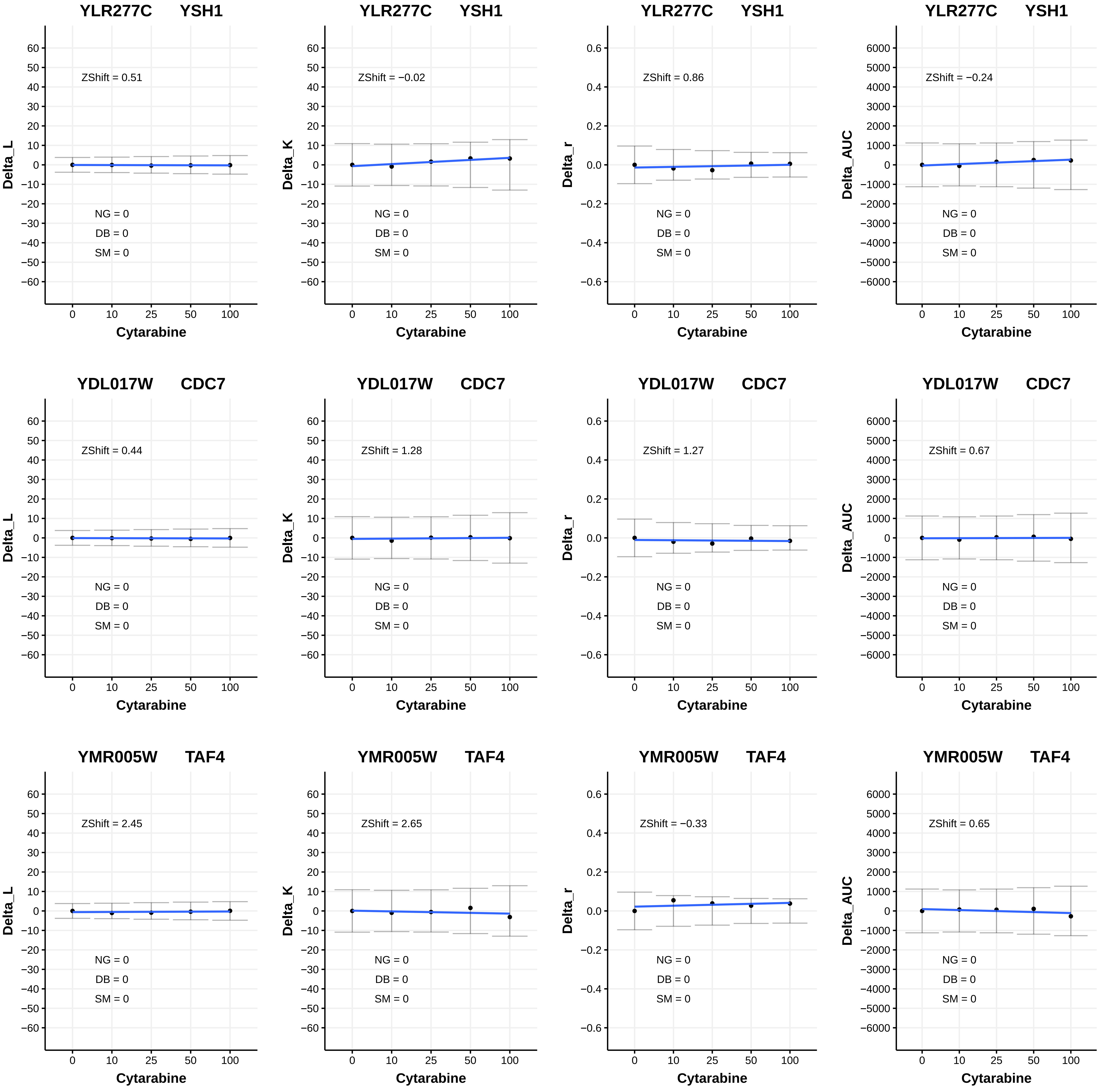
YLR277C YSH1
ZShift = 0.51
Delta_L
NG = 0
DB = 0
SM = 0
Cytarabine
YLR277C YSH1
ZShift = −0.02
Delta_K
NG = 0
DB = 0
SM = 0
Cytarabine
YLR277C YSH1
ZShift = 0.86
Delta_r
NG = 0
DB = 0
SM = 0
Cytarabine
YLR277C YSH1
ZShift = −0.24
Delta_AUC
NG = 0
DB = 0
SM = 0
Cytarabine
YDL017W CDC7
ZShift = 0.44
Delta_L
NG = 0
DB = 0
SM = 0
Cytarabine
YDL017W CDC7
ZShift = 1.28
Delta_K
NG = 0
DB = 0
SM = 0
Cytarabine
YDL017W CDC7
ZShift = 1.27
Delta_r
NG = 0
DB = 0
SM = 0
Cytarabine
YDL017W CDC7
ZShift = 0.67
Delta_AUC
NG = 0
DB = 0
SM = 0
Cytarabine
YMR005W TAF4
ZShift = 2.45
Delta_L
NG = 0
DB = 0
SM = 0
Cytarabine
YMR005W TAF4
ZShift = 2.65
Delta_K
NG = 0
DB = 0
SM = 0
Cytarabine
YMR005W TAF4
ZShift = −0.33
Delta_r
NG = 0
DB = 0
SM = 0
Cytarabine
YMR005W TAF4
ZShift = 0.65
Delta_AUC
NG = 0
DB = 0
SM = 0
Cytarabine

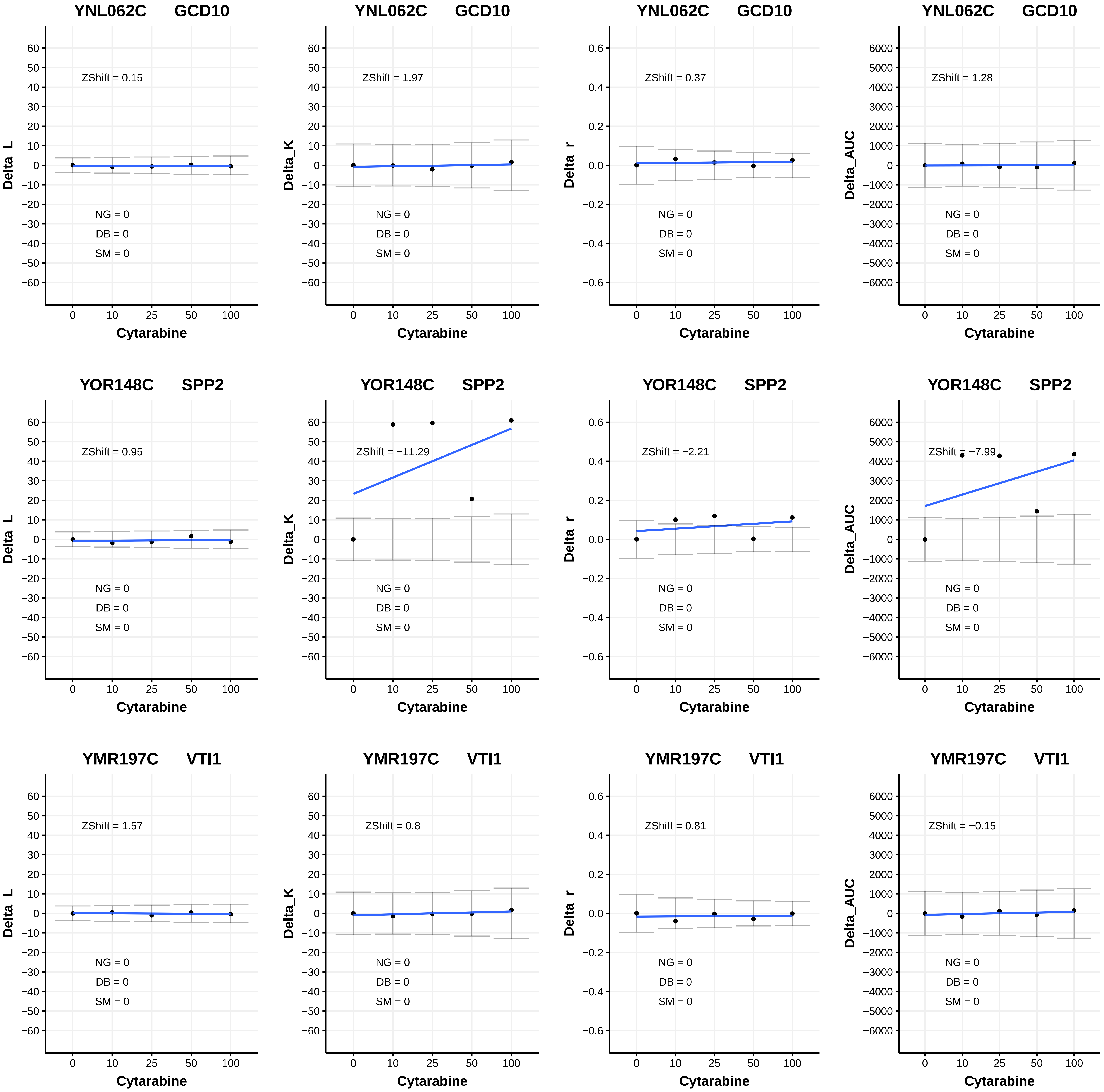
YNL062C GCD10
ZShift = 0.15
NG = 0
DB = 0
SM = 0
Delta_L
Cytarabine
YNL062C GCD10
ZShift = 1.97
NG = 0
DB = 0
SM = 0
Delta_K
Cytarabine
YNL062C GCD10
ZShift = 0.37
NG = 0
DB = 0
SM = 0
Delta_r
Cytarabine
YNL062C GCD10
ZShift = 1.28
NG = 0
DB = 0
SM = 0
Delta_AUC
Cytarabine
YOR148C SPP2
ZShift = 0.95
NG = 0
DB = 0
SM = 0
Delta_L
Cytarabine
YOR148C SPP2
ZShift = −11.29
NG = 0
DB = 0
SM = 0
Delta_K
Cytarabine
YOR148C SPP2
ZShift = −2.21
NG = 0
DB = 0
SM = 0
Delta_r
Cytarabine
YOR148C SPP2
ZShift = −7.99
NG = 0
DB = 0
SM = 0
Delta_AUC
Cytarabine
YMR197C VTI1
ZShift = 1.57
NG = 0
DB = 0
SM = 0
Delta_L
Cytarabine
YMR197C VTI1
ZShift = 0.8
NG = 0
DB = 0
SM = 0
Delta_K
Cytarabine
YMR197C VTI1
ZShift = 0.81
NG = 0
DB = 0
SM = 0
Delta_r
Cytarabine
YMR197C VTI1
ZShift = −0.15
NG = 0
DB = 0
SM = 0
Delta_AUC
Cytarabine

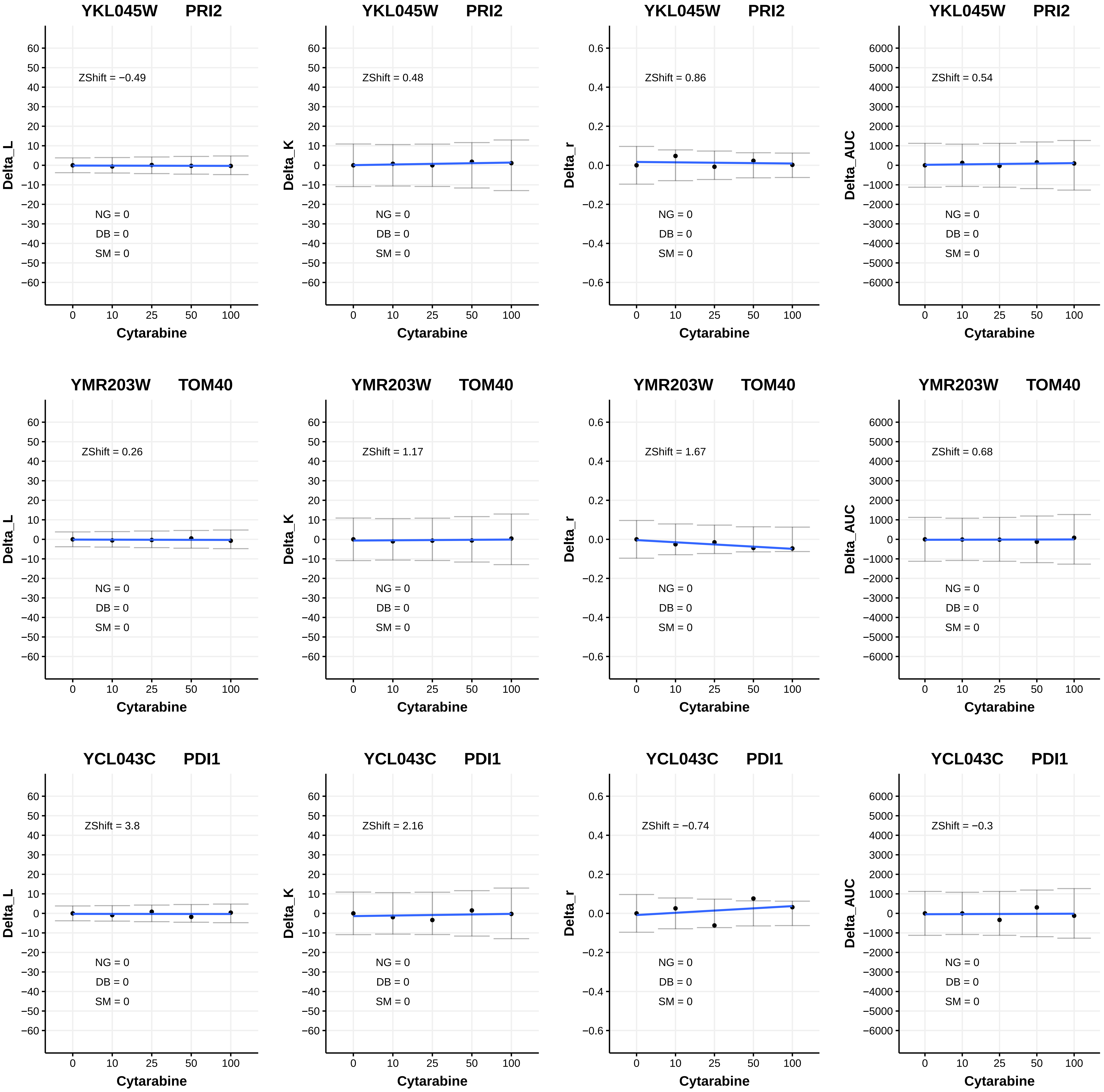

YKL045W PRI2
ZShift = −0.49
NG = 0
DB = 0
SM = 0
Delta_L
Cytarabine
YKL045W PRI2
ZShift = 0.48
NG = 0
DB = 0
SM = 0
Delta_K
Cytarabine
YKL045W PRI2
ZShift = 0.86
NG = 0
DB = 0
SM = 0
Delta_r
Cytarabine
YKL045W PRI2
ZShift = 0.54
NG = 0
DB = 0
SM = 0
Delta_AUC
Cytarabine
YMR203W TOM40
ZShift = 0.26
NG = 0
DB = 0
SM = 0
Delta_L
Cytarabine
YMR203W TOM40
ZShift = 1.17
NG = 0
DB = 0
SM = 0
Delta_K
Cytarabine
YMR203W TOM40
ZShift = 1.67
NG = 0
DB = 0
SM = 0
Delta_r
Cytarabine
YMR203W TOM40
ZShift = 0.68
NG = 0
DB = 0
SM = 0
Delta_AUC
Cytarabine
YCL043C PDI1
ZShift = 3.8
NG = 0
DB = 0
SM = 0
Delta_L
Cytarabine
YCL043C PDI1
ZShift = 2.16
NG = 0
DB = 0
SM = 0
Delta_K
Cytarabine
YCL043C PDI1
ZShift = −0.74
NG = 0
DB = 0
SM = 0
Delta_r
Cytarabine
YCL043C PDI1
ZShift = −0.3
NG = 0
DB = 0
SM = 0
Delta_AUC
Cytarabine

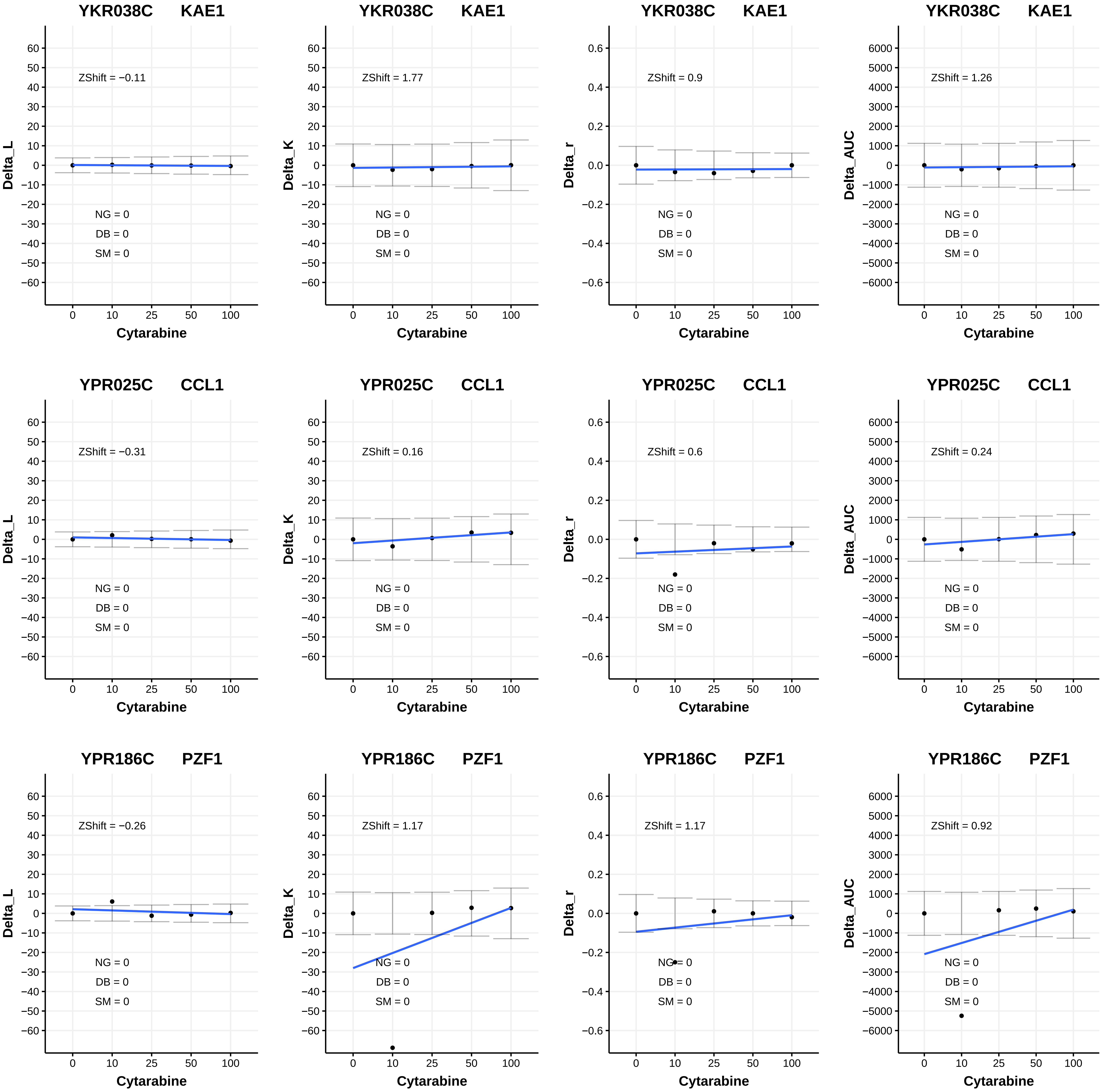
YKR038C KAE1
ZShift = −0.11
NG = 0
DB = 0
SM = 0
Delta_L
Cytarabine
YKR038C KAE1
ZShift = 1.77
NG = 0
DB = 0
SM = 0
Delta_K
Cytarabine
YKR038C KAE1
ZShift = 0.9
NG = 0
DB = 0
SM = 0
Delta_r
Cytarabine
YKR038C KAE1
ZShift = 1.26
NG = 0
DB = 0
SM = 0
Delta_AUC
Cytarabine
YPR025C CCL1
ZShift = −0.31
NG = 0
DB = 0
SM = 0
Delta_L
Cytarabine
YPR025C CCL1
ZShift = 0.16
NG = 0
DB = 0
SM = 0
Delta_K
Cytarabine
YPR025C CCL1
ZShift = 0.6
NG = 0
DB = 0
SM = 0
Delta_r
Cytarabine
YPR025C CCL1
ZShift = 0.24
NG = 0
DB = 0
SM = 0
Delta_AUC
Cytarabine
YPR186C PZF1
ZShift = −0.26
NG = 0
DB = 0
SM = 0
Delta_L
Cytarabine
YPR186C PZF1
ZShift = 1.17
NG = 0
DB = 0
SM = 0
Delta_K
Cytarabine
YPR186C PZF1
ZShift = 1.17
NG = 0
DB = 0
SM = 0
Delta_r
Cytarabine
YPR186C PZF1
ZShift = 0.92
NG = 0
DB = 0
SM = 0
Delta_AUC
Cytarabine

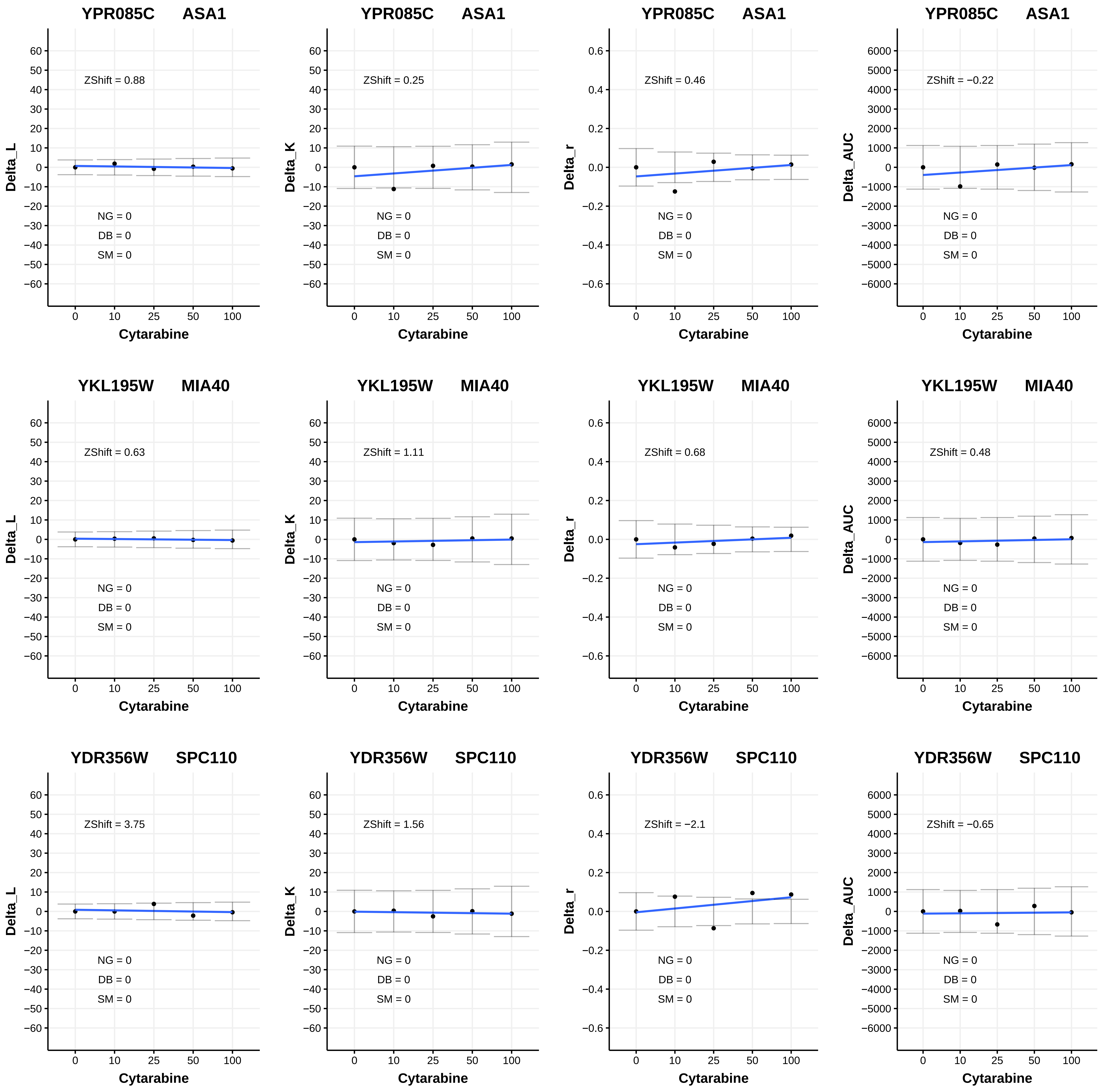
YPR085C ASA1
ZShift = 0.88
NG = 0
DB = 0
SM = 0
Delta_L
Cytarabine
YPR085C ASA1
ZShift = 0.25
NG = 0
DB = 0
SM = 0
Delta_K
Cytarabine
YPR085C ASA1
ZShift = 0.46
NG = 0
DB = 0
SM = 0
Delta_r
Cytarabine
YPR085C ASA1
ZShift = −0.22
NG = 0
DB = 0
SM = 0
Delta_AUC
Cytarabine
YKL195W MIA40
ZShift = 0.63
NG = 0
DB = 0
SM = 0
Delta_L
Cytarabine
YKL195W MIA40
ZShift = 1.11
NG = 0
DB = 0
SM = 0
Delta_K
Cytarabine
YKL195W MIA40
ZShift = 0.68
NG = 0
DB = 0
SM = 0
Delta_r
Cytarabine
YKL195W MIA40
ZShift = 0.48
NG = 0
DB = 0
SM = 0
Delta_AUC
Cytarabine
YDR356W SPC110
ZShift = 3.75
NG = 0
DB = 0
SM = 0
Delta_L
Cytarabine
YDR356W SPC110
ZShift = 1.56
NG = 0
DB = 0
SM = 0
Delta_K
Cytarabine
YDR356W SPC110
ZShift = −2.1
NG = 0
DB = 0
SM = 0
Delta_r
Cytarabine
YDR356W SPC110
ZShift = −0.65
NG = 0
DB = 0
SM = 0
Delta_AUC
Cytarabine
0 10 25 50 100

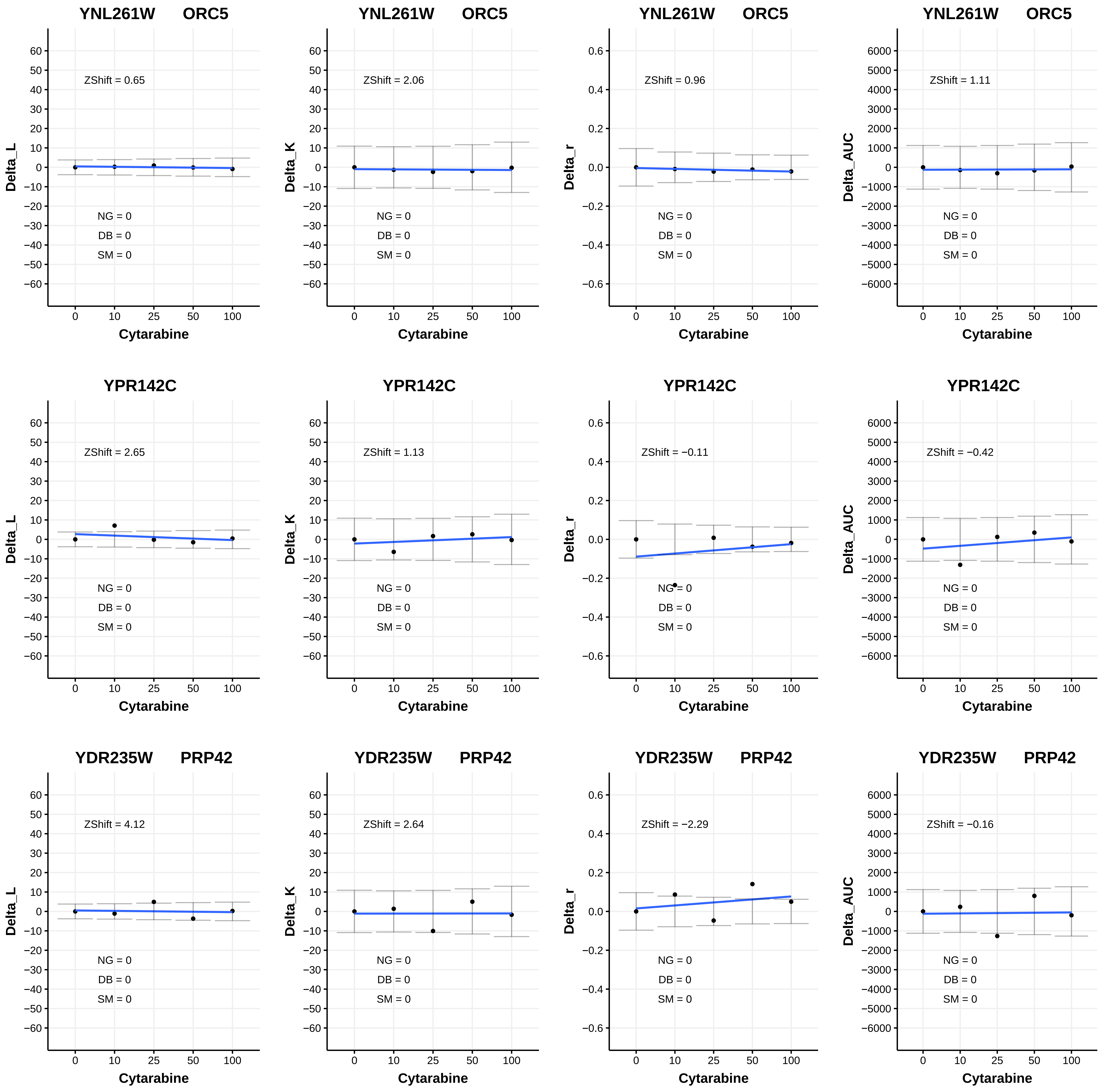
YNL261W ORC5
ZShift = 0.65
ZShift = 2.06
ZShift = 0.96
ZShift = 1.11
YPR142C
ZShift = 2.65
ZShift = 1.13
ZShift = −0.11
ZShift = −0.42
YDR235W PRP42
ZShift = 4.12
ZShift = 2.64
ZShift = −2.29
ZShift = −0.16
NG = 0
DB = 0
SM = 0
Delta_L
Delta_K
Delta_r
Delta_AUC
Cytarabine
0
10
25
50
100

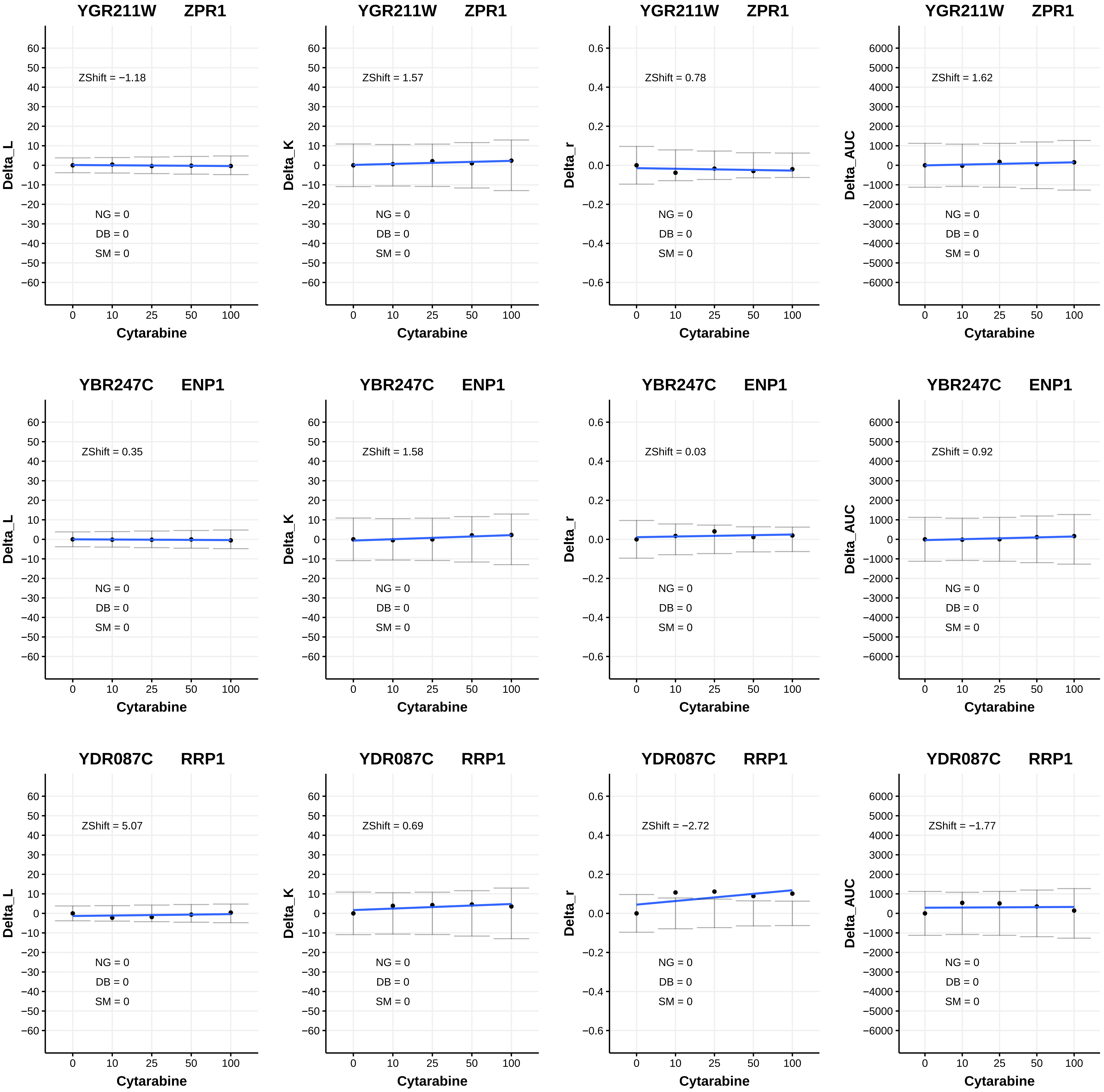
YGR211W ZPR1
ZShift = −1.18
NG = 0
DB = 0
SM = 0
Delta_L
Cytarabine
YGR211W ZPR1
ZShift = 1.57
NG = 0
DB = 0
SM = 0
Delta_K
Cytarabine
YGR211W ZPR1
ZShift = 0.78
NG = 0
DB = 0
SM = 0
Delta_r
Cytarabine
YGR211W ZPR1
ZShift = 1.62
NG = 0
DB = 0
SM = 0
Delta_AUC
Cytarabine
YBR247C ENP1
ZShift = 0.35
NG = 0
DB = 0
SM = 0
Delta_L
Cytarabine
YBR247C ENP1
ZShift = 1.58
NG = 0
DB = 0
SM = 0
Delta_K
Cytarabine
YBR247C ENP1
ZShift = 0.03
NG = 0
DB = 0
SM = 0
Delta_r
Cytarabine
YBR247C ENP1
ZShift = 0.92
NG = 0
DB = 0
SM = 0
Delta_AUC
Cytarabine
YDR087C RRP1
ZShift = 5.07
NG = 0
DB = 0
SM = 0
Delta_L
Cytarabine
YDR087C RRP1
ZShift = 0.69
NG = 0
DB = 0
SM = 0
Delta_K
Cytarabine
YDR087C RRP1
ZShift = −2.72
NG = 0
DB = 0
SM = 0
Delta_r
Cytarabine
YDR087C RRP1
ZShift = −1.77
NG = 0
DB = 0
SM = 0
Delta_AUC
Cytarabine

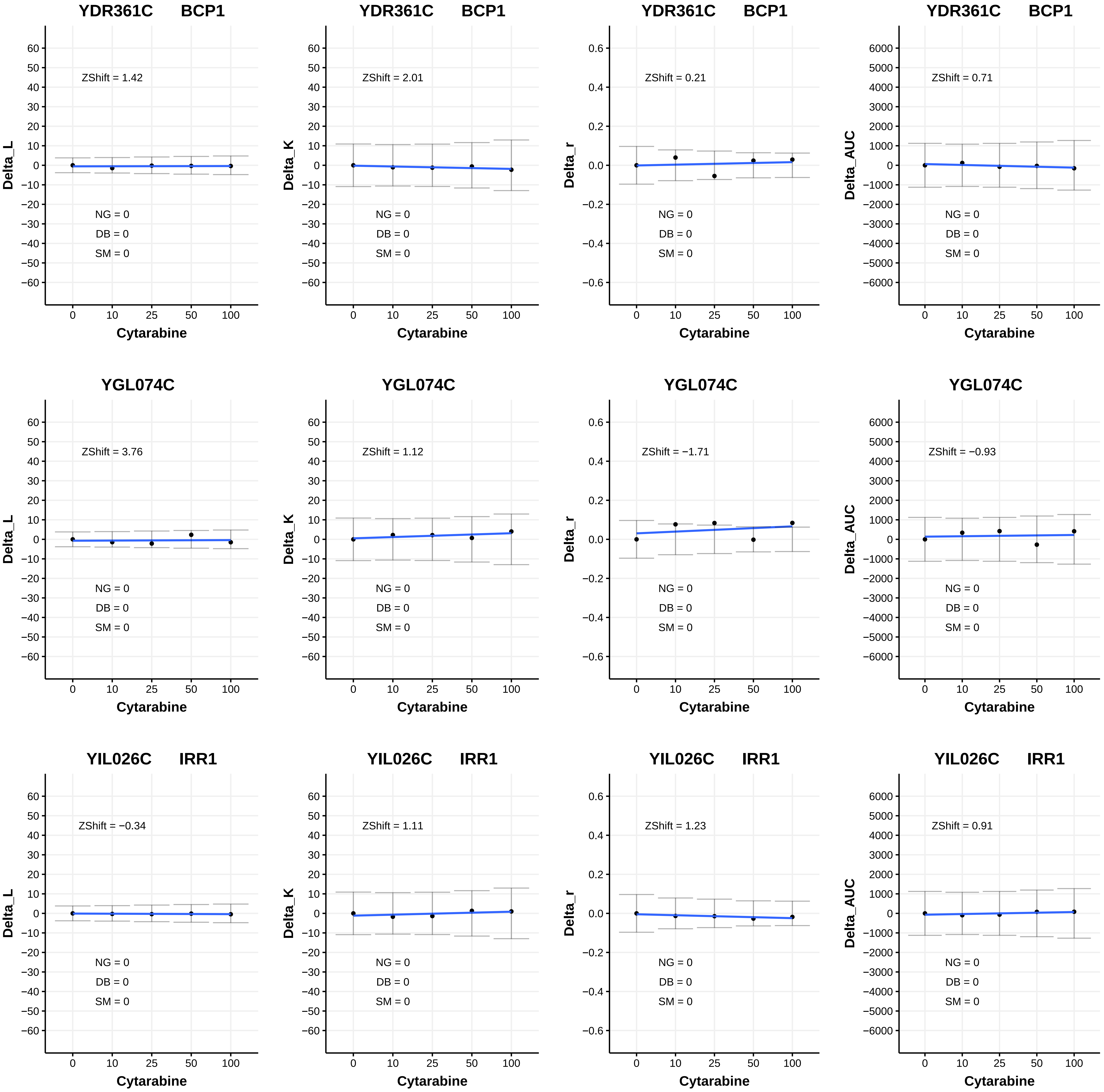
YDR361C BCP1
ZShift = 1.42
NG = 0
DB = 0
SM = 0
Delta_L
Cytarabine
YDR361C BCP1
ZShift = 2.01
NG = 0
DB = 0
SM = 0
Delta_K
Cytarabine
YDR361C BCP1
ZShift = 0.21
NG = 0
DB = 0
SM = 0
Delta_r
Cytarabine
YDR361C BCP1
ZShift = 0.71
NG = 0
DB = 0
SM = 0
Delta_AUC
Cytarabine
YGL074C
ZShift = 3.76
NG = 0
DB = 0
SM = 0
Delta_L
Cytarabine
YGL074C
ZShift = 1.12
NG = 0
DB = 0
SM = 0
Delta_K
Cytarabine
YGL074C
ZShift = −1.71
NG = 0
DB = 0
SM = 0
Delta_r
Cytarabine
YGL074C
ZShift = −0.93
NG = 0
DB = 0
SM = 0
Delta_AUC
Cytarabine
YIL026C IRR1
ZShift = −0.34
NG = 0
DB = 0
SM = 0
Delta_L
Cytarabine
YIL026C IRR1
ZShift = 1.11
NG = 0
DB = 0
SM = 0
Delta_K
Cytarabine
YIL026C IRR1
ZShift = 1.23
NG = 0
DB = 0
SM = 0
Delta_r
Cytarabine
YIL026C IRR1
ZShift = 0.91
NG = 0
DB = 0
SM = 0
Delta_AUC
Cytarabine

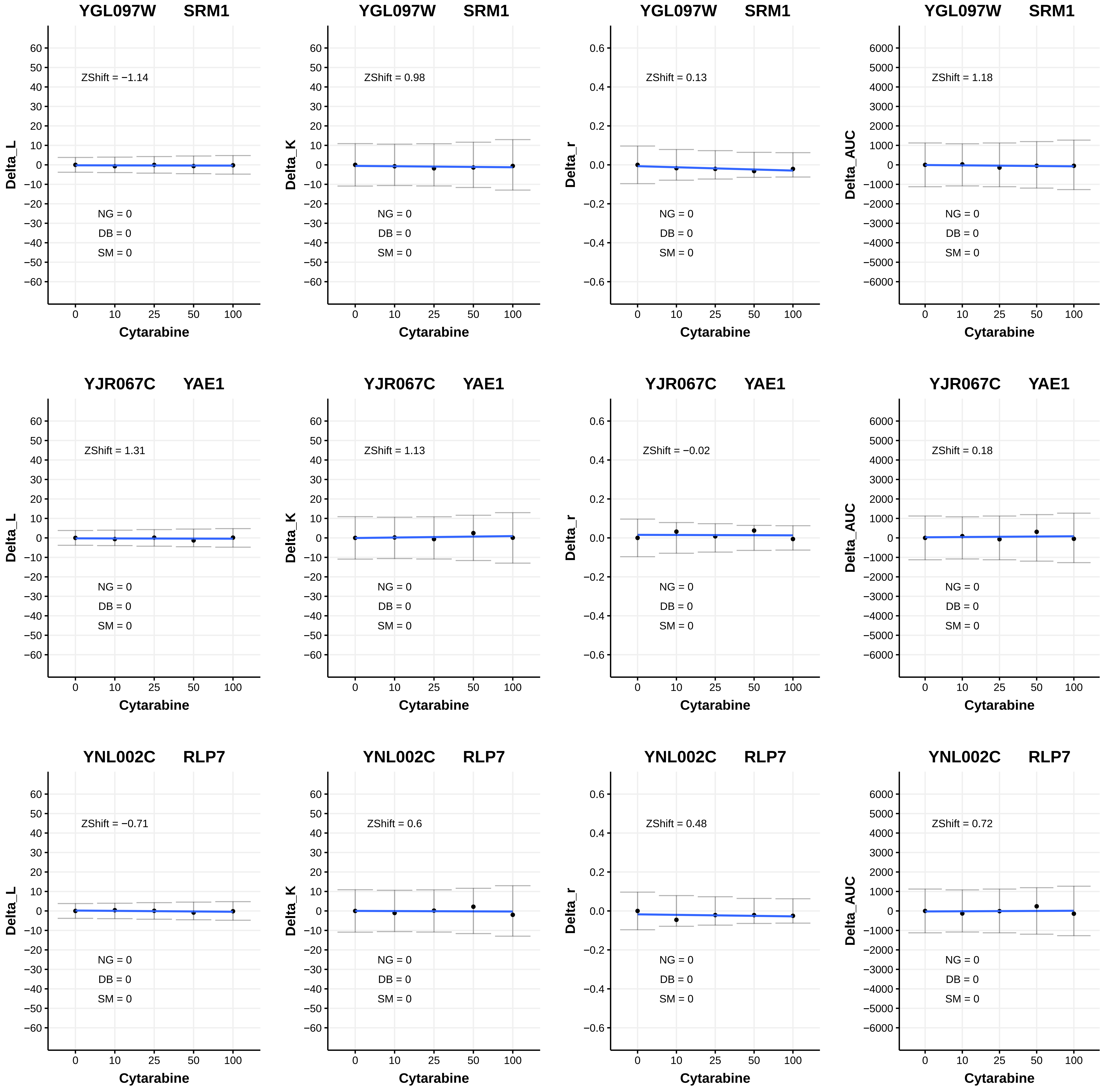
YGL097W SRM1
ZShift = −1.14
NG = 0
DB = 0
SM = 0
Delta_L
YGL097W SRM1
ZShift = 0.98
NG = 0
DB = 0
SM = 0
Delta_K
YGL097W SRM1
ZShift = 0.13
NG = 0
DB = 0
SM = 0
Delta_r
YGL097W SRM1
ZShift = 1.18
NG = 0
DB = 0
SM = 0
Delta_AUC
YJR067C YAE1
ZShift = 1.31
NG = 0
DB = 0
SM = 0
Delta_L
YJR067C YAE1
ZShift = 1.13
NG = 0
DB = 0
SM = 0
Delta_K
YJR067C YAE1
ZShift = −0.02
NG = 0
DB = 0
SM = 0
Delta_r
YJR067C YAE1
ZShift = 0.18
NG = 0
DB = 0
SM = 0
Delta_AUC
YNL002C RLP7
ZShift = −0.71
NG = 0
DB = 0
SM = 0
Delta_L
YNL002C RLP7
ZShift = 0.6
NG = 0
DB = 0
SM = 0
Delta_K
YNL002C RLP7
ZShift = 0.48
NG = 0
DB = 0
SM = 0
Delta_r
YNL002C RLP7
ZShift = 0.72
NG = 0
DB = 0
SM = 0
Delta_AUC
Cytarabine
0
10
25
50
100

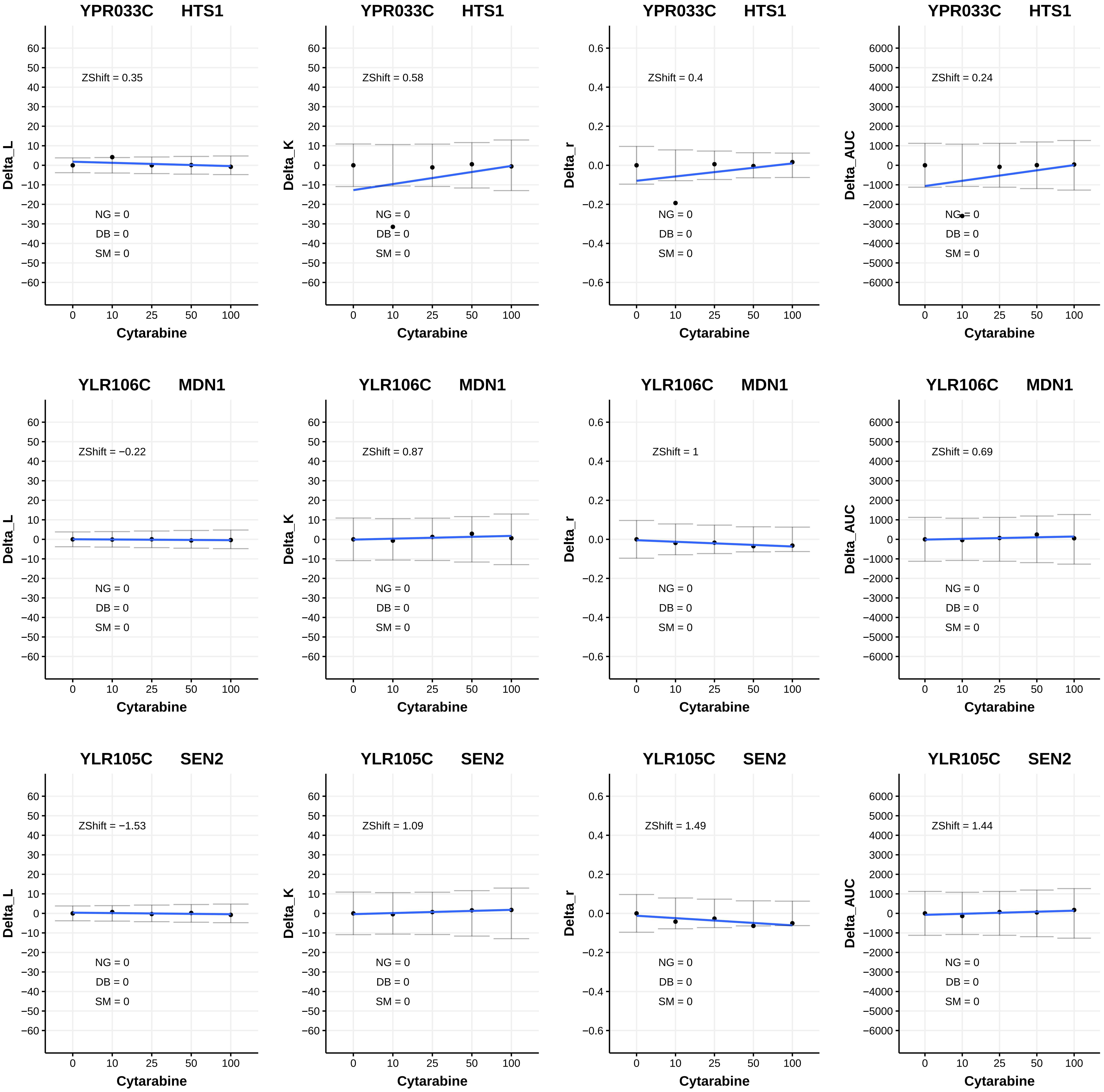

YPR033C HTS1
ZShift = 0.35
NG = 0
DB = 0
SM = 0
Delta_L
Cytarabine
YPR033C HTS1
ZShift = 0.58
NG = 0
DB = 0
SM = 0
Delta_K
Cytarabine
YPR033C HTS1
ZShift = 0.4
NG = 0
DB = 0
SM = 0
Delta_r
Cytarabine
YPR033C HTS1
ZShift = 0.24
NG = 0
DB = 0
SM = 0
Delta_AUC
Cytarabine
YLR106C MDN1
ZShift = −0.22
NG = 0
DB = 0
SM = 0
Delta_L
Cytarabine
YLR106C MDN1
ZShift = 0.87
NG = 0
DB = 0
SM = 0
Delta_K
Cytarabine
YLR106C MDN1
ZShift = 1
NG = 0
DB = 0
SM = 0
Delta_r
Cytarabine
YLR106C MDN1
ZShift = 0.69
NG = 0
DB = 0
SM = 0
Delta_AUC
Cytarabine
YLR105C SEN2
ZShift = −1.53
NG = 0
DB = 0
SM = 0
Delta_L
Cytarabine
YLR105C SEN2
ZShift = 1.09
NG = 0
DB = 0
SM = 0
Delta_K
Cytarabine
YLR105C SEN2
ZShift = 1.49
NG = 0
DB = 0
SM = 0
Delta_r
Cytarabine
YLR105C SEN2
ZShift = 1.44
NG = 0
DB = 0
SM = 0
Delta_AUC
Cytarabine

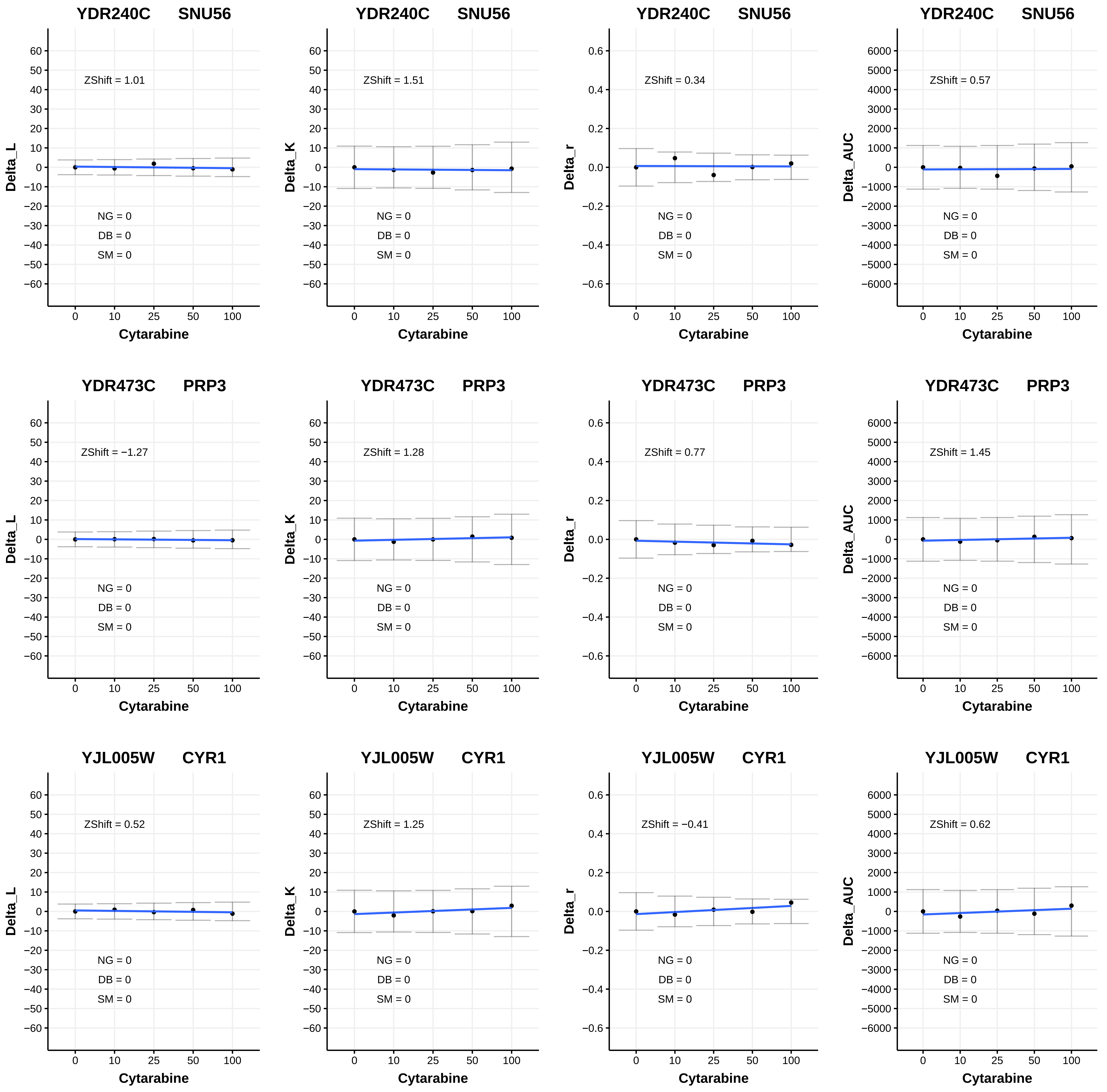
YDR240C SNU56
ZShift = 1.01
NG = 0
DB = 0
SM = 0
Delta_L
Cytarabine
YDR240C SNU56
ZShift = 1.51
NG = 0
DB = 0
SM = 0
Delta_K
Cytarabine
YDR240C SNU56
ZShift = 0.34
NG = 0
DB = 0
SM = 0
Delta_r
Cytarabine
YDR240C SNU56
ZShift = 0.57
NG = 0
DB = 0
SM = 0
Delta_AUC
Cytarabine
YDR473C PRP3
ZShift = −1.27
NG = 0
DB = 0
SM = 0
Delta_L
Cytarabine
YDR473C PRP3
ZShift = 1.28
NG = 0
DB = 0
SM = 0
Delta_K
Cytarabine
YDR473C PRP3
ZShift = 0.77
NG = 0
DB = 0
SM = 0
Delta_r
Cytarabine
YDR473C PRP3
ZShift = 1.45
NG = 0
DB = 0
SM = 0
Delta_AUC
Cytarabine
YJL005W CYR1
ZShift = 0.52
NG = 0
DB = 0
SM = 0
Delta_L
Cytarabine
YJL005W CYR1
ZShift = 1.25
NG = 0
DB = 0
SM = 0
Delta_K
Cytarabine
YJL005W CYR1
ZShift = −0.41
NG = 0
DB = 0
SM = 0
Delta_r
Cytarabine
YJL005W CYR1
ZShift = 0.62
NG = 0
DB = 0
SM = 0
Delta_AUC
Cytarabine
0 10 25 50 100

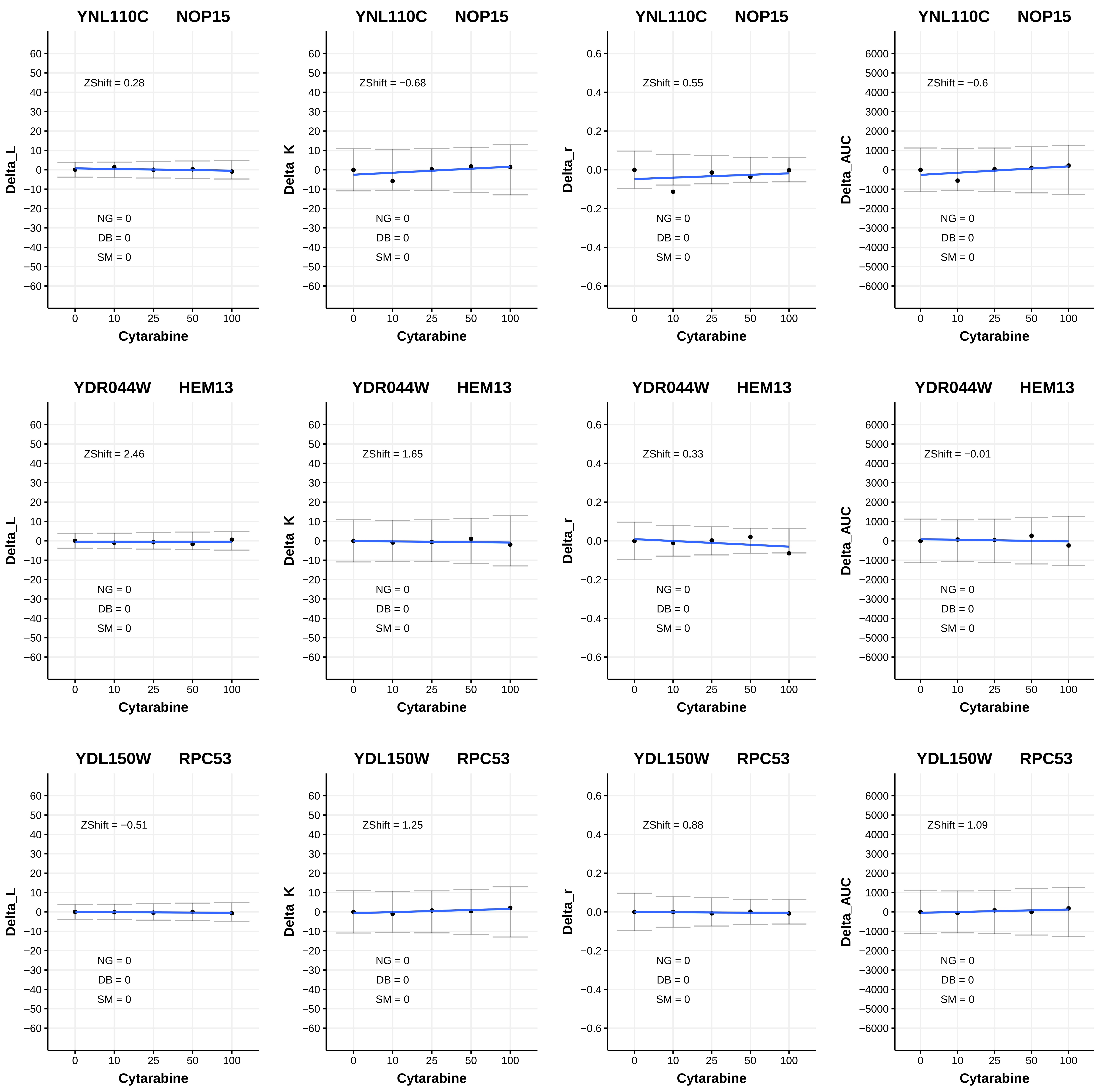

YNL110C NOP15
ZShift = 0.28
NG = 0
DB = 0
SM = 0
Delta_L
Cytarabine
YNL110C NOP15
ZShift = −0.68
NG = 0
DB = 0
SM = 0
Delta_K
Cytarabine
YNL110C NOP15
ZShift = 0.55
NG = 0
DB = 0
SM = 0
Delta_r
Cytarabine
YNL110C NOP15
ZShift = −0.6
NG = 0
DB = 0
SM = 0
Delta_AUC
Cytarabine
YDR044W HEM13
ZShift = 2.46
NG = 0
DB = 0
SM = 0
Delta_L
Cytarabine
YDR044W HEM13
ZShift = 1.65
NG = 0
DB = 0
SM = 0
Delta_K
Cytarabine
YDR044W HEM13
ZShift = 0.33
NG = 0
DB = 0
SM = 0
Delta_r
Cytarabine
YDR044W HEM13
ZShift = −0.01
NG = 0
DB = 0
SM = 0
Delta_AUC
Cytarabine
YDL150W RPC53
ZShift = −0.51
NG = 0
DB = 0
SM = 0
Delta_L
Cytarabine
YDL150W RPC53
ZShift = 1.25
NG = 0
DB = 0
SM = 0
Delta_K
Cytarabine
YDL150W RPC53
ZShift = 0.88
NG = 0
DB = 0
SM = 0
Delta_r
Cytarabine
YDL150W RPC53
ZShift = 1.09
NG = 0
DB = 0
SM = 0
Delta_AUC
Cytarabine

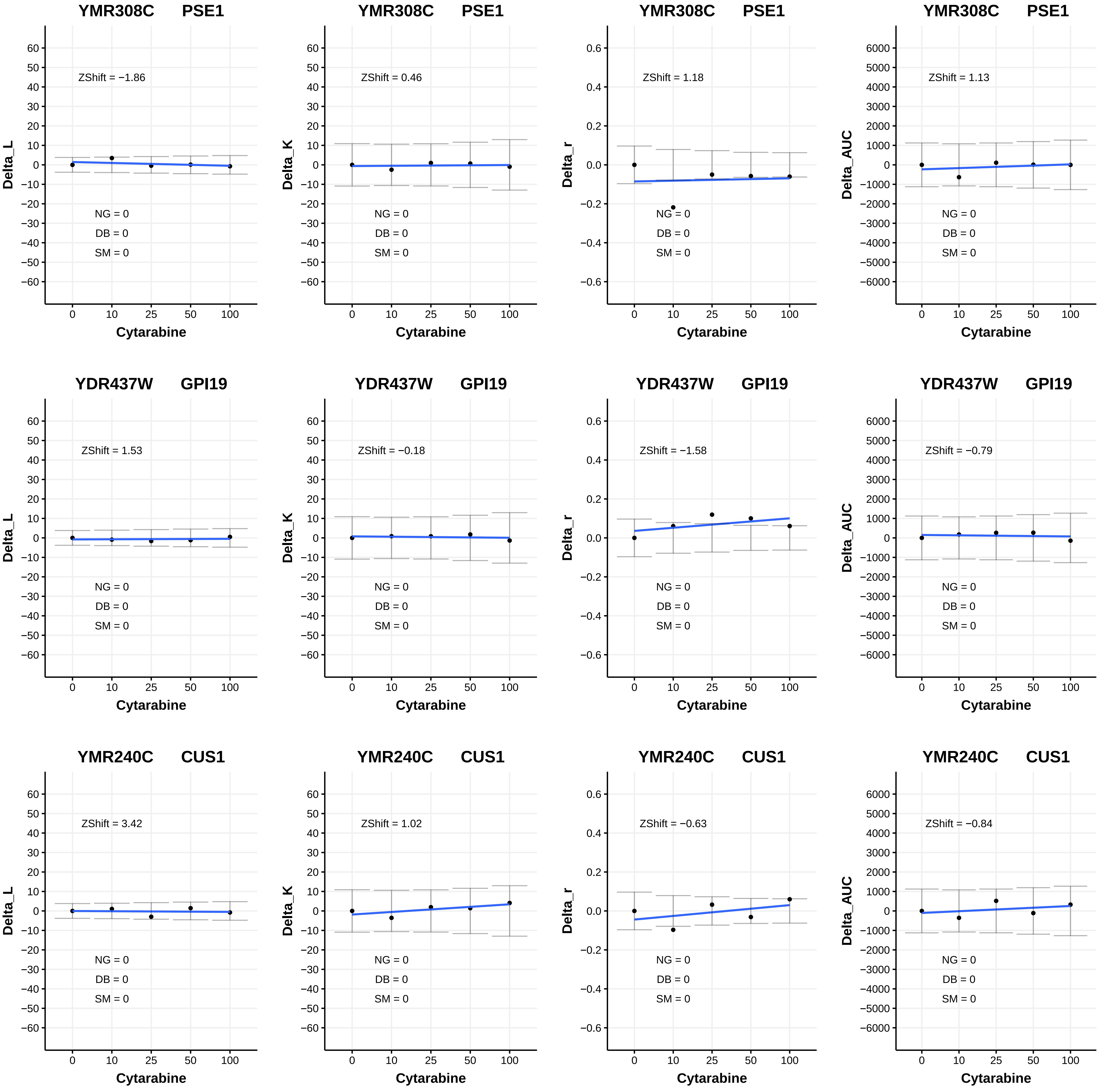
YMR308C PSE1
ZShift = −1.86
ZShift = 0.46
ZShift = 1.18
ZShift = 1.13
YDR437W GPI19
ZShift = 1.53
ZShift = −0.18
ZShift = −1.58
ZShift = −0.79
YMR240C CUS1
ZShift = 3.42
ZShift = 1.02
ZShift = −0.63
ZShift = −0.84
NG = 0
DB = 0
SM = 0
Delta_L
Delta_K
Delta_r
Delta_AUC
Cytarabine
0
10
25
50
100

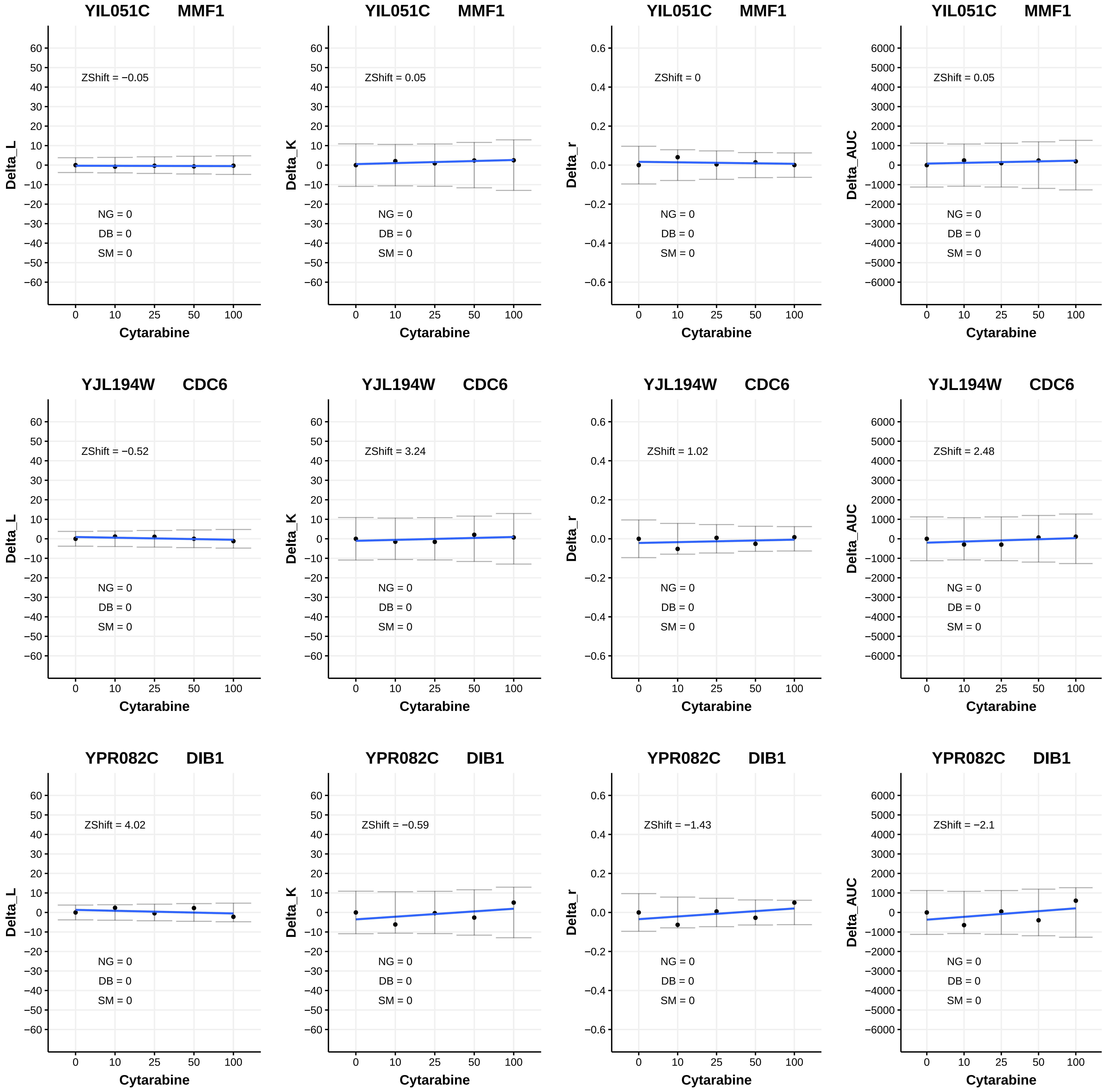
YIL051C MMF1
ZShift = −0.05
NG = 0
DB = 0
SM = 0
Delta_L
Cytarabine
YIL051C MMF1
ZShift = 0.05
NG = 0
DB = 0
SM = 0
Delta_K
Cytarabine
YIL051C MMF1
ZShift = 0
NG = 0
DB = 0
SM = 0
Delta_r
Cytarabine
YIL051C MMF1
ZShift = 0.05
NG = 0
DB = 0
SM = 0
Delta_AUC
Cytarabine
YJL194W CDC6
ZShift = −0.52
NG = 0
DB = 0
SM = 0
Delta_L
Cytarabine
YJL194W CDC6
ZShift = 3.24
NG = 0
DB = 0
SM = 0
Delta_K
Cytarabine
YJL194W CDC6
ZShift = 1.02
NG = 0
DB = 0
SM = 0
Delta_r
Cytarabine
YJL194W CDC6
ZShift = 2.48
NG = 0
DB = 0
SM = 0
Delta_AUC
Cytarabine
YPR082C DIB1
ZShift = 4.02
NG = 0
DB = 0
SM = 0
Delta_L
Cytarabine
YPR082C DIB1
ZShift = −0.59
NG = 0
DB = 0
SM = 0
Delta_K
Cytarabine
YPR082C DIB1
ZShift = −1.43
NG = 0
DB = 0
SM = 0
Delta_r
Cytarabine
YPR082C DIB1
ZShift = −2.1
NG = 0
DB = 0
SM = 0
Delta_AUC
Cytarabine

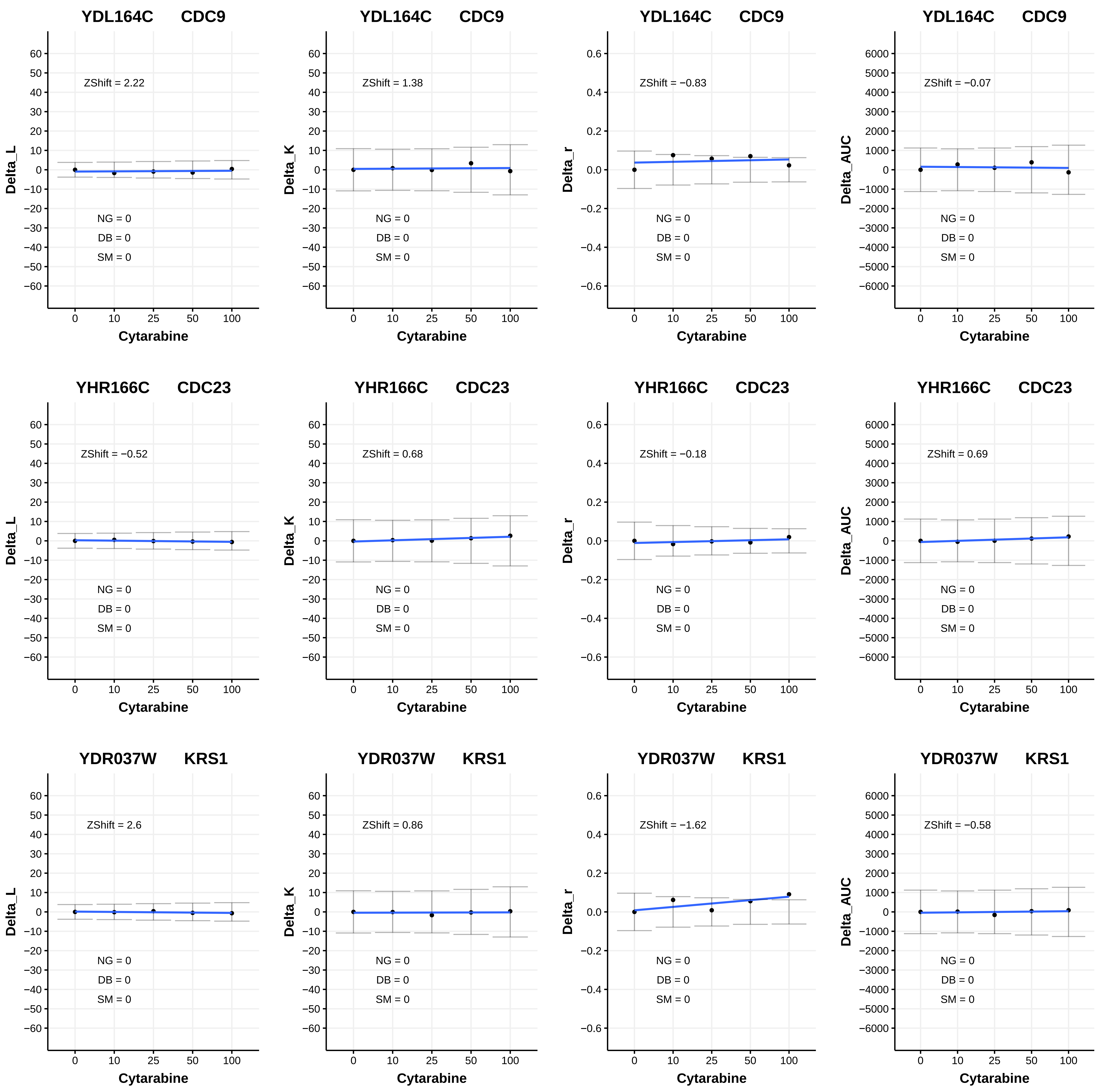
YDL164C CDC9
ZShift = 2.22
Delta_L
NG = 0
DB = 0
SM = 0
Cytarabine
YDL164C CDC9
ZShift = 1.38
Delta_K
NG = 0
DB = 0
SM = 0
Cytarabine
YDL164C CDC9
ZShift = −0.83
Delta_r
NG = 0
DB = 0
SM = 0
Cytarabine
YDL164C CDC9
ZShift = −0.07
Delta_AUC
NG = 0
DB = 0
SM = 0
Cytarabine
YHR166C CDC23
ZShift = −0.52
Delta_L
NG = 0
DB = 0
SM = 0
Cytarabine
YHR166C CDC23
ZShift = 0.68
Delta_K
NG = 0
DB = 0
SM = 0
Cytarabine
YHR166C CDC23
ZShift = −0.18
Delta_r
NG = 0
DB = 0
SM = 0
Cytarabine
YHR166C CDC23
ZShift = 0.69
Delta_AUC
NG = 0
DB = 0
SM = 0
Cytarabine
YDR037W KRS1
ZShift = 2.6
Delta_L
NG = 0
DB = 0
SM = 0
Cytarabine
YDR037W KRS1
ZShift = 0.86
Delta_K
NG = 0
DB = 0
SM = 0
Cytarabine
YDR037W KRS1
ZShift = −1.62
Delta_r
NG = 0
DB = 0
SM = 0
Cytarabine
YDR037W KRS1
ZShift = −0.58
Delta_AUC
NG = 0
DB = 0
SM = 0
Cytarabine

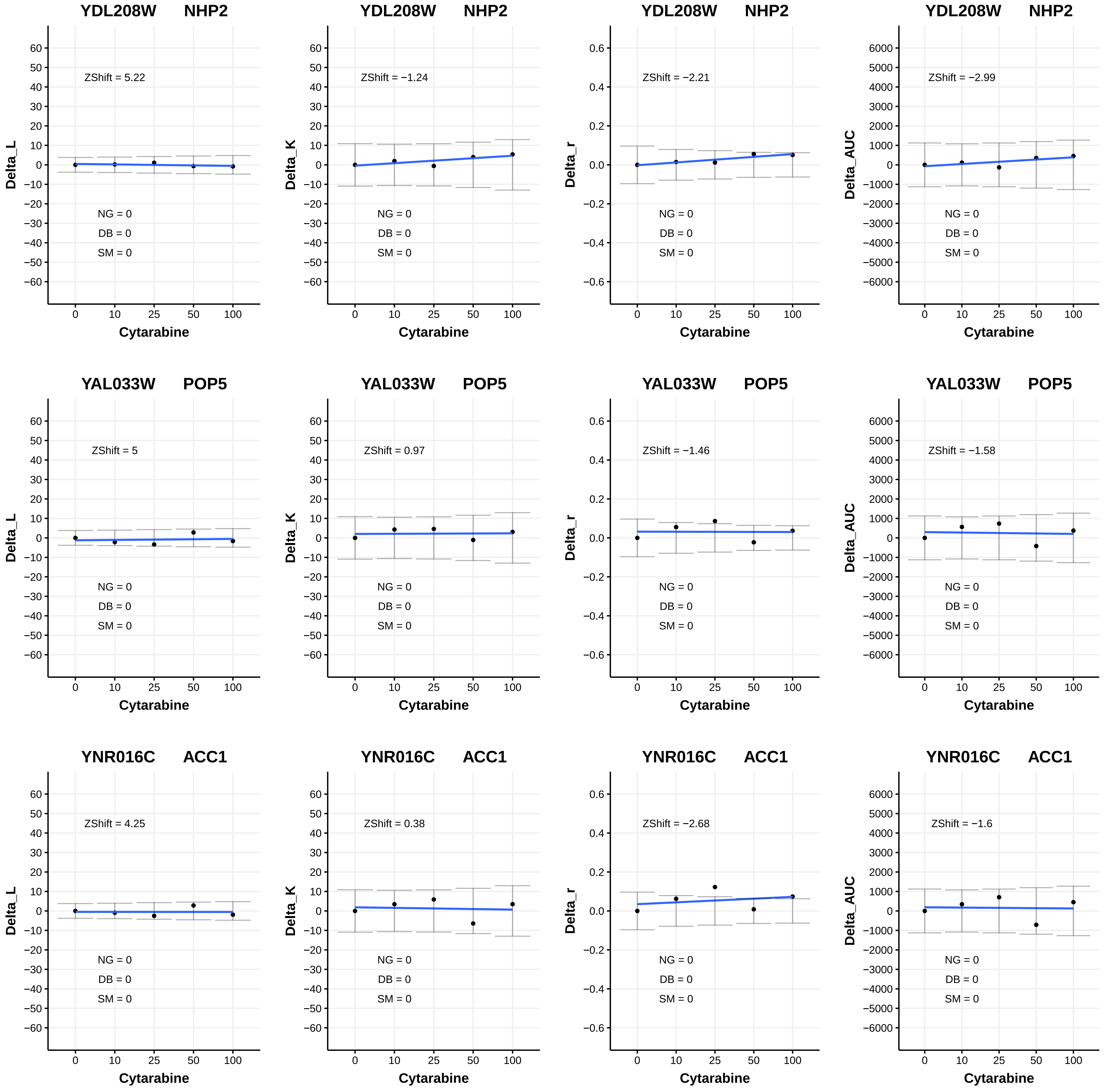
YDL208W NHP2
ZShift = 5.22
NG = 0
DB = 0
SM = 0
Delta_L
Cytarabine
YDL208W NHP2
ZShift = −1.24
NG = 0
DB = 0
SM = 0
Delta_K
Cytarabine
YDL208W NHP2
ZShift = −2.21
NG = 0
DB = 0
SM = 0
Delta_r
Cytarabine
YDL208W NHP2
ZShift = −2.99
NG = 0
DB = 0
SM = 0
Delta_AUC
Cytarabine
YAL033W POP5
ZShift = 5
NG = 0
DB = 0
SM = 0
Delta_L
Cytarabine
YAL033W POP5
ZShift = 0.97
NG = 0
DB = 0
SM = 0
Delta_K
Cytarabine
YAL033W POP5
ZShift = −1.46
NG = 0
DB = 0
SM = 0
Delta_r
Cytarabine
YAL033W POP5
ZShift = −1.58
NG = 0
DB = 0
SM = 0
Delta_AUC
Cytarabine
YNR016C ACC1
ZShift = 4.25
NG = 0
DB = 0
SM = 0
Delta_L
Cytarabine
YNR016C ACC1
ZShift = 0.38
NG = 0
DB = 0
SM = 0
Delta_K
Cytarabine
YNR016C ACC1
ZShift = −2.68
NG = 0
DB = 0
SM = 0
Delta_r
Cytarabine
YNR016C ACC1
ZShift = −1.6
NG = 0
DB = 0
SM = 0
Delta_AUC
Cytarabine

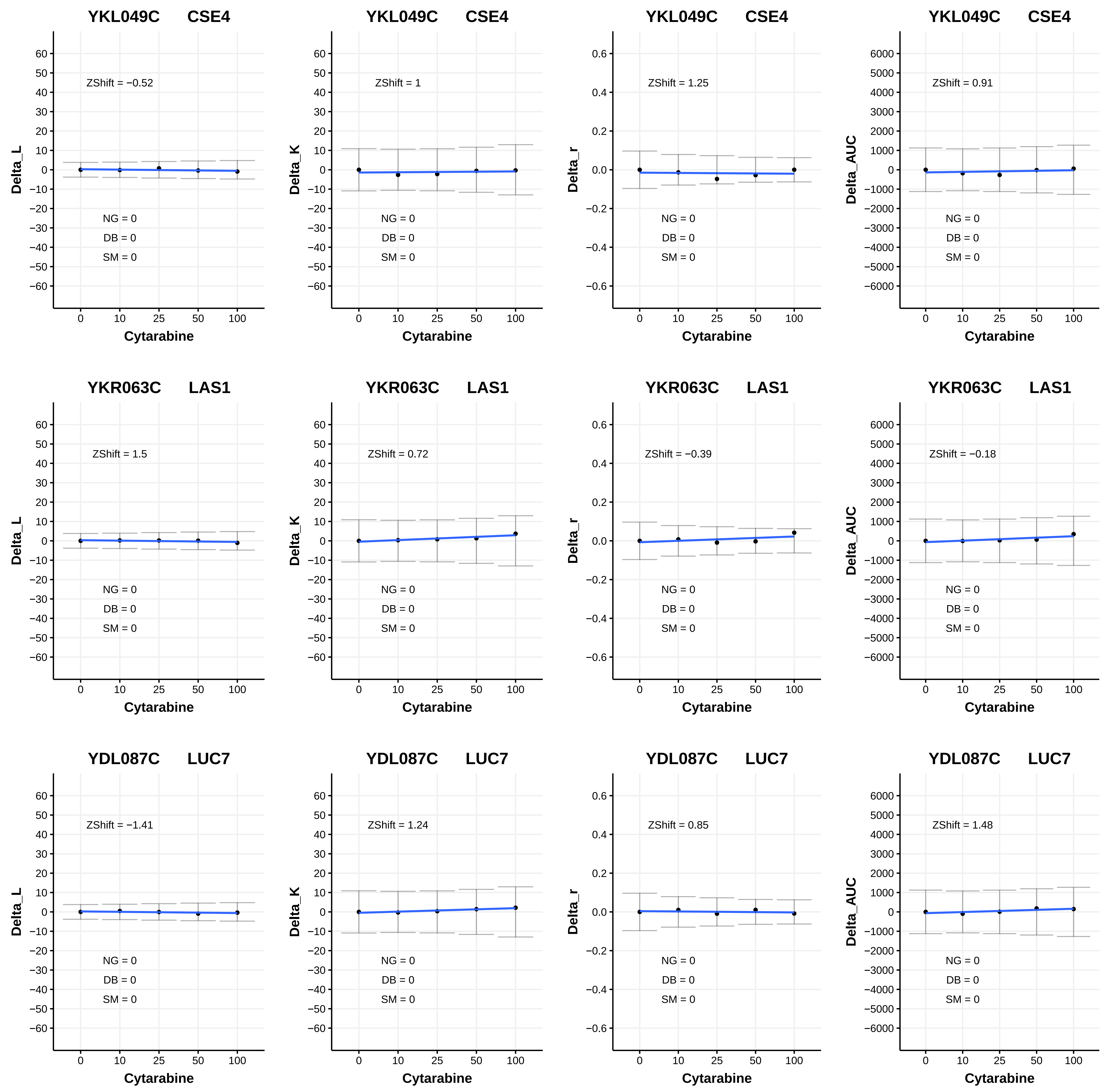
YKL049C CSE4
ZShift = −0.52
ZShift = 1
ZShift = 1.25
ZShift = 0.91
YKR063C LAS1
ZShift = 1.5
ZShift = 0.72
ZShift = −0.39
ZShift = −0.18
YDL087C LUC7
ZShift = −1.41
ZShift = 1.24
ZShift = 0.85
ZShift = 1.48
NG = 0
DB = 0
SM = 0
Delta_L
Delta_K
Delta_r
Delta_AUC
Cytarabine
0
10
25
50
100

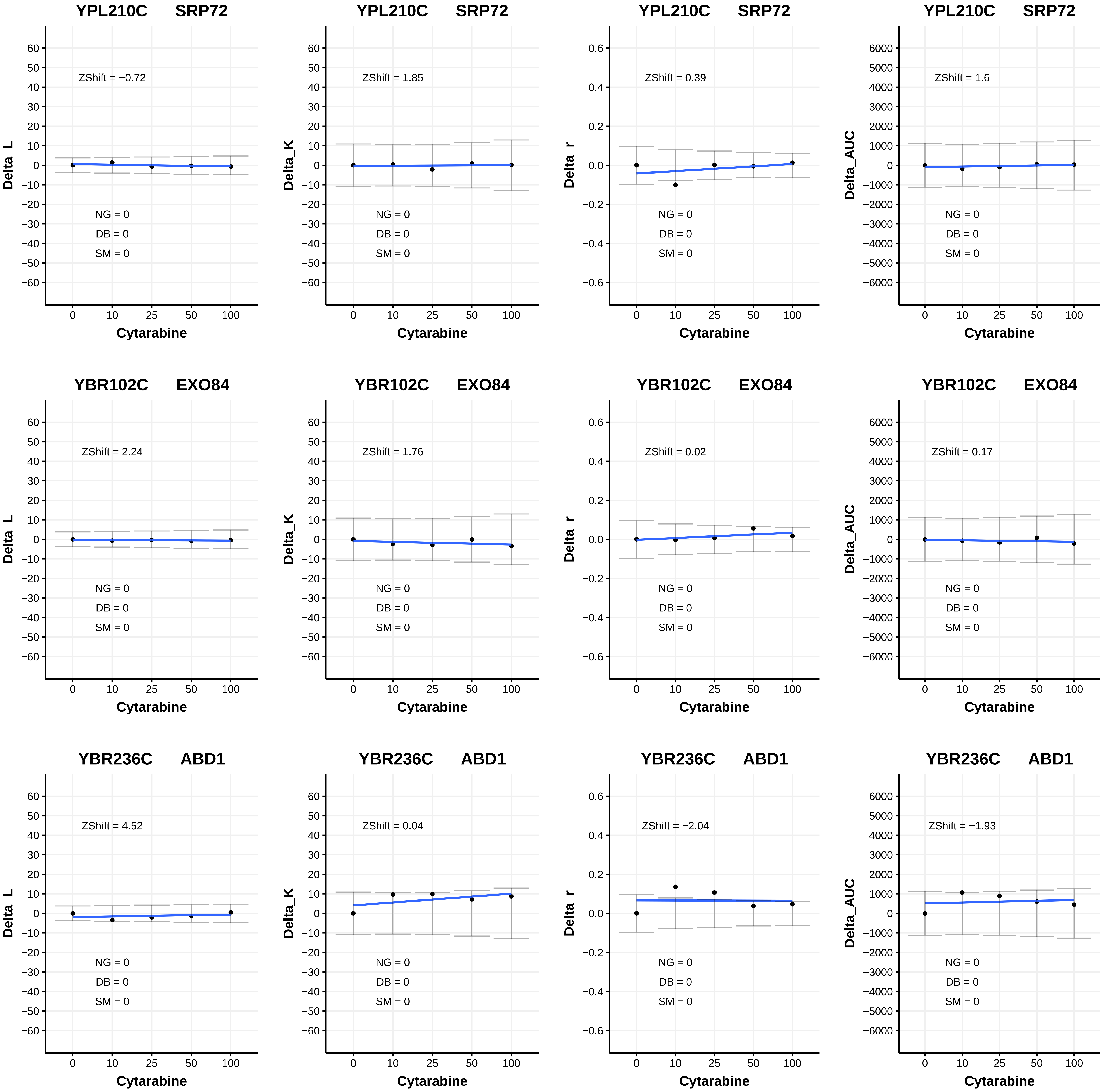
YPL210C SRP72
Delta_L
ZShift = −0.72
NG = 0
DB = 0
SM = 0
Cytarabine
YPL210C SRP72
Delta_K
ZShift = 1.85
NG = 0
DB = 0
SM = 0
Cytarabine
YPL210C SRP72
Delta_r
ZShift = 0.39
NG = 0
DB = 0
SM = 0
Cytarabine
YPL210C SRP72
Delta_AUC
ZShift = 1.6
NG = 0
DB = 0
SM = 0
Cytarabine
YBR102C EXO84
Delta_L
ZShift = 2.24
NG = 0
DB = 0
SM = 0
Cytarabine
YBR102C EXO84
Delta_K
ZShift = 1.76
NG = 0
DB = 0
SM = 0
Cytarabine
YBR102C EXO84
Delta_r
ZShift = 0.02
NG = 0
DB = 0
SM = 0
Cytarabine
YBR102C EXO84
Delta_AUC
ZShift = 0.17
NG = 0
DB = 0
SM = 0
Cytarabine
YBR236C ABD1
Delta_L
ZShift = 4.52
NG = 0
DB = 0
SM = 0
Cytarabine
YBR236C ABD1
Delta_K
ZShift = 0.04
NG = 0
DB = 0
SM = 0
Cytarabine
YBR236C ABD1
Delta_r
ZShift = −2.04
NG = 0
DB = 0
SM = 0
Cytarabine
YBR236C ABD1
Delta_AUC
ZShift = −1.93
NG = 0
DB = 0
SM = 0
Cytarabine

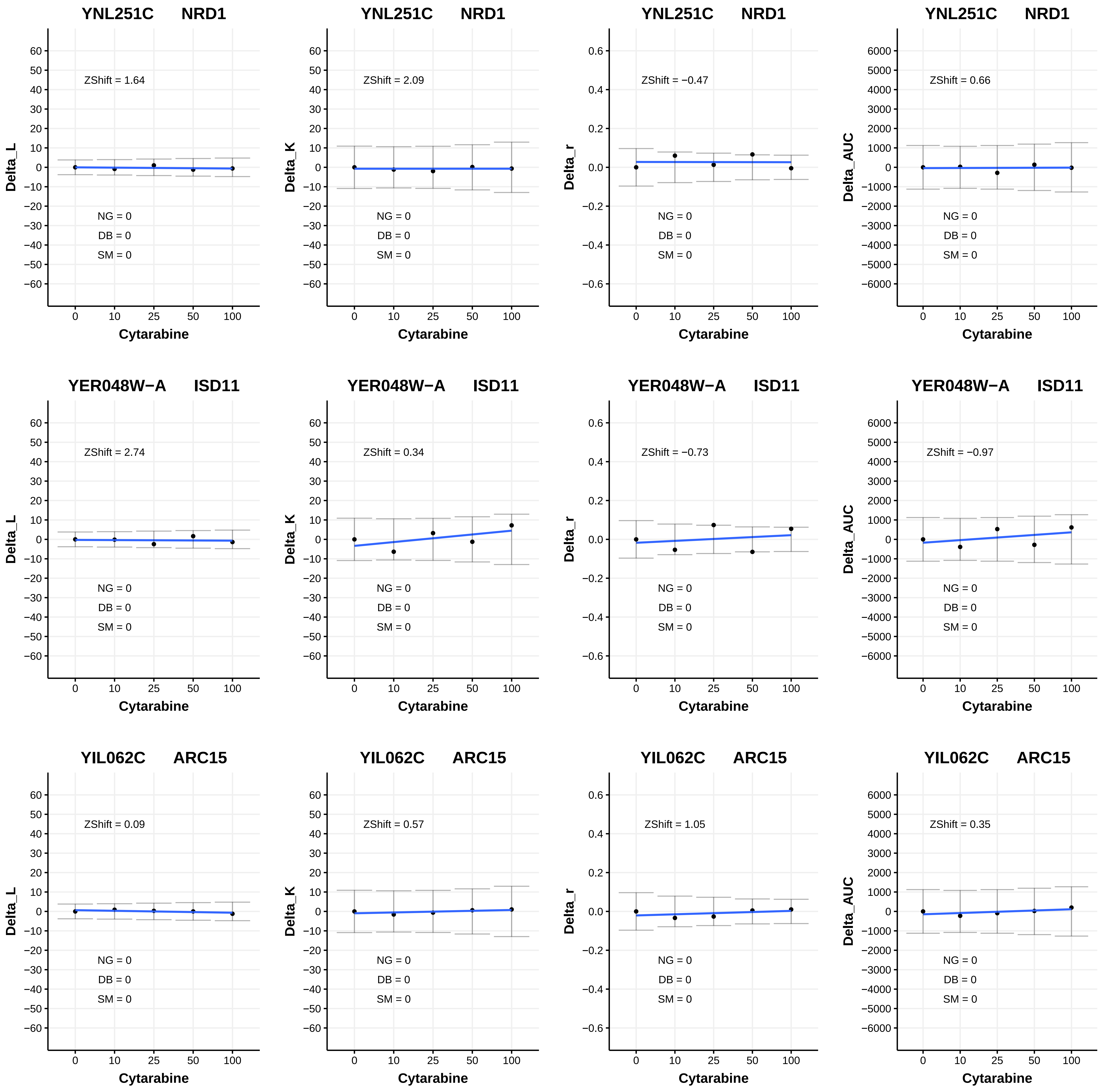
YNL251C NRD1
ZShift = 1.64
ZShift = 2.09
ZShift = −0.47
ZShift = 0.66
YER048W−A ISD11
ZShift = 2.74
ZShift = 0.34
ZShift = −0.73
ZShift = −0.97
YIL062C ARC15
ZShift = 0.09
ZShift = 0.57
ZShift = 1.05
ZShift = 0.35
NG = 0
DB = 0
SM = 0
Delta_L
Delta_K
Delta_r
Delta_AUC
Cytarabine
0
10
25
50
100

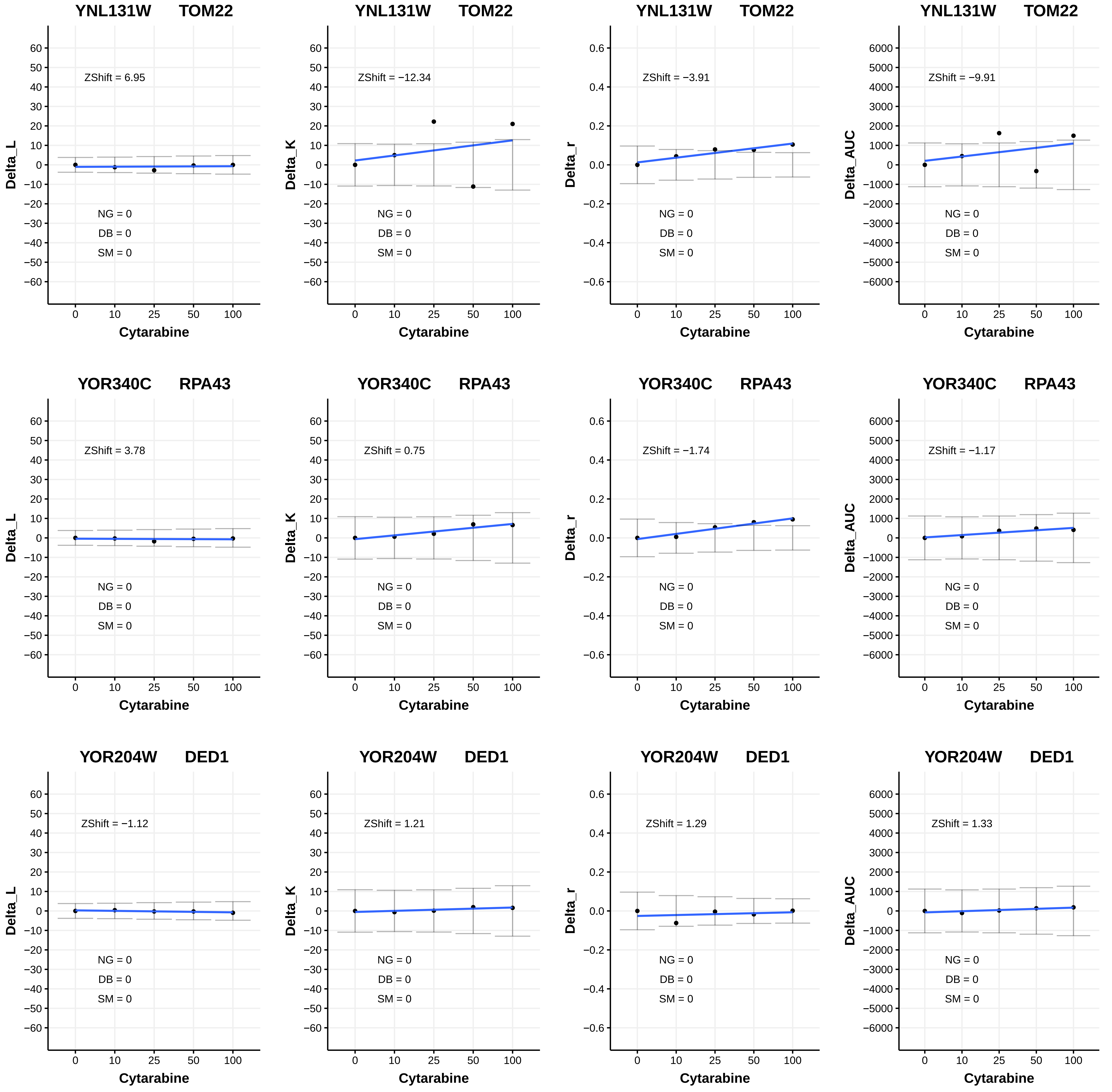
YNL131W TOM22
ZShift = 6.95
ZShift = −12.34
ZShift = −3.91
ZShift = −9.91
YOR340C RPA43
ZShift = 3.78
ZShift = 0.75
ZShift = −1.74
ZShift = −1.17
YOR204W DED1
ZShift = −1.12
ZShift = 1.21
ZShift = 1.29
ZShift = 1.33
NG = 0
DB = 0
SM = 0
Delta_L
Delta_K
Delta_r
Delta_AUC
Cytarabine
0
10
25
50
100

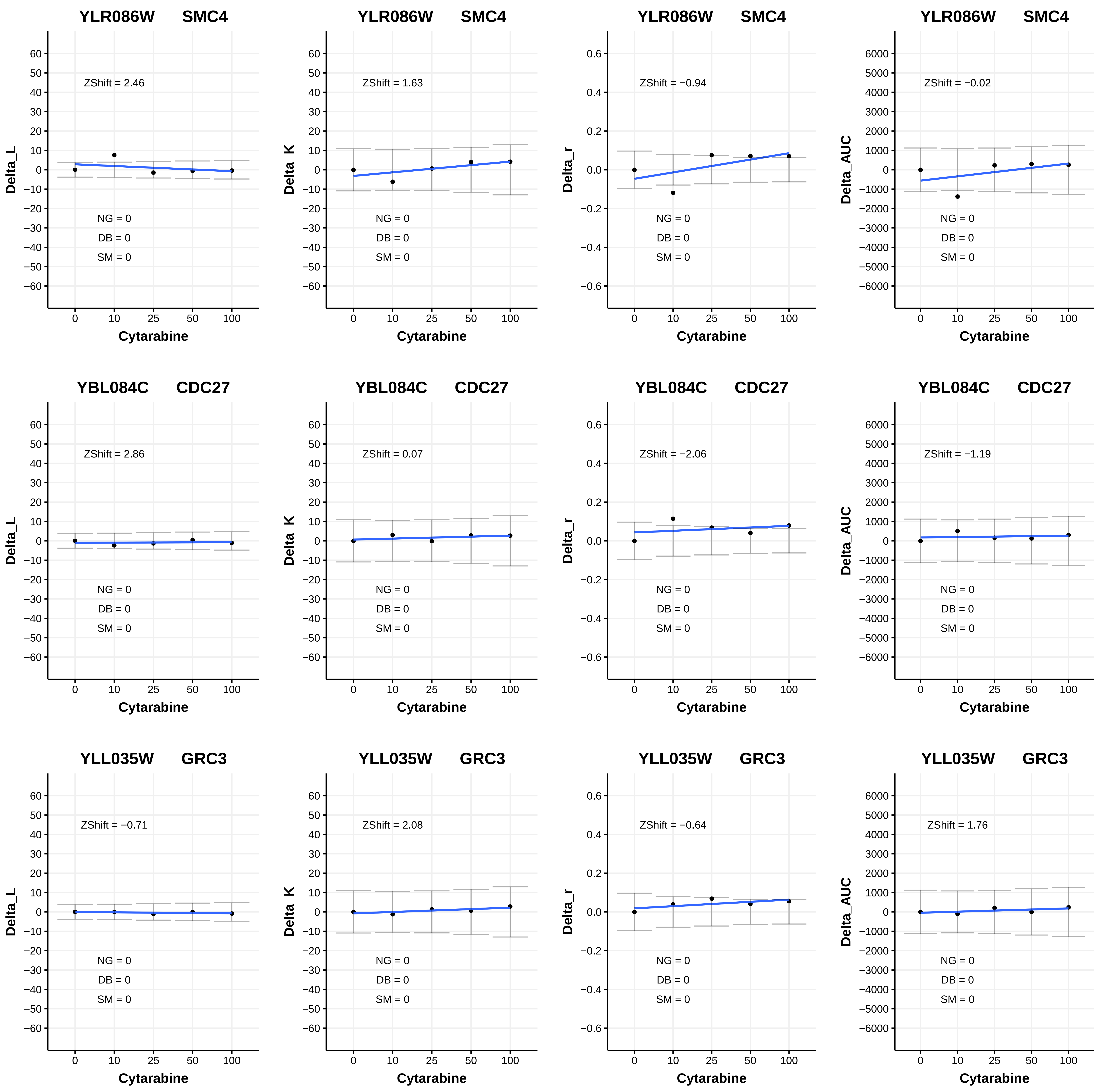
YLR086W SMC4
ZShift = 2.46
ZShift = 1.63
ZShift = −0.94
ZShift = −0.02
YBL084C CDC27
ZShift = 2.86
ZShift = 0.07
ZShift = −2.06
ZShift = −1.19
YLL035W GRC3
ZShift = −0.71
ZShift = 2.08
ZShift = −0.64
ZShift = 1.76
NG = 0
DB = 0
SM = 0
Delta_L
Delta_K
Delta_r
Delta_AUC
Cytarabine

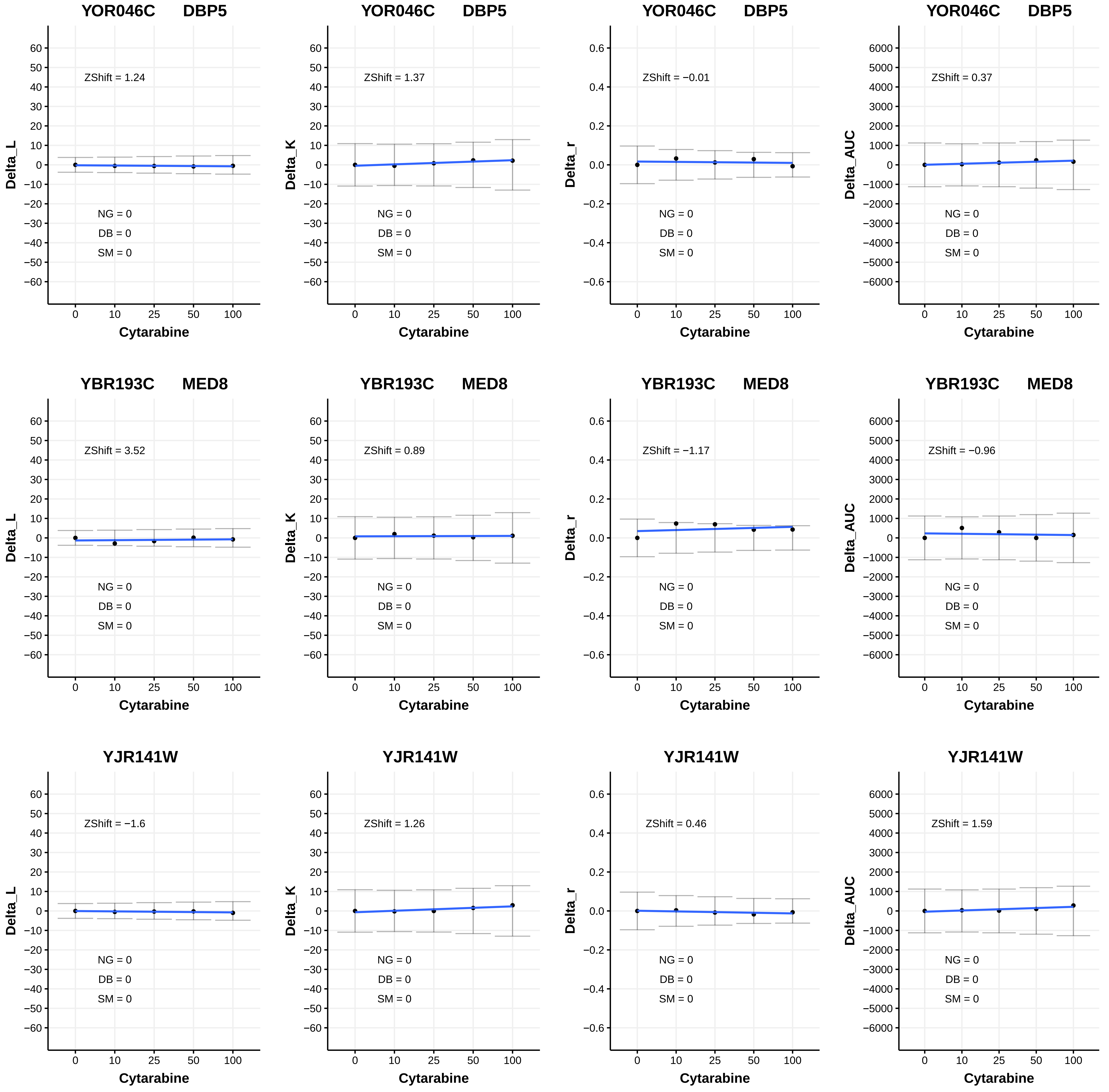
YOR046C DBP5
ZShift = 1.24
ZShift = 1.37
ZShift = −0.01
ZShift = 0.37
YBR193C MED8
ZShift = 3.52
ZShift = 0.89
ZShift = −1.17
ZShift = −0.96
YJR141W
ZShift = −1.6
ZShift = 1.26
ZShift = 0.46
ZShift = 1.59
NG = 0
DB = 0
SM = 0
Delta_L
Delta_K
Delta_r
Delta_AUC
Cytarabine
0 10 25 50 100

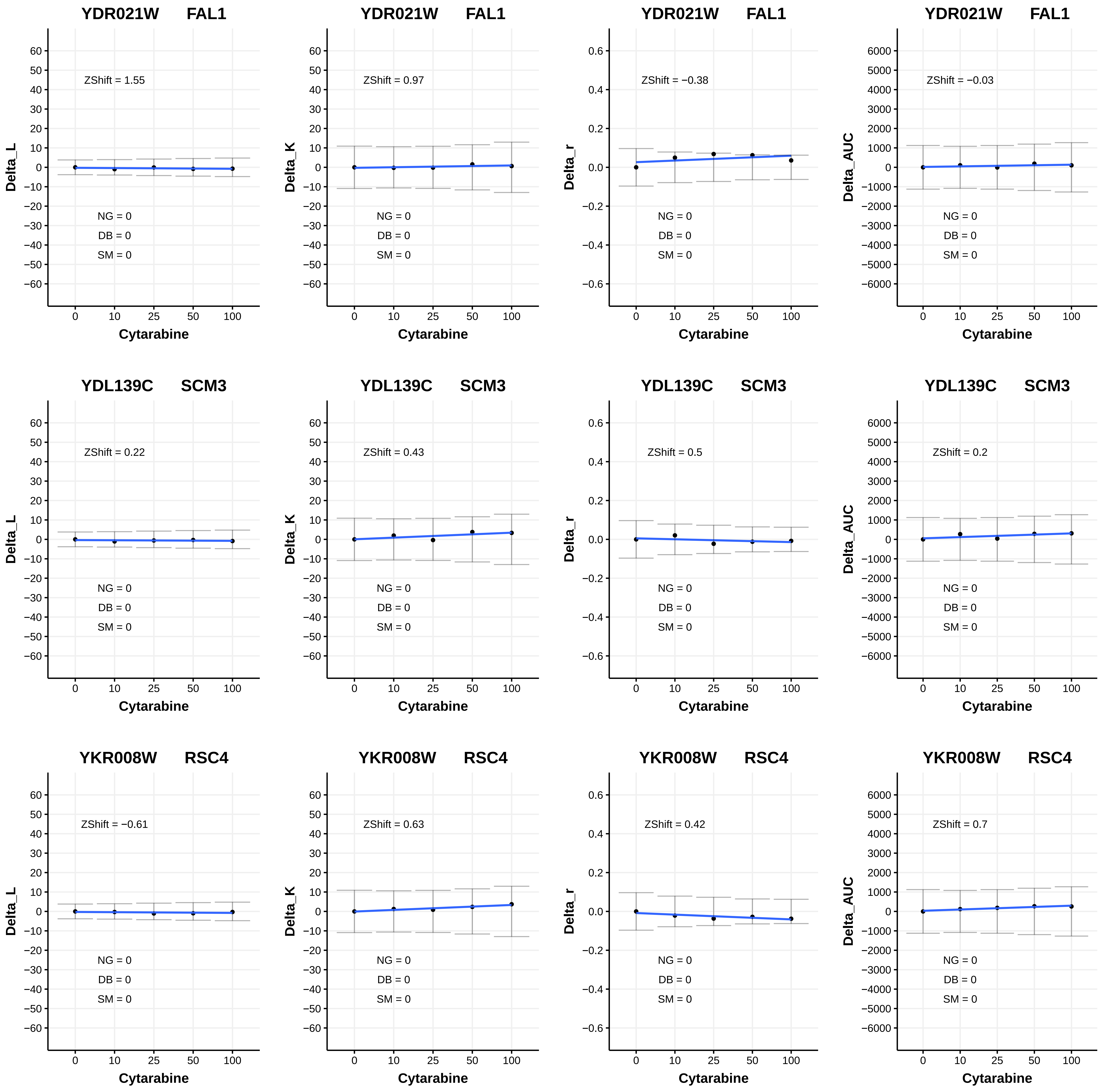
YDR021W FAL1
ZShift = 1.55
NG = 0
DB = 0
SM = 0
Delta_L
Cytarabine
YDR021W FAL1
ZShift = 0.97
NG = 0
DB = 0
SM = 0
Delta_K
Cytarabine
YDR021W FAL1
ZShift = −0.38
NG = 0
DB = 0
SM = 0
Delta_r
Cytarabine
YDR021W FAL1
ZShift = −0.03
NG = 0
DB = 0
SM = 0
Delta_AUC
Cytarabine
YDL139C SCM3
ZShift = 0.22
NG = 0
DB = 0
SM = 0
Delta_L
Cytarabine
YDL139C SCM3
ZShift = 0.43
NG = 0
DB = 0
SM = 0
Delta_K
Cytarabine
YDL139C SCM3
ZShift = 0.5
NG = 0
DB = 0
SM = 0
Delta_r
Cytarabine
YDL139C SCM3
ZShift = 0.2
NG = 0
DB = 0
SM = 0
Delta_AUC
Cytarabine
YKR008W RSC4
ZShift = −0.61
NG = 0
DB = 0
SM = 0
Delta_L
Cytarabine
YKR008W RSC4
ZShift = 0.63
NG = 0
DB = 0
SM = 0
Delta_K
Cytarabine
YKR008W RSC4
ZShift = 0.42
NG = 0
DB = 0
SM = 0
Delta_r
Cytarabine
YKR008W RSC4
ZShift = 0.7
NG = 0
DB = 0
SM = 0
Delta_AUC
Cytarabine

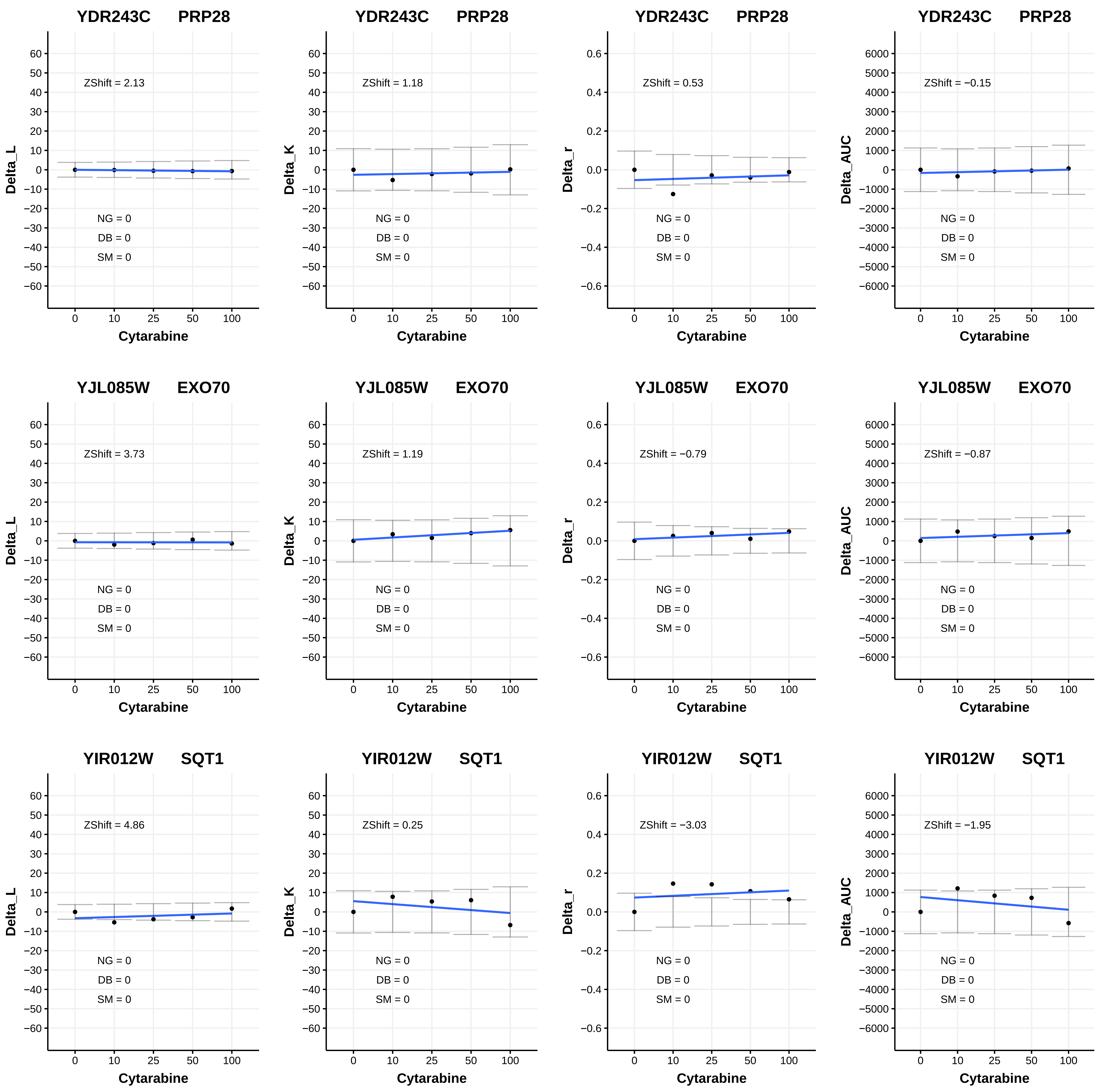
YDR243C PRP28
ZShift = 2.13
ZShift = 1.18
ZShift = 0.53
ZShift = −0.15
YJL085W EXO70
ZShift = 3.73
ZShift = 1.19
ZShift = −0.79
ZShift = −0.87
YIR012W SQT1
ZShift = 4.86
ZShift = 0.25
ZShift = −3.03
ZShift = −1.95
NG = 0
DB = 0
SM = 0
Delta_L
Delta_K
Delta_r
Delta_AUC
Cytarabine
0 10 25 50 100

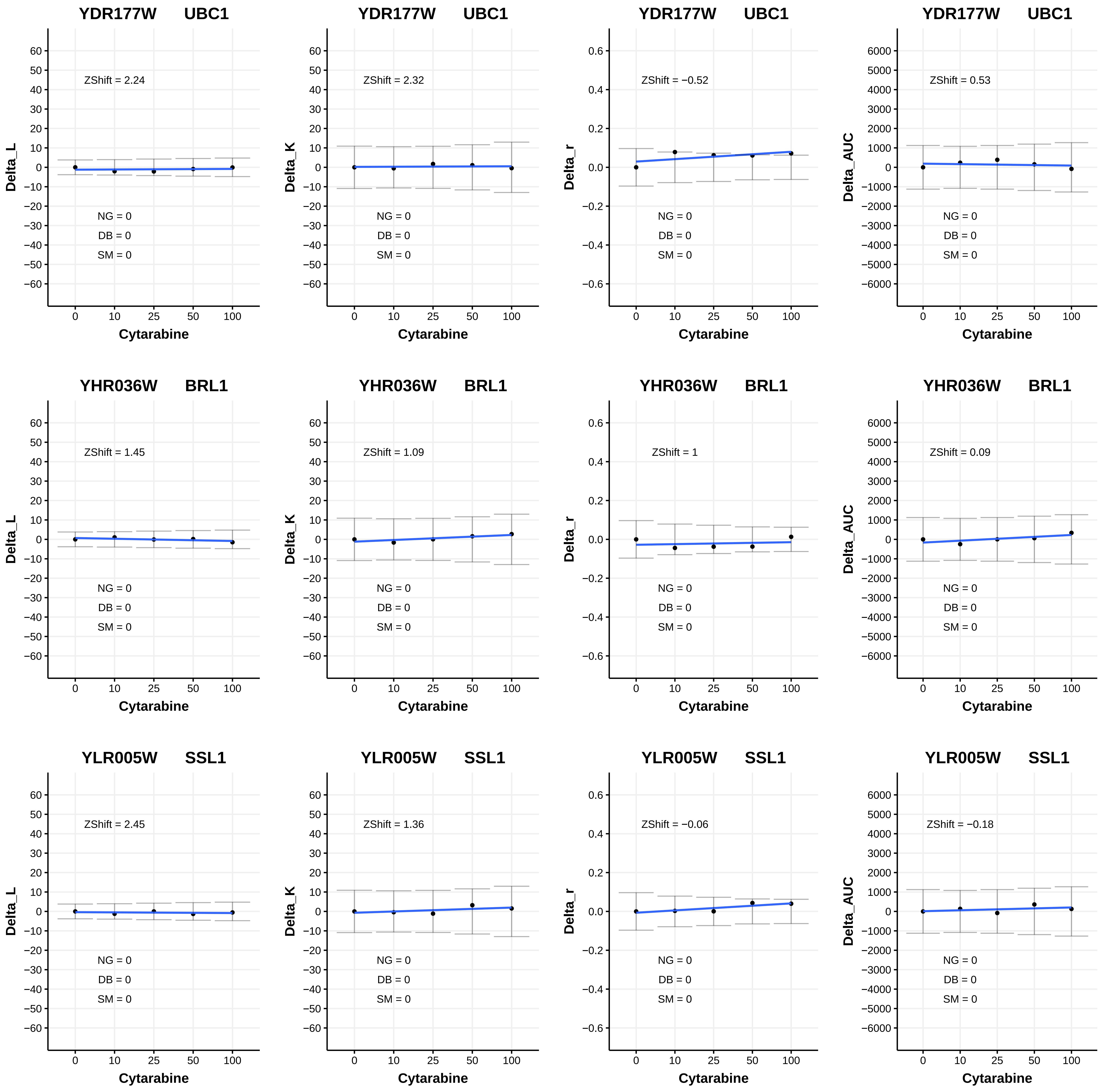

YDR177W UBC1
ZShift = 2.24
NG = 0
DB = 0
SM = 0
Delta_L
Cytarabine
YDR177W UBC1
ZShift = 2.32
NG = 0
DB = 0
SM = 0
Delta_K
Cytarabine
YDR177W UBC1
ZShift = −0.52
NG = 0
DB = 0
SM = 0
Delta_r
Cytarabine
YDR177W UBC1
ZShift = 0.53
NG = 0
DB = 0
SM = 0
Delta_AUC
Cytarabine
YHR036W BRL1
ZShift = 1.45
NG = 0
DB = 0
SM = 0
Delta_L
Cytarabine
YHR036W BRL1
ZShift = 1.09
NG = 0
DB = 0
SM = 0
Delta_K
Cytarabine
YHR036W BRL1
ZShift = 1
NG = 0
DB = 0
SM = 0
Delta_r
Cytarabine
YHR036W BRL1
ZShift = 0.09
NG = 0
DB = 0
SM = 0
Delta_AUC
Cytarabine
YLR005W SSL1
ZShift = 2.45
NG = 0
DB = 0
SM = 0
Delta_L
Cytarabine
YLR005W SSL1
ZShift = 1.36
NG = 0
DB = 0
SM = 0
Delta_K
Cytarabine
YLR005W SSL1
ZShift = −0.06
NG = 0
DB = 0
SM = 0
Delta_r
Cytarabine
YLR005W SSL1
ZShift = −0.18
NG = 0
DB = 0
SM = 0
Delta_AUC
Cytarabine
0
10
25
50
100

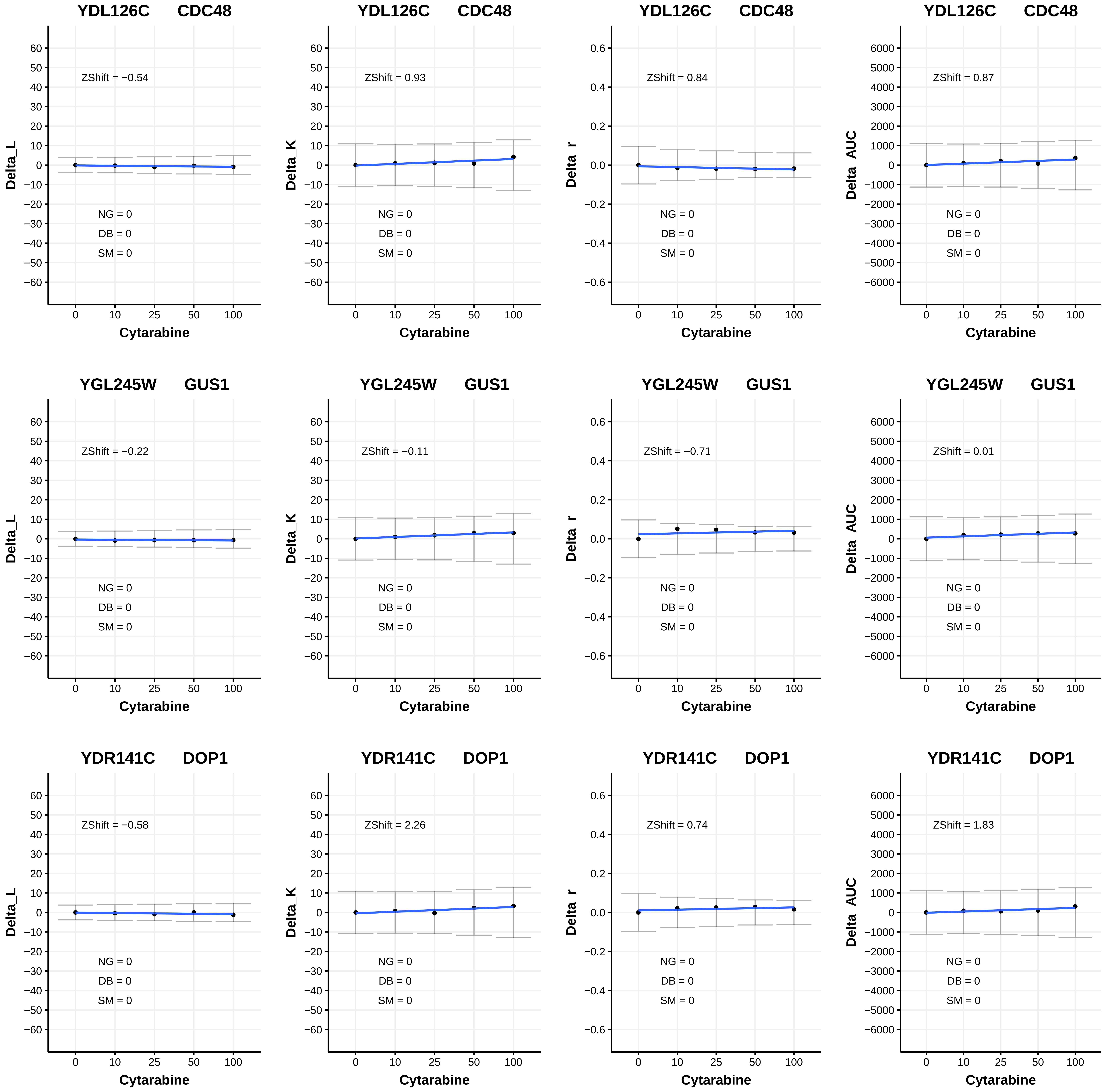
YDL126C CDC48
ZShift = −0.54
NG = 0
DB = 0
SM = 0
Delta_L
Cytarabine
YDL126C CDC48
ZShift = 0.93
NG = 0
DB = 0
SM = 0
Delta_K
Cytarabine
YDL126C CDC48
ZShift = 0.84
NG = 0
DB = 0
SM = 0
Delta_r
Cytarabine
YDL126C CDC48
ZShift = 0.87
NG = 0
DB = 0
SM = 0
Delta_AUC
Cytarabine
YGL245W GUS1
ZShift = −0.22
NG = 0
DB = 0
SM = 0
Delta_L
Cytarabine
YGL245W GUS1
ZShift = −0.11
NG = 0
DB = 0
SM = 0
Delta_K
Cytarabine
YGL245W GUS1
ZShift = −0.71
NG = 0
DB = 0
SM = 0
Delta_r
Cytarabine
YGL245W GUS1
ZShift = 0.01
NG = 0
DB = 0
SM = 0
Delta_AUC
Cytarabine
YDR141C DOP1
ZShift = −0.58
NG = 0
DB = 0
SM = 0
Delta_L
Cytarabine
YDR141C DOP1
ZShift = 2.26
NG = 0
DB = 0
SM = 0
Delta_K
Cytarabine
YDR141C DOP1
ZShift = 0.74
NG = 0
DB = 0
SM = 0
Delta_r
Cytarabine
YDR141C DOP1
ZShift = 1.83
NG = 0
DB = 0
SM = 0
Delta_AUC
Cytarabine
0
10
25
50
100

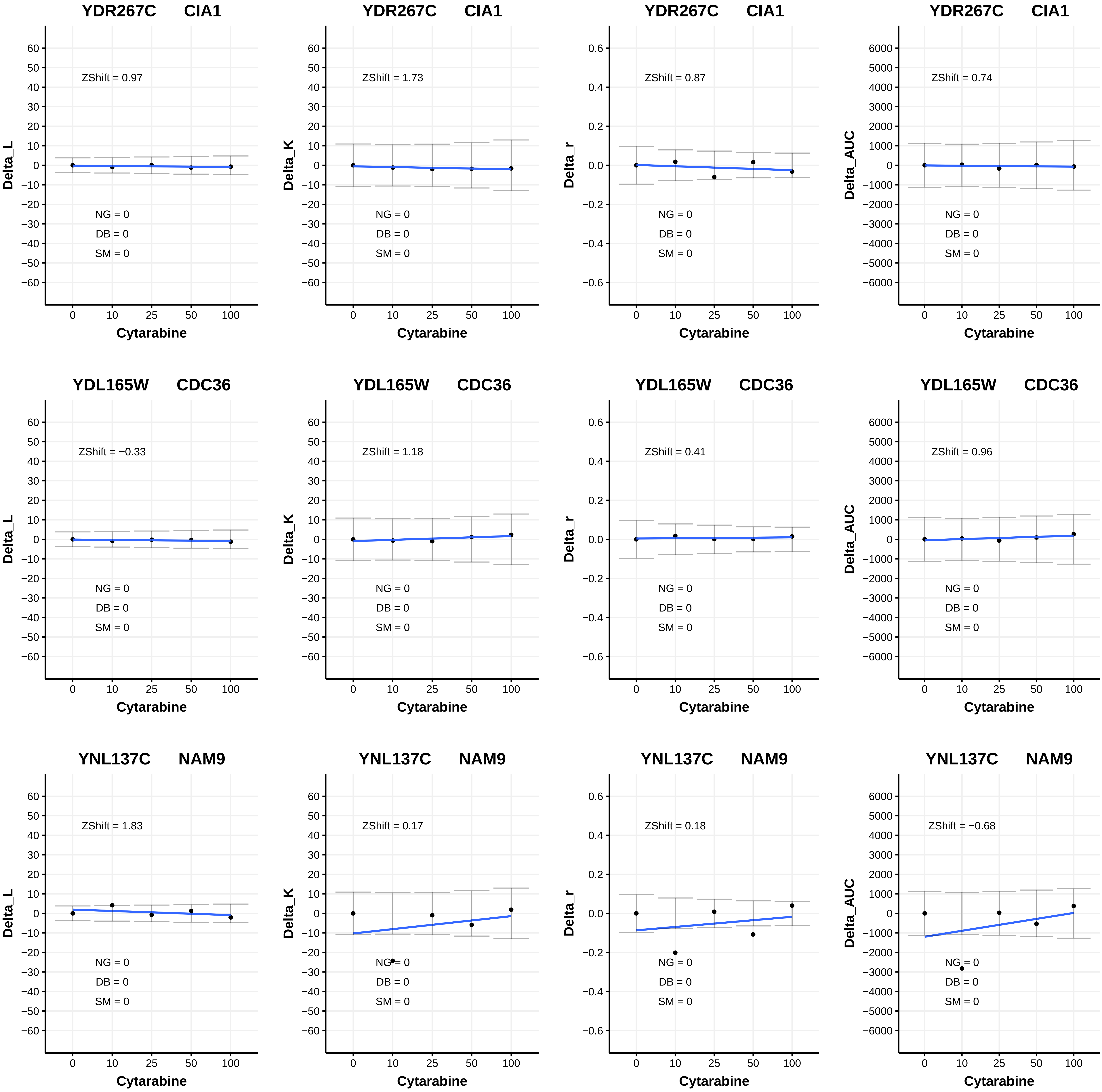
YDR267C CIA1
ZShift = 0.97
NG = 0
DB = 0
SM = 0
Delta_L
Cytarabine
YDR267C CIA1
ZShift = 1.73
NG = 0
DB = 0
SM = 0
Delta_K
Cytarabine
YDR267C CIA1
ZShift = 0.87
NG = 0
DB = 0
SM = 0
Delta_r
Cytarabine
YDR267C CIA1
ZShift = 0.74
NG = 0
DB = 0
SM = 0
Delta_AUC
Cytarabine
YDL165W CDC36
ZShift = −0.33
NG = 0
DB = 0
SM = 0
Delta_L
Cytarabine
YDL165W CDC36
ZShift = 1.18
NG = 0
DB = 0
SM = 0
Delta_K
Cytarabine
YDL165W CDC36
ZShift = 0.41
NG = 0
DB = 0
SM = 0
Delta_r
Cytarabine
YDL165W CDC36
ZShift = 0.96
NG = 0
DB = 0
SM = 0
Delta_AUC
Cytarabine
YNL137C NAM9
ZShift = 1.83
NG = 0
DB = 0
SM = 0
Delta_L
Cytarabine
YNL137C NAM9
ZShift = 0.17
NG = 0
DB = 0
SM = 0
Delta_K
Cytarabine
YNL137C NAM9
ZShift = 0.18
NG = 0
DB = 0
SM = 0
Delta_r
Cytarabine
YNL137C NAM9
ZShift = −0.68
NG = 0
DB = 0
SM = 0
Delta_AUC
Cytarabine

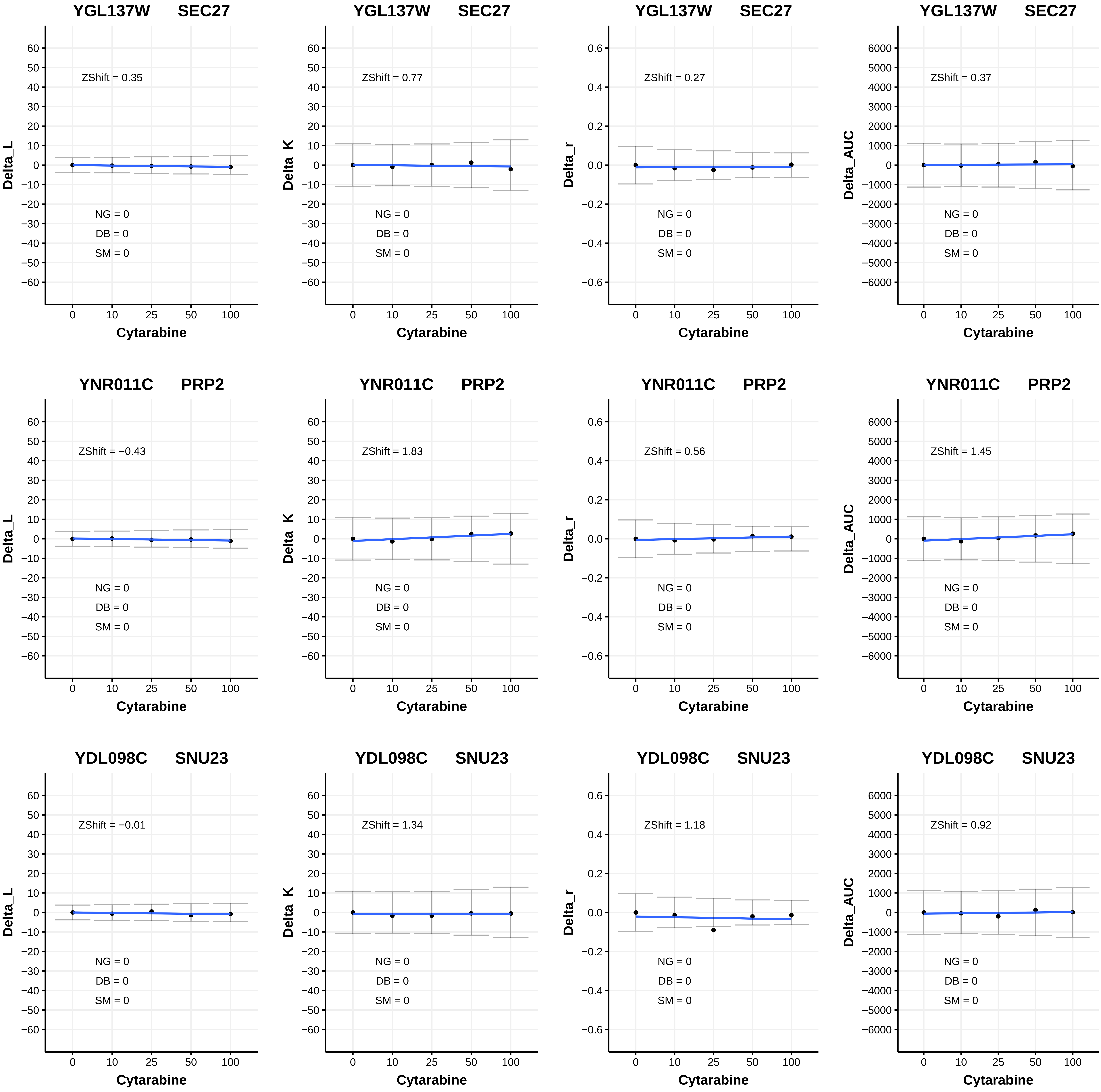
YGL137W SEC27
ZShift = 0.35
NG = 0
DB = 0
SM = 0
Delta_L
Cytarabine
YGL137W SEC27
ZShift = 0.77
NG = 0
DB = 0
SM = 0
Delta_K
Cytarabine
YGL137W SEC27
ZShift = 0.27
NG = 0
DB = 0
SM = 0
Delta_r
Cytarabine
YGL137W SEC27
ZShift = 0.37
NG = 0
DB = 0
SM = 0
Delta_AUC
Cytarabine
YNR011C PRP2
ZShift = −0.43
NG = 0
DB = 0
SM = 0
Delta_L
Cytarabine
YNR011C PRP2
ZShift = 1.83
NG = 0
DB = 0
SM = 0
Delta_K
Cytarabine
YNR011C PRP2
ZShift = 0.56
NG = 0
DB = 0
SM = 0
Delta_r
Cytarabine
YNR011C PRP2
ZShift = 1.45
NG = 0
DB = 0
SM = 0
Delta_AUC
Cytarabine
YDL098C SNU23
ZShift = −0.01
NG = 0
DB = 0
SM = 0
Delta_L
Cytarabine
YDL098C SNU23
ZShift = 1.34
NG = 0
DB = 0
SM = 0
Delta_K
Cytarabine
YDL098C SNU23
ZShift = 1.18
NG = 0
DB = 0
SM = 0
Delta_r
Cytarabine
YDL098C SNU23
ZShift = 0.92
NG = 0
DB = 0
SM = 0
Delta_AUC
Cytarabine

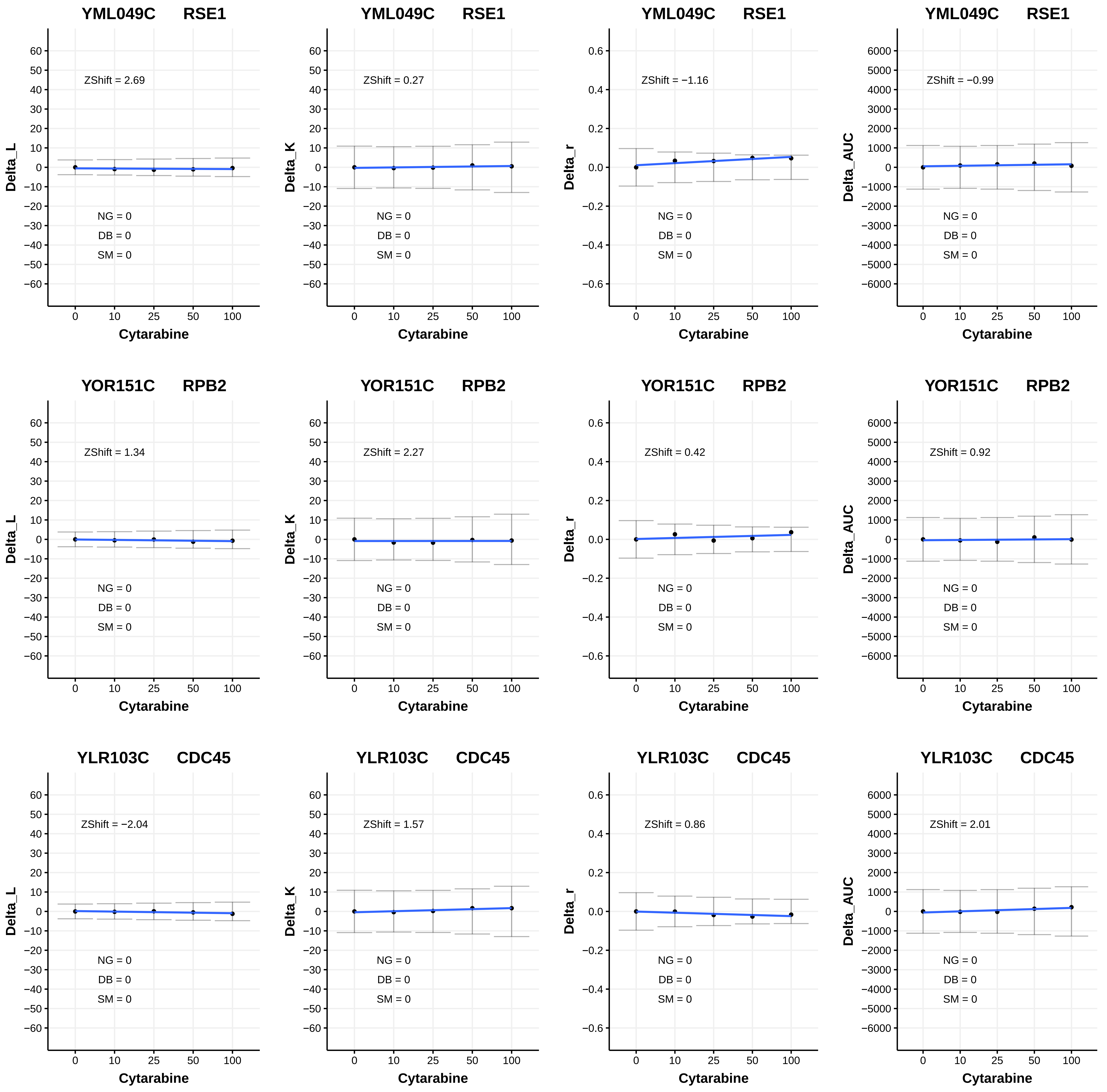
YML049C RSE1
ZShift = 2.69
ZShift = 0.27
ZShift = −1.16
ZShift = −0.99
YOR151C RPB2
ZShift = 1.34
ZShift = 2.27
ZShift = 0.42
ZShift = 0.92
YLR103C CDC45
ZShift = −2.04
ZShift = 1.57
ZShift = 0.86
ZShift = 2.01
NG = 0
DB = 0
SM = 0
Delta_L
Delta_K
Delta_r
Delta_AUC
Cytarabine

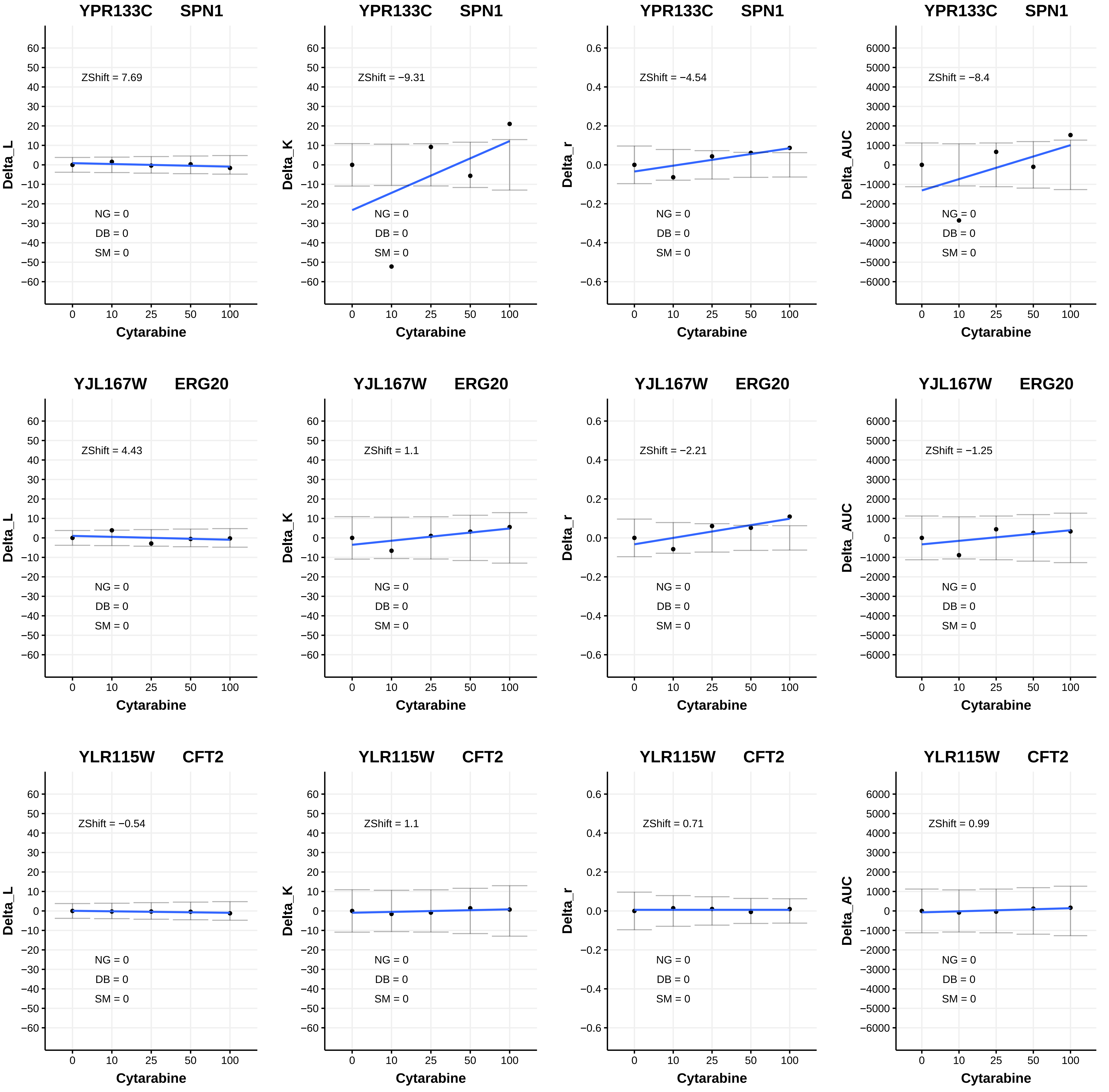

YPR133C SPN1
ZShift = 7.69
Delta_L
NG = 0
DB = 0
SM = 0
Cytarabine
YPR133C SPN1
ZShift = −9.31
Delta_K
NG = 0
DB = 0
SM = 0
Cytarabine
YPR133C SPN1
ZShift = −4.54
Delta_r
NG = 0
DB = 0
SM = 0
Cytarabine
YPR133C SPN1
ZShift = −8.4
Delta_AUC
NG = 0
DB = 0
SM = 0
Cytarabine
YJL167W ERG20
ZShift = 4.43
Delta_L
NG = 0
DB = 0
SM = 0
Cytarabine
YJL167W ERG20
ZShift = 1.1
Delta_K
NG = 0
DB = 0
SM = 0
Cytarabine
YJL167W ERG20
ZShift = −2.21
Delta_r
NG = 0
DB = 0
SM = 0
Cytarabine
YJL167W ERG20
ZShift = −1.25
Delta_AUC
NG = 0
DB = 0
SM = 0
Cytarabine
YLR115W CFT2
ZShift = −0.54
Delta_L
NG = 0
DB = 0
SM = 0
Cytarabine
YLR115W CFT2
ZShift = 1.1
Delta_K
NG = 0
DB = 0
SM = 0
Cytarabine
YLR115W CFT2
ZShift = 0.71
Delta_r
NG = 0
DB = 0
SM = 0
Cytarabine
YLR115W CFT2
ZShift = 0.99
Delta_AUC
NG = 0
DB = 0
SM = 0
Cytarabine

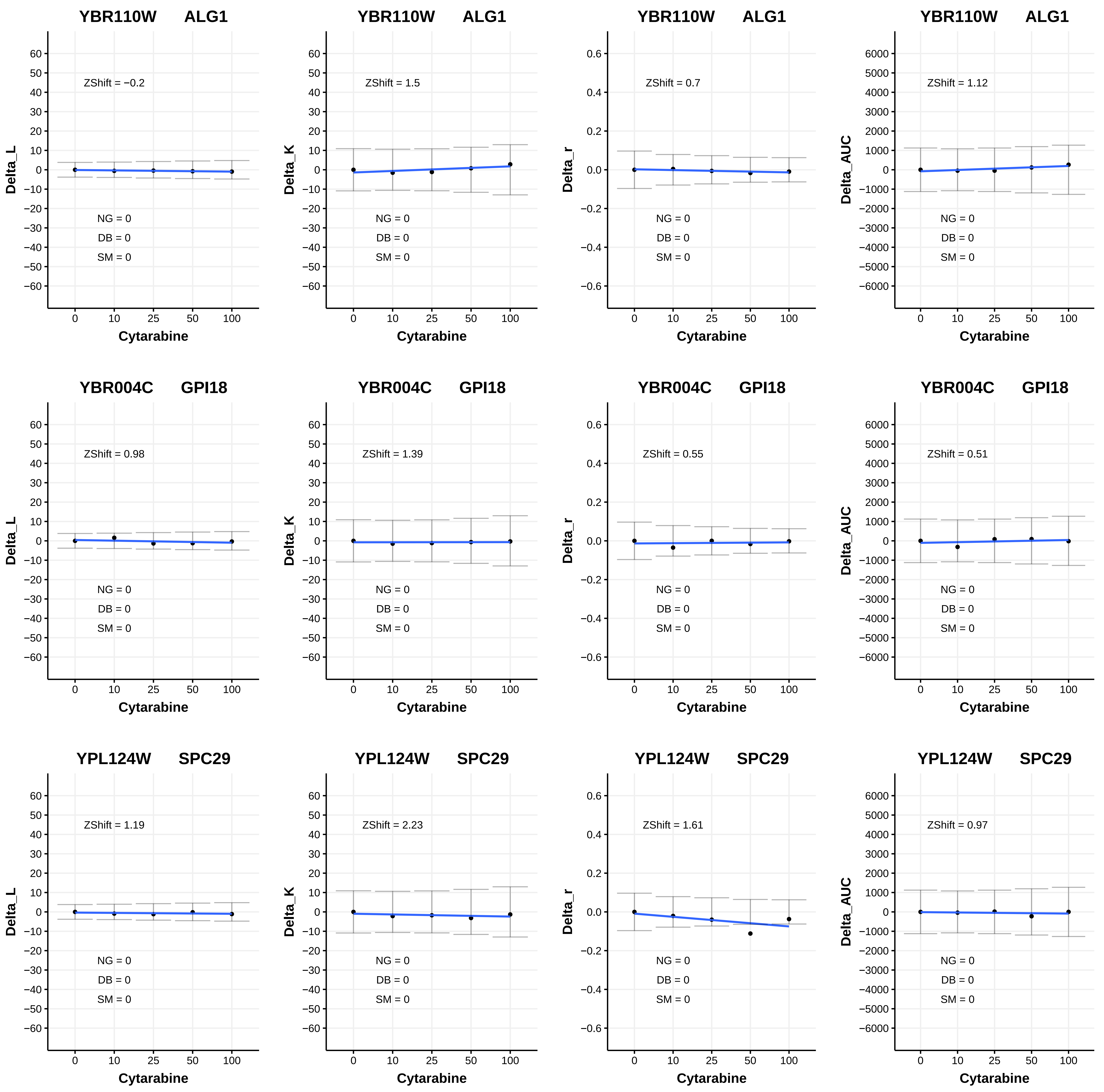
YBR110W ALG1
ZShift = −0.2
NG = 0
DB = 0
SM = 0
Delta_L
Cytarabine
YBR110W ALG1
ZShift = 1.5
NG = 0
DB = 0
SM = 0
Delta_K
Cytarabine
YBR110W ALG1
ZShift = 0.7
NG = 0
DB = 0
SM = 0
Delta_r
Cytarabine
YBR110W ALG1
ZShift = 1.12
NG = 0
DB = 0
SM = 0
Delta_AUC
Cytarabine
YBR004C GPI18
ZShift = 0.98
NG = 0
DB = 0
SM = 0
Delta_L
Cytarabine
YBR004C GPI18
ZShift = 1.39
NG = 0
DB = 0
SM = 0
Delta_K
Cytarabine
YBR004C GPI18
ZShift = 0.55
NG = 0
DB = 0
SM = 0
Delta_r
Cytarabine
YBR004C GPI18
ZShift = 0.51
NG = 0
DB = 0
SM = 0
Delta_AUC
Cytarabine
YPL124W SPC29
ZShift = 1.19
NG = 0
DB = 0
SM = 0
Delta_L
Cytarabine
YPL124W SPC29
ZShift = 2.23
NG = 0
DB = 0
SM = 0
Delta_K
Cytarabine
YPL124W SPC29
ZShift = 1.61
NG = 0
DB = 0
SM = 0
Delta_r
Cytarabine
YPL124W SPC29
ZShift = 0.97
NG = 0
DB = 0
SM = 0
Delta_AUC
Cytarabine

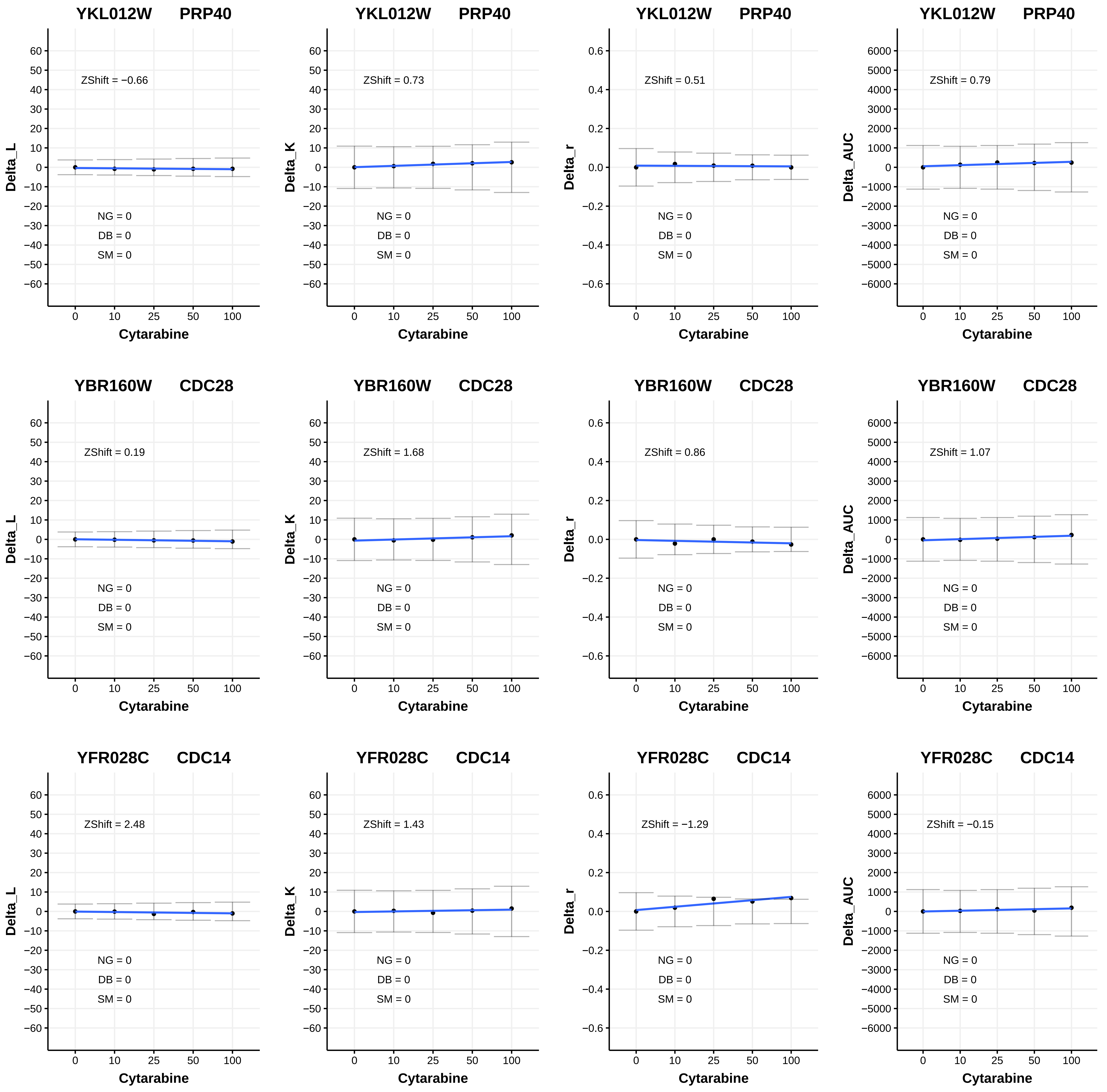
YKL012W PRP40
ZShift = −0.66
NG = 0
DB = 0
SM = 0
Delta_L
Cytarabine
YKL012W PRP40
ZShift = 0.73
NG = 0
DB = 0
SM = 0
Delta_K
Cytarabine
YKL012W PRP40
ZShift = 0.51
NG = 0
DB = 0
SM = 0
Delta_r
Cytarabine
YKL012W PRP40
ZShift = 0.79
NG = 0
DB = 0
SM = 0
Delta_AUC
Cytarabine
YBR160W CDC28
ZShift = 0.19
NG = 0
DB = 0
SM = 0
Delta_L
Cytarabine
YBR160W CDC28
ZShift = 1.68
NG = 0
DB = 0
SM = 0
Delta_K
Cytarabine
YBR160W CDC28
ZShift = 0.86
NG = 0
DB = 0
SM = 0
Delta_r
Cytarabine
YBR160W CDC28
ZShift = 1.07
NG = 0
DB = 0
SM = 0
Delta_AUC
Cytarabine
YFR028C CDC14
ZShift = 2.48
NG = 0
DB = 0
SM = 0
Delta_L
Cytarabine
YFR028C CDC14
ZShift = 1.43
NG = 0
DB = 0
SM = 0
Delta_K
Cytarabine
YFR028C CDC14
ZShift = −1.29
NG = 0
DB = 0
SM = 0
Delta_r
Cytarabine
YFR028C CDC14
ZShift = −0.15
NG = 0
DB = 0
SM = 0
Delta_AUC
Cytarabine

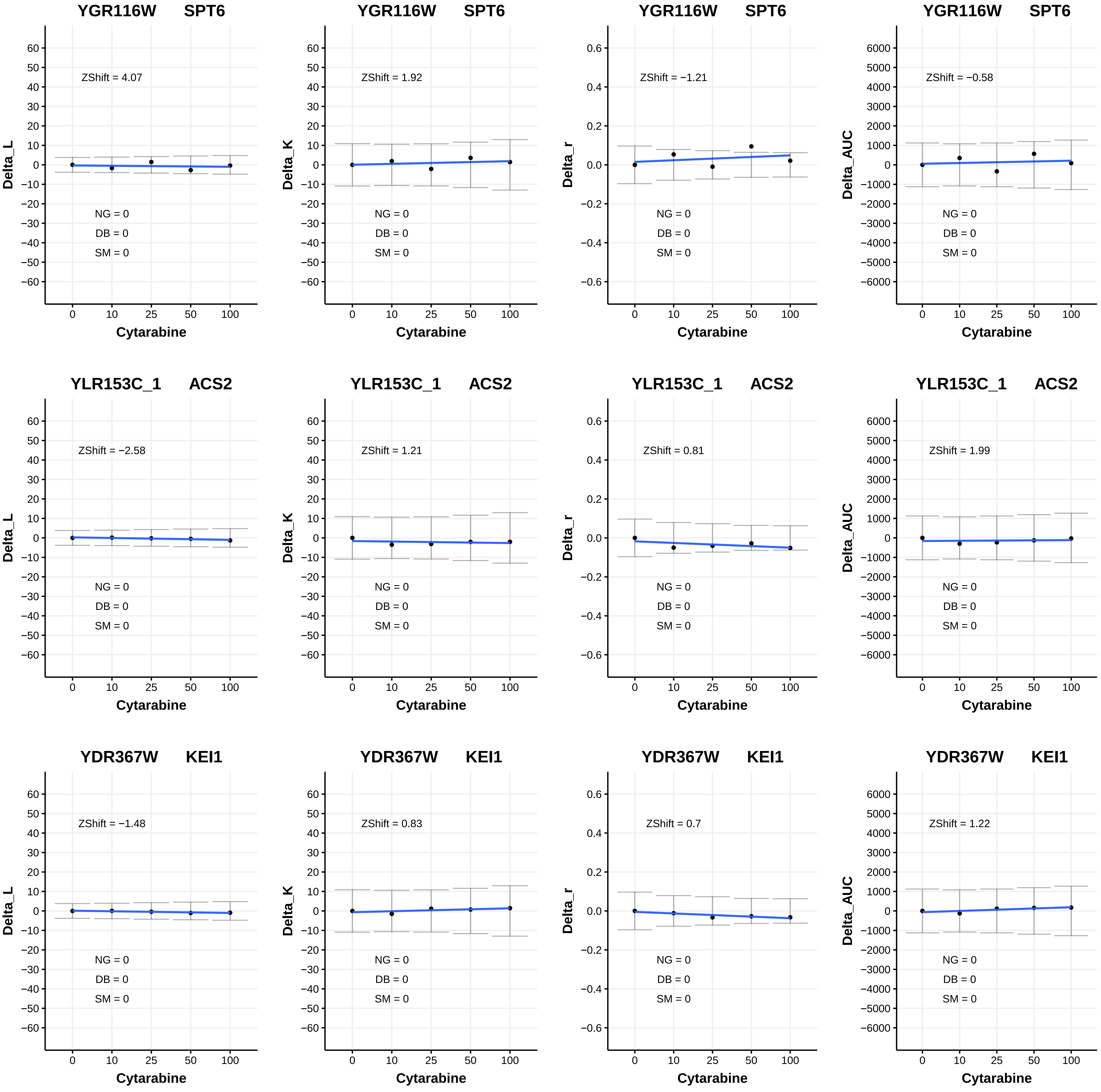
YGR116W SPT6
ZShift = 4.07
NG = 0
DB = 0
SM = 0
Delta_L
Cytarabine
YGR116W SPT6
ZShift = 1.92
NG = 0
DB = 0
SM = 0
Delta_K
Cytarabine
YGR116W SPT6
ZShift = −1.21
NG = 0
DB = 0
SM = 0
Delta_r
Cytarabine
YGR116W SPT6
ZShift = −0.58
NG = 0
DB = 0
SM = 0
Delta_AUC
Cytarabine
YLR153C_1 ACS2
ZShift = −2.58
NG = 0
DB = 0
SM = 0
Delta_L
Cytarabine
YLR153C_1 ACS2
ZShift = 1.21
NG = 0
DB = 0
SM = 0
Delta_K
Cytarabine
YLR153C_1 ACS2
ZShift = 0.81
NG = 0
DB = 0
SM = 0
Delta_r
Cytarabine
YLR153C_1 ACS2
ZShift = 1.99
NG = 0
DB = 0
SM = 0
Delta_AUC
Cytarabine
YDR367W KEI1
ZShift = −1.48
NG = 0
DB = 0
SM = 0
Delta_L
Cytarabine
YDR367W KEI1
ZShift = 0.83
NG = 0
DB = 0
SM = 0
Delta_K
Cytarabine
YDR367W KEI1
ZShift = 0.7
NG = 0
DB = 0
SM = 0
Delta_r
Cytarabine
YDR367W KEI1
ZShift = 1.22
NG = 0
DB = 0
SM = 0
Delta_AUC
Cytarabine

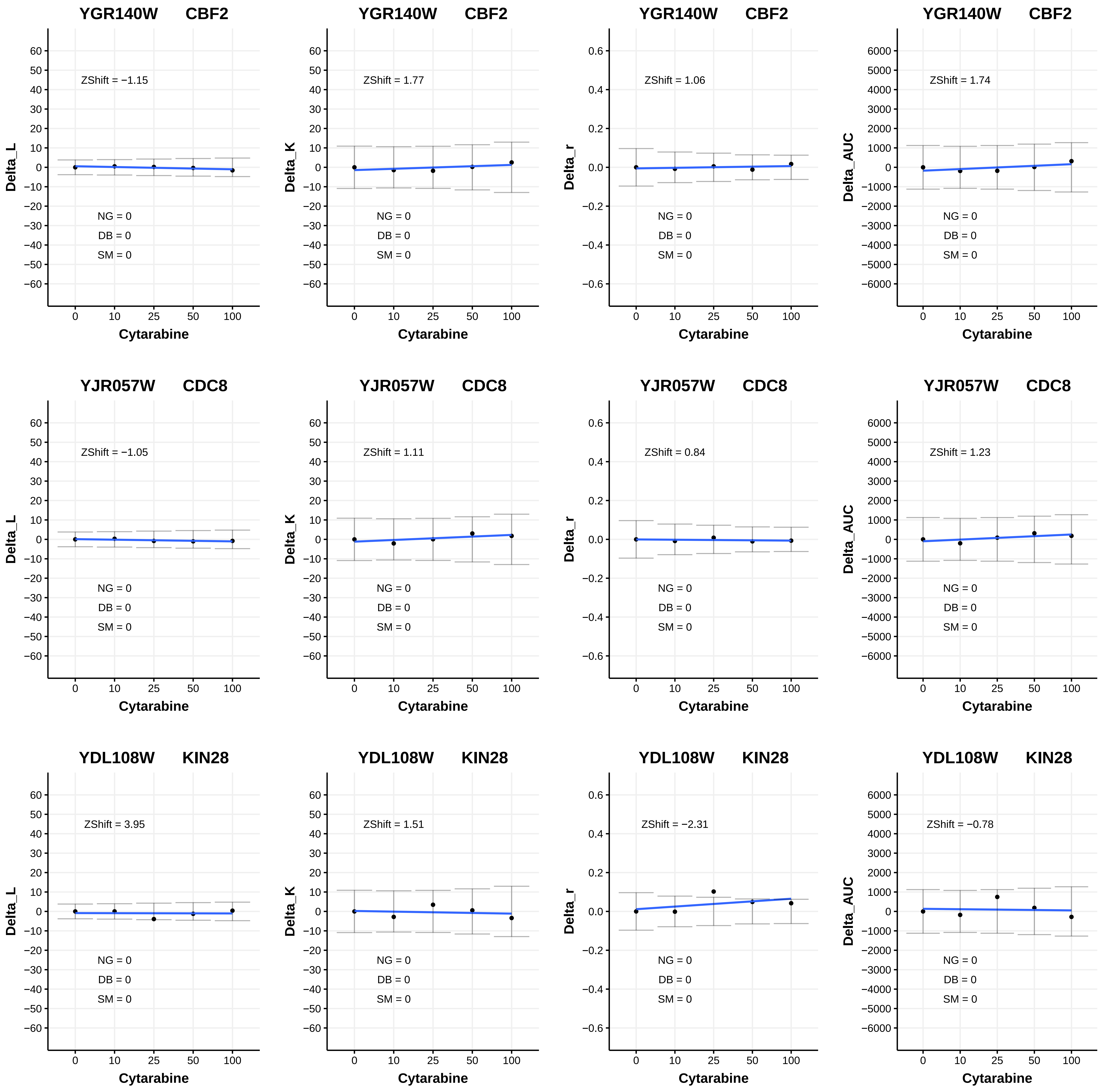
YGR140W CBF2
ZShift = −1.15
NG = 0
DB = 0
SM = 0
Delta_L
Cytarabine
YGR140W CBF2
ZShift = 1.77
NG = 0
DB = 0
SM = 0
Delta_K
Cytarabine
YGR140W CBF2
ZShift = 1.06
NG = 0
DB = 0
SM = 0
Delta_r
Cytarabine
YGR140W CBF2
ZShift = 1.74
NG = 0
DB = 0
SM = 0
Delta_AUC
Cytarabine
YJR057W CDC8
ZShift = −1.05
NG = 0
DB = 0
SM = 0
Delta_L
Cytarabine
YJR057W CDC8
ZShift = 1.11
NG = 0
DB = 0
SM = 0
Delta_K
Cytarabine
YJR057W CDC8
ZShift = 0.84
NG = 0
DB = 0
SM = 0
Delta_r
Cytarabine
YJR057W CDC8
ZShift = 1.23
NG = 0
DB = 0
SM = 0
Delta_AUC
Cytarabine
YDL108W KIN28
ZShift = 3.95
NG = 0
DB = 0
SM = 0
Delta_L
Cytarabine
YDL108W KIN28
ZShift = 1.51
NG = 0
DB = 0
SM = 0
Delta_K
Cytarabine
YDL108W KIN28
ZShift = −2.31
NG = 0
DB = 0
SM = 0
Delta_r
Cytarabine
YDL108W KIN28
ZShift = −0.78
NG = 0
DB = 0
SM = 0
Delta_AUC
Cytarabine

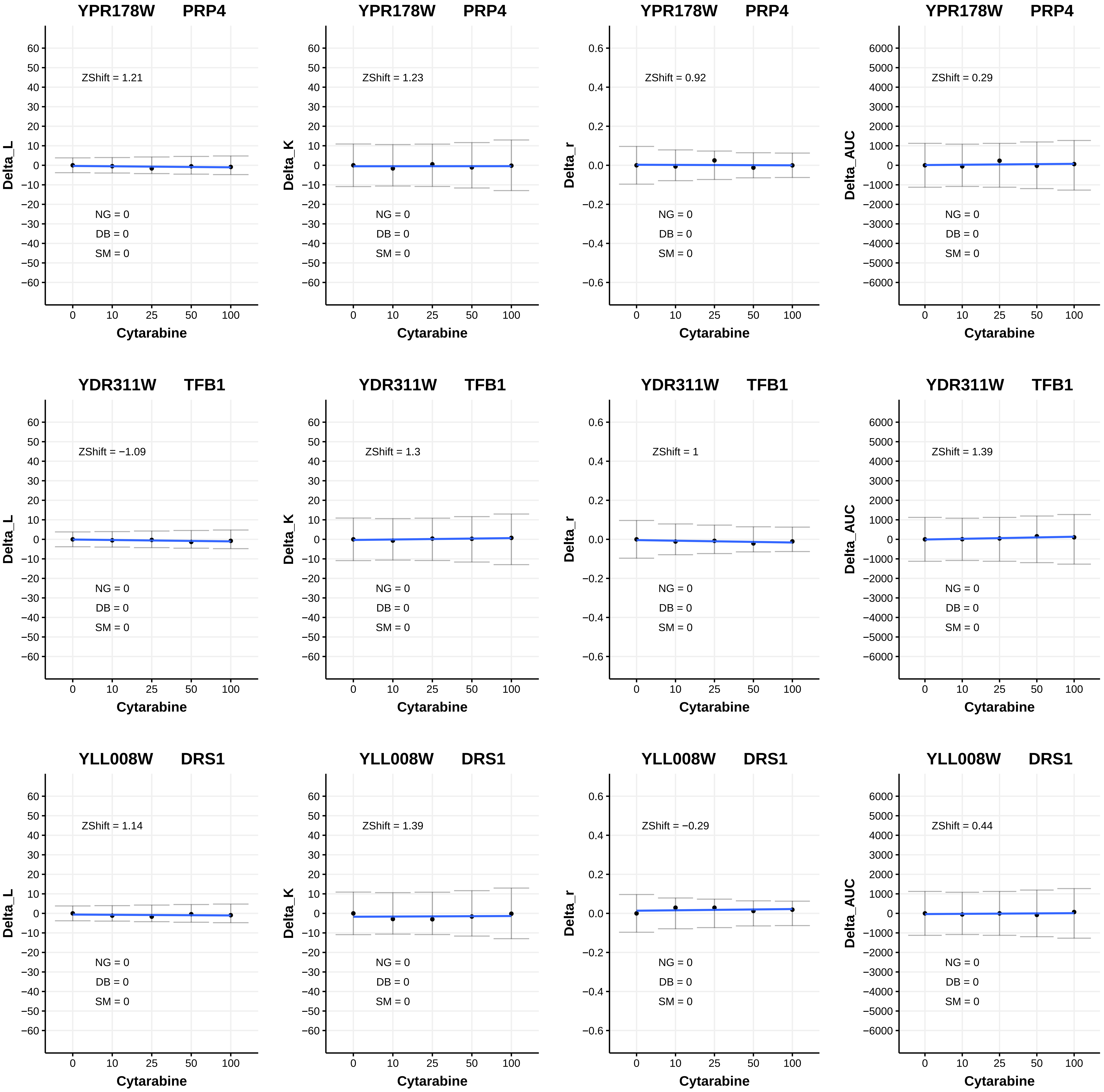

YPR178W PRP4
ZShift = 1.21
ZShift = 1.23
ZShift = 0.92
ZShift = 0.29
YDR311W TFB1
ZShift = −1.09
ZShift = 1.3
ZShift = 1
ZShift = 1.39
YLL008W DRS1
ZShift = 1.14
ZShift = 1.39
ZShift = −0.29
ZShift = 0.44
NG = 0
DB = 0
SM = 0
Delta_L
Delta_K
Delta_r
Delta_AUC
Cytarabine
0
10
25
50
100

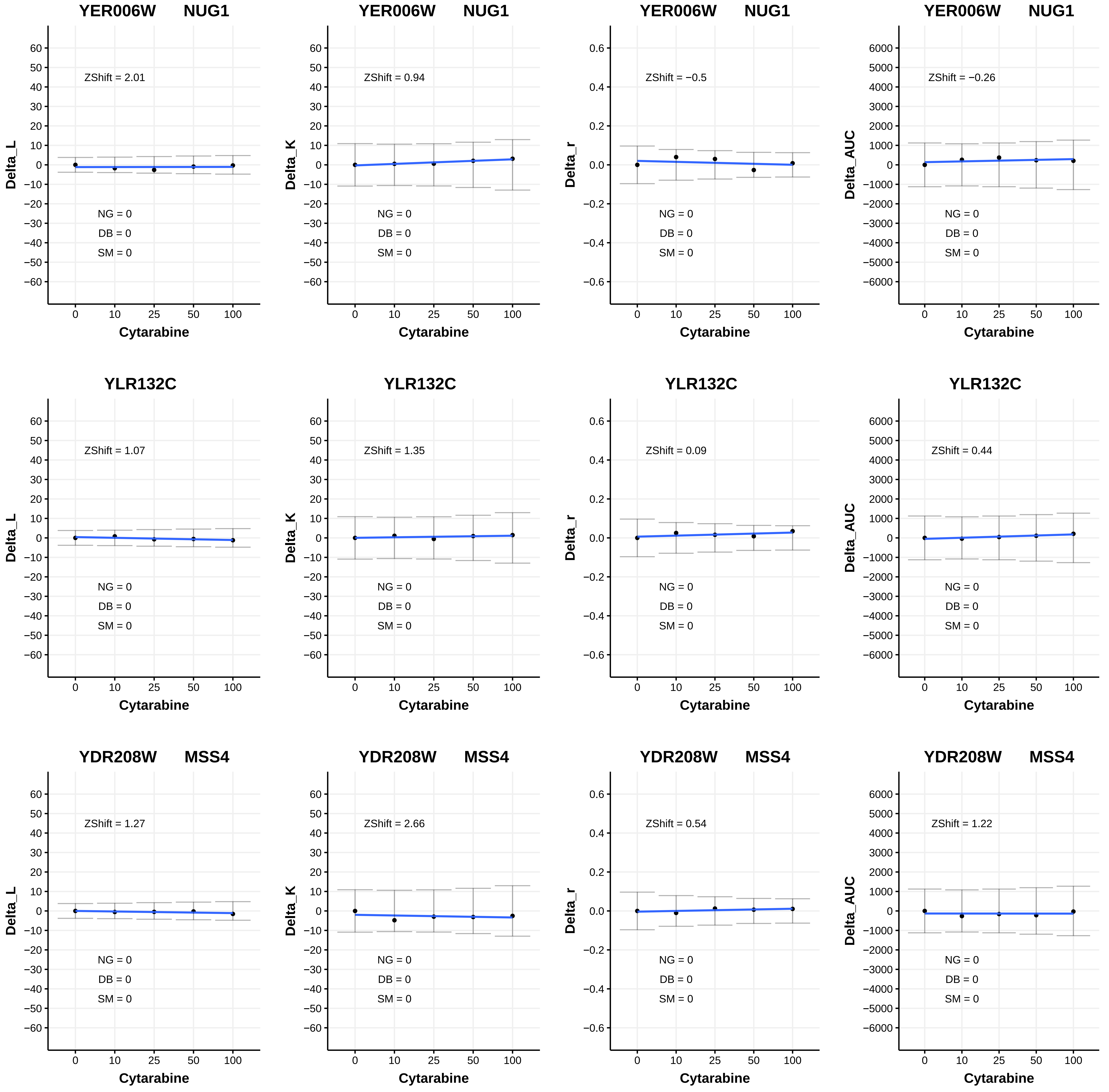

YER006W NUG1
ZShift = 2.01
NG = 0
DB = 0
SM = 0
Delta_L
Cytarabine
YER006W NUG1
ZShift = 0.94
NG = 0
DB = 0
SM = 0
Delta_K
Cytarabine
YER006W NUG1
ZShift = −0.5
NG = 0
DB = 0
SM = 0
Delta_r
Cytarabine
YER006W NUG1
ZShift = −0.26
NG = 0
DB = 0
SM = 0
Delta_AUC
Cytarabine
YLR132C
ZShift = 1.07
NG = 0
DB = 0
SM = 0
Delta_L
Cytarabine
YLR132C
ZShift = 1.35
NG = 0
DB = 0
SM = 0
Delta_K
Cytarabine
YLR132C
ZShift = 0.09
NG = 0
DB = 0
SM = 0
Delta_r
Cytarabine
YLR132C
ZShift = 0.44
NG = 0
DB = 0
SM = 0
Delta_AUC
Cytarabine
YDR208W MSS4
ZShift = 1.27
NG = 0
DB = 0
SM = 0
Delta_L
Cytarabine
YDR208W MSS4
ZShift = 2.66
NG = 0
DB = 0
SM = 0
Delta_K
Cytarabine
YDR208W MSS4
ZShift = 0.54
NG = 0
DB = 0
SM = 0
Delta_r
Cytarabine
YDR208W MSS4
ZShift = 1.22
NG = 0
DB = 0
SM = 0
Delta_AUC
Cytarabine

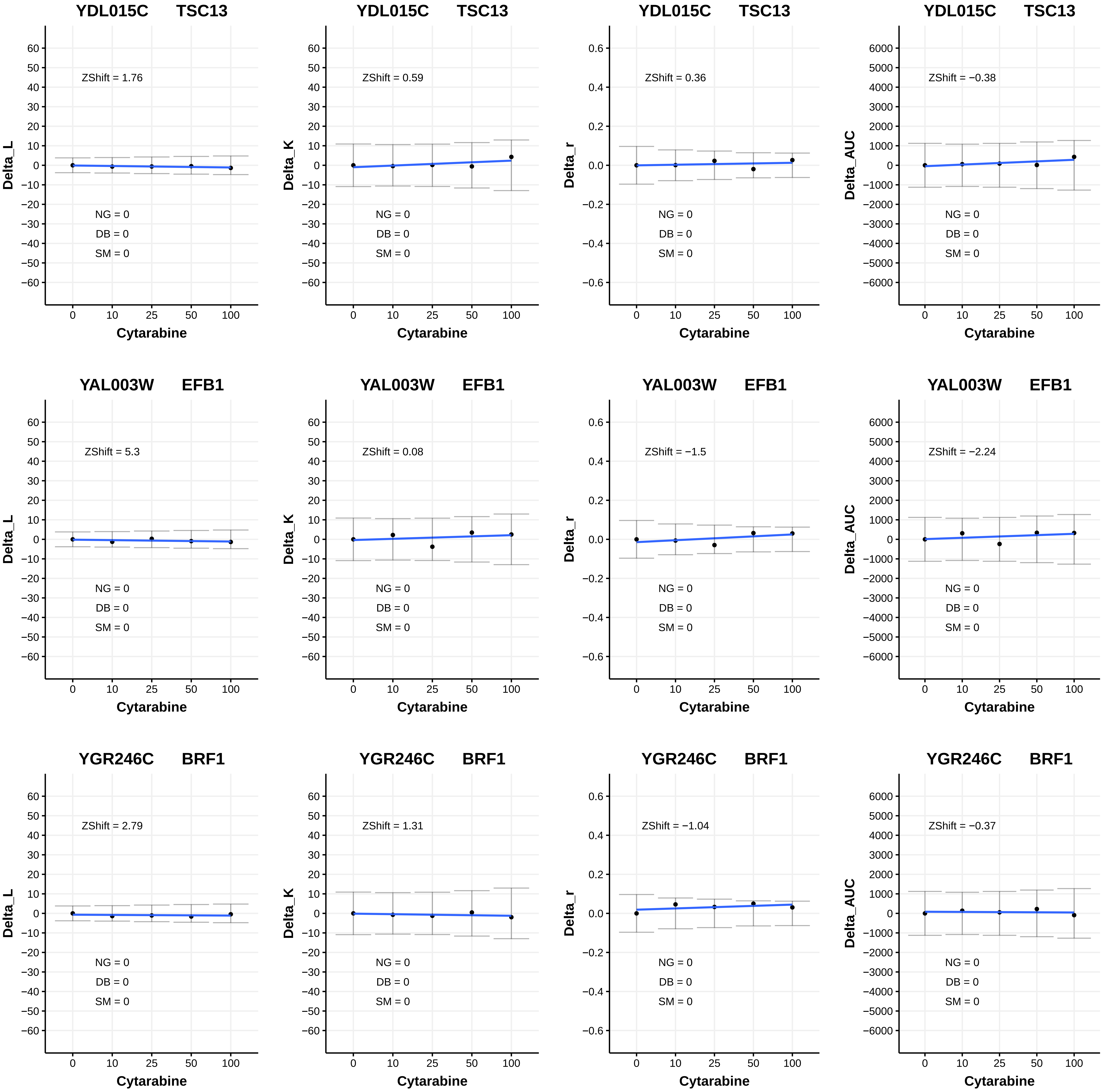
YDL015C TSC13
ZShift = 1.76
NG = 0
DB = 0
SM = 0
Delta_L
Cytarabine
YDL015C TSC13
ZShift = 0.59
NG = 0
DB = 0
SM = 0
Delta_K
Cytarabine
YDL015C TSC13
ZShift = 0.36
NG = 0
DB = 0
SM = 0
Delta_r
Cytarabine
YDL015C TSC13
ZShift = −0.38
NG = 0
DB = 0
SM = 0
Delta_AUC
Cytarabine
YAL003W EFB1
ZShift = 5.3
NG = 0
DB = 0
SM = 0
Delta_L
Cytarabine
YAL003W EFB1
ZShift = 0.08
NG = 0
DB = 0
SM = 0
Delta_K
Cytarabine
YAL003W EFB1
ZShift = −1.5
NG = 0
DB = 0
SM = 0
Delta_r
Cytarabine
YAL003W EFB1
ZShift = −2.24
NG = 0
DB = 0
SM = 0
Delta_AUC
Cytarabine
YGR246C BRF1
ZShift = 2.79
NG = 0
DB = 0
SM = 0
Delta_L
Cytarabine
YGR246C BRF1
ZShift = 1.31
NG = 0
DB = 0
SM = 0
Delta_K
Cytarabine
YGR246C BRF1
ZShift = −1.04
NG = 0
DB = 0
SM = 0
Delta_r
Cytarabine
YGR246C BRF1
ZShift = −0.37
NG = 0
DB = 0
SM = 0
Delta_AUC
Cytarabine

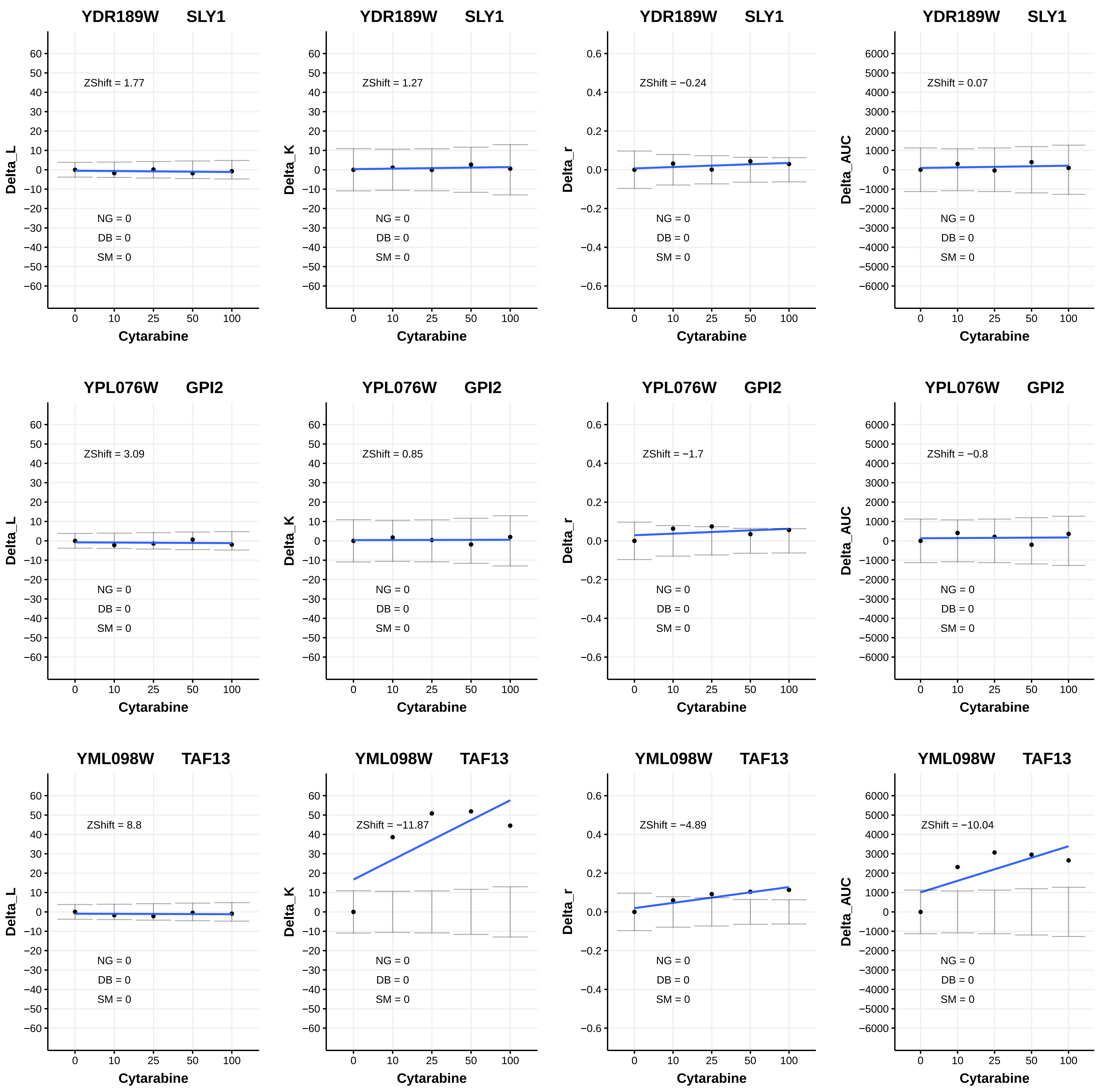

YDR189W SLY1
ZShift = 1.77
ZShift = 1.27
ZShift = −0.24
ZShift = 0.07
YPL076W GPI2
ZShift = 3.09
ZShift = 0.85
ZShift = −1.7
ZShift = −0.8
YML098W TAF13
ZShift = 8.8
ZShift = −11.87
ZShift = −4.89
ZShift = −10.04
NG = 0
DB = 0
SM = 0
Delta_L
Delta_K
Delta_r
Delta_AUC
Cytarabine
0 10 25 50 100

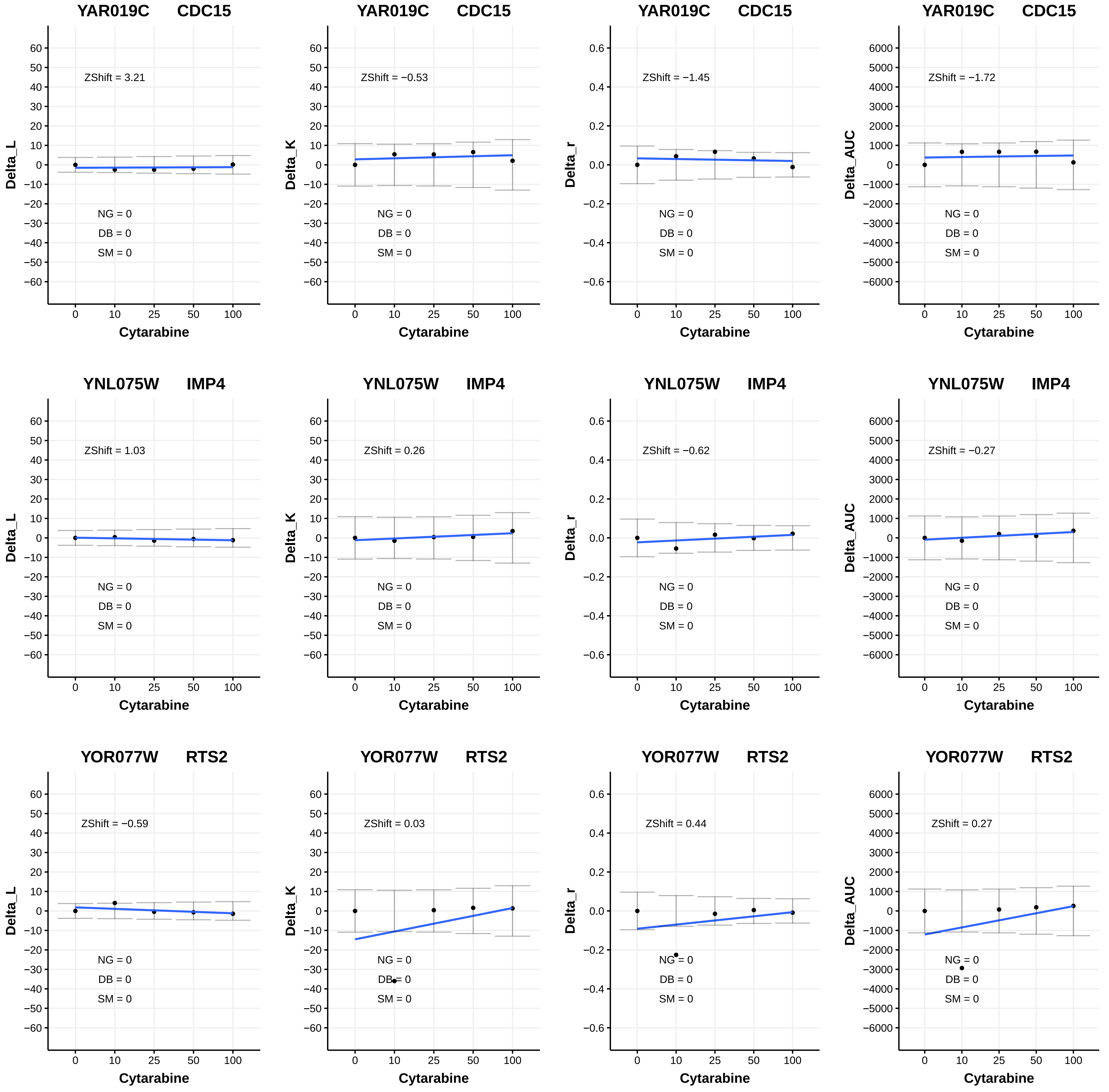

YAR019C CDC15
ZShift = 3.21
ZShift = −0.53
ZShift = −1.45
ZShift = −1.72
YNL075W IMP4
ZShift = 1.03
ZShift = 0.26
ZShift = −0.62
ZShift = −0.27
YOR077W RTS2
ZShift = −0.59
ZShift = 0.03
ZShift = 0.44
ZShift = 0.27
NG = 0
DB = 0
SM = 0
Delta_L
Delta_K
Delta_r
Delta_AUC
Cytarabine

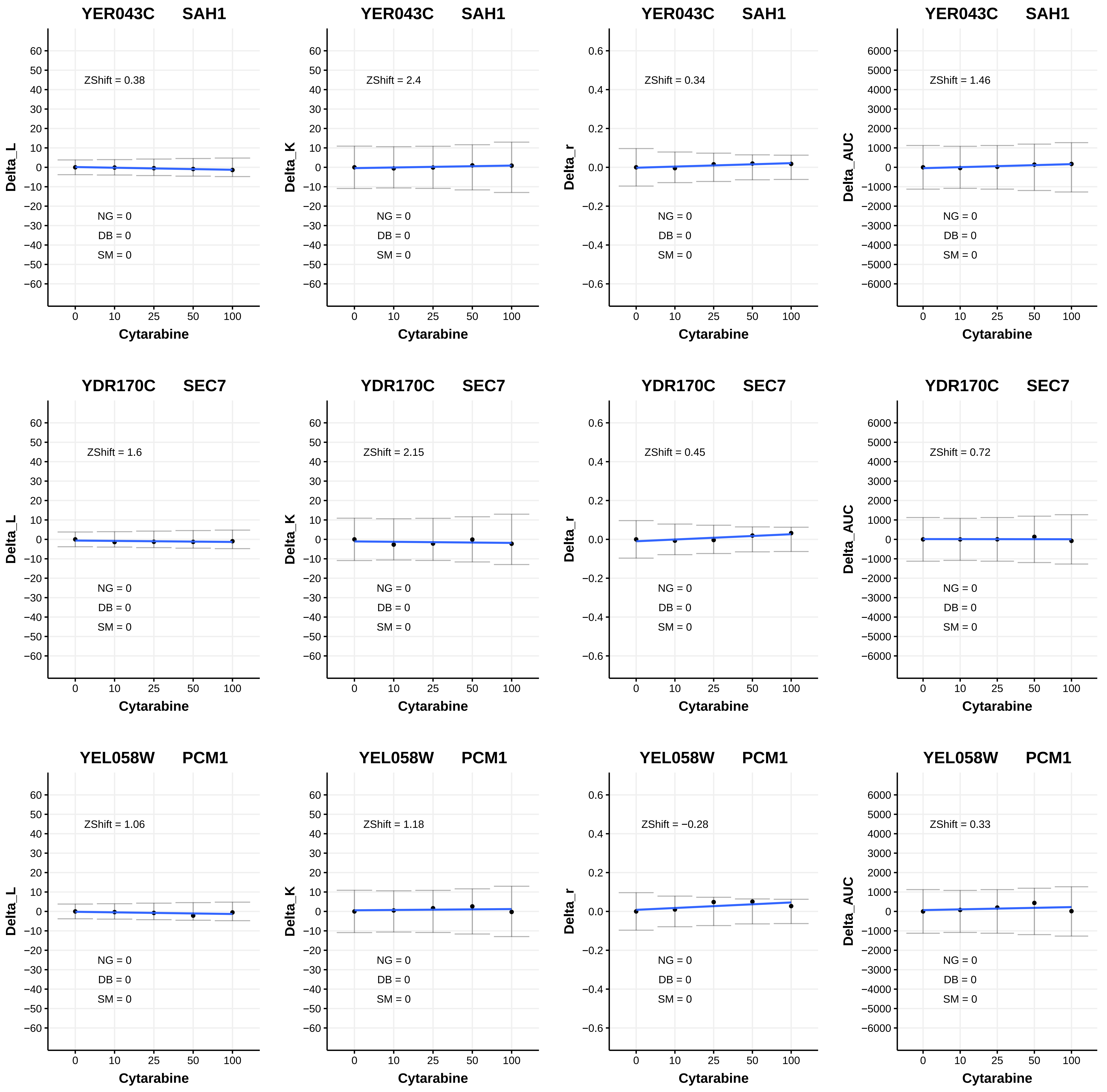

YER043C SAH1
ZShift = 0.38
Delta_L
NG = 0
DB = 0
SM = 0
Cytarabine
YER043C SAH1
ZShift = 2.4
Delta_K
NG = 0
DB = 0
SM = 0
Cytarabine
YER043C SAH1
ZShift = 0.34
Delta_r
NG = 0
DB = 0
SM = 0
Cytarabine
YER043C SAH1
ZShift = 1.46
Delta_AUC
NG = 0
DB = 0
SM = 0
Cytarabine
YDR170C SEC7
ZShift = 1.6
Delta_L
NG = 0
DB = 0
SM = 0
Cytarabine
YDR170C SEC7
ZShift = 2.15
Delta_K
NG = 0
DB = 0
SM = 0
Cytarabine
YDR170C SEC7
ZShift = 0.45
Delta_r
NG = 0
DB = 0
SM = 0
Cytarabine
YDR170C SEC7
ZShift = 0.72
Delta_AUC
NG = 0
DB = 0
SM = 0
Cytarabine
YEL058W PCM1
ZShift = 1.06
Delta_L
NG = 0
DB = 0
SM = 0
Cytarabine
YEL058W PCM1
ZShift = 1.18
Delta_K
NG = 0
DB = 0
SM = 0
Cytarabine
YEL058W PCM1
ZShift = −0.28
Delta_r
NG = 0
DB = 0
SM = 0
Cytarabine
YEL058W PCM1
ZShift = 0.33
Delta_AUC
NG = 0
DB = 0
SM = 0
Cytarabine

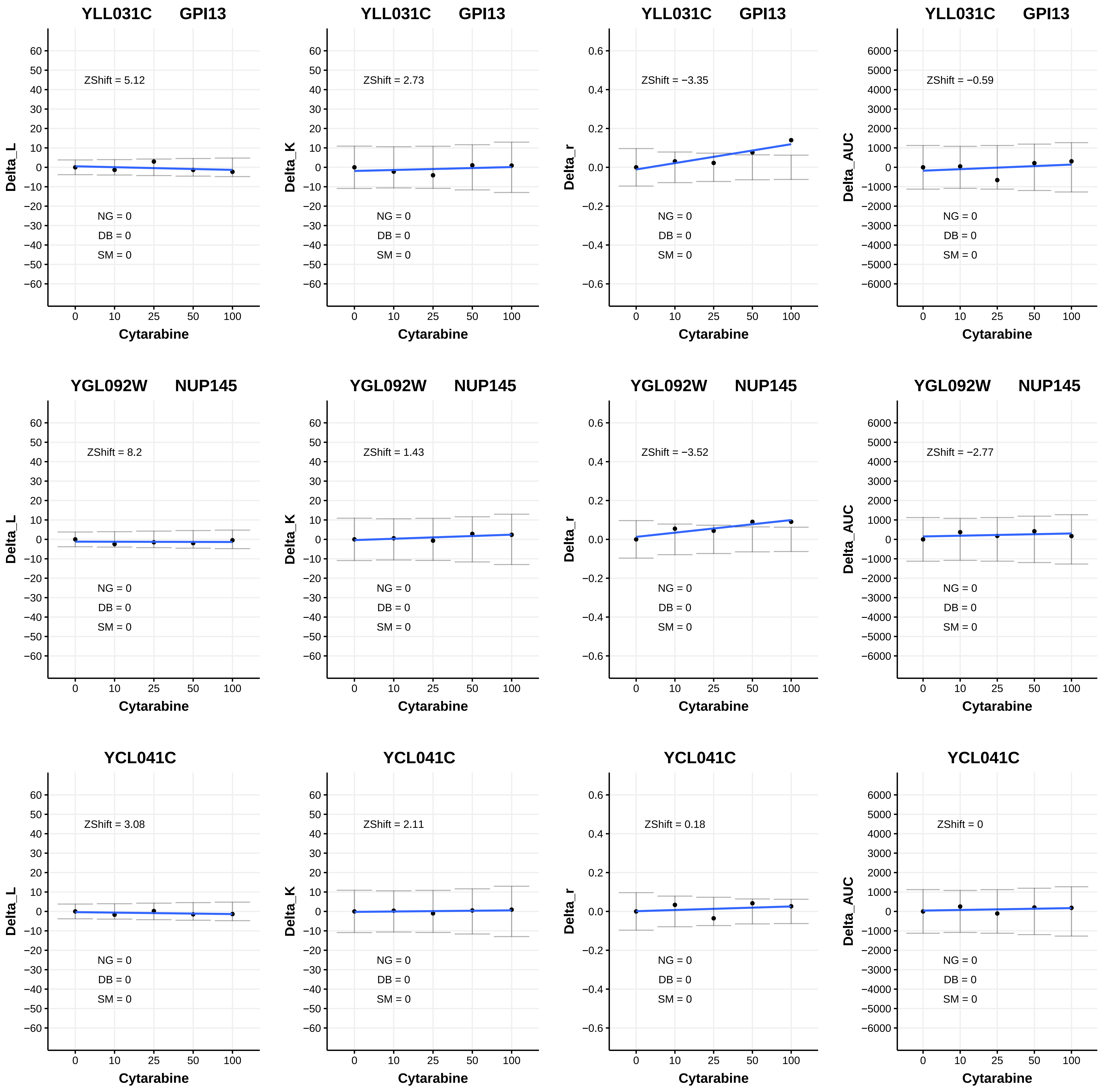
YLL031C GPI13
ZShift = 5.12
ZShift = 2.73
ZShift = −3.35
ZShift = −0.59
YGL092W NUP145
ZShift = 8.2
ZShift = 1.43
ZShift = −3.52
ZShift = −2.77
YCL041C
ZShift = 3.08
ZShift = 2.11
ZShift = 0.18
ZShift = 0
NG = 0
DB = 0
SM = 0
Delta_L
Delta_K
Delta_r
Delta_AUC
Cytarabine
0 10 25 50 100

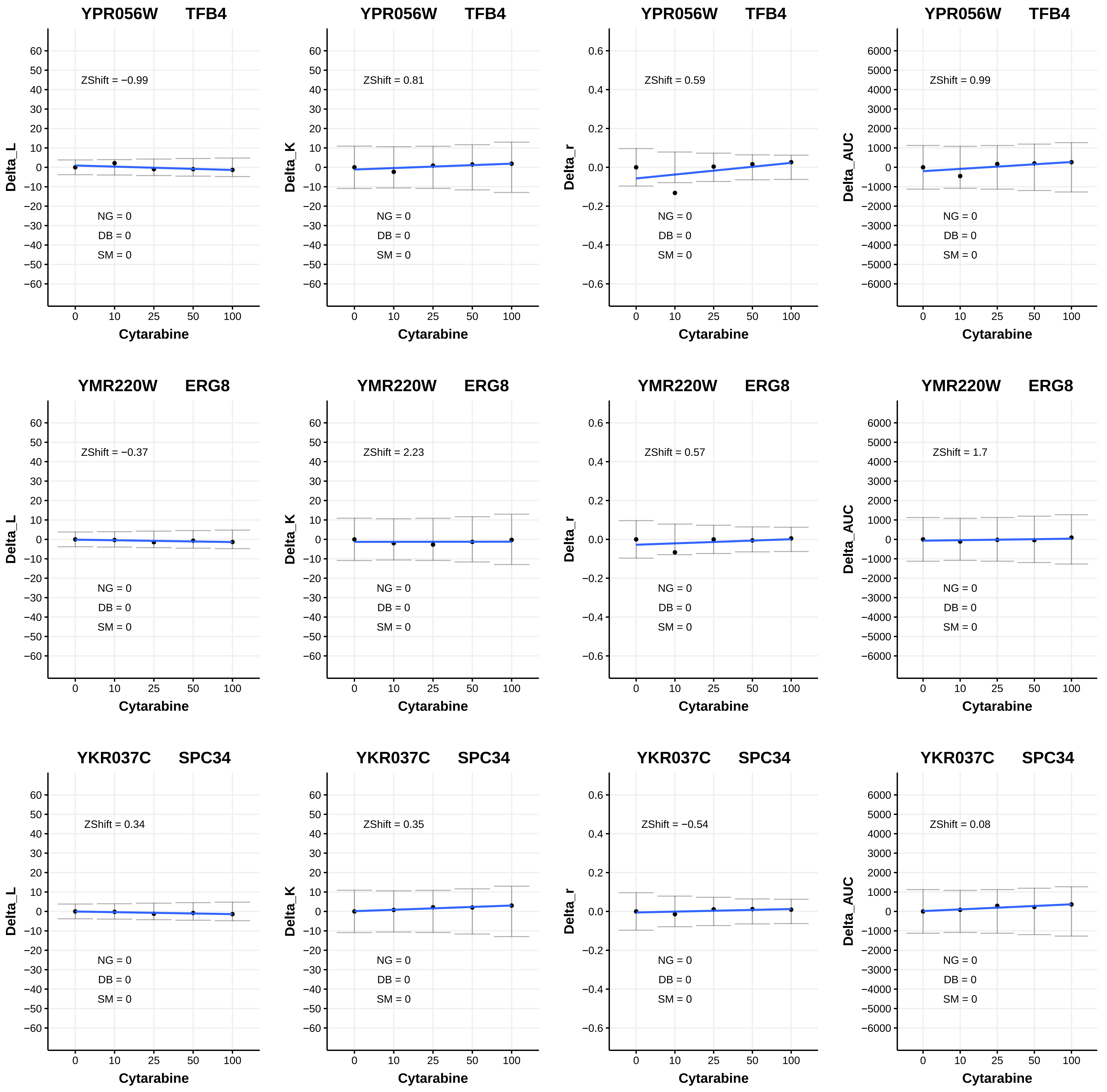
YPR056W TFB4
ZShift = −0.99
ZShift = 0.81
ZShift = 0.59
ZShift = 0.99
YMR220W ERG8
ZShift = −0.37
ZShift = 2.23
ZShift = 0.57
ZShift = 1.7
YKR037C SPC34
ZShift = 0.34
ZShift = 0.35
ZShift = −0.54
ZShift = 0.08
NG = 0
DB = 0
SM = 0
Delta_L
Delta_K
Delta_r
Delta_AUC
Cytarabine
0
10
25
50
100

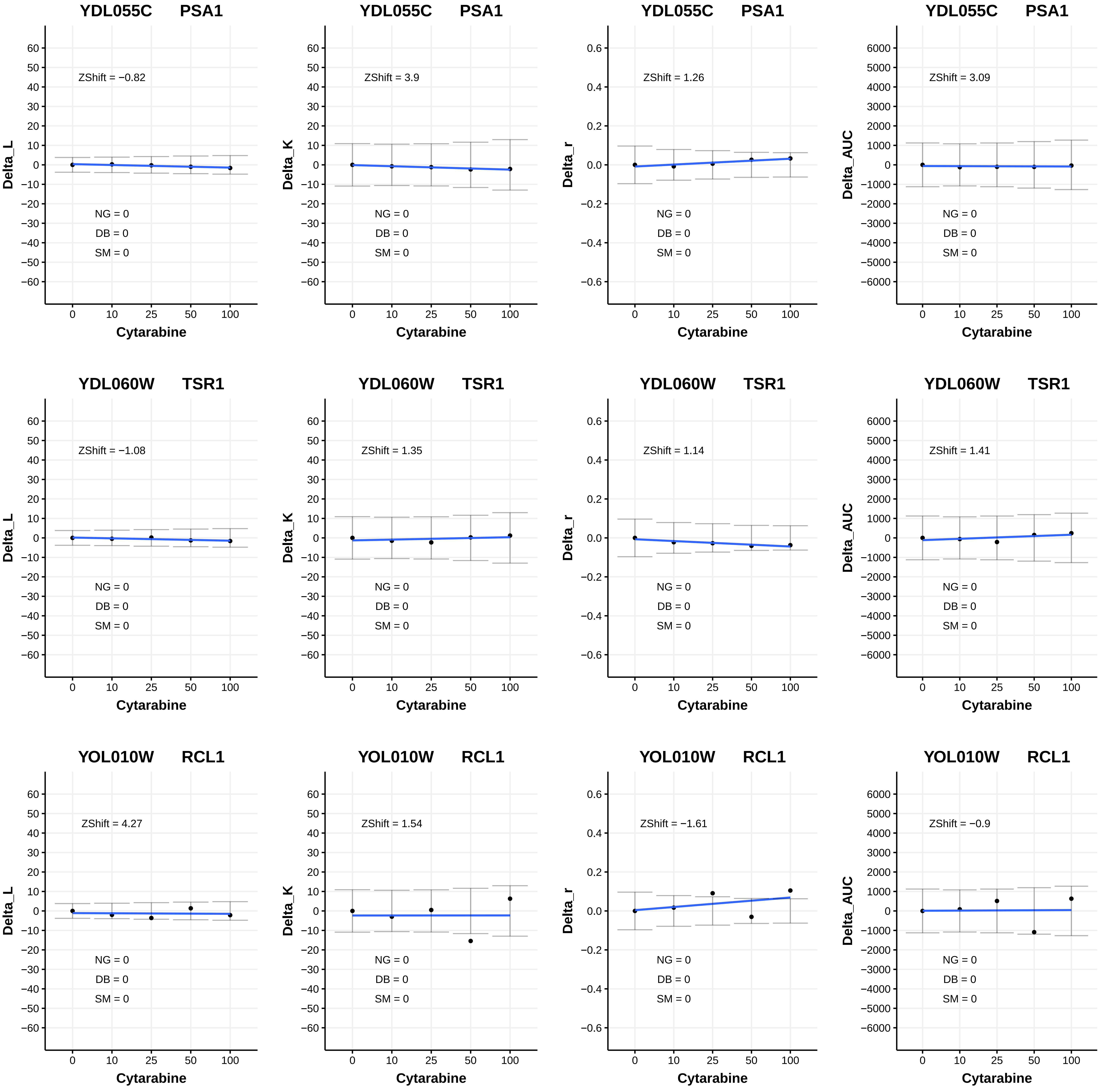
YDL055C PSA1
ZShift = −0.82
NG = 0
DB = 0
SM = 0
Delta_L
Cytarabine
YDL055C PSA1
ZShift = 3.9
NG = 0
DB = 0
SM = 0
Delta_K
Cytarabine
YDL055C PSA1
ZShift = 1.26
NG = 0
DB = 0
SM = 0
Delta_r
Cytarabine
YDL055C PSA1
ZShift = 3.09
NG = 0
DB = 0
SM = 0
Delta_AUC
Cytarabine
YDL060W TSR1
ZShift = −1.08
NG = 0
DB = 0
SM = 0
Delta_L
Cytarabine
YDL060W TSR1
ZShift = 1.35
NG = 0
DB = 0
SM = 0
Delta_K
Cytarabine
YDL060W TSR1
ZShift = 1.14
NG = 0
DB = 0
SM = 0
Delta_r
Cytarabine
YDL060W TSR1
ZShift = 1.41
NG = 0
DB = 0
SM = 0
Delta_AUC
Cytarabine
YOL010W RCL1
ZShift = 4.27
NG = 0
DB = 0
SM = 0
Delta_L
Cytarabine
YOL010W RCL1
ZShift = 1.54
NG = 0
DB = 0
SM = 0
Delta_K
Cytarabine
YOL010W RCL1
ZShift = −1.61
NG = 0
DB = 0
SM = 0
Delta_r
Cytarabine
YOL010W RCL1
ZShift = −0.9
NG = 0
DB = 0
SM = 0
Delta_AUC
Cytarabine

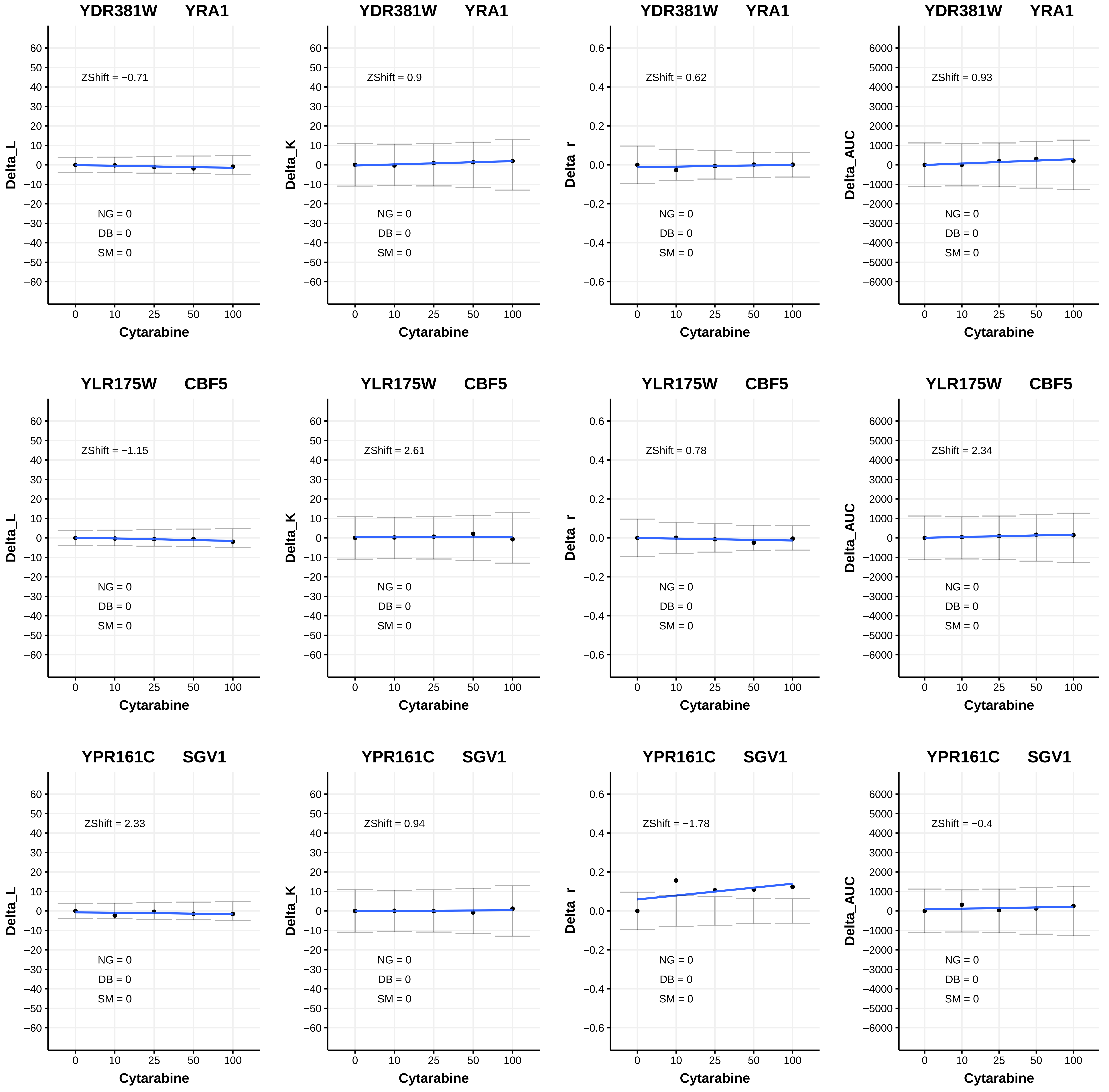

YDR381W YRA1
ZShift = −0.71
Delta_L
NG = 0
DB = 0
SM = 0
Cytarabine
YDR381W YRA1
ZShift = 0.9
Delta_K
NG = 0
DB = 0
SM = 0
Cytarabine
YDR381W YRA1
ZShift = 0.62
Delta_r
NG = 0
DB = 0
SM = 0
Cytarabine
YDR381W YRA1
ZShift = 0.93
Delta_AUC
NG = 0
DB = 0
SM = 0
Cytarabine
YLR175W CBF5
ZShift = −1.15
Delta_L
NG = 0
DB = 0
SM = 0
Cytarabine
YLR175W CBF5
ZShift = 2.61
Delta_K
NG = 0
DB = 0
SM = 0
Cytarabine
YLR175W CBF5
ZShift = 0.78
Delta_r
NG = 0
DB = 0
SM = 0
Cytarabine
YLR175W CBF5
ZShift = 2.34
Delta_AUC
NG = 0
DB = 0
SM = 0
Cytarabine
YPR161C SGV1
ZShift = 2.33
Delta_L
NG = 0
DB = 0
SM = 0
Cytarabine
YPR161C SGV1
ZShift = 0.94
Delta_K
NG = 0
DB = 0
SM = 0
Cytarabine
YPR161C SGV1
ZShift = −1.78
Delta_r
NG = 0
DB = 0
SM = 0
Cytarabine
YPR161C SGV1
ZShift = −0.4
Delta_AUC
NG = 0
DB = 0
SM = 0
Cytarabine

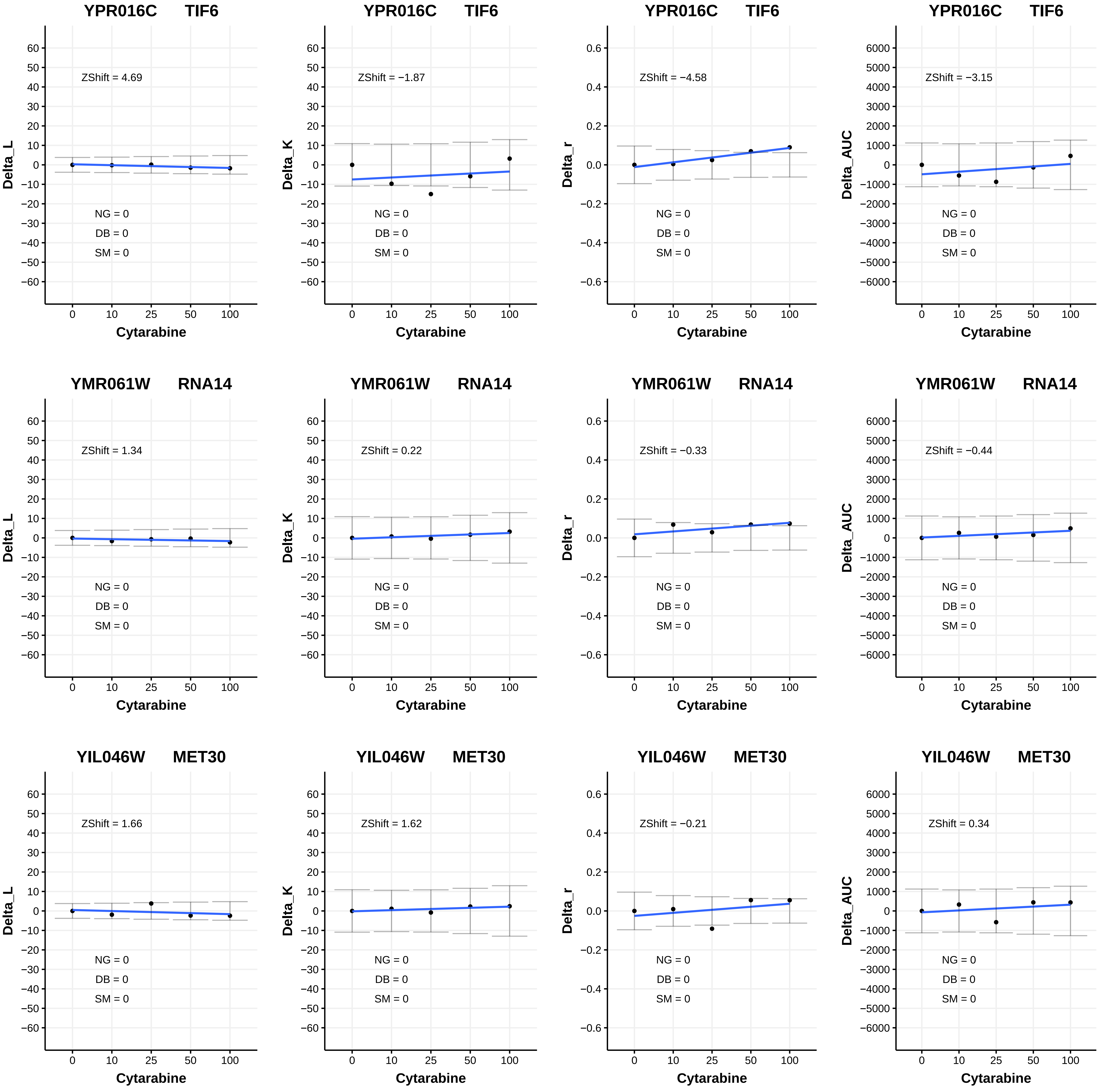
YPR016C TIF6
ZShift = 4.69
Delta_L
NG = 0
DB = 0
SM = 0
Cytarabine
0
10
25
50
100
YPR016C TIF6
ZShift = −1.87
Delta_K
NG = 0
DB = 0
SM = 0
Cytarabine
YPR016C TIF6
ZShift = −4.58
Delta_r
NG = 0
DB = 0
SM = 0
Cytarabine
YPR016C TIF6
ZShift = −3.15
Delta_AUC
NG = 0
DB = 0
SM = 0
Cytarabine
YMR061W RNA14
ZShift = 1.34
Delta_L
NG = 0
DB = 0
SM = 0
Cytarabine
YMR061W RNA14
ZShift = 0.22
Delta_K
NG = 0
DB = 0
SM = 0
Cytarabine
YMR061W RNA14
ZShift = −0.33
Delta_r
NG = 0
DB = 0
SM = 0
Cytarabine
YMR061W RNA14
ZShift = −0.44
Delta_AUC
NG = 0
DB = 0
SM = 0
Cytarabine
YIL046W MET30
ZShift = 1.66
Delta_L
NG = 0
DB = 0
SM = 0
Cytarabine
YIL046W MET30
ZShift = 1.62
Delta_K
NG = 0
DB = 0
SM = 0
Cytarabine
YIL046W MET30
ZShift = −0.21
Delta_r
NG = 0
DB = 0
SM = 0
Cytarabine
YIL046W MET30
ZShift = 0.34
Delta_AUC
NG = 0
DB = 0
SM = 0
Cytarabine

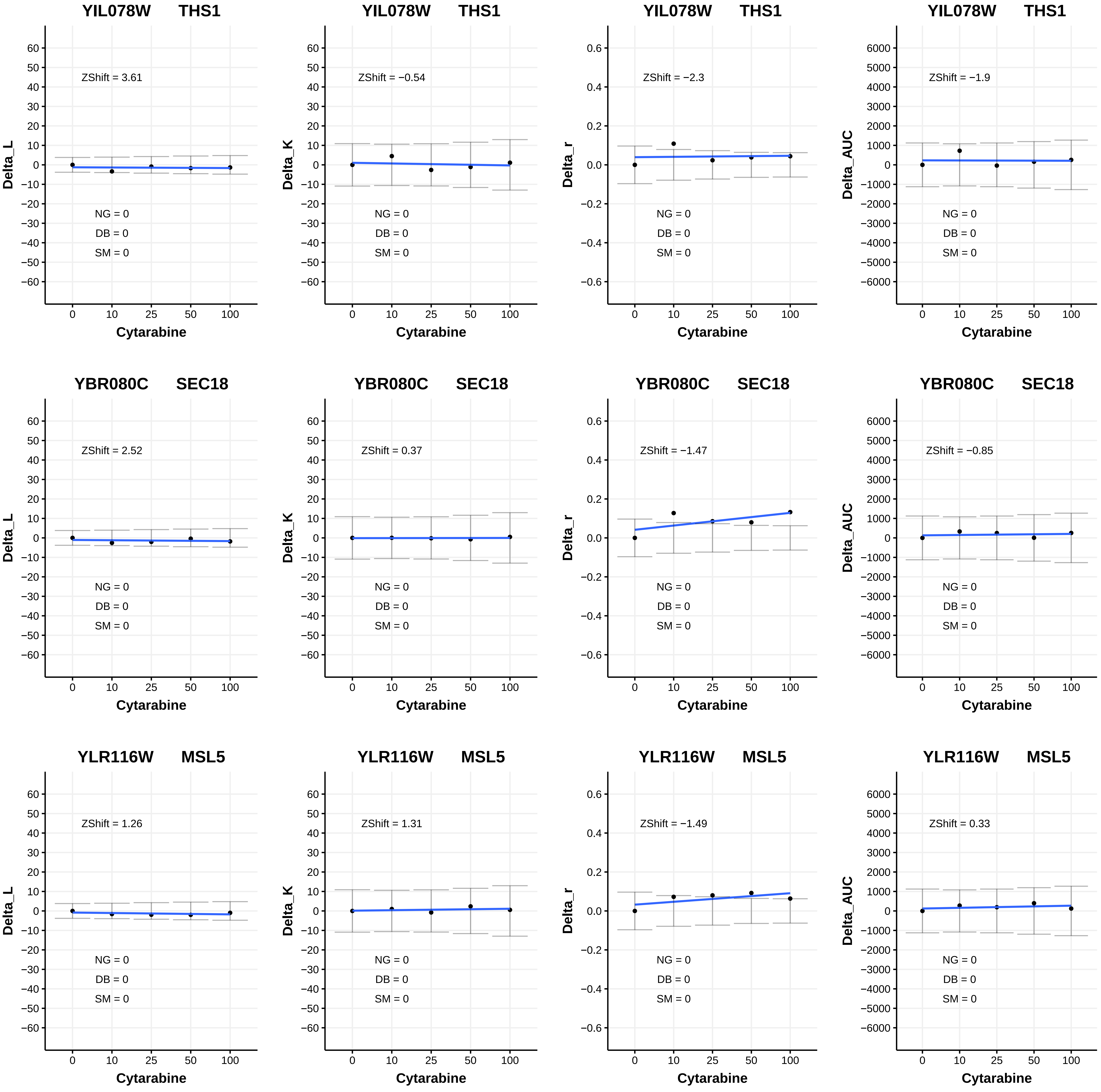
YIL078W THS1
ZShift = 3.61
NG = 0
DB = 0
SM = 0
Delta_L
Cytarabine
YIL078W THS1
ZShift = −0.54
NG = 0
DB = 0
SM = 0
Delta_K
Cytarabine
YIL078W THS1
ZShift = −2.3
NG = 0
DB = 0
SM = 0
Delta_r
Cytarabine
YIL078W THS1
ZShift = −1.9
NG = 0
DB = 0
SM = 0
Delta_AUC
Cytarabine
YBR080C SEC18
ZShift = 2.52
NG = 0
DB = 0
SM = 0
Delta_L
Cytarabine
YBR080C SEC18
ZShift = 0.37
NG = 0
DB = 0
SM = 0
Delta_K
Cytarabine
YBR080C SEC18
ZShift = −1.47
NG = 0
DB = 0
SM = 0
Delta_r
Cytarabine
YBR080C SEC18
ZShift = −0.85
NG = 0
DB = 0
SM = 0
Delta_AUC
Cytarabine
YLR116W MSL5
ZShift = 1.26
NG = 0
DB = 0
SM = 0
Delta_L
Cytarabine
YLR116W MSL5
ZShift = 1.31
NG = 0
DB = 0
SM = 0
Delta_K
Cytarabine
YLR116W MSL5
ZShift = −1.49
NG = 0
DB = 0
SM = 0
Delta_r
Cytarabine
YLR116W MSL5
ZShift = 0.33
NG = 0
DB = 0
SM = 0
Delta_AUC
Cytarabine

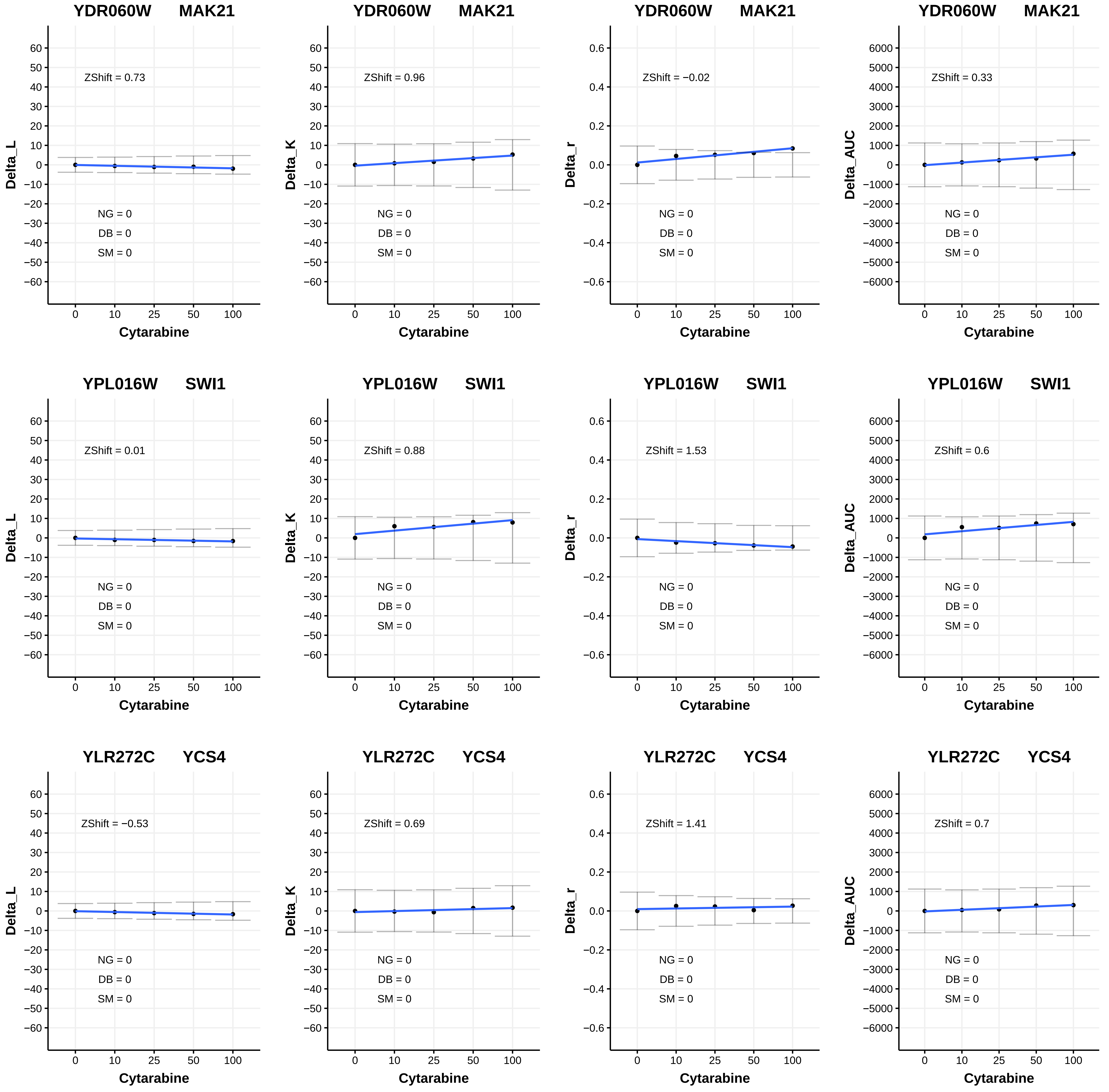
YDR060W MAK21
ZShift = 0.73
NG = 0
DB = 0
SM = 0
Delta_L
Cytarabine
YDR060W MAK21
ZShift = 0.96
NG = 0
DB = 0
SM = 0
Delta_K
Cytarabine
YDR060W MAK21
ZShift = −0.02
NG = 0
DB = 0
SM = 0
Delta_r
Cytarabine
YDR060W MAK21
ZShift = 0.33
NG = 0
DB = 0
SM = 0
Delta_AUC
Cytarabine
YPL016W SWI1
ZShift = 0.01
NG = 0
DB = 0
SM = 0
Delta_L
Cytarabine
YPL016W SWI1
ZShift = 0.88
NG = 0
DB = 0
SM = 0
Delta_K
Cytarabine
YPL016W SWI1
ZShift = 1.53
NG = 0
DB = 0
SM = 0
Delta_r
Cytarabine
YPL016W SWI1
ZShift = 0.6
NG = 0
DB = 0
SM = 0
Delta_AUC
Cytarabine
YLR272C YCS4
ZShift = −0.53
NG = 0
DB = 0
SM = 0
Delta_L
Cytarabine
YLR272C YCS4
ZShift = 0.69
NG = 0
DB = 0
SM = 0
Delta_K
Cytarabine
YLR272C YCS4
ZShift = 1.41
NG = 0
DB = 0
SM = 0
Delta_r
Cytarabine
YLR272C YCS4
ZShift = 0.7
NG = 0
DB = 0
SM = 0
Delta_AUC
Cytarabine

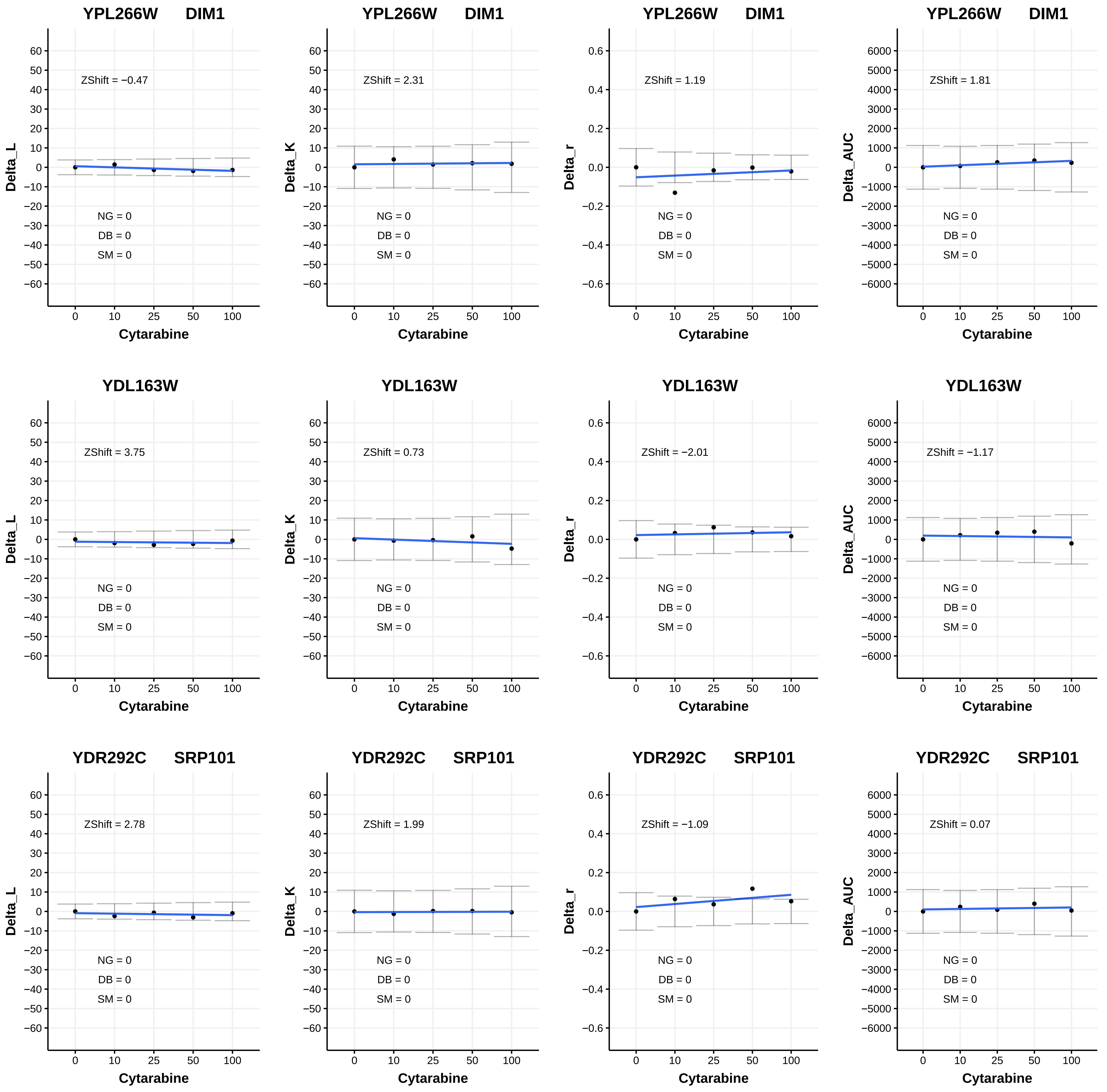
YPL266W DIM1
ZShift = −0.47
ZShift = 2.31
ZShift = 1.19
ZShift = 1.81
YDL163W
ZShift = 3.75
ZShift = 0.73
ZShift = −2.01
ZShift = −1.17
YDR292C SRP101
ZShift = 2.78
ZShift = 1.99
ZShift = −1.09
ZShift = 0.07
NG = 0
DB = 0
SM = 0
Delta_L
Delta_K
Delta_r
Delta_AUC
Cytarabine
0 10 25 50 100

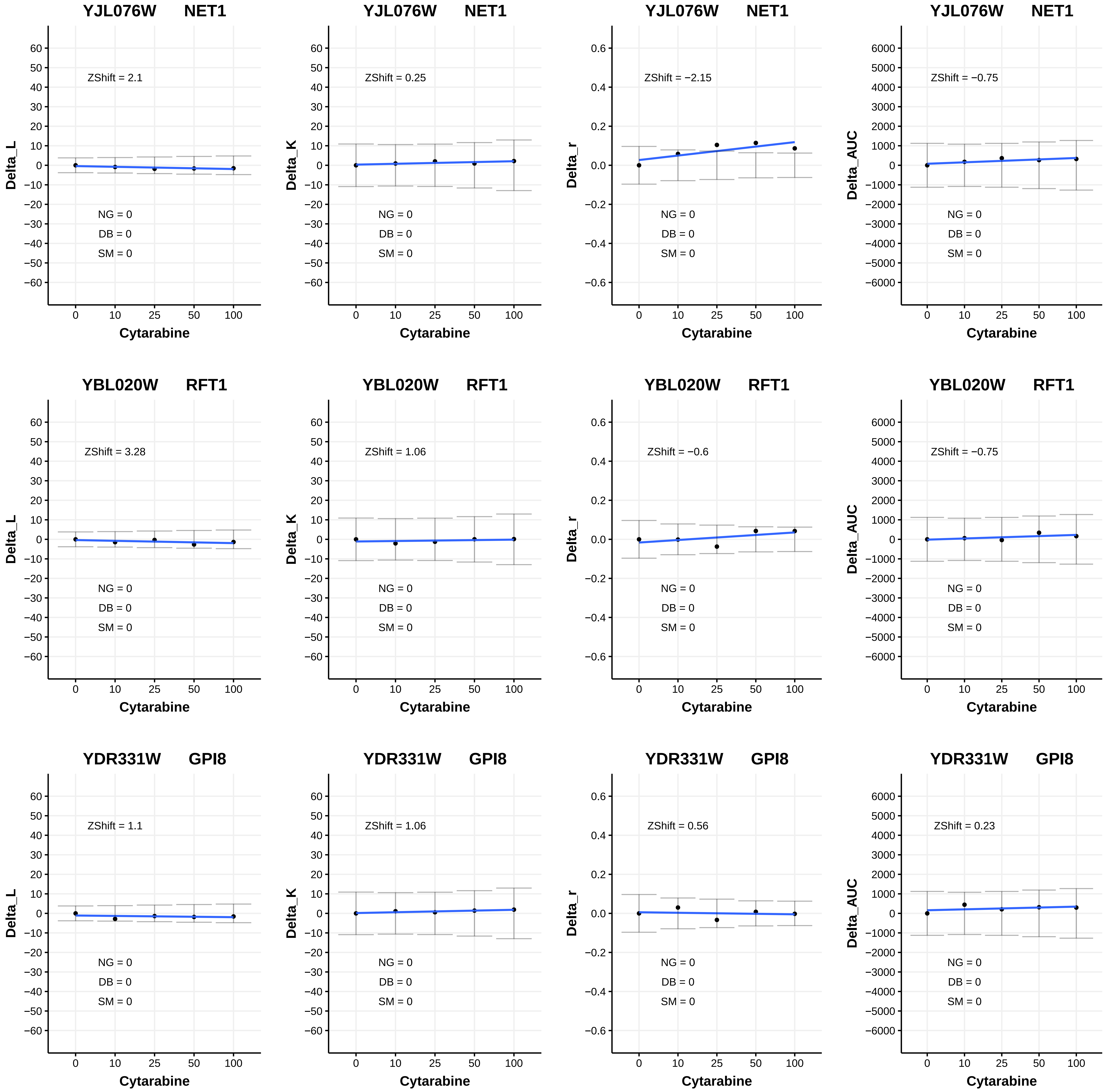

YJL076W NET1
ZShift = 2.1
NG = 0
DB = 0
SM = 0
Delta_L
Cytarabine
YJL076W NET1
ZShift = 0.25
NG = 0
DB = 0
SM = 0
Delta_K
Cytarabine
YJL076W NET1
ZShift = −2.15
NG = 0
DB = 0
SM = 0
Delta_r
Cytarabine
YJL076W NET1
ZShift = −0.75
NG = 0
DB = 0
SM = 0
Delta_AUC
Cytarabine
YBL020W RFT1
ZShift = 3.28
NG = 0
DB = 0
SM = 0
Delta_L
Cytarabine
YBL020W RFT1
ZShift = 1.06
NG = 0
DB = 0
SM = 0
Delta_K
Cytarabine
YBL020W RFT1
ZShift = −0.6
NG = 0
DB = 0
SM = 0
Delta_r
Cytarabine
YBL020W RFT1
ZShift = −0.75
NG = 0
DB = 0
SM = 0
Delta_AUC
Cytarabine
YDR331W GPI8
ZShift = 1.1
NG = 0
DB = 0
SM = 0
Delta_L
Cytarabine
YDR331W GPI8
ZShift = 1.06
NG = 0
DB = 0
SM = 0
Delta_K
Cytarabine
YDR331W GPI8
ZShift = 0.56
NG = 0
DB = 0
SM = 0
Delta_r
Cytarabine
YDR331W GPI8
ZShift = 0.23
NG = 0
DB = 0
SM = 0
Delta_AUC
Cytarabine

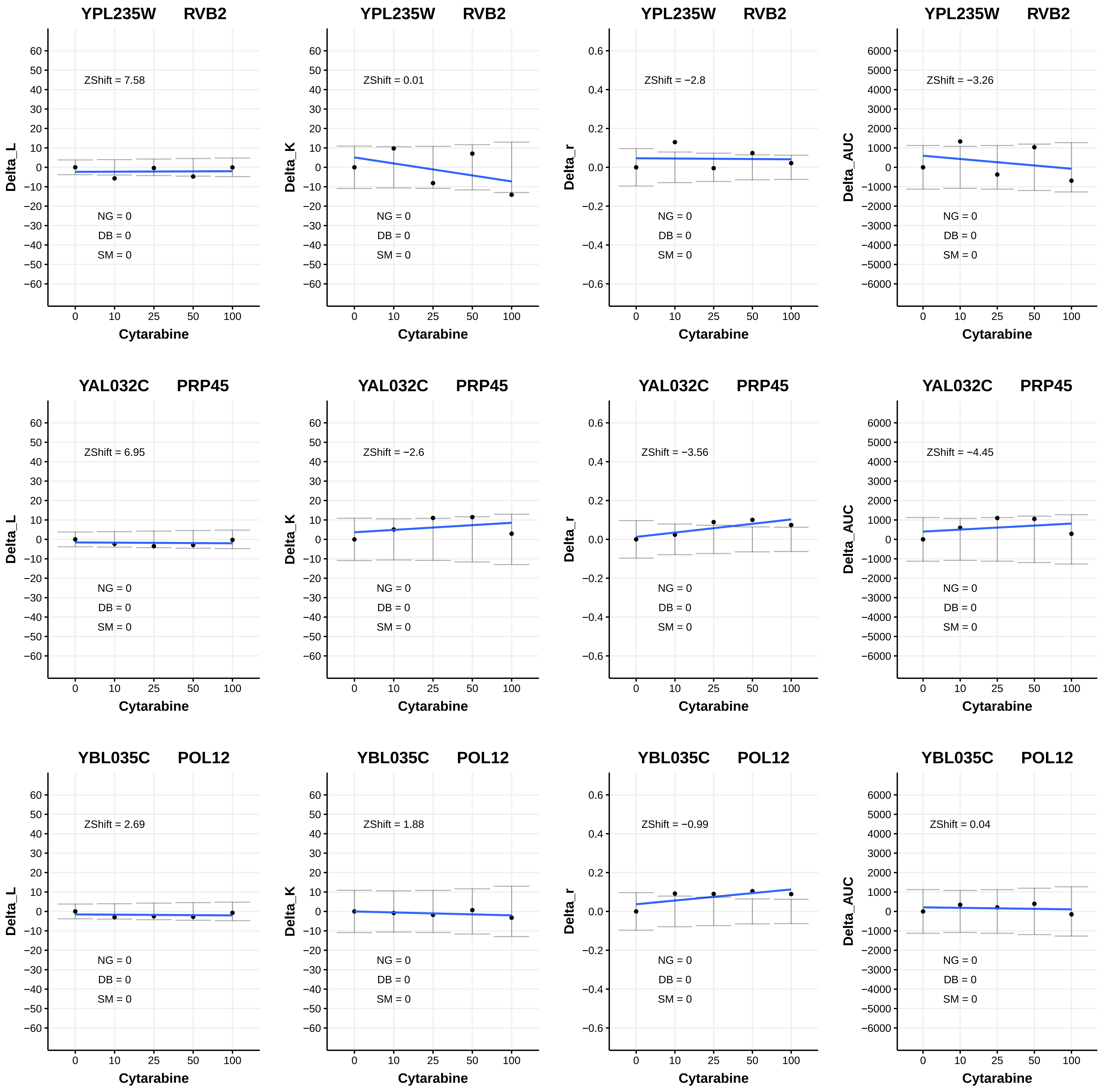
YPL235W RVB2
ZShift = 7.58
NG = 0
DB = 0
SM = 0
Delta_L
Cytarabine
YPL235W RVB2
ZShift = 0.01
NG = 0
DB = 0
SM = 0
Delta_K
Cytarabine
YPL235W RVB2
ZShift = −2.8
NG = 0
DB = 0
SM = 0
Delta_r
Cytarabine
YPL235W RVB2
ZShift = −3.26
NG = 0
DB = 0
SM = 0
Delta_AUC
Cytarabine
YAL032C PRP45
ZShift = 6.95
NG = 0
DB = 0
SM = 0
Delta_L
Cytarabine
YAL032C PRP45
ZShift = −2.6
NG = 0
DB = 0
SM = 0
Delta_K
Cytarabine
YAL032C PRP45
ZShift = −3.56
NG = 0
DB = 0
SM = 0
Delta_r
Cytarabine
YAL032C PRP45
ZShift = −4.45
NG = 0
DB = 0
SM = 0
Delta_AUC
Cytarabine
YBL035C POL12
ZShift = 2.69
NG = 0
DB = 0
SM = 0
Delta_L
Cytarabine
YBL035C POL12
ZShift = 1.88
NG = 0
DB = 0
SM = 0
Delta_K
Cytarabine
YBL035C POL12
ZShift = −0.99
NG = 0
DB = 0
SM = 0
Delta_r
Cytarabine
YBL035C POL12
ZShift = 0.04
NG = 0
DB = 0
SM = 0
Delta_AUC
Cytarabine

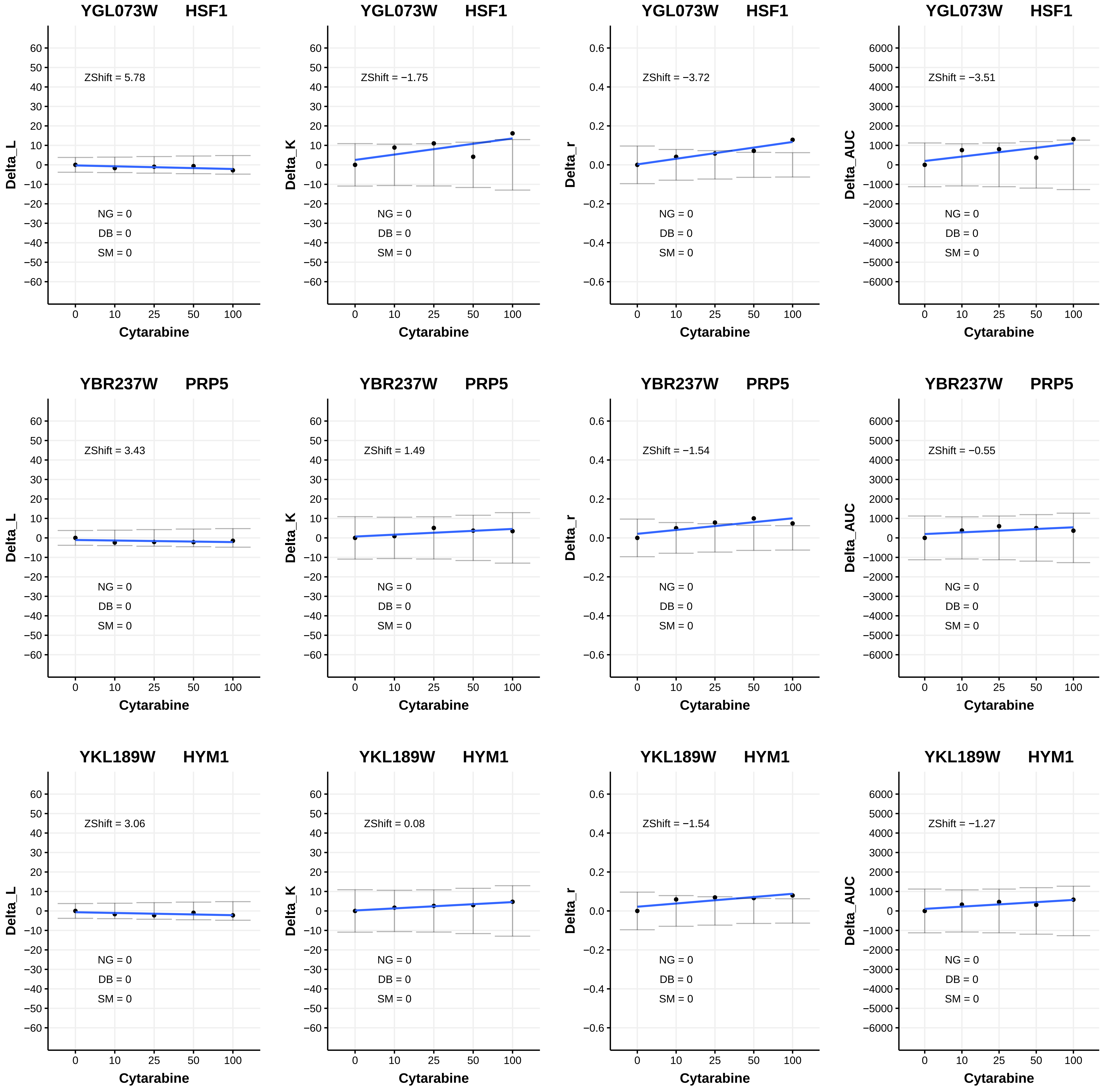
YGL073W HSF1
ZShift = 5.78
NG = 0
DB = 0
SM = 0
Delta_L
Cytarabine
YGL073W HSF1
ZShift = −1.75
NG = 0
DB = 0
SM = 0
Delta_K
Cytarabine
YGL073W HSF1
ZShift = −3.72
NG = 0
DB = 0
SM = 0
Delta_r
Cytarabine
YGL073W HSF1
ZShift = −3.51
NG = 0
DB = 0
SM = 0
Delta_AUC
Cytarabine
YBR237W PRP5
ZShift = 3.43
NG = 0
DB = 0
SM = 0
Delta_L
Cytarabine
YBR237W PRP5
ZShift = 1.49
NG = 0
DB = 0
SM = 0
Delta_K
Cytarabine
YBR237W PRP5
ZShift = −1.54
NG = 0
DB = 0
SM = 0
Delta_r
Cytarabine
YBR237W PRP5
ZShift = −0.55
NG = 0
DB = 0
SM = 0
Delta_AUC
Cytarabine
YKL189W HYM1
ZShift = 3.06
NG = 0
DB = 0
SM = 0
Delta_L
Cytarabine
YKL189W HYM1
ZShift = 0.08
NG = 0
DB = 0
SM = 0
Delta_K
Cytarabine
YKL189W HYM1
ZShift = −1.54
NG = 0
DB = 0
SM = 0
Delta_r
Cytarabine
YKL189W HYM1
ZShift = −1.27
NG = 0
DB = 0
SM = 0
Delta_AUC
Cytarabine

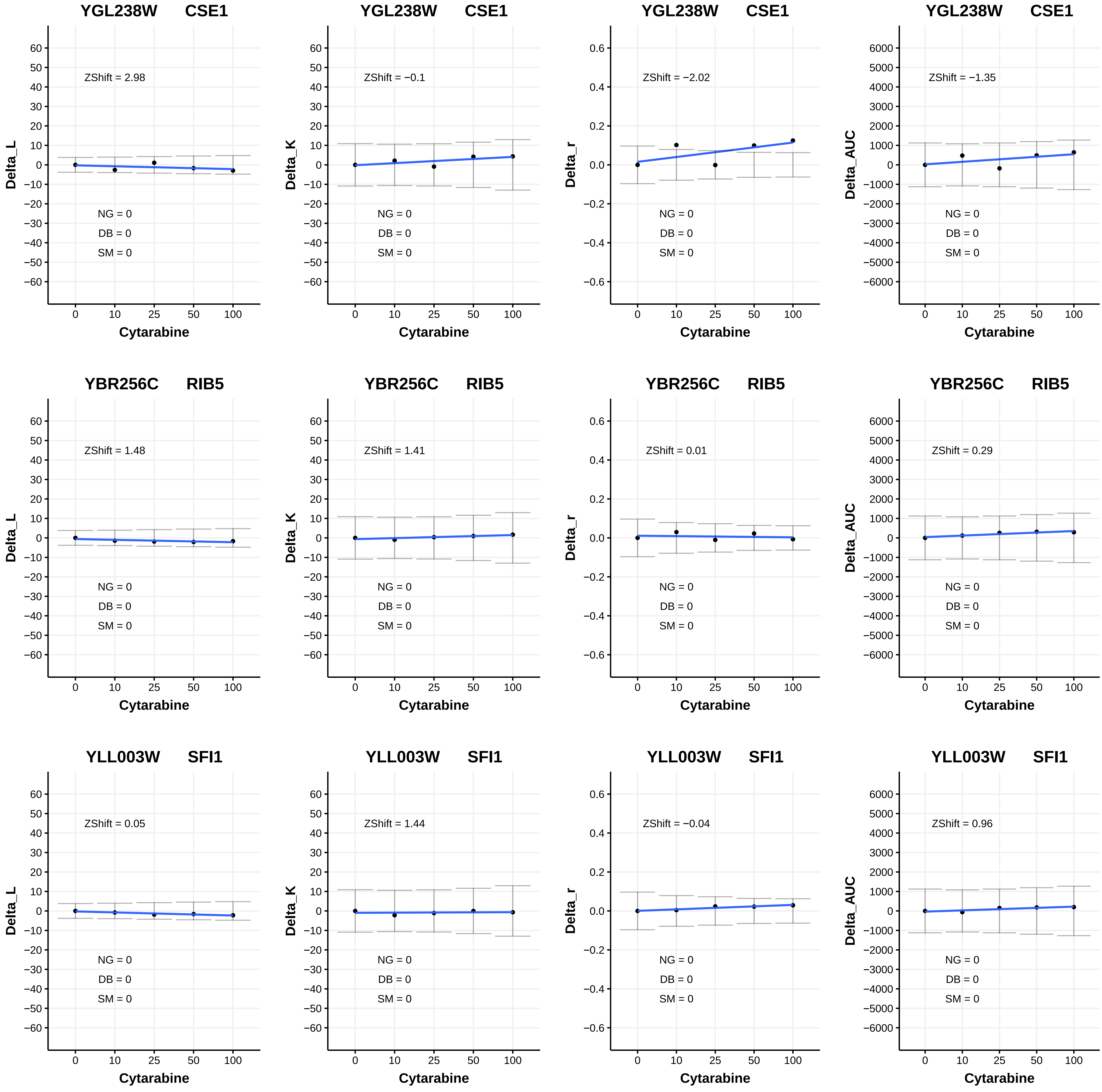

YGL238W CSE1
ZShift = 2.98
Delta_L
NG = 0
DB = 0
SM = 0
Cytarabine
YGL238W CSE1
ZShift = −0.1
Delta_K
NG = 0
DB = 0
SM = 0
Cytarabine
YGL238W CSE1
ZShift = −2.02
Delta_r
NG = 0
DB = 0
SM = 0
Cytarabine
YGL238W CSE1
ZShift = −1.35
Delta_AUC
NG = 0
DB = 0
SM = 0
Cytarabine
YBR256C RIB5
ZShift = 1.48
Delta_L
NG = 0
DB = 0
SM = 0
Cytarabine
YBR256C RIB5
ZShift = 1.41
Delta_K
NG = 0
DB = 0
SM = 0
Cytarabine
YBR256C RIB5
ZShift = 0.01
Delta_r
NG = 0
DB = 0
SM = 0
Cytarabine
YBR256C RIB5
ZShift = 0.29
Delta_AUC
NG = 0
DB = 0
SM = 0
Cytarabine
YLL003W SFI1
ZShift = 0.05
Delta_L
NG = 0
DB = 0
SM = 0
Cytarabine
YLL003W SFI1
ZShift = 1.44
Delta_K
NG = 0
DB = 0
SM = 0
Cytarabine
YLL003W SFI1
ZShift = −0.04
Delta_r
NG = 0
DB = 0
SM = 0
Cytarabine
YLL003W SFI1
ZShift = 0.96
Delta_AUC
NG = 0
DB = 0
SM = 0
Cytarabine
0 10 25 50 100

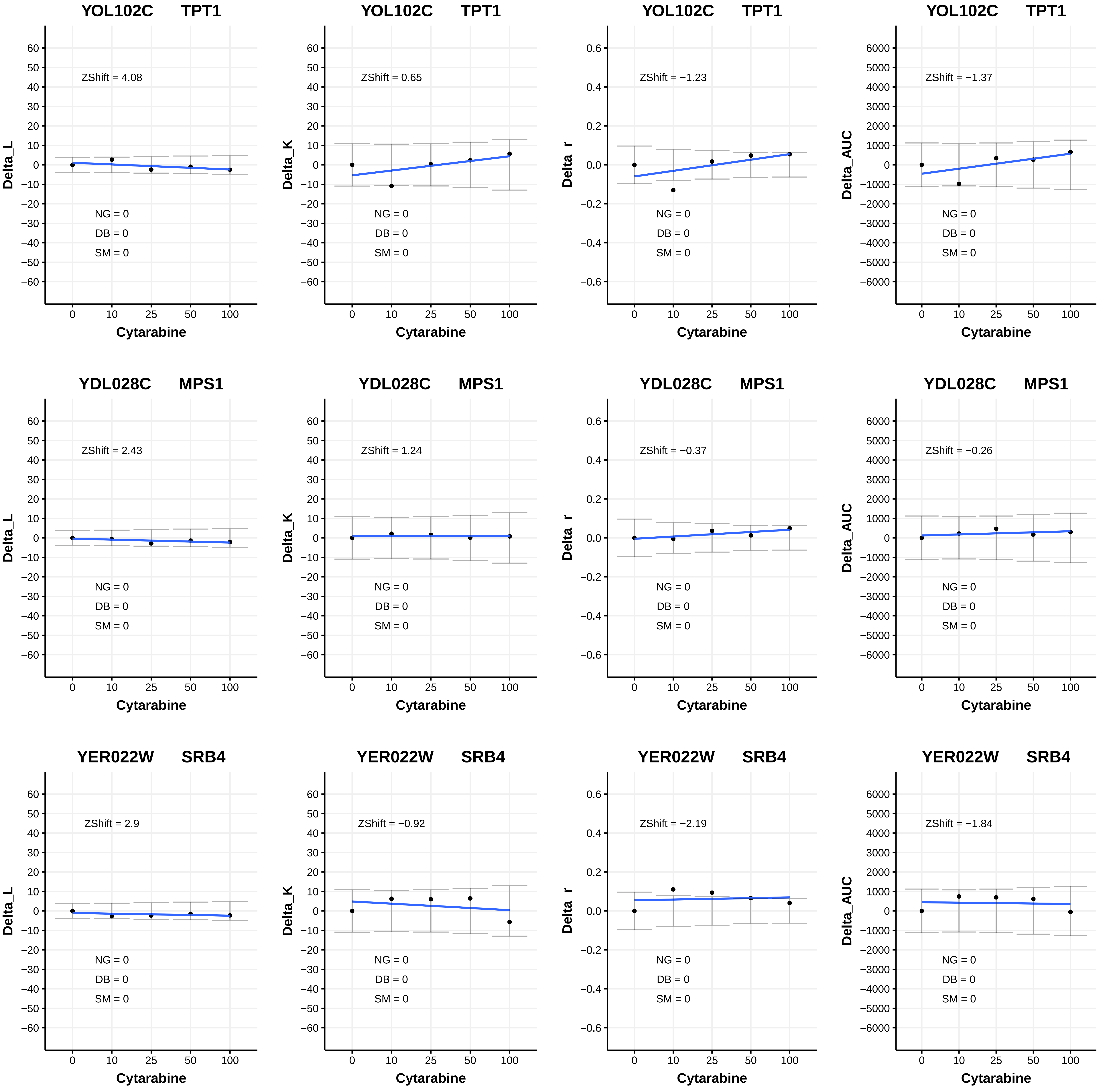
YOL102C TPT1
ZShift = 4.08
ZShift = 0.65
ZShift = −1.23
ZShift = −1.37
YDL028C MPS1
ZShift = 2.43
ZShift = 1.24
ZShift = −0.37
ZShift = −0.26
YER022W SRB4
ZShift = 2.9
ZShift = −0.92
ZShift = −2.19
ZShift = −1.84
NG = 0
DB = 0
SM = 0
Delta_L
Delta_K
Delta_r
Delta_AUC
Cytarabine
0
10
25
50
100

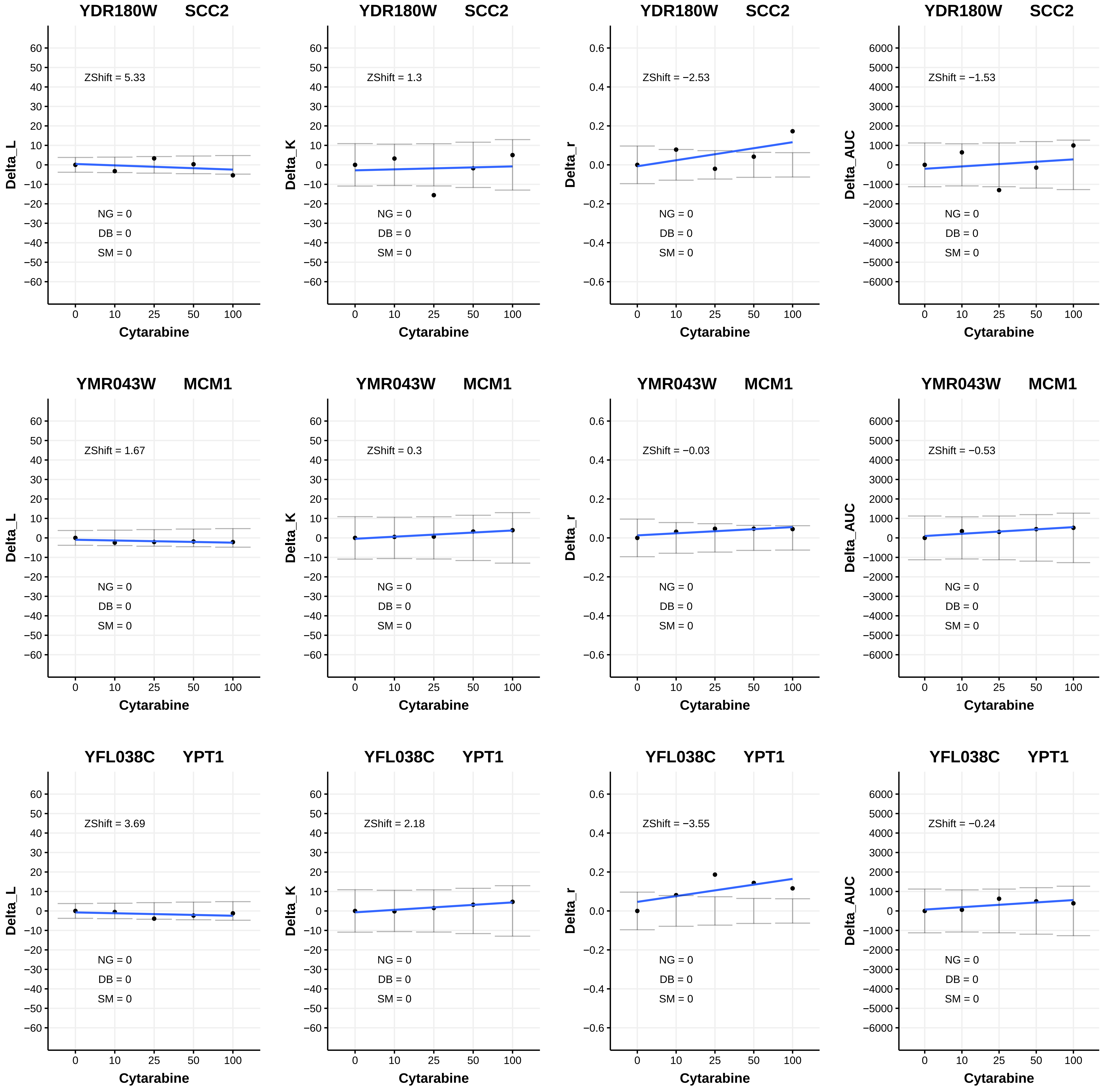
YDR180W SCC2
Delta_L
ZShift = 5.33
NG = 0
DB = 0
SM = 0
Cytarabine
YDR180W SCC2
Delta_K
ZShift = 1.3
NG = 0
DB = 0
SM = 0
Cytarabine
YDR180W SCC2
Delta_r
ZShift = −2.53
NG = 0
DB = 0
SM = 0
Cytarabine
YDR180W SCC2
Delta_AUC
ZShift = −1.53
NG = 0
DB = 0
SM = 0
Cytarabine
YMR043W MCM1
Delta_L
ZShift = 1.67
NG = 0
DB = 0
SM = 0
Cytarabine
YMR043W MCM1
Delta_K
ZShift = 0.3
NG = 0
DB = 0
SM = 0
Cytarabine
YMR043W MCM1
Delta_r
ZShift = −0.03
NG = 0
DB = 0
SM = 0
Cytarabine
YMR043W MCM1
Delta_AUC
ZShift = −0.53
NG = 0
DB = 0
SM = 0
Cytarabine
YFL038C YPT1
Delta_L
ZShift = 3.69
NG = 0
DB = 0
SM = 0
Cytarabine
YFL038C YPT1
Delta_K
ZShift = 2.18
NG = 0
DB = 0
SM = 0
Cytarabine
YFL038C YPT1
Delta_r
ZShift = −3.55
NG = 0
DB = 0
SM = 0
Cytarabine
YFL038C YPT1
Delta_AUC
ZShift = −0.24
NG = 0
DB = 0
SM = 0
Cytarabine

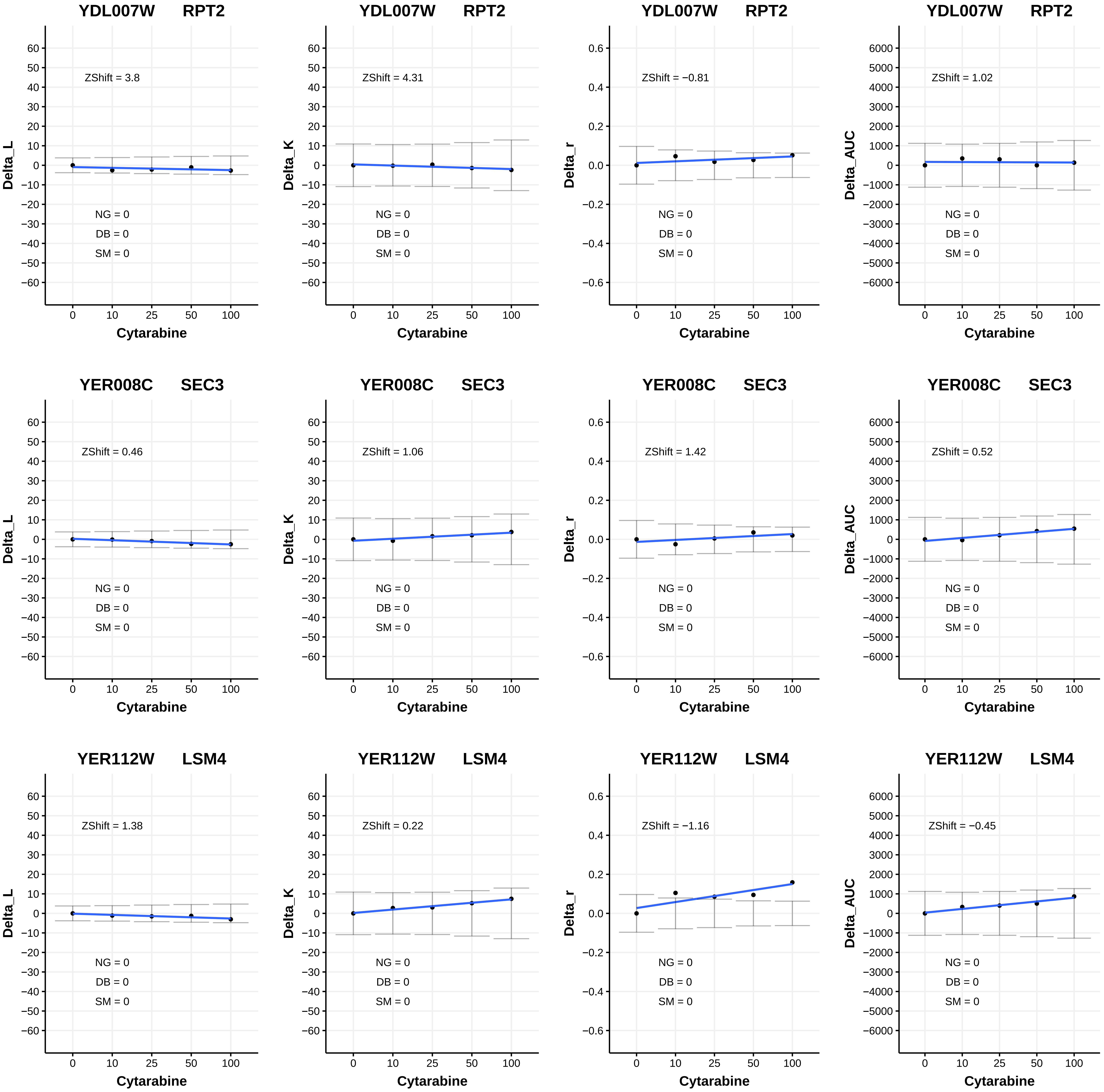
YDL007W RPT2
ZShift = 3.8
NG = 0
DB = 0
SM = 0
Delta_L
Cytarabine
YDL007W RPT2
ZShift = 4.31
NG = 0
DB = 0
SM = 0
Delta_K
Cytarabine
YDL007W RPT2
ZShift = −0.81
NG = 0
DB = 0
SM = 0
Delta_r
Cytarabine
YDL007W RPT2
ZShift = 1.02
NG = 0
DB = 0
SM = 0
Delta_AUC
Cytarabine
YER008C SEC3
ZShift = 0.46
NG = 0
DB = 0
SM = 0
Delta_L
Cytarabine
YER008C SEC3
ZShift = 1.06
NG = 0
DB = 0
SM = 0
Delta_K
Cytarabine
YER008C SEC3
ZShift = 1.42
NG = 0
DB = 0
SM = 0
Delta_r
Cytarabine
YER008C SEC3
ZShift = 0.52
NG = 0
DB = 0
SM = 0
Delta_AUC
Cytarabine
YER112W LSM4
ZShift = 1.38
NG = 0
DB = 0
SM = 0
Delta_L
Cytarabine
YER112W LSM4
ZShift = 0.22
NG = 0
DB = 0
SM = 0
Delta_K
Cytarabine
YER112W LSM4
ZShift = −1.16
NG = 0
DB = 0
SM = 0
Delta_r
Cytarabine
YER112W LSM4
ZShift = −0.45
NG = 0
DB = 0
SM = 0
Delta_AUC
Cytarabine

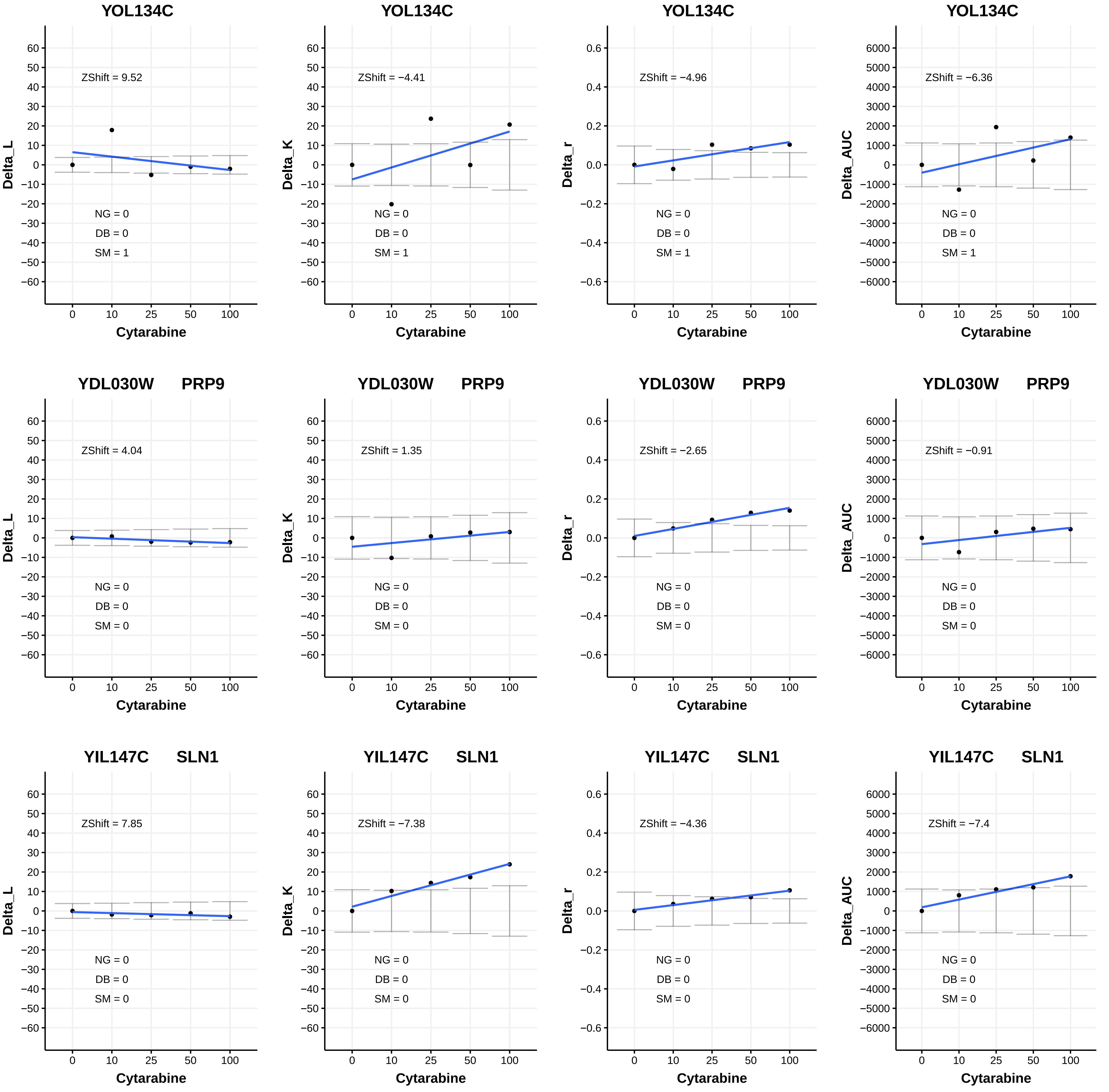
YOL134C
ZShift = 9.52
NG = 0
DB = 0
SM = 1
Delta_L
Cytarabine
YOL134C
ZShift = −4.41
NG = 0
DB = 0
SM = 1
Delta_K
Cytarabine
YOL134C
ZShift = −4.96
NG = 0
DB = 0
SM = 1
Delta_r
Cytarabine
YOL134C
ZShift = −6.36
NG = 0
DB = 0
SM = 1
Delta_AUC
Cytarabine
YDL030W PRP9
ZShift = 4.04
NG = 0
DB = 0
SM = 0
Delta_L
Cytarabine
YDL030W PRP9
ZShift = 1.35
NG = 0
DB = 0
SM = 0
Delta_K
Cytarabine
YDL030W PRP9
ZShift = −2.65
NG = 0
DB = 0
SM = 0
Delta_r
Cytarabine
YDL030W PRP9
ZShift = −0.91
NG = 0
DB = 0
SM = 0
Delta_AUC
Cytarabine
YIL147C SLN1
ZShift = 7.85
NG = 0
DB = 0
SM = 0
Delta_L
Cytarabine
YIL147C SLN1
ZShift = −7.38
NG = 0
DB = 0
SM = 0
Delta_K
Cytarabine
YIL147C SLN1
ZShift = −4.36
NG = 0
DB = 0
SM = 0
Delta_r
Cytarabine
YIL147C SLN1
ZShift = −7.4
NG = 0
DB = 0
SM = 0
Delta_AUC
Cytarabine
0 10 25 50 100

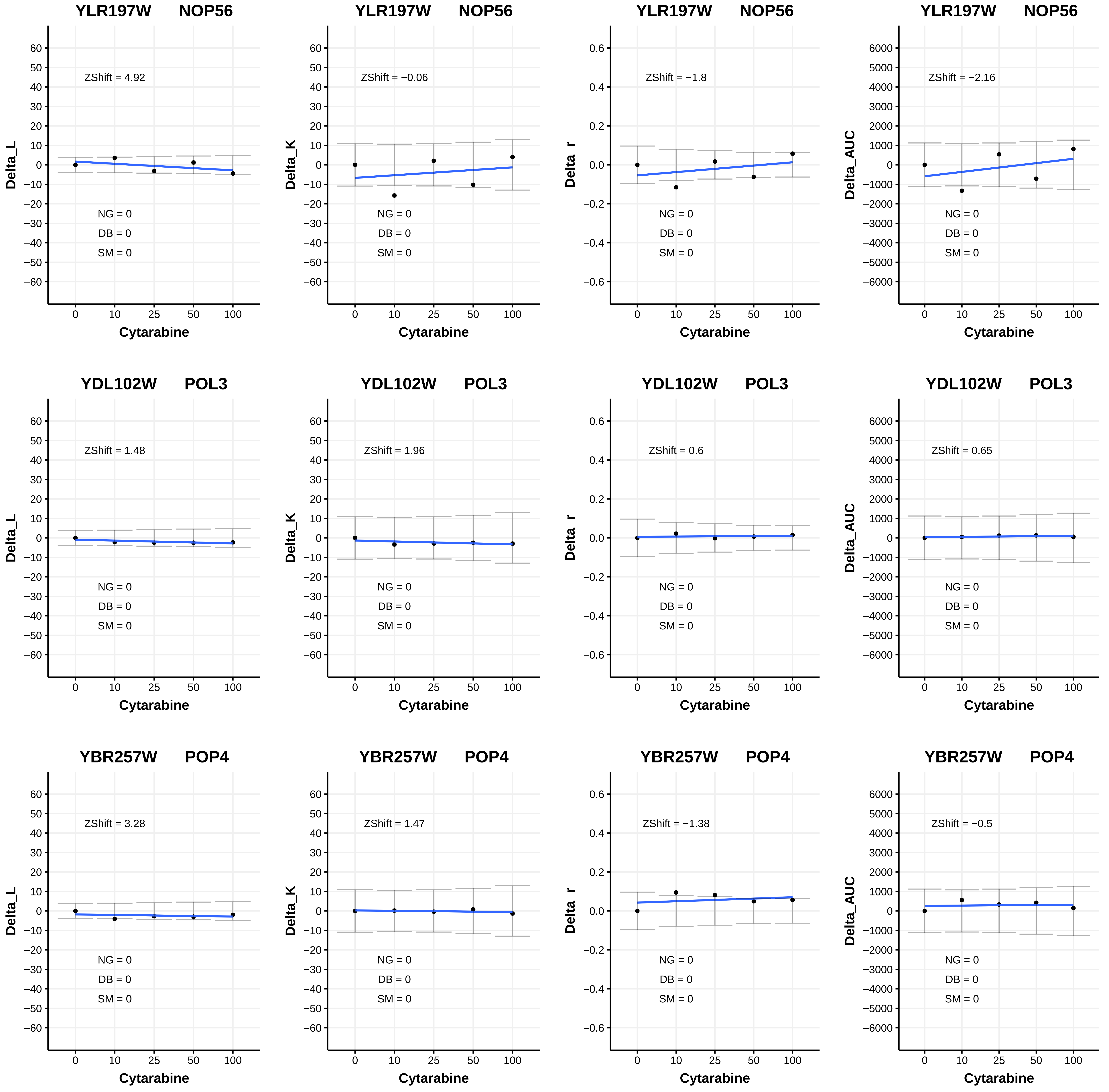
YLR197W NOP56
ZShift = 4.92
NG = 0
DB = 0
SM = 0
Delta_L
Cytarabine
YLR197W NOP56
ZShift = −0.06
NG = 0
DB = 0
SM = 0
Delta_K
Cytarabine
YLR197W NOP56
ZShift = −1.8
NG = 0
DB = 0
SM = 0
Delta_r
Cytarabine
YLR197W NOP56
ZShift = −2.16
NG = 0
DB = 0
SM = 0
Delta_AUC
Cytarabine
YDL102W POL3
ZShift = 1.48
NG = 0
DB = 0
SM = 0
Delta_L
Cytarabine
YDL102W POL3
ZShift = 1.96
NG = 0
DB = 0
SM = 0
Delta_K
Cytarabine
YDL102W POL3
ZShift = 0.6
NG = 0
DB = 0
SM = 0
Delta_r
Cytarabine
YDL102W POL3
ZShift = 0.65
NG = 0
DB = 0
SM = 0
Delta_AUC
Cytarabine
YBR257W POP4
ZShift = 3.28
NG = 0
DB = 0
SM = 0
Delta_L
Cytarabine
YBR257W POP4
ZShift = 1.47
NG = 0
DB = 0
SM = 0
Delta_K
Cytarabine
YBR257W POP4
ZShift = −1.38
NG = 0
DB = 0
SM = 0
Delta_r
Cytarabine
YBR257W POP4
ZShift = −0.5
NG = 0
DB = 0
SM = 0
Delta_AUC
Cytarabine

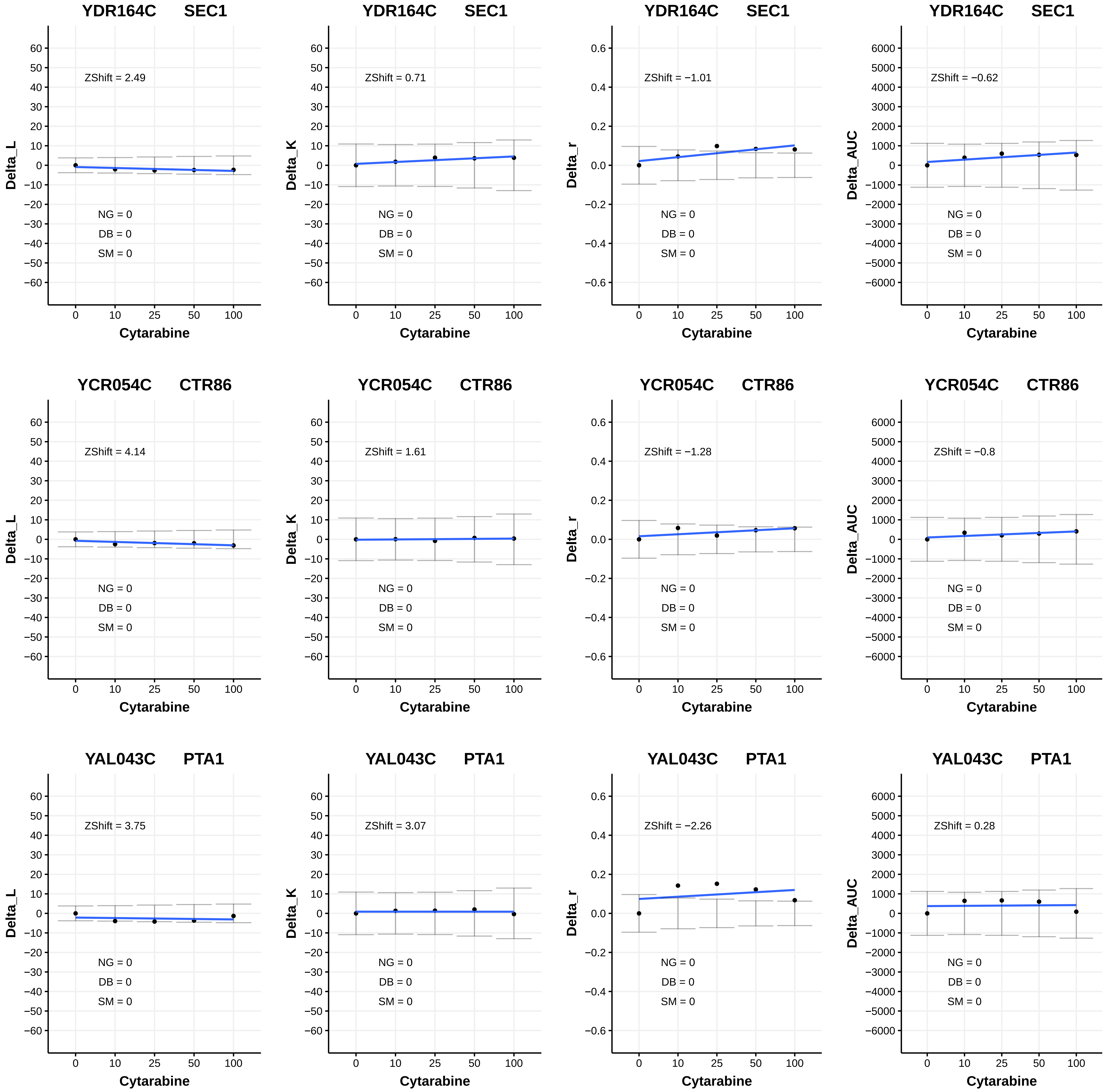

YDR164C SEC1
ZShift = 2.49
NG = 0
DB = 0
SM = 0
Delta_L
Cytarabine
YDR164C SEC1
ZShift = 0.71
NG = 0
DB = 0
SM = 0
Delta_K
Cytarabine
YDR164C SEC1
ZShift = −1.01
NG = 0
DB = 0
SM = 0
Delta_r
Cytarabine
YDR164C SEC1
ZShift = −0.62
NG = 0
DB = 0
SM = 0
Delta_AUC
Cytarabine
YCR054C CTR86
ZShift = 4.14
NG = 0
DB = 0
SM = 0
Delta_L
Cytarabine
YCR054C CTR86
ZShift = 1.61
NG = 0
DB = 0
SM = 0
Delta_K
Cytarabine
YCR054C CTR86
ZShift = −1.28
NG = 0
DB = 0
SM = 0
Delta_r
Cytarabine
YCR054C CTR86
ZShift = −0.8
NG = 0
DB = 0
SM = 0
Delta_AUC
Cytarabine
YAL043C PTA1
ZShift = 3.75
NG = 0
DB = 0
SM = 0
Delta_L
Cytarabine
YAL043C PTA1
ZShift = 3.07
NG = 0
DB = 0
SM = 0
Delta_K
Cytarabine
YAL043C PTA1
ZShift = −2.26
NG = 0
DB = 0
SM = 0
Delta_r
Cytarabine
YAL043C PTA1
ZShift = 0.28
NG = 0
DB = 0
SM = 0
Delta_AUC
Cytarabine
0
10
25
50
100

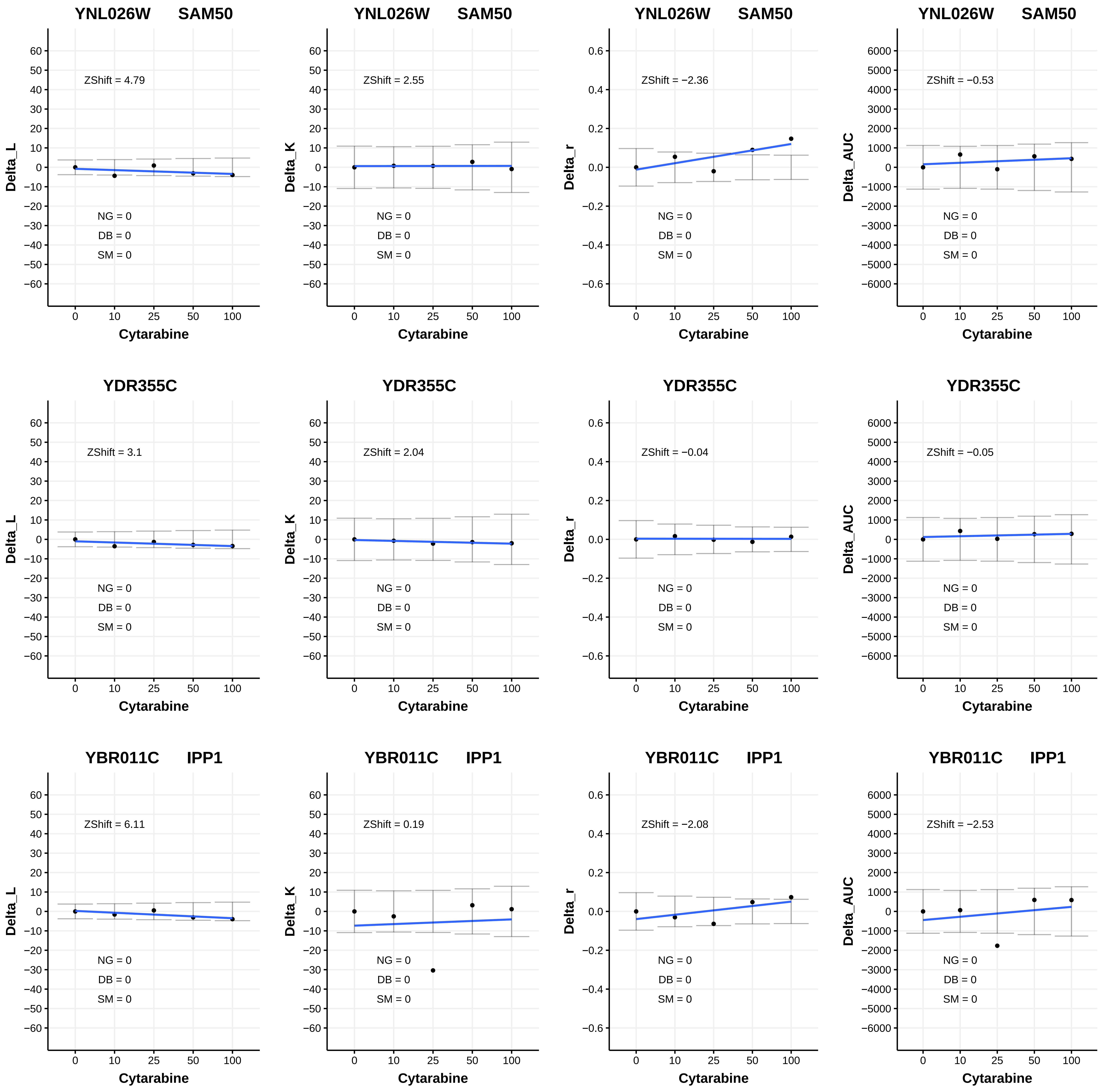
YNL026W SAM50
ZShift = 4.79
Delta_L
NG = 0
DB = 0
SM = 0
Cytarabine
YNL026W SAM50
ZShift = 2.55
Delta_K
NG = 0
DB = 0
SM = 0
Cytarabine
YNL026W SAM50
ZShift = −2.36
Delta_r
NG = 0
DB = 0
SM = 0
Cytarabine
YNL026W SAM50
ZShift = −0.53
Delta_AUC
NG = 0
DB = 0
SM = 0
Cytarabine
YDR355C
ZShift = 3.1
Delta_L
NG = 0
DB = 0
SM = 0
Cytarabine
YDR355C
ZShift = 2.04
Delta_K
NG = 0
DB = 0
SM = 0
Cytarabine
YDR355C
ZShift = −0.04
Delta_r
NG = 0
DB = 0
SM = 0
Cytarabine
YDR355C
ZShift = −0.05
Delta_AUC
NG = 0
DB = 0
SM = 0
Cytarabine
YBR011C IPP1
ZShift = 6.11
Delta_L
NG = 0
DB = 0
SM = 0
Cytarabine
YBR011C IPP1
ZShift = 0.19
Delta_K
NG = 0
DB = 0
SM = 0
Cytarabine
YBR011C IPP1
ZShift = −2.08
Delta_r
NG = 0
DB = 0
SM = 0
Cytarabine
YBR011C IPP1
ZShift = −2.53
Delta_AUC
NG = 0
DB = 0
SM = 0
Cytarabine
0
10
25
50
100

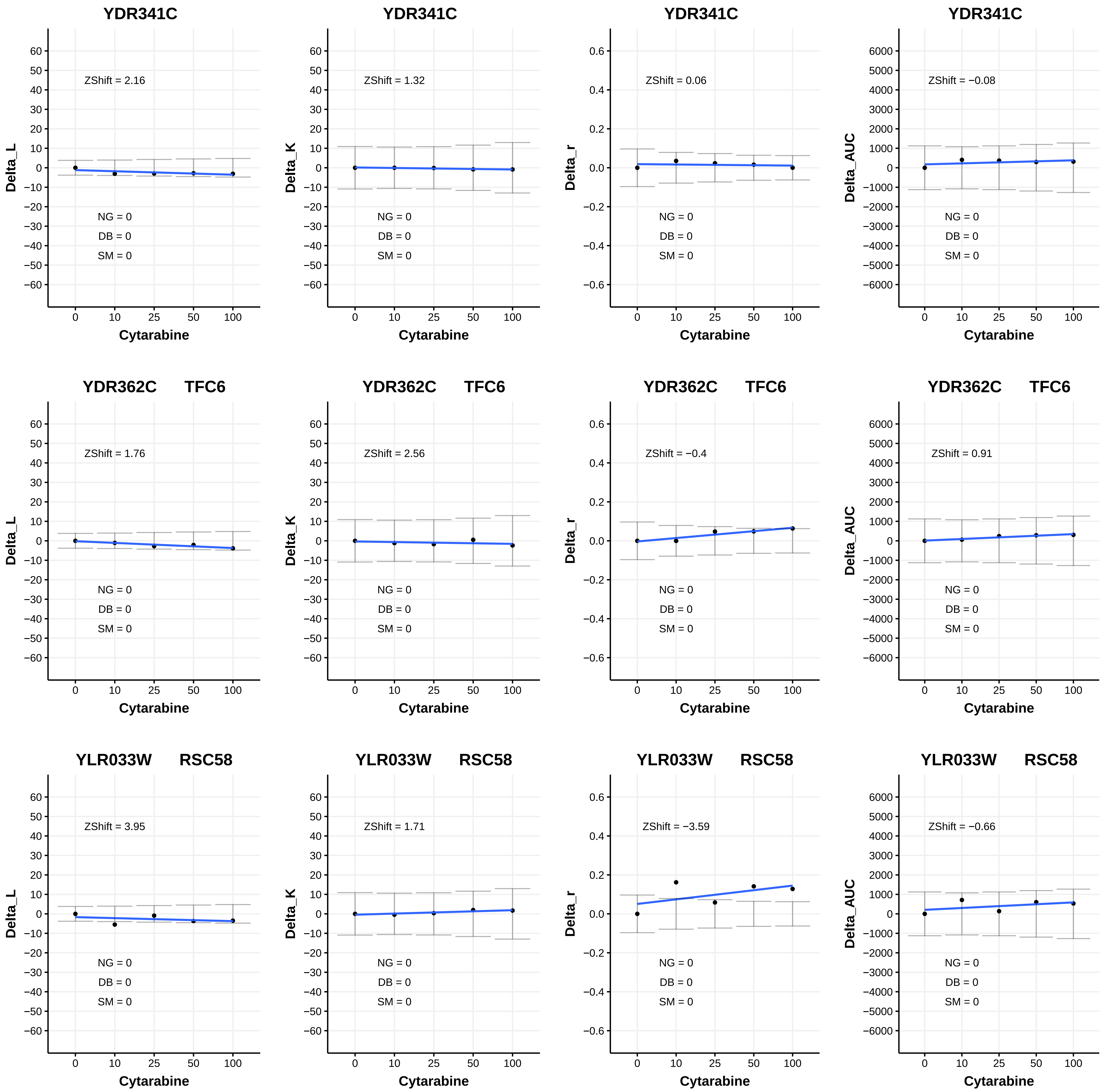
YDR341C
ZShift = 2.16
ZShift = 1.32
ZShift = 0.06
ZShift = −0.08
YDR362C TFC6
ZShift = 1.76
ZShift = 2.56
ZShift = −0.4
ZShift = 0.91
YLR033W RSC58
ZShift = 3.95
ZShift = 1.71
ZShift = −3.59
ZShift = −0.66
NG = 0
DB = 0
SM = 0
Delta_L
Delta_K
Delta_r
Delta_AUC
Cytarabine
0
10
25
50
100

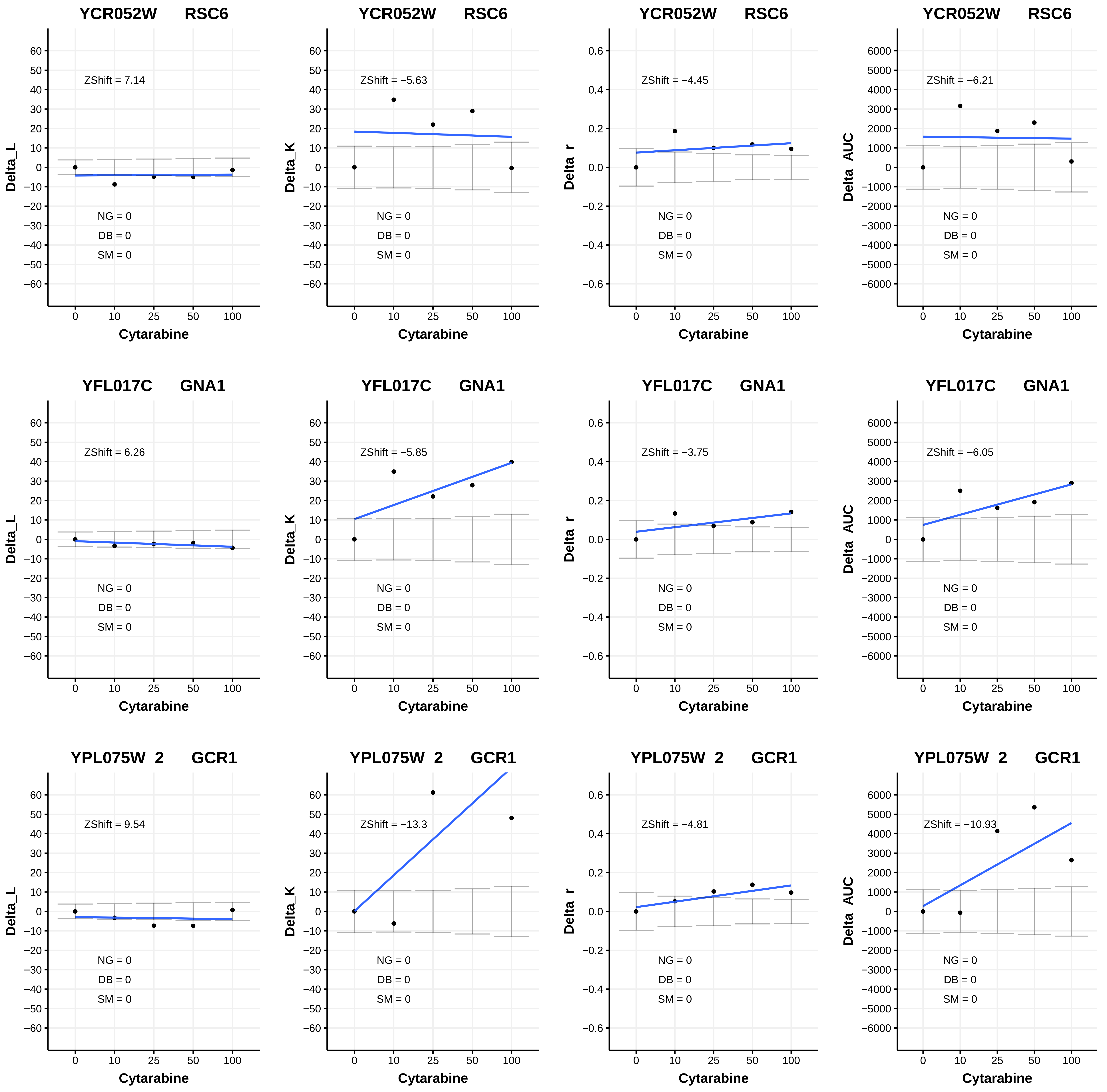

YCR052W RSC6
ZShift = 7.14
NG = 0
DB = 0
SM = 0
Delta_L
Cytarabine
YCR052W RSC6
ZShift = −5.63
NG = 0
DB = 0
SM = 0
Delta_K
Cytarabine
YCR052W RSC6
ZShift = −4.45
NG = 0
DB = 0
SM = 0
Delta_r
Cytarabine
YCR052W RSC6
ZShift = −6.21
NG = 0
DB = 0
SM = 0
Delta_AUC
Cytarabine
YFL017C GNA1
ZShift = 6.26
NG = 0
DB = 0
SM = 0
Delta_L
Cytarabine
YFL017C GNA1
ZShift = −5.85
NG = 0
DB = 0
SM = 0
Delta_K
Cytarabine
YFL017C GNA1
ZShift = −3.75
NG = 0
DB = 0
SM = 0
Delta_r
Cytarabine
YFL017C GNA1
ZShift = −6.05
NG = 0
DB = 0
SM = 0
Delta_AUC
Cytarabine
YPL075W_2 GCR1
ZShift = 9.54
NG = 0
DB = 0
SM = 0
Delta_L
Cytarabine
YPL075W_2 GCR1
ZShift = −13.3
NG = 0
DB = 0
SM = 0
Delta_K
Cytarabine
YPL075W_2 GCR1
ZShift = −4.81
NG = 0
DB = 0
SM = 0
Delta_r
Cytarabine
YPL075W_2 GCR1
ZShift = −10.93
NG = 0
DB = 0
SM = 0
Delta_AUC
Cytarabine

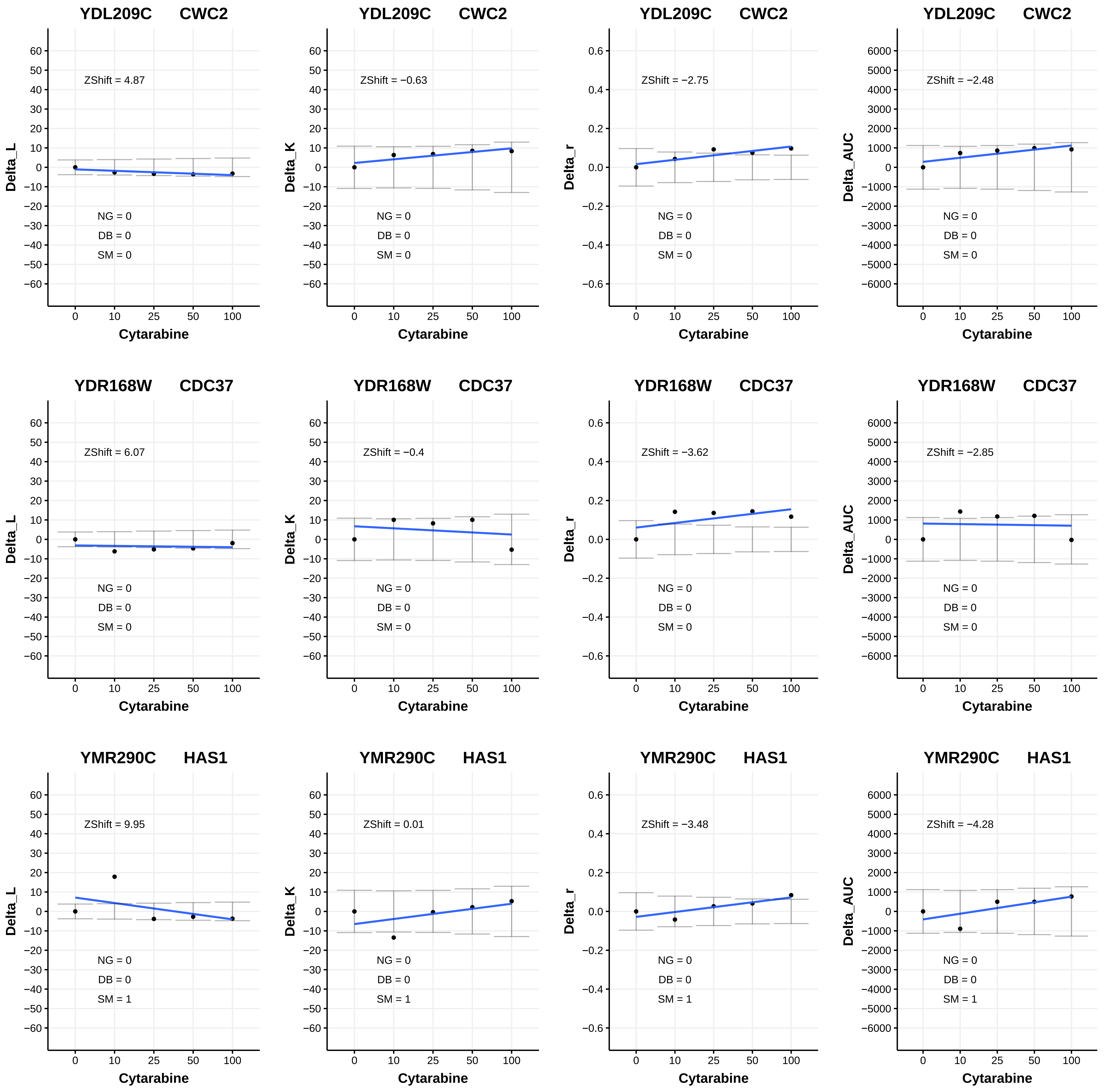
YDL209C CWC2
ZShift = 4.87
NG = 0
DB = 0
SM = 0
Delta_L
Cytarabine
YDL209C CWC2
ZShift = −0.63
NG = 0
DB = 0
SM = 0
Delta_K
Cytarabine
YDL209C CWC2
ZShift = −2.75
NG = 0
DB = 0
SM = 0
Delta_r
Cytarabine
YDL209C CWC2
ZShift = −2.48
NG = 0
DB = 0
SM = 0
Delta_AUC
Cytarabine
YDR168W CDC37
ZShift = 6.07
NG = 0
DB = 0
SM = 0
Delta_L
Cytarabine
YDR168W CDC37
ZShift = −0.4
NG = 0
DB = 0
SM = 0
Delta_K
Cytarabine
YDR168W CDC37
ZShift = −3.62
NG = 0
DB = 0
SM = 0
Delta_r
Cytarabine
YDR168W CDC37
ZShift = −2.85
NG = 0
DB = 0
SM = 0
Delta_AUC
Cytarabine
YMR290C HAS1
ZShift = 9.95
NG = 0
DB = 0
SM = 1
Delta_L
Cytarabine
YMR290C HAS1
ZShift = 0.01
NG = 0
DB = 0
SM = 1
Delta_K
Cytarabine
YMR290C HAS1
ZShift = −3.48
NG = 0
DB = 0
SM = 1
Delta_r
Cytarabine
YMR290C HAS1
ZShift = −4.28
NG = 0
DB = 0
SM = 1
Delta_AUC
Cytarabine

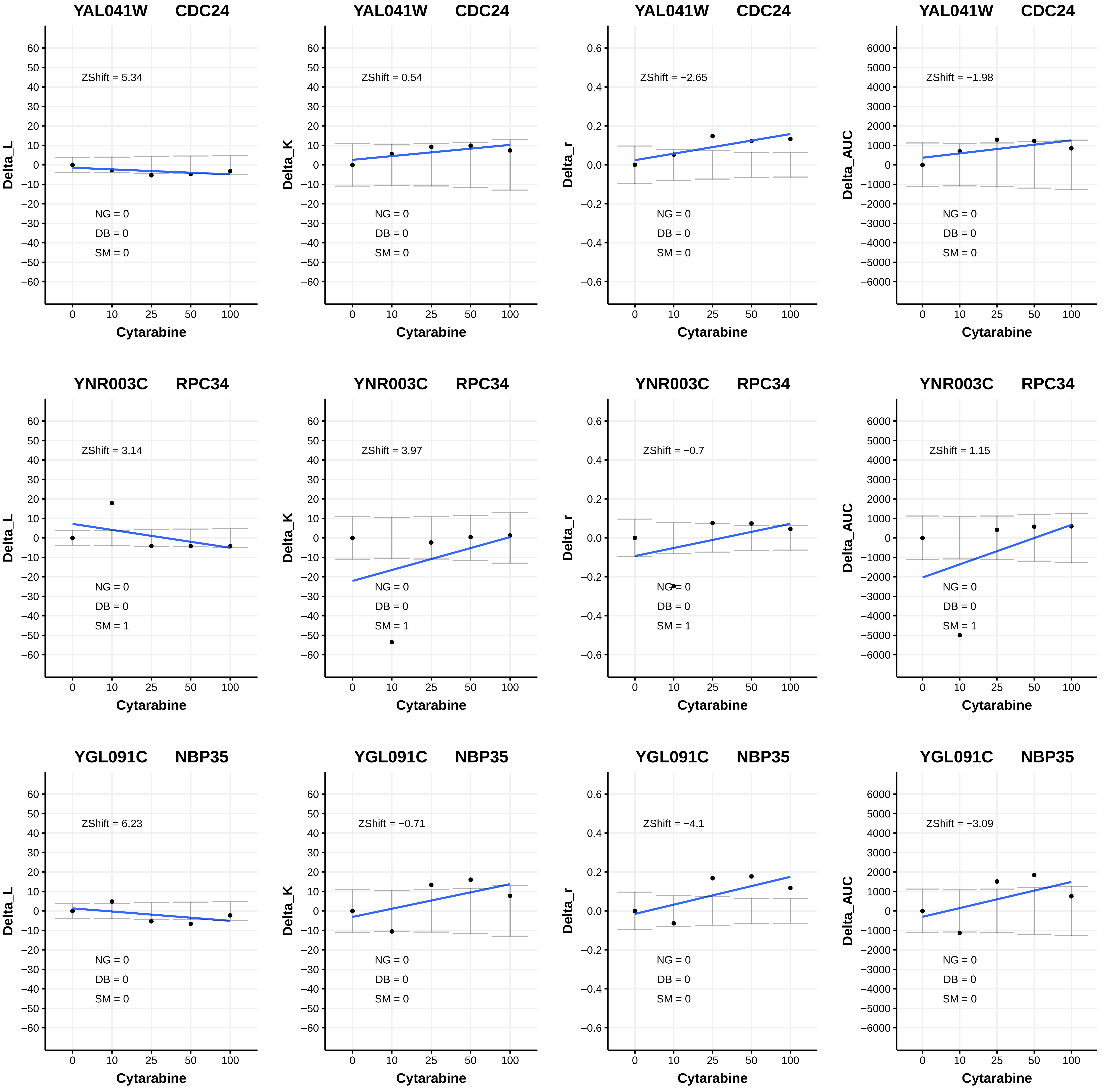
YAL041W CDC24
ZShift = 5.34
NG = 0
DB = 0
SM = 0
Delta_L
Cytarabine
YAL041W CDC24
ZShift = 0.54
NG = 0
DB = 0
SM = 0
Delta_K
Cytarabine
YAL041W CDC24
ZShift = −2.65
NG = 0
DB = 0
SM = 0
Delta_r
Cytarabine
YAL041W CDC24
ZShift = −1.98
NG = 0
DB = 0
SM = 0
Delta_AUC
Cytarabine
YNR003C RPC34
ZShift = 3.14
NG = 0
DB = 0
SM = 1
Delta_L
Cytarabine
YNR003C RPC34
ZShift = 3.97
NG = 0
DB = 0
SM = 1
Delta_K
Cytarabine
YNR003C RPC34
ZShift = −0.7
NG = 0
DB = 0
SM = 1
Delta_r
Cytarabine
YNR003C RPC34
ZShift = 1.15
NG = 0
DB = 0
SM = 1
Delta_AUC
Cytarabine
YGL091C NBP35
ZShift = 6.23
NG = 0
DB = 0
SM = 0
Delta_L
Cytarabine
YGL091C NBP35
ZShift = −0.71
NG = 0
DB = 0
SM = 0
Delta_K
Cytarabine
YGL091C NBP35
ZShift = −4.1
NG = 0
DB = 0
SM = 0
Delta_r
Cytarabine
YGL091C NBP35
ZShift = −3.09
NG = 0
DB = 0
SM = 0
Delta_AUC
Cytarabine

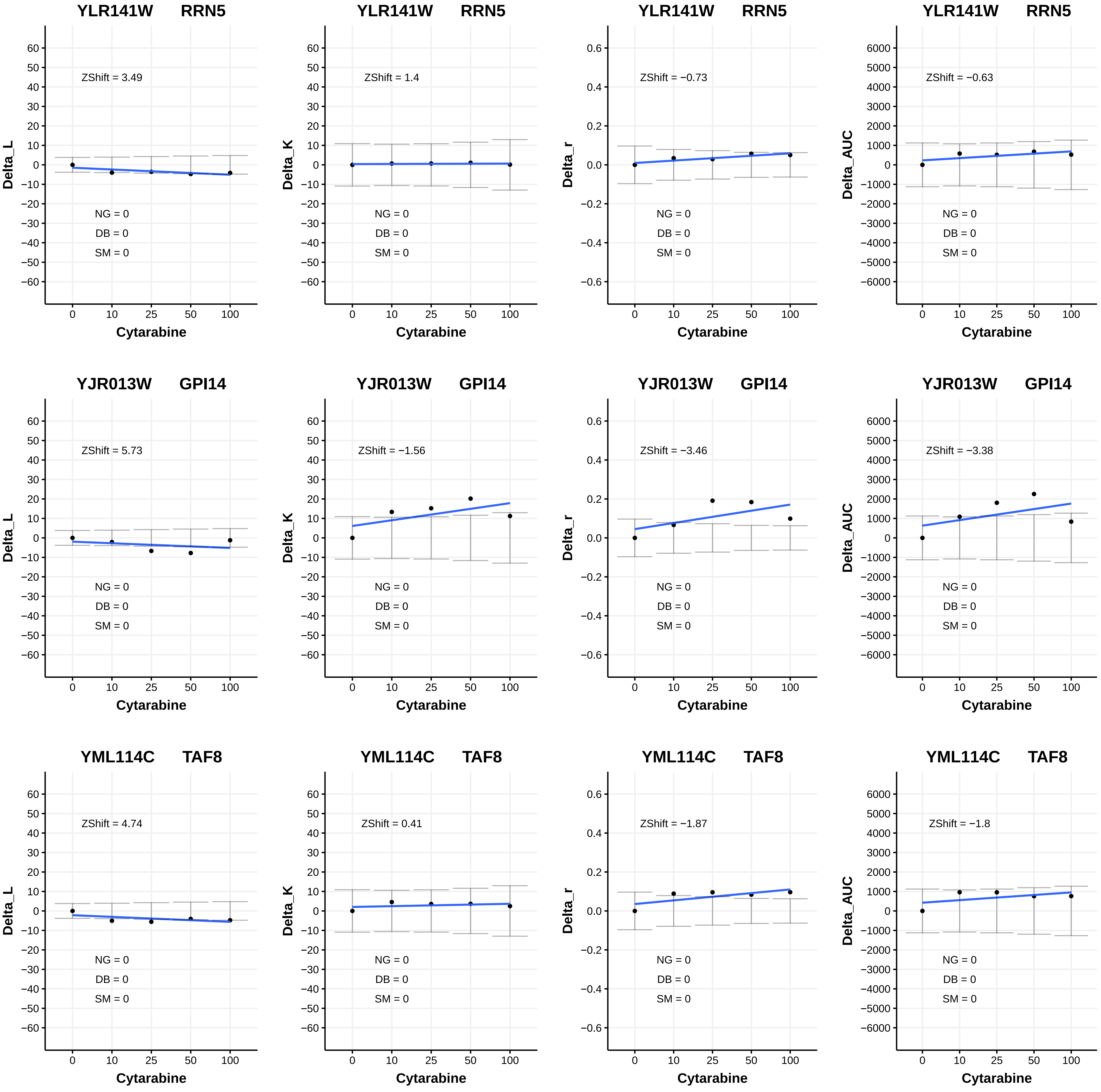
YLR141W RRN5
ZShift = 3.49
NG = 0
DB = 0
SM = 0
Delta_L
Cytarabine
YLR141W RRN5
ZShift = 1.4
NG = 0
DB = 0
SM = 0
Delta_K
Cytarabine
YLR141W RRN5
ZShift = −0.73
NG = 0
DB = 0
SM = 0
Delta_r
Cytarabine
YLR141W RRN5
ZShift = −0.63
NG = 0
DB = 0
SM = 0
Delta_AUC
Cytarabine
YJR013W GPI14
ZShift = 5.73
NG = 0
DB = 0
SM = 0
Delta_L
Cytarabine
YJR013W GPI14
ZShift = −1.56
NG = 0
DB = 0
SM = 0
Delta_K
Cytarabine
YJR013W GPI14
ZShift = −3.46
NG = 0
DB = 0
SM = 0
Delta_r
Cytarabine
YJR013W GPI14
ZShift = −3.38
NG = 0
DB = 0
SM = 0
Delta_AUC
Cytarabine
YML114C TAF8
ZShift = 4.74
NG = 0
DB = 0
SM = 0
Delta_L
Cytarabine
YML114C TAF8
ZShift = 0.41
NG = 0
DB = 0
SM = 0
Delta_K
Cytarabine
YML114C TAF8
ZShift = −1.87
NG = 0
DB = 0
SM = 0
Delta_r
Cytarabine
YML114C TAF8
ZShift = −1.8
NG = 0
DB = 0
SM = 0
Delta_AUC
Cytarabine
0
10
25
50
100

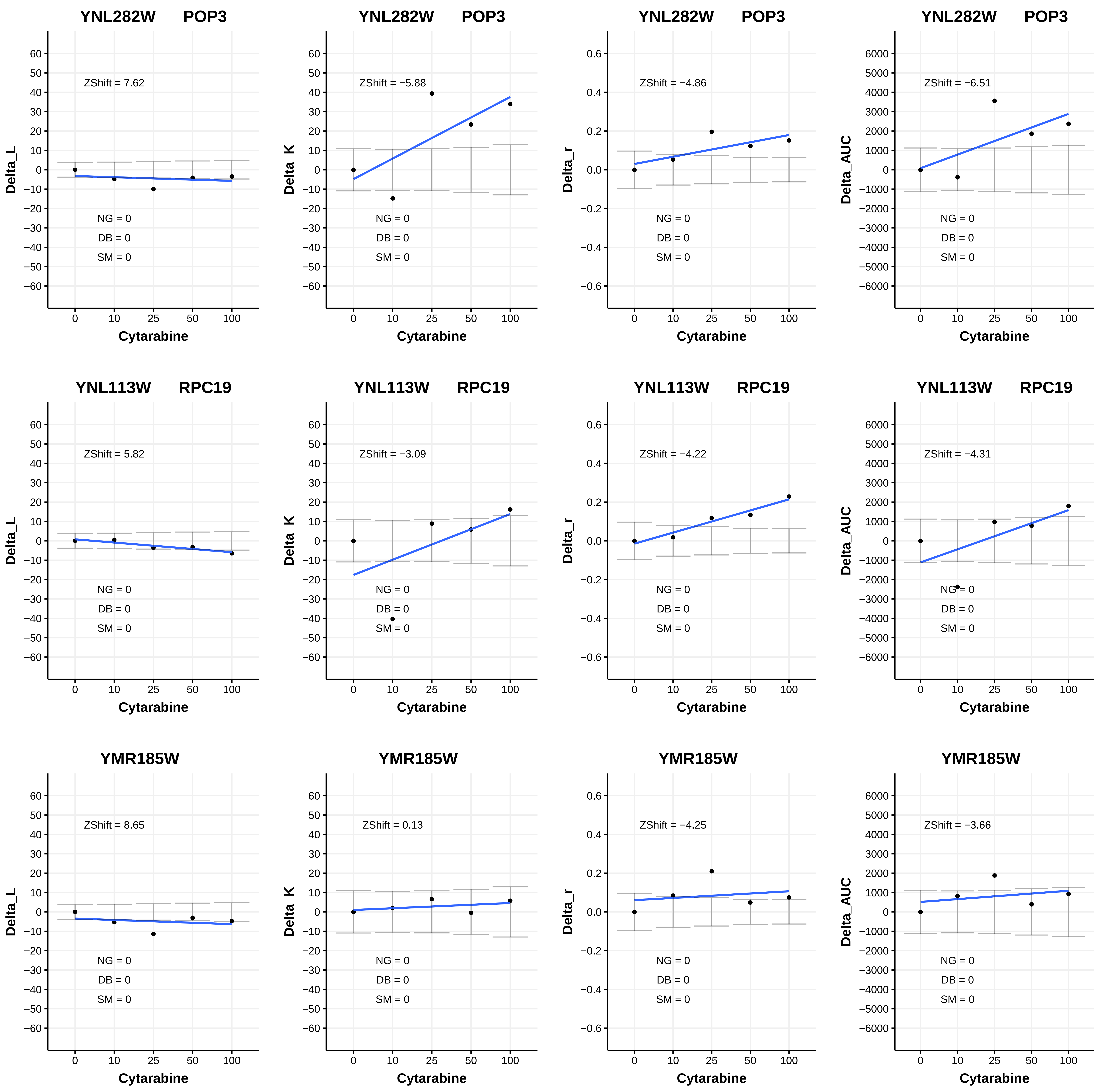
YNL282W POP3
ZShift = 7.62
NG = 0
DB = 0
SM = 0
Delta_L
Cytarabine
YNL282W POP3
ZShift = −5.88
NG = 0
DB = 0
SM = 0
Delta_K
Cytarabine
YNL282W POP3
ZShift = −4.86
NG = 0
DB = 0
SM = 0
Delta_r
Cytarabine
YNL282W POP3
ZShift = −6.51
NG = 0
DB = 0
SM = 0
Delta_AUC
Cytarabine
YNL113W RPC19
ZShift = 5.82
NG = 0
DB = 0
SM = 0
Delta_L
Cytarabine
YNL113W RPC19
ZShift = −3.09
NG = 0
DB = 0
SM = 0
Delta_K
Cytarabine
YNL113W RPC19
ZShift = −4.22
NG = 0
DB = 0
SM = 0
Delta_r
Cytarabine
YNL113W RPC19
ZShift = −4.31
NG = 0
DB = 0
SM = 0
Delta_AUC
Cytarabine
YMR185W
ZShift = 8.65
NG = 0
DB = 0
SM = 0
Delta_L
Cytarabine
YMR185W
ZShift = 0.13
NG = 0
DB = 0
SM = 0
Delta_K
Cytarabine
YMR185W
ZShift = −4.25
NG = 0
DB = 0
SM = 0
Delta_r
Cytarabine
YMR185W
ZShift = −3.66
NG = 0
DB = 0
SM = 0
Delta_AUC
Cytarabine

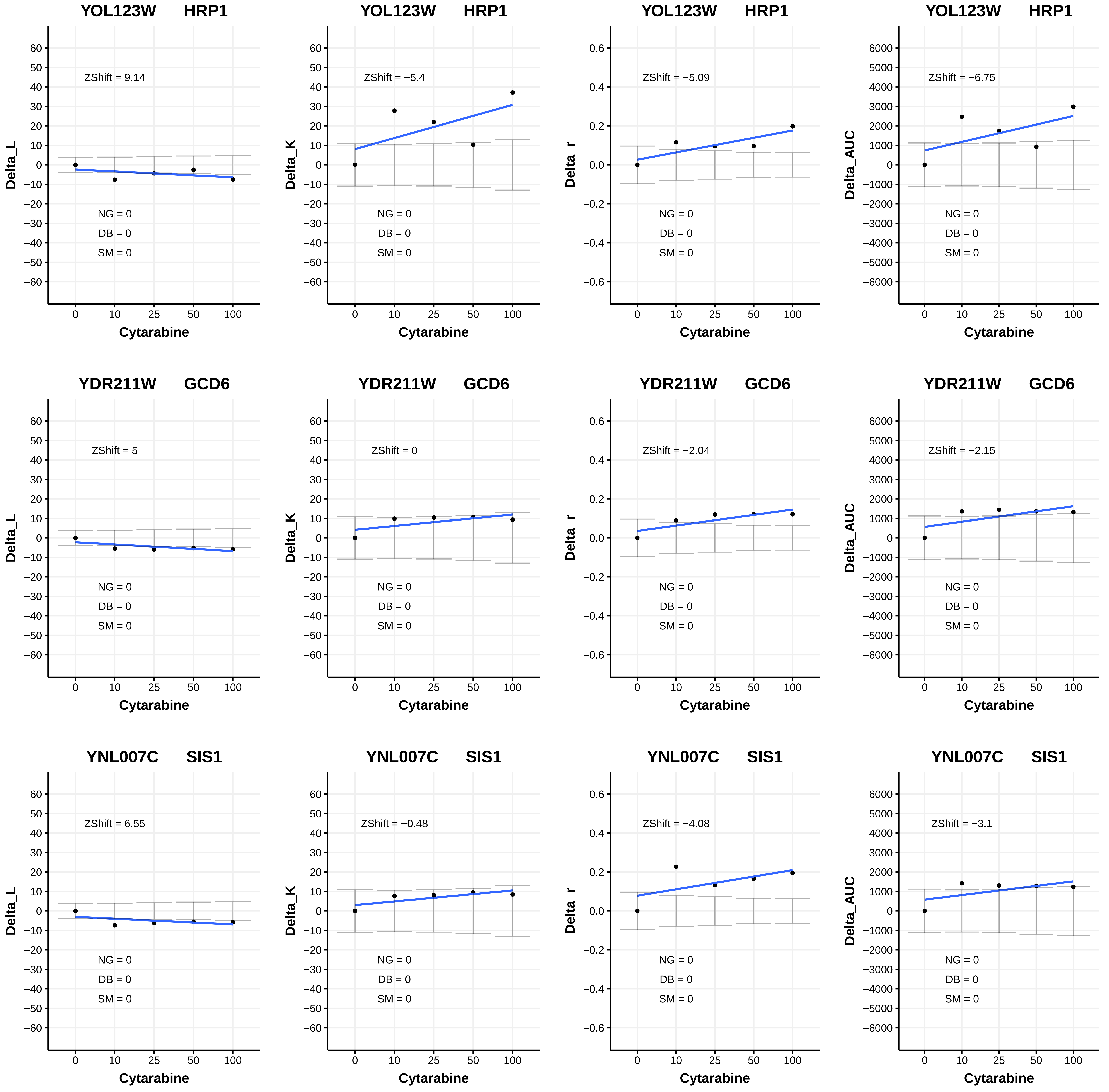
YOL123W HRP1
ZShift = 9.14
ZShift = −5.4
ZShift = −5.09
ZShift = −6.75
YDR211W GCD6
ZShift = 5
ZShift = 0
ZShift = −2.04
ZShift = −2.15
YNL007C SIS1
ZShift = 6.55
ZShift = −0.48
ZShift = −4.08
ZShift = −3.1
NG = 0
DB = 0
SM = 0
Delta_L
Delta_K
Delta_r
Delta_AUC
Cytarabine

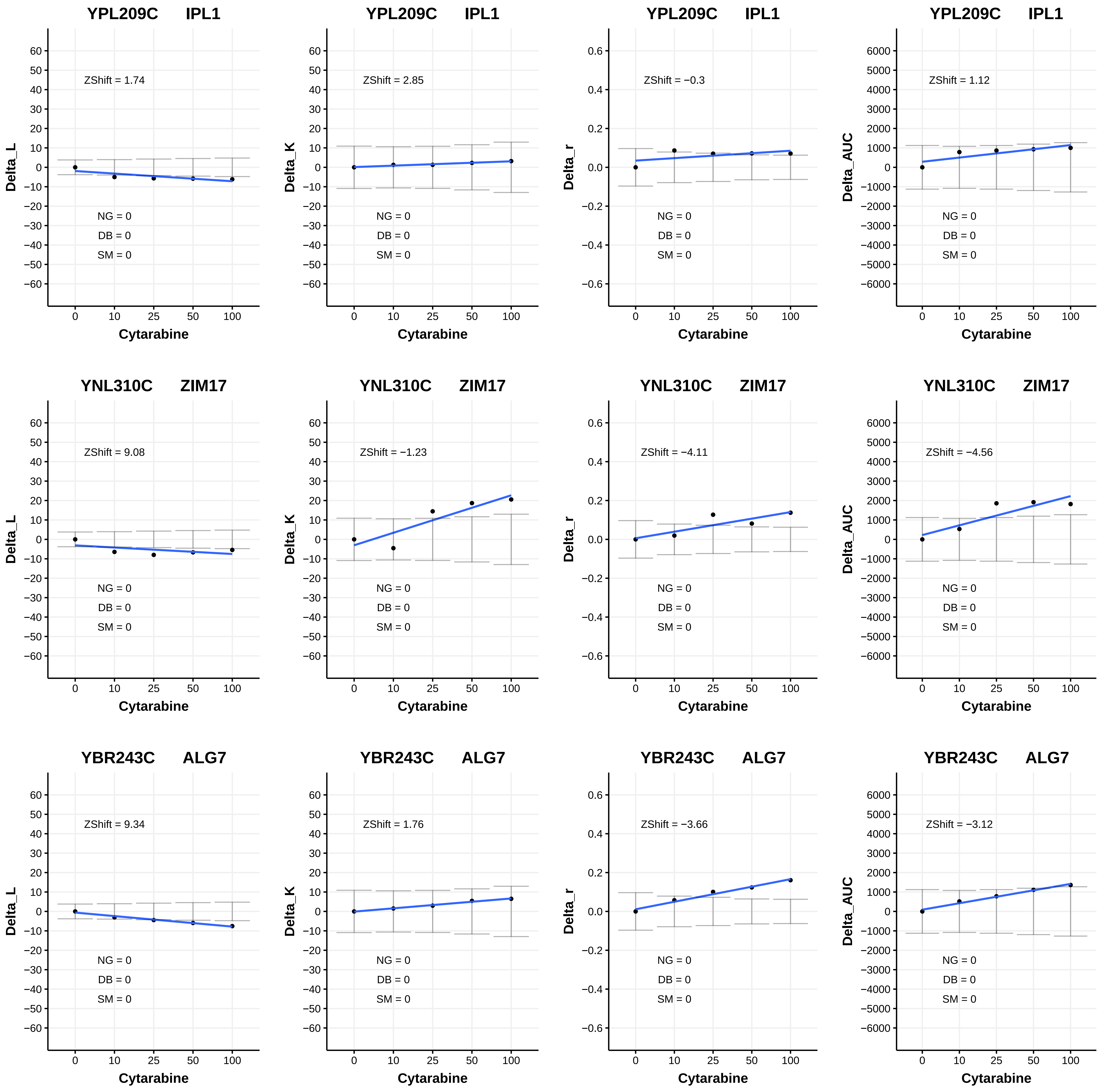
YPL209C IPL1
ZShift = 1.74
NG = 0
DB = 0
SM = 0
Delta_L
Cytarabine
YPL209C IPL1
ZShift = 2.85
NG = 0
DB = 0
SM = 0
Delta_K
Cytarabine
YPL209C IPL1
ZShift = −0.3
NG = 0
DB = 0
SM = 0
Delta_r
Cytarabine
YPL209C IPL1
ZShift = 1.12
NG = 0
DB = 0
SM = 0
Delta_AUC
Cytarabine
YNL310C ZIM17
ZShift = 9.08
NG = 0
DB = 0
SM = 0
Delta_L
Cytarabine
YNL310C ZIM17
ZShift = −1.23
NG = 0
DB = 0
SM = 0
Delta_K
Cytarabine
YNL310C ZIM17
ZShift = −4.11
NG = 0
DB = 0
SM = 0
Delta_r
Cytarabine
YNL310C ZIM17
ZShift = −4.56
NG = 0
DB = 0
SM = 0
Delta_AUC
Cytarabine
YBR243C ALG7
ZShift = 9.34
NG = 0
DB = 0
SM = 0
Delta_L
Cytarabine
YBR243C ALG7
ZShift = 1.76
NG = 0
DB = 0
SM = 0
Delta_K
Cytarabine
YBR243C ALG7
ZShift = −3.66
NG = 0
DB = 0
SM = 0
Delta_r
Cytarabine
YBR243C ALG7
ZShift = −3.12
NG = 0
DB = 0
SM = 0
Delta_AUC
Cytarabine

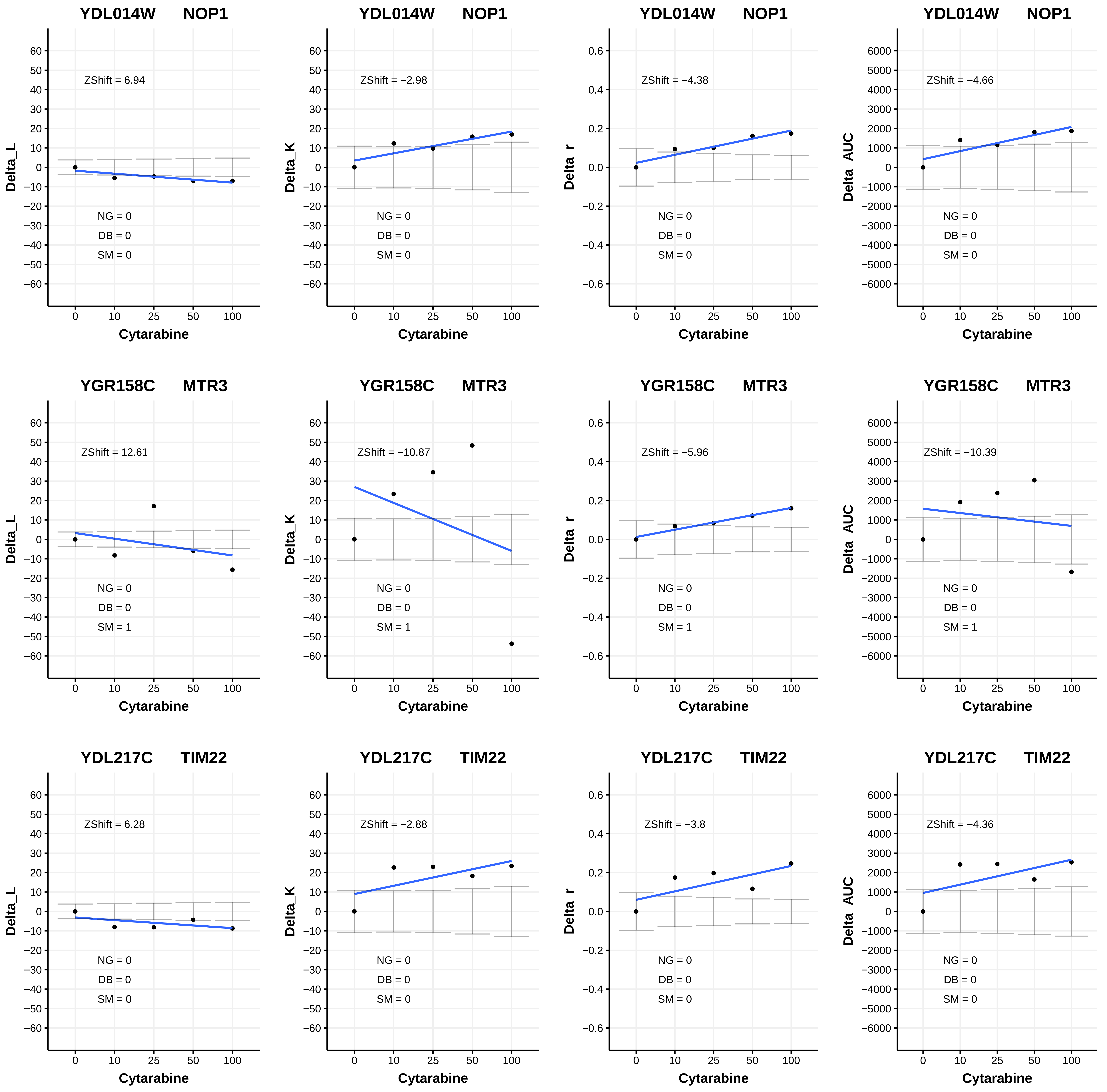
YDL014W NOP1
ZShift = 6.94
Delta_L
NG = 0
DB = 0
SM = 0
Cytarabine
YDL014W NOP1
ZShift = −2.98
Delta_K
NG = 0
DB = 0
SM = 0
Cytarabine
YDL014W NOP1
ZShift = −4.38
Delta_r
NG = 0
DB = 0
SM = 0
Cytarabine
YDL014W NOP1
ZShift = −4.66
Delta_AUC
NG = 0
DB = 0
SM = 0
Cytarabine
YGR158C MTR3
ZShift = 12.61
Delta_L
NG = 0
DB = 0
SM = 1
Cytarabine
YGR158C MTR3
ZShift = −10.87
Delta_K
NG = 0
DB = 0
SM = 1
Cytarabine
YGR158C MTR3
ZShift = −5.96
Delta_r
NG = 0
DB = 0
SM = 1
Cytarabine
YGR158C MTR3
ZShift = −10.39
Delta_AUC
NG = 0
DB = 0
SM = 1
Cytarabine
YDL217C TIM22
ZShift = 6.28
Delta_L
NG = 0
DB = 0
SM = 0
Cytarabine
YDL217C TIM22
ZShift = −2.88
Delta_K
NG = 0
DB = 0
SM = 0
Cytarabine
YDL217C TIM22
ZShift = −3.8
Delta_r
NG = 0
DB = 0
SM = 0
Cytarabine
YDL217C TIM22
ZShift = −4.36
Delta_AUC
NG = 0
DB = 0
SM = 0
Cytarabine
0 10 25 50 100

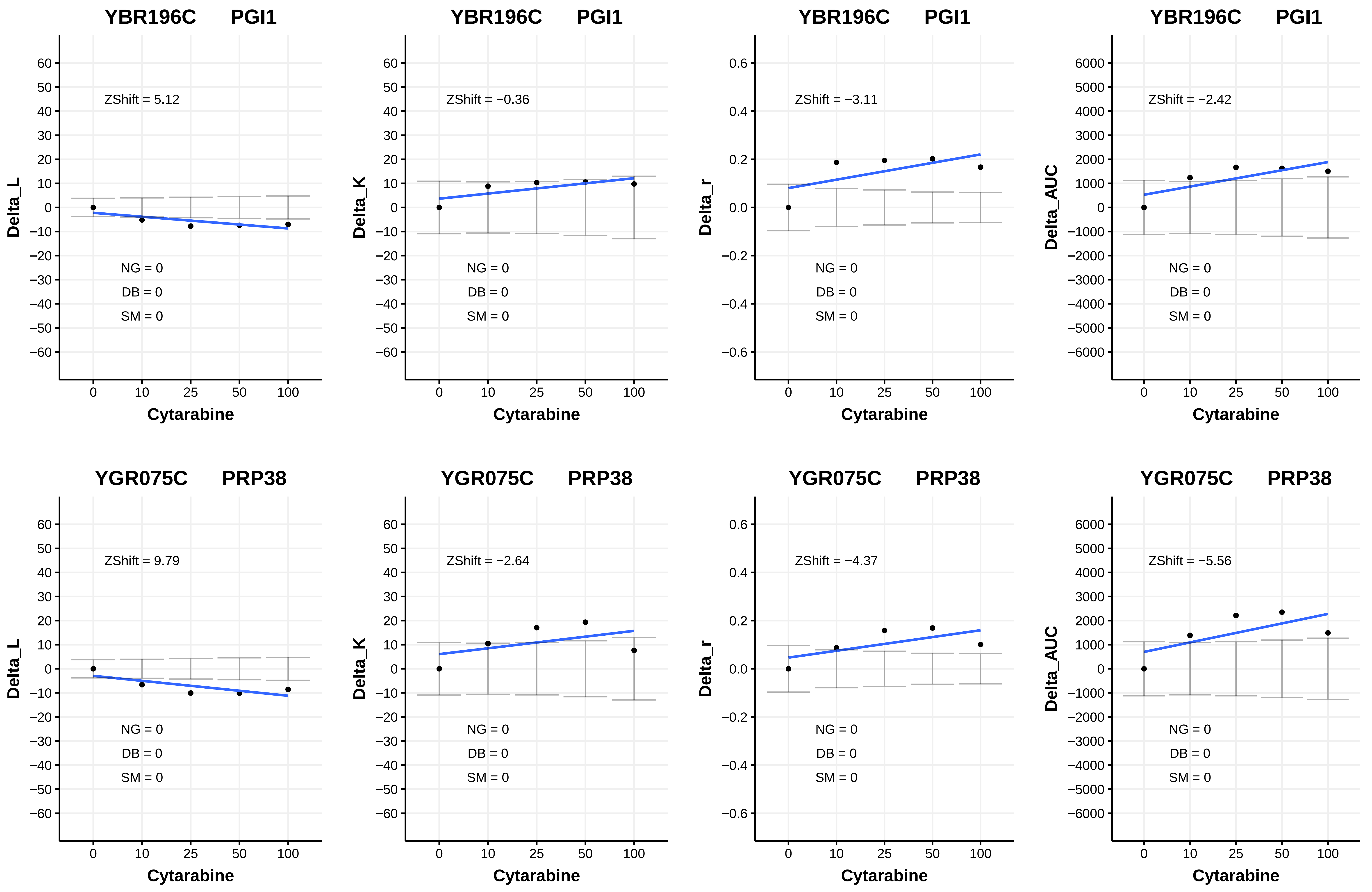
YBR196C PGI1
Delta_L
ZShift = 5.12
NG = 0
DB = 0
SM = 0
Cytarabine
YBR196C PGI1
Delta_K
ZShift = −0.36
NG = 0
DB = 0
SM = 0
Cytarabine
YBR196C PGI1
Delta_r
ZShift = −3.11
NG = 0
DB = 0
SM = 0
Cytarabine
YBR196C PGI1
Delta_AUC
ZShift = −2.42
NG = 0
DB = 0
SM = 0
Cytarabine
YGR075C PRP38
Delta_L
ZShift = 9.79
NG = 0
DB = 0
SM = 0
Cytarabine
YGR075C PRP38
Delta_K
ZShift = −2.64
NG = 0
DB = 0
SM = 0
Cytarabine
YGR075C PRP38
Delta_r
ZShift = −4.37
NG = 0
DB = 0
SM = 0
Cytarabine
YGR075C PRP38
Delta_AUC
ZShift = −5.56
NG = 0
DB = 0
SM = 0
Cytarabine
